# C−F bond activation enables synthesis of aryl difluoromethyl bicyclopentanes as benzophenone-type bioisosteres

**DOI:** 10.1038/s41467-023-44653-6

**Published:** 2024-01-10

**Authors:** Mingshuo Chen, Yuang Cui, Xiaoping Chen, Rui Shang, Xiaheng Zhang

**Affiliations:** 1 School of Chemistry and Materials Science, Hangzhou Institute for Advanced Study, University of Chinese Academy of Sciences, 1 Sub-lane Xiangshan, 310024 Hangzhou, People’s Republic of China; 2 Department of Chemistry, The University of Tokyo, Tokyo, 113-0033 Japan

**Keywords:** Reaction mechanisms, Synthetic chemistry methodology, Homogeneous catalysis

## Abstract

Bioisosteric design has become an essential approach in the development of drug molecules. Recent advancements in synthetic methodologies have enabled the rapid adoption of this strategy into drug discovery programs. Consequently, conceptionally innovative practices would be appreciated by the medicinal chemistry community. Here we report an expeditous synthetic method for synthesizing aryl difluoromethyl bicyclopentane (ADB) as a bioisostere of the benzophenone core. This approach involves the merger of light-driven C−F bond activation and strain-release chemistry under the catalysis of a newly designed *N*-anionic-based organic photocatalyst. This defluorinative coupling methodology enables the direct conversion of a wide variety of commercially available trifluoromethylaromatic C−F bonds (more than 70 examples) into the corresponding difluoromethyl bicyclo[1.1.1]pentanes (BCP) arenes/difluoromethyl BCP boronates in a single step. The strategy can also be applied to [3.1.1]and [4.1.1]propellane systems, providing access to analogues with different geometries. Moreover, we have successfully used this protocol to rapidly prepare ADB-substituted analogues of the bioactive molecule Adiporon. Biological testing has shown that the ADB scaffold has the potential to enhance the pharmacological properties of benzophenone-type drug candidates.

## Abstract (web summary)

Bioisosteric design has become an essential approach during drug molecule discovery process. Here, the authors describe a strategy for defluorinative couplings of propellanes, enabling the synthesis of aryl difluoromethyl bicyclopentanes as benzophenone-type bioisosteres.

## Introduction

**Fig. 1:**
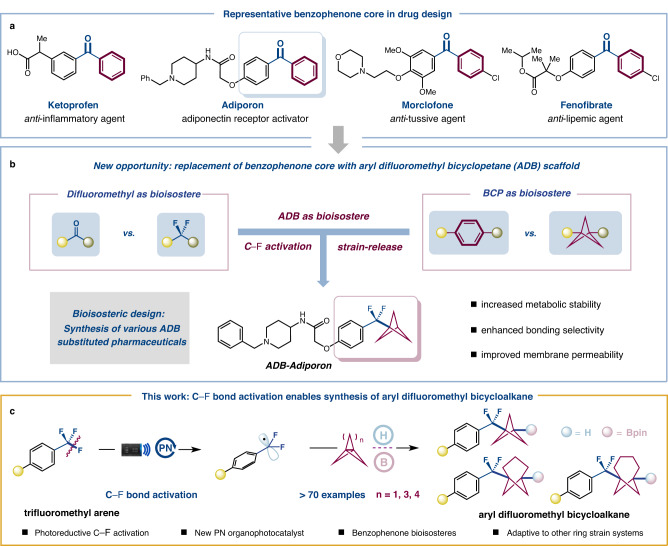
Development of a C–F bond activation strategy for the synthesis of benzophenone-type bioisosteres. **a** Representative benzophenone core in drug design. **b** Replacement of benzophenone core with aryl difluoromethyl bicyclopentane scaffold. **c** C**–**F bond activation enables the synthesis of aryl difluoromethyl bicycloalkanes (this work). BCP bicyclopentane, PN *N*-anionic-based photocatalyst, H hydrogen atom donor, B borylation reagent.

The replacement of a pharmaceutical core with a corresponding bioisostere scaffold has emerged as a useful strategy for modulating bioavailability and metabolic stability in the drug discovery process^1–11^. Consequently, the design of new bioisosteres has been adopted as a creative and effective manner to increase the potential for developing lead compounds and creating new drugs. Over the last few decades, one tactic that has been explored to improve metabolic stability while maintaining bioactivity is the substitution of the ketone functional group with a difluoromethylene moiety^6^. In addition, sp^3^-hybridized small-ring cage hydrocarbons, such as bicyclo- [1.1.1]pentane (BCP), have been recognized as an attractive bioisostere for benzene rings, due to their ability to improve pharmacokinetic properties^7–13^. While each of these bioisosteres has been extensively studied as a sole functional group isostere in drug development, their merger for the synthesis of difluoromethyl BCP arene as a new surrogate of the benzoyl group and the subsequent evaluation of their pharmacokinetic properties remain largely underexplored (Fig. 1b)^14,15^. Limited precedents have been reported for the synthesis of difluoromethyl BCPs, primarily relying on the use of activated precursor difluoroalkyl bromide^16,17^. For instance, the Dell’Amico group disclosed a single example of aryl difluoromethyl bicycloalkane synthesis using aryl difluoromethyl bromide as a coupling partner. However, these current methods lack generalizability across different substrate classes and have limited application to late-stage functionalization. This is because the corresponding alkyl/aryl difluoromethyl bromides are not widely accessible or cost-effective yet, which prevents their practical use by end users^18–20^. The benzophenone core is a crucial scaffold that occurs in numerous pharmaceuticals and natural products (Fig. 1a)^15,21–23^. Incorporating the ADB moiety as a new bioisostere would create significant opportunities to access a unique chemical space for benzophenone-type drug design. As such, new reaction designs for the rapid installation of the ADB from readily available starting materials (e.g., trifluoromethylaromatics), would be of considerable value in developing pharmaceuticals.

The C−F functionalization of trifluoromethylarenes (ArCF_3_) has been identified as an ideal strategy for synthesizing medicinally relevant ArCF_2_-containing compounds due to the abundance of trifluoromethylarenes and their prevalence in late-stage settings^18,24–37^. Photoredox catalysis has emerged as a valuable platform in recent years for the C**−**F bond activation processes, thereby providing access to difluorobenzylic motifs of importance in medicinal chemistry^38–53^. Recently, a series of impressive advancements have been reported by König^38^, Jui^39,40^, Gouverneur^41^, Molander^42,43^, Glorius^44^, Zhang^45^, Yasuda^46^, and others, utilizing single-electron reduction of the C**−**F bond. Typically, the generated aryl difluoromethyl radical is added across π-bond systems to form a new C**−**C bond^39,40,42–44,49–51^. Alternatively, we wondered whether it would be possible to intercept other types of bonds, such as the strained propellane system, with difluorobenzylic radicals generated directly from commercially available trifluoromethylarenes, a pathway that might provide a simple, direct protocol to access unique ADB scaffolds (Fig. 1c). Specifically, a single-electron reduction can initiate the selective activation of trifluoromethylarenes by using a reducing excited photocatalyst. The resulting difluorobenzylic radicals would then be trapped by [1.1.1]propellane leading to BCP radicals, which could be engaged in hydrogen atom transfer or borylation generating the desired difluoromethyl BCP arene/difluoromethyl BCP boronate scaffolds.

Several challenges must be addressed to achieve such a general defluorinative coupling transformation: (1) tuning the photocatalytic ability to enable the reductive activation of strong C**−**F bonds, regardless of their electronic substitution, despite typically high reductive potential^39–41^; (2) preventing overfunctionalization of the resulting difluoromethyl product as the strength of C**−**F bond decreases during defluorination;^40^ (3) selectively trapping the electronphilic difluorobenzylic radicals with propellane before being quenched by the hydrogen atom donor^40–42^; (4) controlling deleterious propellane oligomerization^54^. Herein, we report our successful efforts to develop an expedient route to ADB scaffold synthesis from readily accessible trifluoromethylarenes. A broad scope of trifluoromethylarenes coupling with [n.1.1]propellane systems has been demonstrated through two-component and three-component coupling (Fig. 1c). This method allowed for the rapid preparation of ADB analogs of known drugs, one of which is found to be more metabolically stable than its commercial progenitor. We anticipate this strategy would serve as a valuable tool for the synthesis and evaluation of ADB motifs as new bioisosteres of benzophenone-type drug derivatives, ultimately leading to the development of pharmaceuticals.

## Results

### Reaction optimization

Our investigation into this defluoronative coupling reaction began with the exposure of ArCF_3_ **5** to various photocatalysts in the presence of [1.1.1]propellane and *γ*-terpinene as hydrogen atom donors (Table 1). To our delight, the desired product, aryl difluoromethyl bicyclopetane **6**, was observed in 36% yield when *N*-phenylphenothiazine (**PTH**) was used as the photocatalyst, along with a ArCF_2_**–***di*BCP side-product **7**, that arose from propellane dimerization. The less reducing photocatalyst *fac*-Ir(ppy)_3_ led to a decreased yield of ArCF_2_**–**BCP **6** (6% yield). At this stage, we surmised that selecting the appropriate photocatalyst would be key to improving reaction efficiency. Indeed, a highly reducing *o*-phosphinophenolate (**PO**) photocatalyst was more efficient than **PTH** to increase the yield to 43%^50^. Based on previous studies on the effect of phosphine group facilitating photo-induced electron transfer processes^50^, we hypothesized that an *ortho*-PPh_2_ substituent on arylamine might have a similar effect to enable a redox cycle of strong reductive ability^55,56^. To this end, we designed and synthesized different types of *o*-phosphinoarylamines (**PNs**) as strongly reductive photocatalysts. Next, we evaluated their effectiveness in catalyzing the defluoroalkylation of **5**, a selection of which is shown in Table 1 (see Supplementary Fig. 14 for a list of all evaluated photocatalysts). The best results were obtained with carbazole-based catalyst **PCN** (**1**), a highly reducing photocatalyst (${E}_{1/2}^{{{\mbox{red}}}}[{\,\!}^\ast {{{\rm{N}}}}^{-}{{{\rm{P}}}}/{{{\mbox{P}}}}{{{\mbox{N}}}}^{{{{\boldsymbol{\bullet }}}}}]=-3.26{{{\mbox{V}}}}$ vs. the saturated calomel electrode (SCE) in DMSO) (see Supplementary Information for measurement), which provided the desired product **6** in 74% yield (entry 1). Structurally related **PCN** (**2**) also catalyzed the defluoroalkylation but with diminished yield (55% yield). We considered that the accelerated intersystem crossing process to access triplet state of long lifetime and steric protection by introducing the second adjacent**–**PPh_2_ account for the improved catalyst efficiency. Interestingly, 2-(diphenylphosphino)biphenyl amine **PBN** (**3**), a potential ligand for transition metal catalysis, was also effective to catalyze the reaction giving a 48% yield. In contrast, the structurally similar phenyl-protected secondary amine **PBN** (**4**) was less effective (12% yield). Unfortunately, dimethyl-protected tertiary amine ***di*Me-PBN** (**I**) and biphenyl amine **BN** (**II**) without *ortho*-PPh_2_ substitution, proved to be ineffective at all. These results suggest that both of the *N*-anion and *ortho*-PPh_2_ substituent are crucial for the photocatalytic reactivity. Increasing the amount of [1.1.1]propellane to 3.0 or 5.0 equiv. resulted in a significant decrease in reaction efficiencies due to the unproductive propellane dimerization leading to dimer **7**(entries 2−3). Finally, control experiments revealed that the photocatalyst, base and visible-light were all essential for the success of this transformation (entries 5−7). The reaction proceeded poorly in the absence of *γ*-terpinene (entry 4). Catalyst **PCN** (**1**) is an air-stable white solid. Of note, it can be prepared on gram-scale and stored under ambient conditions for months without observing any decomposition.

**Table 1:**
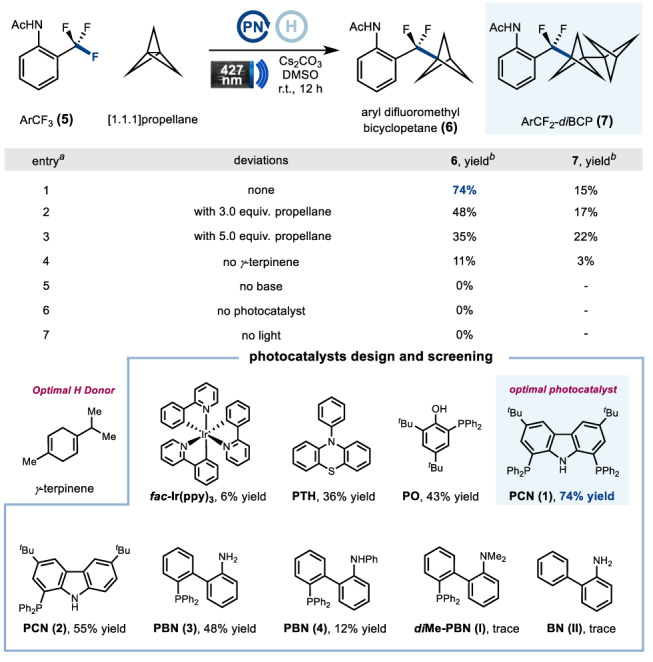
Optimization of the defluorinative coupling of ArCF_3_ with [1.1.1]propellane

^a^Performed with photocatalyst **PCN (1)** (10 mol%), trifluomethyl arene (**5**) (1.0 equiv.), [1.1.1]propellane in DMF (1.2 M, 1.5 equiv.), *γ*-terpinene (5.0 equiv.), Cs_2_CO_3_ (1.2 equiv.).

^b^Yield by ^19^F-NMR analysis of the crude reaction mixtures using PhCF_3_ as internal standard

**Fig. 2:**
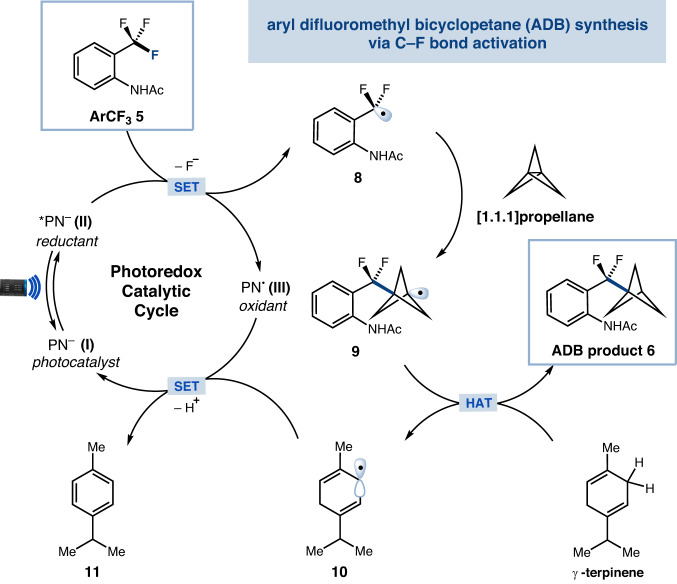
Proposed mechanism. Photocatalytic synthesis of aryl difluoromethyl bicyclopentane. SET single-electron transfer, HAT hydrogen atom transfer.

A plausible mechanism for the proposed defluoroalkylation of ArCF_3_ with [1.1.1]propellane is shown in Fig. 2. Visible-light photoexcitation of electron-rich *N*-anionic catalyst **I** would generate a highly reducing excited state **II**. Single-electron transfer to the trifluoromethylarene **5** (${E}_{1/2}^{{{\mbox{red}}}}{=-}2.16{{\mbox{V}}}$ vs. SCE in DMSO) (see Supplementary Information for measurement) gives rise to the corresponding difluorobenzylic radical **8**, which should be rapidly intercepted by [1.1.1]propellane to form the resulting BCP radical **9**. The electrophilic radical **9** would then abstract a hydrogen atom from *γ*-terpinene, thus enabling the desired ADB product **6** and the cyclohexadienyl radical **10**^57^. Finally, a single-electron transfer between **10** and oxidized photocatalyst **III** would regenerate photocatalyst **I**, thus completing the catalytic cycle.

### Reaction substrate scope

**Fig. 3:**
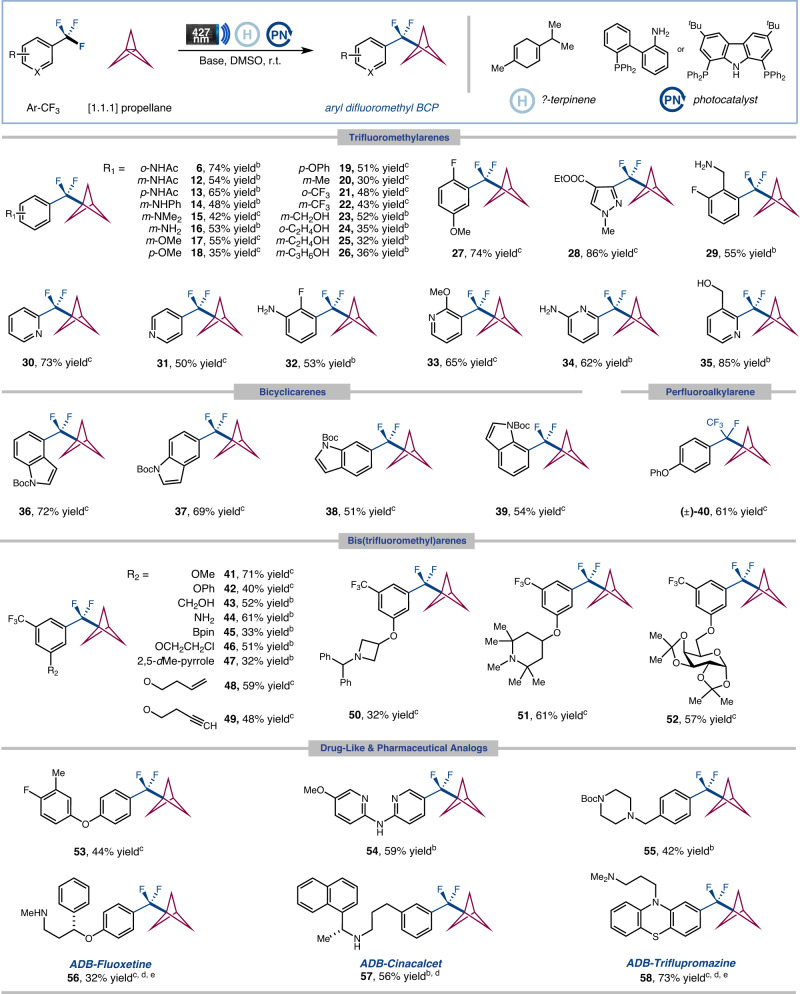
Scope of defluorinative coupling of ArCF_3_ with [1.1.1]propellane^*a*^. ^*a*^Isolated yields. General reaction conditions: trifluoroarenes (0.6 mmol, 1.0 equiv.), [1.1.1]propellane (1.5 equiv.), *γ*-terpinene (5.0 equiv.), photocatalyst (10 mol%), base (1.2 equiv.), DMSO (0.025 M), Kessil LEDs 427 nm (40 W) for 12 h. Selection of photocatalyst & base see Supplementary Information. ^*b*^**PCN (1)** as photocatalyst and Cs_2_CO_3_ as base. ^*c*^**PBN (3)** as photocatalyst and CsOH·H_2_O as base. ^*d*^Performed with trifluoroarenes hydrochloride (0.6 mmol, 1.0 equiv.), base (2.2 equiv.). ^*e*^[1.1.1]propellane (2.0 equiv.).

We next evaluated the generality of this defluorinative coupling reaction. As shown in Fig. 3, a myriad of unactivated trifluoromethylaromatics could be engaged under these conditions. A diverse range trifluoromethylarenes bearing electron-donating and electron-withdrawing substituents were found to be competent substrates (**6,** **12**–**29**, 30**–**86% yield). Notably, functional groups such as amides, ethers, esters, anilines, alkenes, alkynes, alkyl chlorides, aryl boronates, unprotected amines, and alcohols, were all well compatible, allowing for practical handles for further derivatization. Given the significance of nitrogen-containing heterocycles in bioactive molecule production, we were pleased to discover that a wide range of pyridine substrates with trifluoromethyl groups at 2-, 3-, and 4-positions were easily accommodated (**30**−**35**, 50−85% yield). Moreover, this reactivity could be extended to bicyclic heteroaromatics affording good yields (**36**−**39**, 51−72% yield). Particularly, perfluoroalkylarene was readily employed for selectively defluorinative coupling with [1.1.1]propellane (**40**, 61% yield). Additionally, bis(trifluoromethyl) benzenes yielded selective activation of only one of the C−F bonds giving synthetically useful yields (**41**−**52**, 32−71% yield). It is important to note that the corresponding ArCF_2_Br is not readily available and is challenging to synthesize^17–20^. This highlights the unique advantage of our approach in terms of accessibility and practicality in the synthesis of these important compounds compared with previous studies. Finally, the compatibility of the defluoroalkylation protocol with biorelevant molecules was also examined. To our delight, a large variety of biologically relevant systems were successfully employed to install the ADB moiety (**53**−**58**, 32−73% yield). It is worth noting that pharmaceutical analogs, including fluoxetine, cinacalcet, and triflupromazine, were effective substrates for the late-stage C−F functionalization, delivering the corresponding ADB adducts smoothly (**56**−**58**, 32−73% yield). These results further highlight the real-world utility of this defluorinative coupling technology.

**Fig. 4:**
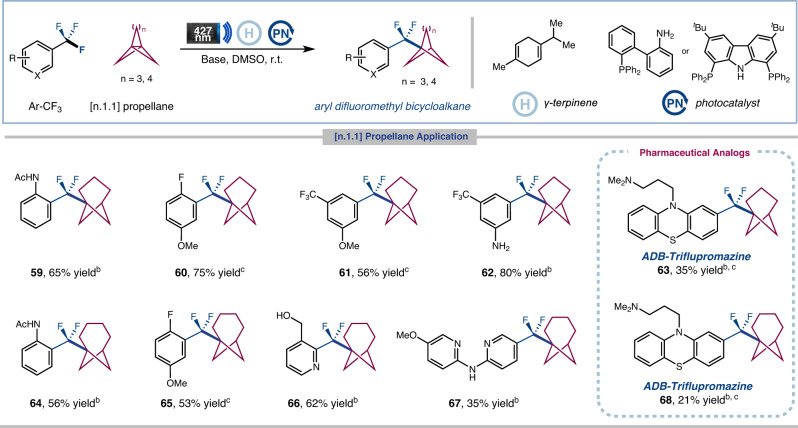
Scope of defluorinative coupling of ArCF_3_ with [3.1.1] and [4.1.1]propellane^*a*^. ^*a*^Isolated yields. General reaction conditions: trifluoroarenes (0.6 mmol, 1.0 equiv.), [n.1.1]propellane (2.0 equiv.), *γ*-terpinene (5.0 equiv.), photocatalyst (10 mol%), base (1.2 equiv.), DMSO (0.025 M), Kessil LEDs 427 nm (40 W) for 12 h. Selection of photocatalyst & base see Supplementary Information. ^*b*^**PCN (1)** as photocatalyst and Cs_2_CO_3_ as base. ^*c*^**PBN (3)** as photocatalyst and CsOH·H_2_O as base. ^*d*^Performed with trifluoroarenes hydrochloride (0.6 mmol, 1.0 equiv.), base (2.2 equiv.).

The installation of bicyclo[3.1.1]heptane (BCH) scaffolds as a bioisostere of *meta*-substituted benzenes in bioactive molecules has become an increasingly popular research area^58^. Pioneer works by Anderson^59^ and Uchiyama^60^ group have established a practical protocol for accessing functionalized BCHs employing a radical-based approach^61,62^. Gratifyingly, our protocol for this defluorinative coupling can be extended to [3.1.1]heptane system (Fig. 4). An array of trifluoromethylaromatics, including pharmaceutical agents, can be utilized to afford ArCF_2_−BCH products in moderate to good yields (**59**−**69**, 35−80% yield). Furthermore, the less-strained [4.1.1]octane system can also be employed in the same way to generate ArCF_2_−BCO products (**64**−**68**, 21−62% yield)^63^. To our knowledge, this is the first instance of the [4.1.1]octane system engaging in a photocatalytic radical addition process to date.

### Scope for three-component coupling

**Fig. 5:**
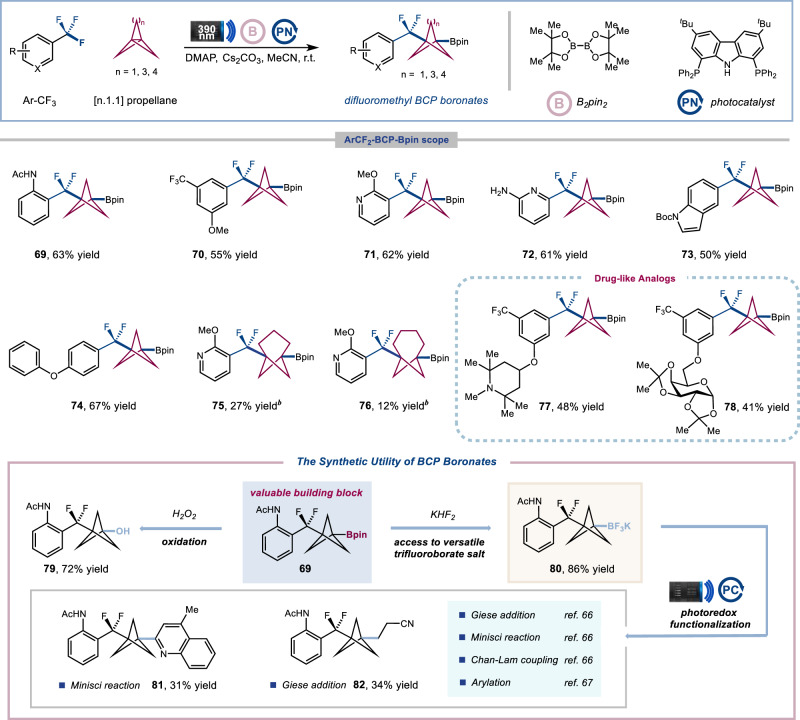
Scope for the defluorinative three-component coupling^*a*^. ^*a*^Isolated yields. General reaction conditions: trifluoromethylarenes (1.2 mmol, 1.0 equiv.), [n.1.1]propellane (1.5 equiv.), B_2_pin_2_ (3.0 equiv.), DMAP (3.0 equiv.), photocatalyst **PCN (1)** (10 mol%), Cs_2_CO_3_ (0.8 equiv.), MeCN (0.05 M), Kessil LEDs 390 nm (40 W) for 6 h. See Supplementary Information for full experimental details. ^*b*^B_2_Pin_2_ (10.0 equiv.), w/o DMAP.

To increase the modularity of our technique for accessing ArCF_2_−BCP frameworks, we explored the possibility of using a multi-component coupling strategy to approach synthetically versatile ArCF_2_−BCP boronates, which can be functionalized in a wide range of bond-forming events to fulfill the demands of medicinal chemistry^64–68^. Indeed, we found that, upon exposing ArCF_3_ and [1.1.1]propellane with B_2_pin_2_ as radical acceptor to 390 nm light-emitting diodes (LEDs) in the presence of **PCN** photocatalyst at ambient temperature, we could obtain the desired ArCF_2_−BCP boronate products **69** in 63% isolated yield (Fig. 5). A wide variety of trifluoromethylaromatics, including mono(trifluoromethyl) benzenes, bis(trifluoromethyl) benzenes, trifluoromethyl pyridines and bicyclic heteroaromatics bearing various functional groups can be incorporated under these reaction conditions (**69**−**74**, 50−67% yield). [3.1.1]propellane and [4.1.1]propellane systems can also be employed to generate the corresponding aryl difluoromethyl bicycloalkane boronate adducts with different geometries (**75** and **76**, 27% and 12% yield, respectively) albeit with diminished efficiency. Lastly, we found that this transformation can accommodate drug-like analogs with useful levels of efficiency (**77** and **78**, 48% and 41% yield, respectively), in which the Bpin group can serve as a universal late-stage functionalization linchpin. For example, the oxidation of BCP boronates **69** led to the medicinally interesting alcohol **79** (72% yield)^64^. The Bpin group can also be converted into organotrifluoroborate salts **80** (86% yield), a versatile building block that can be utilized in subsequent photoredox functionalization for downstream diversification, such as Minisci reaction (**81**, 31% yield)^66^, Giese addition (**82**, 34% yield)^66^, Chan-Lam coupling^66^, arylation^67^ and so on^68^.

### Preparation of pharmaceutical analogs

**Fig. 6:**
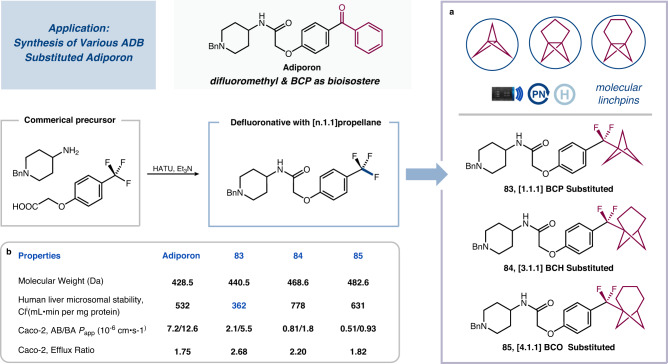
Preparation of pharmaceutical analogs with difluoromethyl and BCP as bioisosteres and pharmacological properties. **a** Successfully synthesized bioisosteres of adiporon; **b** Pharmacological Properties of Adiporon and its bioisosteres.

To demonstrate the potential of these ArCF_2_−BCP cores as bioisosteres for benzophenone-type drugs and the applicability of our procedures in drug discovery settings, we conducted defluoronative coupling reactions to the rapid synthesis of ADB-substituted analogs of Adiporon, an established adiponectin receptor agonist^69,70^. As shown in Fig. 6, a two-step sequence employing condensation and photocatalytic defluorinative coupling is applied to produce three structurally distinct bioisosteres of Adiporon (**83** [1.1.1]BCP−Adiporon, **84** [3.1.1]BCH−Adiporon and **85** [4.1.1]BCO−Adiporon). We then tested these ADB pharmaceutical analogs in comparison to their benzophenone-containing counterparts. Interestingly, [1.1.1]BCP substituted analog **83** was found to be metabolically stable, with reduced clearance rates in human liver microsomes, although its membrane permeability (Caco-2) was slightly decreased compared to its parent drug. These findings underline the potential of the ADB scaffold as a beneficial motif for enhancing the pharmacological properties of drug candidates containing a benzophenone core.

In summary, we describe herein that C−F bond functionalization can be merged with strain-release coupling for the expeditous synthesis of difluoromethyl BCP arenes/difluoromethyl BCP boronates. A diverse array of trifluoromethylaromatics can be employed to couple with [1.1.1]propellane as well as [3.1.1]heptane and [4.1.1]octane strain ring systems delivering a diversifiable ArCF_2_−BCP synthetic linchpin. We demonstrate the applicability of this transformation in synthesizing complex drug substrates, showcasing its potential role in drug discovery. Biological testing of ADB analogs of a known bioactive compound exhibited promising value to the pharmaceutical industry as a substitute for the benzophenone core.

## Methods

### General procedure for defluorinative coupling of ArCF_3_ with [n.1.1]Propellane

To a 40 mL vial equipped with a stir bar, suitable photocatalyst **PCN (1)** (38.9 mg, 0.06 mmol, 10 mol%) or **PBN (3)** (21.2 mg, 0.06 mmol, 10 mol%) were added, followed by substituted trifluoromethylarenes (0.60 mmol, 1.0 equiv.), Cs_2_CO_3_ (235 mg, 0.72 mmol, 1.2 equiv.) or CsOH·H_2_O (121 mg, 0.72 mmol, 1.2 equiv.), *γ*-terpinene (480 μL, 3.0 mmol, 5.0 equiv.), anhydrous and degassed DMSO (24.0 mL) in the glovebox and stirring for 30 seconds. A solution of [n.1.1]propellane (1.5~2.0 equiv.) was added at last, and the vial was quickly sealed with Parafilm and PVC tape. Subsequently, the solution was stirred 2 min to accelerate the dissolution of the base. The reaction was stirred and irradiated using 40 W 427 nm blue LED lamps (5 cm away, with a cooling fan & refrigeration air-conditioning at 18 °C to maintain the reaction at room temperature) for 12 hours. The reaction mixture was removed from the light, cooled to ambient temperature, and quenched by exposure to air. diluted with water and EA, and the aqueous layer was extracted with three portions of EA. The combined organic layers were washed with brine, dried over Na_2_SO_4_, filtered, and concentrated. The residue was purified by flash chromatography on silica gel to afford the desired product.

### General procedure for defluorinative three-component coupling

To a 40 mL vial equipped with a stir bar, photocatalyst **PCN (1)** (77.8 mg, 0.12 mmol, 10 mol%) was added, followed by substituted trifluoromethylarenes (1.20 mmol, 1.0 equiv.), Cs_2_CO_3_ (313 mg, 0.96 mmol, 0.8 equiv.), B_2_pin_2_ (918 mg, 3.60 mmol, 3.0 equiv.), 4-dimethylaminopyridine (439 mg, 3.60 mmol, 3.0 equiv.) anhydrous and degassed MeCN (24.0 mL) under nitrogen atmosphere (or nitrogen bubbling) and stirring for 30 s. A Solution of [1.1.1]propellane in DMF (2.5 M) (720 μL, 1.80 mmol, 1.5 equiv.) was added at last, and the vial was quickly sealed with Parafilm and PVC tape. Subsequently, the solution was allowed to be stirred for 2 min to accelerate the dissolution of the base. The reaction was stirred and irradiated using a 40 W 390 nm purple LED lamps (5 cm away, with a cooling fan & refrigeration air-conditioning at 18 °C to maintain the reaction at room temperature) for 6 h. The reaction mixture was removed from the light, cooled to ambient temperature, and quenched by exposure to air. diluted with water and EA, and the aqueous layer was extracted with three portions of EA. The combined organic layers were washed with brine, dried over Na_2_SO_4_, filtered, and concentrated. The residue was purified by flash chromatography on silica gel to afford the desired difluoromethyl BCP boronates.

### Supplementary information

Supplementary Information

Peer Review File

## Data Availability

The data supporting the findings of the study are available in the paper and its Supplementary Information. All data are available from the corresponding authors upon request.
