# Supplementary material for: C−F bond activation enables synthesis of aryl difluoromethyl bicyclopentanes as benzophenone-type bioisosteres
Source: Nat Commun. 2024 Jan 10;15:419. doi: 10.1038/s41467-023-44653-6 (PMC10781780; doi:10.1038/s41467-023-44653-6)
Supplement: Supplementary file 1 — Supplementary Information [file 41467_2023_44653_MOESM1_ESM.pdf]

Supplementary Information

C−F Bond Activation Enables Synthesis of Aryl Difluoromethyl Bicyclopetanes as Benzophenone-Type Bioisosteres

Mingshuo Chen[1], Yuang Cui[1], Xiaoping Chen[1], Rui Shang[2], and Xiaheng Zhang[1,*]

[1]*School of Chemistry and Materials Science, Hangzhou Institute for Advanced Study, University of Chinese Academy of Sciences, 1 Sub-lane Xiangshan, Hangzhou 310024, P. R. China.*

[2]*Department of Chemistry, The University of Tokyo, Tokyo, 113-0033, Japan.*

*Corresponding author. Email: xiahengz@ucas.ac.cn

Table of Contents

1) General Information **S3**

2) PN Photocatalyst Synthesis **S4**

3) PN Photocatalyst Characterization **S13**

4) Stern-Volmer Quenching Study **S17**

5) UV-Vis Absorption Spectra Analysis **S18**

6) Propellane Synthesis **S20**

7) Reaction Optimization **S26**

8) Quantum Yield Measurement **S31**

9) Reaction Scope Limitation **S34**

10) Procedure for Construction of Aryl Difluoromethyl Propellane **S35**

11) Experimental Data **S39**

12) Evaluation of ADME Properties **S93**

13) References **S95**

14) Spectral Data **S97**

1) General Information

Commercial reagents were purified prior to use following the guidelines of Perrin and Armarego.[1] All solvents were purified according to the method of Grubbs.[2] Organic solutions were concentrated under reduced pressure on a Büchi rotary evaporator using a water bath. All reactions were carried out in oven-dried tubes, prepared under nitrogen atmosphere. Commercial reagents were purchased from Bide Pharmatech CO.,Ltd, Honghu Pharmatech Ltd., Adamas-beta, J&K, TCI, Yuanye Bio-Technology. Chromatographic purification of products was accomplished using forced-flow chromatography on silica gel (Silicycle, 230–400 mesh) according to the method of Still. Thin-layer chromatography was performed on Huanghai 0.25 mm silica gel F-254 plates. Visualization of the developed chromatogram was performed by fluorescence quenching, ceric ammonium molybdate stain, Phosphomolybdic Acid stain or $KMnO_4$ stain. ^{1}H NMR spectra were recorded on a Bruker UltraShield Plus Avance III 500 MHz or a Bruker UltraShield Plus Avance III 400 MHz and are internally referenced to residual protic $CDCl_3$ (7.26 ppm), CD_2Cl_2 (5.32 ppm), DMSO-*d*6 (2.50 ppm). Data for ^{1}H NMR are reported as follows: chemical shift (δ ppm), multiplicity (s = singlet, d = doublet, t = triplet, q = quartet, m = multiplet, dd = doublet of doublets, dt = doublet of triplets, dq = doublet of quartets), coupling constant (Hz), and integration. ^{13}C NMR spectra were recorded on a Bruker UltraShield Plus Avance III 500 MHz (125 MHz) or Bruker UltraShield Plus Avance III 400 MHz (100 MHz) and data are reported in terms of chemical shift relative to $CDCl_3$ (77.16 ppm), CD_2Cl_2 (53.84 ppm), DMSO-*d*6 (39.52 ppm). ^{19}F NMR spectra were recorded on a Bruker UltraShield Plus Avance III 500 MHz (471 MHz) or Bruker UltraShield Plus Avance III 400 MHz (376 MHz). ^{31}P NMR spectra were recorded on a Bruker UltraShield Plus Avance III 500 MHz (202 MHz) or Bruker UltraShield Plus Avance III 400 MHz (162 MHz). ^{11}B NMR spectra were recorded on a Bruker UltraShield Plus Avance III 400 MHz (128 MHz). High Resolution Mass Spectra were obtained on Thermo Fisher Exactive Plus Orbitrap Mass Spectrum (ESI), Orbitrap Exploris GC Mass Spectrum (EI) or Waters Synap*t*-G2-Si Mass Spectrum (APCI). Ultraviolet-Visible absorption spectra was acquired using Agilent Cary 60. Steady-state emission spectra and Quantum yield were acquired using a Hitachi F-4700. Electrochemical measurements were carried out using a Gamry Interface 1010 electrochemical analyzer.

2) PN Photocatalyst Synthesis

2'-(Diphenylphosphaneyl)-[1,1'-biphenyl]-2-amine (3)

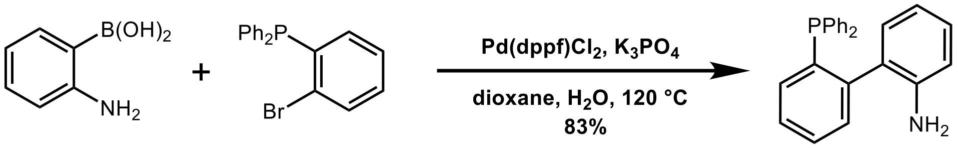

PBN (3)

According to literature[3], to a Schlenk tube equipped with stir bar was added (2-aminophenyl)boronic acid (10.2 g, 30 mmol, 1.0 equiv.), (2-bromophenyl)diphenylphosphane (8.2 g, 60 mmol, 2.0 equiv.), Pd(dppf)Cl_2 (653 mg，0.9 mmol，3 mol%), K_3PO_4 (19.1 g, 90 mmol, 3.0 equiv.), 1,4-dioxane (120 mL), water (20 mL) in glovebox and sealed. The reaction was heated to 120 °C outside the glovebox. After 12 hours the reaction was quenched by exposure to air and dilute with water and DCM and filtered to remove the inorganic precipitate. The organic layer was collected and washed by brine, dried over Na_2SO_4, filtered, concentrated *in vacuo*, giving a pale yellow oil. The ice cold methanol was added and kept at 0 °C, giving a white solid (**3**, 8.8 g, 83%)

^{1}H NMR (500 MHz, $CDCl_3$) δ 7.28 (td, *J* = 7.4, 1.4 Hz, 1H), 7.19 – 7.10 (m, 7H), 7.06 (td, *J* = 7.5, 1.9 Hz, 2H), 7.02 – 6.95 (m, 2H), 6.59 – 6.53 (m, 2H), 6.46 (t, *J* = 7.4 Hz, 1H), 3.27 (s, 2H).

^{13}C NMR (125 MHz, $CDCl_3$) δ 144.74 (d, *J* = 30.7 Hz), 143.58 (d, *J* = 1.0 Hz), 138.16 (d, *J* = 12.4 Hz), 137.52 (d, *J* = 12.2 Hz), 137.09 (d, *J* = 11.4 Hz), 134.17 – 133.87 (m), 133.94, 133.84, 131.12 (d, *J* = 3.2 Hz), 130.67 (d, *J* = 5.1 Hz), 129.39, 128.81, 128.56 (d, *J* = 1.7 Hz), 128.43 (d, *J* = 6.7 Hz), 128.33 (d, *J* = 7.1 Hz), 127.95, 127.15 (d, *J* = 6.9 Hz), 117.87, 115.36.

^{31}P NMR (202 MHz, $CDCl_3$) δ -12.97 (s, 1P).

HRMS (ESI) m/z calculated for $C_{24}H_{21}NP^+$ ($[M+H]^+$): 354.1406, found 354.1398.

***N*-(2'-(Diphenylphosphaneyl)-[1,1'-biphenyl]-2-yl)acetamide (c)**

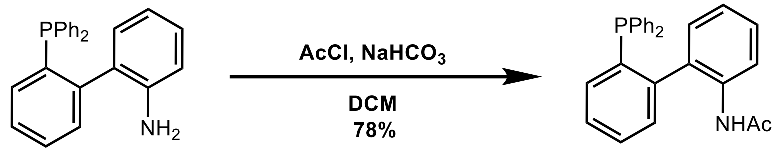

PBN (3) **PBN (c)**

To a round flask equipped with stir bar was added **PBN (3)** (1.06 g, 3. mmol, 1.0 equiv.), $NaHCO_3$ (1.51 g, 18.0 mmol, 6.0 equiv.), DCM (20 mL) in glovebox. The mixture was stirred for 10 mins, the acetyl chloride (0.42 mL, 6.0 mmol, 2.0 equiv.) was added dropwise. After 4 hours, the reaction was quenched by water and diluted with DCM, the organic layer was combined, washed by brine, dried over Na_2SO_4, filtered, concentrated *in vacuo*, giving a pale yellow oil. The residue was purified by flash column chromatography on silica gel (PE:EA = 4:1) to afford a yellow solid (**c**, 924 mg, 78%).

^{1}H NMR (500 MHz, CD_2Cl_2) δ 8.14 (d, J = 8.3 Hz, 1H), 7.48 (td, J = 7.5, 1.4 Hz, 1H), 7.43 – 7.14 (m, 15H), 7.06 – 6.96 (m, 2H), 6.64 (s, 1H), 1.79 (s, 3H).

^{13}C NMR (125 MHz, CD_2Cl_2) δ 168.05, 143.62 (d, J = 29.9 Hz), 138.60 (d, J = 13.2 Hz), 137.14 (d, J = 11.8 Hz), 136.94 (d, J = 12.1 Hz), 135.91, 134.58 (d, J = 21.1 Hz), 134.19, 133.88 (d, J = 19.7 Hz), 132.25 (d, J = 6.5 Hz), 131.27, 130.99 (d, J = 4.9 Hz), 129.76, 129.46, 129.05 (d, J = 7.3 Hz), 128.90 (d, J = 13.9 Hz), 128.79 (d, J = 6.4 Hz), 128.73, 123.76, 121.50, 24.58.

^{31}P NMR (202 MHz, CD_2Cl_2) δ -13.83 (s, 1P).

HRMS (ESI) m/z calculated for $C_{26}H_{23}NOP^+$ ($[M+H]^+$): 396.1512, found 396.1503.

2'-(Diphenylphosphaneyl)-*N*-methyl-[1,1'-biphenyl]-2-amine (d)

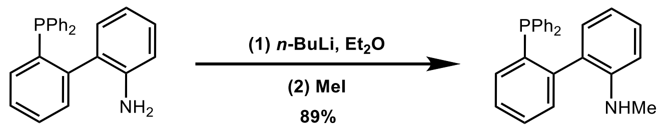

PBN (3) **PBN (d)**

To an oven-dried round bottom flask equipped with a stir bar was added **PBN (3)** (707 mg, 2 mmol, 1.0 equiv.), anhydrous THF (10 mL) in glovebox and sealed. The flask was

removed from glovebox and cold to -78 °C and *n*-BuLi (0.8 mL,2.0 mmol, 2.0 equiv., 2.5 M in hexane) was added dropwise. After 15 mins, methyl iodide (125 μL, 2.0 mmol, 1.0 equiv.) was added dropwise and moved back to room temperature. After 3 hours, the reaction was quenched and exacted by water and EA. The organic layer was combined, washed with brine, dried over Na_2SO_4, filtered, concentrated *in vacuo*. The yellow residue was purified by flash column chromatography on silica gel (PE:EA = 4:1) to afford a yellow solid (**d**, 420 mg, 57%).

^{1}H NMR (400 MHz, CD_2Cl_2) δ 7.43 (td, *J* = 7.5, 1.4 Hz, 1H), 7.34 – 7.28 (m, 7H), 7.28 – 7.12 (m, 7H), 6.76 (dd, *J* = 7.9, 1.7 Hz, 1H), 6.57 (d, *J* = 7.7 Hz, 2H), 3.28 (s, 1H), 2.57 (s, 3H).

^{13}C NMR (100 MHz, CD_2Cl_2) δ 146.95, 145.27 (d, *J* = 31.5 Hz), 138.53 (d, *J* = 12.8 Hz), 138.09 (d, *J* = 3.7 Hz), 137.96 (d, *J* = 4.0 Hz), 134.74, 134.22 (d, *J* = 20.6 Hz), 133.94 (d, *J* = 19.8 Hz), 131.35 (d, *J* = 5.2 Hz), 130.82 (d, *J* = 3.0 Hz), 129.84, 129.22, 128.88 (d, *J* = 10.6 Hz), 128.73, 128.71 (d, *J* = 10.6 Hz), 128.24, 127.31 (d, *J* = 6.8 Hz), 116.21, 109.72, 30.62.

^{31}P NMR (162 MHz, CD_2Cl_2) δ -14.11 (s, 1P).

HRMS (ESI) m/z calculated for $C_{25}H_{23}NP^+$ ($[M+H]^+$): 368.1563, found 368.1552.

2'-(Diphenylphosphaneyl)-*N*-phenyl-[1,1'-biphenyl]-2-amine (4)

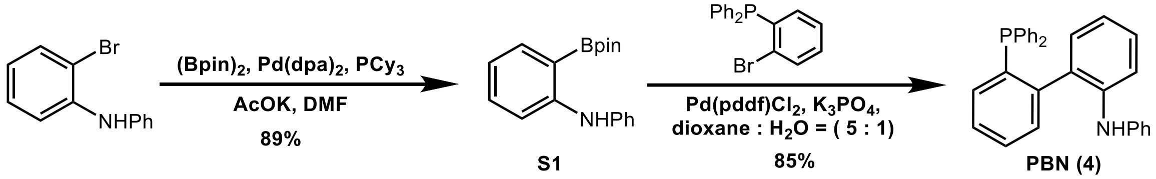

According to literature[4], to a Schlenk tube equipped with stir bar was added 2-bromo-*N*-phenylaniline (5.0 g, 17.6 mmol, 1.0 equiv.), B_2pin_2 (15.0 g, 35.2 mmol, 2.0 equiv.), Pd(dppf)Cl_2 (1.18 g, 0.88 mmol, 5 mol%), KOAc (8.5 g, 52.8 mmol, 3.0 equiv.), DMF (80 mL) in glovebox and sealed. The reaction was heated to 130 °C outside the glovebox. After 12 hours the reaction was quenched by exposure to air and dilute with water and DCM,

filtered to remove the inorganic precipitate. The organic layer was collected and washed by brine, dried over Na_2SO_4, filtered, concentrated *in vacuo*, giving a pale yellow oil. The yellow residue purified by flash column chromatography on silica gel (PE:EA = 3:1) to afford a yellow solid (**S1**, 4.62 g, 89%).

To a Schlenk tube equipped with stir bar was added (**S1**, 2.36 g, 8.0 mmol, 1.0 equiv.), and (2-bromophenyl)diphenylphosphane (3.27 g, 9.6 mmol, 1.2 equiv.), Pd(dppf)Cl_2 (290 mg，0.4 mmol，5 mol%), K_3PO_4 (5.1 g, 24 mmol, 3.0 equiv.), 1,4-dioxane (30 mL), water (6 mL) in glovebox and sealed. The reaction was heated to 130 ℃ outside the glovebox. After 12 hours the reaction was quenched by exposure to air and dilute with water and DCM and filtered to remove the inorganic precipitate. The organic layer was collected and washed by brine, dried over Na_2SO_4, filtered, concentrated *in vacuo*, giving a pale yellow oil. The yellow residue was purified by flash column chromatography on silica gel (PE:EA = 10:1) to afford yellow solid (**4**, 2.93 g, 85%).

^{1}H NMR (400 MHz, CD_2Cl_2) δ 7.46 (t, *J* = 7.5 Hz, 1H), 7.38 – 7.26 (m, 9H), 7.26 – 7.13 (m, 8H), 6.98 – 6.87 (m, 4H), 6.81 (t, *J* = 7.4 Hz, 1H), 5.27 (s, 1H).

^{13}C NMR (100 MHz, CD_2Cl_2) δ 145.05 (d, *J* = 31.3 Hz), 143.45, 141.29, 138.37 (d, *J* = 13.0 Hz), 137.74 (d, *J* = 5.6 Hz), 137.62 (d, *J* = 6.2 Hz), 134.61, 134.26 (d, *J* = 20.4 Hz), 133.95 (d, *J* = 19.8 Hz), 131.89 (d, *J* = 3.2 Hz), 131.45 (d, *J* = 6.5 Hz), 131.08 (d, *J* = 5.3 Hz), 129.85, 129.46, 128.97 (d, *J* = 12.3 Hz), 128.84, 128.73 (d, *J* = 11.1 Hz), 128.73 (d, *J* = 6.7 Hz), 128.45, 121.40, 120.36, 118.96, 116.80.

^{31}P NMR (162 MHz, CD_2Cl_2) δ -14.04 (s, 1P).

HRMS (ESI) m/z calculated for $C_{30}H_{25}NP^+$ ($[M+H]^+$): 430.1719, found 430.1706.

1-(Diphenylphosphaneyl)-9*H*-carbazole (b)

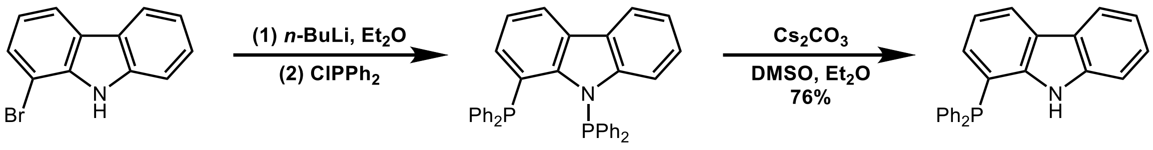

S2 **PCN (b)**

According to literature[5] with modified attempts, to an oven-dried 500 mL round bottom flask equipped with a stir bar was added 1-bromo-9*H*-carbazole (9.84 g, 40.0 mmol, 1.0 equiv.) and the oxygen was replaced by nitrogen with Schlenk line techniques. Anhydrous Et_2O (250 mL) was added in room temperature and stirred until completely dissolved. Then the flask was cold to -78 °C and *n*-BuLi (26.8 mL, 2.3 equiv., 2.5 M in hexane) was added dropwise, giving immediately a white suspension. The cold bath was removed and continued stirring at room temperature for 30 mins, giving a pale yellow solution, then returned to the cold bath at -78 °C. Chlorodiphenylphosphine (21.5 mL, 120 mmol, 3.0 equiv.) was added into this solution, dropwise, drip acceleration rate less than 1mL per 10 mins per syringe. After 6 hours, the reaction was quenched by aqueous solutions of NaH_2PO_4 (0.1 M, 2 × 100 mL) and filtered to removed inorganic salts and the aqueous layer was extracted with two portions of Et_2O. The combined organic layers were washed with brine, dried over Na_2SO_4, filtered, and concentrated *in vacuo* giving yellow oil in flask. A stir bar and Et_2O (5 mL, avoiding oiling-out) were added, and cold to 0 °C. The methanol (about 20 mL) was added dropwise giving a white precipitate from a pale yellow solution. The turbid liquid was reduced *in vacuo* at 0 °C to remove the Et_2O, and the turbid liquid was quickly separated by filtration, washed with ice cold methanol and dried under vacuum, giving a white solid (**S2**).

Then white solid **S2** was dissolved by DMSO (150 mL) and water (10 mL), a stir bar and Cs_2CO_3 (25 g) was added subsequently to hydrolyze **S2**. After 15 min, the solution was diluted with water, extracted by three portions of Et_2O, the combined organic layers were washed with brine, dried over Na_2SO_4, filtered, and concentrated *in vacuo* giving colorless oil in flask. A stir bar and Et_2O (5 mL, avoiding oiling-out) were added, and cold to 0 °C. The methanol (about 20 mL) was added dropwise giving a white precipitate from the colorless solution. The turbid liquid was reduced *in vacuo* at 0 °C to remove the Et_2O, and the turbid liquid was quickly separated by filtration, washed with ice cold methanol and dried under vacuum, giving a white solid (**b**, 10.64g, 76%).

^{1}H NMR (400 MHz, CD_2Cl_2) δ 8.37 (s, 1H), 8.13 (d, *J* = 7.7 Hz, 1H), 8.09 (d, *J* = 7.8 Hz, 1H), 7.45 – 7.32 (m, 12H), 7.23 (td, *J* = 7.4, 4.1 Hz, 2H), 7.15 (t, *J* = 7.1 Hz, 1H).

^{13}C NMR (100 MHz, CD_2Cl_2) δ 142.88 (d, *J* = 17.2 Hz), 139.67, 135.91 (d, *J* = 8.9 Hz), 133.89 (d, *J* = 19.2 Hz), 131.58 (d, *J* = 8.4 Hz), 129.40, 129.12 (d, *J* = 7.0 Hz), 126.42, 123.31, 123.27, 121.80, 120.67, 120.16 (d, *J* = 4.0 Hz), 120.01, 117.73 (d, *J* = 11.9 Hz), 111.19.

^{31}P NMR (162 MHz, CD_2Cl_2) δ -19.01 (d, *J* = 10.1 Hz, 1P).

HRMS (ESI) m/z calculated for $C_{24}H_{19}NP^+$ ($[M+H]^+$): 352.1250, found 352.1238.

3,6-Di-*tert*-butyl-1-(diphenylphosphaneyl)-9*H*-carbazole (2)

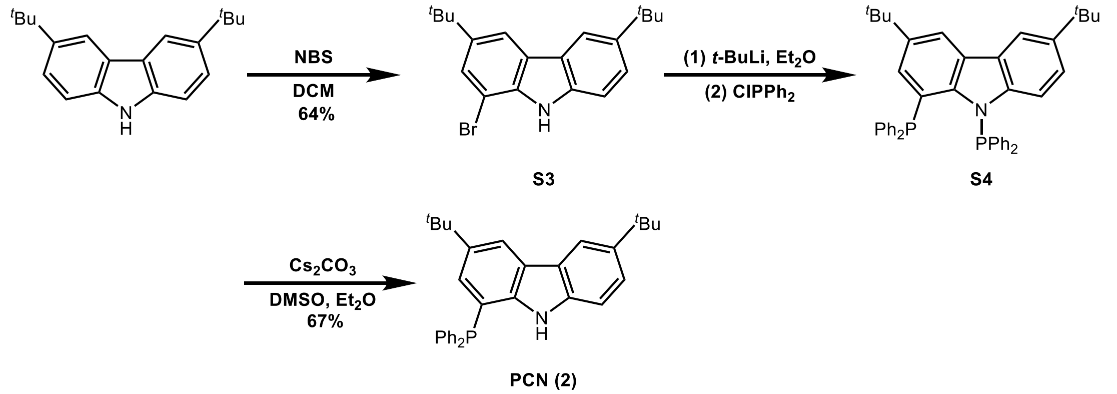

According to literature[5] with modified attempts, to an oven-dried 1 L flask equipped with a stir bar was added 3,6-di-*tert*-butyl-9*H*-carbazole (15.78 g, 56.5 mmol, 1.0 equiv.), silica gel (30 g) and DCM (750 mL) at 0 °C. The *N*-Bromosuccinimide (10.77 g, 59.3 mmol) was gradually added to the solution. After 6 hours, the reaction mixture was filtered, the precipitate was washed with DCM. The combined organic layer was washed with brine, dried over Na_2SO_4, filtered, and concentrated *in vacuo* giving pale grey oil. The residue was purified by flash column chromatography on silica gel (PE:Et_2O = 30:1) to afford colorless mesh-like solid (**S3**, 13.0 g, 64%).

To an oven-dried 300 mL round bottom flask equipped with a stir bar was added **S3** (3.58 g, 10.0 mmol, 1.0 equiv.) and the oxygen was replaced by nitrogen with Schlenk line

techniques. Anhydrous Et_2O (100 mL) was added at room temperature and stirred until completely dissolved. Then the flask was cold to -78 ℃ and *t*-BuLi (23.0 mL, 3.0 equiv., 1.3 M in pentane) was added dropwise, giving immediately a white suspension. The cold bath was removed and stirring was continued at room temperature for 30 mins, giving a pale yellow solution, then returned to the cold bath at -78 ℃. Chlorodiphenylphosphine (5.4 mL, 30.0 mmol, 3.0 equiv.) was added into this solution, dropwise, drip acceleration rate less than 1mL per 10 mins per syringe. After 6 hours, the reaction was quenched by aqueous solutions of NaH_2PO_4 (0.1 M, 2 × 50 mL) and filtered to removed inorganic salts and the aqueous layer was extracted with two portions of Et_2O. The combined organic layers were washed with brine, dried over Na_2SO_4, filtered, and concentrated *in vacuo* giving yellow oil in flask. A stir bar and Et_2O (3 mL, avoiding oiling-out) were added, and cold to 0 ℃. The methanol (about 10 mL) was added dropwise giving a white precipitate from a pale yellow solution. The turbid liquid was reduced *in vacuo* at 0 ℃ to remove the Et_2O, and the turbid liquid was quickly separated by filtration, washed with ice cold methanol and dried under vacuum, giving a white solid (**S4**).

The white solid **S4** was dissolved by DMSO (50 mL) and water (5 mL), a stir bar and Cs_2CO_3 (10 g) was added subsequently to hydrolyze **S4**. After 15 mins, the solution was diluted with water, extracted by three portions of Et_2O, the combined organic layers were washed with brine, dried over Na_2SO_4, filtered, and concentrated *in vacuo* giving colorless oil in flask. A stir bar and Et_2O (3 mL, avoiding oiling-out) were added, and cold to 0 ℃. The methanol (about 10 mL) was added dropwise giving a white precipitate from the colorless solution. The turbid liquid was reduced *in vacuo* at 0 ℃ to remove the Et_2O, and the turbid liquid was quickly separated by filtration, washed with ice cold methanol and dried under vacuum, giving a white solid (**2**, 3.12 g, 67%).

^{1}H NMR (400 MHz, CD_2Cl_2) δ 8.15 (s, 1H), 8.09 (s, 1H), 7.97 (s, 1H), 7.43 (d, *J* = 8.5 Hz, 1H), 7.36 (s, 8H), 7.27 (d, *J* = 8.7 Hz, 1H), 7.20 (d, *J* = 8.5 Hz, 1H), 1.42 (s, 10H), 1.34 (s, 4H).

^{13}C NMR (100 MHz, CD_2Cl_2) δ 142.87, 142.82 (d, *J* = 5.3 Hz), 141.27 (d, *J* = 14.2 Hz), 138.11, 136.29 (d, *J* = 9.8 Hz), 133.70 (d, *J* = 19.1 Hz), 129.99 (d, *J* = 14.6 Hz), 129.25,

129.06 (d, J = 6.8 Hz), 124.12, 123.39 (d, J = 3.6 Hz), 123.18 (d, J = 1.8 Hz), 118.14, 116.59, 116.50, 110.53, 35.00 (d, J = 8.0 Hz), 32.04 (d, J = 12.4 Hz).

^{31}P NMR (162 MHz, CD_2Cl_2) δ -16.07 (s, 1P).

HRMS (ESI) m/z calculated for $C_{32}H_{35}NP^+$ ($[M+H]^+$): 464.2502, found 464.2490.

3,6-Di-*tert*-butyl-1,8-bis(diphenylphosphaneyl)-9*H*-carbazole (1)

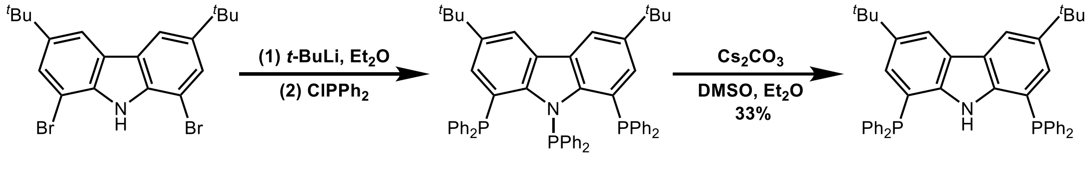

According to literature[5] with modified attempts, to an oven-dried 300 mL round bottom flask equipped with a stir bar was added 1,8-dibromo-3,6-di-*tert*-butyl-9*H*-carbazole (17.48 g, 40 mmol, 1.0 equiv.) and the oxygen was replaced by nitrogen with Schlenk line techniques. Anhydrous Et_2O (200 mL) was added in room temperature and stirred until completely dissolved. Then the flask was cold to -78 °C and *t*-BuLi (172 mL, 6.0 equiv., 1.3 M in pentane) was added dropwise, giving immediately a white suspension. The cold bath was removed and stirring was continued at room temperature for 30 mins, giving a pale yellow solution, then returned to the cold bath at -78 °C. Chlorodiphenylphosphine (36 mL, 200 mmol, 5.0 equiv.) was added into this solution, dropwise, drip acceleration rate less than 1mL per 10 mins per syringe. After 6 hours, the reaction was quenched by aqueous solutions of NaH_2PO_4 (0.1 M, 2 × 100 mL) and filtered to removed inorganic salts and the aqueous layer was extracted with two portions of Et_2O. The combined organic layers were washed with brine, dried over Na_2SO_4, filtered, and concentrated *in vacuo* giving yellow oil in flask. A stir bar and Et_2O (5 mL, avoiding oiling-out) were added, and cold to 0 °C. The methanol (about 20 mL) was added dropwise giving a white precipitate from the pale yellow solution. The turbid liquid was reduced *in vacuo* at 0 °C to remove the Et_2O, and the turbid liquid was quickly separated by filtration, washed with ice cold methanol and dried under vacuum, giving a white solid (**S5**).

The above white solid was dissolved by DMSO (200mL) and water (10 mL), a stir bar and Cs_2CO_3 (32 g) was added subsequently to hydrolyze **S5**. After 15 min, the solution was diluted with water, extracted by three portions of Et_2O, the combined organic layers were washed with brine, dried over Na_2SO_4, filtered, and concentrated *in vacuo* giving colorless oil in flask. A stir bar and Et_2O (5 mL, avoiding oiling-out) were added, and cold to 0 °C. The methanol (about 20 mL) was added dropwise giving a white precipitate from the colorless solution. The turbid liquid was reduced *in vacuo* at 0 °C to remove the Et_2O, and the turbid liquid was quickly separated by filtration, washed with ice cold methanol and dried under vacuum, giving a white solid (**1**, 8.63 g, 33%).

^{1}H NMR (500 MHz, CD_2Cl_2) δ 8.15 (d, *J* = 1.9 Hz, 2H), 7.85 (s, 1H), 7.34 – 7.20 (m, 22H), 1.34 (s, 18H).

^{13}C NMR (125 MHz, CD_2Cl_2) δ 143.13 (d, *J* = 6.1 Hz), 140.90 (d, *J* = 12.0 Hz), 136.22 (d, *J* = 10.3 Hz), 133.52 (d, *J* = 19.0 Hz), 130.49 (d, *J* = 18.2 Hz), 129.20, 129.06 (d, *J* = 6.8 Hz), 123.18 (d, *J* = 5.0 Hz), 118.19, 117.04 (d, *J* = 12.3 Hz), 35.07, 32.00.

^{31}P NMR (202 MHz, CD_2Cl_2) δ -14.90 (q, *J* = 8.3 Hz, 1P).

HRMS (ESI) m/z calculated for $C_{44}H_{44}NP_2^+$ ($[M+H]^+$): 648.2943, found 648.2927.

3) PN Photocatalyst Characterization

UV-Visible Absorption Data

In a nitrogen glove box, a 10 μM DMSO solution of photocatalyst and 20 mg Cs_2CO_3 was added in the cuvette, then sealed with parafilm.

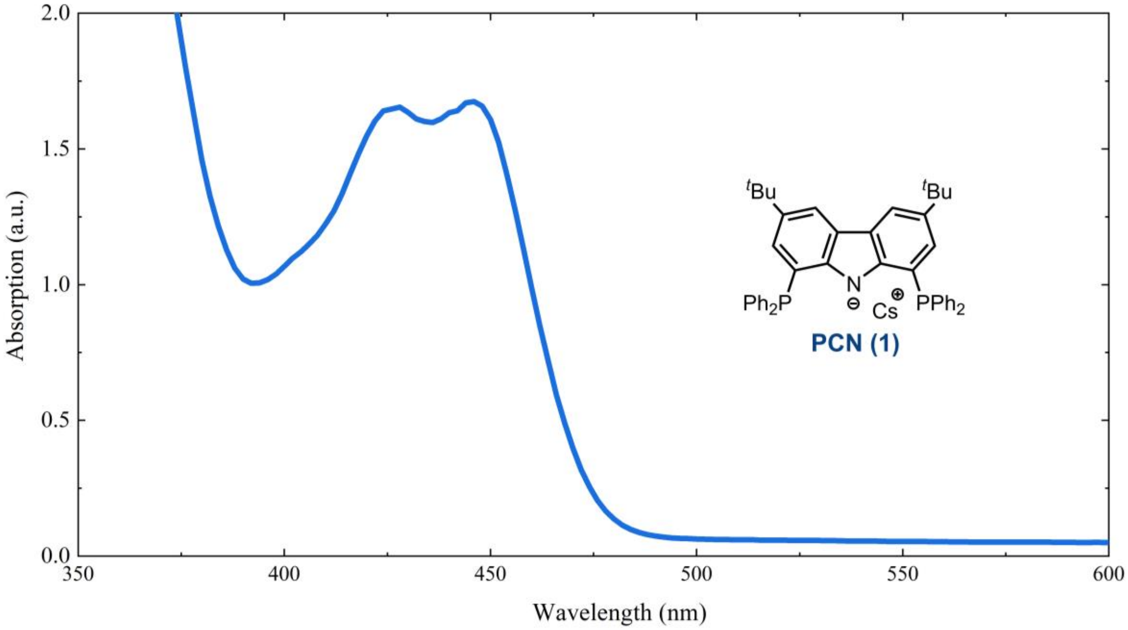

Supplementary Figure 1. UV-Visible Spectrum of **PCN (1)** anion, the maxima was obtained at 428 nm and 446 nm

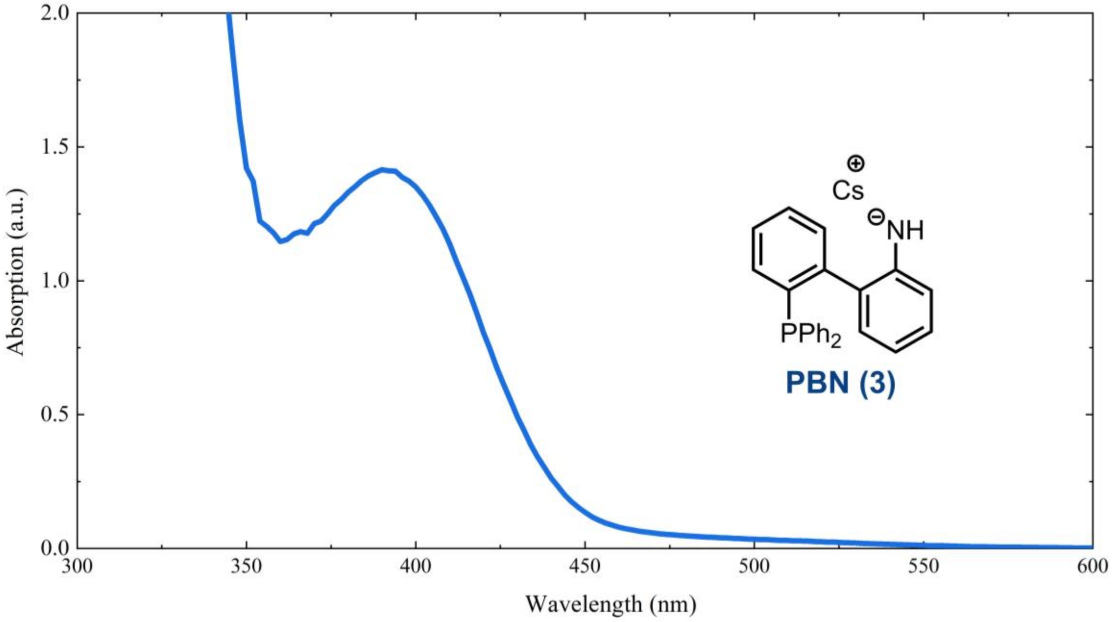

Supplementary Figure 2. UV-Visible Spectrum of **PBN (3)** anion, the maxima was obtained at 390 nm

Emission Data

Using same batch of photocatalyst sample in the UV-vis Absorption, irradiated at 390 nm.

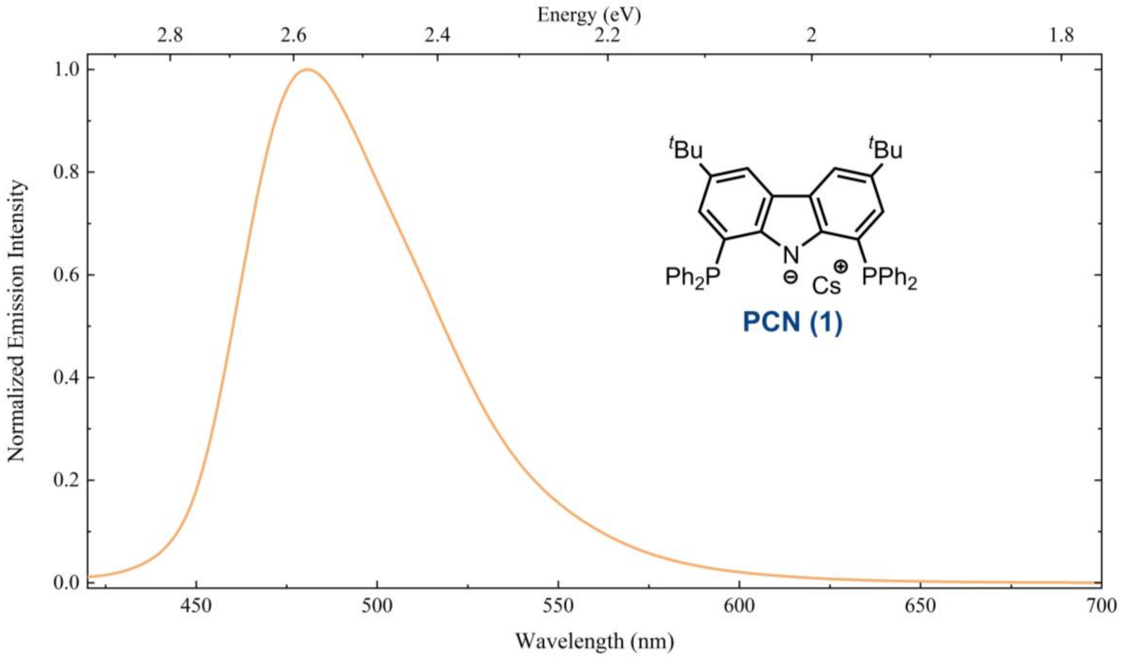

Supplementary Figure 3. Emission spectra of **PCN (1)** anion in DMSO. The maxima was obtained at 482 nm.

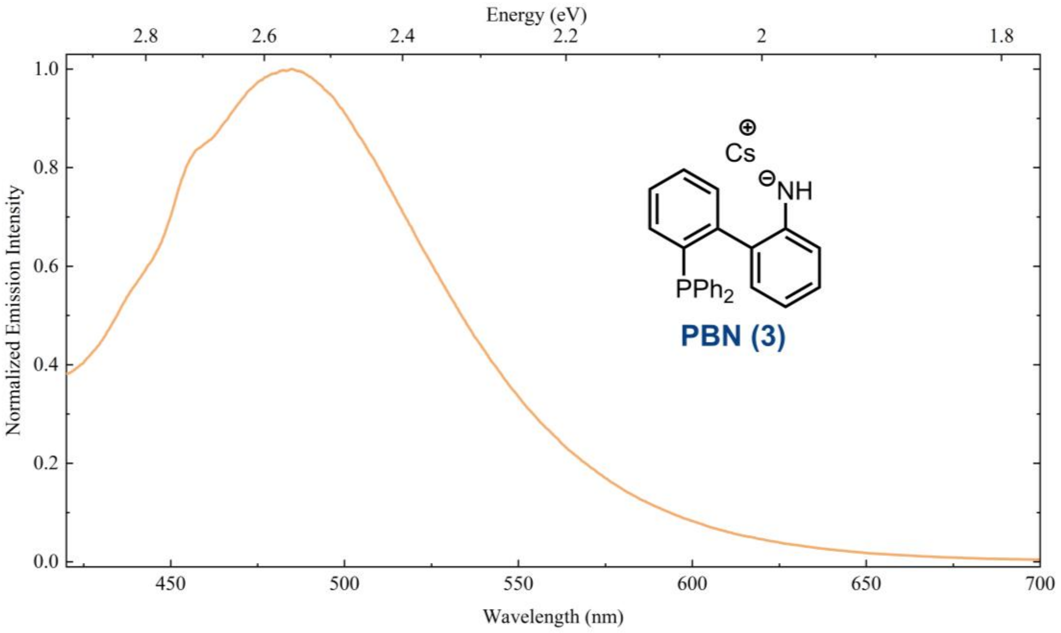

Supplementary Figure 4. Emission spectra of **PBN (3)** anion in DMSO. The maxima was obtained at 472 nm.

Cyclic Voltammogram

The oxidation/reduction potential was measured using platinum disc working electrode, a platinum wire counter electrode

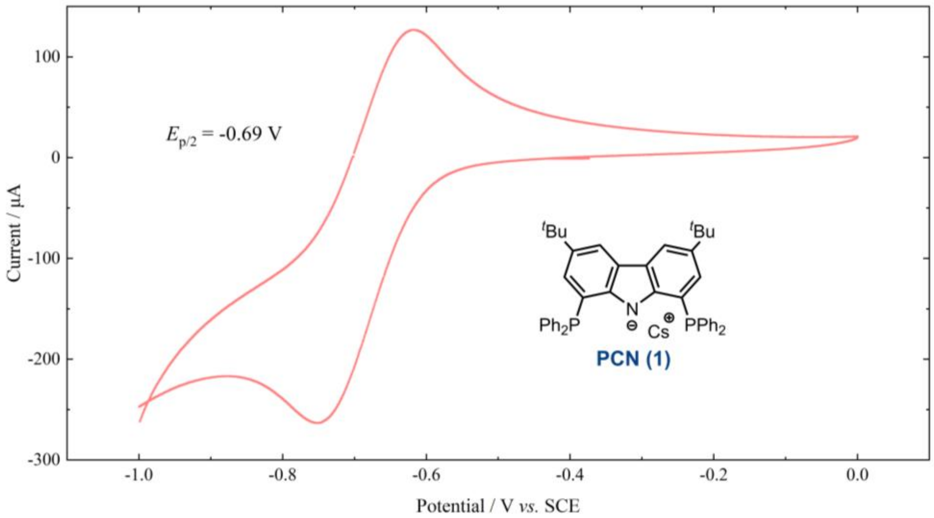

Supplementary Figure 5. Cyclic voltammogram of 20 mM **PCN (1)** anion in DMSO, using 250 mM NBu_4PF_6 as supporting electrolyte, platinum disc working electrode, a platinum wire counter electrode, scan rate 100 mV/s

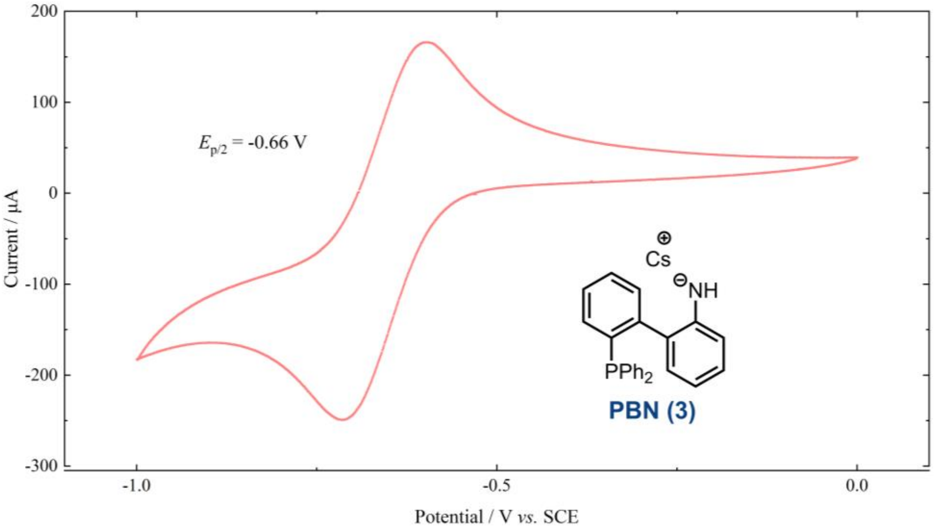

Supplementary Figure 6. Cyclic voltammogram of 20 mM **PBN (3)** anion in DMSO, using 250 mM NBu_4PF_6 as supporting electrolyte, platinum disc working electrode, a platinum wire counter electrode, scan rate 100 mV/s

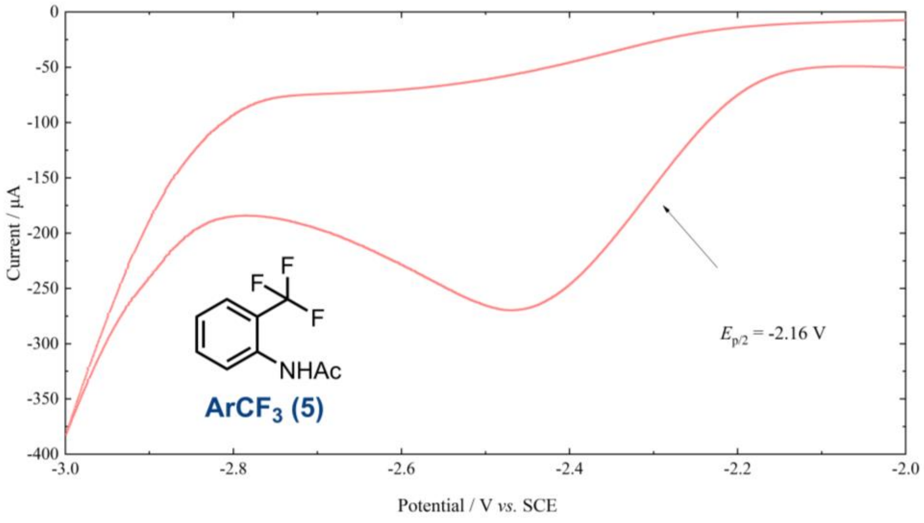

Supplementary Figure 7. Cyclic voltammogram of 20 mM **$ArCF_3$ (5)** in MeCN, using 0.25 M NBu_4PF_6 as supporting electrolyte, glass carbon working electrode, platinum disc counter electrode, scan rate 100mV/s

Determination of Triplet Excited State Potentials

According to Supplementary Figure 3 & Supplementary Figure 4, Triplet Excited State Energy of **PCN (1)** & **PBN (3)** anion employing the follow equation:

$$E^{T}_{0\text{-}0}(\mathrm{PCN1^{-}} / \mathrm{PCN1^{-*}}) = 1240/482 = 2.57 \text{ eV}$$

$$E^{T}_{0\text{-}0}(\mathrm{PBN3^{-}} / \mathrm{PCN3^{-*}}) = 1240/472 = 2.62 \text{ eV}$$

According to Supplementary Figure 5 & Supplementary Figure 6, Ground State Redox Potential of **PCN (1)** & **PBN (3)** anion as follow:

$$E_{\mathrm{p/2}}(\mathrm{PCN1^{\cdot}/PCN1^{-}}) = -0.69 \text{ eV}$$

$$E_{\mathrm{p/2}}(\mathrm{PBN3^{\cdot}/PBN3^{-}}) = -0.66 \text{ eV}$$

With data in hand, we calculated the Triplet Excited State Redox Potential **PCN (1)** & **PBN (3)** as follow:

$$E^{T}_{\mathrm{p/2}}(\mathrm{PCN1^{\cdot}/PCN1^{-*}}) = E_{\mathrm{p/2}}(\mathrm{PCN1^{\cdot}/PCN1^{-}}) - E^{T}_{0\text{-}0}(\mathrm{PCN1^{-}/PCN1^{-*}})$$

$$= -0.69 - 2.57 = -3.26 \text{ V}$$

$$E^{T}_{\mathrm{p/2}}(\mathrm{PBN3^{\cdot}/PBN3^{-*}}) = E_{\mathrm{p/2}}(\mathrm{PBN3^{\cdot}/PBN3^{-}}) - E^{T}_{0\text{-}0}(\mathrm{PBN3^{-}/PBN3^{-*}})$$

$$= -0.66 - 2.62 = -3.28 \text{ V}$$

4) Stern-Volmer Quenching Study

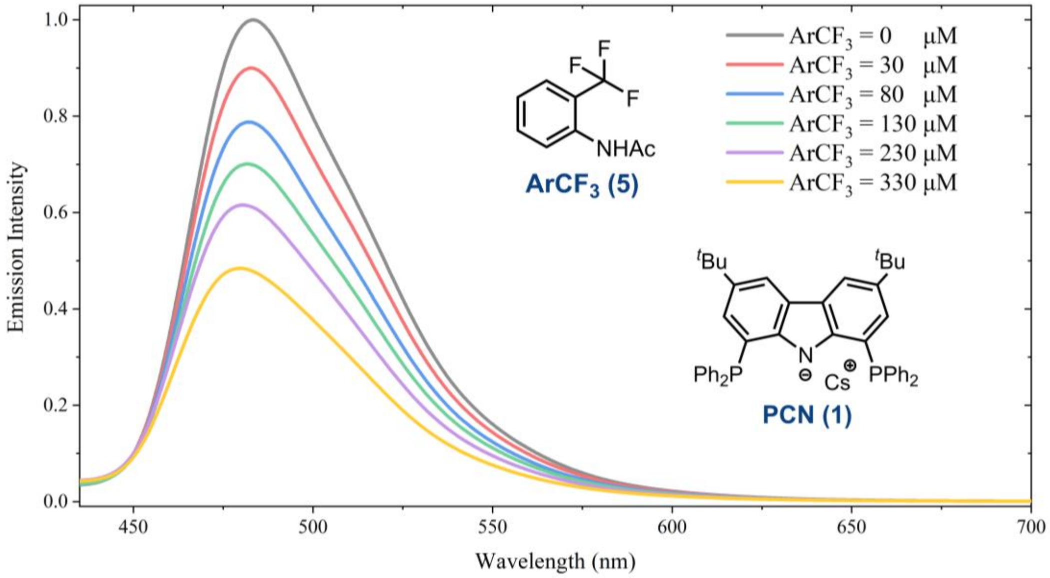

Supplementary Figure 8. Quenching of the **PCN (1)** anion emission in the presence of increasing amounts of **ArCF$_3$ (5)**

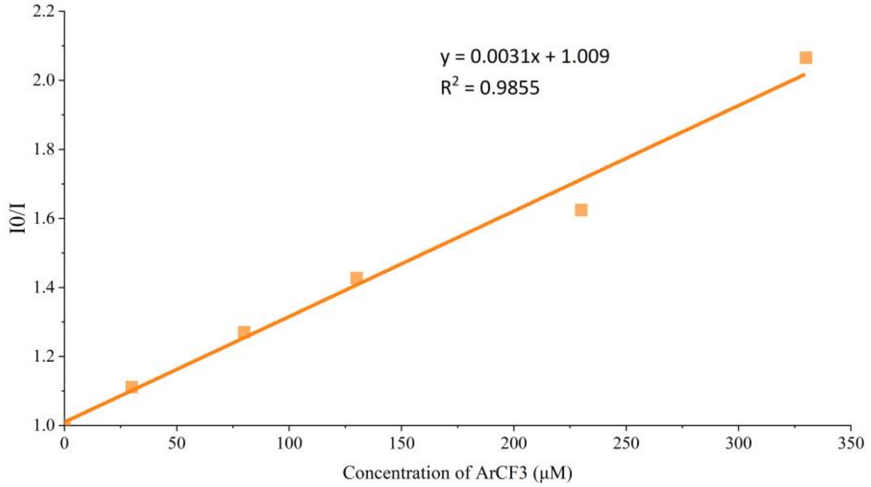

Supplementary Figure 9. Stern-Volmer quenching plot of **ArCF$_3$ (5)**.

5) UV-Vis Absorption Spectra Analysis

In a nitrogen glove box, a 10 μM DMSO solution of photocatalyst or ArCF substrate and 20 mg of base were added to the cuvette, which was then sealed with parafilm. For the convenience of explanation and to avoid overlap, samples "PCN + $ArCF_3$", "PBN + $ArCF_3$", and "PBN + Cs_2CO_3" were slightly diluted.

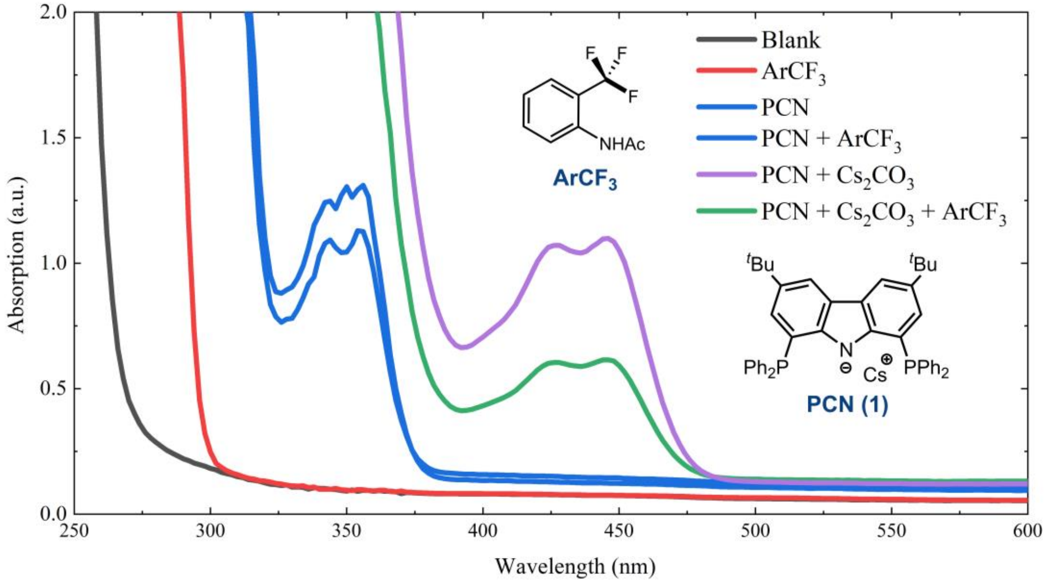

Supplementary Figure 10. UV-Visible Spectrum of **PCN (1)** with additive.

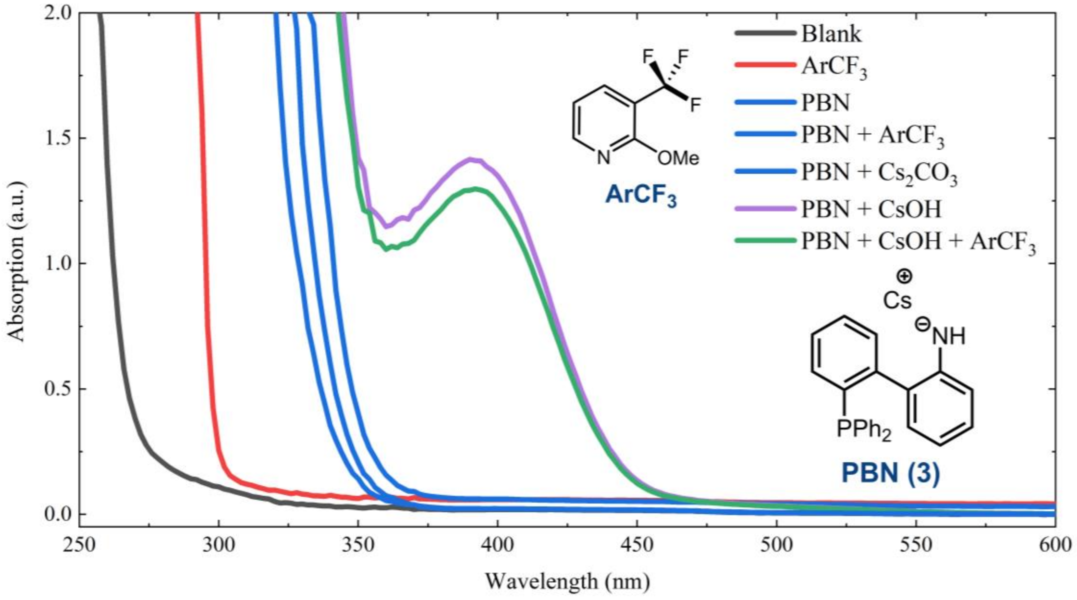

Supplementary Figure 11. UV-Visible Spectrum of **PBN (3)** with additive.

As shown in Supplementary Figure 10, when the photocatalyst **PCN (1)** was mixed with Cs_2CO_3, the absorption spectra of **PCN (1)** exhibited a redshift and generated two absorption peaks at 427 nm and 446 nm. Individually mixing **PCN (1)** and $ArCF_3$ or adding $ArCF_3$ to the sample "PCN + Cs_2CO_3" did not result in a redshift of the photocatalyst absorption spectra.

As shown in Supplementary Figure 11, when the photocatalyst **PBN (3)** was mixed with CsOH, the absorption spectra of **PBN (3)** exhibited a redshift and generated an absorption peak at 390 nm, indicating that Cs_2CO_3 is not strong enough to ionize **PBN (3)**. Individually mixing **PBN (3)** and $ArCF_3$ or adding $ArCF_3$ to the sample "PBN + CsOH" did not result in a redshift of the photocatalyst absorption spectra.

Based on the previous discussion, we found that the photocatalyst could be ionized by a suitable base and the absorption spectra would redshift into the purple to blue range. With this redshift, the photocatalyst could be excited by photons and further initiate the photocatalytic cycle by proceeding with the single electron reduction of trifluoromethylarenes, as shown in Fig. 2.

6) Propellane Synthesis

[1.1.1]propellane, **[3.1.1]propellane**, **[4.1.1]propellane** were known compounds. **[3.1.1]propellane** was prepared according to the literature procedure[6] without modification. **[1.1.1]propellane**[7] and **[4.1.1]propellane**[8] were prepared according to the literature procedures with modification which was shown as below.

Preparation of [1.1.1]propellane

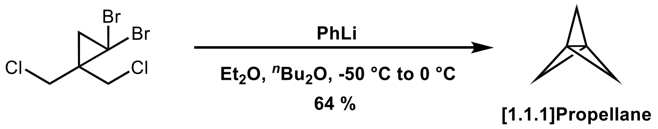

According to literature[7] with modified attempts, to an oven-dried round flask equipped with stir bar was added 1,1-dibromo-2,2-bis(chloromethyl)cyclopropane (50 g, 168.5 mmol, 1.0 equiv.) and the oxygen was replaced by nitrogen with Schlenk line techniques. Anhydrous *n*-Bu_2O (350 mL), was added at -78 °C, then PhLi (280 mL, 532 mmol, 3.15 equiv., 1.9 M in *n*-Bu_2O) was slowly added. After 15 mins, the flask was moved to 0 °C and stirred. After 2 hours, vacuum distillation was applied to the flask with receiving flask containing DMF (17 mL), condensation temperature at -78 °C with terminate pressure at 35 mbar at 30 °C to afford a solution of [1.1.1]propellane in Et_2O (from PhLi), *n*-Bu_2O and DMF (89 mL), which was stored under an inert atmosphere at -80 °C. The yield was determined by ^1H NMR spectroscopy using mesitylene as an internal standard. The concentration of the [1.1.1]propellane solution was 1.2 M, with yield of 64%. the highest concentration of [1.1.1]propellane solution we accessed was 2.5 M.

NOTE: We highly recommend storing the propellane solution at -80 °C when the concentration is higher than 0.7 M.

Determination of the Concentration of [1.1.1]propellane

In an 5 mm NMR tube was added 50 μL (0.359 mmol) mesitylene and 100 μL the [1.1.1]propellane solution and filled with appropriate amount of $CDCl_3$. Integration of 6 [1.1.1]propellane protons (at ~2.0) are set to 6, and the 3 aromatic protons on mesitylene are integrated at ~6.8, the concentration of propellane could be calculated as follow equation:

$$C(propellane) = \frac{n}{v} = \frac{\frac{0.359}{Int(mesitylene)/3}}{0.1} = \frac{10.8}{Int(mesitylene)} = \frac{10.8}{8.97} = 1.20\ \mathrm{M}$$

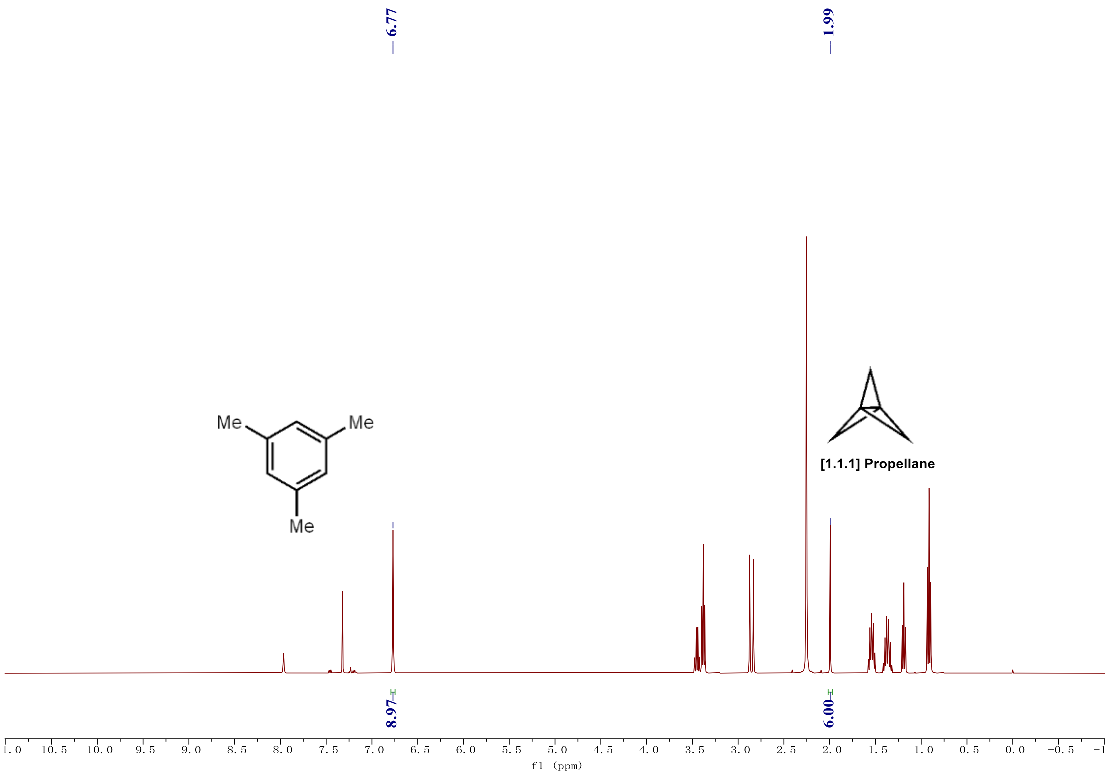

Supplementary Figure 12. ^{1}H NMR spectrum for the [1.1.1]propellane with mesitylene in $CDCl_3$

Preparation of [4.1.1]propellane

1-(4-Chlorobutyl)cyclopropan-1-ol (S6)

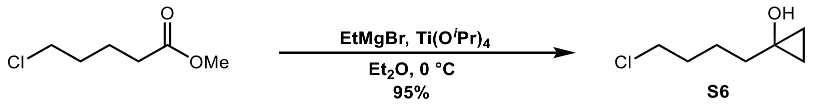

According to literature[9] with modified attempts, to a solution of methyl 5-chloropantanoate (86.3 mL, 600 mmol, 1.0 equiv.), and $Ti(O^iPr)_4$ (17.8 mL, 60 mmol, 0.1 equiv.) in anhydrous Et_2O (1 L) was cooled to 0 °C, and a solution of EtMgBr (500 mL, 1.5 mol, 2.5 equiv., 3.0 M in Et_2O) was added dropwise over 120 mins. The mixture was stirred for further 1 hour at 0 °C, then the mixture was slowly quenched by dropwise addition of 10% aqueous H_2SO_4. The organic layer was washed sequentially with aqueous $NaHCO_3$, brine,

dried over Na_2SO_4, filtered and concentrated *in vacuo* to afford **S6** (84.5 g, 568.8 mmol, 95%) as a colorless oil which was used without further purification.

The residue could be purified by flash column chromatography on silica gel (PE:Et_2O = 3:1) to afford pure **S6.**

^{1}H NMR (400 MHz, $CDCl_3$) δ 3.54 (t, *J* = 6.7 Hz, 2H), 1.87 – 1.74 (m, 3H), 1.70 – 1.61 (m, 2H), 1.59 – 1.52 (m, 2H), 0.76 – 0.69 (m, 2H), 0.46 – 0.38 (m, 2H).

^{13}C NMR (100 MHz, $CDCl_3$) δ 55.60, 45.17, 37.57, 32.57, 23.43, 13.54.

1-(4-Chlorobutyl)cyclopropyl ethanesulfonate (S7)

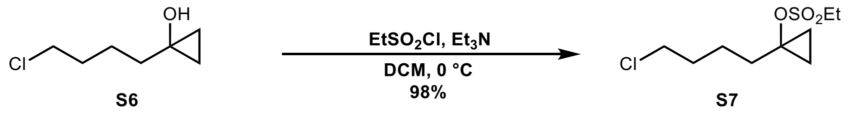

According to literature[9] with modified attempts, to a solution of **S6** (84.5 g, 568.8 mmol, 1.0 equiv.), and Et_3N (110.7 mL, 853.2 mmol, 1.5 equiv.) in anhydrous DCM (550 mL) was cooled to 0 ℃ and ethanesulfonyl chloride (59.3 mL, 625.7 mmol, 1.1 equiv.) was added dropwise over 1 hour. The mixture was stirred for further 30 mins at 0 ℃, then quenched by water. The organic layer was separated, and washed by 10% aqueous H_2SO_4, aqueous $NaHCO_3$, brine, dried over Na_2SO_4, filtered and concentrated *in vacuo* to afford **S7** (134.6 g, 559.1 mmol, 98%), as a black oil which was used without further purification.

The residue could be purified by flash column chromatography on silica gel (PE:Et_2O = 10:1) to afford pure **S7**.

^{1}H NMR (400 MHz, $CDCl_3$) δ 3.52 (t, *J* = 6.5 Hz, 2H), 3.04 (q, *J* = 7.4 Hz, 2H), 1.90 – 1.76 (m, 4H), 1.73 – 1.63 (m, 2H), 1.34 (t, *J* = 7.4 Hz, 3H), 1.26 – 1.15 (m, 2H), 0.74 – 0.62 (m, 2H).

^{13}C NMR (100 MHz, $CDCl_3$) δ 65.90, 46.85, 44.86, 35.41, 32.10, 23.03, 11.81, 8.18.

6-Chloro-2-(chloromethyl)hex-1-ene (S8)

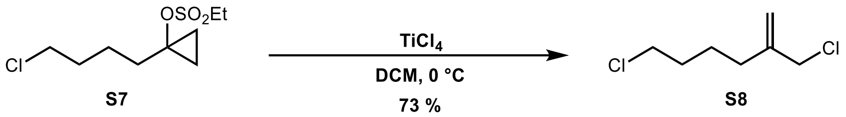

According to literature[10] with modified attempts, to a solution of 1-(4-chlorobutyl)cyclopropyl ethanesulfonate **S7** (65.0 g, 270 mmol, 1.0 equiv.) in anhydrous DCM (540 mL), was slowly added $TiCl_4$ (35.6 mL, 324 mmol, 1.2 equiv.), at 0 ℃ over 1hour. The mixture was stirred at 0 ℃ for 0.5 hours, the reaction was quenched by H_2O at 0 ℃ with vigorous stirring. The organic layer was washed by aqueous $NaHCO_3$, brine, dried over Na_2SO_4, filtered and concentrated *in vacuo* to afford **S8** (33.0 g, 197.6 mmol, 73%) as a dark yellow liquid which was used without further purification.

The residue could be purified by flash column chromatography on silica gel (PE) to afford pure **S8** and **S8 BP**.

^{1}H NMR (400 MHz, CDCl$_3$) δ 5.12 (s, 1H), 4.96 (t, *J* = 1.4 Hz, 1H), 4.02 (s, 2H), 3.54 (t, *J* = 6.6 Hz, 2H), 2.19 (t, *J* = 7.7 Hz, 2H), 1.84 – 1.73 (m, 2H), 1.68 – 1.56 (m, 2H).

^{13}C NMR (100 MHz, CDCl$_3$) δ 144.57, 114.72, 48.20, 44.82, 32.18, 32.07, 24.53.

1,2,6-trichloro-2-methylhexane (S8-BP)

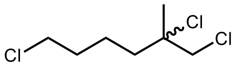

S8-BP

^{1}H NMR (400 MHz, CDCl$_3$) δ 3.79 – 3.63 (m, 2H), 3.55 (t, *J* = 6.6 Hz, 2H), 1.89 – 1.74 (m, 4H), 1.70 – 1.56 (m, 5H).

^{13}C NMR (100 MHz, CDCl$_3$) δ 71.17, 52.55, 44.74, 39.80, 32.40, 28.11, 21.74.

1,1-Dibromo-2-(4-chlorobutyl)-2-(chloromethyl)cyclopropane (S9)

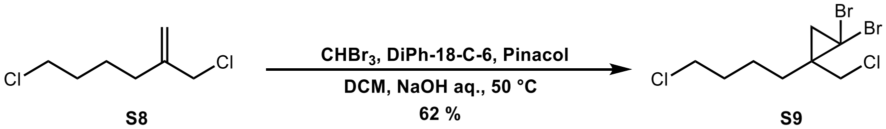

According to literature[8] with modified attempts, to stirred solution of **S8** (48.8g, 144 mmol, 1.0 equiv.), $CHBr_3$ (100 mL, 1.15 mol, 8.0 equiv.), dibenzo-18-crown-6 (2.6 g, 7.2 mmol,

0.05 equiv.), and pinacol (1.7 g, 14.4 mmol, 0.1 equiv.) in DCM (100 mL), was added ice cold 100% NaOH solution (71 mL) dropwise over 30 mins. The mixture was stirred for 6 hours at 70 °C, then cooled to room temperature and diluted with hexane and distilled water. The turbid liquid was filtered through a pad of celite to remove the precipitate. Additional water and hexane were added to the filtrate. The organic layer was separated, washed with brine, dried over Na_2SO_4, filtered and concentrated *in vacuo* affording a pale-yellow liquid. The crude product was purified by flash column chromatography on silica gel (Hexane) to afford pure **S9** (30.4g, 89.7 mmol, 62%) as a light yellow oil.

^{1}H NMR (400 MHz, $CDCl_3$) δ 3.77 (d, *J* = 1.2 Hz, 2H), 3.57 (t, *J* = 6.5 Hz, 2H), 1.91 – 1.77 (m, 4H), 1.75 – 1.53 (m, 4H).

^{13}C NMR (100 MHz, $CDCl_3$) δ 49.53, 44.79, 35.35, 34.04, 33.70, 32.93, 32.39, 23.45

[4.1.1]propellane

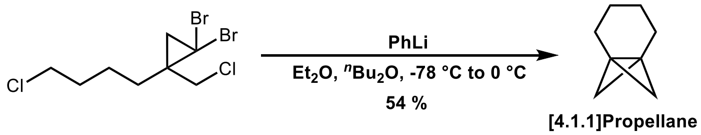

According to literature[6,8] with modified attempts, to an oven-dried round flask equipped with stir bar was added **S9** (4.07g, 12.0 mmol, 1.0 equiv.) and the oxygen was replaced by nitrogen with Schlenk line techniques. Anhydrous Et_2O (10 mL) and *n*-Bu_2O (13 mL) was added at -78 °C, then PhLi (22 mL, 42 mmol, 3.5 equiv., 1.9 M in *n*-Bu_2O) was slowly added. After 15 mins, the flask was moved to 30 °C and stirred. After 6 hours, vacuum distillation was applied to the flask with receiving flask condensation temperature at -78 °C with terminate pressure at 30 °C, 150 mbar to remove Et_2O. Then the remaining solution was heated up to 45 °C, and distillated by slowly reducing the applied pressure to < 10 mbar to afford a solution of [4.1.1]propellane in *n*-Bu_2O (12.3 mL) which was stored under an inert atmosphere at -20 °C. The yield was determined by ^{1}H NMR spectroscopy using DCM as an internal standard. The concentration of the [4.1.1]propellane solution was 0.52 M, with yield of 54%.

Determination of the Concentration of [4.1.1]propellane

In an 5mm NMR tube was added 50 μL (0.780 mmol) DCM and 100 μL the [4.1.1]propellane solution and filled with appropriate amount of $CDCl_3$. Integration of 4 [4.1.1]propellane protons (at ~1.9) are set to 4, and the 2 protons on mesitylene are integrated at ~5.2, the concentration of propellane could be calculated as follow equation:

$$C(propellane) = \frac{n}{v} = \frac{\frac{0.780}{Int(DCM)/2}}{0.1} = \frac{15.6}{Int(DCM)} = \frac{15.6}{30.02} = 0.52\ \mathrm{M}$$

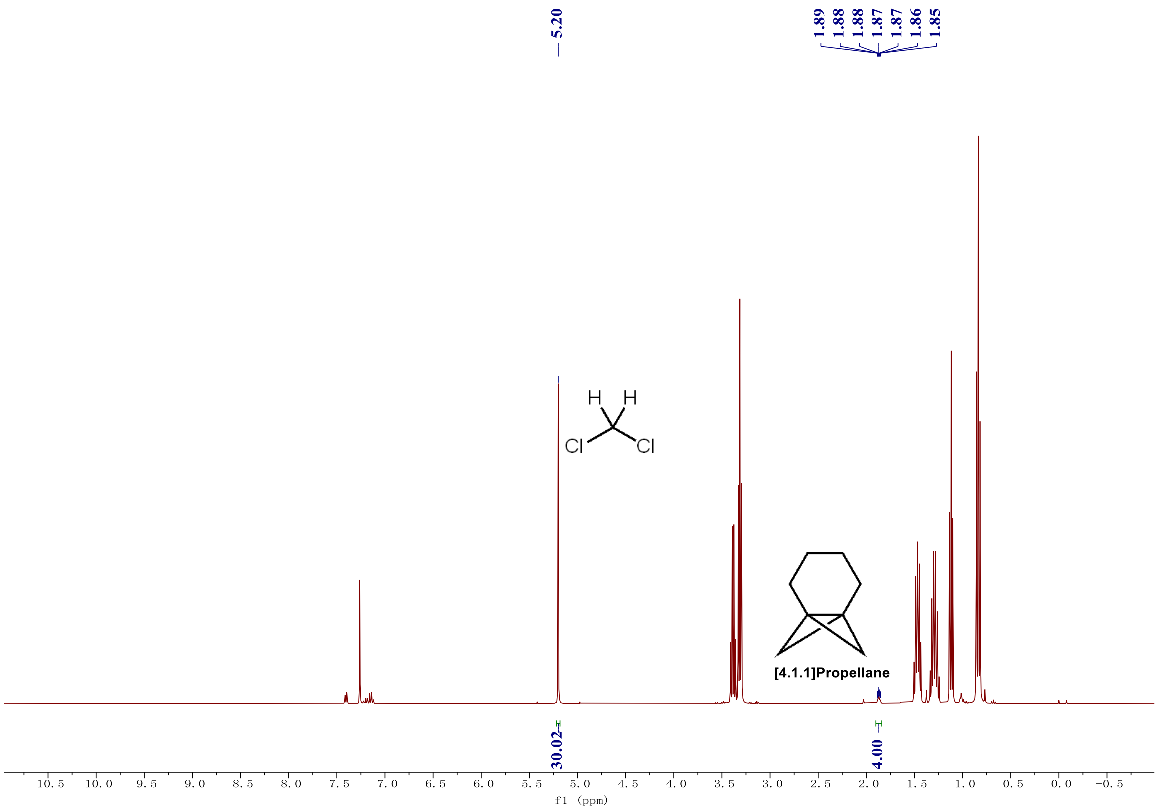

Supplementary Figure 13. ^{1}H NMR spectrum for the [4.1.1]propellane with DCM in $CDCl_3$

7) Reaction Optimization

To a 8 mL vial equipped with a stir bar was added photocatalyst **PCN (1)** (3.3 mg, 0.005 mmol, 10 mol%), *N*-(2-(trifluoromethyl)phenyl)acetamide (10.2 mg, 0.050 mmol, 1.0 equiv.), anhydrous cesium carbonate (19.5 mg, 0.060 mmol, 1.2 equiv.), γ-terpinene (40 μL, 0.25 mmol, 5.0 equiv.), anhydrous and degassed DMSO (2.0 mL) in the glovebox and stirring for 30 seconds. A solution of [1.1.1]propellane in DMF (1.2 M) (68 μL, 1.5 equiv.) was added at last, and the vial was quickly sealed with Parafilm and PVC tape. Subsequently, the solution was allowed to stirred for 2 mins to accelerate the dissolution of cesium carbonate. The reaction was stirred and irradiated using 40 W 427 nm blue LED lamps (5 cm away, with cooling fan & air-condition at 18 °C to maintain the reaction at room temperature) for 12 hours. The reaction was quenched by exposure to air. To a separated volumetric flash, benzotrifluoride (internal standard, 365.3 mg) was dissolved in 100 mL EA and well-distributed. The solution of benzotrifluoride (0.025 M, 2 mL, 1.0 equiv.) as internal standard was added then the reaction mixture was analyzed by ^{19}F NMR.

***N*-(2-(Bicyclo[1.1.1]pentan-1-yldifluoromethyl)phenyl)acetamide (6)**

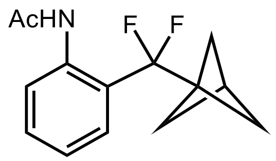

^{1}H NMR (400 MHz, CDCl$_3$) δ 8.05 (d, *J* = 8.0 Hz, 1H), 7.76 (s, 1H), 7.42 (t, *J* = 7.7 Hz, 1H), 7.37 (d, *J* = 7.8 Hz, 1H), 7.17 (t, *J* = 7.4 Hz, 1H), 2.51 (s, 1H), 2.18 (s, 3H), 1.84 (s, 6H).

^{13}C NMR (100 MHz, CDCl$_3$) δ 168.19, 134.71, 130.71, 126.38 (t, *J* = 8.3 Hz), 124.70 (t, *J* = 24.2 Hz), 124.51, 124.32, 120.06 (t, *J* = 242.3 Hz), 48.37 (t, *J* = 3.4 Hz), 45.82 (t, *J* = 35.1 Hz), 27.42, 24.89.

^{19}F NMR (376 MHz, CDCl$_3$) δ -99.06 (s, 2F).

HRMS (ESI) m/z calculated for $C_{14}H_{16}F_2NO^+$ ([M+H]$^+$): 252.1194, found 252.1190.

_N_-(2-([1,1'-Bi(bicyclo[1.1.1]pentan)]-3-yldifluoromethyl)phenyl)acetamide (7)

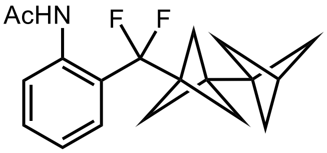

^{1}H NMR (500 MHz, CDCl$_3$) δ 7.68 (s, 1H), 7.63 (d, *J* = 8.2 Hz, 1H), 7.47 (s, 1H), 7.32 (t, *J* = 8.1 Hz, 1H), 7.11 (d, *J* = 7.8 Hz, 1H), 2.37 (d, *J* = 2.1 Hz, 1H), 2.17 (s, 3H), 1.60 (s, 6H), 1.59 (s, 6H).

^{13}C NMR (125 MHz, CDCl$_3$) δ 168.79, 138.01, 136.44 (t, *J* = 27.5 Hz), 129.01, 121.11 (t, *J* = 6.2 Hz), 121.03, 118.98 (d, *J* = 241.7 Hz), 116.82 (t, *J* = 6.1 Hz), 49.22, 47.23 (t, *J* = 2.9 Hz), 44.57, 40.46 (t, *J* = 36.2 Hz), 39.60, 26.70, 24.65.

^{19}F NMR (471 MHz, CDCl$_3$) δ -103.73 (s, 2F).

HRMS (ESI) m/z calculated for $C_{14}H_{16}F_2NO^+$ ($[M+H]^+$): 318.1664, found 318.1656.

Supplementary Table 1. Evaluation of reaction conditions. Yields determined by ^{19}F NMR.

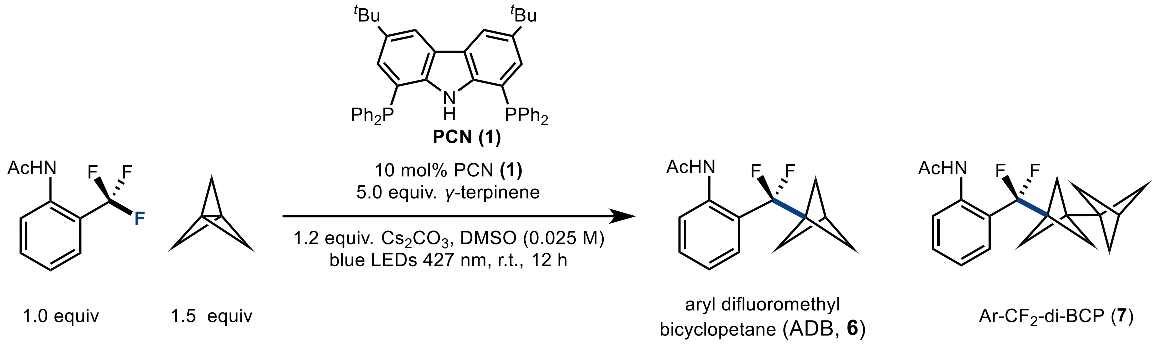

entry	deviations	$ArCF_3$ Remain(%)	ADB, **6**	**6** : **7**
1	standard conditions	**10%**	**74%**	**4.9 : 1**
2	3.0 equiv. propellane	23%	48%	2.8 : 1
3	5.0 equiv. propellane	20%	35%	1.6 : 1
4	0.56 M in Et_2O	11%	57%	9.5 : 1
5	0.79 M in *n*-Bu_2O	28%	47%	5.2 : 1
6	CsOH•H_2O as base	11%	56%	5.1 : 1
7	5 mol% PCN	22%	45%	5.0 : 1
8	390 nm LEDs	20%	30%	1.2 : 1
9	456 nm LEDs	22%	47%	3.1 : 1
10	no γ-terpinene	53%	11%	3.7 : 1
11	no base	100%	0%	-
12	no photocatalyst	82%	0%	-
13	no light	91%	0%	-

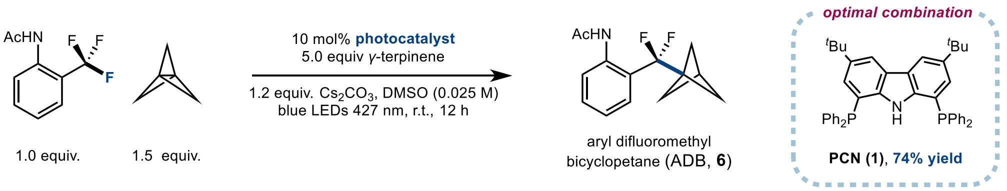

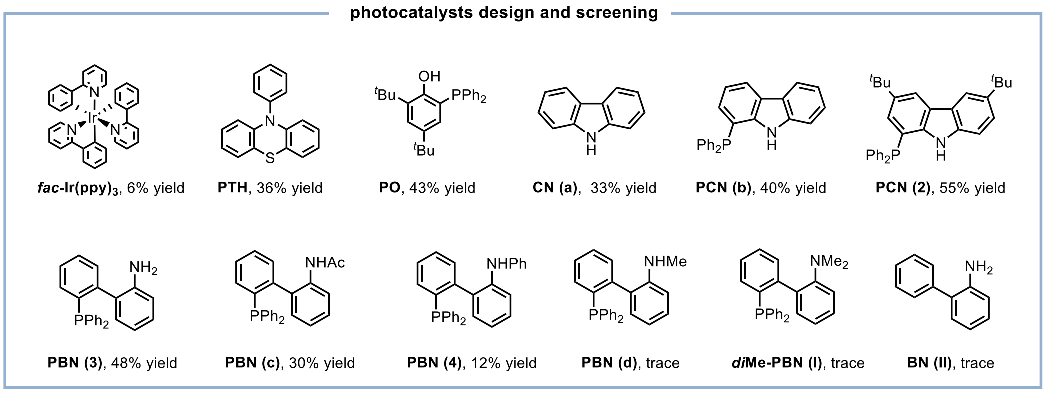

Supplementary Figure 14. Evaluation of photocatalyst. Yields determined by ^{19}F NMR.

Supplementary Table 2. Evaluation of reaction conditions. Yields determined by ^{19}F NMR. [a] Work-up with pinacol.

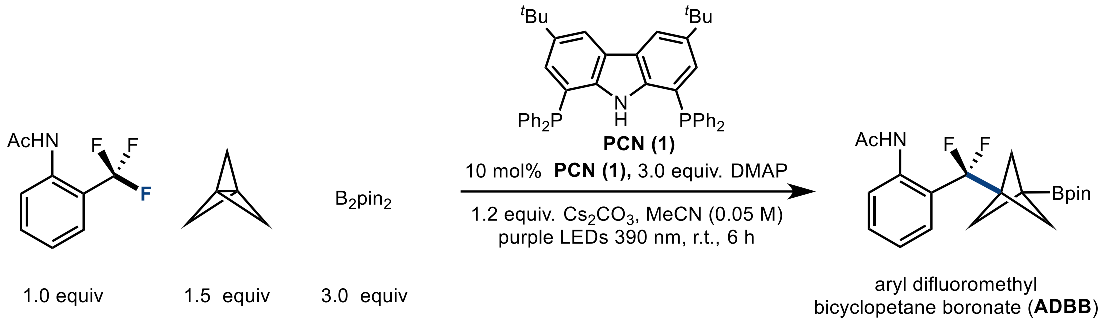

entry	deviations	$ArCF_3$ Remain(%)	**ADBB**
1	standard conditions	**10%**	**75%**
2[a]	B_2cat_2	89%	0%
3	Me_2PhSi-Bpin	12%	35%
4	427 nm LEDs	27%	53%
5	440 nm LEDs	34%	28%
6	no DMAP	33%	56%
7	no base	86%	7%
8	no photocatalyst	96%	2%
9	no light, 60 °C	100%	0%

Supplementary Table 3. Illustration of the double role of base. Yields determined by ^{19}F NMR.

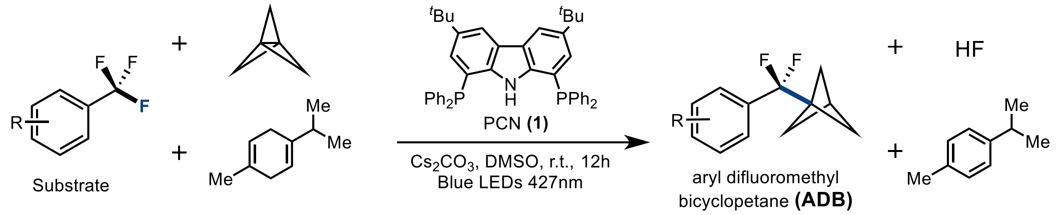

entry	Substrate	Base	Base Loading	ADB Yield
1		no Base	-	0%
2		^{t}BuOK	0.1 equiv.	15%
3		^{t}BuOK	0.2 equiv.	20%
4		^{t}BuOK	0.3 equiv.	24%
5	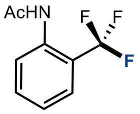	Cs_2CO_3	0.1 equiv.	51%
6		Cs_2CO_3	0.3 equiv.	56%
7		Cs_2CO_3	0.5 equiv.	61%
8		Cs_2CO_3	0.8 equiv.	65%
9		Cs_2CO_3	1.2 equiv.	**74%**
10		^{t}BuOK + Cs_2CO_3	0.1 + 0.2 equiv.	53%
11		^{t}BuOK + Cs_2CO_3	0.1 + 0.5 equiv.	63%
12		^{t}BuOK + Cs_2CO_3	0.1 + 1.0 equiv.	71%
13		^{t}BuOK + Cs_2CO_3	0.1 + 1.5 equiv.	58%
14		no base	-	0%
15		Cs_2CO_3	0.1 equiv.	74%
16	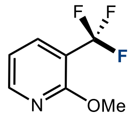	Cs_2CO_3	0.3 equiv.	**88%**
17		Cs_2CO_3	0.5 equiv.	60%
18		Cs_2CO_3	0.8 equiv.	77%
19		Cs_2CO_3	1.2 equiv.	84%
20		no base	-	0%
21		Cs_2CO_3	0.1 equiv.	12%
22	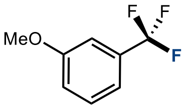	Cs_2CO_3	0.3 equiv.	29%
23		Cs_2CO_3	0.8 equiv.	46%
24		Cs_2CO_3	1.2 equiv.	**68%**

Under our investigation, when the base loading was cut off, the photocatalyst could not be activated and no ADB product was generated. However, when an equivalent inorganic base was loaded, ADB was successfully generated. In some cases, combining different bases or using 0.3 equivalents of base resulted in better yields, but loading 1.2 equivalents of base provided more generality.

We observed that increasing the base loading beyond the equivalent amount of catalyst helped to increase ADB yields. We believe that during the defluorination process, hydrogen fluoride is generated, and the base is needed to protect the catalyst anion from protonation. These experiments demonstrate the dual role of the base.

8) Quantum Yield Measurement

The procedure recently described by the Knowles group[11] (Princeton University) based on standard chemical actinometry[12] was first followed to determine the photon flux of the apparatus used in these experiments. The actinometry data we obtained is presented below.

Preparation of Potassium Ferrioxalate Solution

A 0.15 M solution of potassium ferrioxalate was prepared by dissolving 1.84 g, $K_3Fe(C_2O_4)_3 \cdot 3H_2O$ with the 25 mL H_2SO_4 solution (0.05 M) to a round flask covered with aluminum foil.

Preparation of Developer Solution

13.6 g of NaOAc and 1g 1,10-phenantroline was dissolved in 100 mL of 0.5 M H_2SO_4 solution to a round flask covered with aluminum foil.

Determination of Photon flux

A 1 cm × 1 cm quartz cuvette was charged with 2 mL of 0.15 M aqueous $K_3Fe(C_2O_4)_3$ solution. The solution was irradiated with 405 nm LEDs with an emission slit width of 10 nm. 10 μL aliquots of the solution were taken after 0 seconds, 1 minute, 3 minutes, and 6 minutes of irradiation. This aliquot was immediately added to 5 mL of a Developer Solution, and the vial was quickly wrapped in aluminum foil. The resulting solution was allowed to rest for 1 hour to allow the ferrous ions to completely coordinate with the 1,10-phenanthroline. The absorbance of all the actinometer samples was measured at 510 nm and recorded. The UV-Vis spectra of the actinometer samples and the time-course of absorbance at 510 nm were shown in Supplementary Figure 15.

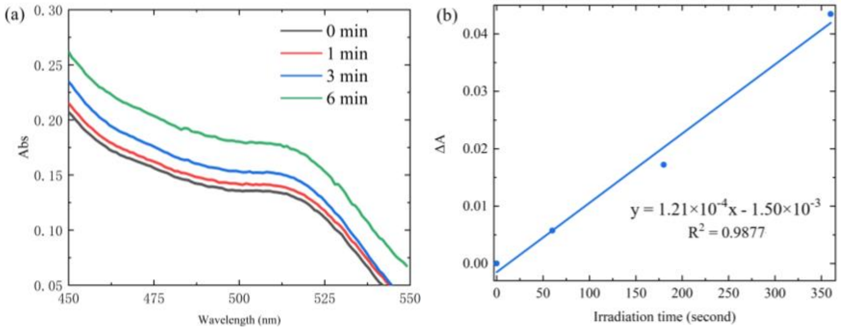

Supplementary Figure 15. (a) The UV-Vis Spectra of actinometer after irradiation of 402 nm for 0 second, 1min, 3 min, 6min. (b) Time-course of absorbance for actinometer at 510 nm

Determination of Photon Flux

The *photon flux* can be determined:

$$photon\ flux = \frac{mole\ Fe^{2+}}{\phi_{402\text{nm}} \cdot t \cdot F}$$

With number of Fe^{2+} ions produced by ferrioxalate photo-degradation calculated as follows:

$$mole\ Fe^{2+} = \frac{\Delta A_{510\text{nm}} \cdot V_1 \cdot V_3}{\varepsilon_{510\text{nm}} \cdot l \cdot V_2}$$

The *photon flux* can be determined by using follow equation:

$$photon\ flux = \frac{\Delta A_{510\text{nm}} \cdot V_1 \cdot V_3}{\phi_{402\text{nm}} \cdot \varepsilon_{510\text{nm}} \cdot l \cdot V_2 \cdot t \cdot F} = \frac{dA}{dt} \cdot \frac{V_1 \cdot V_3}{\phi_{402\text{nm}} \cdot \varepsilon_{510\text{nm}} \cdot l \cdot V_2 \cdot t \cdot F}$$

ΔA = difference in absorbance at 510 nm between sample and "0 second sample"

t = time of irradiation (second)

$\frac{dA}{dt}$ = slope of A and t

V_1 = total volume of irradiated solution (2 mL)

V_2 = volume of aliquot taken from V_1 (0.01 mL)

V_3 = the volume that V_2 diluted into (5 mL)

$\phi_{402\text{nm}}$ = 1.14 (reported literature value)[12]

$\varepsilon_{510\text{nm}}$ = Extinction coefficient of $Fe(phen)_3^{2+}$ complex at 510 nm (11,100 $M^{-1}cm^{-1}$)

F = mean fraction of light absorbed by the ferrioxalate solution (F ~ 1 at 405 nm at 0.15 M ferrioxalate)[12]

Thus, the photon flux can be calculated as follow:

$$photon\ flux = 1.21\times10^{-4}\times\frac{2\times5}{1.14\times11100\times1\times0.01\times1} = 9.56\times10^{-6}$$

Reaction Setup

Two 1 cm × 1 cm quartz cuvettes each equipped with a stir bar was added photocatalyst **PCN (1)** (3.3 mg, 0.005 mmol, 10 mol%), *N*-(2-(trifluoromethyl)phenyl)acetamide (10.2 mg, 0.050 mmol, 1.0 equiv.), anhydrous cesium carbonate (19.5 mg, 0.060 mmol, 1.2 equiv.), *γ*-terpinene (40 μL, 0.25 mmol, 5.0 equiv.), anhydrous and degassed DMSO (2.0 mL) in the glovebox and stirring for 30 seconds. A solution of [1.1.1]propellane in DMF (1.7 M) (44 μL, 1.5 equiv.) was added at last, and the vial was quickly sealed with Parafilm

and PVC tape. Subsequently, the solution was allowed to stirred for 2 mins to accelerate the dissolution of cesium carbonate. The reaction was stirred and irradiated using 402 nm blue LED lamps for 30min and 12 hours. The reaction was quenched by exposure to air. To a separated volumetric flash, benzotrifluoride (internal standard, 365.3 mg) was dissolved in 50 mL EA and well-distributed. The solution of benzotrifluoride (0.025 M, 1 mL, 1.0 equiv.) as internal standard was added then the reaction mixture was analyzed by ^{19}F NMR.

The yield after 30 min reaction is 2%, the yield after 12 hours is 35%

Determination of Quantum Yield

The Quantum Yield for 30 min and 12 h irradiation can be calculated as follow:

$$\phi_{402\text{nm, 30min}} = \frac{mol\ product}{photon\ flux \cdot t \cdot F} = \frac{0.02 \times 0.05 \times 0.001}{9.56 \times 10^{-6} \times 1800 \times 1} = 5.81 \times 10^{-6} < 1$$

$$\phi_{402\text{nm, 12h}} = \frac{mol\ product}{photon\ flux \cdot t \cdot F} = \frac{0.35 \times 0.05 \times 0.001}{9.56 \times 10^{-6} \times (3600 \times 12) \times 1} = 4.24 \times 10^{-6} < 1$$

The Quantum Yield of 30 min and 12 h irradiation is less than 1, which suggests that the catalytic mechanism does not involve a chain reaction[13, 14].

9) Reaction Scope Limitation

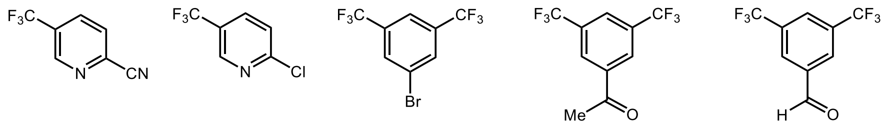

Supplementary Figure 16. Reaction scope limitation

10) Procedure for Construction of Aryl Difluoromethyl Bicyclopentane

Procedure A: To a 40 mL vial equipped with a stir bar was added *suitable* photocatalyst **PCN (1)** (38.9 mg, 0.06 mmol, 10 mol%) or **PBN (3)** (21.2 mg, 0.06 mmol, 10 mol%), substituted trifluoromethylbenzene (0.60 mmol, 1.0 equiv.), Cs_2CO_3 (234.6 mg, 0.72 mmol, 1.2 equiv.) or $CsOH{\cdot}H_2O$ (120.9 mg, 0.72 mmol, 1.2 equiv.), *γ*-terpinene (480 μL, 3.0 mmol, 5.0 equiv.), anhydrous and degassed DMSO (24.0 mL) in the glovebox and stirring for 30 seconds. A solution of **[1.1.1]propellane** in DMF (1.2 M) (744 μL, 1.20 mmol, 1.5 equiv.) was added at last, and the vial was quickly sealed with Parafilm and PVC tape. Subsequently, the solution was allowed to stirred for 2 mins to accelerate the dissolution of base. The reaction was stirred and irradiated using 40 W 427 nm blue LED lamps (5 cm away, with cooling fan & refrigeration air-condition at 18 ℃ to maintain the reaction at room temperature) for 12 hours. The reaction mixture was removed from the light, cooled to ambient temperature, quenched by exposure to air, diluted with water and EA, and the aqueous layer was extracted with three portions of EA. The combined organic layers were washed with brine, dried over Na_2SO_4, filtered, and concentrated. The residue was purified by flash chromatography on silica gel to afford the desired difluoromethyl bicyclo[1.1.1]pentanes (BCP) arenes.

NOTE: if substituted trifluoromethylbenzene was existing in the form of hydrochloride, an additional 1.0 equiv. of base needed to be added.

Procedure B: To a 40 mL vial equipped with a stir bar was added *suitable* photocatalyst **PCN (1)** (38.9 mg, 0.06 mmol, 10 mol%) or **PBN (3)** (21.2 mg, 0.06 mmol, 10 mol%), substituted trifluoromethylbenzene (0.60 mmol, 1.0 equiv.), *suitable* base Cs_2CO_3 (234.6 mg, 0.72 mmol, 1.2 equiv.) or $CsOH{\cdot}H_2O$ (120.9 mg, 0.72 mmol, 1.2 equiv.), *γ*-terpinene (480 μL, 3.0 mmol, 5.0 equiv.), anhydrous and degassed DMSO (24.0 mL) under nitrogen atmosphere (or nitrogen bubbling) and stirring for 30 seconds. A Solution of **[3.1.1]propellane** in *n*-Bu_2O (0.54 M) (2.2 mL, 1.20 mmol, 2.0 equiv.) was added at last, and the vial was quickly sealed with Parafilm and PVC tape. Subsequently, the solution was allowed to stirred for 2 mins to accelerate the dissolution of base. The reaction was stirred and irradiated using 40 W 427 nm blue LED lamps (5 cm away, with cooling fan &

refrigeration air-condition at 18 °C to maintain the reaction at room temperature) for 12 hours. The reaction mixture was removed from the light, cooled to ambient temperature, quenched by exposure to air. diluted with water and EA, and the aqueous layer was extracted with three portions of EA. The combined organic layers were washed with brine, dried over Na_2SO_4, filtered, and concentrated. The residue was purified by flash chromatography on silica gel to afford the desired difluoromethyl BCP arenes.

Procedure C: To a 40 mL vial equipped with a stir bar was added *suitable* photocatalyst **PCN (1)** (38.9 mg, 0.06 mmol, 10 mol%) or **PBN (3)** (21.2 mg, 0.06 mmol, 10 mol%), substituted trifluoromethylbenzene (0.60 mmol, 1.0 equiv.), *suitable* base Cs_2CO_3 (234.6 mg, 0.72 mmol, 1.2 equiv.) or $CsOH{\cdot}H_2O$ (120.9 mg, 0.72 mmol, 1.2 equiv.), γ-terpinene (480 μL, 3.0 mmol, 5.0 equiv.), anhydrous and degassed DMSO (24.0 mL) under nitrogen atmosphere (or nitrogen bubbling) and stirring for 30 seconds. A Solution of **[4.1.1]propellane** in *n*-Bu_2O (0.52 M) (2.3 mL, 1.20 mmol, 2.0 equiv.) was added at last, and the vial was quickly sealed with Parafilm and PVC tape. Subsequently, the solution was allowed to stirred for 2 mins to accelerate the dissolution of base. The reaction was stirred and irradiated using 40 W 427 nm blue LED lamps (5 cm away, with cooling fan & refrigeration air-condition at 18 °C to maintain the reaction at room temperature) for 12 hours. The reaction mixture was removed from the light, cooled to ambient temperature, quenched by exposure to air. diluted with water and EA, and the aqueous layer was extracted with three portions of EA. The combined organic layers were washed with brine, dried over Na_2SO_4, filtered, and concentrated. The residue was purified by flash chromatography on silica gel to afford the desired difluoromethyl BCP arenes.

Procedure for three-component coupling

Procedure D: To a 40 mL vial equipped with a stir bar was added photocatalyst **PCN (1)** (77.8 mg, 0.12 mmol, 10 mol%), substituted trifluoromethylbenzene (1.20 mmol, 1.0 equiv.), Cs_2CO_3 (312.8 mg, 0.96 mmol, 0.8 equiv.), B_2pin_2 (917.7 mg, 3.60 mmol, 3.0 equiv.), 4-Dimethylaminopyridine (439.2 mg, 3.60 mmol, 3.0 equiv.) anhydrous and degassed MeCN (24.0 mL) under nitrogen atmosphere (or nitrogen bubbling) and stirring for 30 seconds. A Solution of **[1.1.1]propellane** in DMF (2.5 M) (720 μL, 1.80 mmol, 1.5

equiv.) was added at last, and the vial was quickly sealed with Parafilm and PVC tape. Subsequently, the solution was allowed to stirred for 2 mins to accelerate the dissolution of base. The reaction was stirred and irradiated using 40 W 390 nm purple LED lamps (5 cm away, with cooling fan & refrigeration air-condition at 18 °C to maintain the reaction at room temperature) for 6 hours. The reaction mixture was removed from the light, cooled to ambient temperature, quenched by exposure to air. diluted with water and EA, and the aqueous layer was extracted with three portions of EA. The combined organic layers were washed with brine, dried over Na_2SO_4, filtered, and concentrated. The residue was purified by flash chromatography on silica gel to afford the desired difluoromethyl BCP boronates.

Procedure E: To a 40 mL vial equipped with a stir bar was added photocatalyst **PCN (1)** (77.8 mg, 0.12 mmol, 10 mol%), substituted trifluoromethylbenzene (1.20 mmol, 1.0 equiv.), Cs_2CO_3 (312.8 mg, 0.96 mmol, 0.8 equiv.), B_2pin_2 (3.047 g, 12.0 mmol, 10.0 equiv.), anhydrous and degassed MeCN (24.0 mL) under nitrogen atmosphere (or nitrogen bubbling) and stirring for 30 seconds. A Solution of **[3.1.1]propellane** in *n*-Bu_2O (0.54 M) (3.3 mL, 1.80 mmol, 1.5 equiv.) or **[4.1.1]propellane** in *n*-Bu_2O (0.52 M) (3.5 mL, 1.80 mmol, 1.5 equiv.) was added at last, and the vial was quickly sealed with Parafilm and PVC tape. Subsequently, the solution was allowed to stirred for 2 mins to accelerate the dissolution of base. The reaction was stirred and irradiated using 40 W 390 nm purple LED lamps (5 cm away, with cooling fan & refrigeration air-condition at 18 °C to maintain the reaction at room temperature) for 6 hours. The reaction mixture was removed from the light, cooled to ambient temperature, quenched by exposure to air. diluted with water and EA, and the aqueous layer was extracted with three portions of EA. The combined organic layers were washed with brine, dried over Na_2SO_4, filtered, and concentrated. The residue was purified by flash chromatography on silica gel to afford the desired difluoromethyl BCP boronates.

Suggestion for Reaction Duplication:

1. Make sure the deoxygenation process is complete.
2. The propellane solution should be transparent and added last. The irradiation should be given as soon as possible.

3. The reaction time should be controlled; otherwise, the product may undergo further defluorination.

4. For substrates with acidic hydrogen, try **PCN (1)** with **Cs_2CO_3** first; for substrates without acidic hydrogen, try **PBN (3)** with **$CsOH{\cdot}H_2O$** first.

5. After accessed the pure product, stored at -20℃ to prevent oxidation.

6. The quality and concentration of propellane play a pivotal role in the reaction, so try a higher concentration and suitable propellane solvents.

11) Experimental Data

***N*- (2-(Bicyclo[1.1.1]pentan-1-yldifluoromethyl)phenyl)acetamide (6)**

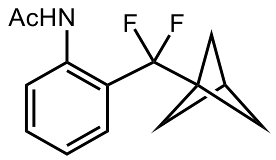

Following the **Procedure A** using **PCN (1)** and Cs_2CO_3 as suitable reagents.
Purified by flash chromatography (PE:EA = 3:1) on silica gel to afford the title compound (98.8 mg, 65% yield) as a pale yellow solid.

^{1}H NMR (400 MHz, $CDCl_3$) δ 8.05 (d, *J* = 8.0 Hz, 1H), 7.76 (s, 1H), 7.42 (t, *J* = 7.7 Hz, 1H), 7.37 (d, *J* = 7.8 Hz, 1H), 7.17 (t, *J* = 7.4 Hz, 1H), 2.51 (s, 1H), 2.18 (s, 3H), 1.84 (s, 6H).

^{13}C NMR (100 MHz, $CDCl_3$) δ 168.19, 134.71, 130.71, 126.38 (t, *J* = 8.3 Hz), 124.70 (t, *J* = 24.2 Hz), 124.51, 124.32, 120.06 (t, *J* = 242.3 Hz), 48.37 (t, *J* = 3.4 Hz), 45.82 (t, *J* = 35.1 Hz), 27.42, 24.89.

^{19}F NMR (376 MHz, $CDCl_3$) δ -99.06 (s, 2F).

HRMS (ESI) m/z calculated for $C_{14}H_{16}F_2NO^+$ ($[M+H]^+$): 252.1194, found 252.1190.

***N*- (3-(Bicyclo[1.1.1]pentan-1-yldifluoromethyl)phenyl)acetamide (12)**

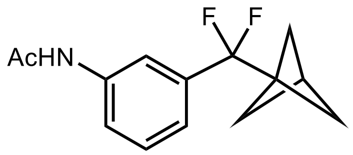

Following the **Procedure A** using **PCN (1)** and Cs_2CO_3 as suitable reagents.
Purified by flash chromatography (PE:EA = 2:1) on silica gel to afford the title compound (82.0 mg, 54% yield) as a pale yellow solid.

^{1}H NMR (500 MHz, CD_2Cl_2) δ 7.97 (s, 1H), 7.64 (d, *J* = 7.6 Hz, 1H), 7.55 (s, 1H), 7.35 (t, *J* = 7.5 Hz, 1H), 7.12 (d, *J* = 7.2 Hz, 1H), 2.51 (s, 1H), 2.15 (s, 3H), 1.82 (s, 6H).

^{13}C NMR (125 MHz, CD_2Cl_2) δ 169.21, 138.77, 136.42 (t, J = 27.0 Hz), 129.26, 121.25, 121.11 (t, J = 5.9 Hz), 118.89 (t, J = 242.0 Hz), 116.88 (t, J = 6.3 Hz), 48.43 (t, J = 3.4 Hz), 46.37 (t, J = 36.1 Hz), 27.87, 24.67.

^{19}F NMR (471 MHz, CD_2Cl_2) δ -103.70 (s, 2F).

HRMS (ESI) m/z calculated for $C_{14}H_{16}F_2NO^+$ ($[M+H]^+$): 252.1194, found 252.1188.

***N*- (4-(Bicyclo[1.1.1]pentan-1-yldifluoromethyl)phenyl)acetamide (13)**

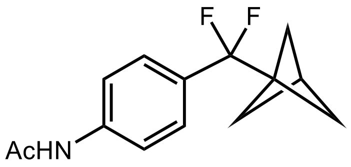

Following the **Procedure A** using **PCN (1)** and Cs_2CO_3 as suitable reagents.
Purified by flash chromatography (PE:EA = 1:1) on silica gel to afford the title compound (98.0 mg, 65% yield) as a pale yellow oil.

^{1}H NMR (400 MHz, $CDCl_3$) δ 7.54 (d, J = 8.4 Hz, 2H), 7.38 (s, 1H), 7.34 (d, J = 8.5 Hz, 2H), 2.52 (s, 1H), 2.19 (s, 3H), 1.81 (s, 6H).

^{13}C NMR (100 MHz, $CDCl_3$) δ 168.54, 139.10, 131.31 (t, J = 27.3 Hz), 126.21 (t, J = 6.1 Hz), 119.33, 118.58 (t, J = 242.2 Hz), 48.20 (t, J = 3.4 Hz), 46.34 (t, J = 36.7 Hz), 27.66, 24.79.

^{19}F NMR (376 MHz, $CDCl_3$) δ -103.28 (s, 2F).

HRMS (ESI) m/z calculated for $C_{14}H_{16}F_2NO^+$ ($[M+H]^+$): 252.1194, found 252.1188.

3-(Bicyclo[1.1.1]pentan-1-yldifluoromethyl)-*N*-phenylaniline (14)

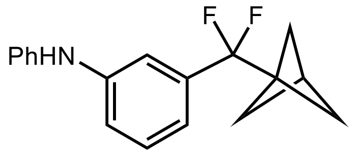

Following the **Procedure A** using **PCN (1)** and Cs_2CO_3 as suitable reagents.

Purified by flash chromatography (PE:EA = 30:1) on silica gel to afford the title compound (82.7 mg, 48% yield) as a pale yellow oil.

^{1}H NMR (400 MHz, $CDCl_3$) δ 7.33 – 7.24 (m, 2H), 7.13 – 7.05 (m, 4H), 7.00 – 6.92 (m, 2H), 5.76 (s, 1H), 2.53 (s, 1H), 1.85 (s, 6H).

^{13}C NMR (100 MHz, $CDCl_3$) δ 143.32, 142.70, 136.79 (t, *J* = 26.9 Hz), 129.58, 129.37, 121.70, 118.59 (d, *J* = 243.0 Hz), 118.43 (d, *J* = 1.8 Hz), 118.34, 117.77 (t, *J* = 6.1 Hz), 114.34 (t, *J* = 6.3 Hz), 48.27 (t, *J* = 3.6 Hz), 46.31, 27.57 (t, *J* = 1.9 Hz).

^{19}F NMR (376 MHz, $CDCl_3$) δ -103.76 (s, 2F).

HRMS (ESI) m/z calculated for $C_{18}H_{18}F_2N^+$ ($[M+H]^+$): 286.1402, found 286.1398.

3-(Bicyclo[1.1.1]pentan-1-yldifluoromethyl)-*N,N*-dimethylaniline (15)

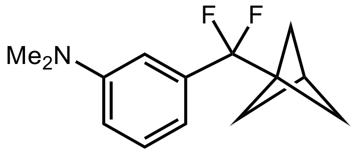

Following the **Procedure A** using **PBN (3)** and $CsOH·H_2O$ as suitable reagents.

Purified by flash chromatography (PE:DCM = 30:1) on silica gel to afford the title compound (59.8 mg, 42% yield) as a colorless oil.

^{1}H NMR (400 MHz, $CDCl_3$) δ 7.26 (t, *J* = 7.9 Hz, 1H), 6.78 – 6.71 (m, 3H), 2.97 (s, 6H), 2.52 (s, 1H), 1.85 (s, 6H).

^{13}C NMR (100 MHz, CDCl$_3$) δ 150.39, 136.24 (t, *J* = 26.3 Hz), 128.98, 119.00 (t, *J* = 242.3 Hz), 113.50 (t, *J* = 6.2 Hz), 113.46 (t, *J* = 1.4 Hz), 109.20 (t, *J* = 6.5 Hz), 48.29 (t, *J* = 3.7 Hz), 46.44 (t, *J* = 36.7 Hz), 40.67, 27.38 (t, *J* = 2.2 Hz).

^{19}F NMR (376 MHz, CDCl$_3$) δ -103.63 (s, 2F).

HRMS (ESI) m/z calculated for $C_{14}H_{18}F_2N^+$ ($[M+H]^+$): 238.1402, found 238.1398.

3-(Bicyclo[1.1.1]pentan-1-yldifluoromethyl)aniline (16)

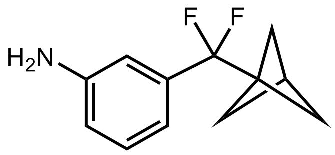

Following the **Procedure A** using **PCN (1)** and Cs_2CO_3 as suitable reagents.
Purified by flash chromatography (PE:DCM = 3:1) on silica gel to afford the title compound (72.5 mg, 53% yield) as a colorless oil.

^{1}H NMR (400 MHz, CDCl$_3$) δ 7.17 (t, 1H), 6.77 (d, *J* = 7.6 Hz, 1H), 6.73 – 6.69 (m, 2H), 3.75 (s, 2H), 2.51 (s, 1H), 1.82 (s, 6H).

^{13}C NMR (100 MHz, CDCl$_3$) δ 146.29, 136.55, 129.27, 118.71 (t, *J* = 242.2 Hz), 116.12 (t, *J* = 1.6 Hz), 115.58 (t, *J* = 6.1 Hz), 111.74 (t, *J* = 6.4 Hz), 48.17 (t, *J* = 3.4 Hz), 46.20 (t, *J* = 36.4 Hz), 27.47 (t, *J* = 2.0 Hz).

^{19}F NMR (376 MHz, CDCl$_3$) δ -103.86 (s, 2F).

HRMS (ESI) m/z calculated for $C_{12}H_{14}F_2N^+$ ($[M+H]^+$): 210.1089, found 210.1084.

1-(Difluoro(3-methoxyphenyl)methyl)bicyclo[1.1.1]pentane (17)

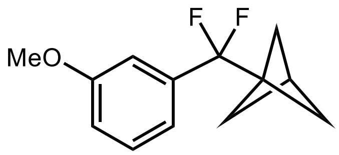

Following the **Procedure A** using **PBN (3)** and $CsOH{\cdot}H_2O$ as suitable reagents.
Purified by flash chromatography (PE = 100%) on silica gel to afford the title compound (74.0 mg, 55% yield) as a colorless oil.

^{1}H NMR (400 MHz, $CDCl_3$) δ 7.32 (t, *J* = 7.5 Hz, 1H), 7.00 – 6.91 (m, 3H), 3.83 (s, 3H), 2.53 (s, 1H), 1.84 (s, 6H).

^{13}C NMR (100 MHz, $CDCl_3$) δ 159.52, 136.97 (t, *J* = 27.0 Hz), 129.45, 118.52 (t, *J* = 242.5 Hz), 117.70 (t, *J* = 6.1 Hz), 115.12 (d, *J* = 1.8 Hz), 111.00 (t, *J* = 6.4 Hz), 55.44, 48.23 (t, *J* = 3.5 Hz), 46.27 (t, *J* = 36.3 Hz), 27.52 (t, *J* = 2.1 Hz).

^{19}F NMR (376 MHz, $CDCl_3$) δ -103.67 (s, 2F).

HRMS (EI) m/z calculated for $C_{13}H_{14}F_2O^+$ ($[M]^+$): 224.1007, found 224.1005.

1-(Difluoro(4-methoxyphenyl)methyl)bicyclo[1.1.1]pentane (18)

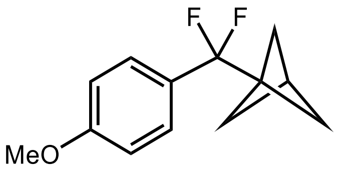

Following the **Procedure A** using **PBN (3)** and $CsOH{\cdot}H_2O$ as suitable reagents.
Purified by flash chromatography (PE = 100%) on silica gel to afford the title compound (47.1 mg, 35% yield) as a colorless oil.

^{1}H NMR (400 MHz, $CDCl_3$) δ 7.32 (d, *J* = 8.8 Hz, 2H), 6.91 (d, *J* = 8.6 Hz, 2H), 3.83 (s, 3H), 2.52 (s, 1H), 1.82 (s, 6H).

^{13}C NMR (100 MHz, $CDCl_3$) δ 160.51, 127.84 (t, *J* = 27.6 Hz), 126.75, 118.87 (t, *J* = 242.0 Hz), 113.62, 55.42, 48.20 (t, *J* = 3.4 Hz), 46.52 (t, *J* = 37.2 Hz), 27.61 (t, *J* = 2.1 Hz).

^{19}F NMR (376 MHz, $CDCl_3$) δ -102.49 (s, 2F).

HRMS (EI) m/z calculated for $C_{13}H_{14}F_2O^+$ ($[M]^+$): 224.1007, found 224.1008.

1-(Difluoro(4-phenoxyphenyl)methyl)bicyclo[1.1.1]pentane (19)

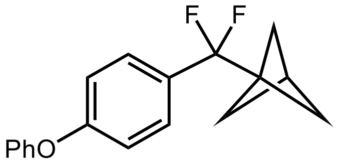

Following the **Procedure A** using **PBN (3)** and CsOH·H_2O as suitable reagents.
Purified by flash chromatography (PE = 100%) on silica gel to afford the title compound (86.9 mg, 51% yield) as a colorless needle-like crystal.

^{1}H NMR (400 MHz, CDCl$_3$) δ 7.40 – 7.31 (m, 4H), 7.15 (t, *J* = 7.4 Hz, 1H), 7.06 – 6.96 (m, 4H), 2.40 (s, 1H), 1.62 (s, 6H), 1.62 (s, 6H).

^{13}C NMR (100 MHz, CDCl$_3$) δ 158.64, 156.55, 130.25 (t, *J* = 34.6 Hz), 130.03, 127.01 (t, *J* = 6.0 Hz), 124.02, 119.63, 117.99 (t, *J* = 245.9 Hz), 117.94, 49.26, 47.23 (t, *J* = 3.2 Hz), 44.64, 40.63 (t, *J* = 36.9 Hz), 39.67 (t, *J* = 2.1 Hz), 26.74.

^{19}F NMR (376 MHz, CDCl$_3$) δ -102.63 (s, 2F).

HRMS (EI) m/z calculated for $C_{18}H_{16}F_2O^+$ ($[M]^+$): 268.1164, found 286.1164.

1-(Difluoro(m-tolyl)methyl)bicyclo[1.1.1]pentane (20)

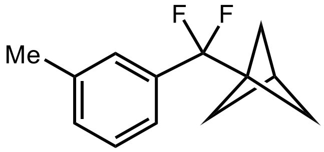

Following the **Procedure A** using **PBN (3)** and CsOH·H_2O as suitable reagents.
Purified by flash chromatography (PE = 100%) on silica gel to afford the title compound (37.6 mg, 30% yield) as a colorless oil.

^{1}H NMR (400 MHz, CDCl$_3$) δ 7.29 (dd, *J* = 8.1, 7.1 Hz, 1H), 7.20 (t, *J* = 8.8 Hz, 3H), 2.52 (s, 1H), 2.39 (s, 3H), 1.83 (s, 6H).

^{13}C NMR (100 MHz, CDCl$_3$) δ 138.01, 135.46 (t, *J* = 26.6 Hz), 130.32 (t, *J* = 1.6 Hz), 128.18, 125.82 (t, *J* = 6.1 Hz), 122.47 (t, *J* = 6.1 Hz), 118.75 (t, *J* = 241.8 Hz), 48.22 (t, *J* = 3.5 Hz), 46.37 (t, *J* = 36.5 Hz), 27.60 (t, *J* = 2.0 Hz), 21.62.

^{19}F NMR (376 MHz, CDCl$_3$) δ -103.61 (s, 2F).

HRMS (EI) m/z calculated for $C_{13}H_{13}F_2^+$ ([M-H]$^+$): 207.0980, found 207.0979.

1-(Difluoro(2-(trifluoromethyl)phenyl)methyl)bicyclo[1.1.1]pentane (21)

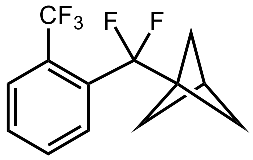

Following the **Procedure A** using **PBN (3)** and CsOH·H_2O as suitable reagents.
Purified by flash chromatography (PE = 100%) on silica gel to afford the title compound (76.0 mg, 48% yield) as a colorless oil.

^{1}H NMR (400 MHz, CDCl$_3$) δ 7.78 (d, *J* = 7.8 Hz, 1H), 7.61 (t, *J* = 7.6 Hz, 1H), 7.52 (t, *J* = 8.6 Hz, 2H), 2.50 (s, 1H), 1.85 (s, 6H).

^{13}C NMR (100 MHz, CDCl$_3$) δ 133.95 (td, *J* = 27.8, 1.9 Hz), 131.57, 129.79, 129.11 (t, *J* = 8.3 Hz), 127.65, 127.28 (q, *J* = 6.6 Hz), 121.66 (q, *J* = 273.5, 272.9 Hz), 118.54 (t, *J* = 244.4 Hz), 48.76 (t, *J* = 3.5 Hz), 46.62 (t, *J* = 35.3 Hz), 27.10.

^{19}F NMR (376 MHz, CDCl$_3$) δ -57.91 (t, *J* = 18.1 Hz, 3F), -97.70 (q, *J* = 18.1 Hz, 2F).

HRMS (EI) m/z calculated for $C_{13}H_{10}F_5^+$ ([M-H]$^+$): 261.0697, found 261.0697.

1-(Difluoro(3-(trifluoromethyl)phenyl)methyl)bicyclo[1.1.1]pentane (22)

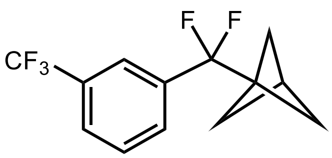

Following the **Procedure A** using **PBN (3)** and $CsOH{\cdot}H_2O$ as suitable reagents.
Purified by flash chromatography (PE = 100%) on silica gel to afford the title compound (67.6 mg, 43% yield) as a colorless oil.

^{1}H NMR (400 MHz, $CDCl_3$) δ 7.71 – 7.65 (m, 2H), 7.64 – 7.51 (m, 2H), 2.56 (s, 1H), 1.84 (s, 6H).

^{13}C NMR (100 MHz, $CDCl_3$) δ 136.59 (t, *J* = 27.8 Hz), 131.00 (q, *J* = 32.7 Hz), 129.00 (t, *J* = 12.6 Hz), 128.80 (t, *J* = 5.5 Hz), 126.54 (dt, *J* = 5.3, 1.4 Hz), 123.94 (q, *J* = 272.3 Hz), 122.48 – 122.14 (m), 118.03 (t, *J* = 243.0 Hz), 48.16 (t, *J* = 3.5 Hz), 46.05 (t, *J* = 35.7 Hz), 27.80 (t, *J* = 2.0 Hz).

^{19}F NMR (376 MHz, $CDCl_3$) δ -62.79 (s, 3F), -104.10 (s, 2F).

HRMS (ESI) m/z calculated for $C_{13}H_{10}F_5^+$ ($[M-H]^+$): 261.0697, found 261.0699.

(3-(Bicyclo[1.1.1]pentan-1-yldifluoromethyl)phenyl)methanol (23)

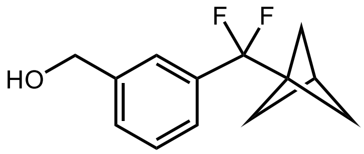

Following the **Procedure A** using **PCN (1)** and Cs_2CO_3 as suitable reagents.
Purified by flash chromatography (PE:EA = 5:1) on silica gel to afford the title compound (70.1 mg, 52% yield) as a colorless oil.

^{1}H NMR (400 MHz, $CDCl_3$) δ 7.40 (dd, *J* = 4.8, 1.9 Hz, 3H), 7.35 – 7.28 (m, 1H), 4.70 (s, 2H), 2.52 (s, 1H), 1.83 (s, 6H).

^{13}C NMR (100 MHz, $CDCl_3$) δ 141.09, 135.84 (t, *J* = 27.0 Hz), 128.55, 128.09, 124.63 (t, *J* = 6.0 Hz), 123.66 (t, *J* = 6.1 Hz), 118.63 (t, *J* = 242.3 Hz), 65.04, 48.19 (t, *J* = 3.5 Hz), 46.27 (t, *J* = 36.3 Hz), 27.63 (t, *J* = 2.0 Hz).

^{19}F NMR (376 MHz, $CDCl_3$) δ -103.51 (s, 2F).

HRMS (APCI) m/z calculated for $C_{13}H_{13}F_2^+$ ($[M-H_2O+H]^+$): 207.0985, found 207.0991.

2-(2-(Bicyclo[1.1.1]pentan-1-yldifluoromethyl)phenyl)ethan-1-ol (24)

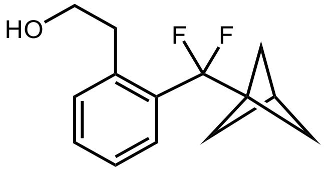

Following the **Procedure A** using **PCN (1)** and Cs_2CO_3 as suitable reagents.
Purified by flash chromatography (PE:EA = 5:1) on silica gel to afford the title compound (53.0 mg, 35% yield) as a colorless oil.

^{1}H NMR (400 MHz, $CDCl_3$) δ 7.41 – 7.32 (m, 2H), 7.31 – 7.24 (m, 3H), 3.83 (t, *J* = 6.9 Hz, 2H), 2.93 (td, *J* = 6.9, 3.5 Hz, 2H), 2.51 (s, 1H), 1.83 (s, 6H).

^{13}C NMR (100 MHz, $CDCl_3$) δ 136.12 (t, *J* = 2.2 Hz), 133.67 (t, *J* = 25.1 Hz), 131.79, 129.61, 126.96 (t, *J* = 8.5 Hz), 126.44, 120.12 (t, *J* = 243.4 Hz), 64.87 – 63.89 (m), 48.50 (t, *J* = 3.5 Hz), 46.70 (t, *J* = 35.9 Hz), 37.35 (t, *J* = 3.0 Hz), 27.44 (t, *J* = 2.1 Hz).

^{19}F NMR (376 MHz, $CDCl_3$) δ -97.50 (s, 2F).

HRMS (APCI) m/z calculated for $C_{14}H_{15}F_2^+$ ($[M-H_2O+H]^+$): 221.1142, found 221.1145.

2-(3-(Bicyclo[1.1.1]pentan-1-yldifluoromethyl)phenyl)ethan-1-ol (25)

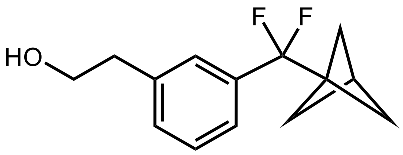

Following the **Procedure A** using **PCN (1)** and Cs_2CO_3 as suitable reagents.
Purified by flash chromatography (PE:EA = 5:1) on silica gel to afford the title compound (46.0 mg, 32% yield) as a colorless oil.

^{1}H NMR (400 MHz, CDCl$_3$) δ 7.35 (t, *J* = 7.7 Hz, 1H), 7.29 – 7.24 (m, 3H), 3.86 (t, *J* = 6.6 Hz, 2H), 2.89 (t, *J* = 6.6 Hz, 2H), 2.52 (s, 1H), 1.82 (s, 6H).

^{13}C NMR (100 MHz, CDCl$_3$) δ 138.75, 135.76 (t, *J* = 26.9 Hz), 130.30 (t, *J* = 1.8 Hz), 128.52, 125.82 (t, *J* = 6.0 Hz), 123.52 (t, *J* = 6.0 Hz), 118.64 (t, *J* = 242.3 Hz), 63.65, 48.17 (t, *J* = 3.5 Hz), 46.27 (t, *J* = 36.4 Hz), 39.20, 27.61 (t, *J* = 2.1 Hz).

^{19}F NMR (376 MHz, CDCl$_3$) δ -103.60 (s, 2F).

HRMS (APCI) m/z calculated for $C_{14}H_{15}F_2^+$ ([M-H_2O+H]$^+$): 221.1142, found 221.1138.

3-(3-(Bicyclo[1.1.1]pentan-1-yldifluoromethyl)phenyl)propan-1-ol (26)

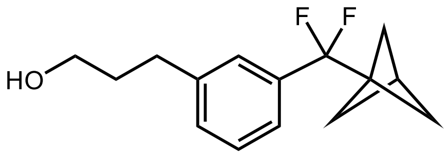

Following the **Procedure A** using **PCN (1)** and Cs_2CO_3 as suitable reagents.
Purified by flash chromatography (PE:EA = 6:1) on silica gel to afford the title compound (54.8 mg, 36% yield) as a colorless oil.

^{1}H NMR (400 MHz, CDCl$_3$) δ 7.32 (t, *J* = 7.8 Hz, 1H), 7.27 – 7.18 (m, 3H), 3.67 (t, *J* = 6.4 Hz, 2H), 2.74 (t, *J* = 7.6 Hz, 2H), 2.52 (s, 1H), 1.89 (p, *J* = 7.8, 7.3, 6.7, 6.4 Hz, 1H), 1.82 (s, 6H).

^{13}C NMR (100 MHz, CDCl$_3$) δ 141.98, 135.55 (t, *J* = 26.7 Hz), 129.94 – 129.35 (m), 128.35, 125.23 (t, *J* = 6.0 Hz), 122.92 (t, *J* = 6.1 Hz), 118.74 (t, *J* = 242.2 Hz), 62.20, 48.16 (t, *J* = 3.5 Hz), 46.32 (t, *J* = 36.4 Hz), 34.26, 32.10, 27.59 (t, *J* = 2.1 Hz).

^{19}F NMR (376 MHz, CDCl$_3$) δ -103.72 (s, 2F).

HRMS (ESI) m/z calculated for $C_{15}H_{17}FO^+$ ([M-HF-H]$^+$): 232.1258, found 232.1258.

1-(Difluoro(2-fluoro-5-methoxyphenyl)methyl)bicyclo[1.1.1]pentane (27)

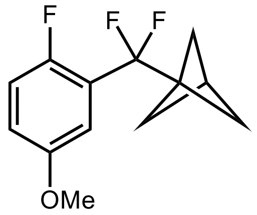

Following the **Procedure A** using **PBN (3)** and $CsOH·H_2O$ as suitable reagents.

Purified by flash chromatography (PE = 100%) on silica gel to afford the title compound (108.3 mg, 74% yield) as a colorless oil.

^{1}H NMR (400 MHz, CDCl$_3$) δ 7.02 (t, *J* = 9.4 Hz, 1H), 6.94 – 6.87 (m, 2H), 3.80 (s, 3H), 2.50 (s, 1H), 1.87 (s, 6H).

^{13}C NMR (100 MHz, CDCl$_3$) δ 155.48 (d, *J* = 2.2 Hz), 153.85 (dt, *J* = 244.6, 3.9 Hz), 123.34 (td, *J* = 27.6, 14.8 Hz), 117.54 (td, *J* = 243.6, 2.7 Hz), 117.22 (d, *J* = 23.8 Hz), 116.52 (d, *J* = 8.0 Hz), 112.26 (td, *J* = 7.9, 3.0 Hz), 55.94, 48.55 (td, *J* = 3.8, 1.5 Hz), 45.89 (t, *J* = 35.0 Hz), 27.62 – 26.34 (m).

^{19}F NMR (376 MHz, CDCl$_3$) δ -102.09 (d, *J* = 15.0 Hz, 2F), -124.68 (tdt, *J* = 15.0, 9.7, 4.7 Hz, 1F).

HRMS (EI) m/z calculated for $C_{13}H_{13}F_3O^+$ ($[M]^+$): 242.0913, found 242.0912.

ethyl 3-(bicyclo[1.1.1]pentan-1-yldifluoromethyl)-1-methyl-1H-pyrazole-4-carboxylate (28)

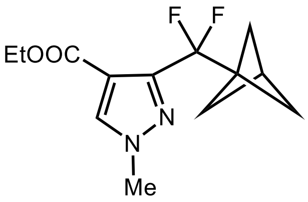

Following the **Procedure A** using **PBN (3)** and $CsOH·H_2O$ as suitable reagents.

Purified by flash chromatography (PE = 100%) on silica gel to afford the title compound (139.3 mg, 86% yield) as a colorless oil.

^{1}H NMR (400 MHz, $CDCl_3$) 7.87 (s, 1H), 4.23 (q, *J* = 7.1 Hz, 2H), 3.88 (s, 3H), 2.46 (s, 1H), 1.91 (s, 6H), 1.29 (t, *J* = 7.1 Hz, 3H)..

^{13}C NMR (100 MHz, $CDCl_3$) δ 161.52, 146.36 (t, *J* = 32.2 Hz), 135.98, 115.42 (t, *J* = 240.1 Hz), 113.14, 60.45, 48.95 (t, *J* = 3.5 Hz), 45.81 (t, *J* = 34.0 Hz), 39.56, 27.32 (t, *J* = 2.1 Hz), 14.26.

^{19}F NMR (376 MHz, $CDCl_3$) δ -99.18 (s, 3F).

HRMS (ESI) m/z calculated for $C_{13}H_{17}F_2N_2O_2^+$ ($[M+H]^+$): 271.1253, found 271.1238.

(2-(Bicyclo[1.1.1]pentan-1-yldifluoromethyl)-6-fluorophenyl)methanamine (29)

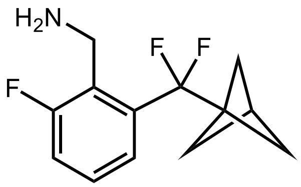

Following the **Procedure A** using **PCN (1)** and Cs_2CO_3 as suitable reagents.
Purified by flash chromatography (PE:EA:Et_3N = 4:2:1) on silica gel to afford the title compound (79.3 mg, 55% yield) as a pale yellow oil.

^{1}H NMR (500 MHz, CD_2Cl_2) δ 7.29 (q, *J* = 7.4 Hz, 1H), 7.22 – 7.11 (m, 2H), 3.82 (s, 2H), 2.52 (s, 1H), 1.83 (s, 6H).

^{13}C NMR (125 MHz, CD_2Cl_2) δ 162.37 (d, *J* = 244.6 Hz), 135.15 (td, *J* = 25.7, 4.0 Hz), 128.53 (d, *J* = 9.2 Hz), 122.65 (td, *J* = 8.8, 3.6 Hz), 120.02 (td, *J* = 243.9, 2.5 Hz), 117.20 (d, *J* = 23.3 Hz), 48.69 (t, *J* = 3.3 Hz), 46.59 (t, *J* = 35.5 Hz), 37.83, 27.74.

^{19}F NMR (471 MHz, CD_2Cl_2) δ -97.11 (s, 2F), -118.83 (t, *J* = 7.5 Hz, 1F).

HRMS (ESI) m/z calculated for $C_{13}H_{15}F_3N^+$ ($[M+H]^+$): 242.1151, found 242.1147.

2-(Bicyclo[1.1.1]pentan-1-yldifluoromethyl)pyridine (30)

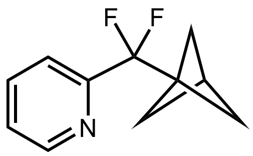

Following the **Procedure A** using **PBN (3)** and CsOH·H_2O as suitable reagents.
Purified by flash chromatography (PE:EA = 15:1) on silica gel to afford the title compound (84.9 mg, 73% yield) as a pale yellow oil.

^{1}H NMR (500 MHz, CD_2Cl_2) δ 8.64 (s, 1H), 7.78 (t, *J* = 8.0 Hz, 2H), 7.51 (d, *J* = 7.4 Hz, 3H), 7.39 – 7.31 (m, 2H), 2.51 (s, 1H), 1.89 (s, 6H).

^{13}C NMR (125 MHz, CD_2Cl_2) δ 154.01 (t, *J* = 28.8 Hz), 149.71, 137.11, 124.77, 120.51 (t, *J* = 4.6 Hz), 116.95 (t, *J* = 242.0 Hz), 48.91 (t, *J* = 3.5 Hz), 45.71 (t, *J* = 34.2 Hz), 28.15 (d, *J* = 2.3 Hz).

^{19}F NMR (471 MHz, CD_2Cl_2) δ -106.80 (s, 2F).

HRMS (ESI) m/z calculated for $C_{11}H_{12}F_2N^+$ ($[M+H]^+$): 196.0932, found 196.0929.

4-(Bicyclo[1.1.1]pentan-1-yldifluoromethyl)pyridine (31)

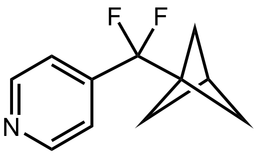

Following the **Procedure A** using **PBN (3)** and CsOH·H_2O as suitable reagents.
Purified by flash chromatography (PE:EA = 10:1) on silica gel to afford the title compound (58.6 mg, 50% yield) as a pale yellow oil.

^{1}H NMR (400 MHz, $CDCl_3$) δ 8.71 – 8.66 (m, 2H), 7.30 – 7.27 (m, 2H), 2.54 (s, 1H), 1.82 (s, 6H).

^{13}C NMR (100 MHz, CDCl$_3$) δ 150.17, 143.58 (t, *J* = 28.5 Hz), 119.94 (t, *J* = 5.6 Hz), 117.31 (t, *J* = 242.8 Hz), 48.10 (t, *J* = 3.5 Hz), 45.54 (t, *J* = 34.8 Hz), 27.80 (t, *J* = 2.1 Hz).

^{19}F NMR (376 MHz, CDCl$_3$) δ -106.56 (s, 2F).

HRMS (ESI) m/z calculated for $C_{11}H_{12}F_2N^+$ ([M+H]$^+$): 196.0932, found 196.0929.

3-(Bicyclo[1.1.1]pentan-1-yldifluoromethyl)-2-fluoroaniline (32)

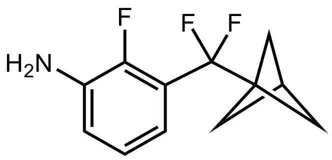

Following the **Procedure A** using **PCN (1)** and Cs_2CO_3 as suitable reagents.
Purified by flash chromatography (PE:DCM = 3:1) on silica gel to afford the title compound (72.5 mg, 53% yield) as a pale yellow oil.

^{1}H NMR (400 MHz, CDCl$_3$) δ 6.94 (t, *J* = 7.8 Hz, 1H), 6.83 (td, *J* = 8.1, 1.7 Hz, 1H), 6.77 (td, *J* = 7.0, 1.6 Hz, 1H), 3.80 (s, 2H), 2.50 (s, 1H), 1.87 (s, 6H).

^{13}C NMR (100 MHz, CDCl$_3$) δ 148.36 (dt, *J* = 244.9, 3.9 Hz), 135.30 (d, *J* = 12.8 Hz), 123.98 (d, *J* = 4.3 Hz), 122.88 (td, *J* = 27.5, 10.7 Hz), 118.50 (d, *J* = 3.8 Hz), 117.90 (td, *J* = 243.1, 1.8 Hz), 116.09 (td, *J* = 7.6, 1.7 Hz), 48.51 (td, *J* = 3.3, 1.3 Hz), 45.99 (t, *J* = 35.1 Hz), 27.01 (d, *J* = 1.9 Hz).

^{19}F NMR (376 MHz, CDCl$_3$) δ -101.61 (d, *J* = 15.8 Hz, 2F), -136.90 (tt, *J* = 15.3, 7.3 Hz, 1F).

HRMS (ESI) m/z calculated for $C_{12}H_{13}F_3N^+$ ([M+H]$^+$): 228.0995, found 288.0990.

3-(Bicyclo[1.1.1]pentan-1-yldifluoromethyl)-2-methoxypyridine (33)

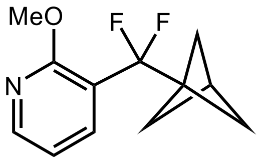

Following the **Procedure A** using **PBN (3)** and $CsOH \cdot H_2O$ as suitable reagents.
Purified by flash chromatography (PE:EA = 30:1) on silica gel to afford the title compound (83.2 mg, 65% yield) as a colorless oil.

^{1}H NMR (500 MHz, $CDCl_3$) δ 8.21 – 8.18 (m, 1H), 7.69 (dd, *J* = 7.5, 1.9 Hz, 1H), 6.91 (dd, *J* = 7.4, 5.0 Hz, 1H), 3.96 (s, 3H), 2.45 (s, 1H), 1.86 (s, 6H).

^{13}C NMR (125 MHz, $CDCl_3$) δ 189.09, 176.55 (d, *J* = 1.8 Hz), 164.34 (t, *J* = 7.9 Hz), 146.19 (t, *J* = 27.6 Hz), 145.83 (t, *J* = 242.8 Hz), 144.44, 81.58, 77.16 (t, *J* = 3.5 Hz), 74.04 (t, *J* = 35.0 Hz), 54.97 (t, *J* = 2.1 Hz).

^{19}F NMR (471 MHz, $CDCl_3$) δ -102.62 (s, 2F).

HRMS (ESI) m/z calculated for $C_{12}H_{14}F_2NO^+$ ($[M+H]^+$): 226.1038, found 226.1032.

3-(Bicyclo[1.1.1]pentan-1-yldifluoromethyl)-2-methoxypyridine (34)

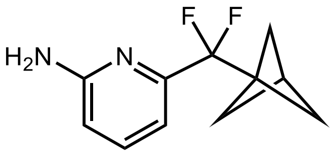

Following the **Procedure A** using **PCN (1)** and Cs_2CO_3 as suitable reagents.
Purified by flash chromatography (PE:EA = 7:1) on silica gel to afford the title compound (78.7 mg, 62% yield) as a pale yellow solid.

^{1}H NMR (400 MHz, $CDCl_3$) δ 7.45 (t, *J* = 7.8 Hz, 1H), 6.80 (d, *J* = 7.4 Hz, 1H), 6.49 (d, *J* = 8.3 Hz, 1H), 4.85 (s, 2H), 2.48 (s, 1H), 1.87 (s, 6H).

^{13}C NMR (100 MHz, CDCl$_3$) δ 158.31, 152.03 (t, *J* = 28.3 Hz), 138.24, 116.54 (t, *J* = 242.3 Hz), 110.08 (t, *J* = 5.0 Hz), 109.60 (d, *J* = 1.7 Hz), 48.64 (t, *J* = 3.6 Hz), 45.48 (t, *J* = 34.7 Hz), 27.57 (t, *J* = 2.1 Hz).

^{19}F NMR (376 MHz, CDCl$_3$) δ -107.40 (s, 2F).

HRMS (ESI) m/z calculated for $C_{11}H_{13}F_2N_2$+ ([M+H]$^+$): 211.1041, found 211.1036.

(2-(Bicyclo[1.1.1]pentan-1-yldifluoromethyl)pyridin-3-yl)methanol (35)

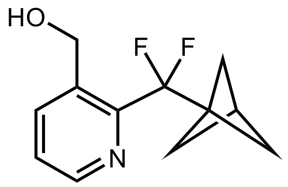

Following the **Procedure A** using **PCN (1)** and Cs_2CO_3 as suitable reagents.
Purified by flash chromatography (PE:EA = 3:1) on silica gel to afford the title compound (114.2 mg, 85% yield) as a pale yellow oil.

^{1}H NMR (400 MHz, CDCl$_3$) δ 8.50 (dd, *J* = 4.8, 1.6 Hz, 1H), 8.04 (dd, *J* = 7.9, 1.6 Hz, 1H), 7.35 (dd, *J* = 7.9, 4.7 Hz, 1H), 4.82 (s, 2H), 3.20 (d, *J* = 4.9 Hz, 1H), 2.50 (s, 1H), 1.88 (s, 6H).

^{13}C NMR (100 MHz, CDCl$_3$) δ 178.17 (t, *J* = 28.5 Hz), 175.78, 165.32, 163.87, 153.16, 146.68 (t, *J* = 242.7 Hz), 89.11 (t, *J* = 7.7 Hz), 77.16 (t, *J* = 3.6 Hz), 73.93 (t, *J* = 34.0 Hz), 56.42 (t, *J* = 2.1 Hz).

^{19}F NMR (376 MHz, CDCl$_3$) δ -101.24 (s, 2F).

HRMS (ESI) m/z calculated for $C_{12}H_{14}F_2NO^+$ ([M+H]$^+$): 226.1038, found 226.1034.

***tert*-Butyl 4-(bicyclo[1.1.1]pentan-1-yldifluoromethyl)-1*H*-indole-1-carboxylate (36)**

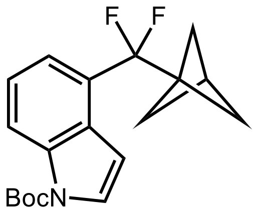

Following the **Procedure A** using **PCN (1)** and Cs_2CO_3 as suitable reagents.

Purified by flash chromatography (PE:DCM = 30:1) on silica gel to afford the title compound (144.4 mg, 72% yield) as a pale yellow solid.

^{1}H NMR (400 MHz, $CDCl_3$) δ 8.25 (d, *J* = 8.2 Hz, 1H), 7.62 (d, *J* = 3.8 Hz, 1H), 7.33 (t, *J* = 7.9 Hz, 1H), 7.26 (dd, *J* = 7.6, 1.0 Hz, 1H), 6.70 (q, *J* = 3.0 Hz, 1H), 2.48 (s, 1H), 1.81 (s, 6H), 1.68 (s, 9H).

^{13}C NMR (100 MHz, $CDCl_3$) δ 149.70, 135.73, 127.70 (t, *J* = 27.6 Hz), 127.52 (t, *J* = 3.1 Hz), 126.49, 123.82, 119.96 (t, *J* = 7.1 Hz), 119.63 (t, *J* = 242.7 Hz), 116.54, 107.00 (t, *J* = 3.7 Hz), 84.11, 48.46 (t, *J* = 3.4 Hz), 46.71 (t, *J* = 36.2 Hz), 28.30, 27.17 (t, *J* = 2.1 Hz).

^{19}F NMR (376 MHz, $CDCl_3$) δ -101.67 (s, 2F).

HRMS (ESI) m/z calculated for $C_{19}H_{22}F_2NO_2^+$ ($[M+H]^+$): 334.1613, found 334.1620.

***tert*-Butyl 5-(bicyclo[1.1.1]pentan-1-yldifluoromethyl)-1*H*-indole-1-carboxylate (37)**

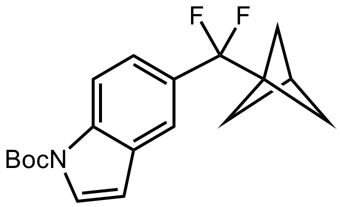

Following the **Procedure A** using **PCN (1)** and Cs_2CO_3 as suitable reagents.

Purified by flash chromatography (PE:DCM = 30:1) on silica gel to afford the title compound (138.6 mg, 69% yield) as a pale yellow solid.

^{1}H NMR (400 MHz, CDCl$_3$) δ 8.16 (d, *J* = 8.7 Hz, 1H), 7.64 (d, *J* = 3.7 Hz, 1H), 7.61 (s, 0H), 7.33 (dd, *J* = 8.7, 1.8 Hz, 1H), 6.61 (d, *J* = 3.7 Hz, 1H), 2.52 (s, 1H), 1.83 (s, 6H), 1.68 (s, 9H).

^{13}C NMR (100 MHz, CDCl$_3$) δ 149.72, 130.24, 129.97 (d, *J* = 27.1 Hz), 126.92, 121.53 (t, *J* = 5.8 Hz), 119.22 (t, *J* = 242.4 Hz), 118.03 (t, *J* = 6.5 Hz), 114.92, 107.57, 84.19, 48.23 (t, *J* = 3.4 Hz), 46.63 (t, *J* = 36.9 Hz), 28.30, 27.60 (t, *J* = 1.9 Hz).

^{19}F NMR (376 MHz, CDCl$_3$) δ -102.33 (s, 2F).

HRMS (ESI) m/z calculated for $C_{19}H_{22}F_2NO_2^+$ ([M+H]$^+$): 334.1613, found 334.1611.

***tert*-Butyl 6-(bicyclo[1.1.1]pentan-1-yldifluoromethyl)-1*H*-indole-1-carboxylate (38)**

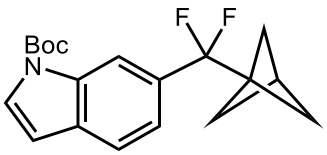

Following the **Procedure A** using **PCN (1)** and Cs_2CO_3 as suitable reagents.
Purified by flash chromatography (PE:EA = 30:1) on silica gel to afford the title compound (102.1 mg, 51% yield) as a pale yellow solid.

^{1}H NMR (400 MHz, CDCl$_3$) δ 8.22 (s, 1H), 7.67 (d, *J* = 3.3 Hz, 1H), 7.60 – 7.55 (m, 1H), 7.26 (dd, *J* = 8.1, 1.4 Hz, 1H), 6.59 (d, *J* = 3.6 Hz, 1H), 2.52 (s, 1H), 1.85 (s, 6H), 1.68 (s, 9H).

^{13}C NMR (100 MHz, CDCl$_3$) δ 149.67, 134.68, 132.00 – 130.91 (m), 127.37, 120.72, 119.90 (t, *J* = 5.9 Hz), 121.93 – 116.59 (m), 112.58 (t, *J* = 7.2 Hz), 107.10, 84.21, 48.28 (t, *J* = 3.4 Hz), 46.60 (t, *J* = 36.9 Hz), 28.30, 27.60 (t, *J* = 1.9 Hz).

^{19}F NMR (376 MHz, CDCl$_3$) δ -101.99 (s, 2F).

HRMS (ESI) m/z calculated for $C_{19}H_{22}F_2NO_2^+$ ([M+H]$^+$): 334.1613, found 344.1610.

***tert*-Butyl 7-(bicyclo[1.1.1]pentan-1-yldifluoromethyl)-1*H*-indole-1-carboxylate (39)**

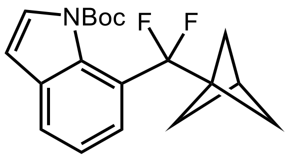

Following the **Procedure A** using **PCN (1)** and Cs_2CO_3 as suitable reagents.
Purified by flash chromatography (PE:EA = 50:1) on silica gel to afford the title compound (108.1 mg, 54% yield) as a pale yellow solid.

^{1}H NMR (400 MHz, CDCl$_3$) δ 7.61 – 7.57 (m, 1H), 7.41 (d, *J* = 3.6 Hz, 1H), 7.30 – 7.21 (m, 2H), 6.56 (d, *J* = 3.6 Hz, 1H), 2.51 (s, 1H), 2.08 (s, 6H), 1.61 (s, 9H).

^{13}C NMR (100 MHz, CDCl$_3$) δ 150.47, 133.05, 131.44, 129.24, 124.17 (t, *J* = 7.5 Hz), 124.06 (t, *J* = 28.4 Hz), 122.46 (t, *J* = 1.6 Hz), 122.27, 119.75 (t, *J* = 241.5 Hz), 106.62, 83.58, 49.84, 46.91 (t, *J* = 36.0 Hz), 27.92, 26.98 (t, *J* = 1.8 Hz).

^{19}F NMR (376 MHz, CDCl$_3$) δ -90.16 (s, 2F).

HRMS (ESI) m/z calculated for $C_{19}H_{22}F_2NO_2^+$ ($[M+H]^+$): 334.1613, found 344.1609.

(±) 1-(1,2,2,2-Tetrafluoro-1-(4-phenoxyphenyl)ethyl)bicyclo[1.1.1]pentane (40)

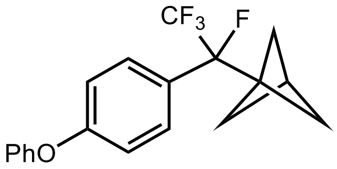

Following the **Procedure A** using **PBN (3)** and $CsOH \cdot H_2O$ as suitable reagents.
Purified by flash chromatography (PE = 100%) on silica gel to afford the title compound (123.3 mg, 61% yield) as a colorless oil.

^{1}H NMR (400 MHz, CDCl$_3$) δ 7.43 – 7.35 (m, 4H), 7.17 (t, *J* = 7.3 Hz, 1H), 7.10 – 7.00 (m, 4H), 2.56 (s, 1H), 1.94 (s, 6H).

^{13}C NMR (100 MHz, CDCl$_3$) δ 158.24, 156.46, 130.04, 127.78 (d, *J* = 22.2 Hz), 126.86 (dq, *J* = 10.5, 1.9 Hz), 124.07, 123.47 (dq, *J* = 286.0, 29.9 Hz), 119.72, 117.97 (d, *J* = 2.0 Hz), 92.72 (dq, *J* = 184.7, 30.7 Hz), 49.55 (d, *J* = 4.7 Hz), 44.85 (d, *J* = 27.9 Hz), 27.97.

^{19}F NMR (376 MHz, CDCl$_3$) δ -76.02 (d, *J* = 8.1 Hz, 3F), -172.73 (q, *J* = 8.2 Hz, 1F).

HRMS (EI) m/z calculated for $C_{19}H_{16}F_4O^+$ ($[M]^+$): 336.1132, found 336.1133.

1-(Difluoro(3-methoxy-5-(trifluoromethyl)phenyl)methyl)bicyclo[1.1.1]pentane (41)

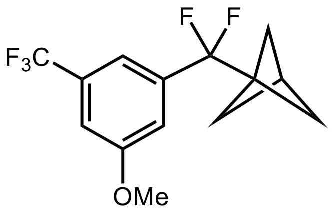

Following the **Procedure A** using **PBN (3)** and CsOH·H_2O as suitable reagents.
Purified by flash chromatography (PE = 100%) on silica gel to afford the title compound (124.9 mg, 71% yield) as a pale yellow oil.

^{1}H NMR (400 MHz, CDCl$_3$) δ 7.23 (s, 1H), 7.17 (s, 1H), 7.08 (s, 1H), 3.87 (s, 3H), 2.55 (s, 1H), 1.84 (s, 6H).

^{13}C NMR (100 MHz, CDCl$_3$) δ 159.87, 138.04 (t, *J* = 27.7 Hz), 132.20 (d, *J* = 32.9 Hz), 123.59 (q, *J* = 273.0 Hz), 117.88 (t, *J* = 243.0 Hz), 114.72 (t, *J* = 5.8 Hz), 114.66 – 114.32 (m), 111.92 (q, *J* = 3.8 Hz), 55.85, 48.20, 45.96 (t, *J* = 35.8 Hz), 27.67 (t, *J* = 1.8 Hz).

^{19}F NMR (376 MHz, CDCl$_3$) δ -62.81 (s, 3F), -103.90 (s, 2F).

HRMS (EI) m/z calculated for $C_{14}H_{13}F_5O^+$ ($[M]^+$): 292.0881, found 2292.0883.

1-(Difluoro(3-phenoxy-5-(trifluoromethyl)phenyl)methyl)bicyclo[1.1.1]pentane (42)

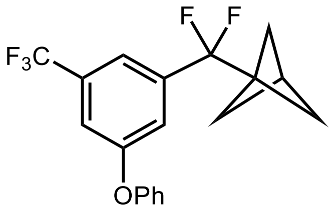

Following the **Procedure A** using **PBN (3)** and $CsOH\cdot H_2O$ as suitable reagents.
Purified by flash chromatography (PE = 100%) on silica gel to afford the title compound (95.5 mg, 40% yield) as a pale yellow oil.

^{1}H NMR (400 MHz, $CDCl_3$) δ 7.45 – 7.35 (m, 3H), 7.28 (s, 1H), 7.24 – 7.18 (m, 1H), 7.09 – 7.02 (m, 2H), 2.56 (s, 1H), 1.84 (s, 6H).

^{13}C NMR (100 MHz, $CDCl_3$) δ 158.23, 155.82, 138.51 (t, J = 28.1 Hz), 132.58 (q, J = 33.2 Hz), 130.36, 124.81, 123.49 (q, J = 272.8 Hz), 119.68, 118.60 (t, J = 6.0 Hz), 117.67 (t, J = 243.5 Hz), 116.52 (td, J = 6.2, 3.9 Hz), 116.21 (q, J = 3.6 Hz), 48.18 (t, J = 3.4 Hz), 45.89 (t, J = 35.6 Hz), 27.76 (t, J = 1.9 Hz).

^{19}F NMR (376 MHz, $CDCl_3$) δ -62.80 (s, 3F), -103.87 (s, 2F).

HRMS (EI) m/z calculated for $C_{19}H_{15}F_5O^+$ ($[M]^+$): 354.1038, found 354.1034.

(3-(Bicyclo[1.1.1]pentan-1-yldifluoromethyl)-5-(trifluoromethyl)phenyl)methanol (43)

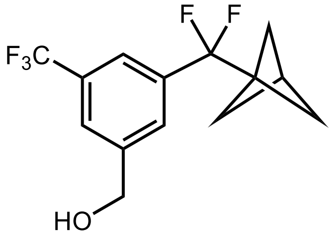

Following the **Procedure A** using **PCN (1)** and Cs_2CO_3 as suitable reagents.
Purified by flash chromatography (PE:EA = 7:1) on silica gel to afford the title compound (90.8 mg, 52% yield) as a white solid.

^{1}H NMR (400 MHz, $CDCl_3$) δ 7.69 (s, 1H), 7.56 (s, 2H), 4.78 (s, 2H), 2.55 (s, 1H), 2.31 (s, 1H), 1.83 (s, 6H).

^{13}C NMR (100 MHz, $CDCl_3$) δ 142.35, 136.86 (t, *J* = 27.8 Hz), 131.22 (q, *J* = 32.8 Hz), 126.74 (t, *J* = 6.2 Hz), 124.66 (dd, *J* = 3.8, 2.0 Hz), 123.84 (q, *J* = 272.6 Hz), 121.45 (td, *J* = 6.3, 3.6 Hz), 118.01 (t, *J* = 243.0 Hz), 64.20, 48.12 (t, *J* = 3.4 Hz), 45.92 (t, *J* = 35.7 Hz), 27.77 (t, *J* = 2.1 Hz).

^{19}F NMR (376 MHz, $CDCl_3$) δ -62.72 (s, 3F), -103.71 (s, 2F).

HRMS (APCI) m/z calculated for $C_{14}H_{12}F_5^+$ ($[M-H_2O+H]^+$): 275.0854, found 275.0856.

3-(Bicyclo[1.1.1]pentan-1-yldifluoromethyl)-5-(trifluoromethyl)aniline (44)

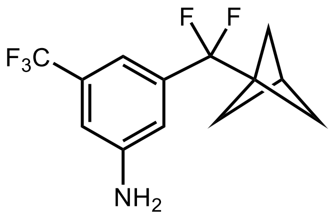

Following the **Procedure A** using **PCN (1)** and Cs_2CO_3 as suitable reagents.
Purified by flash chromatography (PE:EA = 15:1) on silica gel to afford the title compound (101.9 mg, 61% yield) as a pale yellow oil.

^{1}H NMR (500 MHz, CD_2Cl_2) δ 6.96 (d, *J* = 17.7 Hz, 2H), 6.84 (s, 1H), 4.06 (s, 2H), 2.53 (s, 1H), 1.83 (s, 6H).

^{13}C NMR (125 MHz, CD_2Cl_2) δ 147.75, 137.84 (t, *J* = 27.5 Hz), 131.97 (q, *J* = 32.0 Hz), 124.39 (q, *J* = 272.4 Hz), 118.51 (t, *J* = 242.6 Hz), 114.84 (t, *J* = 6.3 Hz), 112.49, 112.25 – 111.54 (m), 48.42, 46.17, 27.86.

^{19}F NMR (471 MHz, CD_2Cl_2) δ -63.35 (s, 3F), -104.26 (s, 2F).

HRMS (ESI) m/z calculated for $C_{13}H_{13}F_5N^+$ ($[M+H]^+$): 278.0963, found 278.0959.

2-(3-(bicyclo[1.1.1]pentan-1-yldifluoromethyl)-5-(trifluoromethyl)phenyl)-4,4,5,5-tetramethyl-1,3,2-dioxaborolane (45)

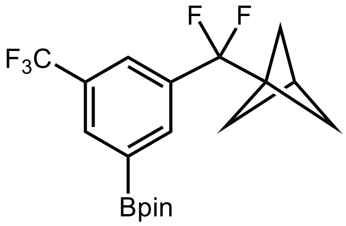

Following the **Procedure A** using **PCN (1)** and Cs_2CO_3 as suitable reagents.
Purified by flash chromatography (PE:EA = 10:1) on silica gel to afford the title compound (112.0 mg, 48% yield) as a pale yellow oil.

^{1}H NMR (400 MHz, CDCl$_3$) δ 8.11 (s, 1H), 8.00 (s, 1H), 7.71 (s, 1H), 2.54 (s, 1H), 1.84 (s, 6H), 1.36 (s, 12H).

^{13}C NMR (100 MHz, CDCl$_3$) δ 135.89 (t, *J* = 27.6 Hz), 134.68 (t, *J* = 5.8 Hz), 132.71 (q, *J* = 3.7 Hz), 130.39 (q, *J* = 32.6 Hz), 124.87 (tq, *J* = 6.2, 3.5 Hz), 124.06 (q, *J* = 272.5 Hz), 118.12 (t, *J* = 243.0 Hz), 84.70, 48.22 (t, *J* = 3.4 Hz), 46.09 (t, *J* = 35.7 Hz), 27.79 (t, *J* = 2.1 Hz), 24.99.

^{19}F NMR (376 MHz, CDCl$_3$) δ -62.66 (s, 3F), -103.69 (s, 2F).

MS (EI) m/z calculated for $C_{19}H_{22}BF_5O_2$+ ($[M]^+$): 388.1628, found 388.12.

1-((3-(2-chloroethoxy)-5 (trifluoromethyl)phenyl)difluoromethyl)bicyclo[1.1.1]pentane (46)

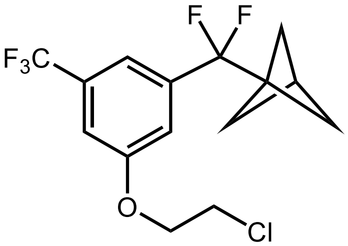

Following the **Procedure A** using **PCN (1)** and Cs_2CO_3 as suitable reagents.

Purified by flash chromatography (PE = 100%) on silica gel to afford the title compound (105.1 mg, 51% yield) as a pale yellow oil.

^{1}H NMR (400 MHz, CDCl$_3$) δ 7.27 (s, 1H), 7.20 (s, 1H), 7.11 (s, 1H), 4.29 (t, *J* = 5.7 Hz, 2H), 3.85 (t, *J* = 5.7 Hz, 2H), 2.55 (s, 1H), 1.84 (s, 6H).

^{13}C NMR (100 MHz, CDCl$_3$) δ 158.53, 138.30 (t, *J* = 27.8 Hz), 132.38 (q, *J* = 33.0 Hz), 123.61 (d, *J* = 272.5 Hz), 117.79 (d, *J* = 242.7 Hz), 115.77 – 114.45 (m), 112.80 (q, *J* = 3.8 Hz), 68.66, 48.21 (t, *J* = 3.4 Hz), 45.92 (t, *J* = 35.7 Hz), 41.70, 27.71 (t, *J* = 2.1 Hz).

^{19}F NMR (376 MHz, CDCl$_3$) δ -62.84 (s, 3F), -103.85 (s, 2F).

HRMS (EI) m/z calculated for $C_{15}H_{14}ClF_5O^+$ ([M]$^+$): 340.0648, found 340.0631.

1-(3-(Bicyclo[1.1.1]pentan-1-yldifluoromethyl)-5-(trifluoromethyl)phenyl)-2,5-dimethyl-1H-pyrrole (47)

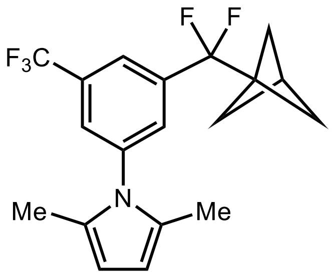

Following the **Procedure A** using **PBN (3)** and CsOH·H$_2$O as suitable reagents.
Purified by flash chromatography (PE = 100%) on silica gel to afford the title compound (68.2 mg, 32% yield) as a pale yellow oil.

^{1}H NMR (400 MHz, CDCl$_3$) δ 7.69 (s, 1H), 7.57 (s, 1H), 7.47 (s, 1H), 5.95 (s, 2H), 2.59 (s, 1H), 2.05 (s, 6H), 1.86 (s, 6H).

^{13}C NMR (100 MHz, CDCl$_3$) δ 139.98, 138.03 (t, *J* = 28.3 Hz), 132.14 (q, *J* = 33.3 Hz), 128.76, 128.74 (t, *J* = 6.7 Hz), 126.35 (q, *J* = 3.6 Hz), 123.37 (q, *J* = 272.9 Hz), 121.37 (tt,

J = 7.1, 3.7 Hz), 117.59 (t, *J* = 243.8 Hz), 107.08, 48.18 (t, *J* = 3.4 Hz), 45.94 (t, *J* = 35.5 Hz), 27.91 (t, *J* = 1.8 Hz), 13.16.

^{19}F NMR (376 MHz, $CDCl_3$) δ -62.72 (s, 3F), -104.18 (s, 2F).

HRMS (EI) m/z calculated for $C_{19}H_{18}F_5N^+$ ($[M]^+$): 355.1354, found 355.1351.

1-((3-(but-3-en-1-yloxy)-5-(trifluoromethyl)phenyl)difluoromethyl)bicyclo[1.1.1]pentane (48)

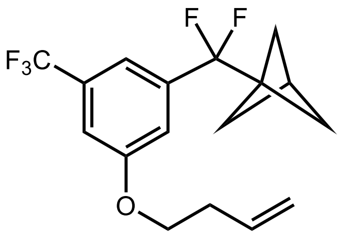

Following the **Procedure A** using **PBN (3)** and $CsOH·H_2O$ as suitable reagents.
Purified by flash chromatography (PE = 100%) on silica gel to afford the title compound (118.4 mg, 59% yield) as a pale yellow oil.

^{1}H NMR (400 MHz, $CDCl_3$) δ 7.23 (s, 1H), 7.18 (s, 1H), 7.09 (s, 1H), 5.91 (ddt, *J* = 17.0, 10.2, 6.7 Hz, 1H), 5.20 (dd, *J* = 17.2, 1.6 Hz, 1H), 5.14 (dd, *J* = 10.1, 1.3 Hz, 1H), 4.07 (t, *J* = 6.6 Hz, 2H), 2.58 (qd, *J* = 6.6, 1.3 Hz, 2H), 2.55 (s, 1H), 1.84 (s, 6H).

^{13}C NMR (100 MHz, $CDCl_3$) δ 159.23, 138.05 (t, *J* = 27.8 Hz), 134.01, 132.18 (q, *J* = 32.7 Hz), 123.77 (q, *J* = 272.5 Hz), 117.90 (t, *J* = 243.3 Hz), 117.66, 115.14 (t, *J* = 6.1 Hz), 114.48 (tq, *J* = 6.2, 2.9 Hz), 112.63 (q, *J* = 3.8 Hz), 67.94, 48.21 (t, *J* = 3.5 Hz), 45.99 (t, *J* = 35.7 Hz), 33.57, 27.67 (t, *J* = 2.0 Hz).

^{19}F NMR (376 MHz, $CDCl_3$) δ -62.85 (s, 3F), -103.90 (s, 2F).

MS (EI) m/z calculated for $C_{17}H_{17}F_5O^+$ ($[M]^+$): 332.1194, found 332.08.

1-((3-(but-3-yn-1-yloxy)-5-(trifluoromethyl)phenyl)difluoromethyl)bicyclo[1.1.1]pentane (49)

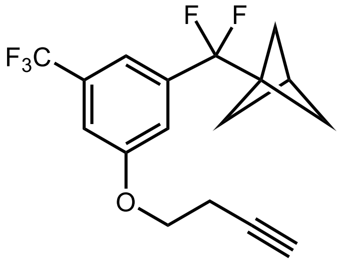

Following the **Procedure A** using **PBN (3)** and CsOH·H_2O as suitable reagents.
Purified by flash chromatography (PE = 100%) on silica gel to afford the title compound (94.2 mg, 48% yield) as a pale yellow oil.

^{1}H NMR (400 MHz, $CDCl_3$) δ 7.24 (s, 1H), 7.19 (s, 1H), 7.10 (s, 1H), 4.15 (t, *J* = 6.8 Hz, 2H), 2.72 (td, *J* = 6.8, 2.7 Hz, 2H), 2.55 (s, 1H), 2.07 (t, *J* = 2.7 Hz, 1H), 1.84 (s, 6H).

^{13}C NMR (100 MHz, $CDCl_3$) δ 158.77, 138.17 (t, *J* = 27.9 Hz), 132.28 (q, *J* = 32.8 Hz), 123.67 (q, *J* = 272.6 Hz), 117.83 (t, *J* = 243.3 Hz), 115.21 (t, *J* = 6.0 Hz), 114.92 (tq, *J* = 6.2, 3.8 Hz), 112.75 (q, *J* = 3.9 Hz), 79.99, 70.42, 66.66, 48.21 (t, *J* = 3.4 Hz), 45.95 (t, *J* = 35.6 Hz), 27.69 (t, *J* = 2.2 Hz), 19.61.

^{19}F NMR (376 MHz, $CDCl_3$) δ -62.85 (s, 3F), -103.87 (s, 2F).

HRMS (EI) m/z calculated for $C_{17}H_{15}F_5O^+$ ($[M]^+$): 330.1038, found 330.1018.

1-Benzhydryl-3-(3-(bicyclo[1.1.1]pentan-1-yldifluoromethyl)-5-(trifluoromethyl)phenoxy)azetidine (50)

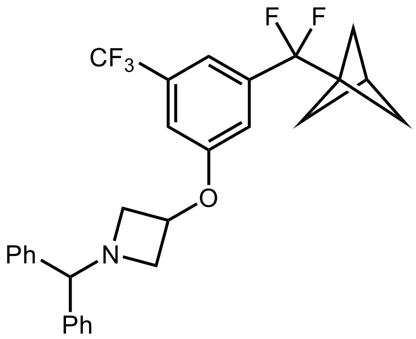

Following the **Procedure A** using **PBN (3)** and CsOH·H_2O as suitable reagents.
Purified by flash chromatography (PE:EA = 7:1) on silica gel to afford the title compound (93.5 mg, 32% yield) as a pale yellow oil.

^{1}H NMR (400 MHz, $CDCl_3$) δ 7.39 (d, *J* = 7.2 Hz, 4H), 7.25 (t, *J* = 7.5 Hz, 4H), 7.19 – 7.13 (m, 3H), 6.95 (d, *J* = 18.7 Hz, 2H), 4.80 (p, *J* = 5.6 Hz, 1H), 4.40 (s, 1H), 3.69 (t, *J* = 7.2 Hz, 2H), 3.11 (dd, *J* = 8.5, 6.5 Hz, 2H), 2.48 (s, 1H), 1.76 (s, 5H).

^{13}C NMR (100 MHz, $CDCl_3$) δ 157.46, 141.81, 138.27 (t, *J* = 27.9 Hz), 132.34 (q, *J* = 32.9 Hz), 128.73, 127.54, 123.59 (d, *J* = 272.3 Hz), 118.94 (d, *J* = 243.4 Hz), 115.34 (t, *J* = 6.0 Hz), 114.96 (dt, *J* = 9.8, 5.2 Hz), 112.59 (t, *J* = 3.6 Hz), 78.46, 66.83, 60.30, 48.17 (t, *J* = 3.4 Hz), 45.89 (t, *J* = 35.6 Hz), 27.68 (t, *J* = 1.7 Hz).

^{19}F NMR (376 MHz, $CDCl_3$) δ -62.83 (s, 3F), -103.87 (s, 2F).

HRMS (ESI) m/z calculated for $C_{29}H_{27}F_5NO^+$ ($[M+H]^+$): 500.2007, found 500.1996.

4-(3-(Bicyclo[1.1.1]pentan-1-yldifluoromethyl)-5-(trifluoromethyl)phenoxy)-1,2,2,6,6-pentamethylpiperidine (51)

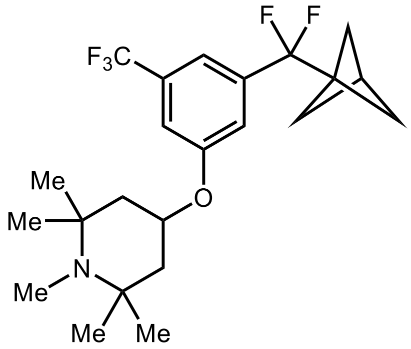

Following the **Procedure A** using **PBN (3)** and CsOH·H_2O as suitable reagents.
Purified by flash chromatography (PE:EA = 5:1) on silica gel to afford the title compound (158.4 mg, 61% yield) as a white solid.

^{1}H NMR (400 MHz, CDCl$_3$) δ 7.21 (s, 1H), 7.16 (s, 1H), 7.07 (s, 1H), 4.58 (tt, *J* = 11.2, 3.9 Hz, 1H), 2.55 (s, 1H), 2.29 (s, 3H), 2.00 (dd, *J* = 12.4, 3.8 Hz, 2H), 1.84 (s, 6H), 1.62 (t, *J* = 11.3 Hz, 2H), 1.21 (s, 6H), 1.14 (s, 6H).

^{13}C NMR (100 MHz, CDCl$_3$) δ 158.02, 138.05 (t, *J* = 27.7 Hz), 132.24 (q, *J* = 32.7 Hz), 123.75 (q, *J* = 272.6 Hz), 117.85 (t, *J* = 243.2 Hz), 115.86 (t, *J* = 5.7 Hz), 114.18 (tq, *J* = 7.8, 4.0 Hz), 113.85 (q, *J* = 3.6 Hz), 70.89, 55.51, 48.20 (t, *J* = 3.4 Hz), 46.02, 33.07, 28.19, 27.70, 21.20.

^{19}F NMR (376 MHz, CDCl$_3$) δ -62.91 (s, 3F), -104.03 (s, 2F).

HRMS (ESI) m/z calculated for $C_{23}H_{31}F_5NO^+$ ($[M+H]^+$): 432.2320, found 432.2307.

(3aR,5aS,8aS,8bS)-5-((3-(Bicyclo[1.1.1]pentan-1-yldifluoromethyl)-5-(trifluoromethyl)phenoxy)methyl)-2,2,7,7-tetramethyltetrahydro-5*H*-bis([1,3]dioxolo)[4,5-b:4',5'-d]pyran (52)

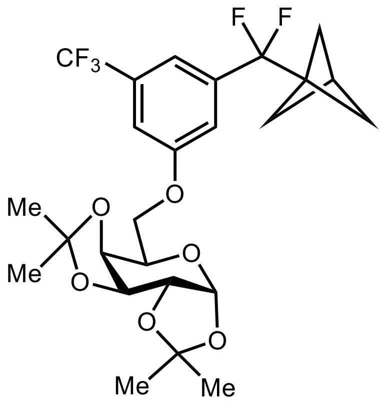

Following the **Procedure A** using **PBN (3)** and CsOH·H$_2$O as suitable reagents.
Purified by flash chromatography (PE:EA = 6:1) on silica gel to afford the title compound (178.6 mg, 57% yield) as a white solid.

^{1}H NMR (400 MHz, CDCl$_3$) δ 7.24 – 7.20 (m, 2H), 7.14 (s, 1H), 5.57 (d, *J* = 5.0 Hz, 1H), 4.66 (dd, *J* = 7.9, 2.5 Hz, 1H), 4.36 (t, *J* = 2.5 Hz, 1H), 4.35 (d, *J* = 3.3 Hz, 1H), 4.24 – 4.14 (m, 3H), 2.53 (s, 1H), 1.82 (s, 6H), 1.53 (s, 3H), 1.48 (s, 3H), 1.36 (s, 3H), 1.34 (s, 3H).

^{13}C NMR (100 MHz, CDCl$_3$) δ 158.98, 138.02 (t, *J* = 27.8 Hz), 132.11 (q, *J* = 32.8 Hz), 123.71 (q, *J* = 272.6 Hz), 117.85 (t, *J* = 243.4 Hz), 115.41 (t, *J* = 6.2 Hz), 112.90 (q, *J* = 3.2 Hz), 109.78, 108.98, 96.50, 71.06, 70.80, 70.68, 67.46, 66.37, 48.19 (t, *J* = 3.6 Hz), 45.94 (t, *J* = 35.7 Hz), 27.64, 26.16, 26.10, 25.01, 24.53.

^{19}F NMR (376 MHz, CDCl$_3$) δ -62.83 (s, 3F), -103.83 (d, *J* = 23.1 Hz, 2F).

HRMS (EI) m/z calculated for $C_{25}H_{29}F_5O_6^+$ ($[M]^+$): 520.1879, found 520.1877.

1-(Difluoro(4-(4-fluoro-3-methylphenoxy)phenyl)methyl)bicyclo[1.1.1]pentane (53)

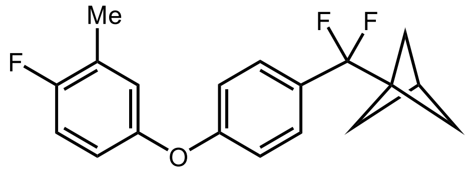

Following the **Procedure A** using **PBN (3)** and CsOH·H_2O as suitable reagents.
Purified by flash chromatography (PE = 100%) on silica gel to afford the title compound (84.0 mg, 44% yield) as a pale yellow oil.

^{1}H NMR (400 MHz, CDCl$_3$) δ 7.34 (d, *J* = 8.7 Hz, 2H), 7.00 (t, *J* = 9.0 Hz, 1H), 6.96 (d, *J* = 8.5 Hz, 2H), 6.89 (dd, *J* = 6.4, 3.0 Hz, 1H), 6.83 (dt, *J* = 8.7, 3.6 Hz, 1H), 2.54 (s, 1H), 2.27 (d, *J* = 2.1 Hz, 3H), 1.84 (s, 6H).

^{13}C NMR (100 MHz, CDCl$_3$) δ 159.17 (d, *J* = 4.8 Hz), 156.75, 151.72 (d, *J* = 2.6 Hz), 129.90 (t, *J* = 27.5 Hz), 127.00 (t, *J* = 6.1 Hz), 126.63 (d, *J* = 19.2 Hz), 122.75 (d, *J* = 5.2 Hz), 118.67 (t, *J* = 242.2 Hz), 118.53 (d, *J* = 8.3 Hz), 117.31, 116.07 (d, *J* = 24.3 Hz), 48.21 (t, *J* = 3.4 Hz), 46.39 (t, *J* = 36.8 Hz), 27.66 (t, *J* = 2.1 Hz), 14.84 (d, *J* = 3.3 Hz).

^{19}F NMR (376 MHz, CDCl$_3$) δ -102.60 (s, 2F), -123.13 – -123.39 (m, 1F).

HRMS (EI) m/z calculated for $C_{19}H_{17}F_3O^+$ ($[M]^+$): 318.1226, found 318.1225.

5-(Bicyclo[1.1.1]pentan-1-yldifluoromethyl)-*N*-(5-methoxypyridin-2-yl)pyridin-2-amine (54)

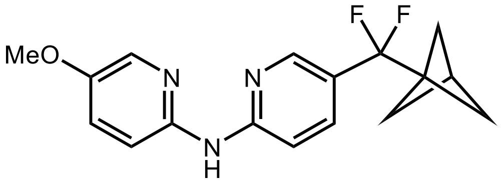

Following the **Procedure A** using **PCN (1)** and Cs_2CO_3 as suitable reagents.
Purified by flash chromatography (PE:EA:Et_3N = 2:1:0.2) on silica gel to afford the title compound (113.1 mg, 59% yield) as a white solid.

^{1}H NMR (400 MHz, $CDCl_3$) δ 8.31 (s, 1H), 7.92 (d, J = 6.4 Hz, 1H), 7.78 (s, 1H), 7.63 (d, J = 8.6 Hz, 1H), 7.45 (d, J = 8.6 Hz, 1H), 6.56 (d, J = 6.4 Hz, 1H), 3.96 (s, 3H), 2.56 (s, 1H), 1.84 (s, 6H).

^{13}C NMR (100 MHz, $CDCl_3$) δ 169.49, 154.62, 153.76, 145.25 – 142.52 (m), 135.71, 125.16 (t, J = 28.6 Hz), 117.92 (t, J = 242.7 Hz), 112.86, 106.66, 96.82, 56.40, 48.11 (t, J = 3.3 Hz), 46.17 (t, J = 36.3 Hz), 27.88.

^{19}F NMR (376 MHz, $CDCl_3$) δ -104.34 (s, 2F).

HRMS (ESI) m/z calculated for $C_{17}H_{18}F_2N_3O$+ ($[M+H]^+$): 318.1412, found 318.1406.

***tert*-Butyl 4-(4-(bicyclo[1.1.1]pentan-1-yldifluoromethyl)benzyl)piperazine-1-carboxylate (55)**

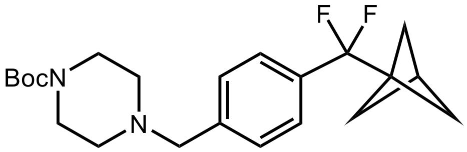

Following the **Procedure A** using **PCN (1)** and Cs_2CO_3 as suitable reagents.
Purified by flash chromatography (PE:EA:Et_3N = 3:1:0.2) on silica gel to afford the title compound (98.7 mg, 42% yield) as a white solid.

^{1}H NMR (400 MHz, $CDCl_3$) δ 7.40 – 7.30 (m, 4H), 3.54 (s, 2H), 3.44 (s, 4H), 2.51 (s, 1H), 2.40 (s, 4H), 1.81 (s, 6H), 1.44 (s, 9H).

^{13}C NMR (100 MHz, $CDCl_3$) δ 154.83, 139.01, 134.54 (t, *J* = 27.0 Hz), 129.01, 125.33 (t, *J* = 6.1 Hz), 118.65 (t, *J* = 242.1 Hz), 79.77, 62.60, 52.93, 48.15 (t, *J* = 3.3 Hz), 46.26 (t, *J* = 36.4 Hz), 44.59 – 42.69 (m), 28.51, 27.61.

^{19}F NMR (376 MHz, $CDCl_3$) δ -103.35 (s, 2F).

HRMS (ESI) m/z calculated for $C_{22}H_{31}F_2N_2O_2^+$ ($[M+H]^+$): 393.2348, found 393.2342.

(±)-3-(4-(Bicyclo[1.1.1]pentan-1-yldifluoromethyl)phenoxy)-*N*-methyl-3-phenylpropan-1-amine (56)

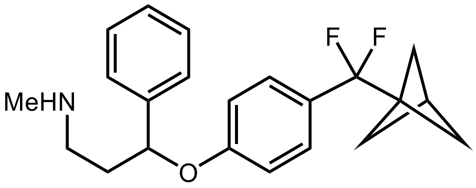

Following the **Procedure A** using **PBN (3)** and $CsOH{\cdot}H_2O$ as suitable reagents.
Purified by flash chromatography (PE:EA:Et_3N = 3:1:0.3) on silica gel to afford the title compound (69.0 mg, 32% yield) as a pale yellow solid.

^{1}H NMR (400 MHz, $CDCl_3$) δ 7.34 – 7.29 (m, 5H), 7.26 (s, 1H), 7.20 (d, *J* = 8.5 Hz, 2H), 6.83 (d, *J* = 8.7 Hz, 2H), 5.31 (dd, *J* = 8.1, 4.2 Hz, 1H), 3.33 – 3.24 (m, 1H), 3.00 – 2.92 (m, 2H), 2.70 (d, *J* = 6.4 Hz, 1H), 2.53 (s, 3H), 2.49 (s, 1H), 2.25 (s, 1H), 1.78 (s, 6H).

^{13}C NMR (100 MHz, $CDCl_3$) δ 128.99, 128.89, 128.14, 127.93, 126.70, 126.65, 125.96, 125.87, 118.66 (t, *J* = 272.1 Hz), 115.57, 115.51, 53.52, 48.21, 47.29, 36.65, 29.85, 27.59, 22.83.

^{19}F NMR (376 MHz, $CDCl_3$) δ -102.28 (s, 2F).

HRMS (ESI) m/z calculated for $C_{22}H_{26}F_2NO^+$ ($[M+H]^+$): 358.1977, found 358.1972.

(R)-3-(3-(Bicyclo[1.1.1]pentan-1-yldifluoromethyl)phenyl)-*N*-(1-(naphthalen-1-yl)ethyl)propan-1-amine (57)

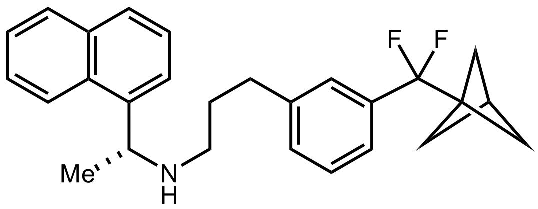

Following the **Procedure A** using **PBN (3)** and CsOH·H_2O as suitable reagents.
Purified by flash chromatography (PE:EA:Et_3N = 10:1:1.7) on silica gel to afford the title compound (136.8 mg, 56% yield) as a pale yellow oil.

^{1}H NMR (400 MHz, $CDCl_3$) δ 8.15 (d, *J* = 8.2 Hz, 1H), 7.90 – 7.85 (m, 1H), 7.76 (d, *J* = 8.2 Hz, 1H), 7.70 (d, *J* = 7.0 Hz, 1H), 7.55 – 7.45 (m, 3H), 7.24 (s, 1H), 7.22 – 7.13 (m, 3H), 4.70 (q, *J* = 6.3 Hz, 1H), 2.74 – 2.56 (m, 4H), 2.47 (s, 1H), 1.88 (p, *J* = 7.4 Hz, 2H), 1.76 (s, 6H), 1.55 (d, *J* = 6.5 Hz, 3H).

^{13}C NMR (100 MHz, $CDCl_3$) δ 142.06, 136.03 – 135.14 (m), 134.12, 131.37, 129.61, 129.19, 128.29, 127.61, 126.11, 125.89, 125.57, 125.18 (t, *J* = 6.0 Hz), 123.05, 122.94, 122.86 (t, *J* = 3.6 Hz), 118.72, 53.80, 48.19 (t, *J* = 3.4 Hz), 47.26 (t, *J* = 95.6 Hz), 33.63, 32.07, 29.85, 27.58, 23.43.

^{19}F NMR (376 MHz, $CDCl_3$) δ -103.74 (s, 2F).

HRMS (ESI) m/z calculated for $C_{27}H_{30}F_2N^+$ ($[M+H]^+$): 406.2341, found 406.2332.

3-(2-(Bicyclo[1.1.1]pentan-1-yldifluoromethyl)-10*H*-phenothiazin-10-yl)-*N*,*N*-dimethylpropan-1-amine (58)

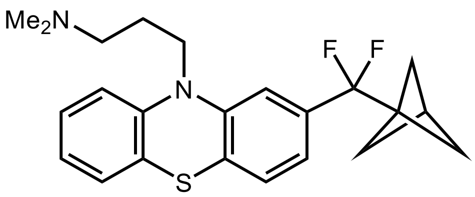

Following the **Procedure A** using **PBN (3)** and $CsOH\cdot H_2O$ as suitable reagents.
Purified by flash chromatography (PE:EA:Et_3N = 10:1:1.7) on silica gel to afford the title compound (175.9 mg, 73% yield) as a pale yellow solid.

^{1}H NMR (400 MHz, $CDCl_3$) δ 7.19 – 7.11 (m, 3H), 6.97 – 6.89 (m, 3H), 6.85 (d, *J* = 1.2 Hz, 1H), 3.93 (t, *J* = 6.9 Hz, 2H), 2.52 (s, 1H), 2.47 (t, *J* = 7.1 Hz, 2H), 2.24 (s, 6H), 1.97 (p, *J* = 7.1 Hz, 2H), 1.82 (s, 6H).

^{13}C NMR (100 MHz, $CDCl_3$) δ 145.21, 145.01, 134.82 (t, *J* = 27.2 Hz), 127.66, 127.64, 127.26, 127.16, 124.78, 122.96, 119.57 (t, *J* = 6.1 Hz), 118.58 (d, *J* = 242.7 Hz), 115.94, 112.34 (t, *J* = 6.3 Hz), 57.10, 48.24 (t, *J* = 3.5 Hz), 46.27 (t, *J* = 36.5 Hz), 45.57, 45.43, 29.84, 27.59, 24.91.

^{19}F NMR (376 MHz, $CDCl_3$) δ -103.49 (s, 2F).

HRMS (ESI) m/z calculated for $C_{23}H_{27}F_2N_2S^+$ ($[M+H]^+$): 401.1858, found 401.1847.

***N*-(2-(Bicyclo[3.1.1]heptan-1-yldifluoromethyl)phenyl)acetamide (59)**

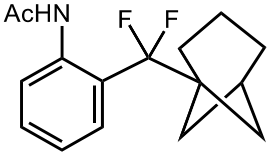

Following the **Procedure B** using **PCN (1)** and Cs_2CO_3 as suitable reagents.
Purified by flash chromatography (PE:EA = 3:1) on silica gel to afford the title compound (109.4 mg, 65% yield) as a pale yellow solid.

^{1}H NMR (400 MHz, $CDCl_3$) δ 8.00 (d, *J* = 8.2 Hz, 1H), 7.85 (s, 1H), 7.39 (t, *J* = 7.7 Hz, 1H), 7.31 (d, *J* = 7.8 Hz, 1H), 7.14 (t, *J* = 7.6 Hz, 1H), 2.24 (d, *J* = 6.5 Hz, 1H), 2.15 (s, 3H), 2.13 – 2.08 (m, 2H), 1.80 – 1.64 (m, 6H), 1.43 – 1.34 (m, 2H).

^{13}C NMR (100 MHz, $CDCl_3$) δ 168.19, 134.86, 130.42, 127.60 (t, *J* = 8.4 Hz), 124.90 (t, *J* = 24.7 Hz), 124.87 (t, *J* = 244.1 Hz), 124.46, 124.21, 48.13 (t, *J* = 28.8 Hz), 32.32 (t, *J* = 4.1 Hz), 29.32, 28.17 (t, *J* = 3.2 Hz), 28.00, 24.92, 15.93.

^{19}F NMR (376 MHz, $CDCl_3$) δ -101.44 (d, *J* = 6.4 Hz, 2F).

HRMS (ESI) m/z calculated for $C_{16}H_{20}F_2NO^+$ ($[M+H]^+$): 280.1507, found 280.1501.

1-(Difluoro(2-fluoro-5-methoxyphenyl)methyl)bicyclo[3.1.1]heptane (60)

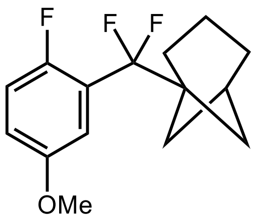

Following the **Procedure B** using **PBN (3)** and $CsOH{\cdot}H_2O$ as suitable reagents.
Purified by flash chromatography (PE = 100%) on silica gel to afford the title compound (112.3 mg, 75% yield) as a colorless oil.

^{1}H NMR (400 MHz, $CDCl_3$) δ 7.04 – 6.94 (m, 1H), 6.87 (dd, *J* = 6.0, 3.1 Hz, 2H), 3.78 (s, 3H), 2.27 (s, 3H), 1.83 – 1.71 (m, 4H), 1.66 (t, *J* = 6.3 Hz, 2H), 1.40 (dd, *J* = 6.6, 2.5 Hz, 2H).

^{13}C NMR (100 MHz, $CDCl_3$) δ 155.25 (d, *J* = 1.9 Hz), 153.71 (dt, *J* = 244.6, 3.7 Hz), 123.39 (td, *J* = 28.3, 14.5 Hz), 122.03 (td, *J* = 244.6, 2.9 Hz), 117.23 (d, *J* = 24.5 Hz), 116.30 (d, *J* = 8.1 Hz), 113.31 (td, *J* = 8.3, 3.2 Hz), 55.93, 48.05 (t, *J* = 28.6 Hz), 32.09 (td, *J* = 3.9, 2.6 Hz), 28.95 (d, *J* = 2.1 Hz), 28.54 (t, *J* = 3.4 Hz), 28.32, 16.06.

^{19}F NMR (376 MHz, CDCl$_3$) δ -105.28 (d, *J* = 16.5 Hz, 2F), -123.13 (dh, *J* = 15.9, 5.0 Hz, 1F).

HRMS (EI) m/z calculated for $C_{15}H_{17}F_3O^+$ ($[M]^+$): 270.1226, found 270.1226.

1-(Difluoro(3-methoxy-5-(trifluoromethyl)phenyl)methyl)bicyclo[3.1.1]heptane (61)

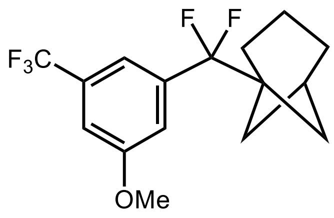

Following the **Procedure B** using **PBN (3)** and CsOH·H_2O as suitable reagents.
Purified by flash chromatography (PE = 100%) on silica gel to afford the title compound (108.4 mg, 56% yield) as a pale yellow oil.

^{1}H NMR (400 MHz, CDCl$_3$) δ 7.20 (s, 1H), 7.16 (s, 1H), 7.06 (s, 1H), 3.86 (s, 3H), 2.36 – 2.24 (m, 3H), 1.79 – 1.71 (m, 5H), 1.60 (d, *J* = 6.4 Hz, 2H), 1.43 – 1.35 (m, 2H).

^{13}C NMR (100 MHz, CDCl$_3$) δ 159.75, 138.28 (t, *J* = 28.6 Hz), 131.98 (q, *J* = 32.7 Hz), 123.81 (q, *J* = 272.5 Hz), 121.93 (t, *J* = 244.2 Hz), 115.36 (t, *J* = 6.1 Hz), 114.98 (tq, *J* = 7.9, 4.0 Hz), 111.63 (q, *J* = 3.6 Hz), 55.79, 47.71 (t, *J* = 28.9 Hz), 31.78 (t, *J* = 3.9 Hz), 28.95, 28.58 (t, *J* = 3.6 Hz), 28.38, 15.98.

^{19}F NMR (376 MHz, CDCl$_3$) δ -62.80 (s, 3F), -107.20 (s, 2F).

HRMS (EI) m/z calculated for $C_{16}H_{17}F_5O^+$ ($[M]^+$): 320.1194, found 320.1196.

3-(Bicyclo[3.1.1]heptan-1-yldifluoromethyl)-5-(trifluoromethyl)aniline (62)

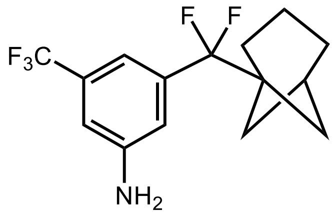

Following the **Procedure B** using **PCN (1)** and Cs_2CO_3 as suitable reagents.
Purified by flash chromatography (PE:EA = 15:1) on silica gel to afford the title compound (147.1 mg, 80% yield) as a pale yellow oil.

^{1}H NMR (400 MHz, $CDCl_3$) δ 6.97 (s, 1H), 6.90 (s, 1H), 6.80 (s, 1H), 3.94 (s, 2H), 2.35 – 2.21 (m, 3H), 1.74 (s, 4H), 1.61 (d, *J* = 5.1 Hz, 2H), 1.41 – 1.35 (m, 2H).

^{13}C NMR (100 MHz, $CDCl_3$) δ 146.83, 137.84 (t, *J* = 28.2 Hz), 131.72 (q, *J* = 32.3 Hz), 124.00 (q, *J* = 272.5 Hz), 122.16 (t, *J* = 243.9 Hz), 115.39 – 114.84 (m), 112.46 (tq, *J* = 8.0, 4.0 Hz), 112.21 (t, *J* = 3.8 Hz), 47.67 (t, *J* = 29.0 Hz), 31.77 (t, *J* = 4.0 Hz), 28.92, 28.56 (t, *J* = 3.6 Hz), 28.36, 15.95.

^{19}F NMR (376 MHz, $CDCl_3$) δ -62.97 (s, 3F), -107.23 (s, 2F).

HRMS (ESI) m/z calculated for $C_{15}H_{17}F_5N^+$ ($[M+H]^+$): 306.1276, found 306.1270.

3-(2-(Bicyclo[3.1.1]heptan-1-yldifluoromethyl)-10*H*-phenothiazin-10-yl)-*N*,*N*-dimethylpropan-1-amine (63)

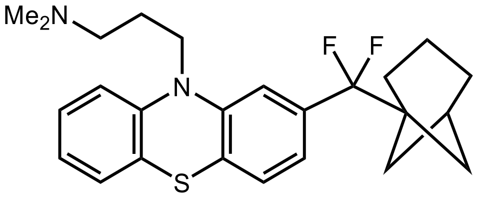

Following the **Procedure B** using **PBN (3)** and $CsOH{\cdot}H_2O$ as suitable reagents.
Purified by flash chromatography (PE:EA:Et_3N = 10:1:1.7) on silica gel to afford the title compound (175.9 mg, 73% yield) as a pale yellow solid.

^{1}H NMR (400 MHz, $CDCl_3$) δ 7.18 – 7.08 (m, 3H), 6.95 – 6.88 (m, 3H), 6.82 (s, 1H), 3.91 (t, *J* = 6.9 Hz, 2H), 2.41 (t, *J* = 7.0 Hz, 2H), 2.21 (s, 6H), 1.97 (dd, *J* = 30.9, 7.1 Hz, 4H), 1.72 (s, 3H), 1.68 – 1.61 (m, 2H), 1.56 – 1.35 (m, 2H), 1.20 (s, 2H).

^{13}C NMR (100 MHz, CDCl$_3$) δ 145.01, 144.95, 135.84 – 134.69 (m), 127.63, 127.60, 127.01, 126.86, 124.74, 122.91, 120.45, 120.01 (t, *J* = 6.2 Hz), 115.88, 112.85 (t, *J* = 6.5 Hz), 57.09, 46.97, 45.39, 43.41 (t, *J* = 29.3 Hz), 42.55, 32.15, 31.13 (t, *J* = 3.6 Hz), 28.84 (d, *J* = 62.4 Hz), 28.80 (t, *J* = 8.3 Hz), 24.84, 16.79.

^{19}F NMR (376 MHz, CDCl$_3$) δ -106.68 (s, 2F).

HRMS (ESI) m/z calculated for $C_{25}H_{31}F_2N_2S^+$ ([M+H]$^+$): 429.2171, found 429.2160.

***N*-(2-(Bicyclo[4.1.1]octan-1-yldifluoromethyl)phenyl)acetamide (64)**

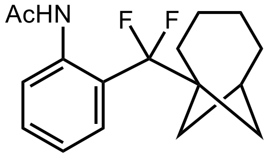

Following the **Procedure C** using **PCN (1)** and Cs_2CO_3 as suitable reagents.
Purified by flash chromatography (PE:EA = 3:1) on silica gel to afford the title compound (106.9 mg, 56% yield) as a pale yellow solid.

^{1}H NMR (400 MHz, CDCl$_3$) δ 8.06 (d, *J* = 8.3 Hz, 1H), 7.90 (s, 1H), 7.40 (t, *J* = 7.9 Hz, 1H), 7.31 (d, *J* = 8.6 Hz, 1H), 7.14 (t, *J* = 7.7 Hz, 1H), 2.52 (td, *J* = 9.1, 2.9 Hz, 2H), 2.40 (dq, *J* = 12.9, 5.4, 4.6 Hz, 1H), 2.17 (s, 3H), 1.69 (h, *J* = 3.0 Hz, 4H), 1.58 (ddt, *J* = 21.4, 9.8, 2.7 Hz, 4H), 1.48 (d, *J* = 5.1 Hz, 2H).

^{13}C NMR (100 MHz, CDCl$_3$) δ 168.25, 135.19, 130.48, 127.86 (t, *J* = 8.1 Hz), 126.04, 125.14 (t, *J* = 240.8 Hz), 124.49, 124.19, 48.16 (t, *J* = 27.0 Hz), 31.17, 30.36, 29.38 (d, *J* = 4.9 Hz), 28.37, 25.05, 24.97, 24.52.

HRMS (ESI) m/z calculated for $C_{17}H_{22}F_2NO^+$ ([M+H]$^+$): 294.1664, found 294.1661.

^{19}F NMR (376 MHz, CDCl$_3$) δ -100.28 (d, *J* = 7.6 Hz, 2F).

1-(Difluoro(2-fluoro-5-methoxyphenyl)methyl)bicyclo[4.1.1]octane (65)

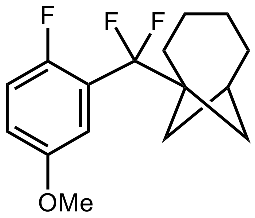

Following the **Procedure C** using **PBN (3)** and CsOH·H_2O as suitable reagents.
Purified by flash chromatography (PE = 100%) on silica gel to afford the title compound (90.6 mg, 53% yield) as a colorless oil.

^{1}H NMR (400 MHz, CDCl$_3$) δ 7.03 – 6.96 (m, 1H), 6.89 (td, *J* = 8.6, 3.3 Hz, 2H), 3.79 (s, 3H), 2.64 (t, *J* = 9.6 Hz, 2H), 2.48 – 2.34 (m, 1H), 1.76 – 1.63 (m, 4H), 1.63 – 1.54 (m, 4H), 1.44 – 1.36 (m, 2H).

^{13}C NMR (100 MHz, CDCl$_3$) δ 155.21 (d, *J* = 1.7 Hz), 153.65 (dt, *J* = 244.6, 3.7 Hz), 123.69 (td, *J* = 28.6, 14.5 Hz), 122.95 (td, *J* = 246.0, 3.0 Hz), 117.29 (d, *J* = 24.8 Hz), 116.25 (d, *J* = 8.2 Hz), 113.62 (td, *J* = 8.2, 3.3 Hz), 55.95, 47.82 (t, *J* = 26.7 Hz), 31.57 (t, *J* = 2.6 Hz), 30.62, 29.56 – 28.74 (m), 28.06 (d, *J* = 2.2 Hz), 25.05, 24.69.

^{19}F NMR (376 MHz, CDCl$_3$) δ -104.80 (d, *J* = 13.4 Hz, 2F), -122.31 (dh, *J* = 15.0, 4.9 Hz, 1F).

HRMS (EI) m/z calculated for $C_{16}H_{19}F_3NaO^+$ ([M]$^+$): 284.1383, found 284.1384.

2-(Bicyclo[4.1.1]octan-1-yl)difluoromethyl)pyridin-3-yl)methanol (66)

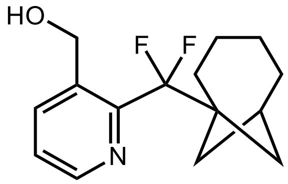

Following the **Procedure C** using **PCN (1)** and Cs_2CO_3 as suitable reagents.
Purified by flash chromatography (PE:EA = 3:1) on silica gel to afford the title compound (98.3 mg, 62% yield) as a pale yellow oil.

^{1}H NMR (400 MHz, CDCl$_3$) δ 8.43 (d, *J* = 4.3 Hz, 1H), 7.98 (d, *J* = 7.8 Hz, 1H), 7.28 (dd, *J* = 7.8, 4.7 Hz, 1H), 4.85 (s, 2H), 2.96 (s, 1H), 2.61 (td, *J* = 9.2, 2.7 Hz, 2H), 2.44 – 2.31 (m, 1H), 1.73 – 1.64 (m, 6H), 1.61 – 1.45 (m, 4H).

^{13}C NMR (100 MHz, CDCl$_3$) δ 150.70 (t, *J* = 31.1 Hz), 146.78, 136.63, 136.08, 124.32, 124.06 (t, *J* = 244.1 Hz), 60.97 (t, *J* = 8.6 Hz), 47.67 (t, *J* = 25.1 Hz), 31.26 (t, *J* = 2.8 Hz), 30.63, 29.72 (t, *J* = 5.1 Hz), 28.93, 25.04, 24.87.

^{19}F NMR (376 MHz, CDCl$_3$) δ -103.02 (s, 2F).

HRMS (ESI) m/z calculated for $C_{15}H_{20}F_2NO^+$ ($[M+H]^+$): 268.1507, found 268.1499.

5-(Bicyclo[4.1.1]octan-1-yl)difluoromethyl)-*N*-(5-methoxypyridin-2-yl)pyridin-2-amine (67)

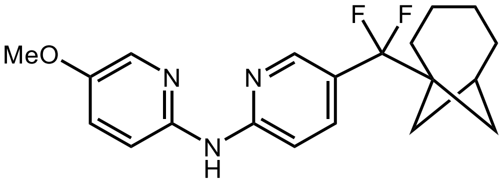

Following the **Procedure C** using **PCN (1)** and Cs_2CO_3 as suitable reagents.
Purified by flash chromatography (PE:EA:Et_3N = 2:1:0.2) on silica gel to afford the title compound (76.3 mg, 35% yield) as a white solid.

^{1}H NMR (400 MHz, CDCl$_3$) δ 9.36 (s, 1H), 8.28 (s, 1H), 8.03 (d, *J* = 6.0 Hz, 1H), 7.56 (dd, *J* = 8.7, 2.0 Hz, 1H), 7.49 – 7.40 (m, 2H), 6.47 (dd, *J* = 6.0, 2.1 Hz, 1H), 3.86 (s, 3H), 2.58 (td, *J* = 9.1, 2.2 Hz, 2H), 2.43 (d, *J* = 8.5 Hz, 1H), 1.65 (s, 4H), 1.55 (d, *J* = 10.2 Hz, 4H), 1.38 (s, 2H).

^{13}C NMR (100 MHz, CDCl$_3$) δ 169.91, 167.89, 155.18, 154.96, 146.76, 145.01 (t, *J* = 6.9 Hz), 124.31 (t, *J* = 28.9 Hz), 123.07 (t, *J* = 244.9 Hz), 111.36, 105.39, 96.72, 55.50, 47.51 (t, *J* = 27.3 Hz), 31.57, 30.52, 28.65 (t, *J* = 4.5 Hz), 28.10, 24.96, 24.61.

^{19}F NMR (376 MHz, CDCl$_3$) δ -106.11 (s, 2F).

HRMS (ESI) m/z calculated for $C_{20}H_{24}F_2N_3O^+$ ($[M+H]^+$): 360.1882, found 360.1870.

3-(2-(Bicyclo[4.1.1]octan-1-yl)difluoromethyl)-10*H*-phenothiazin-10-yl)-*N,N*-dimethylpropan-1-amine (68)

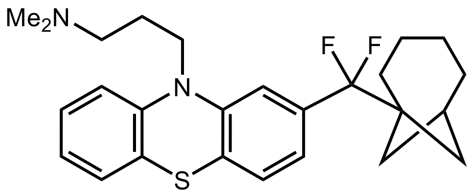

Following the **Procedure C** using **PBN (3)** and CsOH·H$_2$O as suitable reagents.
Purified by flash chromatography (PE:EA:Et$_3$N = 10:1:1.7) on silica gel to afford the title compound (55.5 mg, 21% yield) as a pale yellow solid.

^{1}H NMR (400 MHz, CDCl$_3$) δ 7.25 – 7.15 (m, 3H), 7.04 – 6.95 (m, 2H), 6.92 (d, *J* = 7.9 Hz, 1H), 6.85 (s, 1H), 4.04 (s, 2H), 3.13 (s, 2H), 2.68 (s, 6H), 2.64 – 2.56 (m, 2H), 2.49 – 2.39 (m, 1H), 2.25 (s, 4H), 1.65 (s, 4H), 1.31 (s, 2H).

^{13}C NMR (100 MHz, CDCl$_3$) δ 144.62, 144.15, 137.32, 137.22, 135.84, 128.05, 128.01, 127.46, 123.81 (t, *J* = 242.6 Hz), 120.84, 116.47, 55.82, 44.30, 43.07, 31.68, 30.51, 28.90, 28.16, 24.97, 24.61, 21.76, 13.50.Z

^{19}F NMR (376 MHz, CDCl$_3$) δ -75.70 (s, 2F).

HRMS (ESI) m/z calculated for $C_{26}H_{33}F_2N_2S^+$ ($[M+H]^+$): 443.2327, found 443.2318.

***N-(*2-(Difluoro(3-(4,4,5,5-tetramethyl-1,3,2-dioxaborolan-2-yl)bicyclo[1.1.1]pentan-1-yl)methyl)phenyl)acetamide (69)**

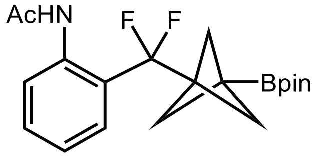

Following the **Procedure D**.

Purified by flash chromatography (PE:EA = 4:1) on silica gel to afford the title compound (286.8 mg, 63% yield) as a white solid.

^{1}H NMR (400 MHz, CDCl$_3$) δ 8.07 (d, *J* = 8.3 Hz, 1H), 7.83 – 7.72 (m, 1H), 7.40 (t, *J* = 7.3 Hz, 1H), 7.36 (d, *J* = 7.2 Hz, 1H), 7.15 (t, *J* = 7.1 Hz, 1H), 2.17 (s, 3H), 1.89 (s, 6H), 1.20 (s, 12H).

^{13}C NMR (100 MHz, CDCl$_3$) δ 168.31, 134.43, 130.67, 126.47 (t, *J* = 8.2 Hz), 124.89, 124.48, 124.13, 119.74 (d, *J* = 244.9 Hz), 83.81, 49.17 (t, *J* = 3.5 Hz), 48.85, 46.06, 45.23, 24.82.

^{19}F NMR (376 MHz, CDCl$_3$) δ -99.14 (d, *J* = 6.1 Hz, 2F).

^{11}B NMR (128 MHz, CDCl$_3$) δ 30.03 (s, 1B).

HRMS (ESI) m/z calculated for $C_{20}H_{29}BF_2NO_3^+$ ($[M+H]^+$): 378.2047, found 378.2033.

2-(3-(Difluoro(3-methoxy-5-(trifluoromethyl)phenyl)methyl)bicyclo[1.1.1]pentan-1-yl)-4,4,5,5-tetramethyl-1,3,2-dioxaborolane (70)

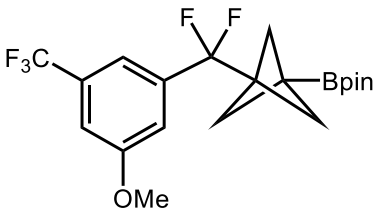

Following the **Procedure D**.

Purified by flash chromatography (PE = 100%) on silica gel to afford the title compound (275.0 mg, 55% yield) as a pale yellow oil.

^{1}H NMR (400 MHz, CDCl$_3$) δ 7.21 (s, 1H), 7.15 (s, 1H), 7.07 (s, 1H), 3.85 (s, 3H), 1.89 (s, 6H), 1.21 (s, 12H).

^{13}C NMR (100 MHz, CDCl$_3$) δ 159.90, 137.88 (t, *J* = 27.7 Hz), 132.18 (q, *J* = 32.8 Hz), 123.81 (q, *J* = 273.5 Hz), 117.42 (t, *J* = 220.4 Hz), 114.59.

^{19}F NMR (376 MHz, CDCl$_3$) δ -62.89 (s, 3F), -105.75 (s, 2F).

^{11}B NMR (128 MHz, CDCl$_3$) δ 30.27 (s, 1B).

HRMS (ESI) m/z calculated for $C_{20}H_{24}BF_5O_3^+$ (M^+): 418.1733, found 418.1722.

3-(Difluoro(3-(4,4,5,5-tetramethyl-1,3,2-dioxaborolan-2-yl)bicyclo[1.1.1]pentan-1-yl)methyl)-2-methoxypyridine (71)

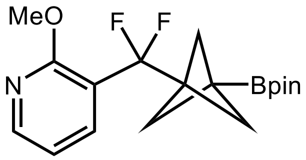

Following the **Procedure D**.

Purified by flash chromatography (PE:EA = 7:1) on silica gel to afford the title compound (263.7 mg, 62% yield) as a white solid.

^{1}H NMR (400 MHz, CDCl$_3$) δ 8.16 (d, *J* = 4.8 Hz, 1H), 7.66 (d, *J* = 7.4 Hz, 1H), 6.99 – 6.77 (m, 1H), 3.92 (s, 3H), 1.90 (s, 6H), 1.19 (s, 12H).

^{13}C NMR (100 MHz, CDCl$_3$) δ 160.94 (t, *J* = 3.3 Hz), 148.32, 136.17 (t, *J* = 7.9 Hz), 117.83 (t, *J* = 27.5 Hz), 117.29 (t, *J* = 243.2 Hz), 116.21, 83.62, 53.41, 49.81 (t, *J* = 3.6 Hz), 49.51 (t, *J* = 3.8 Hz), 46.05 (t, *J* = 34.8 Hz), 24.80.

^{19}F NMR (376 MHz, CDCl$_3$) δ -104.29 (s, 2F).

^{11}B NMR (128 MHz, CDCl$_3$) δ 30.44 (s, 1B).

HRMS (ESI) m/z calculated for $C_{18}H_{25}BF_2NO_3^+$ ($[M+H]^+$): 352.1890, found 352.1878.

6-(Difluoro(3-(4,4,5,5-tetramethyl-1,3,2-dioxaborolan-2-yl)bicyclo[1.1.1]pentan-1-yl)methyl)pyridin-2-amine (72)

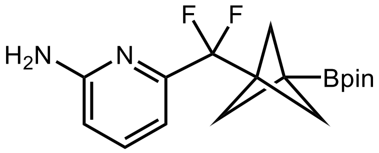

Following the **Procedure D**.

Purified by flash chromatography (PE:EA = 7:1) on silica gel to afford the title compound (247.3 mg, 61% yield) as a pale yellow solid.

^{1}H NMR (400 MHz, CDCl$_3$) δ 7.45 (t, *J* = 7.8 Hz, 1H), 6.81 (d, *J* = 7.4 Hz, 1H), 6.48 (d, *J* = 8.2 Hz, 1H), 4.61 (s, 1H), 1.95 (s, 6H), 1.21 (s, 12H).

^{13}C NMR (100 MHz, CDCl$_3$) δ 158.10, 152.07, 138.26, 116.04, 110.37 (t, *J* = 5.0 Hz), 109.48, 83.64, 49.50 (t, *J* = 3.6 Hz), 49.18 (t, *J* = 3.3 Hz), 45.73 (t, *J* = 34.1 Hz), 24.84.

^{19}F NMR (376 MHz, CDCl$_3$) δ -109.09 (s, 2F).

^{11}B NMR (128 MHz, CDCl$_3$) δ 30.06 (s, 1B).

HRMS (ESI) m/z calculated for $C_{17}H_{24}BF_2N_2O_2^+$ ([M+H]$^+$): 337.1893, found 337.1879.

***tert*-Butyl 5-(difluoro(3-(4,4,5,5-tetramethyl-1,3,2-dioxaborolan-2-yl)bicyclo[1.1.1]pentan-1-yl)methyl)-1*H*-indole-1-carboxylate (73)**

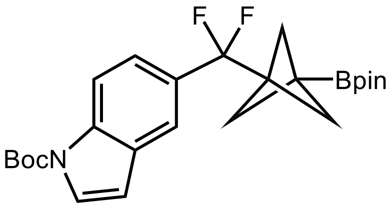

Following the **Procedure D**.

Purified by flash chromatography (PE:DCM = 20:1) on silica gel to afford the title compound (273.3 mg, 50% yield) as a white solid.

^{1}H NMR (400 MHz, CDCl$_3$) δ 8.22 (s, 1H), 7.66 (d, *J* = 3.4 Hz, 1H), 7.55 (d, *J* = 8.1 Hz, 1H), 7.25 (d, *J* = 8.3 Hz, 1H), 6.57 (d, *J* = 3.7 Hz, 1H), 1.93 (s, 6H), 1.68 (s, 9H), 1.21 (s, 12H).

^{13}C NMR (100 MHz, CDCl$_3$) δ 149.60, 134.69, 131.46, 131.28 (t, *J* = 26.6 Hz), 127.26, 120.63, 119.83 (t, *J* = 5.9 Hz), 118.81 (t, *J* = 243.2 Hz), 112.62 (t, *J* = 7.0 Hz), 107.05, 84.11, 83.59, 49.06 (t, *J* = 3.3 Hz), 48.28 (t, *J* = 3.5 Hz), 46.76 (t, *J* = 36.7 Hz).

^{19}F NMR (376 MHz, CDCl$_3$) δ -103.75 (s, 2F).

^{11}B NMR (128 MHz, CDCl$_3$) δ 30.81 (s, 1B).

HRMS (ESI) m/z calculated for $C_{25}H_{32}BF_2NO_4^+$ ([M]$^+$): 459.2387, found 459.2377.

2-(3-(Difluoro(4-phenoxyphenyl)methyl)bicyclo[1.1.1]pentan-1-yl)-4,4,5,5-tetramethyl-1,3,2-dioxaborolane (74)

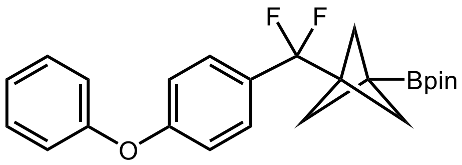

Following the **Procedure D**.

Purified by flash chromatography (PE = 100%) on silica gel to afford the title compound (332.9 mg, 67% yield) as a colorless oil.

^{1}H NMR (400 MHz, CDCl$_3$) δ 7.36 (t, *J* = 8.2 Hz, 4H), 7.14 (t, *J* = 7.4 Hz, 1H), 7.01 (dd, *J* = 10.8, 8.6 Hz, 4H), 1.92 (s, 6H), 1.23 (s, 12H).

^{13}C NMR (100 MHz, CDCl$_3$) δ 158.56, 156.64, 130.53 – 129.40 (m), 126.99 (t, *J* = 6.0 Hz), 123.90, 119.46, 118.25 (t, *J* = 242.7 Hz), 118.05, 83.66, 48.95 (t, *J* = 3.5 Hz), 48.60 (t, *J* = 3.5 Hz), 46.51 (t, *J* = 36.5 Hz), 24.80.

^{19}F NMR (376 MHz, CDCl$_3$) δ -104.45 (s, 2F).

^{11}B NMR (128 MHz, CDCl$_3$) δ 30.29 (s, 1B).

HRMS (ESI) m/z calculated for $C_{24}H_{27}BF_2O_3^+$ ([M]$^+$): 412.2016, found 412.2006.

3-(Difluoro(5-(4,4,5,5-tetramethyl-1,3,2-dioxaborolan-2-yl)bicyclo[3.1.1]heptan-1-yl)methyl)-2-methoxypyridine (75)

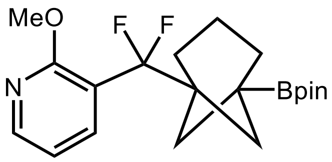

Following the **Procedure E**.

Purified by flash chromatography (PE:EA = 20:1) on silica gel to afford the title compound (124.2 mg, 27% yield) as a white solid.

^{1}H NMR (400 MHz, CDCl$_3$) δ 8.17 (d, *J* = 4.1 Hz, 1H), 7.66 (d, *J* = 7.4 Hz, 1H), 6.92 – 6.82 (m, 1H), 3.92 (s, 3H), 2.41 (d, *J* = 7.5 Hz, 2H), 1.75 (s, 4H), 1.52 (d, *J* = 6.3 Hz, 2H), 1.45 (d, *J* = 7.1 Hz, 2H), 1.24 (s, 12H).

^{13}C NMR (100 MHz, CDCl$_3$) δ 160.74, 148.09, 137.13 (t, *J* = 8.3 Hz), 122.21 (d, *J* = 243.6 Hz), 118.42 (t, *J* = 28.2 Hz), 116.12, 83.23, 53.42, 47.70 (t, *J* = 28.8 Hz), 33.96 (t, *J* = 4.4 Hz), 29.35, 24.84, 16.23.

^{19}F NMR (376 MHz, CDCl$_3$) δ -106.41 (s, 2F)

^{11}B NMR (128 MHz, CDCl$_3$) δ 32.79 (s, 1B).

HRMS (ESI) m/z calculated for $C_{20}H_{29}BF_2NO_3^+$ ([M+H]$^+$): 380.2203, found 380.2195.

3-(Difluoro(6-(4,4,5,5-tetramethyl-1,3,2-dioxaborolan-2-yl)bicyclo[4.1.1]octan-1-yl)methyl)-2-methoxypyridine (76)

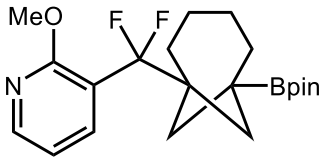

Following the **Procedure E**.

Purified by flash chromatography (PE:EA = 20:1) on silica gel to afford the title compound (58.0 mg, 12% yield) as a white solid.

^{1}H NMR (400 MHz, CDCl$_3$) δ 8.17 (d, *J* = 4.7 Hz, 1H), 7.69 (d, *J* = 7.5 Hz, 1H), 7.02 – 6.82 (m, 1H), 3.93 (s, 3H), 2.68 (d, *J* = 12.1 Hz, 2H), 1.73 – 1.66 (m, 8H), 1.59 (t, *J* = 5.4 Hz, 2H), 1.23 (s, 12H).

^{13}C NMR (100 MHz, CDCl$_3$) δ 160.70, 148.04, 137.45 (t, *J* = 8.6 Hz), 123.10, 117.47, 116.14, 83.22, 53.44, 47.65 (t, *J* = 26.4 Hz), 32.01, 31.70, 30.98 (t, *J* = 5.2 Hz), 25.43, 25.14, 24.86, 24.76.

^{19}F NMR (376 MHz, CDCl$_3$) δ -106.03 (s, 2F).

^{11}B NMR (128 MHz, CDCl$_3$) δ 33.85 (s, 1B).

HRMS (ESI) m/z calculated for $C_{21}H_{31}BF_2NO_3^+$ ($[M+H]^+$): 394.2360, found 394.2357.

4-(3-(Difluoro(3-(4,4,5,5-tetramethyl-1,3,2-dioxaborolan-2-yl)bicyclo[1.1.1]pentan-1-yl)methyl)-5-(trifluoromethyl)phenoxy)-1,2,2,6,6-pentamethylpiperidine (77)

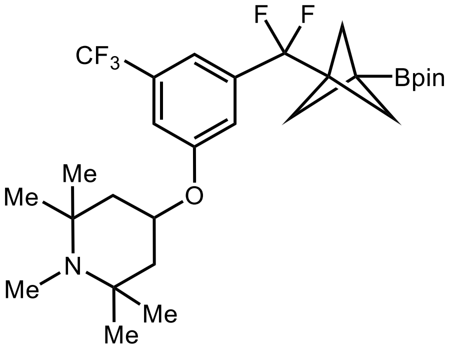

Following the **Procedure D**.

Purified by flash chromatography (PE:EA:Et_3N = 5:1) on silica gel to afford the title compound (318.3 mg, 48% yield) as a white solid.

^{1}H NMR (400 MHz, $CDCl_3$) δ 7.17 (s, 1H), 7.13 (s, 1H), 7.05 (s, 1H), 4.56 (tt, *J* = 11.1, 3.5 Hz, 1H), 2.28 (s, 3H), 1.97 (dd, *J* = 12.4, 2.6 Hz, 2H), 1.89 (s, 6H), 1.59 (t, *J* = 11.7 Hz, 2H), 1.21 (s, 18H), 1.13 (s, 6H).

^{13}C NMR (100 MHz, $CDCl_3$) δ 158.01, 137.83 (t, *J* = 27.5 Hz), 132.19 (q, *J* = 32.7 Hz), 123.74 (q, *J* = 272.5 Hz), 117.49 (d, *J* = 243.2 Hz), 115.87 (t, *J* = 6.0 Hz), 114.26 (q, *J* = 5.8 Hz), 113.66 (t, *J* = 3.0 Hz), 83.78, 70.89, 55.41, 48.95, 46.13 (t, *J* = 35.4 Hz), 46.06, 33.12, 28.22, 24.84, 21.20.

^{19}F NMR (376 MHz, $CDCl_3$) δ -62.91 (s, 3F), -105.90 (s, 2F)

^{11}B NMR (128 MHz, $CDCl_3$) δ 30.52.

HRMS (ESI) m/z calculated for $C_{29}H_{42}BF_5NO_3^+$ ($[M+H]^+$): 558.3172, found 558.3148.

2-(3-(Difluoro(3-(((3aR,5R,5aS,8aS,8bR)-2,2,7,7-tetramethyltetrahydro-5H-bis([1,3]dioxolo)[4,5-b:4',5'-d]pyran-5-yl)methoxy)-5-(trifluoromethyl)phenyl)methyl)bicyclo[1.1.1]pentan-1-yl)-4,4,5,5-tetramethyl-1,3,2-dioxaborolane (78)

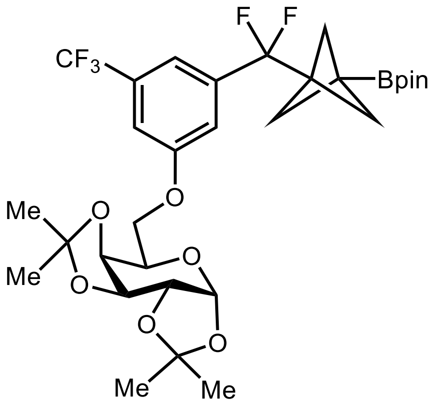

Following the **Procedure D**.

Purified by flash chromatography (PE:EA = 6:1) on silica gel to afford the title compound (318.0 mg, 41% yield) as a colorless oil.

^{1}H NMR (400 MHz, CDCl$_3$) δ 7.20 (s, 2H), 7.13 (s, 1H), 5.57 (d, *J* = 5.0 Hz, 1H), 4.67 (dd, *J* = 7.9, 2.0 Hz, 1H), 4.39 – 4.32 (m, 3H), 4.24 – 4.12 (m, 4H), 1.89 (s, 6H), 1.54 (s, 3H), 1.48 (s, 3H), 1.37 (s, 3H), 1.35 (s, 3H), 1.21 (s, 12H).

^{13}C NMR (100 MHz, CDCl$_3$) δ 158.94, 137.79 (t, *J* = 27.8 Hz), 132.08 (q, *J* = 33.1 Hz), 123.69 (d, *J* = 274.0 Hz), 117.45 (t, *J* = 243.6 Hz), 115.35, 114.78, 112.91, 109.78, 108.98, 96.49, 83.75, 71.03, 70.78, 70.68, 67.43, 66.35, 48.97 (t, *J* = 3.2 Hz), 46.07 (t, *J* = 35.6 Hz), 45.99, 29.82, 26.19, 26.11, 25.03, 24.82, 24.55.

^{19}F NMR (376 MHz, CDCl$_3$) δ -62.84 (s, 3F), -105.56 (d, *J* = 29.2 Hz, 2F).

^{11}B NMR (128 MHz, CDCl$_3$) δ 30.46.

HRMS (ESI) m/z calculated for $C_{31}H_{40}BF_5O_8^+$ ([M]$^+$): 646.2731, found 646.2700.

_N_-(2-(Difluoro(3-hydroxybicyclo[1.1.1]pentan-1-yl)methyl)phenyl)acetamide (79)

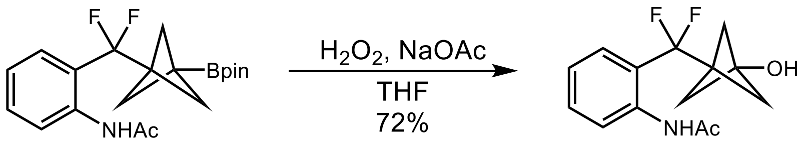

Follow the reported procedures in the literature[15], to a solution of **65** (75.4 mg, 0.2 mmol) and NaOAc (32.8 mg, 0.4 mmol) in THF (4.0 mL) at 0 °C was added H_2O_2 (30 wt.% in water, 0.24 mL) dropwise. The resulting mixture was stirred at room temperature for 2 hours. Saturated aqueous solution of $Na_2S_2O_3$ was added and the mixture was stirred for 10 min. The mixture was extracted with Et_2O (4 × 10 mL). The combined organic layers were washed with brine, dried over anhydrous Na_2SO_4, concentrated and purified by column chromatography (PE:EA = 1:9) on silica gel to afford title compound (38.6 mg, 72% yield) as a white solid.

^{1}H NMR (400 MHz, CDCl$_3$) δ 7.96 (d, *J* = 7.2 Hz, 1H), 7.71 (s, 0H), 7.49 – 7.32 (m, 2H), 7.18 (t, *J* = 7.0 Hz, 1H), 3.20 (s, 1H), 2.16 (s, 3H), 1.94 (d, *J* = 4.3 Hz, 7H).

^{13}C NMR (100 MHz, CDCl$_3$) δ 168.39, 134.42, 130.91, 126.38 (t, *J* = 8.3 Hz), 125.26 (t, *J* = 7.6 Hz), 124.91, 124.86, 122.21 (d, *J* = 241.4 Hz), 62.96, 52.58, 34.25 (t, *J* = 36.1 Hz), 24.80.

^{19}F NMR (376 MHz, CDCl$_3$) δ -95.67 (d, *J* = 6.1 Hz, 2F).

HRMS (ESI) m/z calculated for $C_{14}H_{16}F_2NO_2^+$ ($[M+H]^+$): 268.1144, found 268.1138

N-**(2-(Difluoro(3-(trifluoro-l4-boraneyl)bicyclo[1.1.1]pentan-1-yl)methyl)phenyl)acetamide, potassium salt (80)**

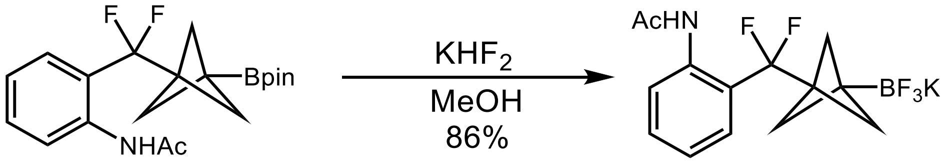

Follow the reported procedures in the literature[15], to a solution of **65** (415 mg, 1.1 mmol) in MeOH (5 mL) was added KHF_2 solution (2 mL, 10 mmol). The system was stirred at room temperature for 2 hours. Then, the solvents were evaporated to dryness under reduced pressure. The residue was extracted with hot acetone (4 × 10 mL), followed by filtration. The combined filtrates were concentrated to 2 mL. Cold Et_2O was added into the system, and the resultant precipitate was collected and dried to afford the title compound (339 mg, 86%) as a white solid.

^{1}H NMR (400 MHz, DMSO-*d*$_6$) δ 8.72 (s, 1H), 7.53 (d, *J* = 8.0 Hz, 1H), 7.41 (t, *J* = 7.7 Hz, 1H), 7.33 (d, *J* = 7.8 Hz, 1H), 7.23 (t, *J* = 7.5 Hz, 1H), 2.04 (s, 3H), 1.30 (s, 6H).

^{13}C NMR (100 MHz, DMSO-*d6*) δ 168.52, 134.80, 129.78, 128.07 (t, *J* = 24.4 Hz), 127.57, 126.13 (t, *J* = 8.3 Hz), 124.94, 119.50 (t, *J* = 242.5 Hz), 46.87, 43.13 (t, *J* = 33.8 Hz), 30.76, 23.67.

^{19}F NMR (376 MHz, DMSO-*d6*) δ -97.73 (s, 2F), -143.09(s, 3F)

^{11}B NMR (128 MHz, DMSO-*d6*) δ 1.48 (s, 1B)

HRMS (ESI) m/z calculated for $C_{14}H_{14}BF_5NO^-$ ([M-K$^+$]$^-$): 318.1094, found 318.1085

***N*-(2-(difluoro(3-(4-methylquinolin-2-yl)bicyclo[1.1.1]pentan-1-yl)methyl)phenyl)acetamide (81)**

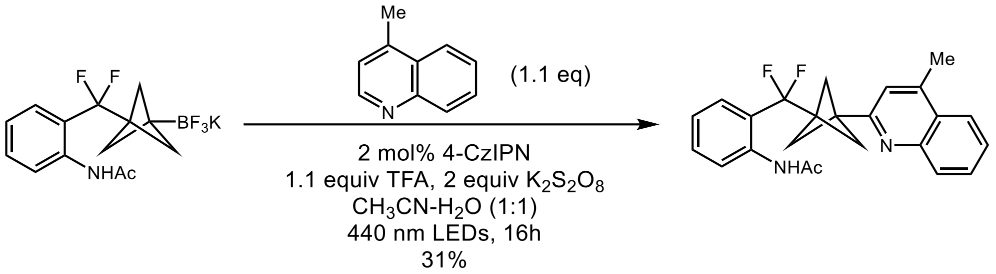

Follow the reported procedures in the literature[16], to a 8 mL reaction vial was added BCP-BF_3K **76** (35.7 mg, 0.1 mmol), 4-CzIPN (4 mg, 0.005 mmol, 0.05 equiv), 4-methylquinoline (15 µL, 0.11 mmol, 1.1 equiv.), $K_2S_2O_8$ (54 mg, 0.2 mmol, 2 equiv.), TFA (8 µL, 0.11 mmol, 1.1 equiv.) and MeCN/H_2O (1:1, 1 mL). The mixture was irradiated with 40W blue LEDs (440 nm) at room temperature. After 16 hours, saturated aqueous solution of $NaHCO_3$ was added into the system, and the mixture was extracted with DCM (4 × 5 mL). The combined organic layers were washed with brine, dried over anhydrous Na_2SO_4, concentrated and purified by column chromatography (PE:EA = 1:3) to afford colorless oil **S10** (12.1 mg, 31%).

^{1}H NMR (400 MHz, $CDCl_3$) δ 8.10 (d, *J* = 8.1 Hz, 1H), 8.04 (d, *J* = 8.5 Hz, 1H), 7.93 (d, *J* = 8.3 Hz, 1H), 7.84 (s, 1H), 7.66 (t, *J* = 7.7 Hz, 1H), 7.48 (dt, *J* = 21.1, 7.7 Hz, 4H), 7.20 (t, *J* = 7.5 Hz, 1H), 7.10 (s, 1H), 2.65 (s, 4H), 2.27 (s, 7H), 2.21 (s, 3H).

^{13}C NMR (100 MHz, $CDCl_3$) δ 168.24, 157.96, 147.81, 144.75, 134.88, 130.91, 129.81, 129.44, 127.26, 126.50, 126.16, 124.63, 124.44, 123.74, 120.99 (d, *J* = 242.7 Hz), 119.46, 50.29, 42.74, 41.02 (t, *J* = 35.7 Hz), 25.01, 18.81.

^{19}F NMR (376 MHz, CDCl$_3$) δ -98.92 (d, *J* = 5.7 Hz, 2F).

HRMS (ESI) m/z calculated for $C_{24}H_{23}F_2N_2O^+$ ($[M+H]^+$): 393.1773, found 393.1757.

***N*-(2-((3-(2-cyanoethyl)bicyclo[1.1.1]pentan-1-yl)difluoromethyl)phenyl)acetamide (82)**

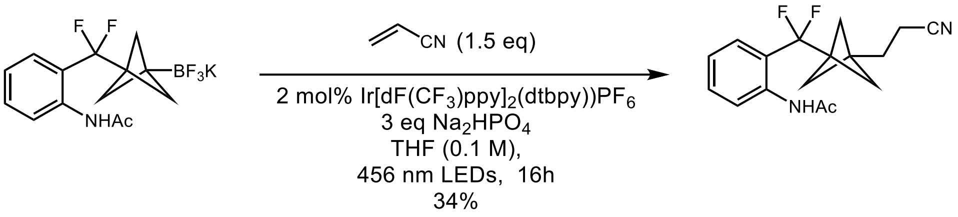

Follow the reported procedures in the literature[17], to a 8 mL reaction vial was added BCP-BF_3K **76** (35.7 mg, 0.1 mmol), Ir[dF(CF_3)ppy]$_2$(dtbpy)PF_6 (2.2 mg, 0.002 mmol, 0.02 equiv), acrylonitrile (20 µL, 0.3 mmol, 3 equiv), Na_2HPO_4 (43 mg, 0.3 mmol, 3 equiv), and THF (1 mL). The mixture was irradiated with a 40W blue LEDs (456 nm) at room temperature. After 16 hours, solvent was removed *in vacuo*, and the crude material was purified by column chromatography (PE:EA = 1:2) to afford colorless oil **S11** (10.8 mg, 34%).

^{1}H NMR (400 MHz, Chloroform-*d*) δ 8.01 (d, *J* = 8.0 Hz, 1H), 7.73 (s, 1H), 7.43 (t, *J* = 7.8 Hz, 1H), 7.37 (d, *J* = 7.7 Hz, 1H), 7.18 (t, *J* = 7.5 Hz, 1H), 2.26 (t, *J* = 7.2 Hz, 2H), 2.17 (s, 3H), 1.83 (t, *J* = 7.2 Hz, 2H), 1.77 (s, 6H).

^{13}C NMR (100 MHz, Chloroform-*d*) δ 168.22, 134.67, 130.90, 126.46 (t, *J* = 8.2 Hz), 124.94 (t, *J* = 5.2 Hz), 124.73, 124.72, 121.58 (d, *J* = 241.4 Hz), 119.38, 48.46 (t, *J* = 3.1 Hz), 41.35 (t, *J* = 35.8 Hz), 38.67, 27.15, 24.88, 14.43.

^{19}F NMR (376 MHz, Chloroform-*d*) δ -99.00 (d, *J* = 5.5 Hz, 2F).

HRMS (ESI) m/z calculated for $C_{17}H_{19}F_2N_2O^+$ ([M+H]]$^+$): 305.1460, found 305.1449.

***N*-(1-Benzylpiperidin-4-yl)-2-(4-(bicyclo[1.1.1]pentan-1-yldifluoromethyl)phenoxy)acetamide (83)**

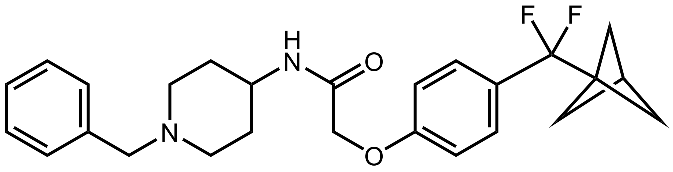

Following the **Procedure A** using **PCN (1)** and Cs_2CO_3 as suitable reagents.
Purified by flash chromatography (Tol:PE:EA:DCM:Et_3N = 2:2:1:1:0.2) on silica gel to afford the title compound (92.4 mg, 35% yield) as a white solid.

^{1}H NMR (400 MHz, $CDCl_3$) δ 7.35 (d, *J* = 8.8 Hz, 2H), 7.33 – 7.23 (m, 5H), 6.93 (d, *J* = 8.7 Hz, 2H), 6.41 (d, *J* = 8.4 Hz, 1H), 4.48 (s, 2H), 3.91 (tdd, *J* = 10.8, 8.6, 4.3 Hz, 1H), 3.50 (s, 2H), 2.81 (d, *J* = 11.5 Hz, 2H), 2.52 (s, 1H), 2.21 – 2.09 (m, 2H), 1.92 (dd, *J* = 13.1, 3.7 Hz, 2H), 1.81 (s, 6H), 1.52 (qd, *J* = 11.3, 3.8 Hz, 2H).

^{13}C NMR (100 MHz, $CDCl_3$) δ 167.14, 157.96, 138.29, 129.41 (t, *J* = 27.7 Hz), 129.24, 128.40, 127.27, 127.11 (t, *J* = 6.0 Hz), 118.60 (t, *J* = 242.0 Hz), 114.40, 67.39, 63.10, 52.23, 48.16 (t, *J* = 3.4 Hz), 46.35 (t, *J* = 36.8 Hz), 46.28, 32.18, 27.65 (t, *J* = 1.7 Hz).

^{19}F NMR (376 MHz, $CDCl_3$) δ -102.83 (s, 2F).

HRMS (ESI) m/z calculated for $C_{26}H_{31}F_2N_2O_2^+$ ($[M+H]^+$): 441.2348, found 411.2340.

***N*-(1-Benzylpiperidin-4-yl)-2-(4-(bicyclo[3.1.1]heptan-1-yldifluoromethyl)phenoxy)acetamide (84)**

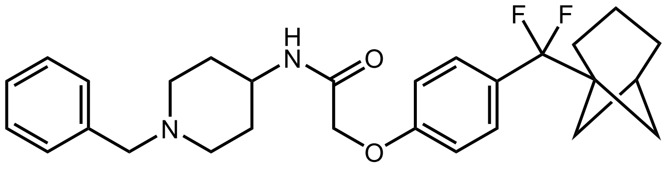

Following the **Procedure B** using **PCN (1)** and Cs_2CO_3 as suitable reagents.
Purified by flash chromatography (Tol:PE:EA:DCM:Et_3N = 2:2:1:1:0.2) on silica gel to afford the title compound (44.9 mg, 16% yield) as a white solid.

^{1}H NMR (400 MHz, CDCl$_3$) δ 7.45 (d, *J* = 6.5 Hz, 2H), 7.37 (q, *J* = 5.9 Hz, 3H), 7.31 (d, *J* = 8.8 Hz, 2H), 6.92 (d, *J* = 8.7 Hz, 2H), 6.59 (d, *J* = 7.2 Hz, 1H), 4.47 (s, 2H), 3.98 (dd, *J* = 15.0, 8.8 Hz, 1H), 3.80 (s, 2H), 3.11 (s, 2H), 2.49 – 2.38 (m, 2H), 2.32 – 2.21 (m, 3H), 1.99 (s, 4H), 1.73 (s, 4H), 1.58 (s, 2H), 1.39 – 1.32 (m, 2H).

^{13}C NMR (100 MHz, CDCl$_3$) δ 167.50, 157.81, 136.00, 130.31, 129.73, 128.96, 128.80, 127.59 (t, *J* = 6.3 Hz), 122.63 (t, *J* = 243.9 Hz), 28.92, 16.04, 114.26, 67.34, 62.15, 51.84, 47.98, 45.26, 31.82 (t, *J* = 3.7 Hz), 31.08 – 30.06 (m), 30.01 – 29.68 (m), 28.68 (t, *J* = 3.5 Hz), 28.47.

^{19}F NMR (376 MHz, CDCl$_3$) δ -106.12 (s, 2F).

HRMS (ESI) m/z calculated for $C_{28}H_{35}F_2N_2O_2^+$ ($[M+H]^+$): 469.2661, found 469.2651.

***N-(1*-Benzylpiperidin-4-yl)-2-(4-(bicyclo[4.1.1]octan-1-yldifluoromethyl)phenoxy)acetamide (85)**

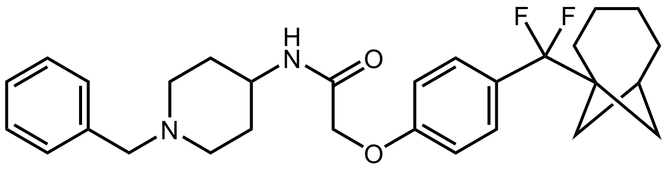

Following the **Procedure C** using **PCN (1)** and Cs_2CO_3 as suitable reagents.
Purified by flash chromatography (Tol:PE:EA:DCM:Et_3N = 2:2:1:1:0.2) on silica gel to afford the title compound (39.8 mg, 14% yield) as a white solid.

^{1}H NMR (400 MHz, CDCl$_3$) δ 7.32 – 7.18 (m, 7H), 6.84 (d, *J* = 8.6 Hz, 2H), 6.38 (d, *J* = 7.9 Hz, 1H), 4.41 (s, 2H), 3.87 (td, *J* = 14.8, 7.3 Hz, 1H), 3.50 (s, 2H), 2.81 (d, *J* = 10.7 Hz, 2H), 2.61 – 2.49 (m, 2H), 2.36 (t, *J* = 8.6 Hz, 1H), 2.14 (t, *J* = 11.1 Hz, 2H), 1.87 (d, *J* = 11.5 Hz, 2H), 1.64 – 1.45 (m, 10H), 1.22 (d, *J* = 28.3 Hz, 2H).

^{13}C NMR (100 MHz, CDCl$_3$) δ 167.25, 157.85, 137.91, 130.03, 129.45, 128.51, 127.66 (d, *J* = 6.2 Hz), 127.57, 123.48 (t, *J* = 243.1, 240.2 Hz), 114.24, 67.46, 62.90, 52.13, 47.61

(t, *J* = 27.3 Hz), 46.08, 31.84, 31.73 (t, *J* = 2.7 Hz), 30.62, 29.83, 28.86 (t, *J* = 4.6 Hz), 28.13, 25.04, 24.69.

^{19}F NMR (376 MHz, $CDCl_3$) δ -105.67 (s, 2F).

HRMS (ESI) m/z calculated for $C_{29}H_{37}F_2N_2O_2^+$ ($[M+H]^+$): 483.2818, found 483.2805.

12) Evaluation of ADME Properties

Selected compounds were screened for aqueous solubility in PBS (pH 7.4), metabolic stability in Human liver microsomes (MLM) as a measure of clearance and finally inhibition of representative CYP450 enzymes. ADME studies reported in this work were independently performed by Chinese Academy of Sciences Shanghai Institute of Materia Medica (Shanghai, China).

Metabolic stability in Human Liver Microsomes

Microsomes in 0.1 M TRIS buffer pH 7.4 (final concentration 0.33 mg/mL), co-factor MgCl2 (final concentration 5 mM) and tested compound (final concentration 0.1 μM, co-solvent (0.01% DMSO) and 0.005% Bovin serum albumin (BSA)) were incubated at 37°C for 10 min. The reaction was started by the addition of NADPH (final concentration 1 mM). Aliquots were sampled at 0, 5, 15, 30 and 60 min respectively and methanol (cold in wet ice) was added to terminate the reaction. After centrifugation (4000 rpm,5 min), samples were then analyzed by LC-MS/MS.

Intrinsic clearance:

$$Cl_{int} = \frac{1000 \times slope}{P}$$

In vivo clearance:

$$Cl_{int,\ in\,vivo} = \frac{Cl_{int} \times Houston \times LW}{1000}$$

Hepatic clearance:

$$Cl_{hep} = \frac{HBF \times fu \times Cl_{int,\ in\,vivo}}{HBF + fu \times Cl_{int,\ in\,vivo}}$$

Metabolic bioavailability:

$$\%MF = 100 - \frac{Cl_{int} \times 100}{HBF}$$

Where *P* is microsomal protein concentration (mg/mL); *Houston* is Houston factor (45 mg of microsomal protein/g liver); *LW* is liver weight (g) (per species); *HBF* is hepatic blood flow (mL/min) (per species); *fu* is unbound fraction (fu=1 is generally used).

Cell Permeability Studies

Caco-2 cells purchased from ATCC cultured in high glucose DMEM medium in an incubator with 37 ℃, 5% CO_2 and 90% relative air humidity and 10% fetal cattle, 10mmol/L HEPES, 1mmol/L Sodium pyruvate, 1% glutamine, 1% non essential amino acid, 100U/mL penicillin and 100 μ G/mL Streptomycin were added to the medium Serum. After washing cells with HBSS three times, the compounds (10 μM) were added to the corresponding cell pores (pH 6.8 on the A side and pH 7.4 on the B side). Incubate in a 37 ℃ incubator for 95 mins, take samples from the administration side at 5 mins and 95 mins respectively, and take samples from the receiving side at 35 mins and 95 mins respectively. Use LC-MS/MS to detect the concentration of the sample. Measure the Papp and Efflux values of the tested compound.

apparent permeability coefficient:

$$P_{app} = \frac{(dQ/dt)}{A \times C_0}$$

efflux ratio:

$$Efflux\ Ratio = \frac{P_{app(B \to A)}}{P_{app(A \to B)}}$$

Where dQ/dt is the Drug transport volume per unit time; A is the surface area for the transport; C_0 is the initial concentration in the donor chamber (μM).

13) References

1. Perrin, D. D.; Armarego, W. L. F. *Purification of Laboratory Chemicals* (Pergamon Press: Oxford, 1988) ed 3..
2. Lowry, M. S. *et al.* Single-Layer Electroluminescent Devices and Photoinduced Hydrogen Production from an Ionic Iridium(III) Complex. *Chem. Mater.* **17**, 5712–5719 (2005).
3. Li, X. & Zhou, Q. Manganese-Catalyzed Selective Hydrogenative Cross-Coupling of Nitriles and Amines to Form Secondary Imines. *Adv. Synth. Catal.* **363**, 3471–3475 (2021).
4. Cho, S. *et al.* Heterocyclic Compound and Organic Light Emitting Device Comprising the Same. (2021).
5. Wang, L., Cui, D., Hou, Z., Li, W. & Li, Y. Highly Cis-1,4-Selective Living Polymerization of 1,3-Conjugated Dienes and Copolymerization with ε-Caprolactone by Bis(phosphino)carbazolide Rare-Earth-Metal Complexes. *Organometallics* **30**, 760–767 (2011).
6. Frank, N. *et al.* Synthesis of meta-substituted arene bioisosteres from [3.1.1]propellane. *Nature* **611**, 721–726 (2022).
7. Pickford, H. D. *et al.* Twofold Radical-Based Synthesis of N,C-Difunctionalized Bicyclo[1.1.1]pentanes. *J. Am. Chem. Soc.* **143**, 9729–9736 (2021).
8. Fuchs, J. & Szeimies, G. Synthese von [n.1.1]Propellanen (n = 2, 3, 4). *Chem. Ber.* **125**, 2517–2522 (1992).
9. Kulinkovich, O. G., Kozyrkov, Y. Y., Bekish, A. V., Matiushenkov, E. A. & Lysenko, I. L. A Convenient Way for the Conversion of Carboxylic Esters into 2-Substituted Allyl Halides. *Synthesis* 1713–1717 (2005) doi:10.1055/s-2004-834869.
10. Kozyrkov, Y. Yu. & Kulinkovich, O. G. A Simple and Efficient Conversion of Tertiary Cyclopropanols to 2-Substituted Allyl Halides. *Synlett* **2002**, 0443–0446 (2002).
11. Yayla, H. G. *et al.* Discovery and mechanistic study of a photocatalytic indoline dehydrogenation for the synthesis of elbasvir. *Chem. Sci.* **7**, 2066–2073 (2016).
12. Hatchard, C. G., Parker, C. A. & Bowen, E. J. A new sensitive chemical actinometer - II. Potassium ferrioxalate as a standard chemical actinometer. *Proceedings of the Royal Society of London. Series A. Mathematical and Physical Sciences* **235**, 518–536 (1997).

13 Cismesia, M. A. & Yoon, T. P. Characterizing chain processes in visible light photoredox catalysis. *Chem. Sci.* **6**, 5426–5434 (2015).

14. Buzzetti, L., Crisenza, G. E. M. & Melchiorre, P. Mechanistic Studies in Photocatalysis. *Angew Chem Int Ed* **58**, 3730–3747 (2019).

15. Yang, Y. *et al.* An intramolecular coupling approach to alkyl bioisosteres for the synthesis of multisubstituted bicycloalkyl boronates. *Nat. Chem.* **13**, 950–955 (2021).

16. Dong, W. *et al.* Exploiting the sp2 character of bicyclo[1.1.1]pentyl radicals in the transition-metal-free multi-component difunctionalization of [1.1.1]propellane. *Nat. Chem.* **14**, 1068–1077 (2022).

17. Matsui, J. K., Primer, D. N. & Molander, G. A. Metal-free C–H alkylation of heteroarenes with alkyltrifluoroborates: a general protocol for 1°, 2° and 3° alkylation. *Chem. Sci.* **8**, 3512–3522 (2017).

14) Spectral Data

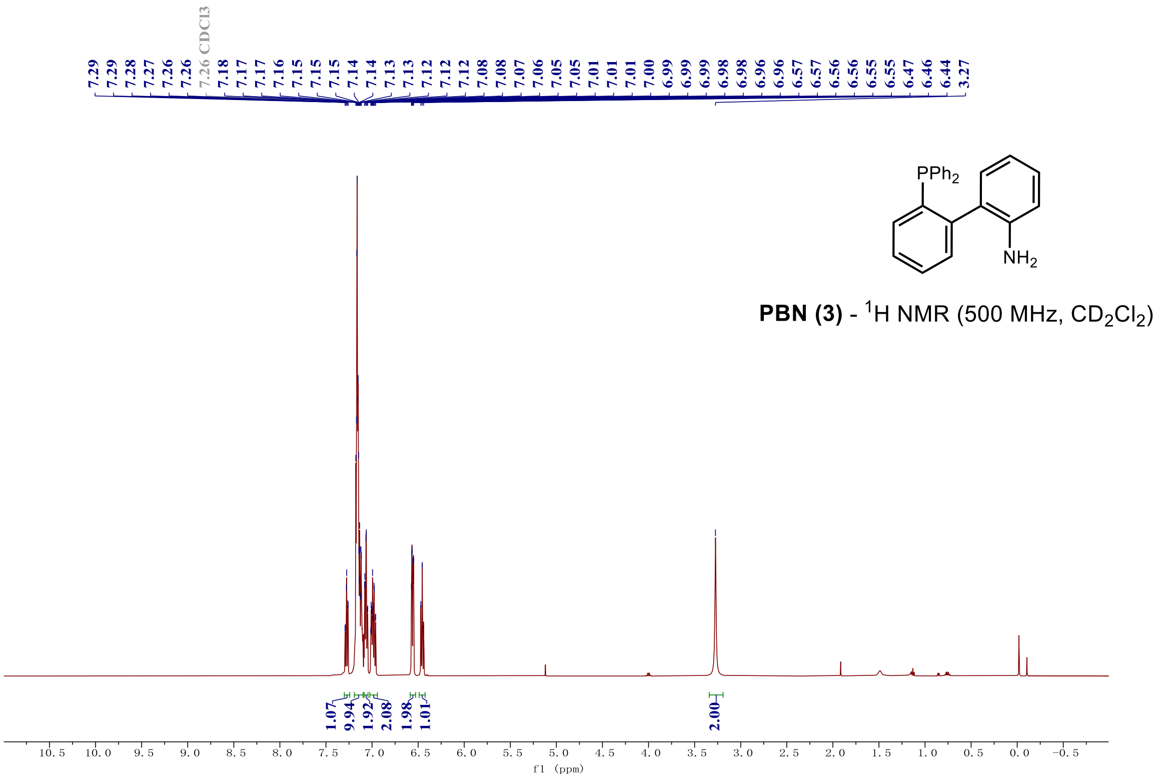

Supplementary Figure 17 ^{1}H NMR spectra of compound **3**

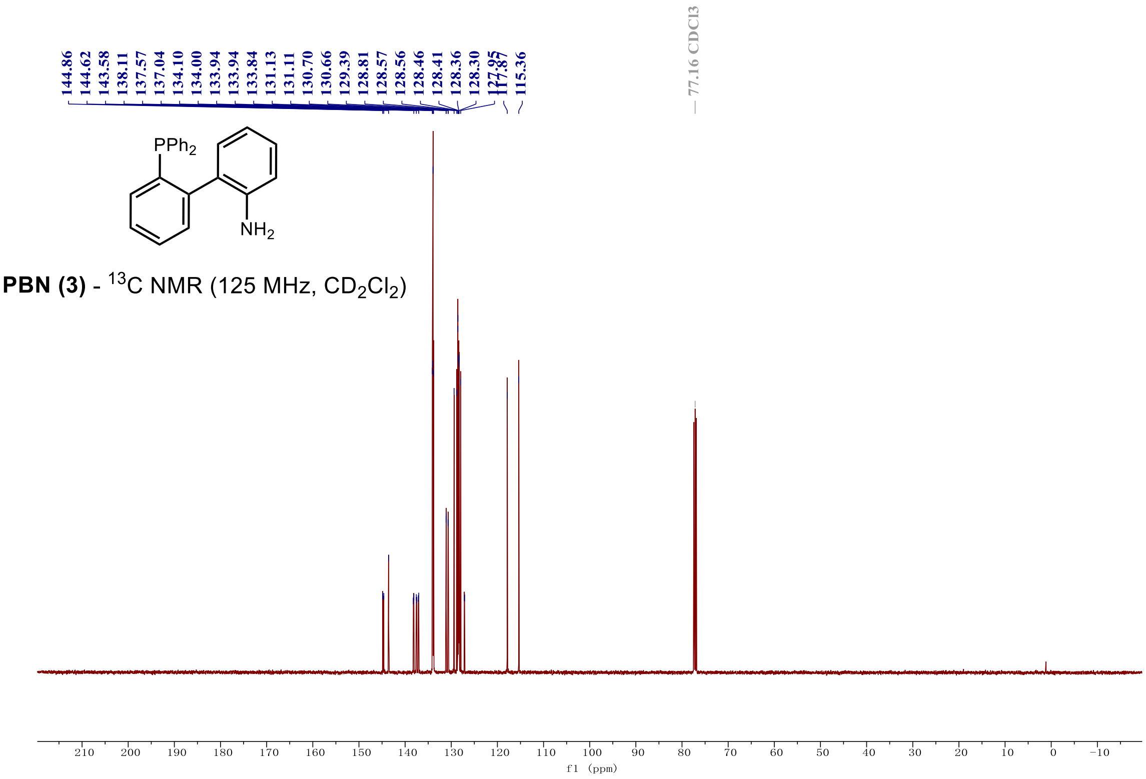

Supplementary Figure 18 ^{13}C NMR spectra of compound **3**

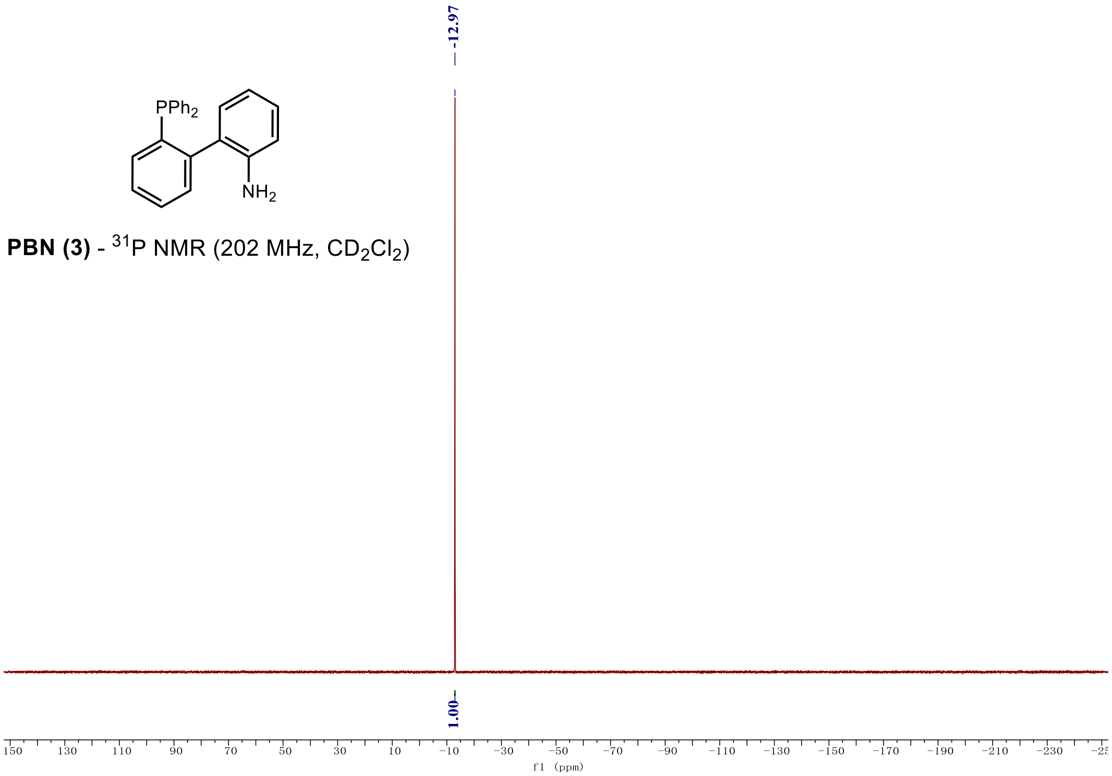

Supplementary Figure 19 ^{31}P NMR spectra of compound **3**

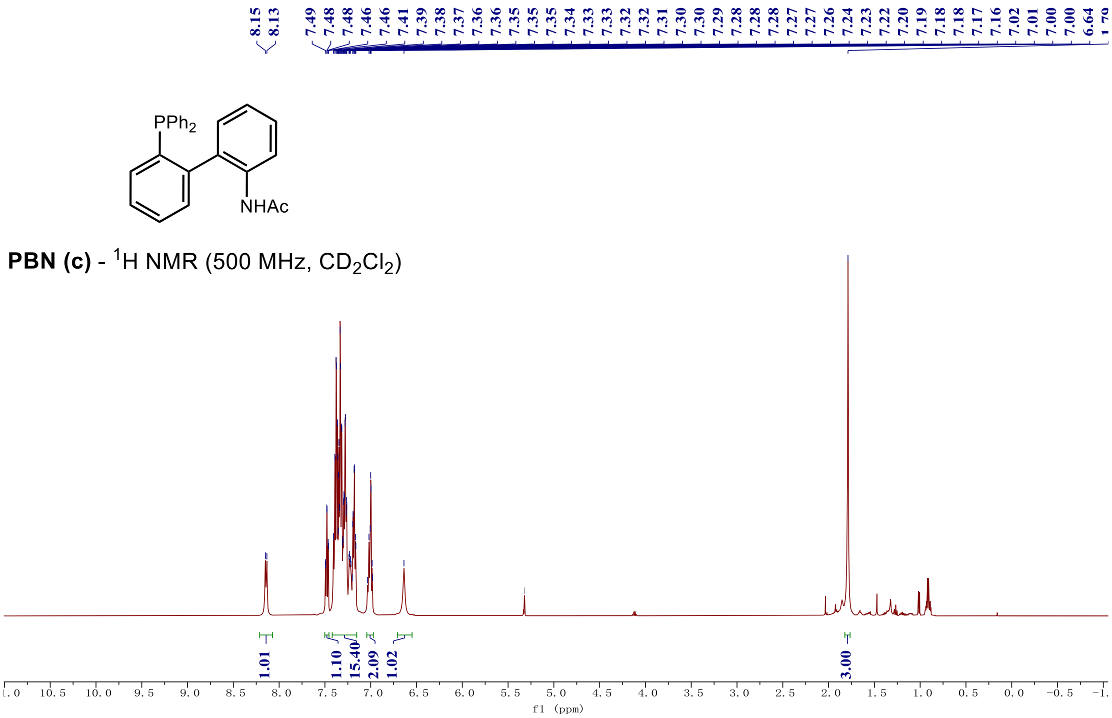

Supplementary Figure 20 ^{1}H NMR spectra of compound **c**

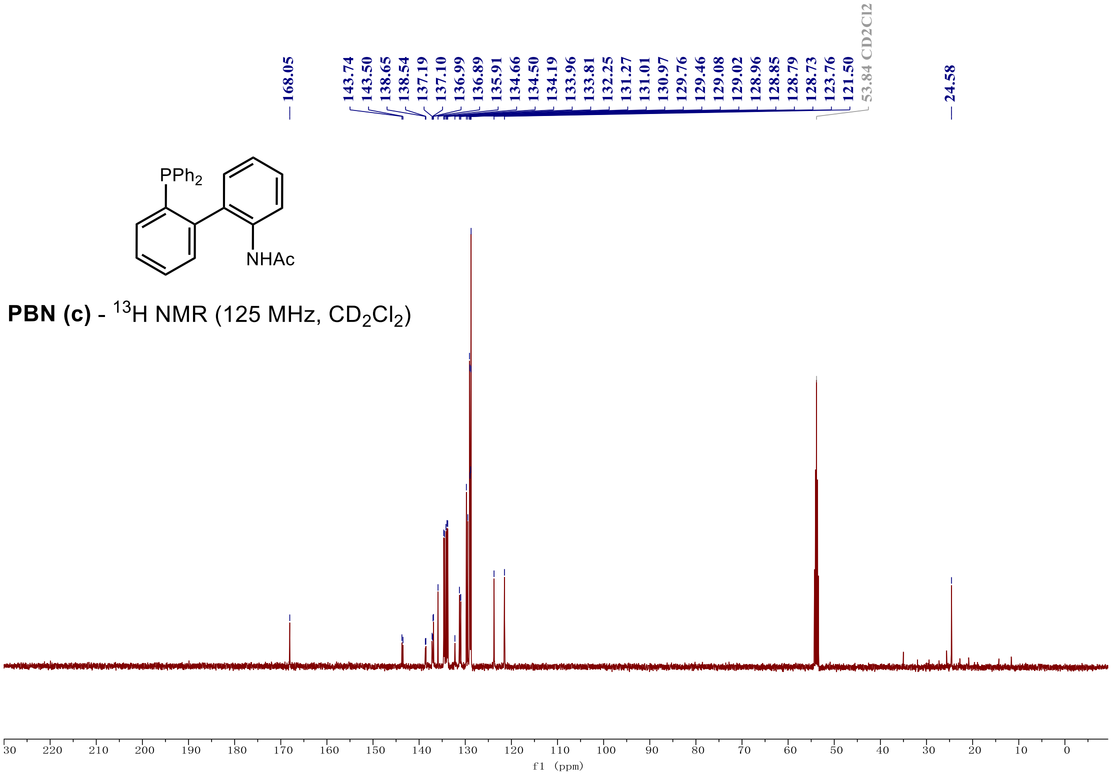

Supplementary Figure 21 ^{13}C NMR spectra of compound **c**

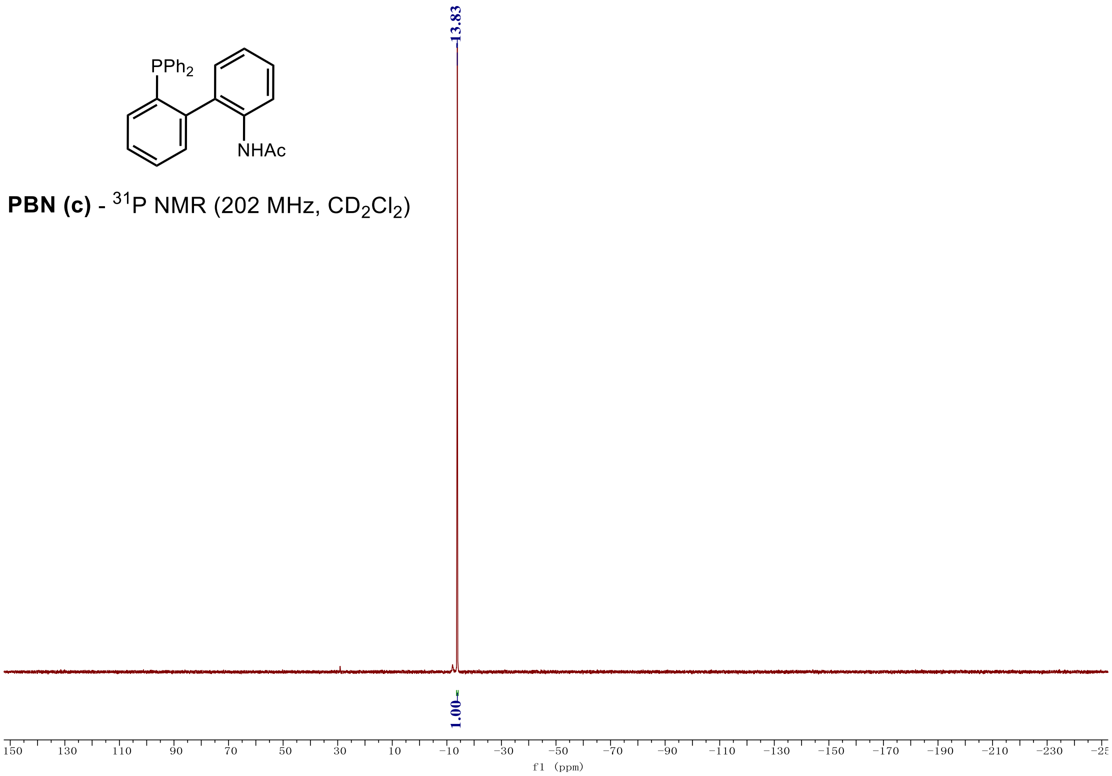

Supplementary Figure 22 ^{31}P NMR spectra of compound **c**

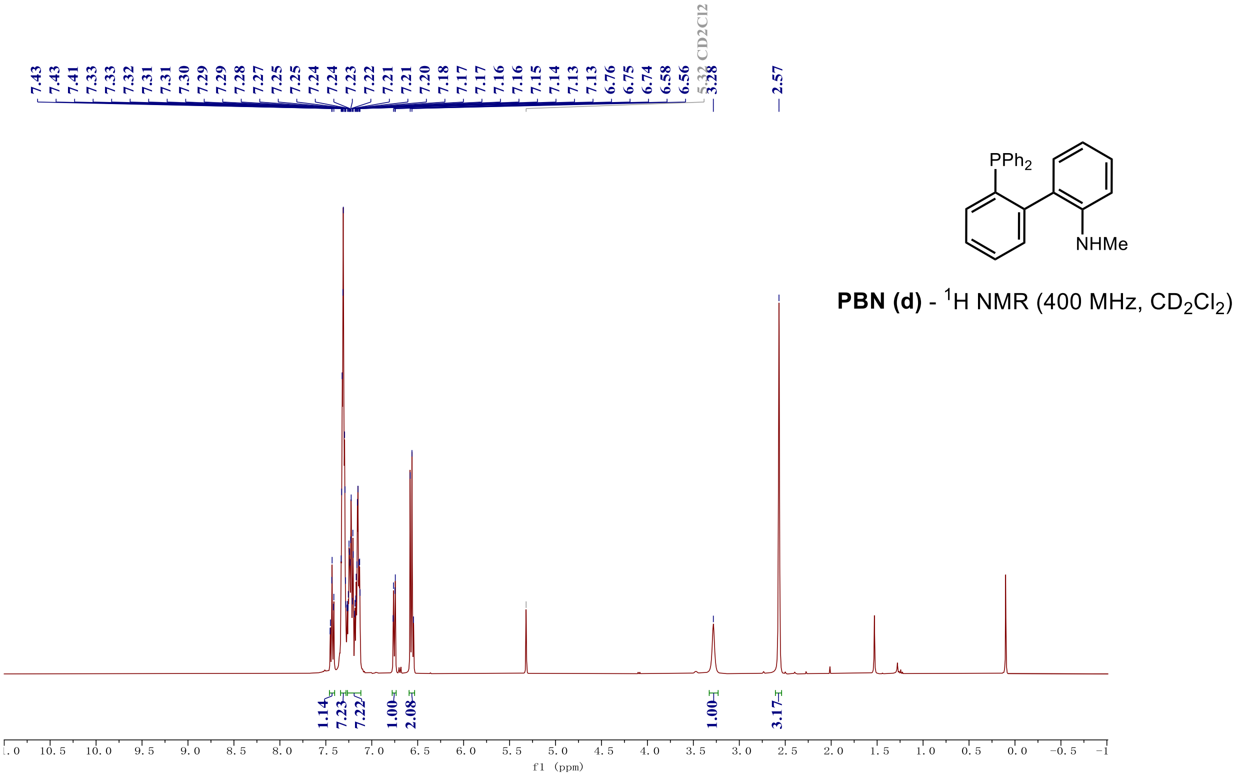

Supplementary Figure 23 ^{1}H NMR spectra of compound **d**

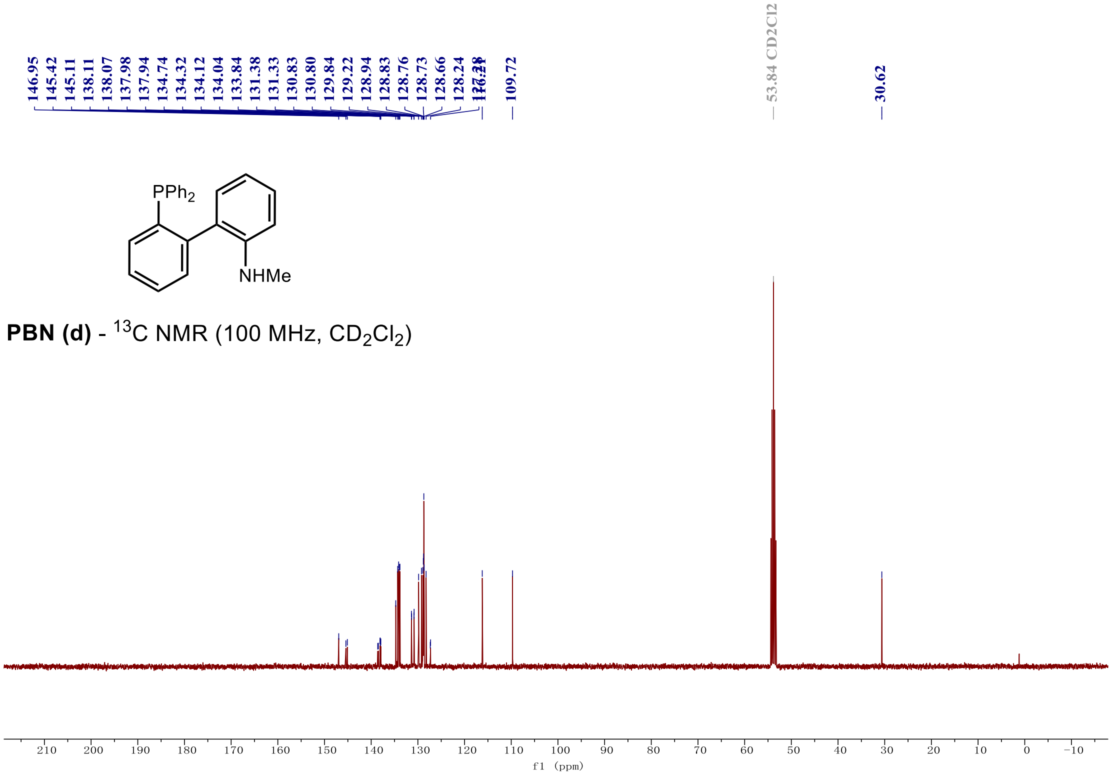

Supplementary Figure 24 ^{13}C NMR spectra of compound **d**

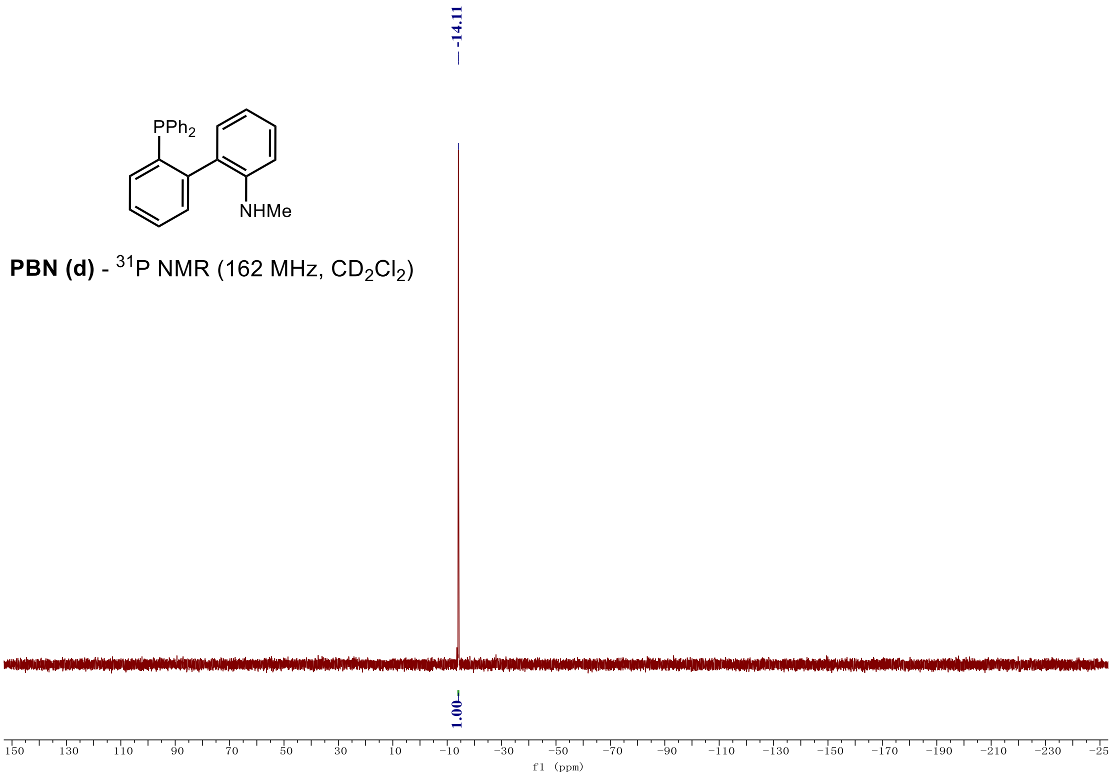

Supplementary Figure 25 ^{31}P NMR spectra of compound **d**

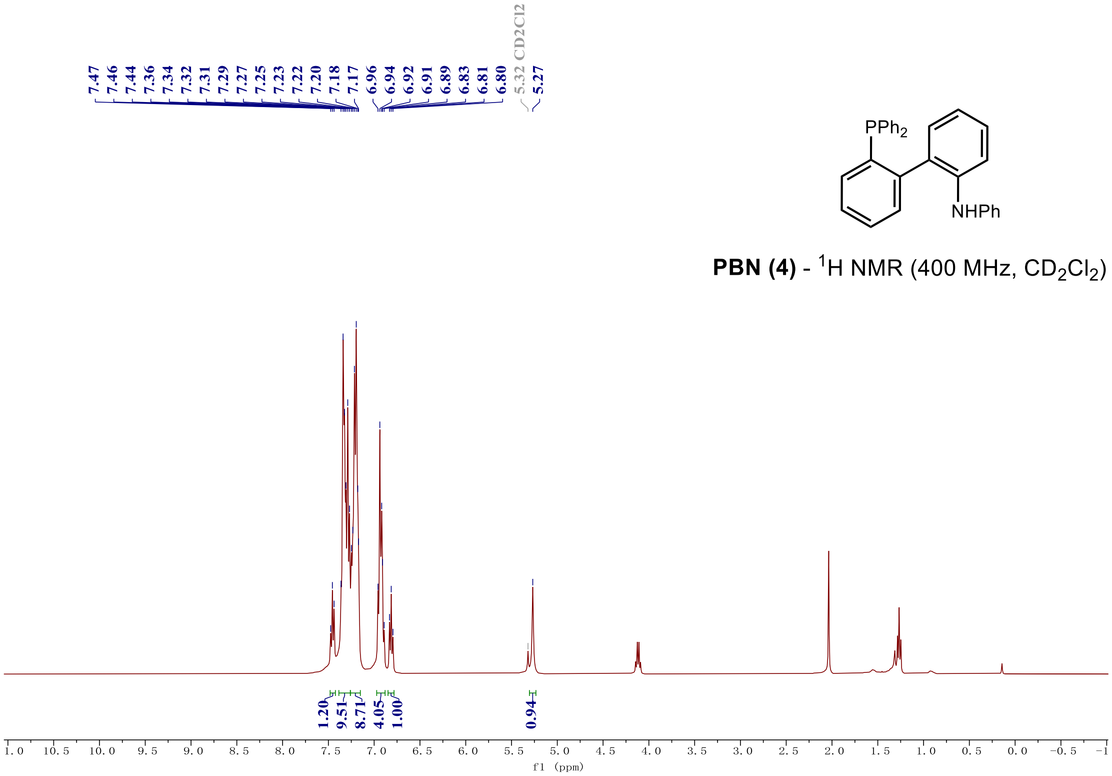

Supplementary Figure 26 ^{1}H NMR spectra of compound **4**

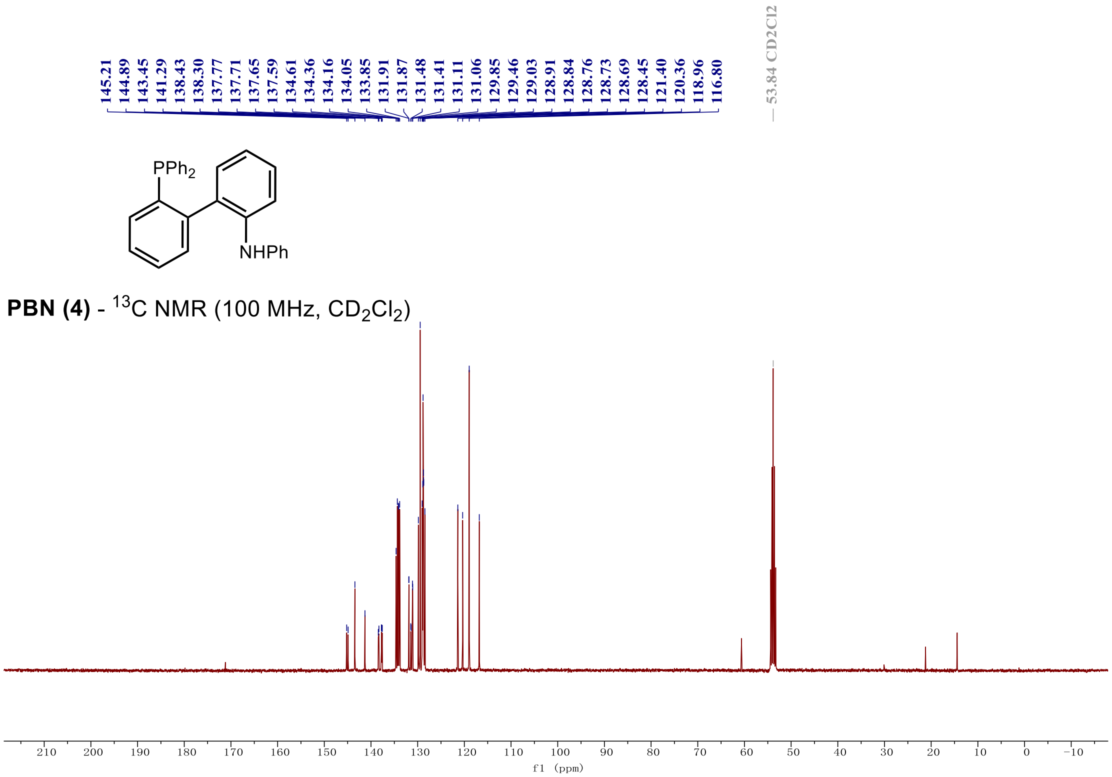

Supplementary Figure 27 ^{13}C NMR spectra of compound **4**

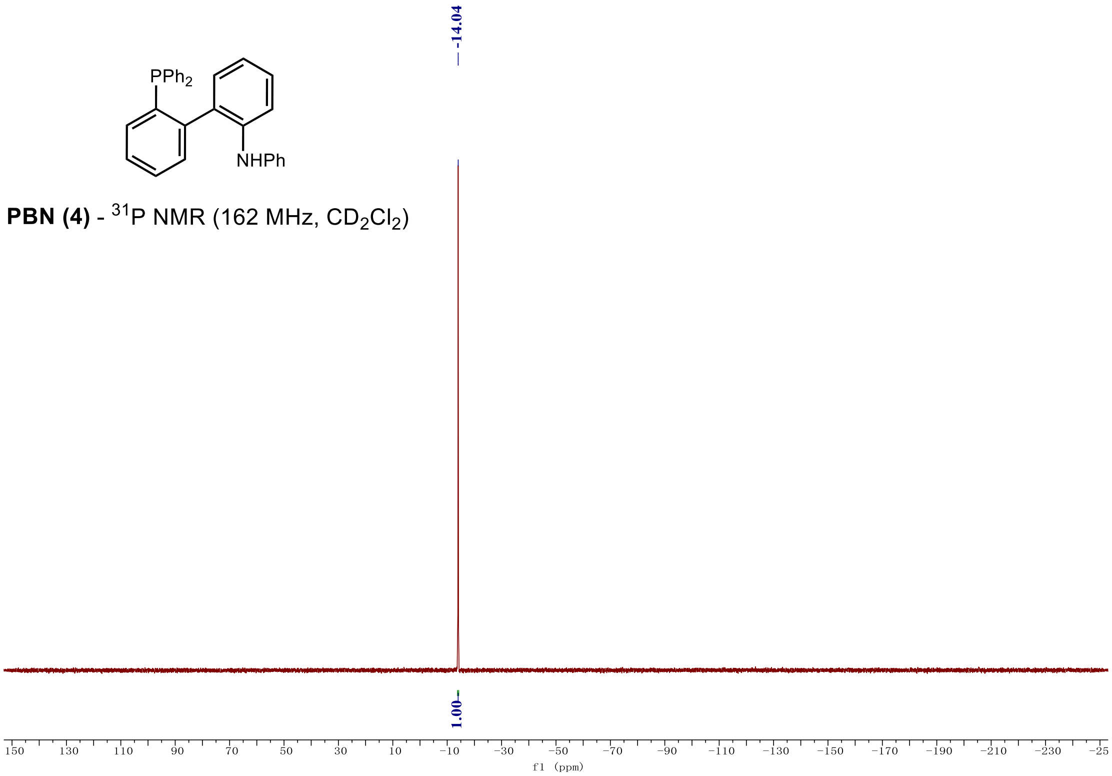

Supplementary Figure 28 ^{31}P NMR spectra of compound **4**

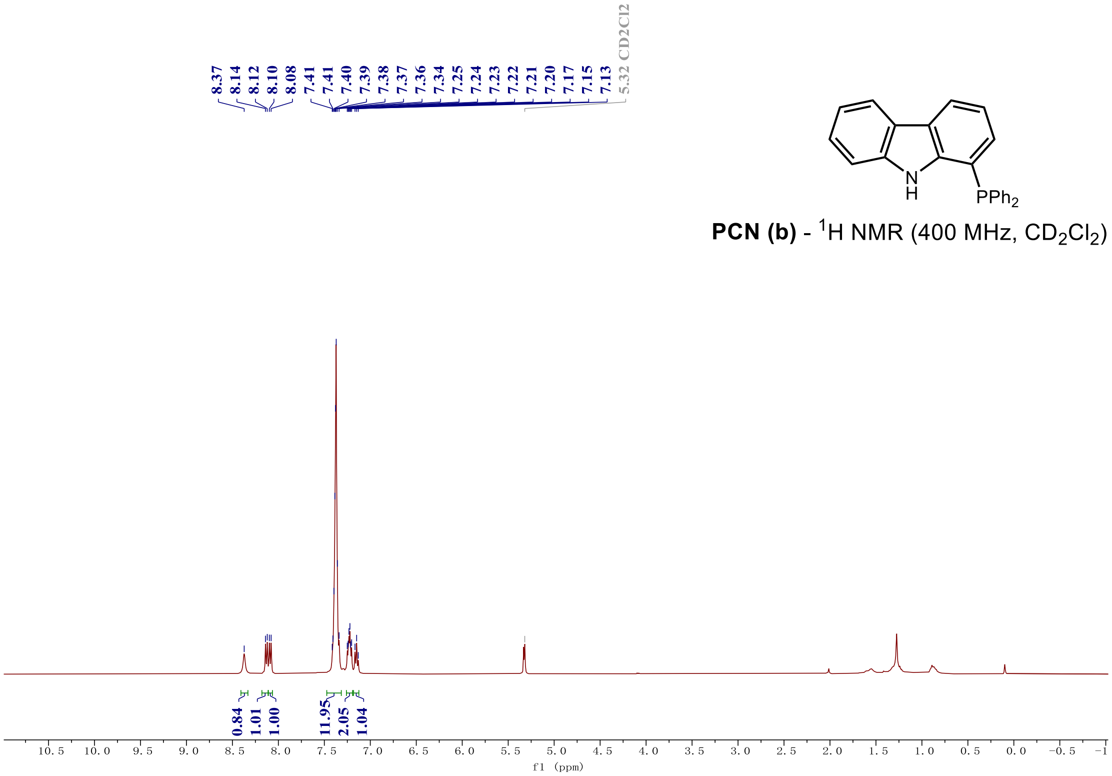

Supplementary Figure 29 ^{1}H NMR spectra of compound **b**

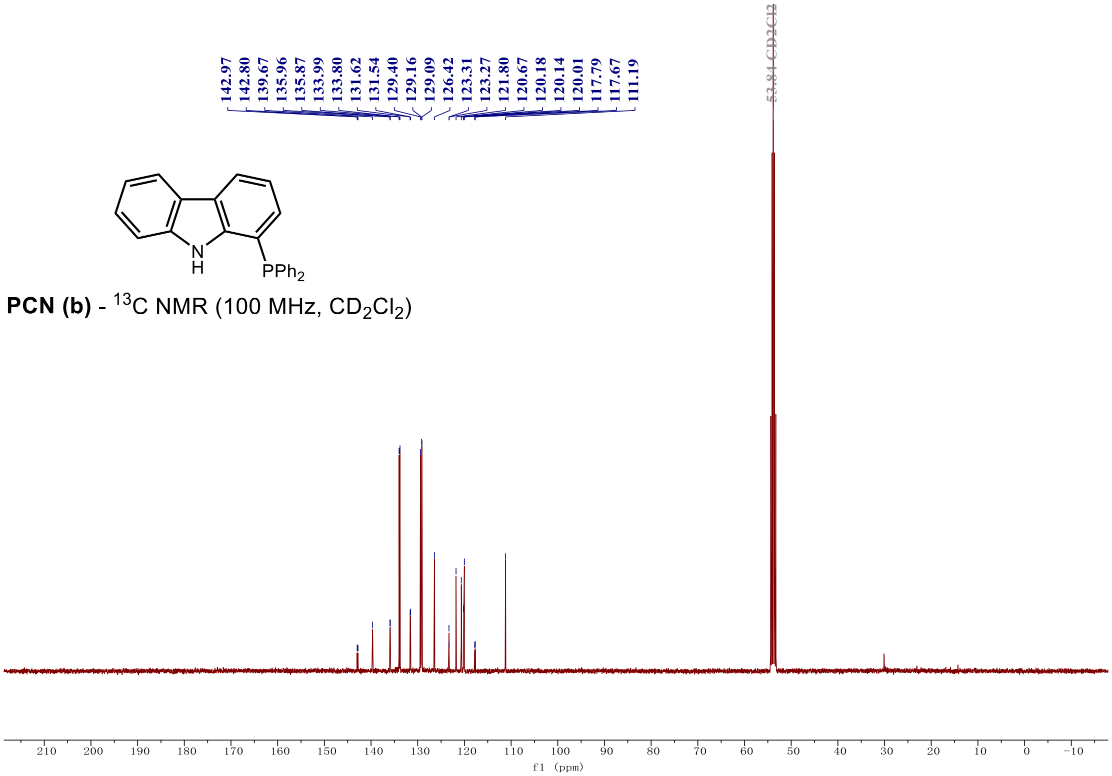

Supplementary Figure 30 ^{13}C NMR spectra of compound **b**

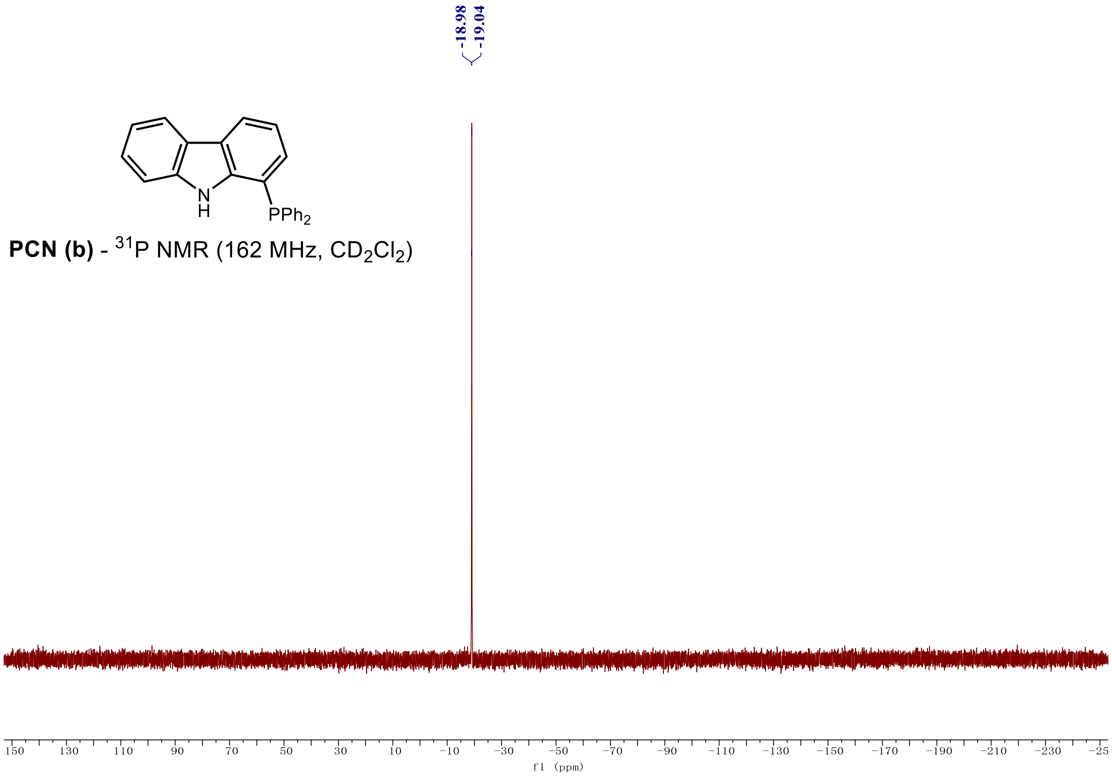

Supplementary Figure 31 ^{31}P NMR spectra of compound **b**

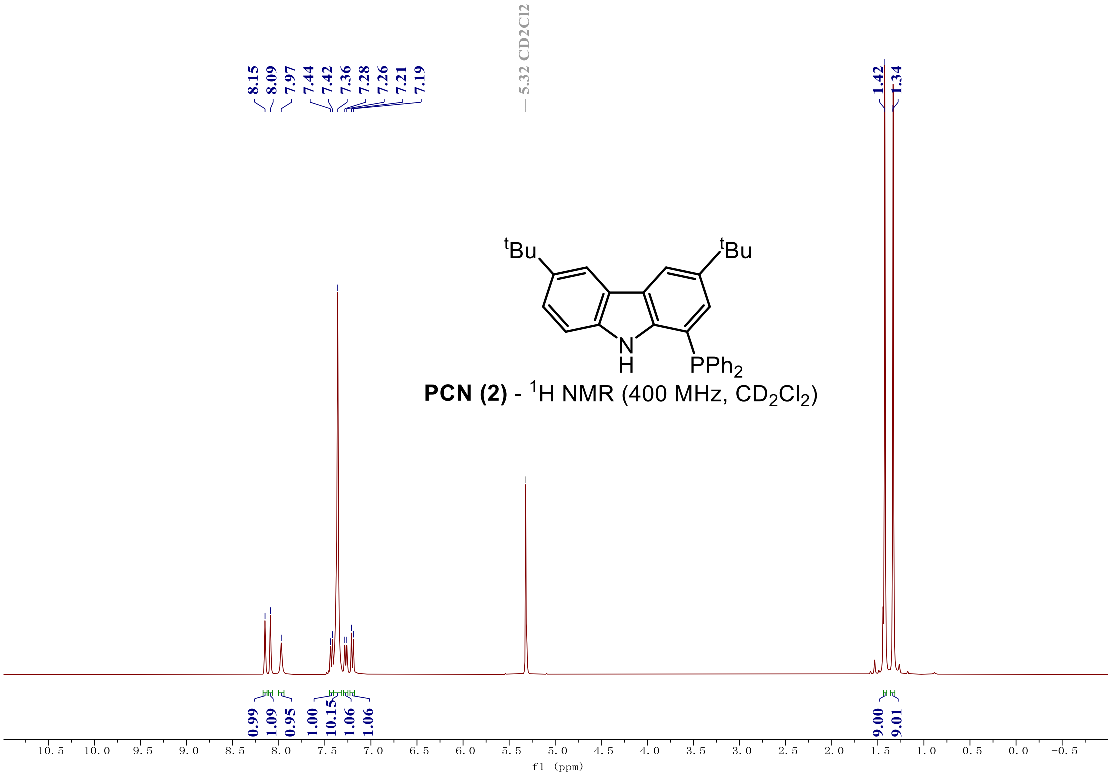

Supplementary Figure 32 ^{1}H NMR spectra of compound **2**

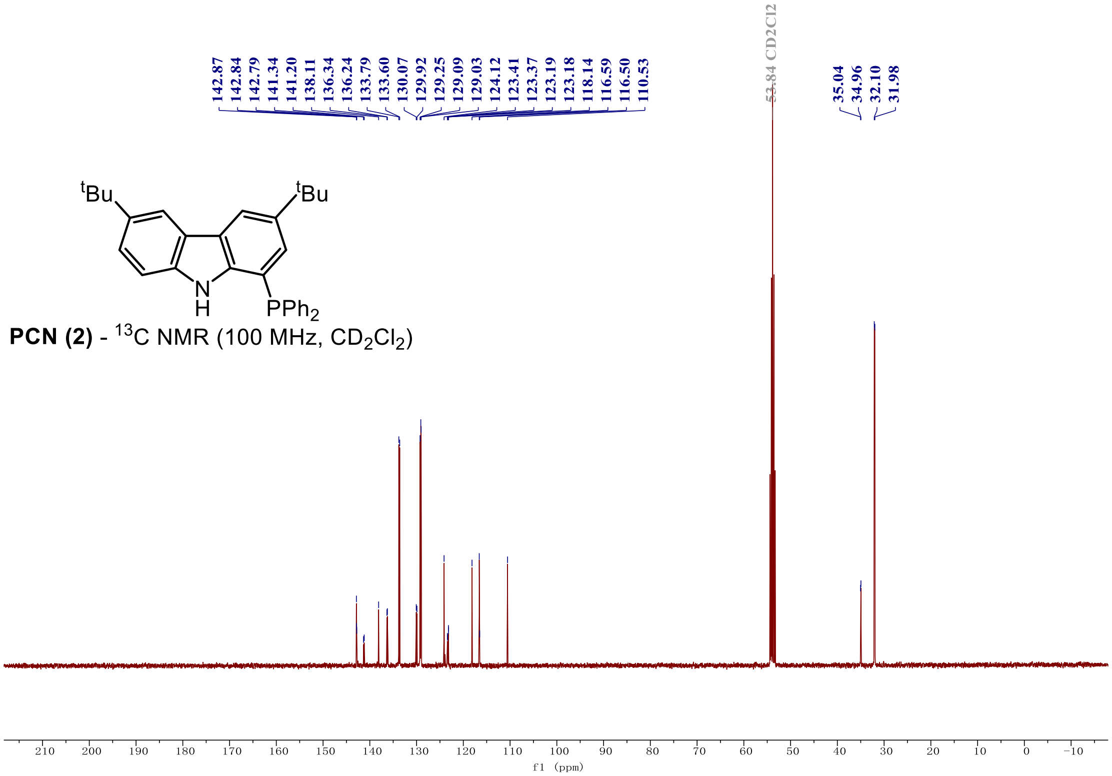

Supplementary Figure 33 ^{13}C NMR spectra of compound **2**

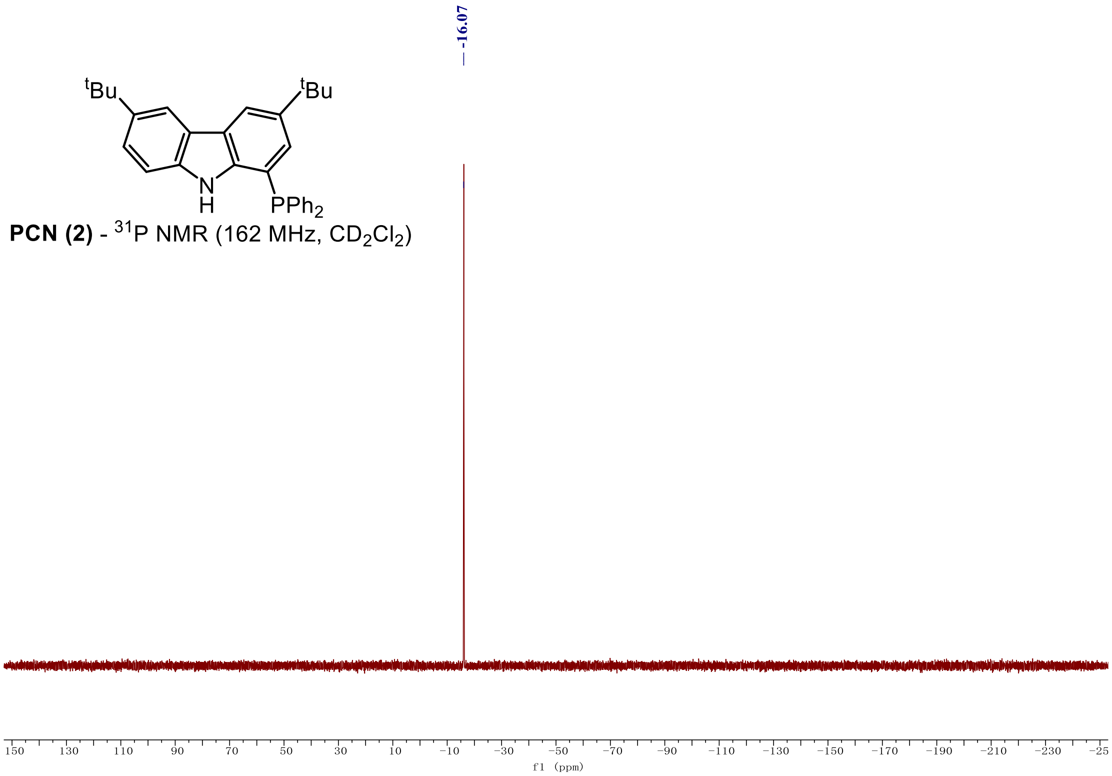

Supplementary Figure 34 ^{31}P NMR spectra of compound **2**

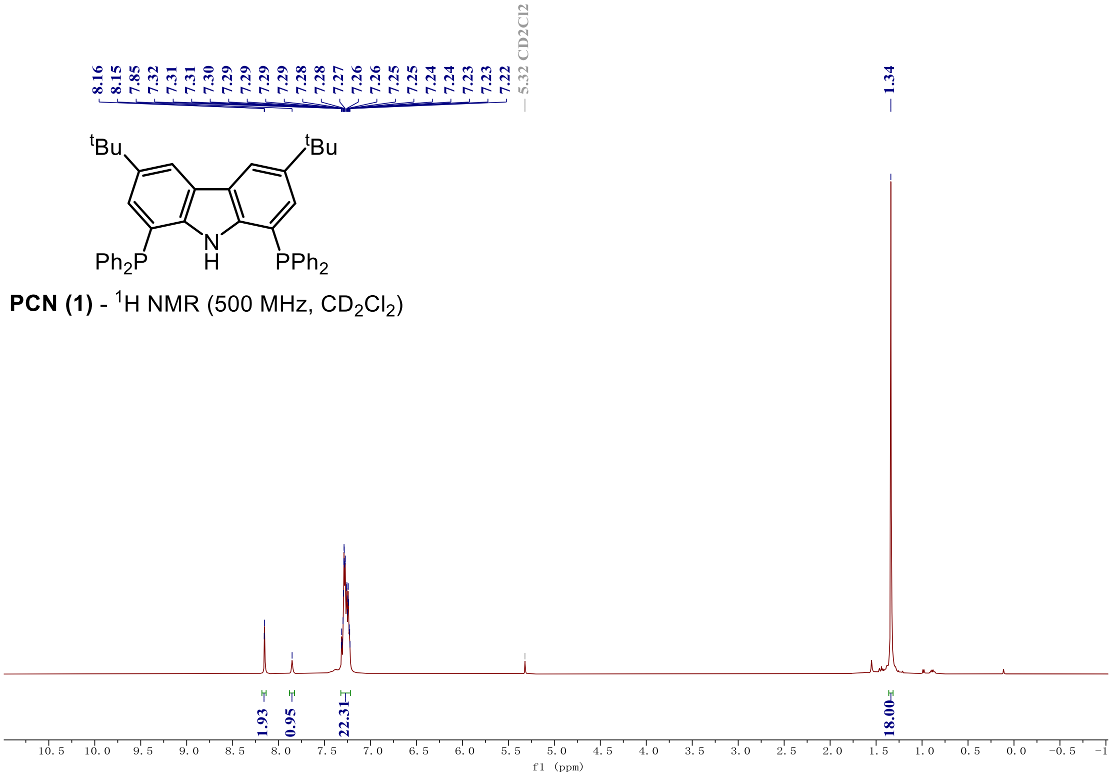

Supplementary Figure 35 ^{1}H NMR spectra of compound **1**

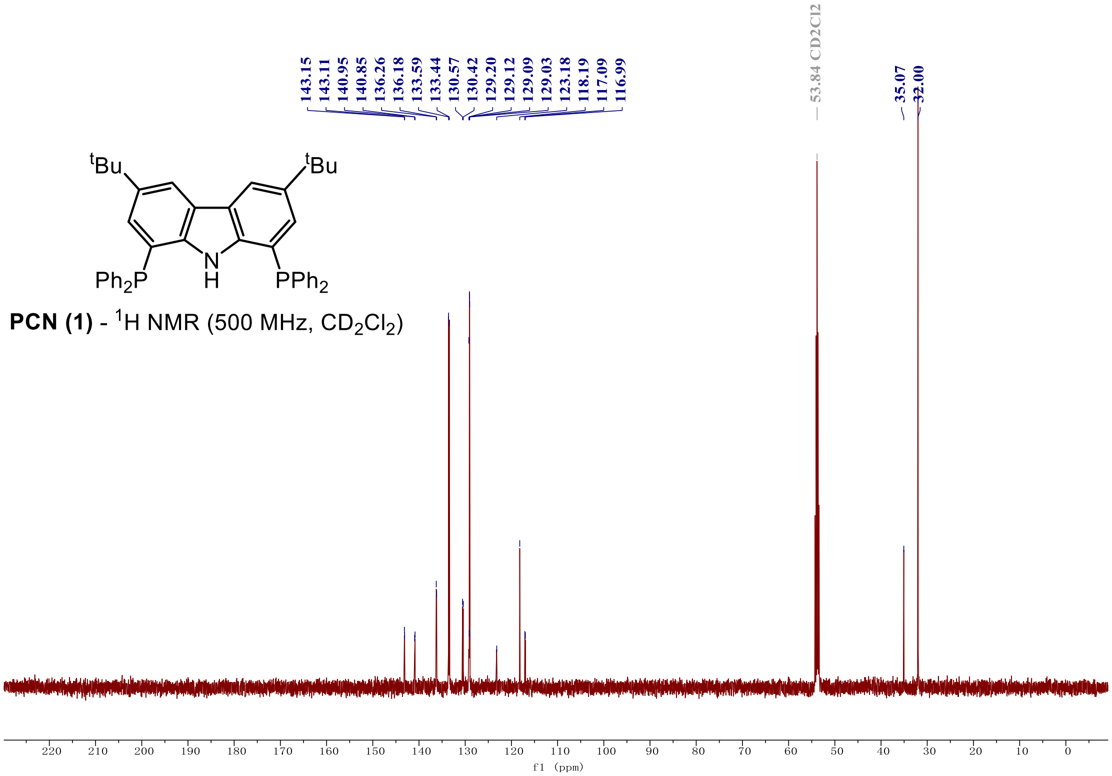

Supplementary Figure 36 ^{13}C NMR spectra of compound **1**

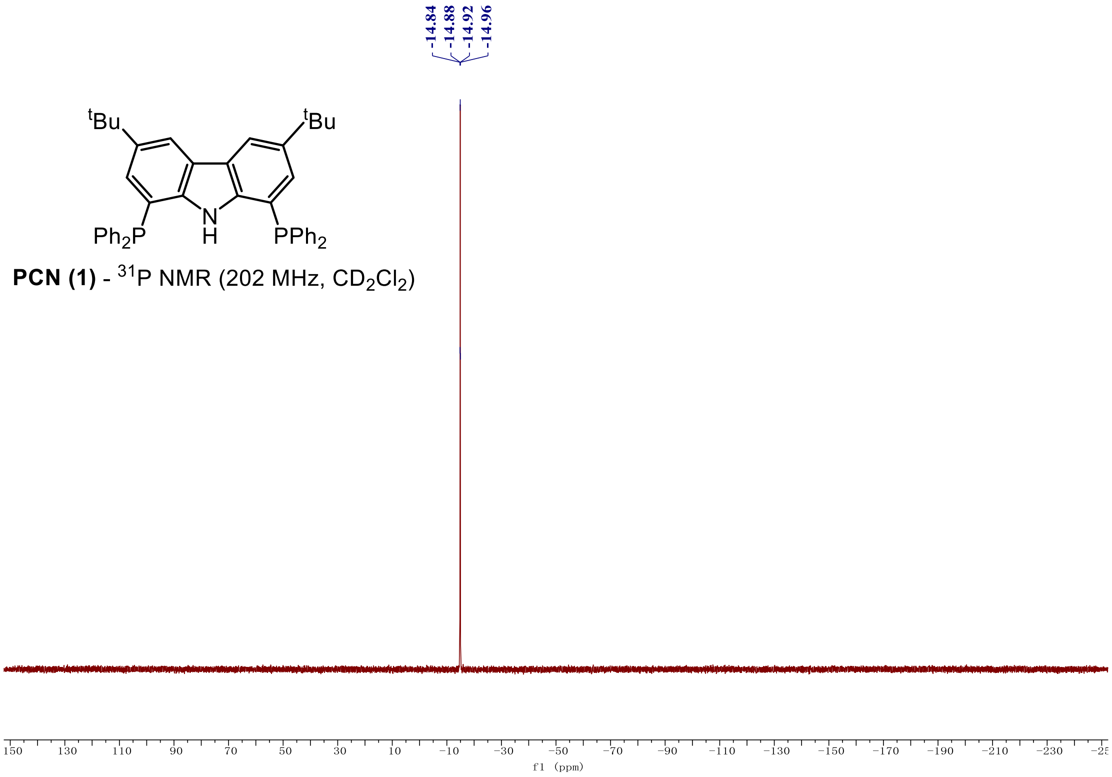

Supplementary Figure 37 ^{31}P NMR spectra of compound **1**

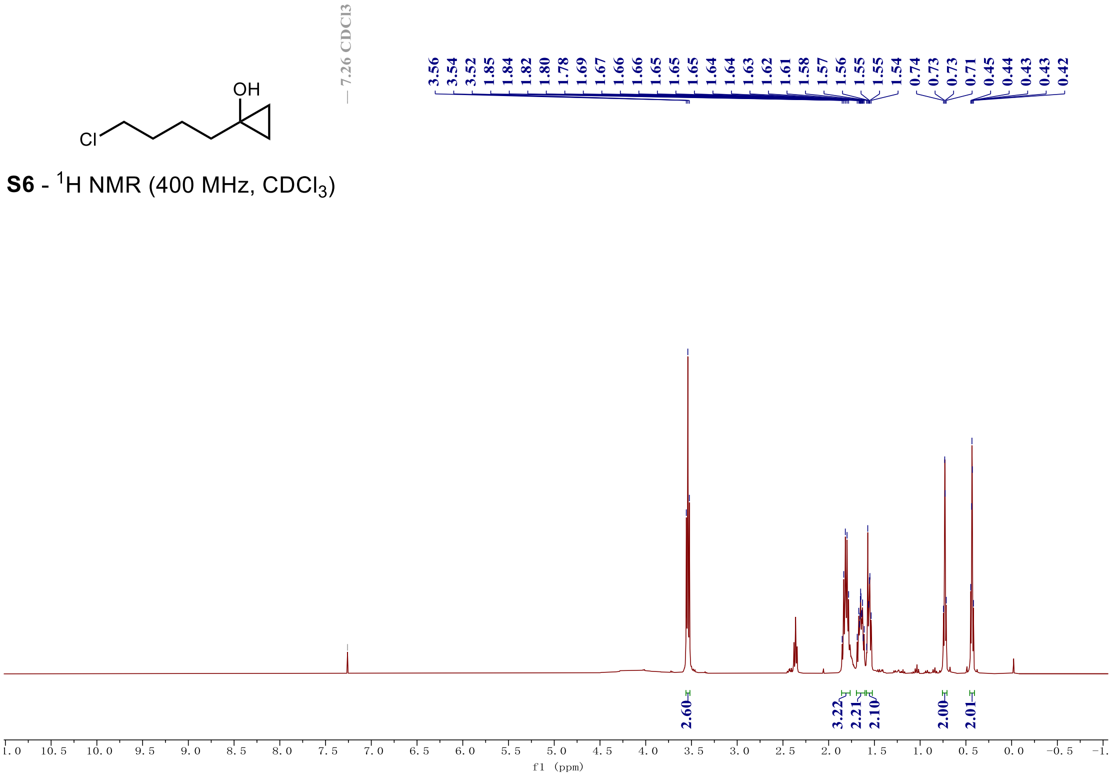

Supplementary Figure 38 ^{1}H NMR spectra of compound **S6**

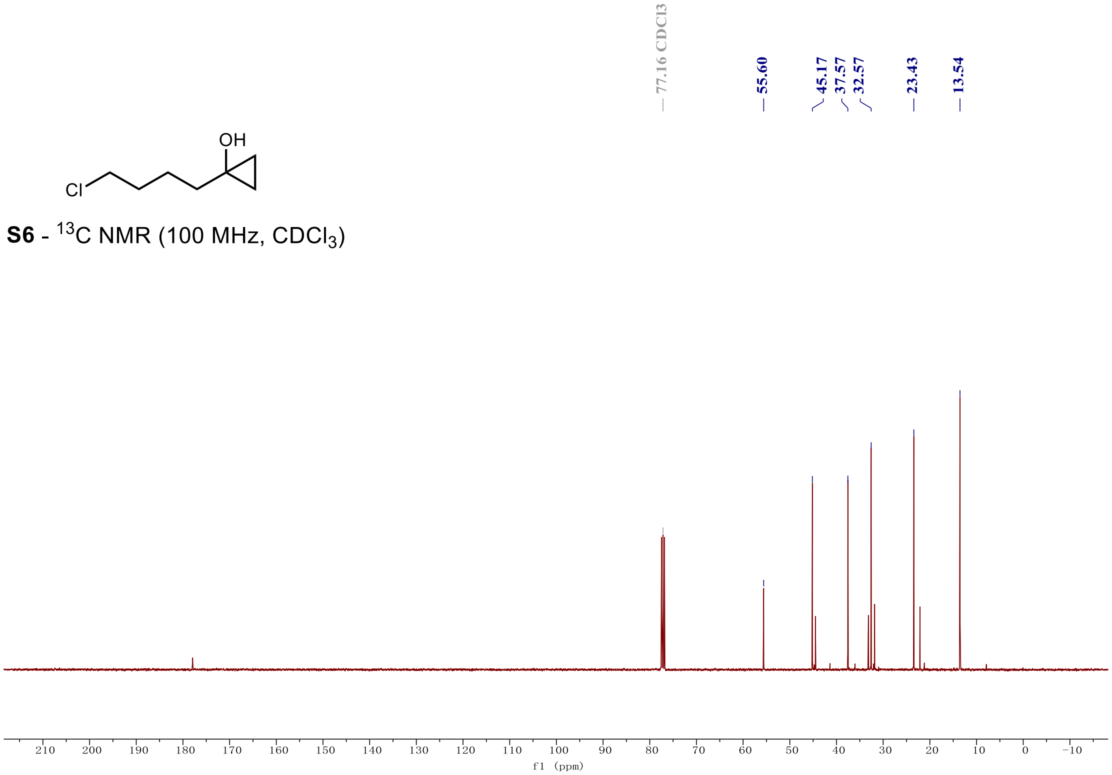

Supplementary Figure 39 ^{13}C NMR spectra of compound **S6**

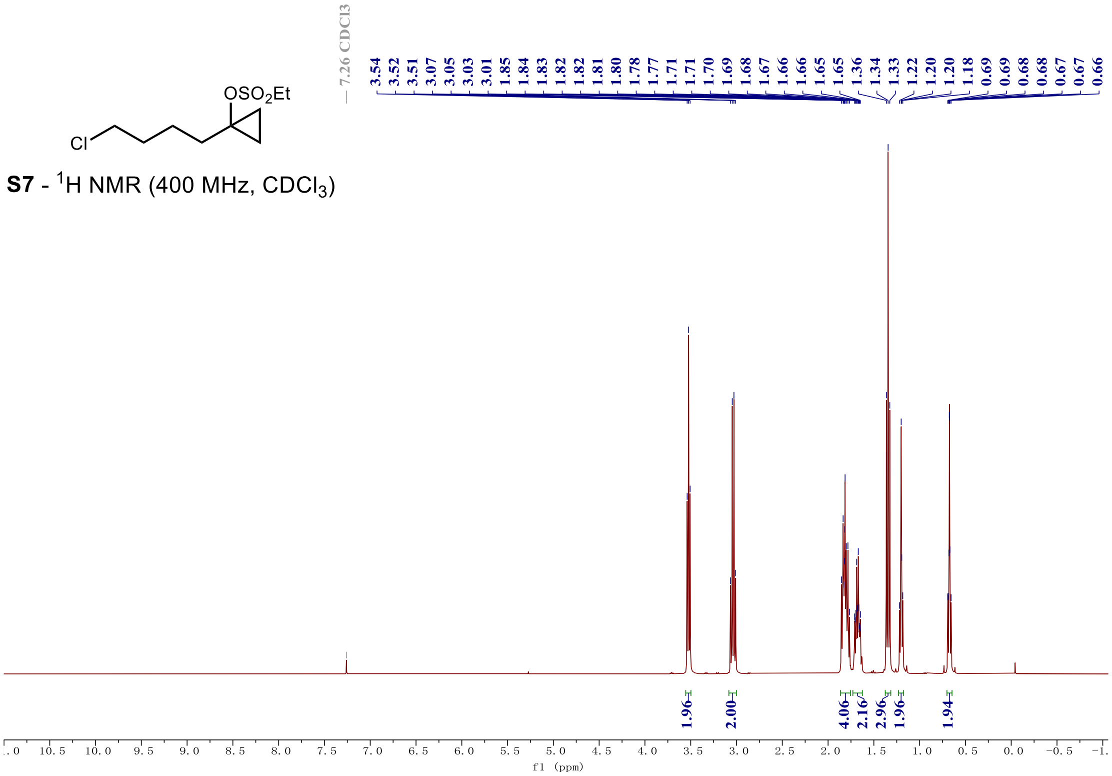

Supplementary Figure 40 ^{1}H NMR spectra of compound **S7**

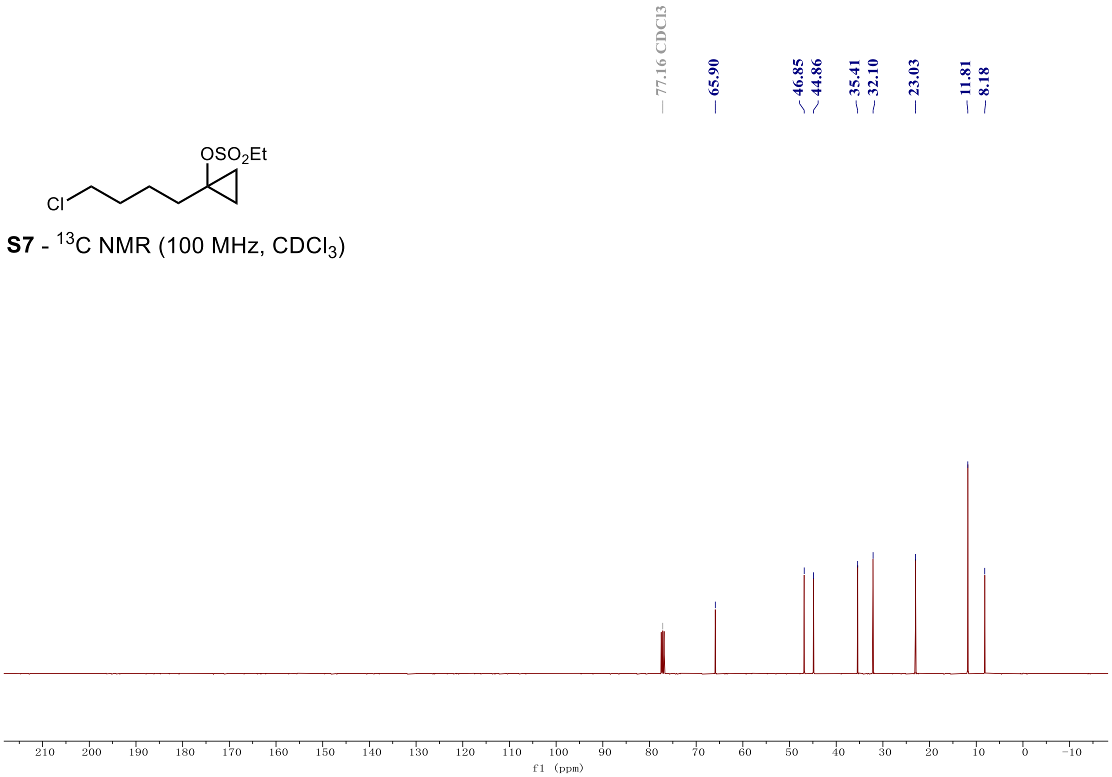

Supplementary Figure 41 ^{13}C NMR spectra of compound **S7**

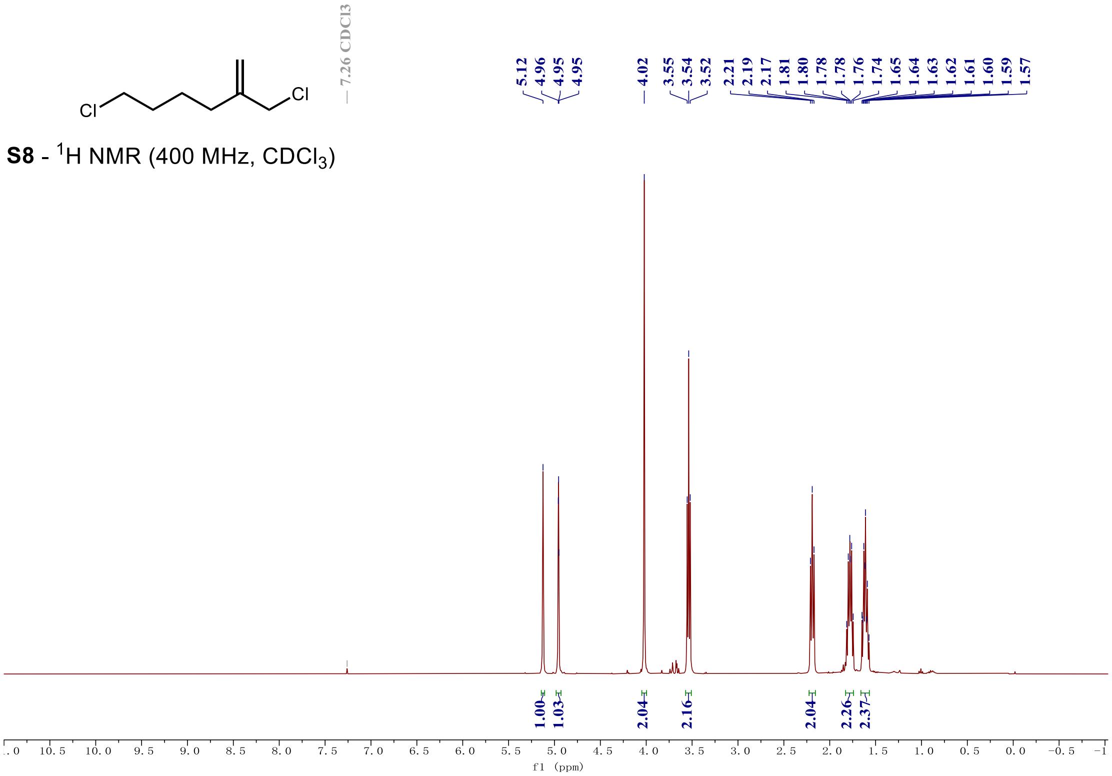

Supplementary Figure 42 ^{1}H NMR spectra of compound **S8**

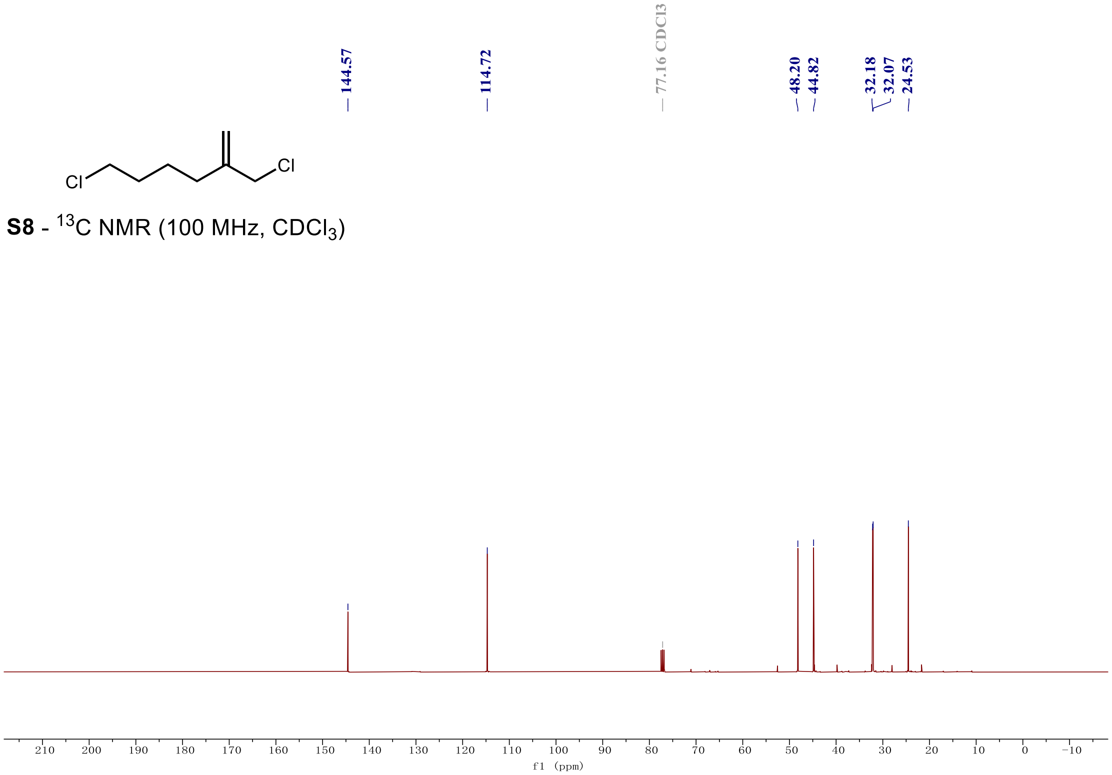

Supplementary Figure 43 ^{13}C NMR spectra of compound **S8**

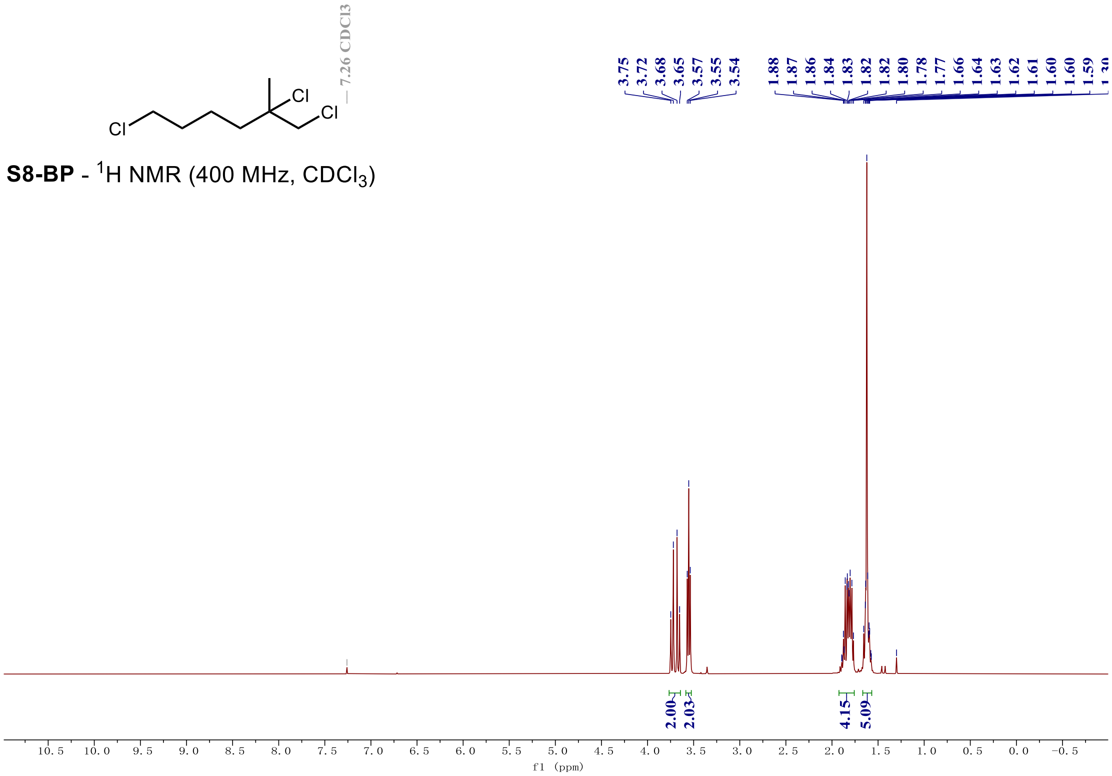

Supplementary Figure 44 ^{1}H NMR spectra of compound **S8-BP**

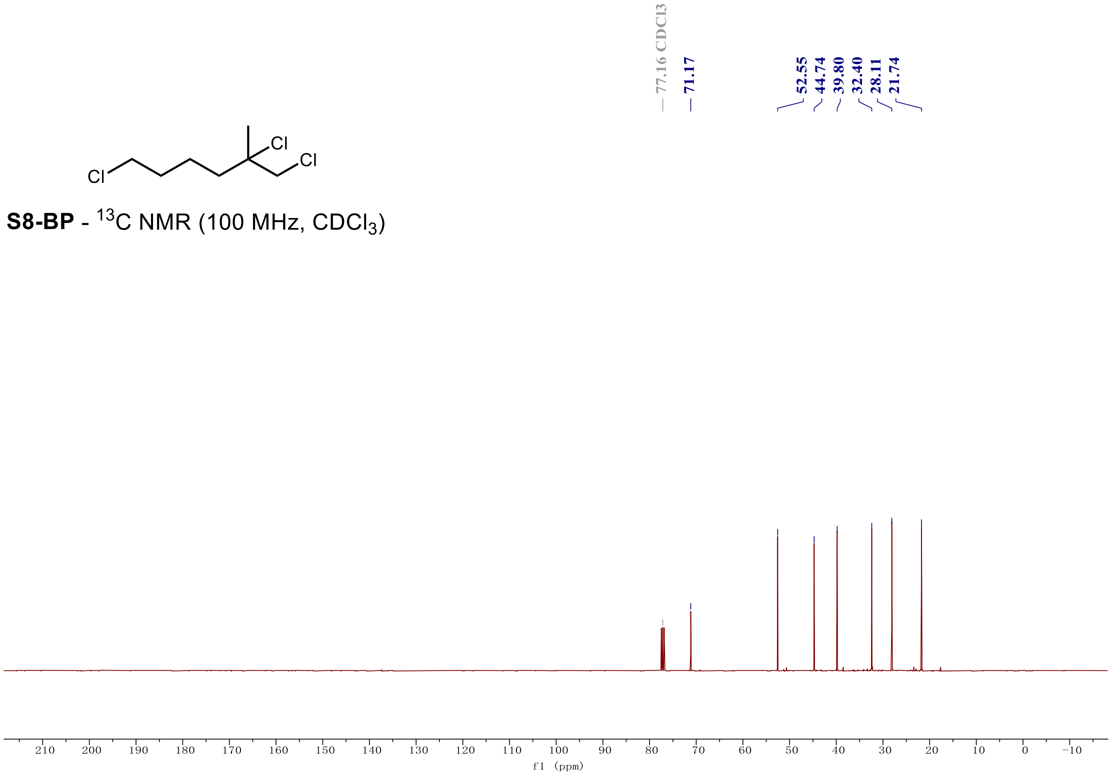

Supplementary Figure 45 ^{13}C NMR spectra of compound **S8-BP**

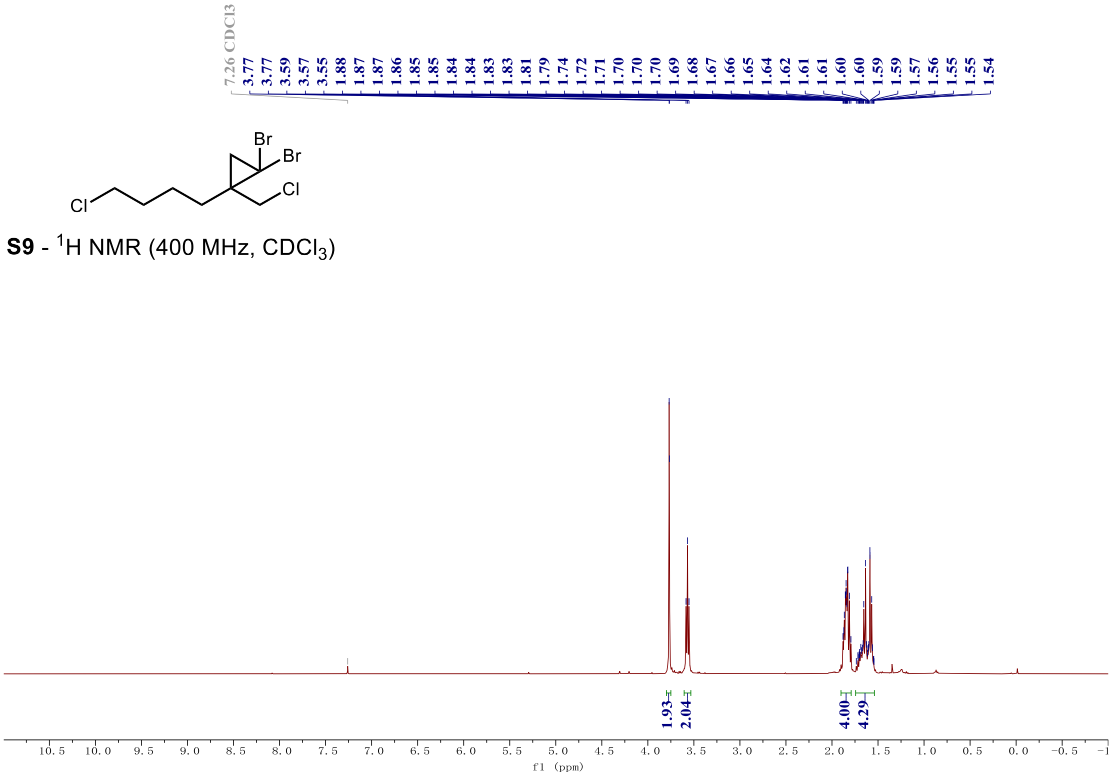

Supplementary Figure 46 ^{1}H NMR spectra of compound **S9**

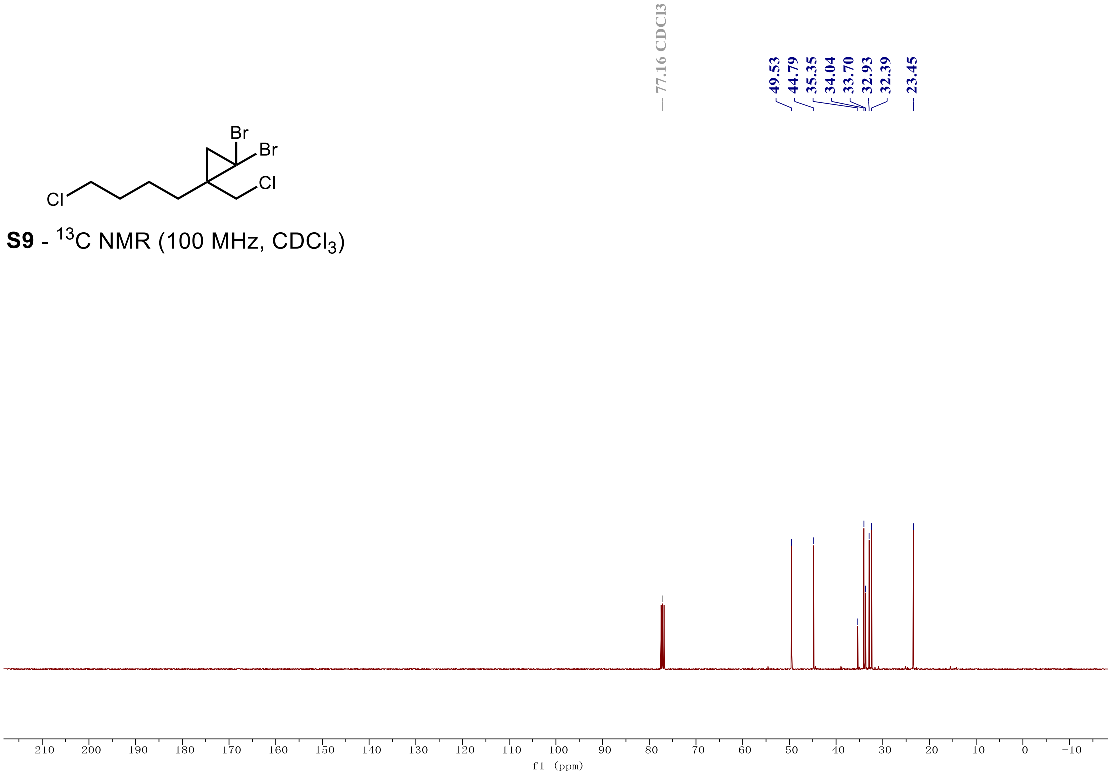

Supplementary Figure 47 ^{13}C NMR spectra of compound **S9**

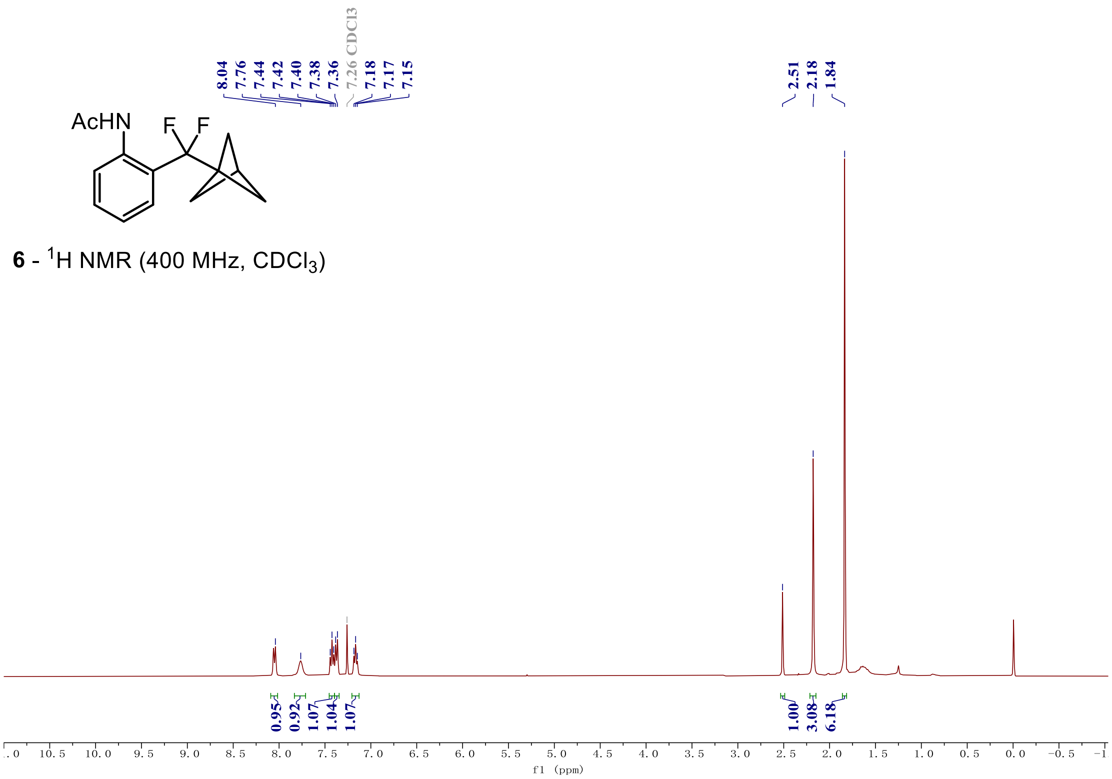

Supplementary Figure 48 ^{1}H NMR spectra of compound **6**

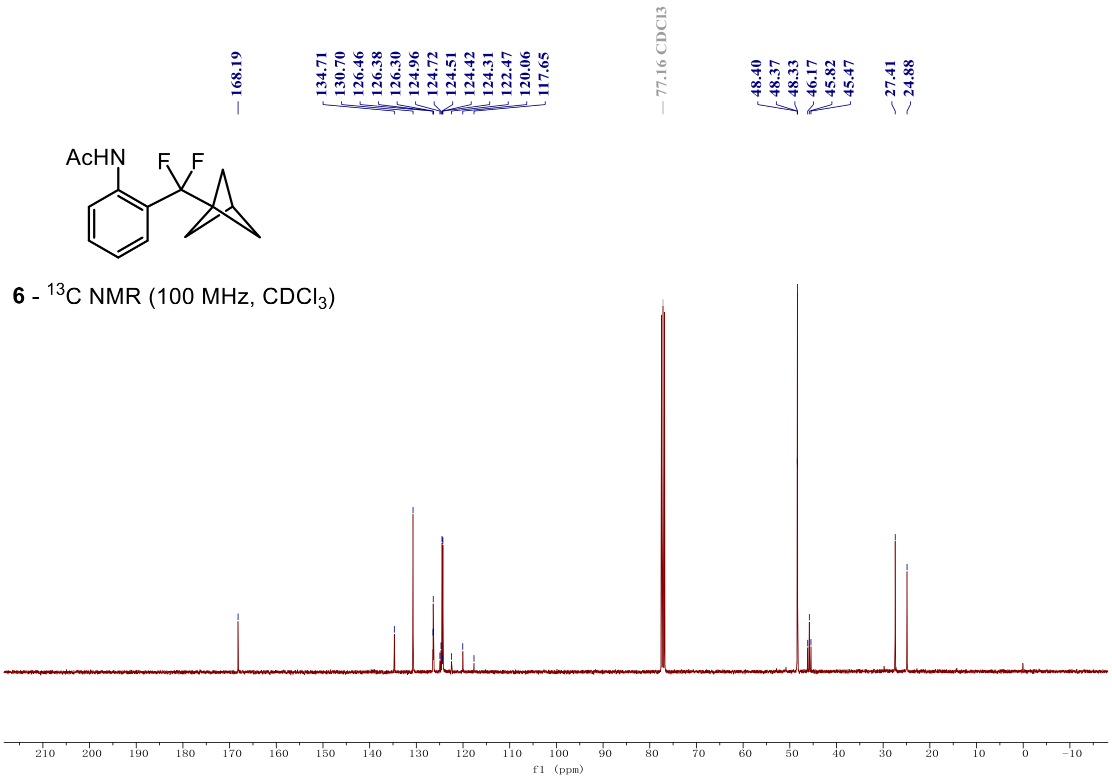

Supplementary Figure 49 ^{13}C NMR spectra of compound **6**

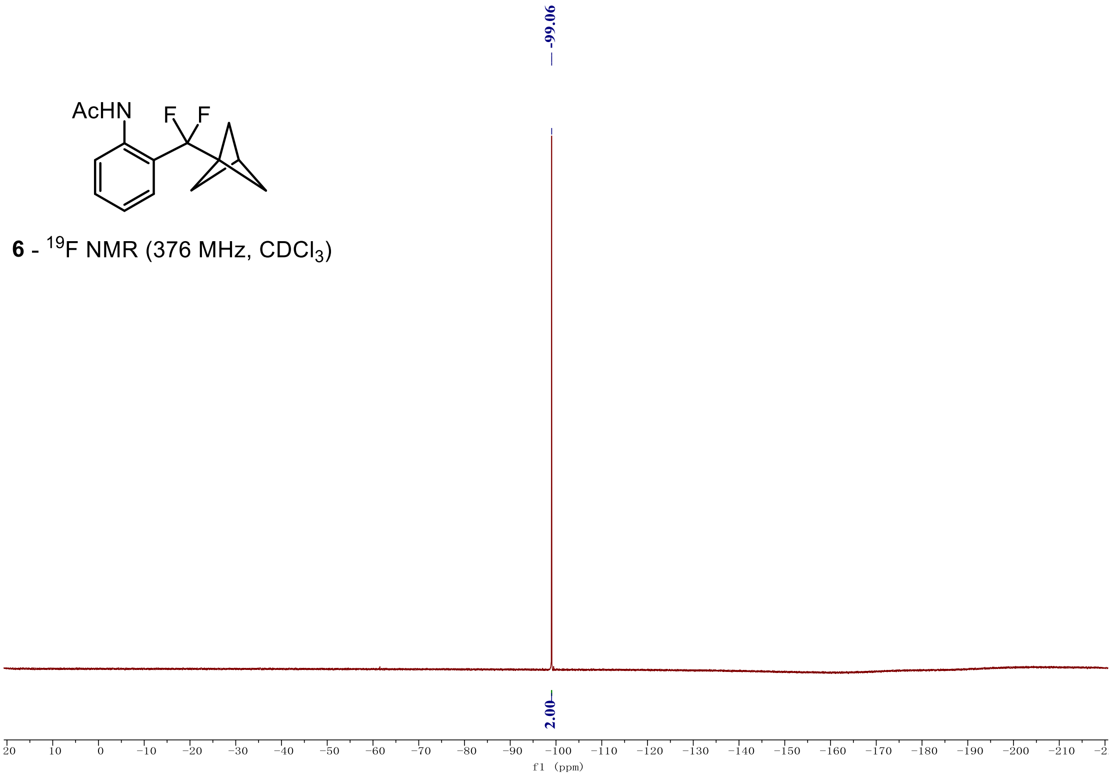

Supplementary Figure 50 ^{19}F NMR spectra of compound **6**

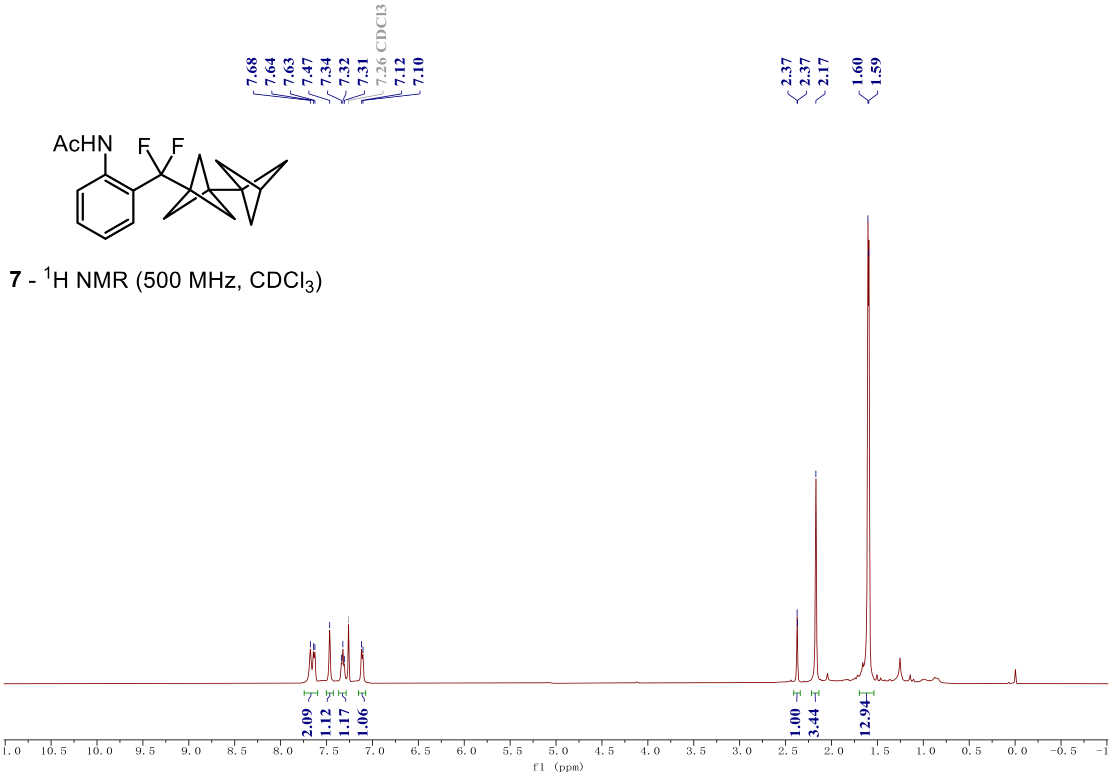

Supplementary Figure 51 ^{1}H NMR spectra of compound **7**

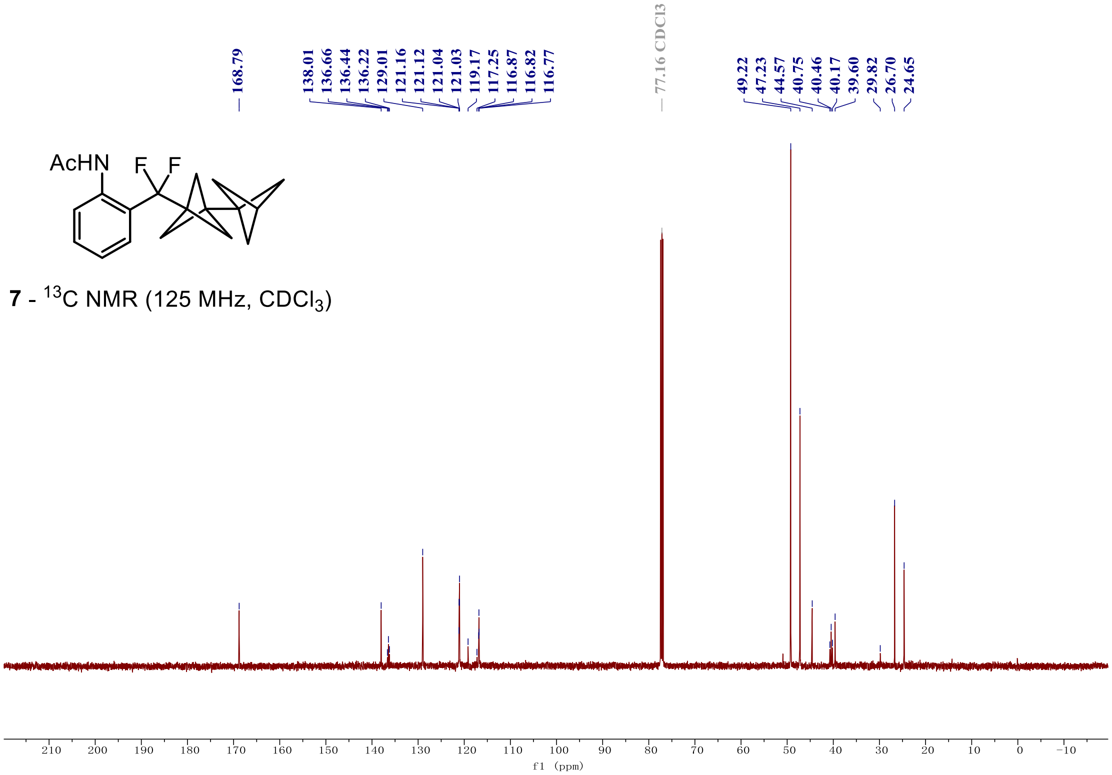

Supplementary Figure 52 ^{13}C NMR spectra of compound **7**

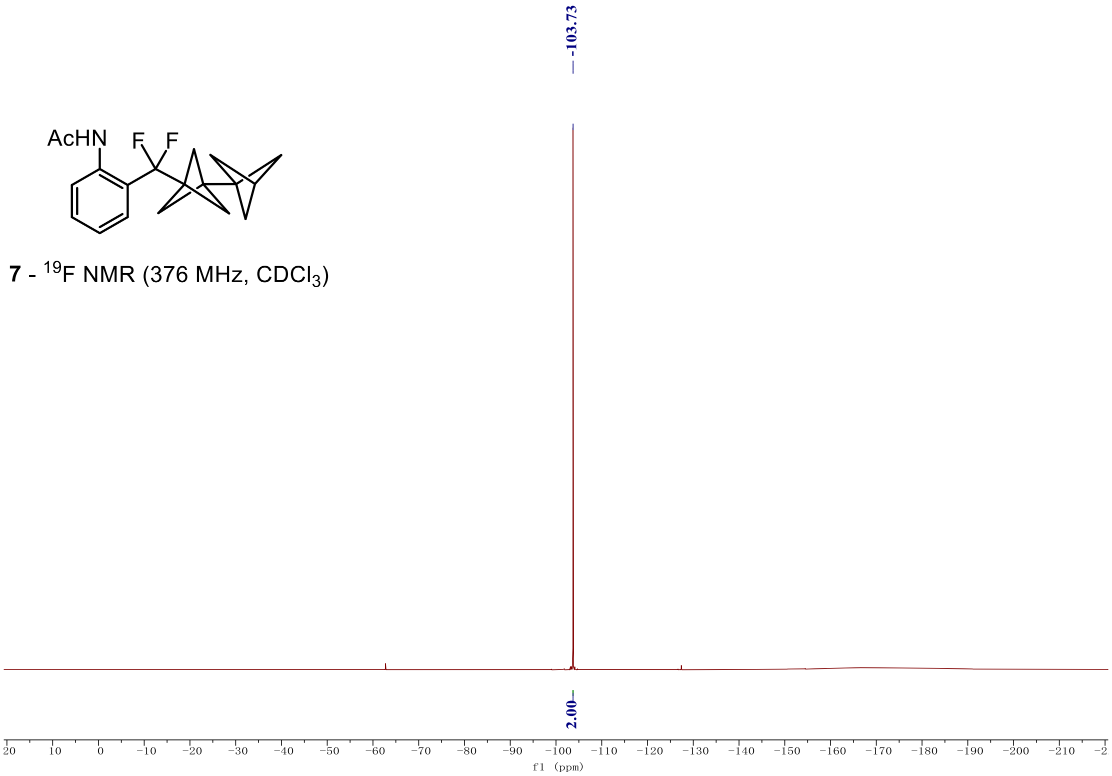

Supplementary Figure 53 ^{19}F NMR spectra of compound **7**

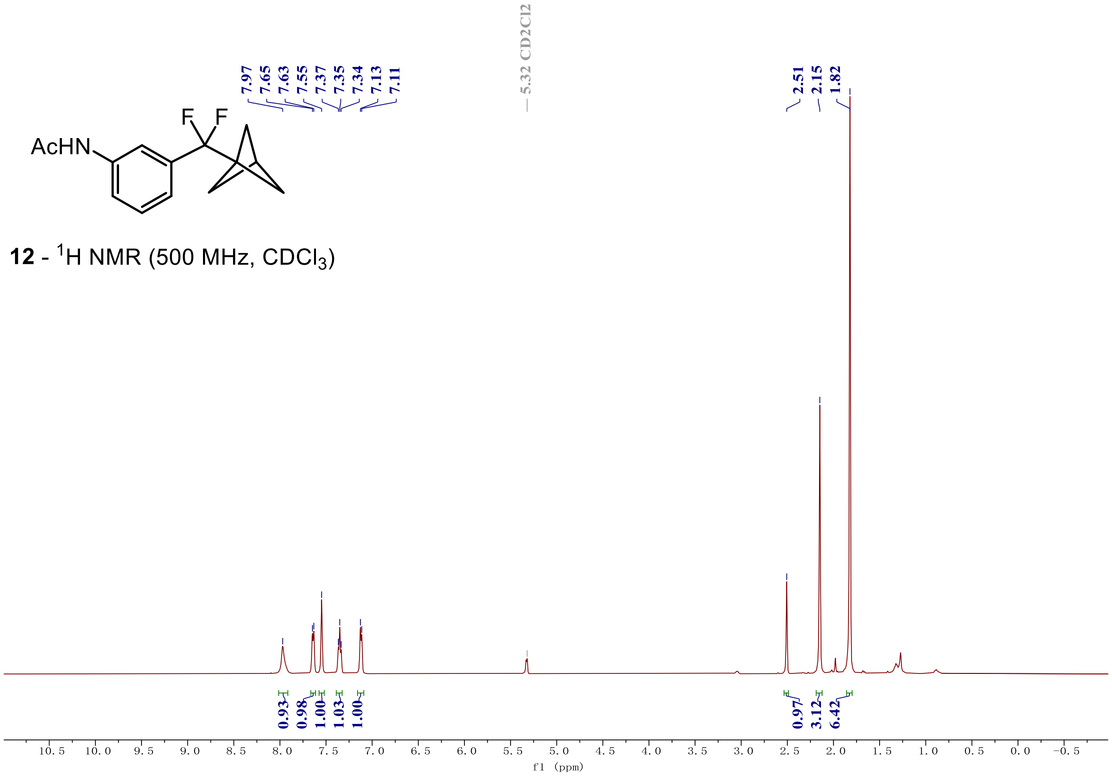

Supplementary Figure 54 ^{1}H NMR spectra of compound **12**

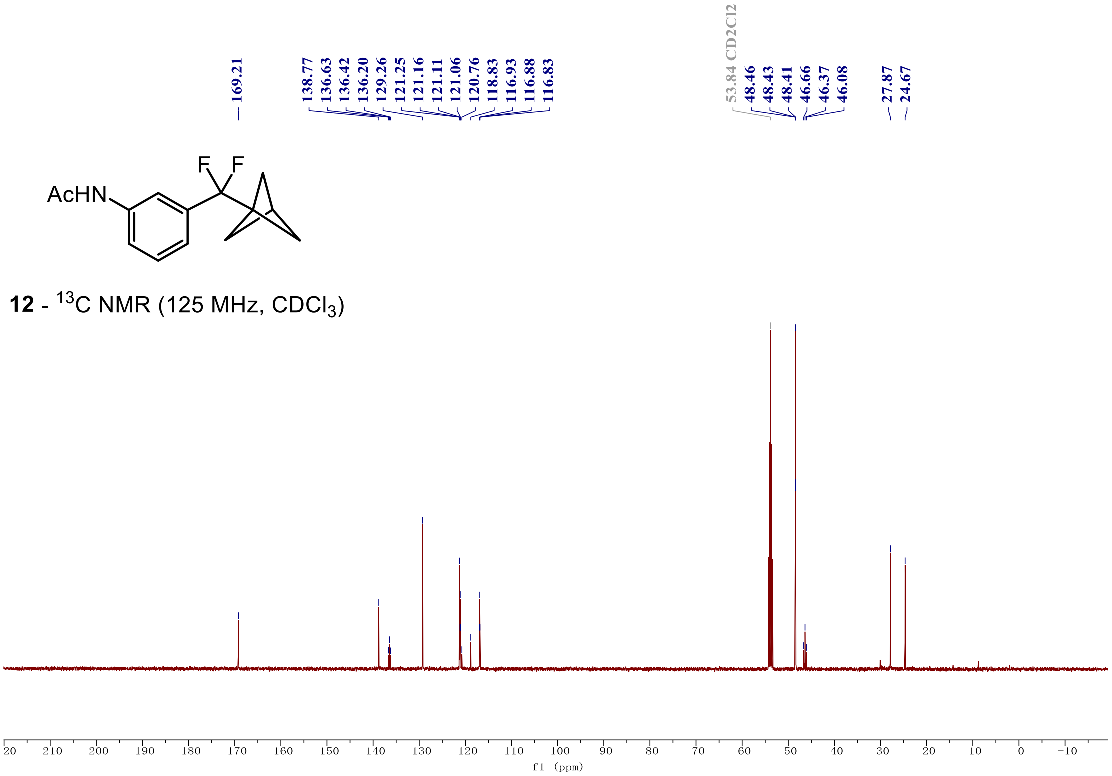

Supplementary Figure 55 ^{13}C NMR spectra of compound **12**

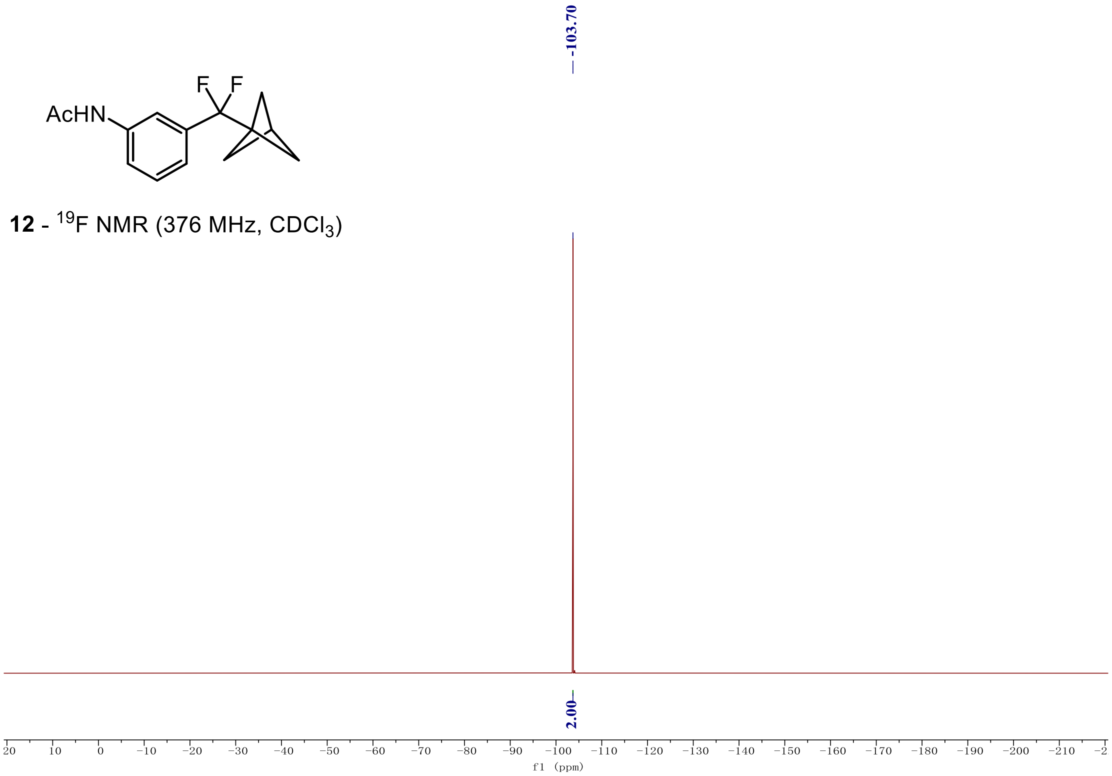

Supplementary Figure 56 ^{19}F NMR spectra of compound **12**

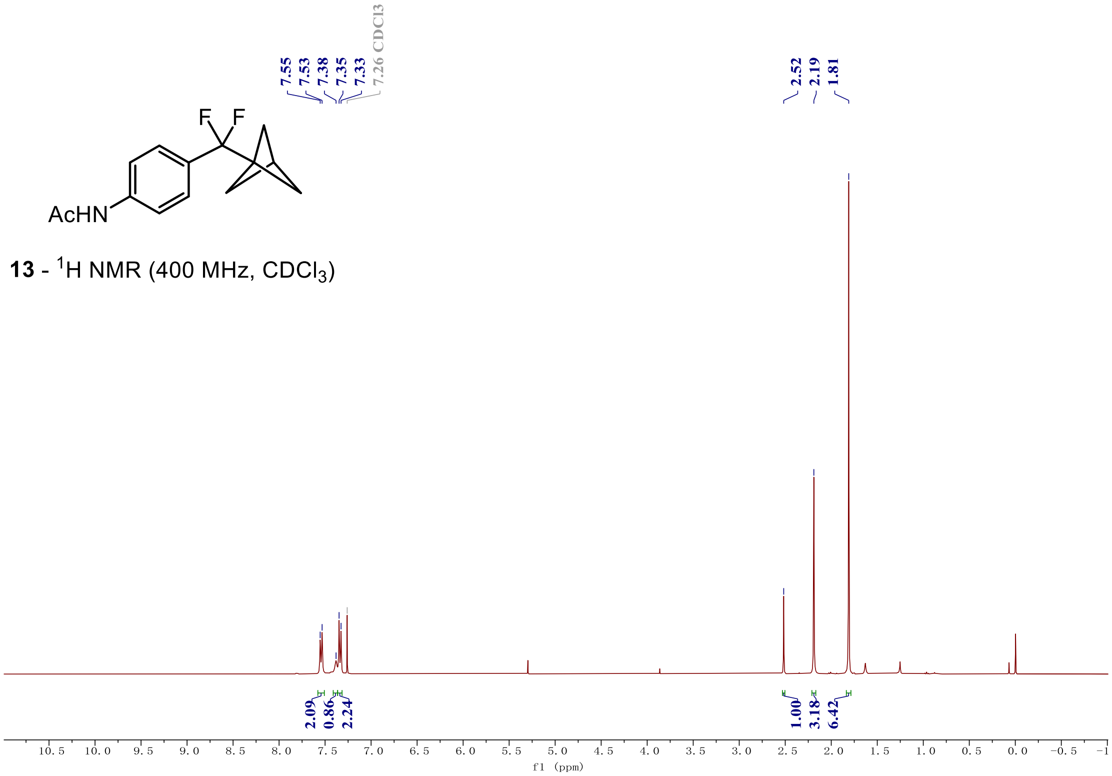

Supplementary Figure 57 ^{1}H NMR spectra of compound **13**

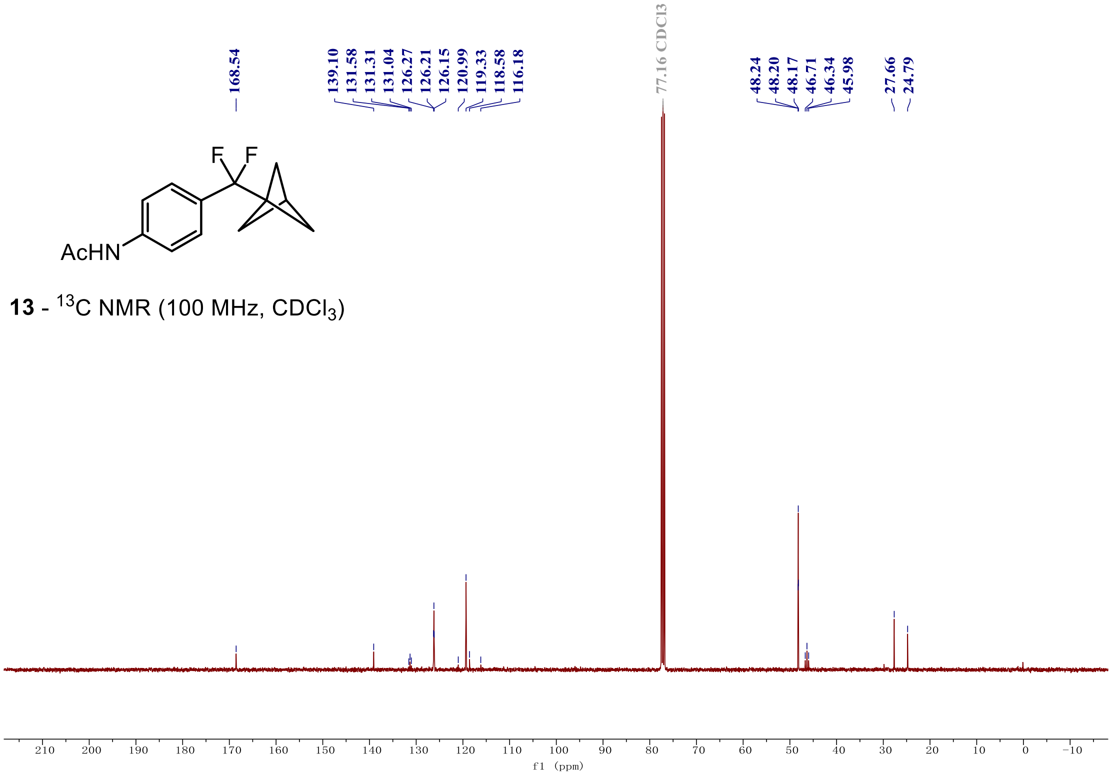

Supplementary Figure 58 ^{13}C NMR spectra of compound **13**

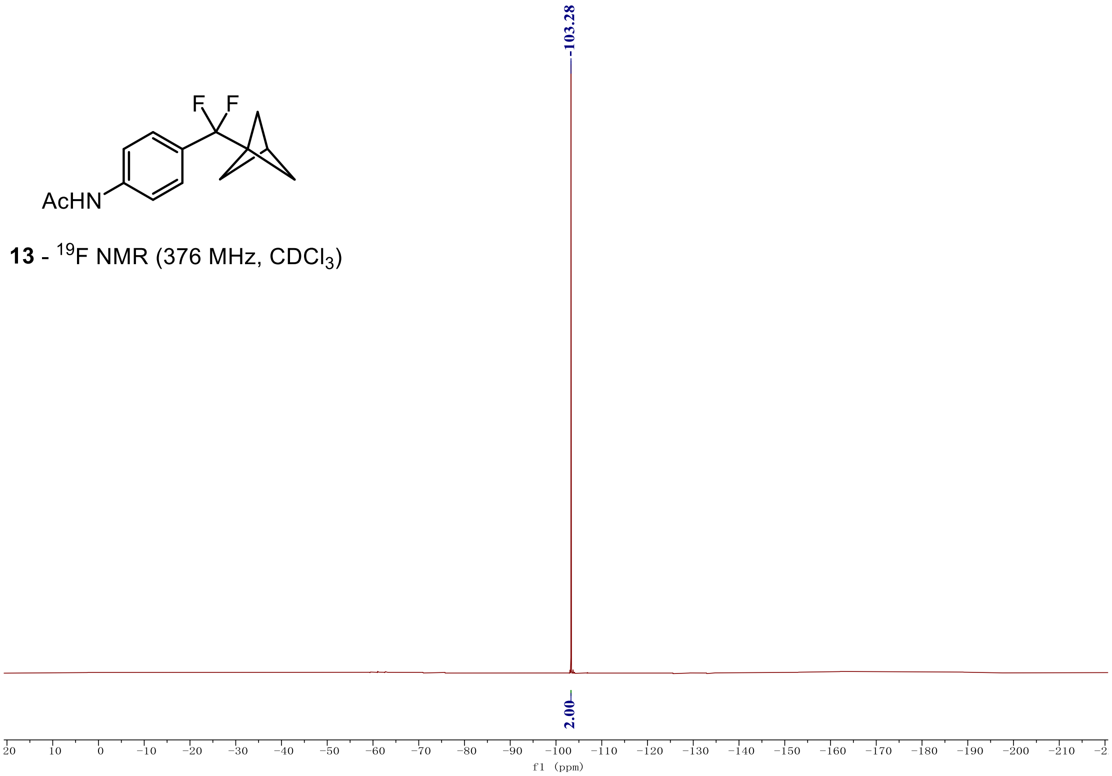

Supplementary Figure 59 ^{19}F NMR spectra of compound **13**

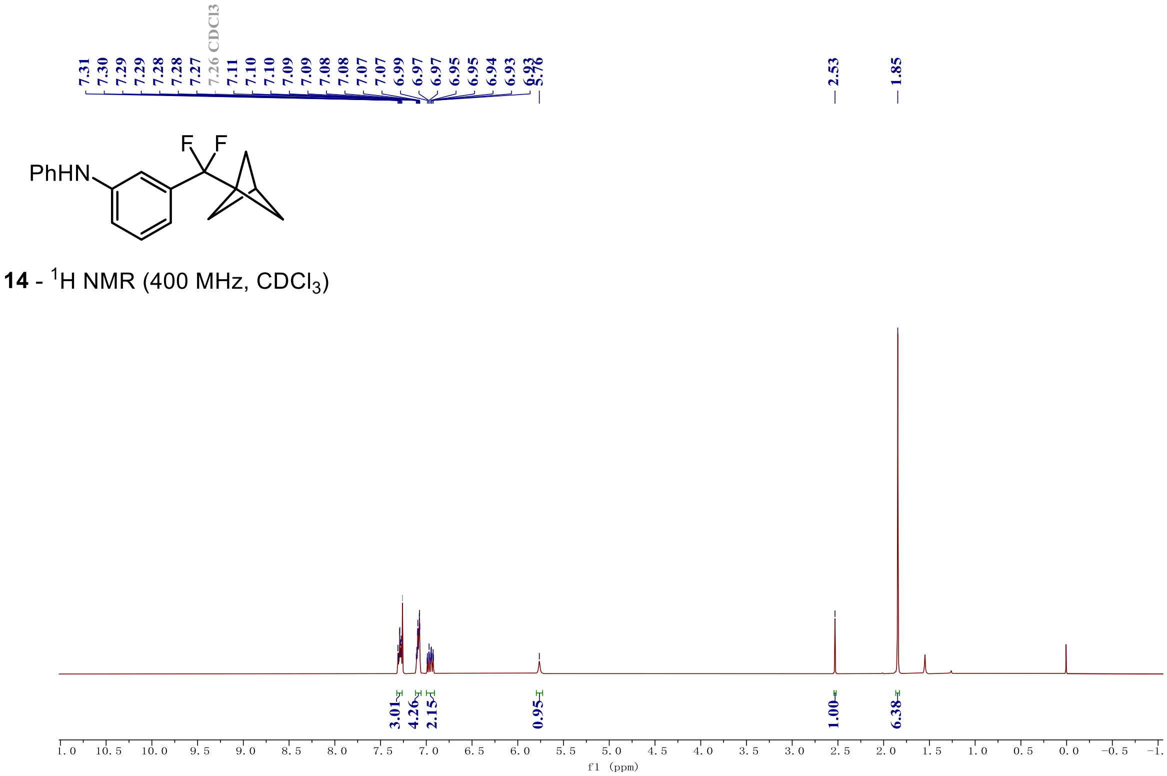

Supplementary Figure 60 ^{1}H NMR spectra of compound **14**

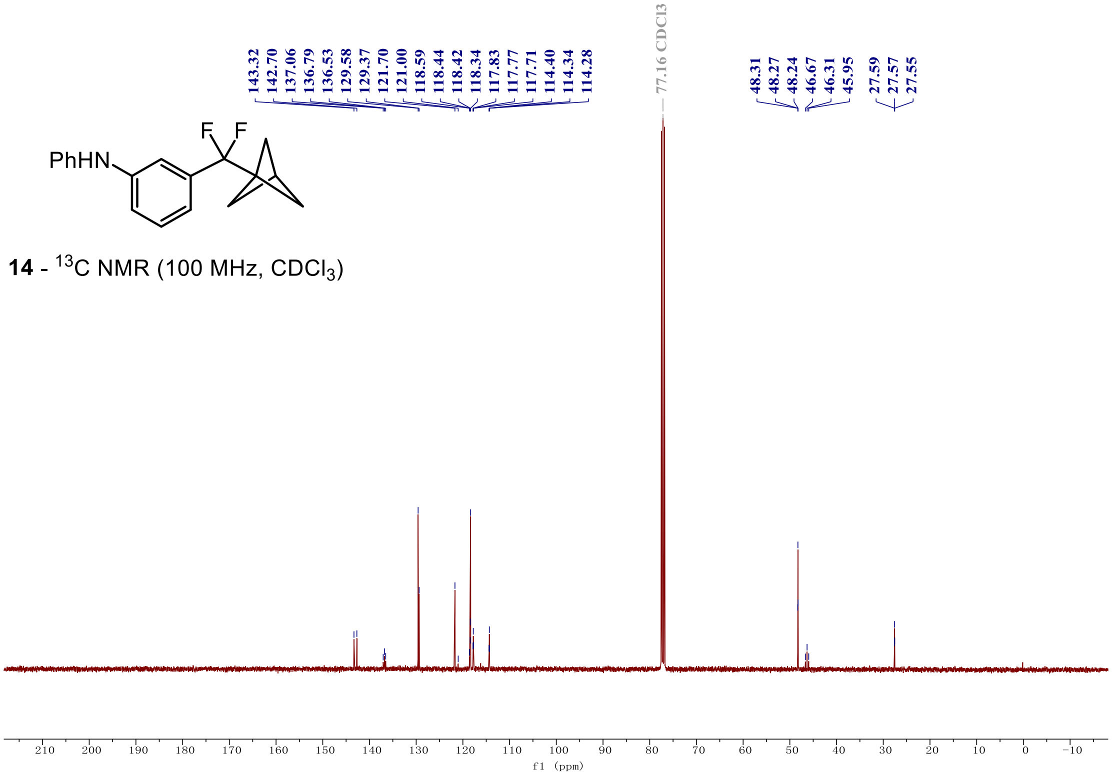

Supplementary Figure 61 ^{13}C NMR spectra of compound **14**

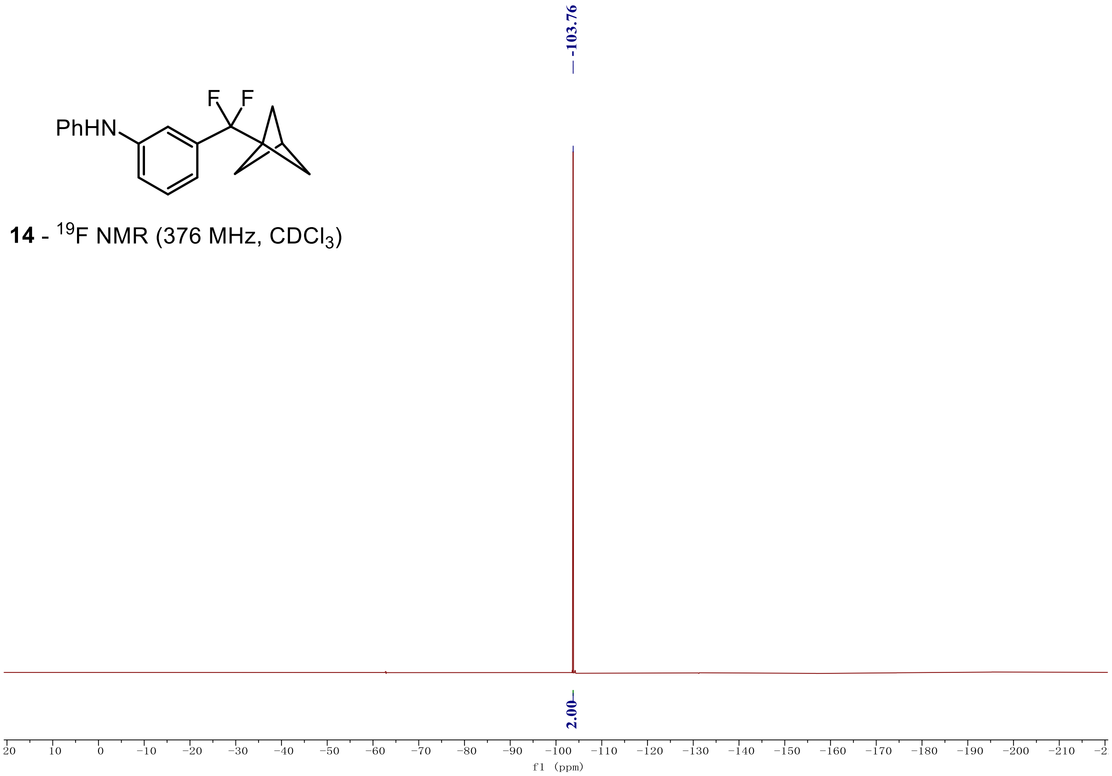

Supplementary Figure 62 ^{19}F NMR spectra of compound **14**

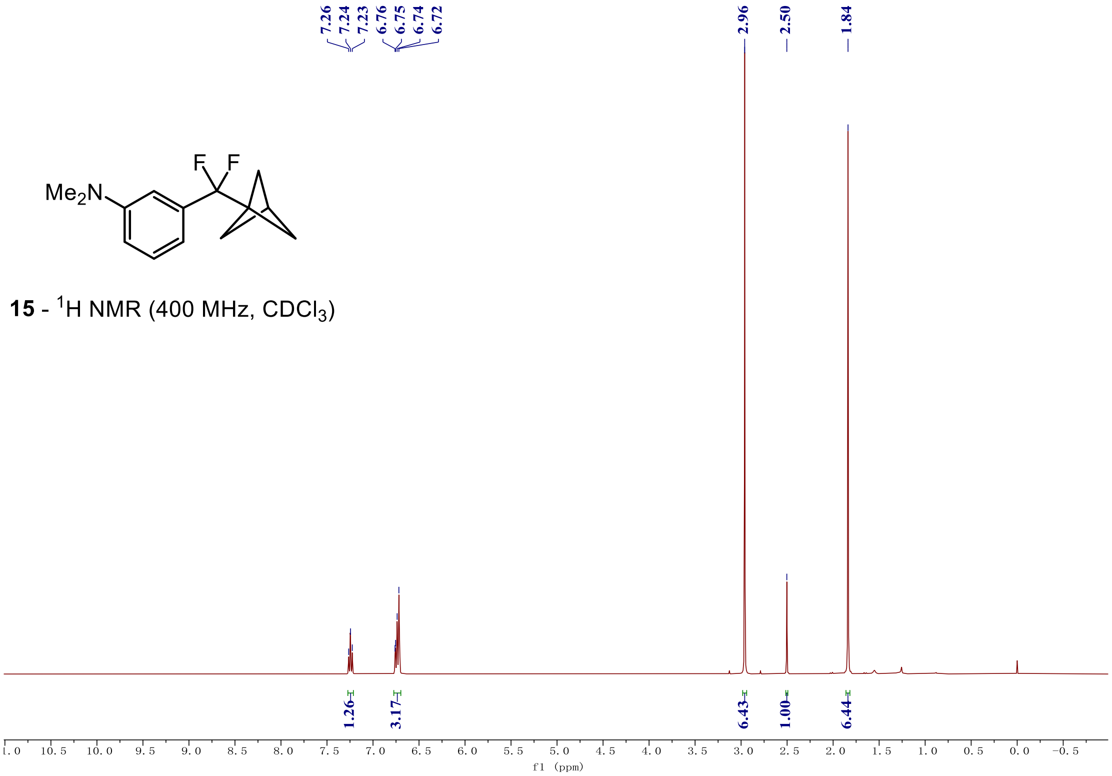

Supplementary Figure 63 ^{1}H NMR spectra of compound **15**

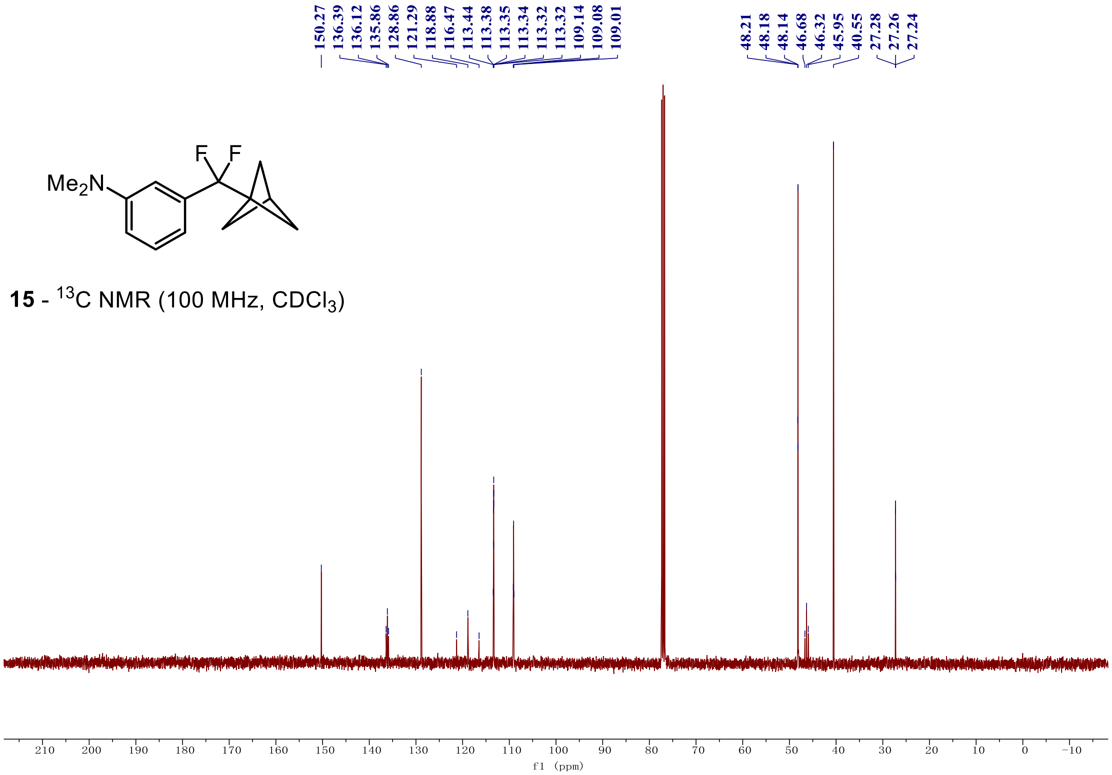

Supplementary Figure 64 ^{13}C NMR spectra of compound **15**

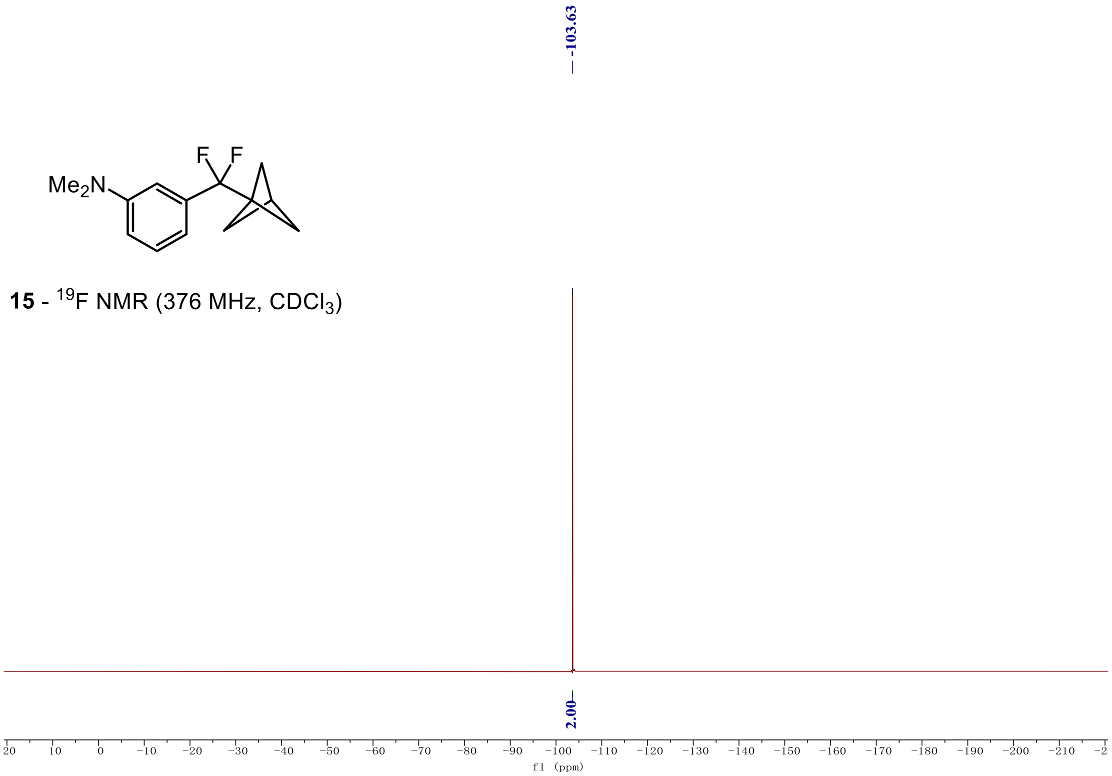

Supplementary Figure 65 ^{19}F NMR spectra of compound **15**

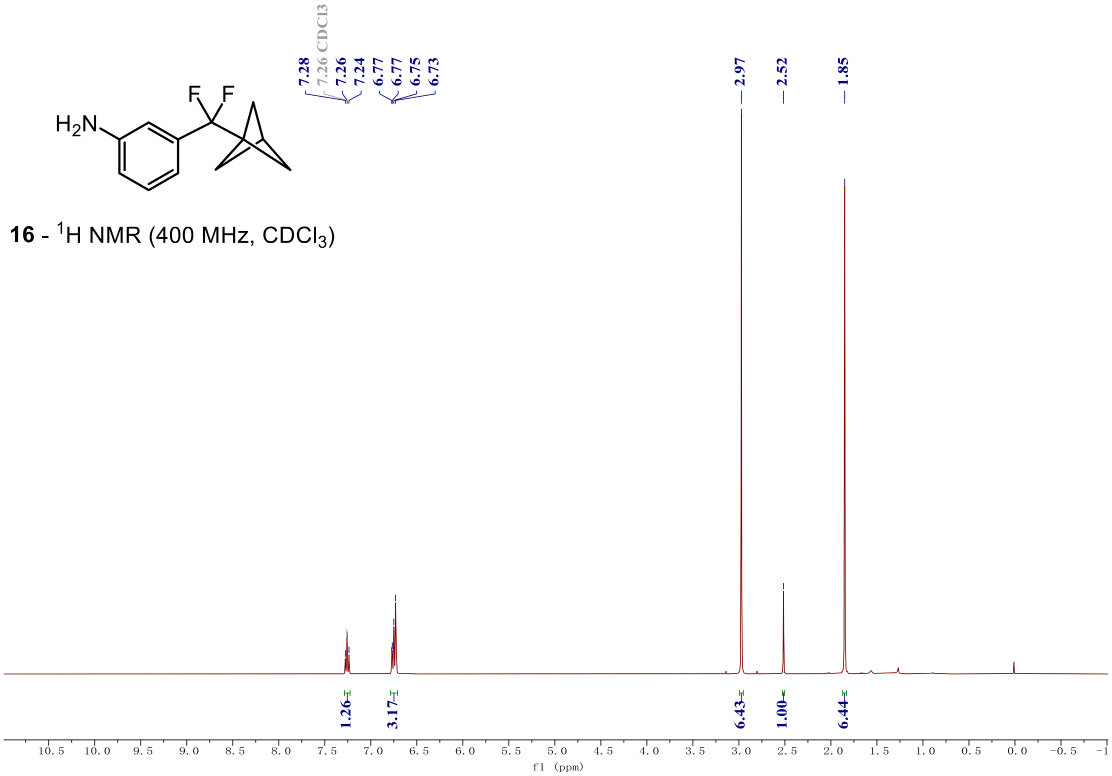

Supplementary Figure 66 ^{1}H NMR spectra of compound **16**

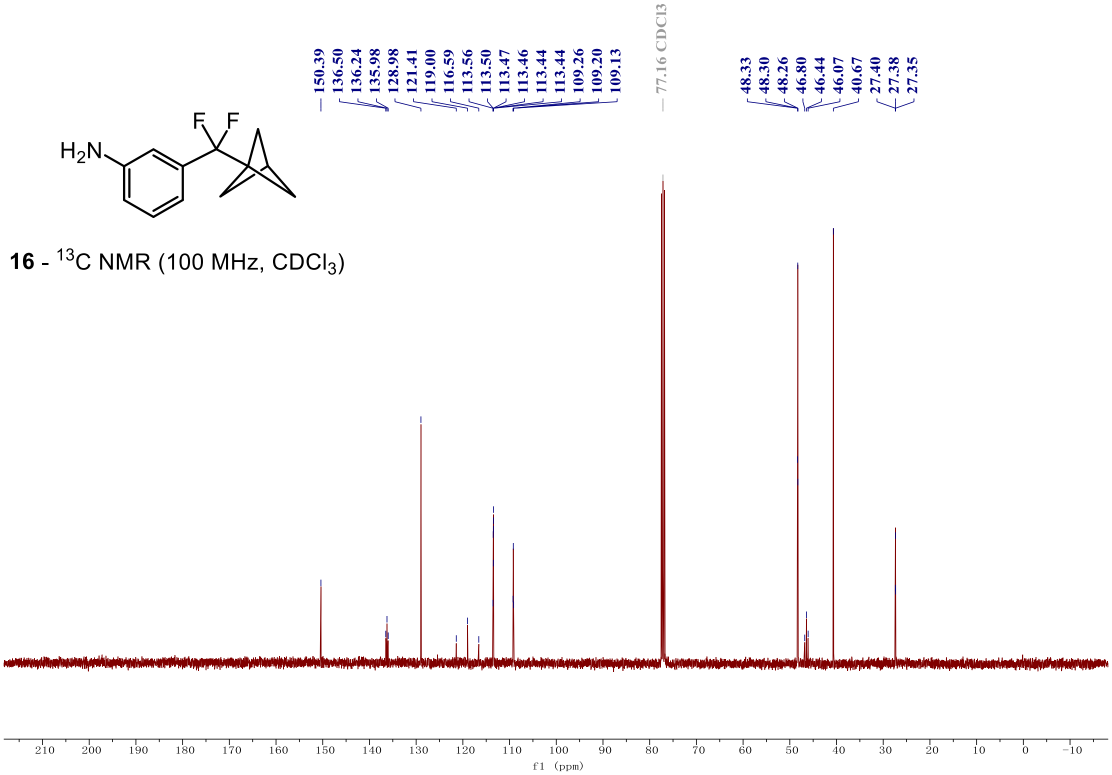

Supplementary Figure 67 ^{13}C NMR spectra of compound **16**

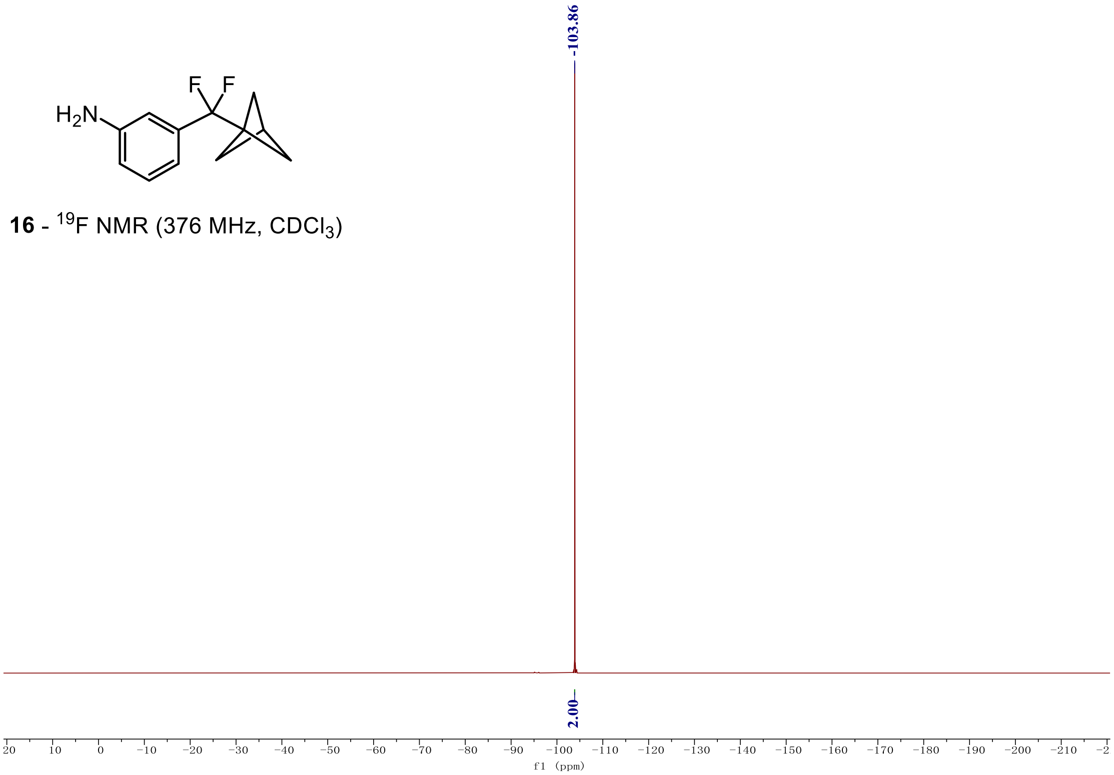

Supplementary Figure 68 ^{19}F NMR spectra of compound **16**

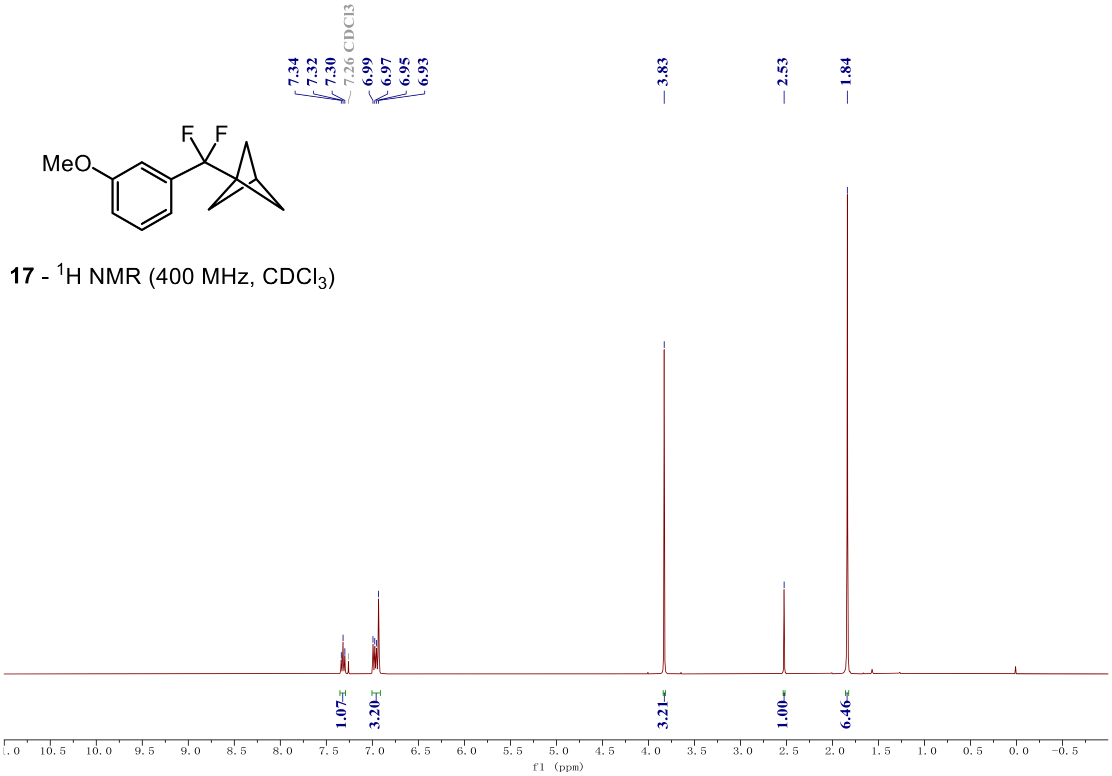

Supplementary Figure 69 ^{1}H NMR spectra of compound **17**

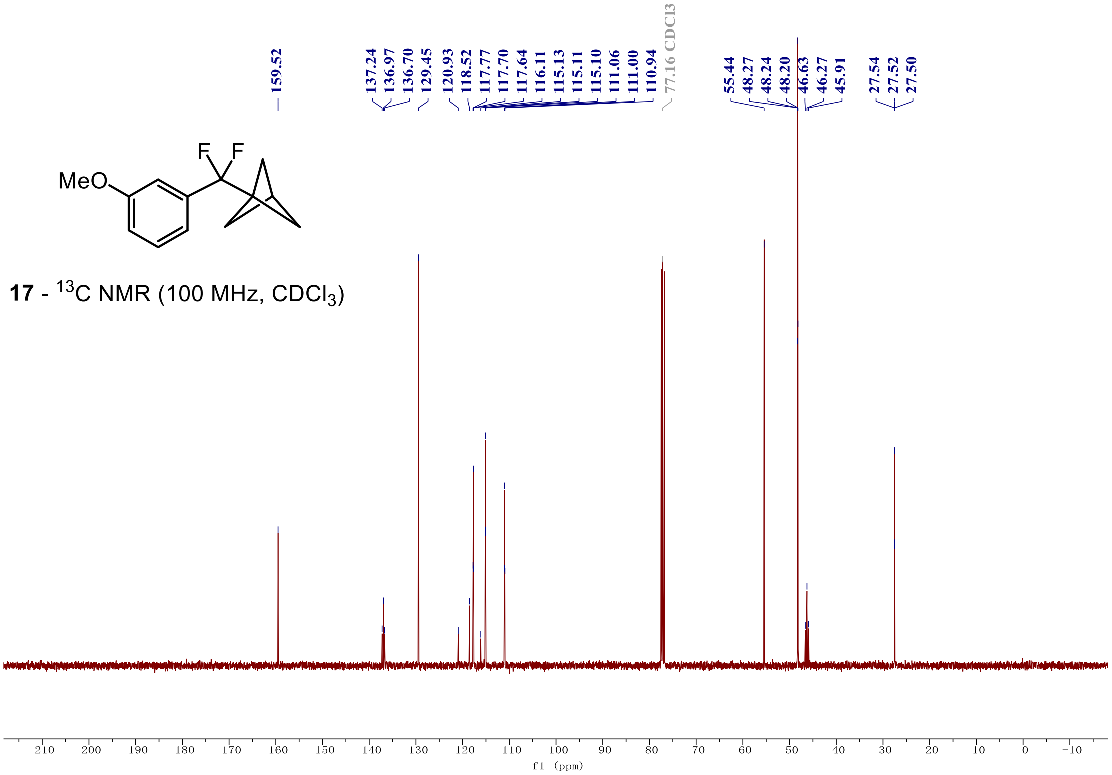

Supplementary Figure 70 ^{13}C NMR spectra of compound **17**

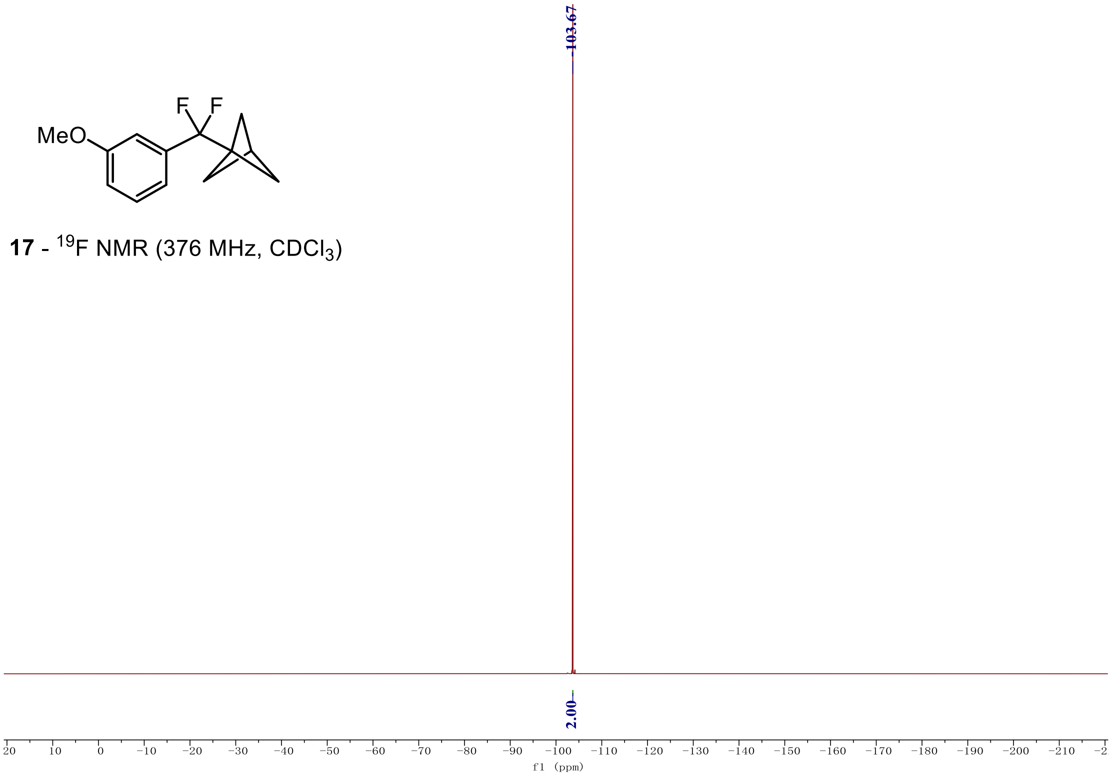

Supplementary Figure 71 ^{19}F NMR spectra of compound **17**

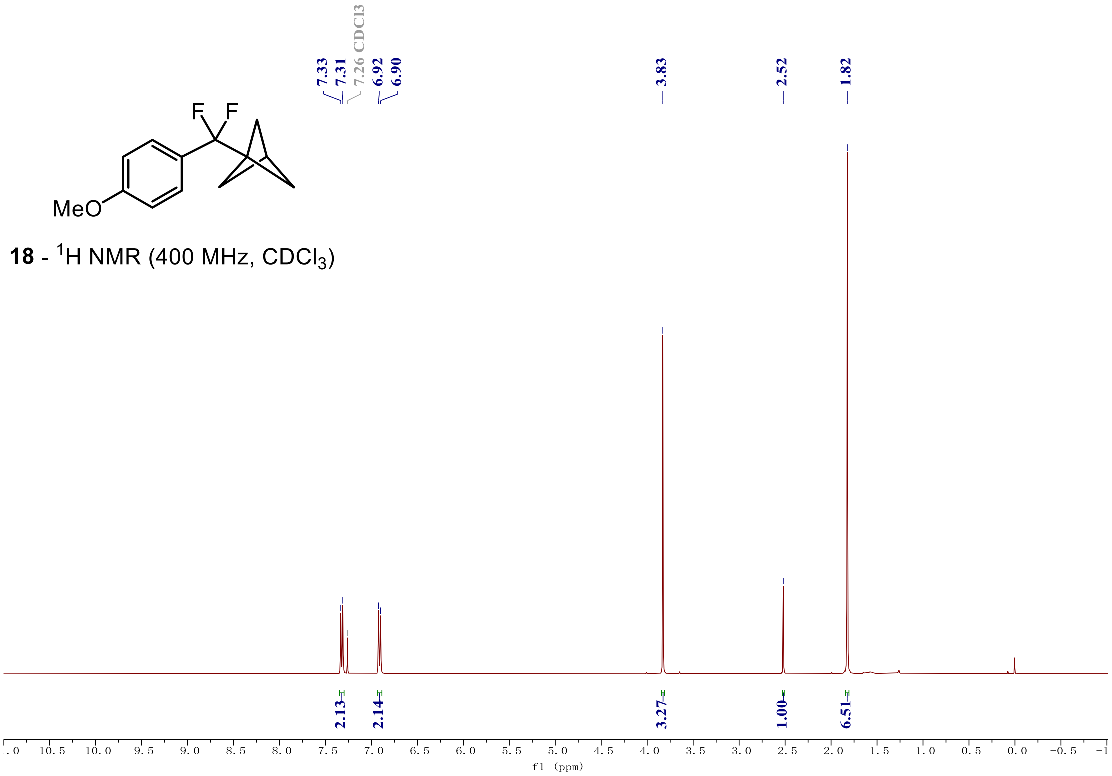

Supplementary Figure 72 ^{1}H NMR spectra of compound **18**

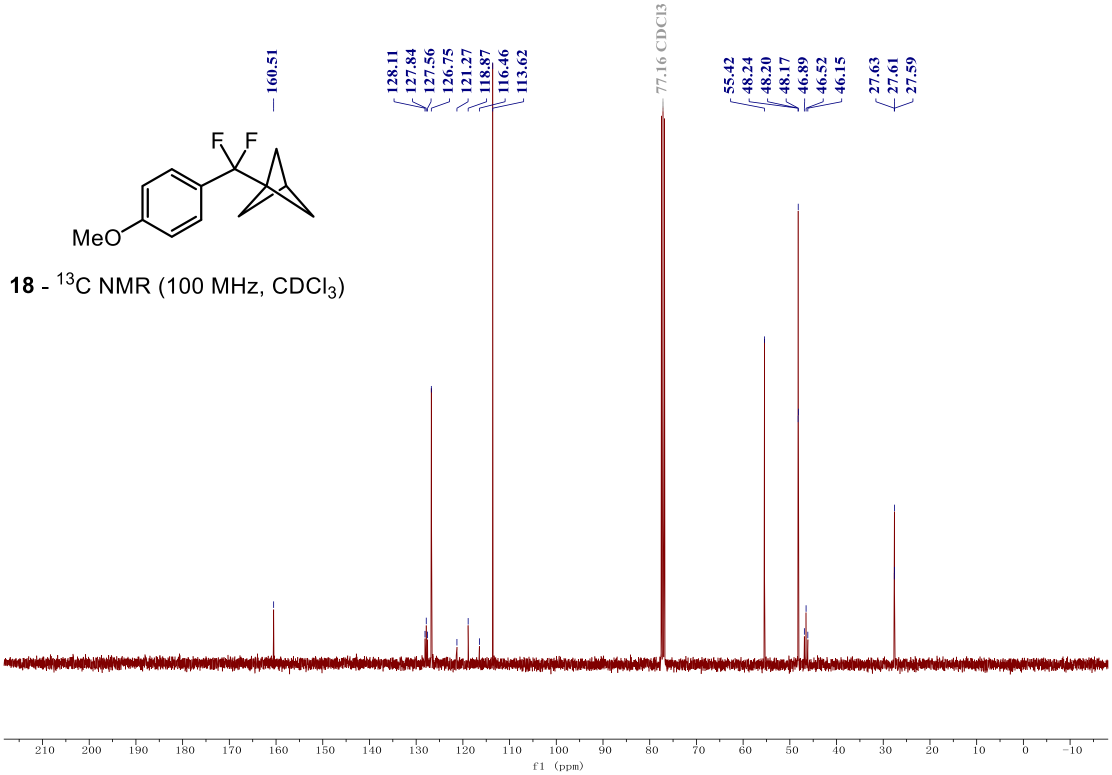

Supplementary Figure 73 ^{13}C NMR spectra of compound **18**

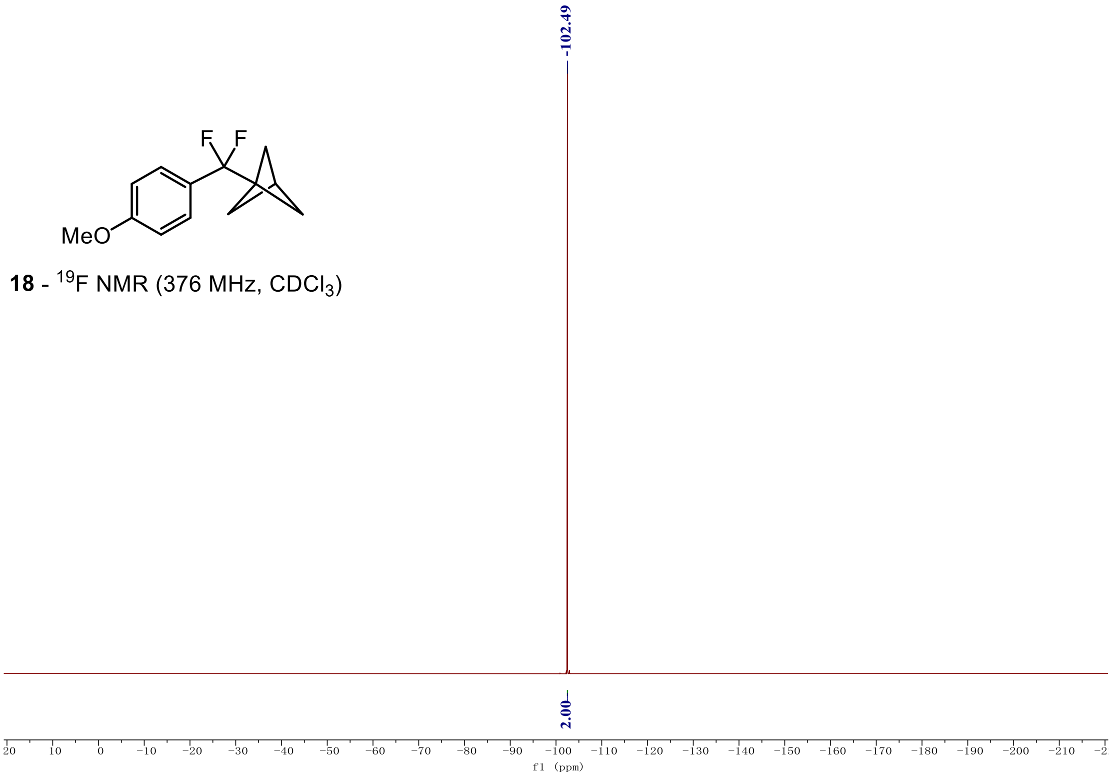

Supplementary Figure 74 ^{19}F NMR spectra of compound **18**

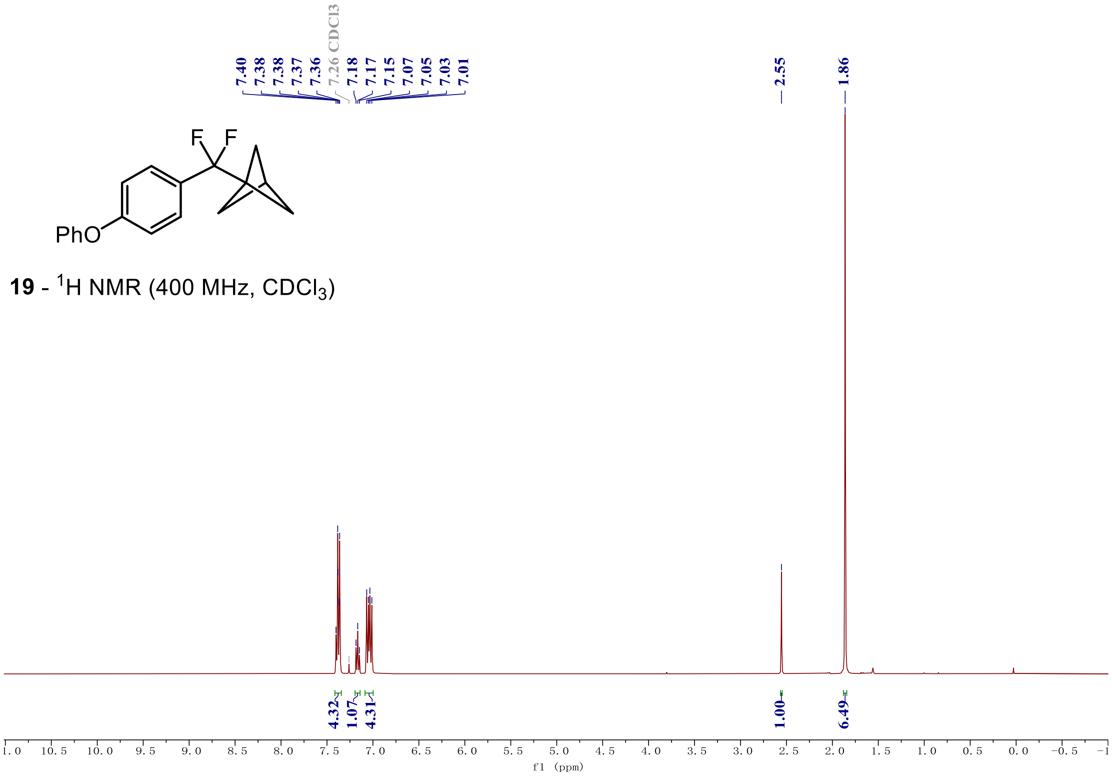

Supplementary Figure 75 ^{1}H NMR spectra of compound **19**

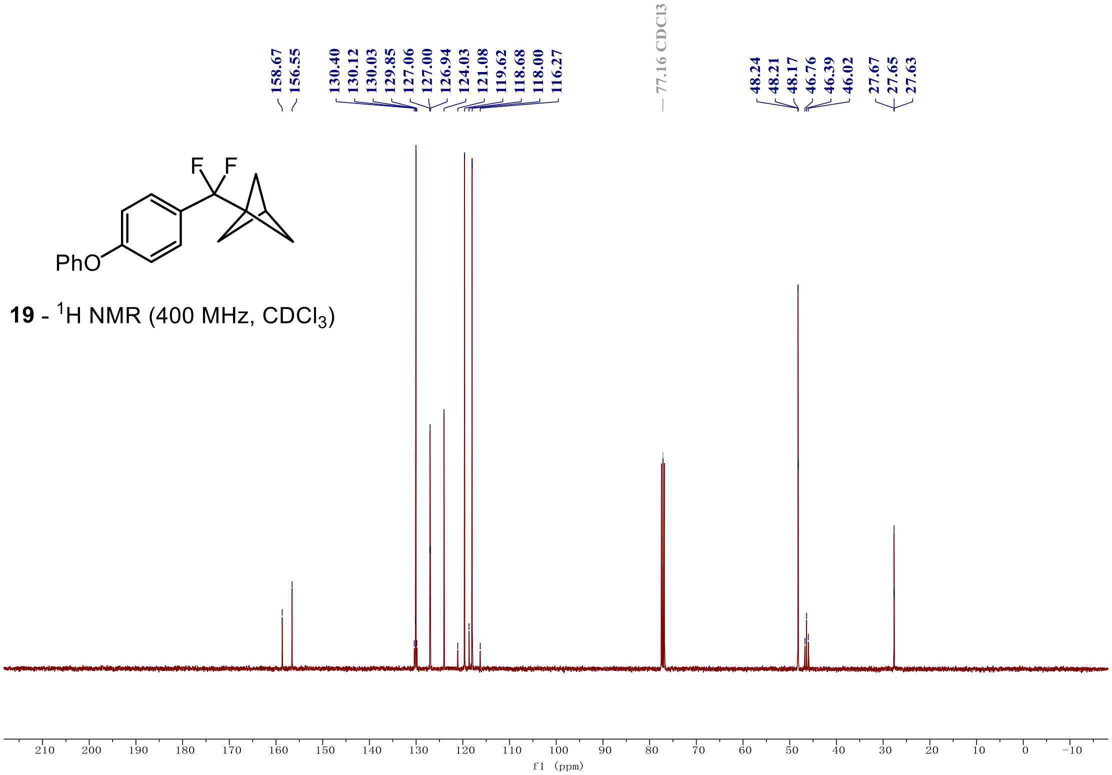

Supplementary Figure 76 ^{13}C NMR spectra of compound **19**

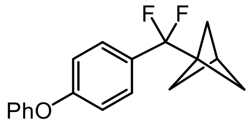

19 - ^{19}F NMR (376 MHz, $CDCl_3$)

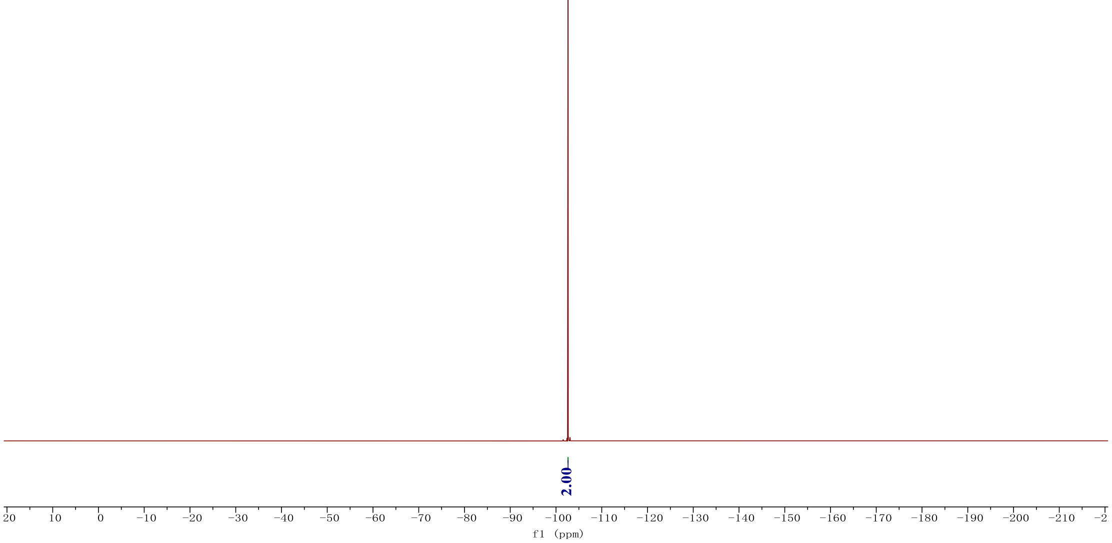

Supplementary Figure 77 ^{19}F NMR spectra of compound **19**

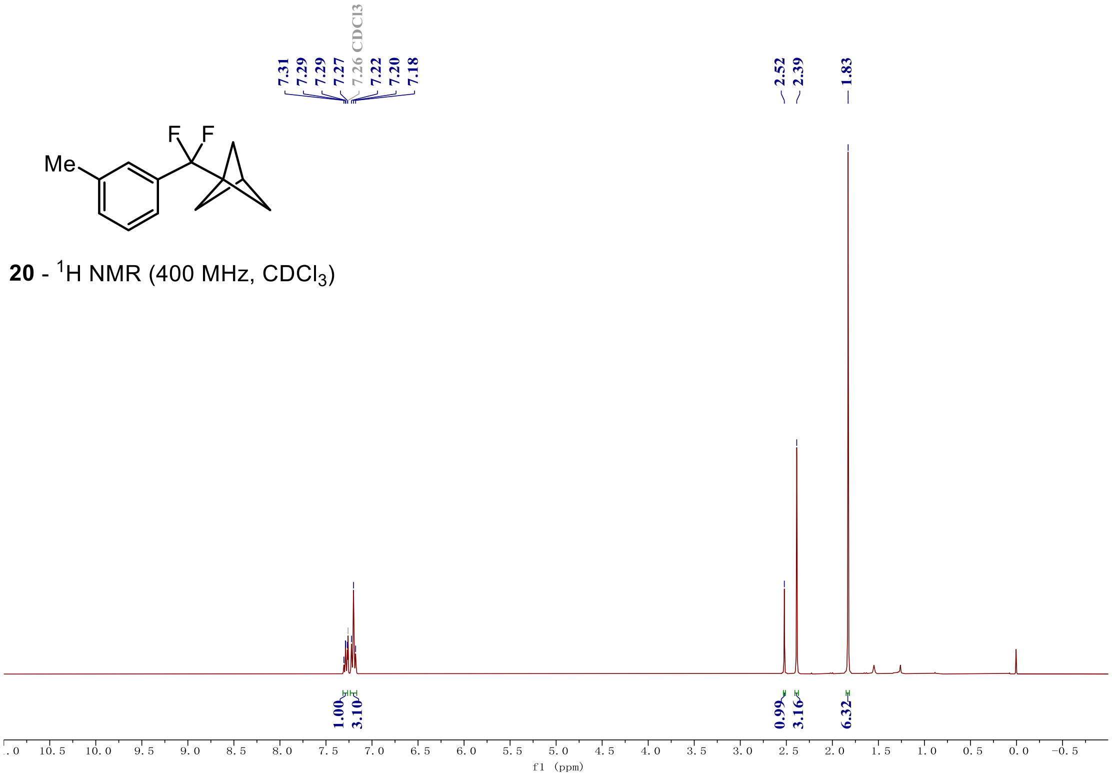

Supplementary Figure 78 ^{1}H NMR spectra of compound **20**

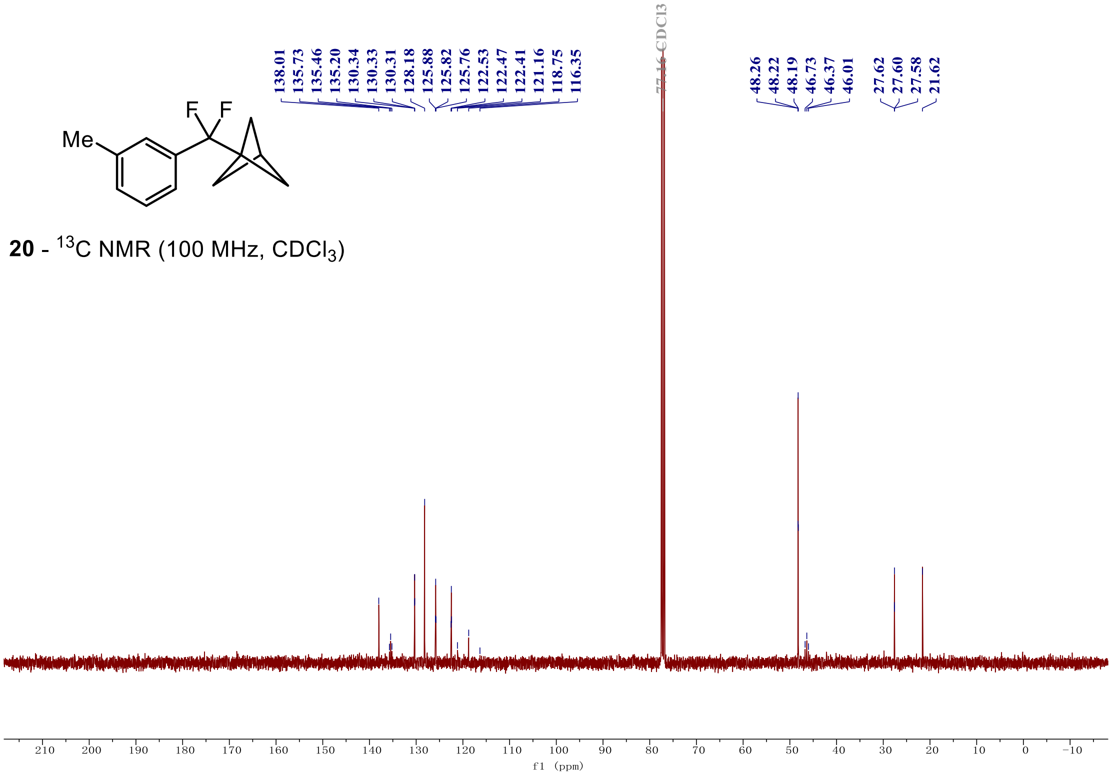

Supplementary Figure 79 ^{13}C NMR spectra of compound **20**

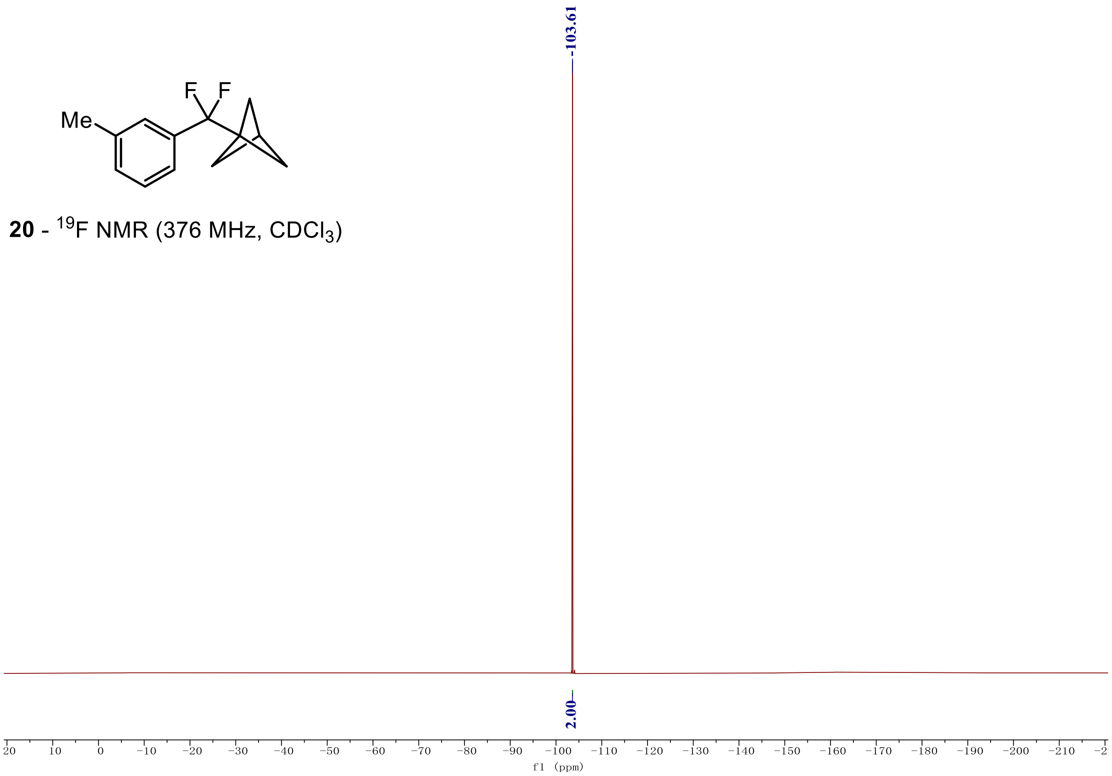

Supplementary Figure 80 ^{19}F NMR spectra of compound **20**

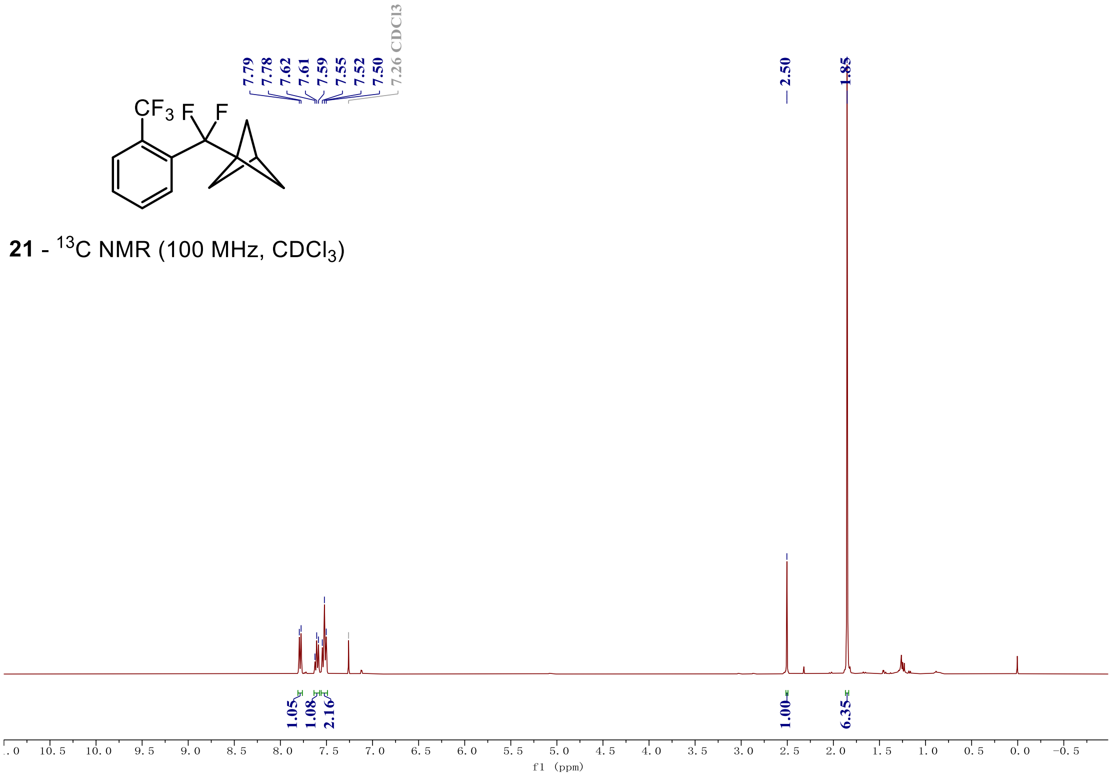

Supplementary Figure 81 ^{1}H NMR spectra of compound **21**

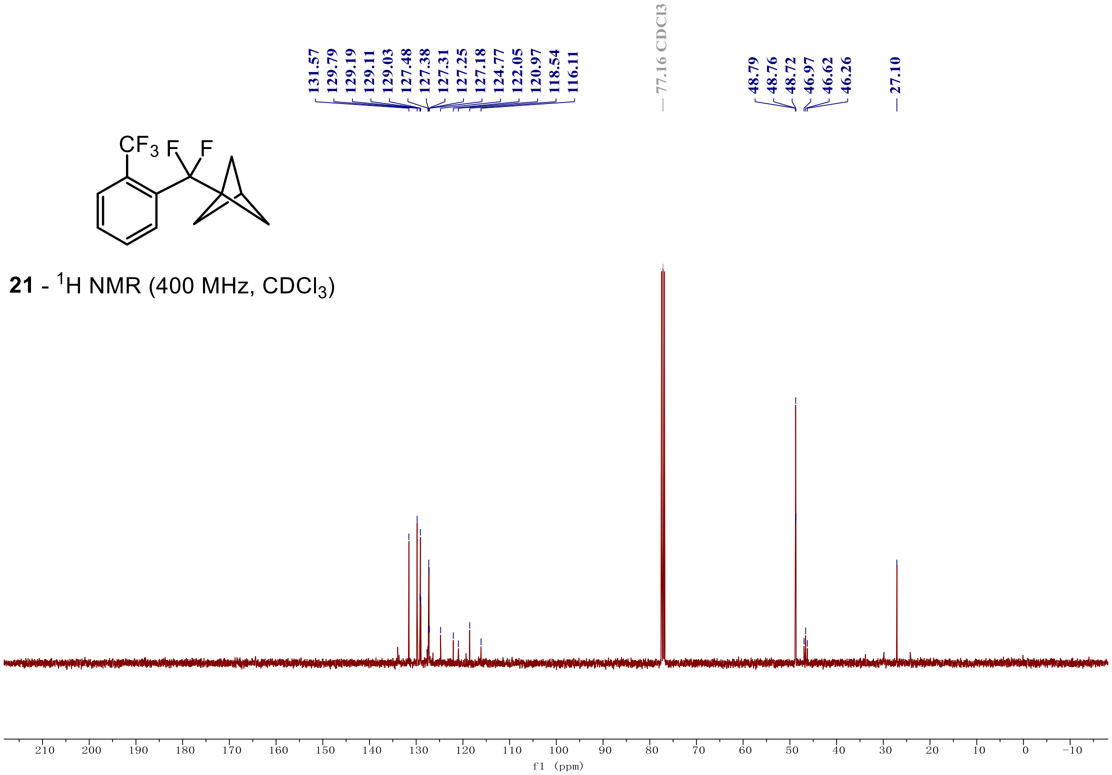

Supplementary Figure 82 ^{13}C NMR spectra of compound **21**

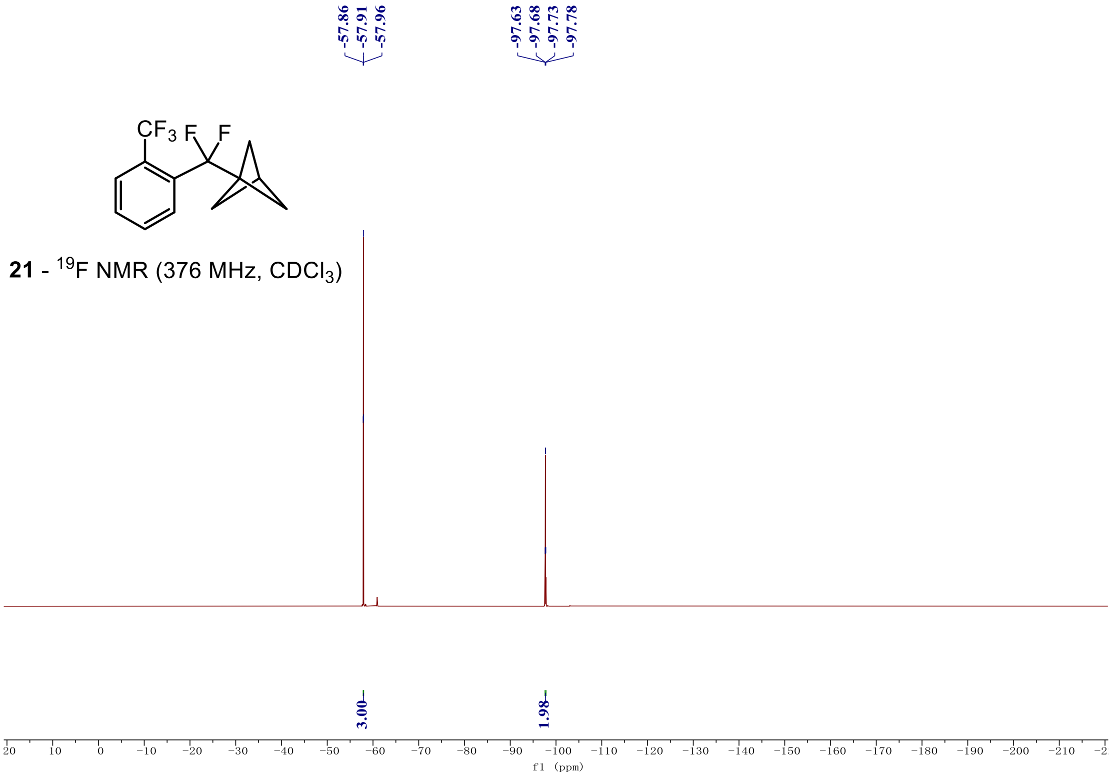

Supplementary Figure 83 ^{19}F NMR spectra of compound **21**

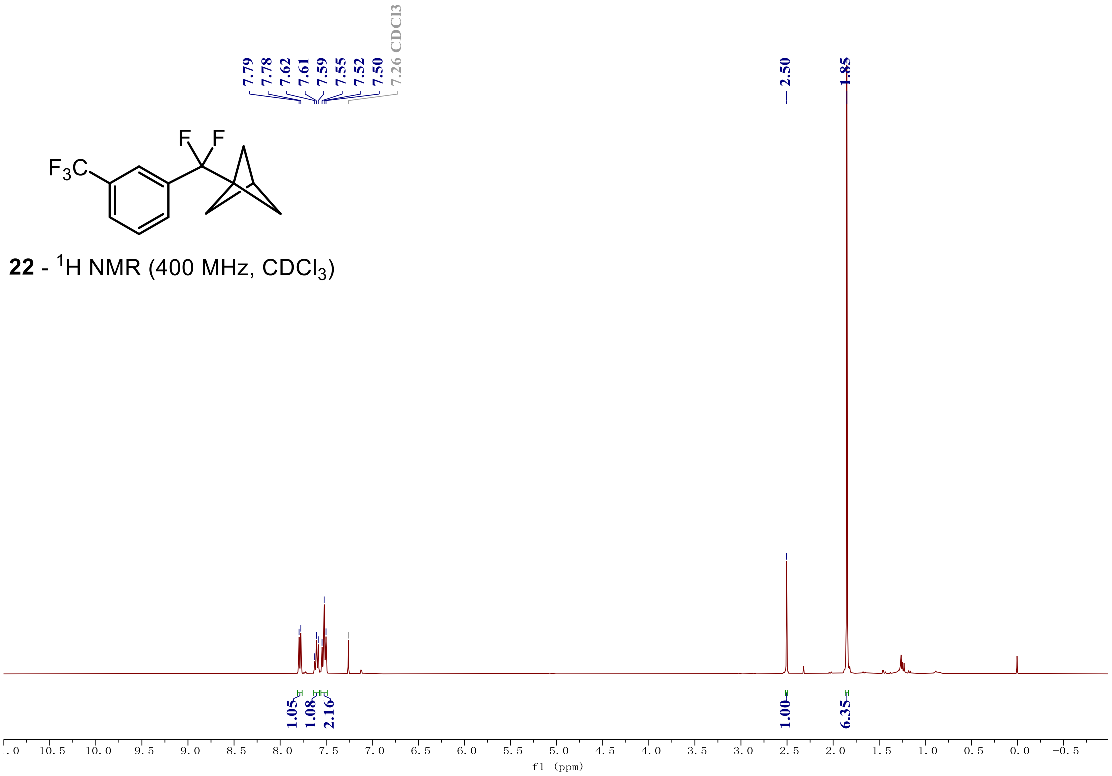

Supplementary Figure 84 ^{1}H NMR spectra of compound **22**

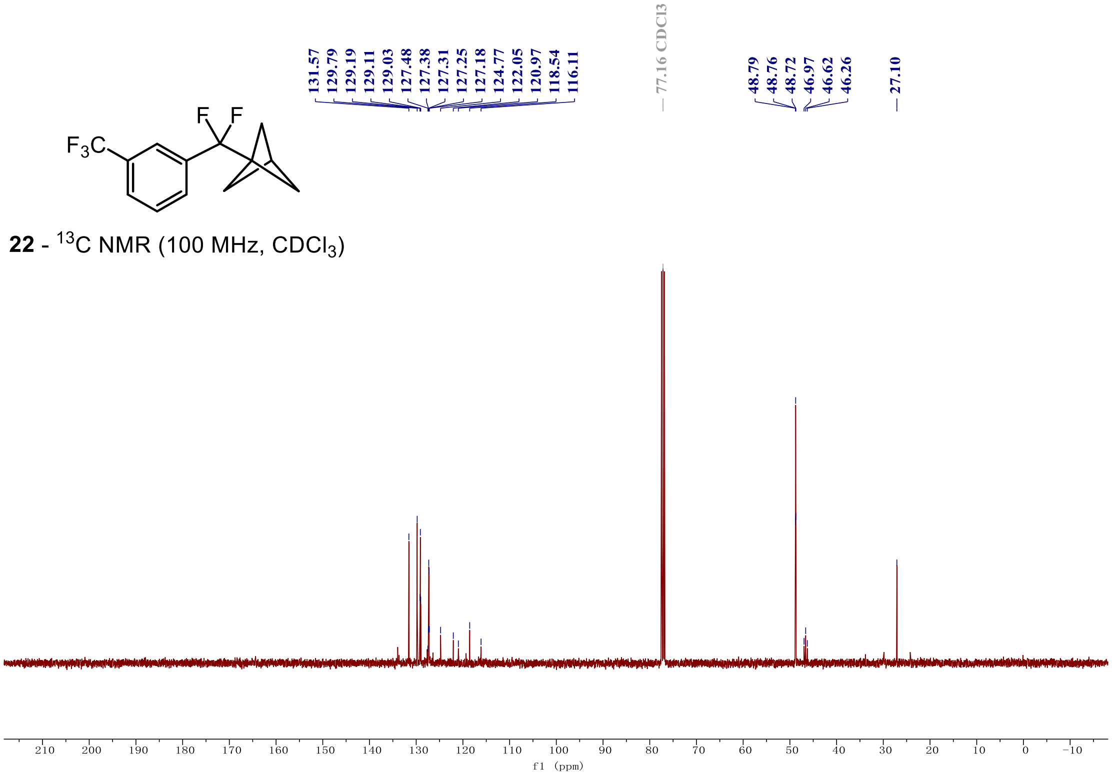

Supplementary Figure 85 ^{13}C NMR spectra of compound **22**

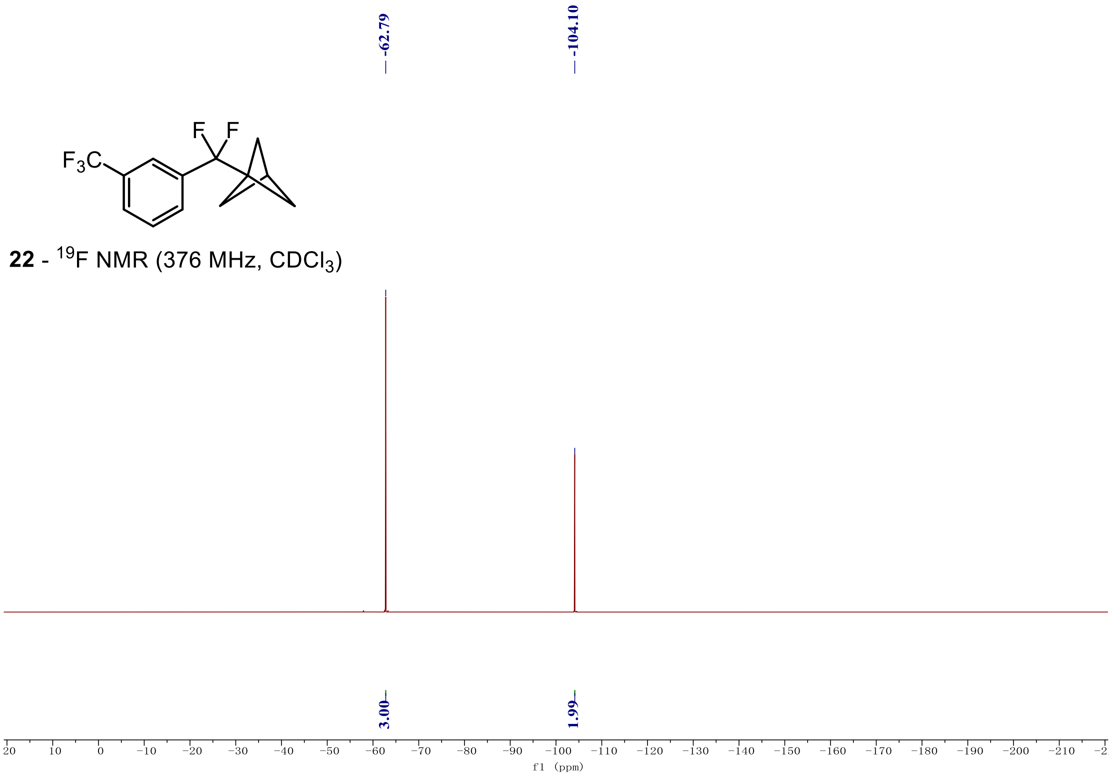

Supplementary Figure 86 ^{19}F NMR spectra of compound **22**

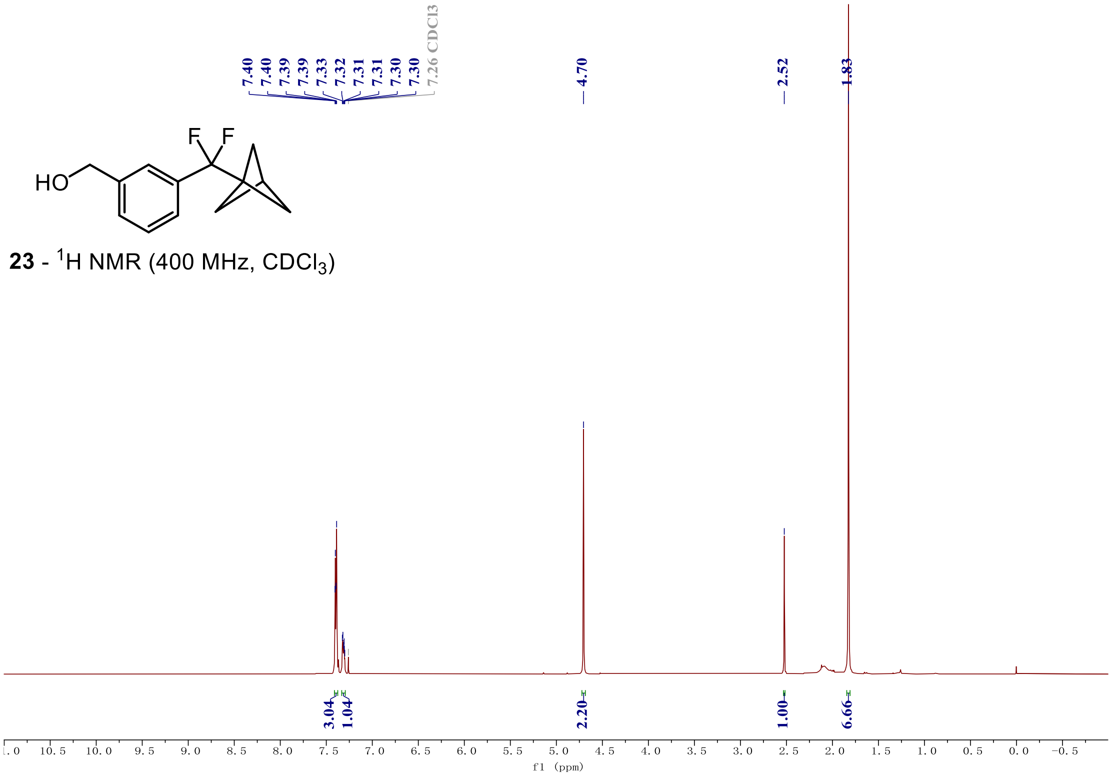

Supplementary Figure 87 ^{1}H NMR spectra of compound **23**

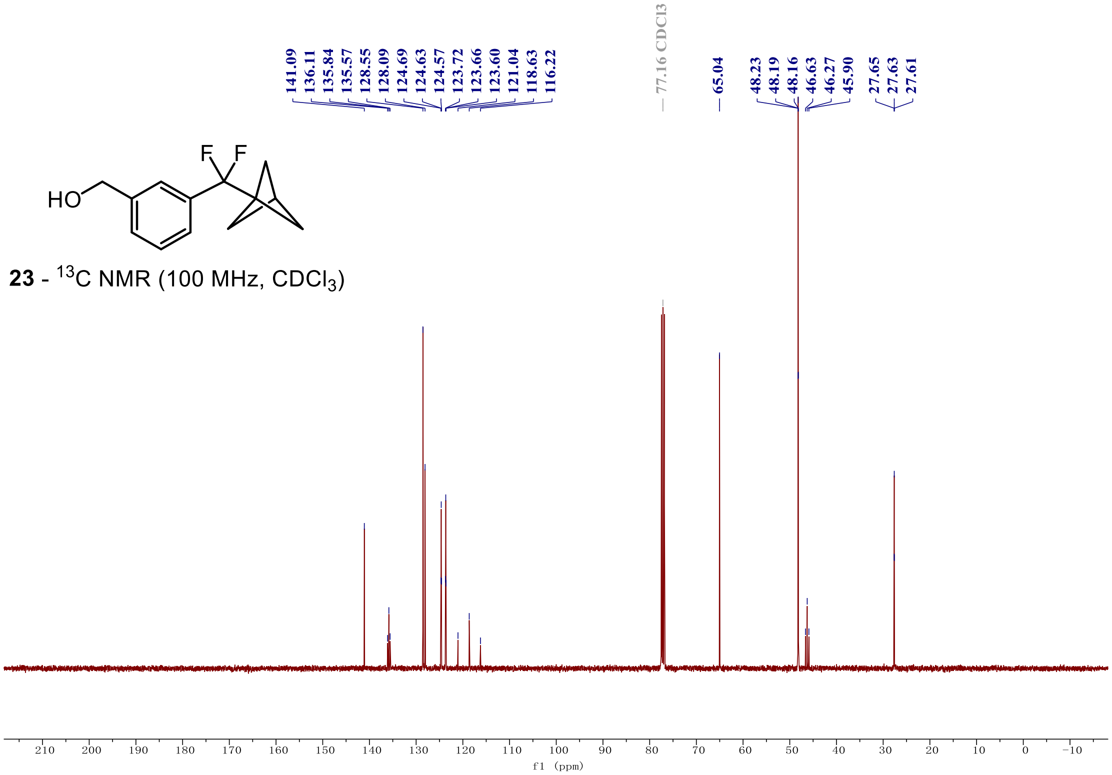

Supplementary Figure 88 ^{13}C NMR spectra of compound **23**

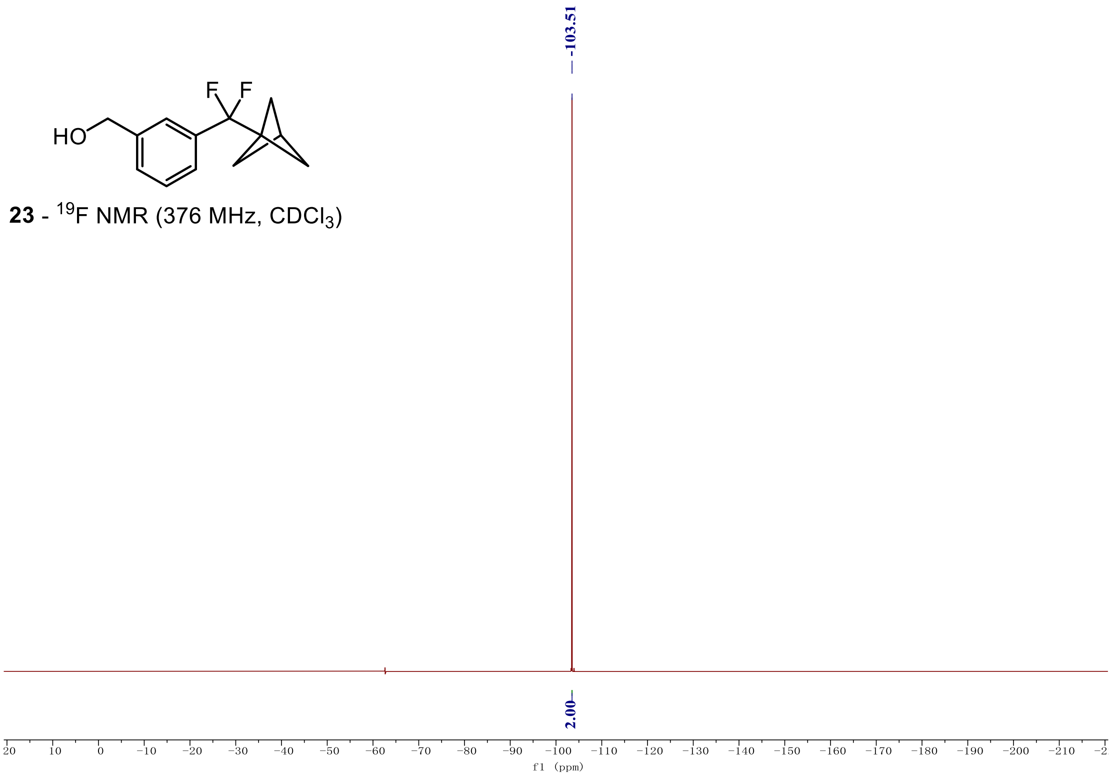

Supplementary Figure 89 ^{19}F NMR spectra of compound **23**

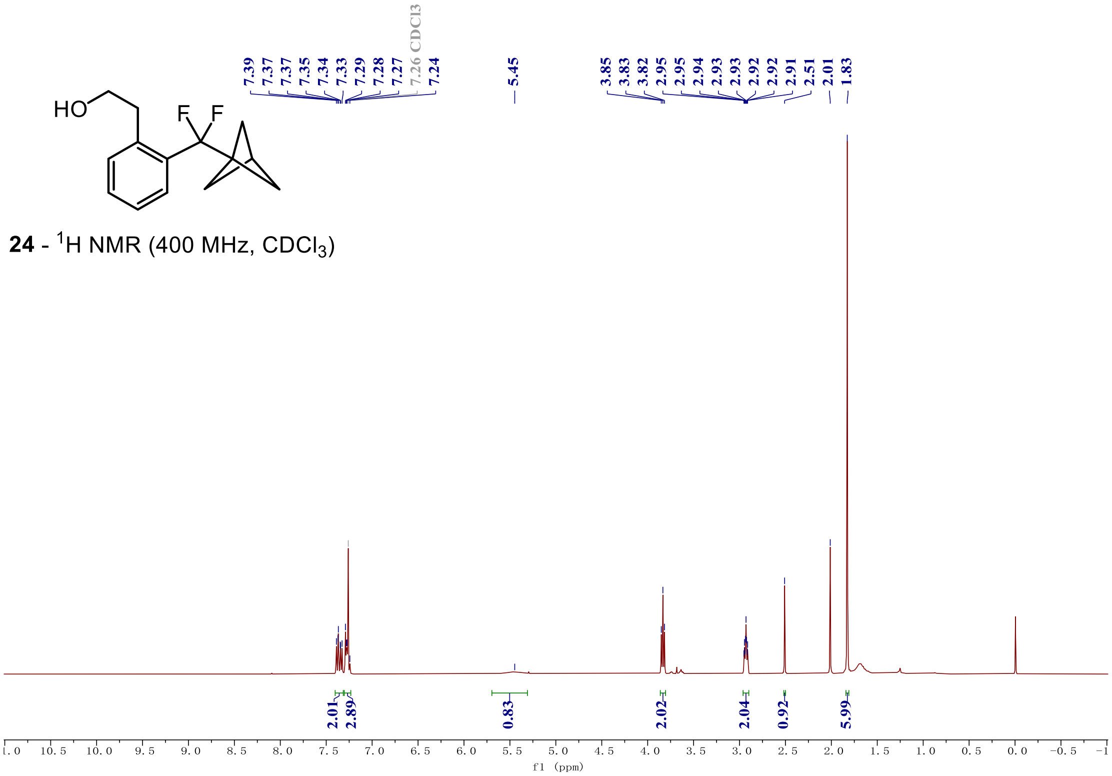

Supplementary Figure 90 ^{1}H NMR spectra of compound **24**

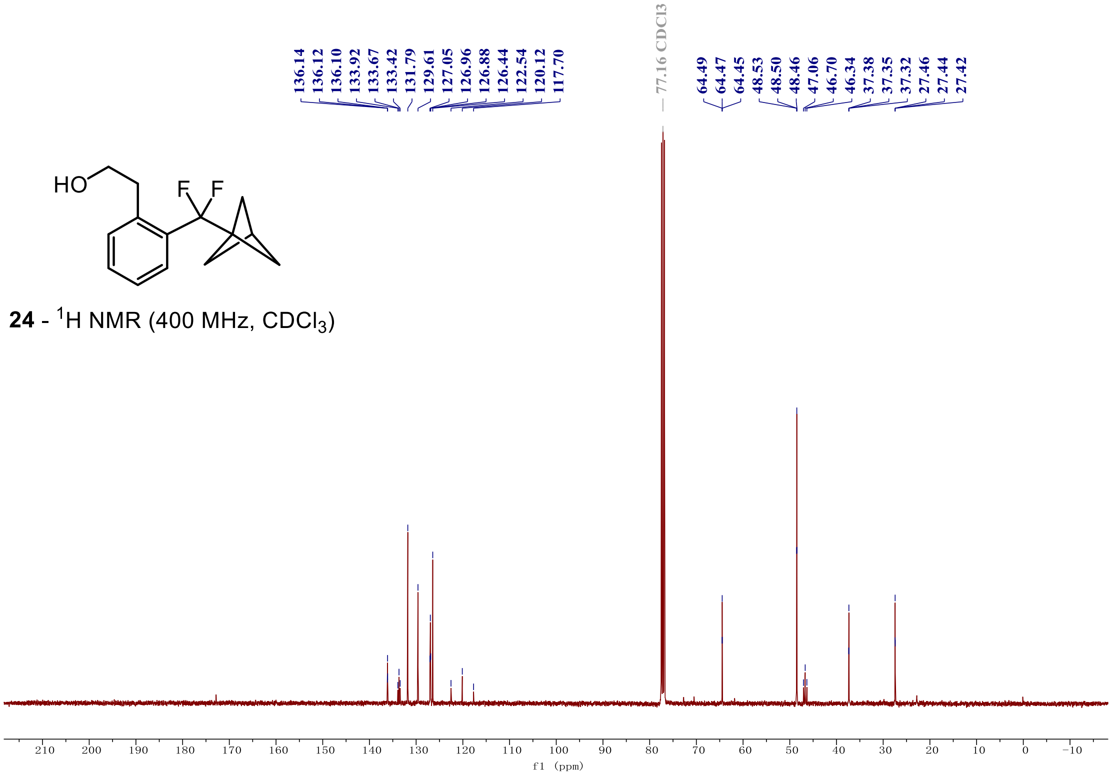

Supplementary Figure 91 ^{13}C NMR spectra of compound **24**

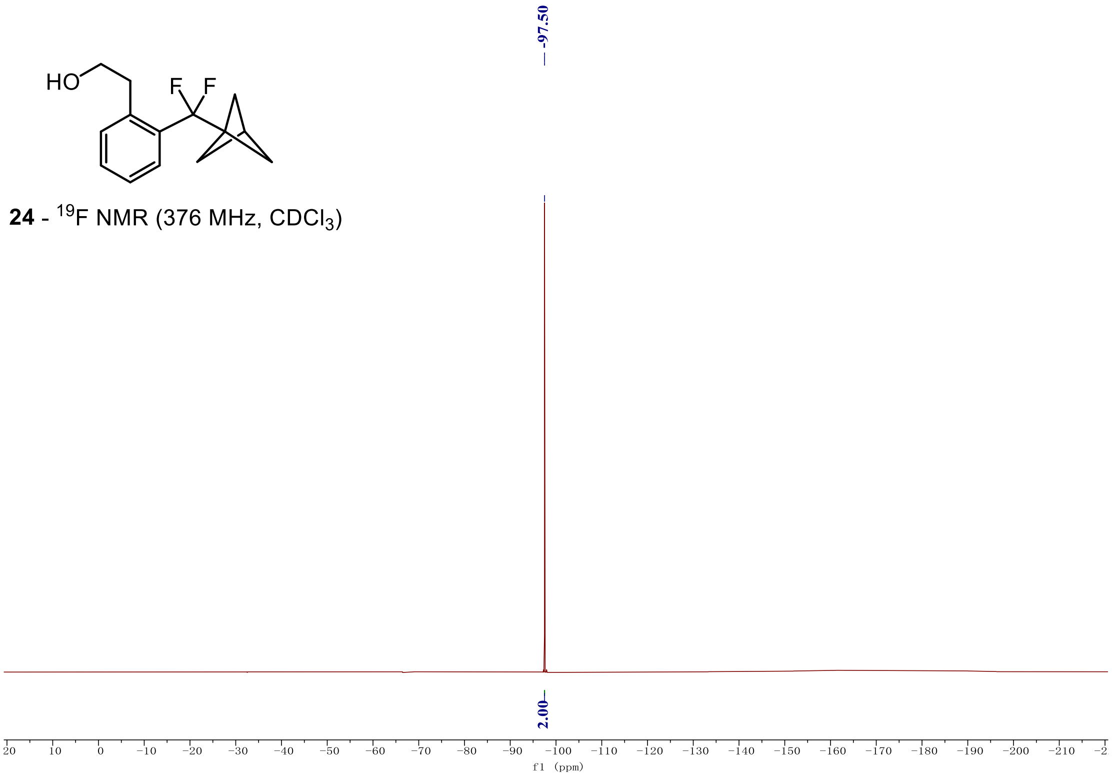

Supplementary Figure 92 ^{19}F NMR spectra of compound **24**

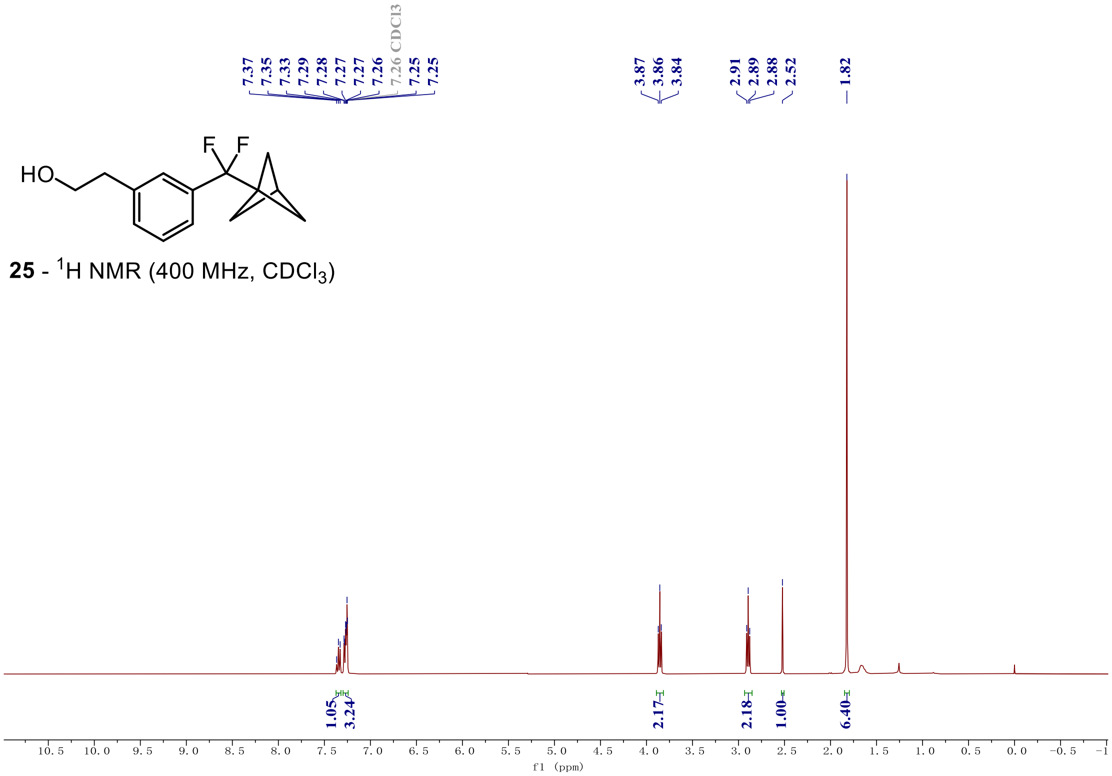

Supplementary Figure 93 ^{1}H NMR spectra of compound **25**

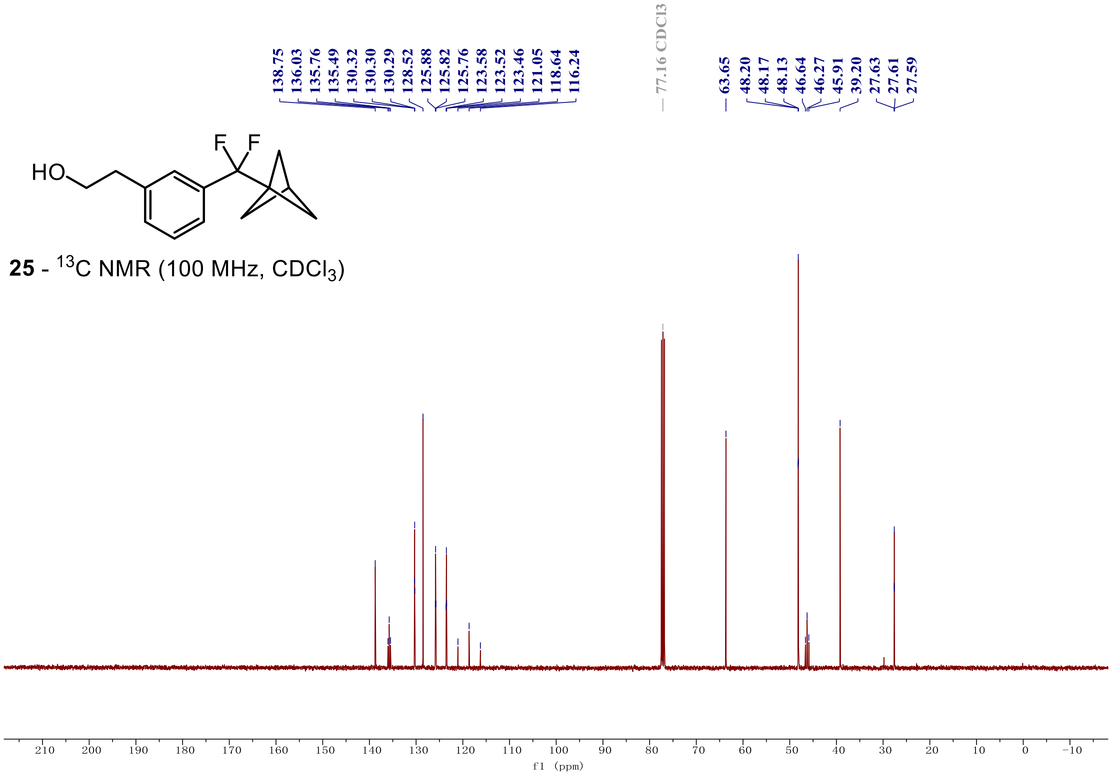

Supplementary Figure 94 ^{13}C NMR spectra of compound **25**

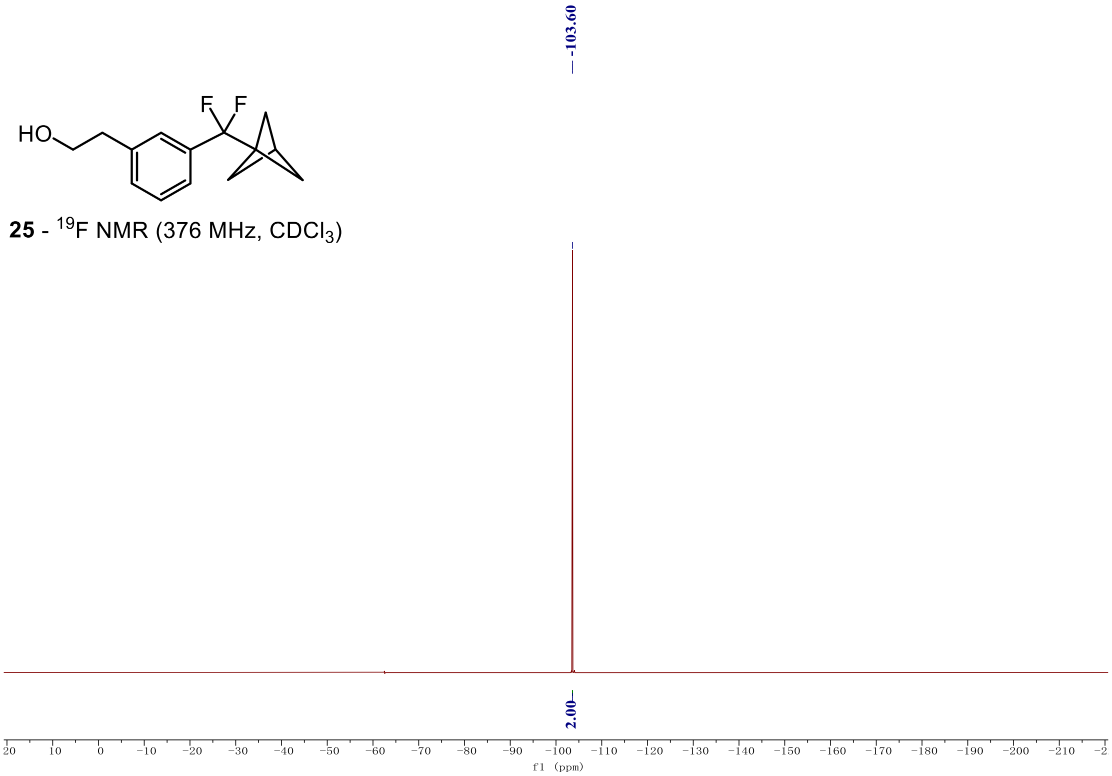

Supplementary Figure 95 ^{19}F NMR spectra of compound **25**

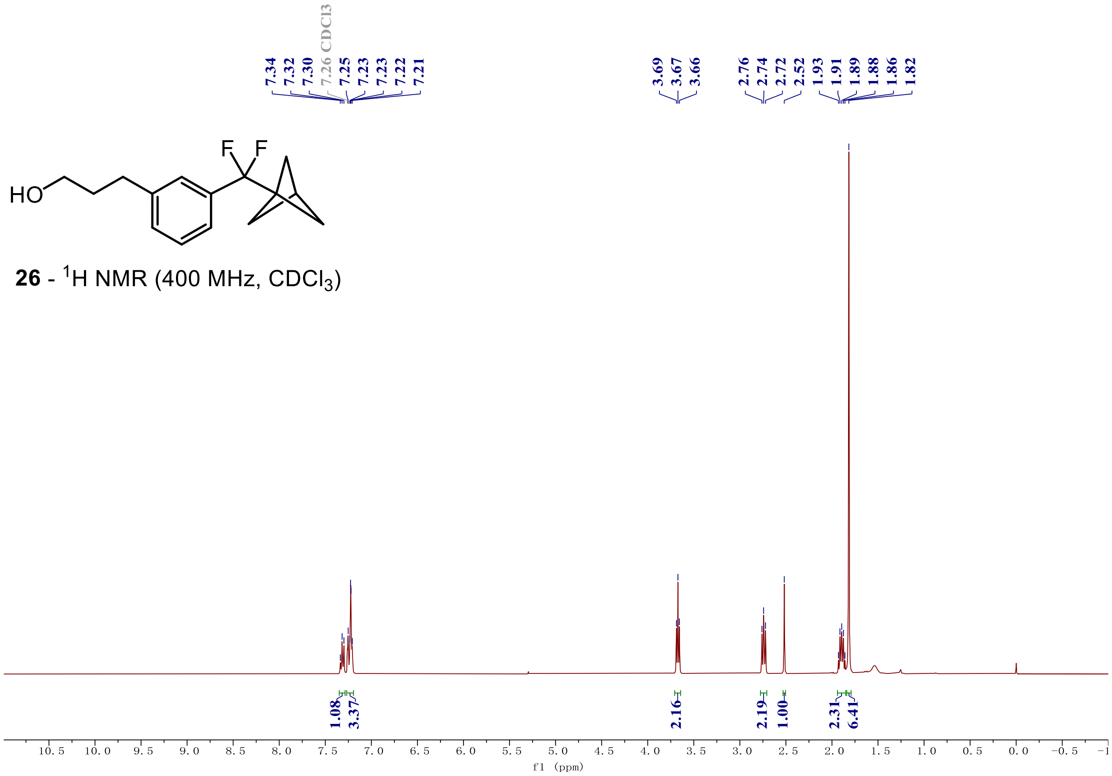

Supplementary Figure 96 ^{1}H NMR spectra of compound **26**

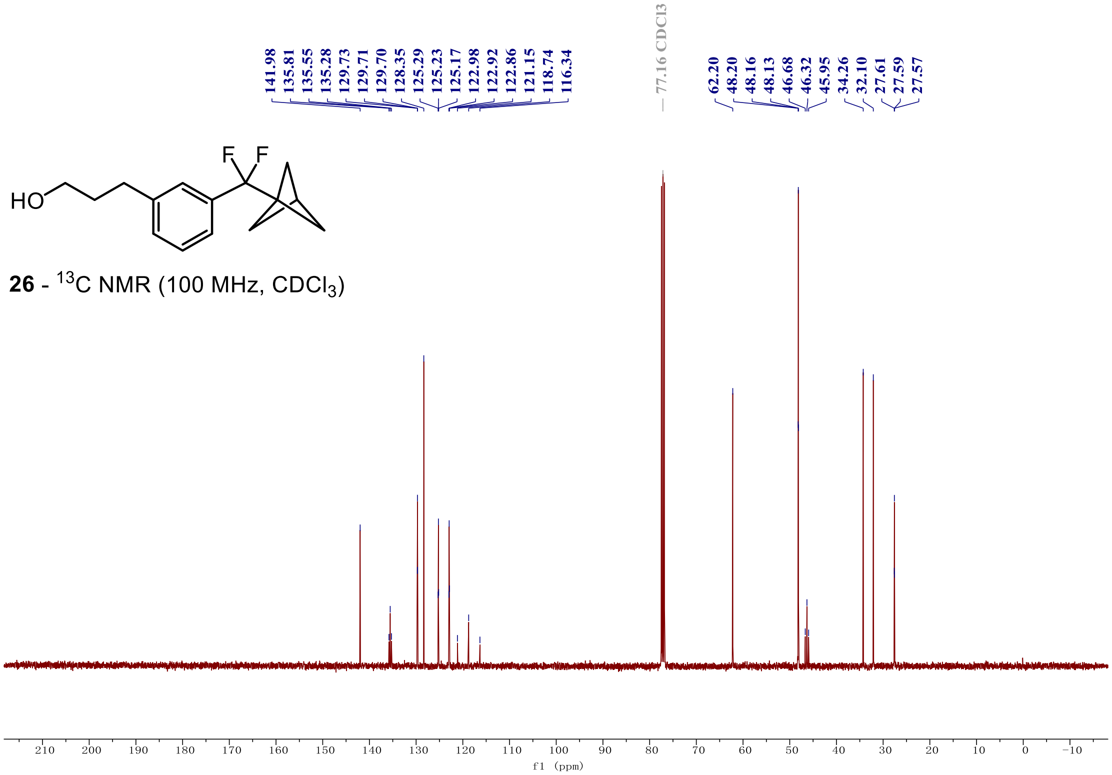

Supplementary Figure 97 ^{13}C NMR spectra of compound **26**

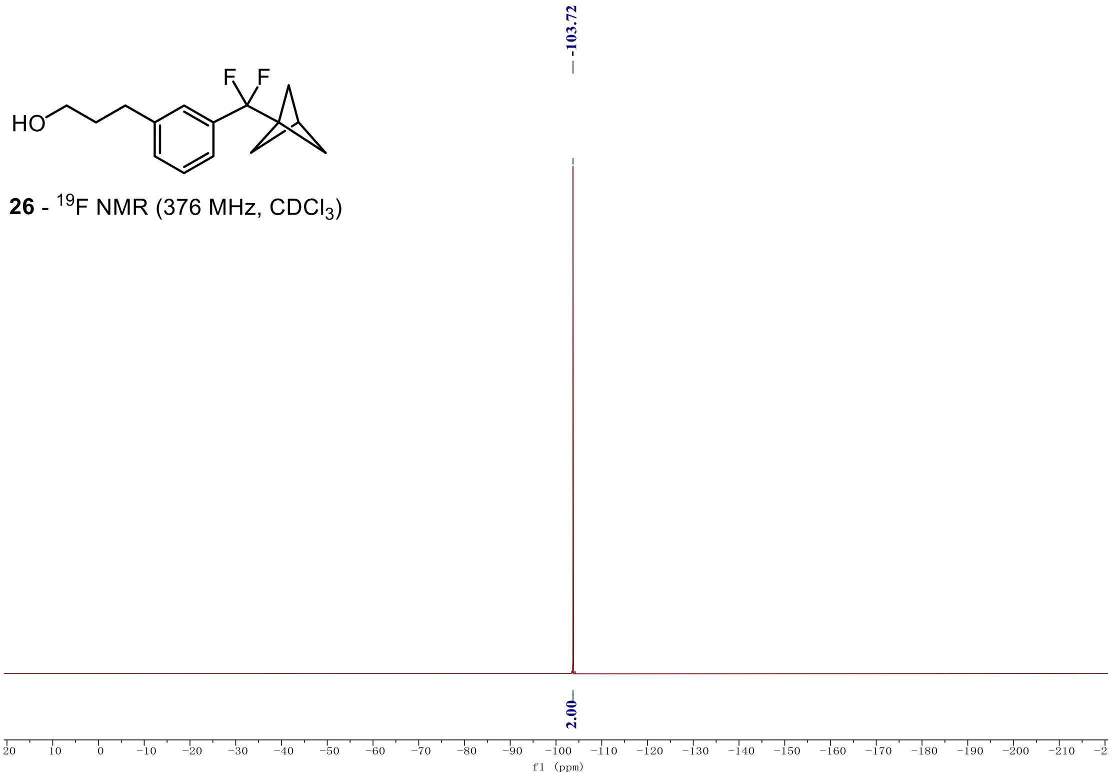

Supplementary Figure 98 ^{19}F NMR spectra of compound **26**

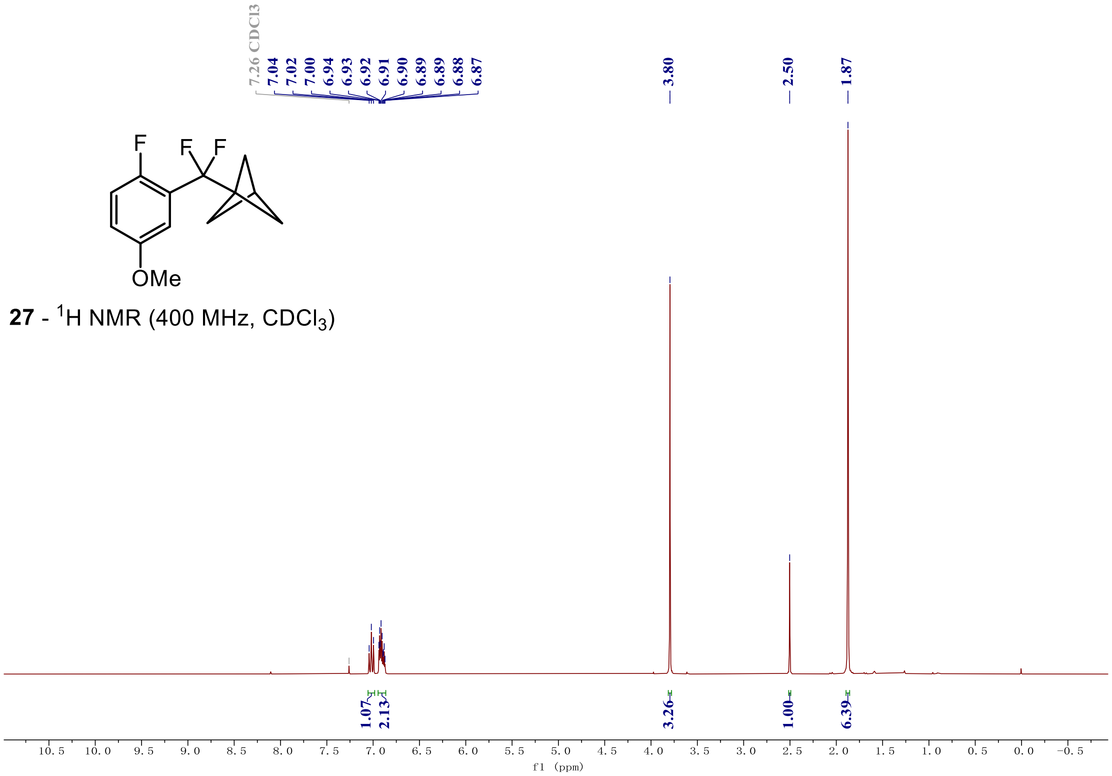

Supplementary Figure 99 ^{1}H NMR spectra of compound **27**

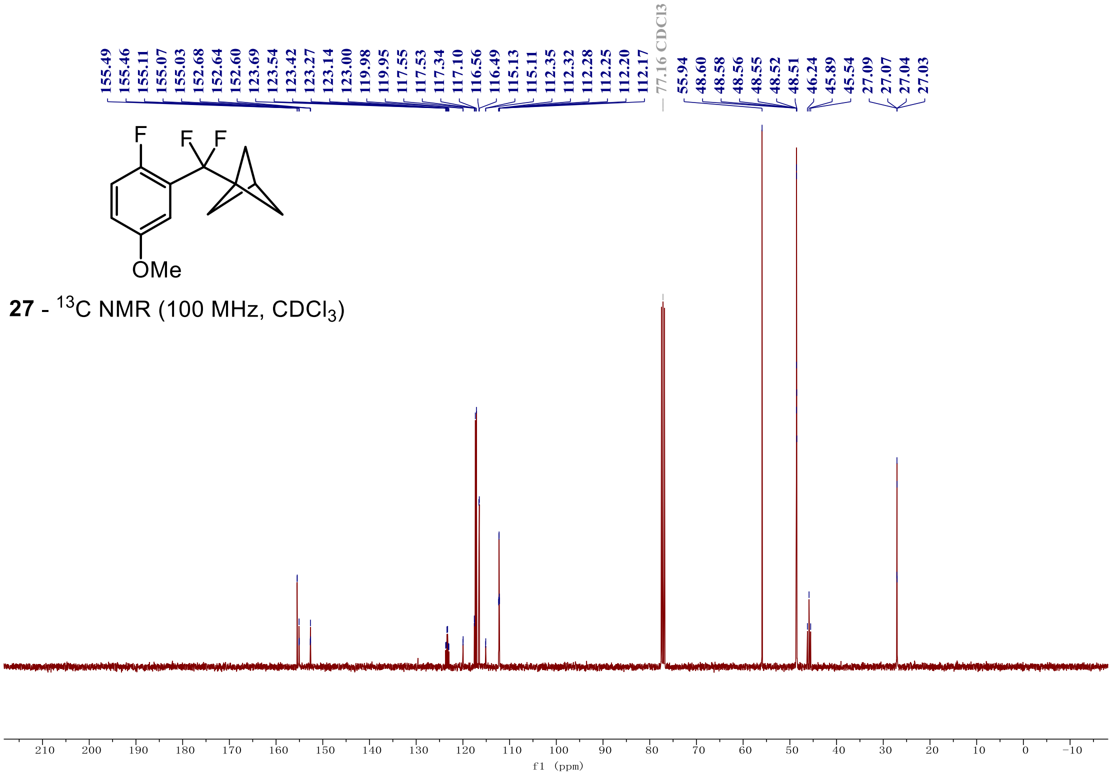

Supplementary Figure 100 ^{13}C NMR spectra of compound **27**

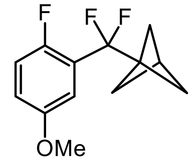

27 - ^{19}F NMR (376 MHz, $CDCl_3$)

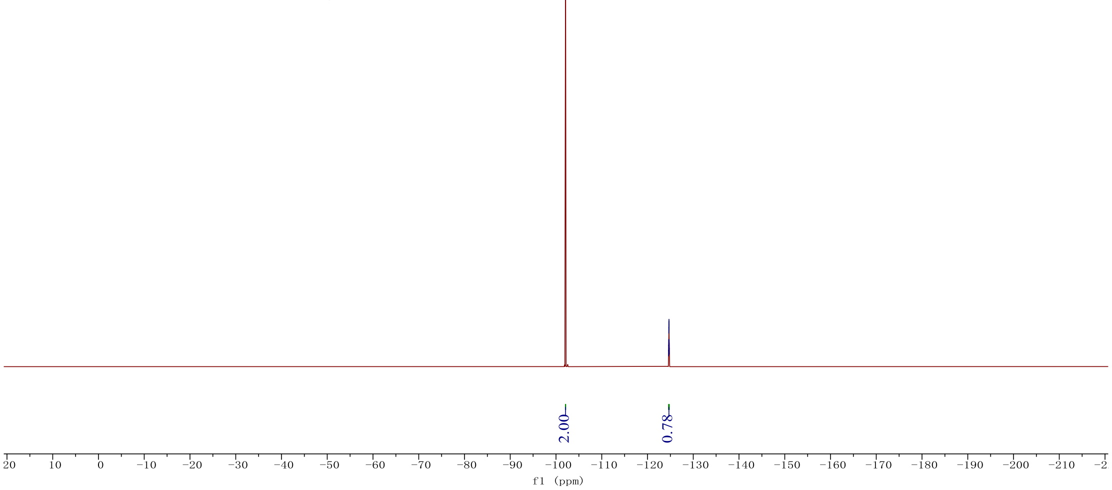

Supplementary Figure 101 ^{19}F NMR spectra of compound **27**

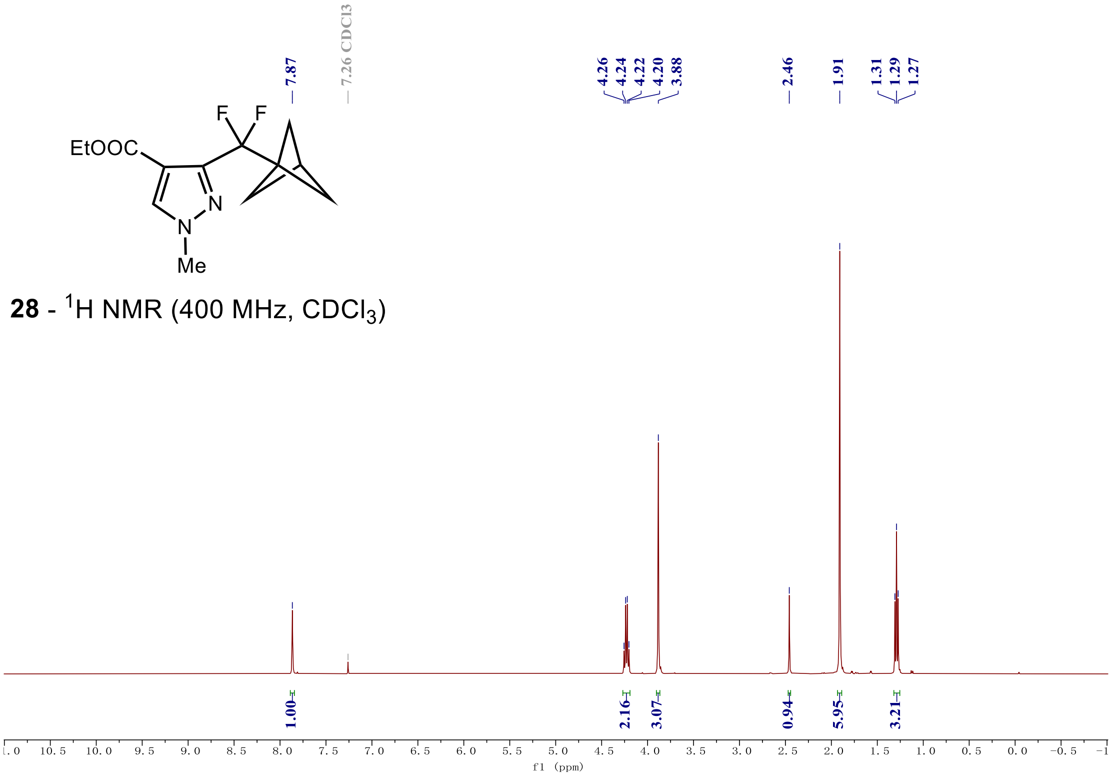

Supplementary Figure 102 ^{1}H NMR spectra of compound **28**

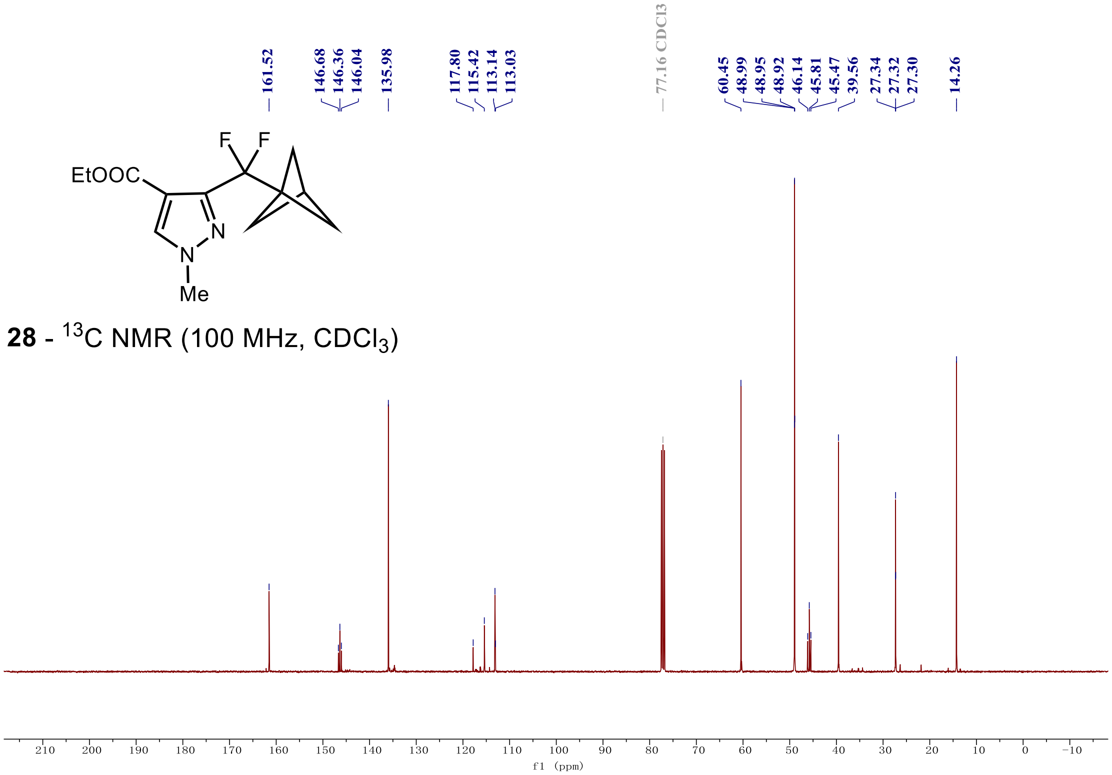

Supplementary Figure 103 ^{13}C NMR spectra of compound **28**

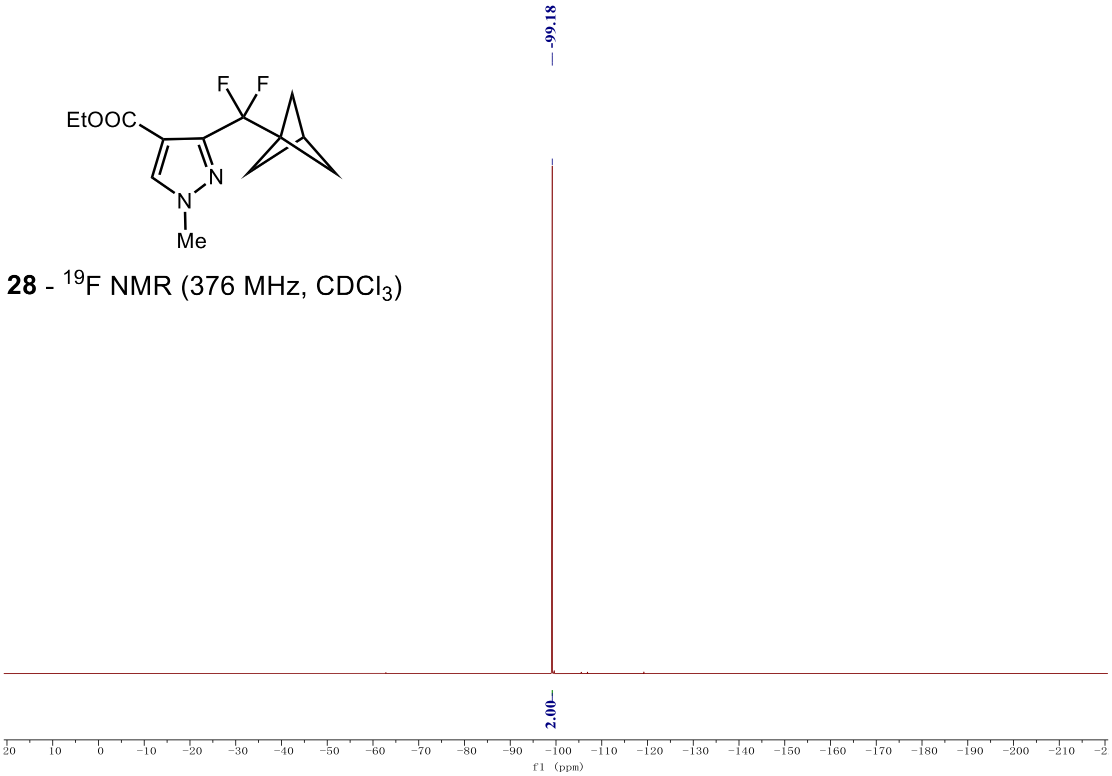

Supplementary Figure 104 ^{19}F NMR spectra of compound **28**

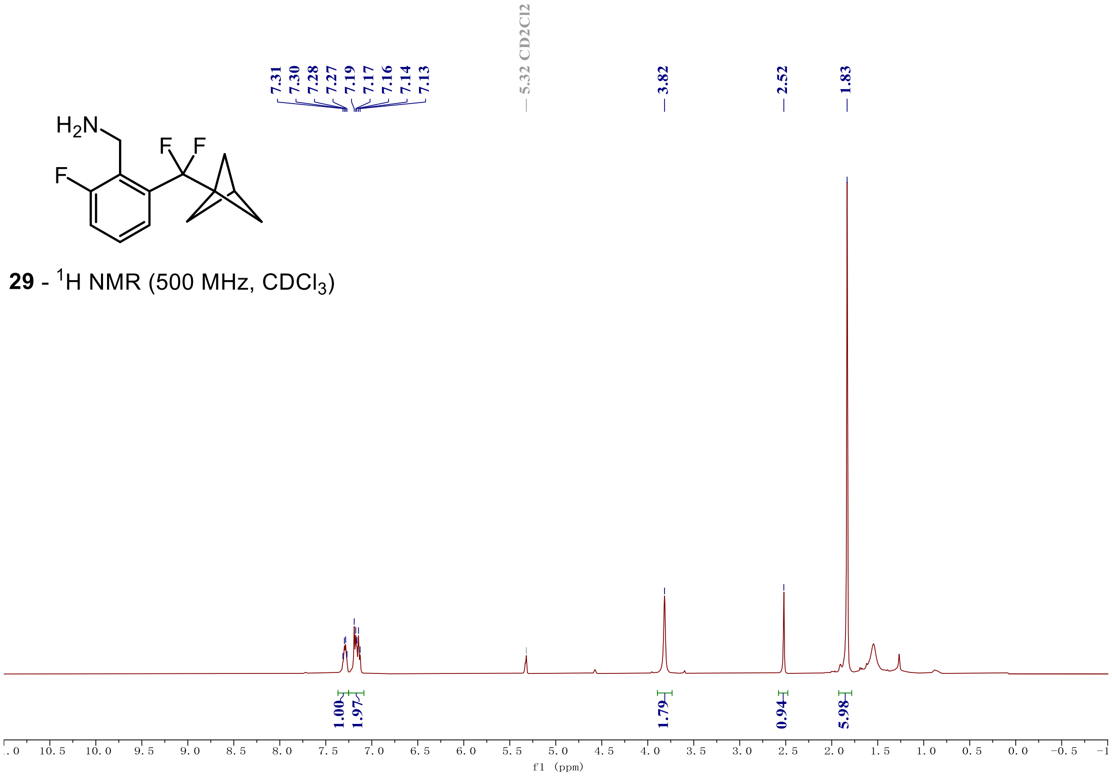

Supplementary Figure 105 ^{1}H NMR spectra of compound **29**

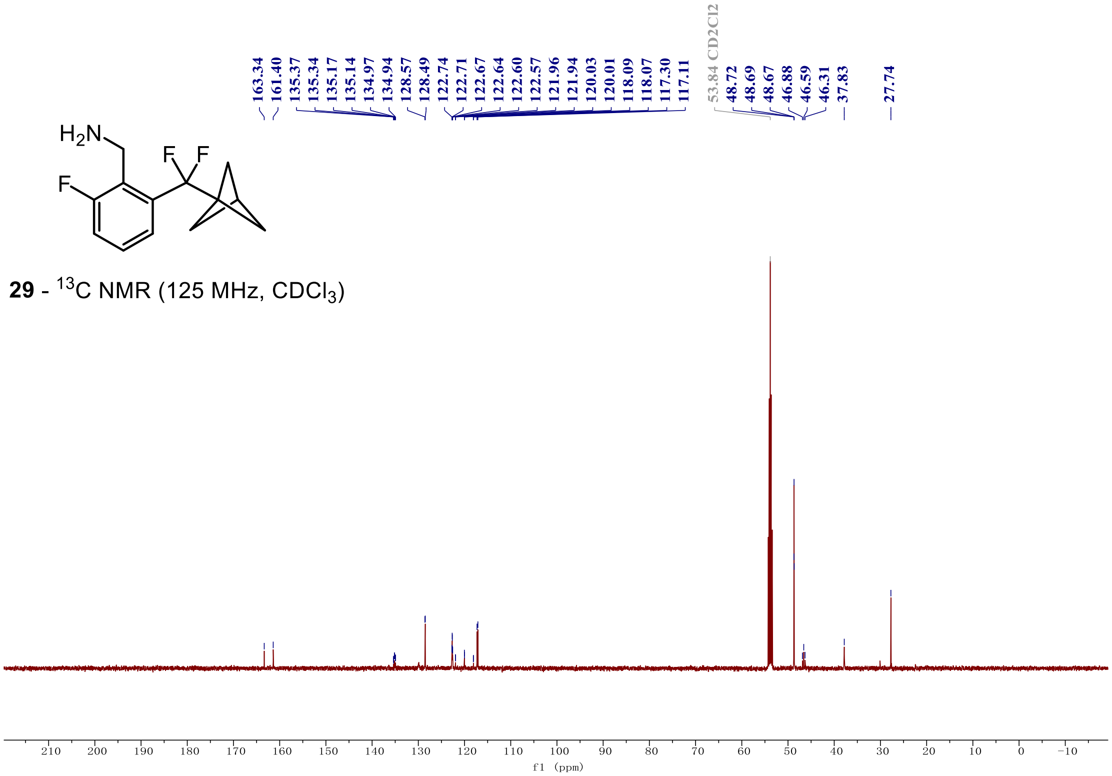

Supplementary Figure 106 ^{13}C NMR spectra of compound **29**

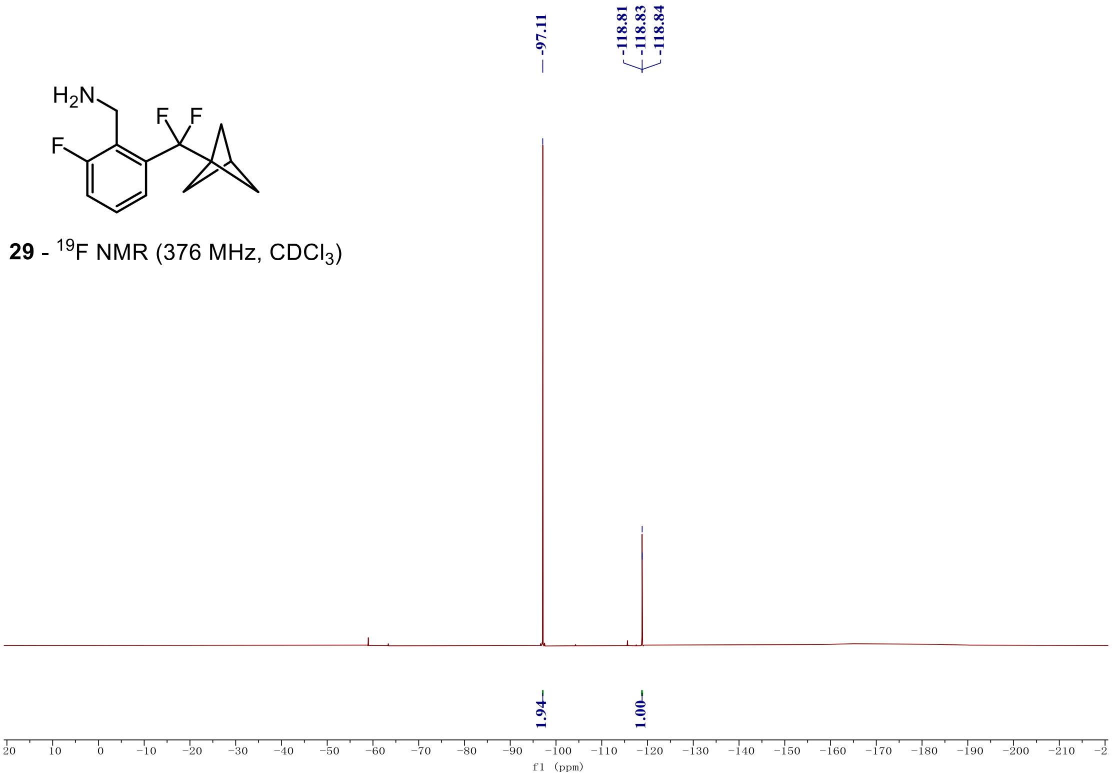

Supplementary Figure 107 ^{19}F NMR spectra of compound **29**

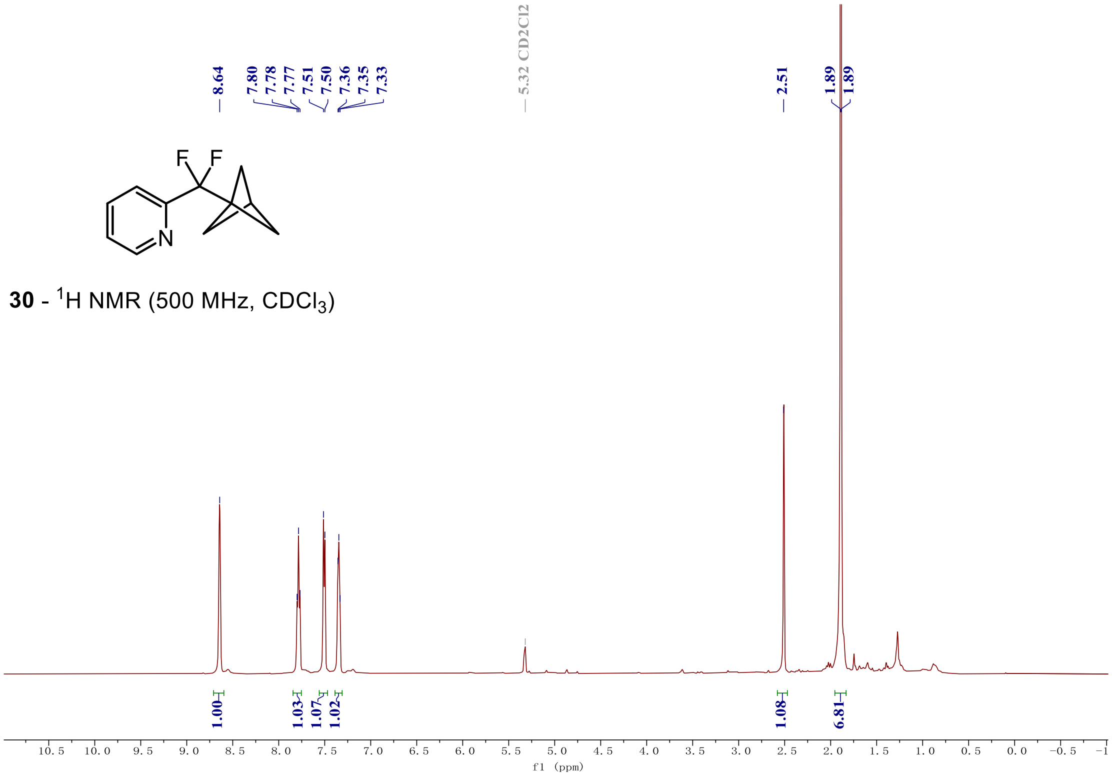

Supplementary Figure 108 ^{1}H NMR spectra of compound **30**

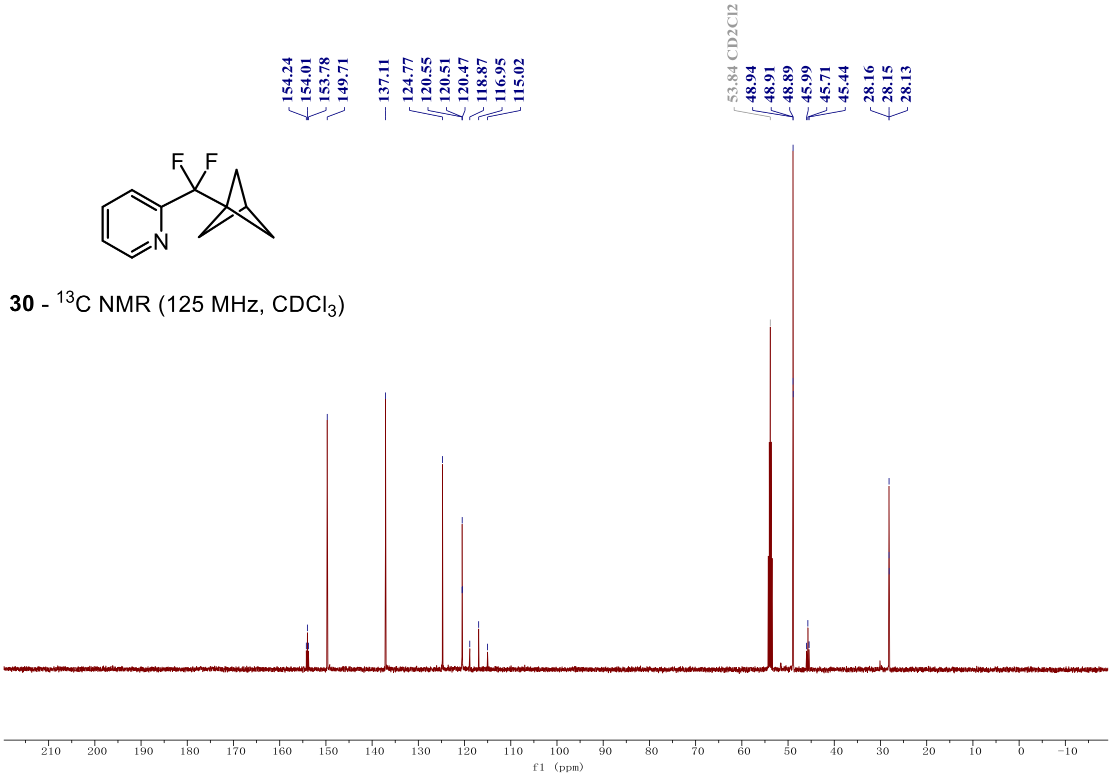

Supplementary Figure 109 ^{13}C NMR spectra of compound **30**

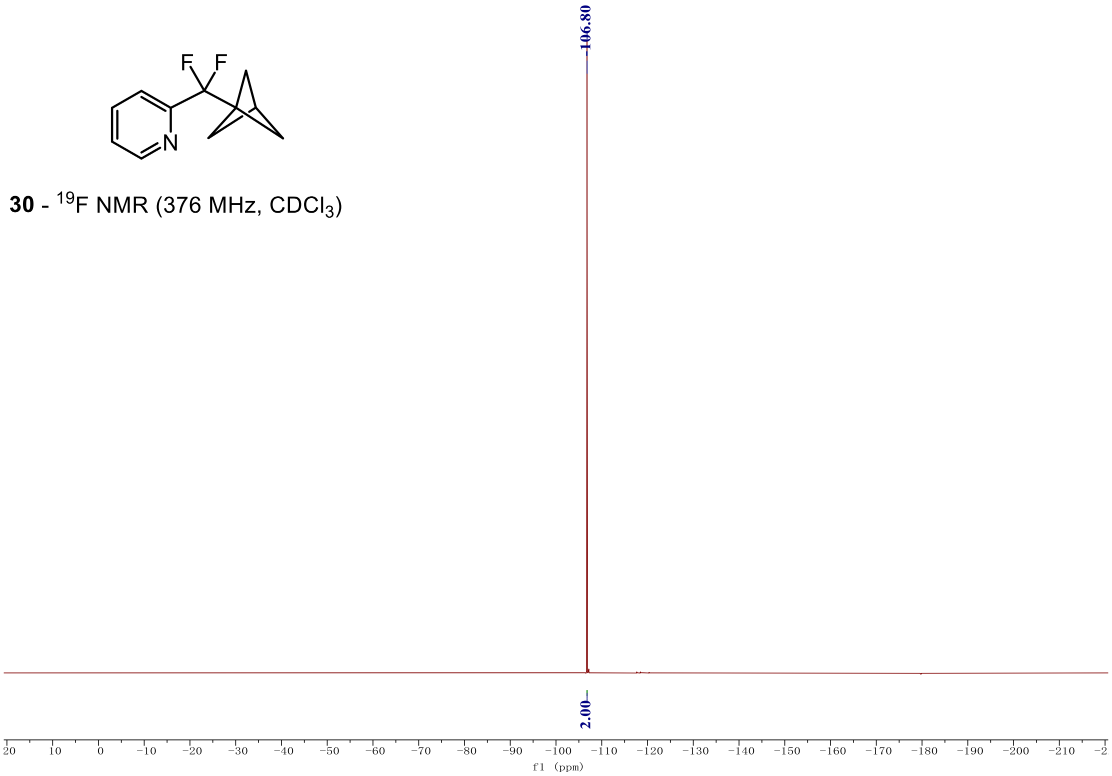

Supplementary Figure 110 ^{19}F NMR spectra of compound **30**

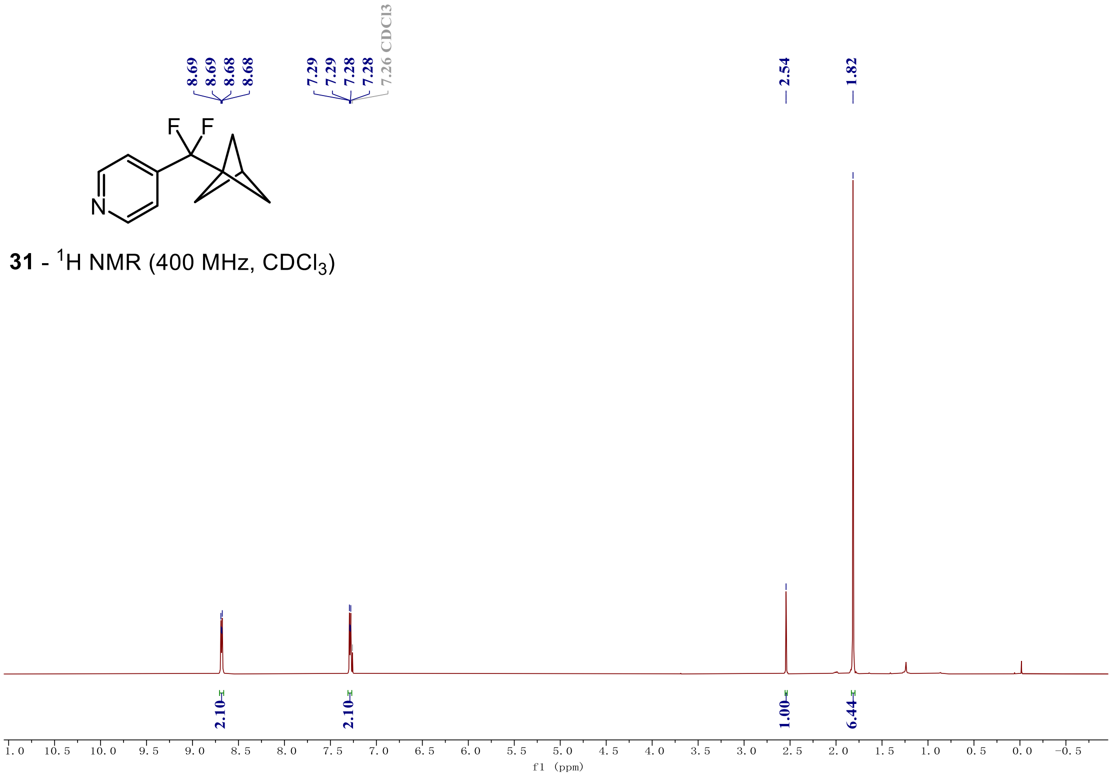

Supplementary Figure 111 ^{1}H NMR spectra of compound **31**

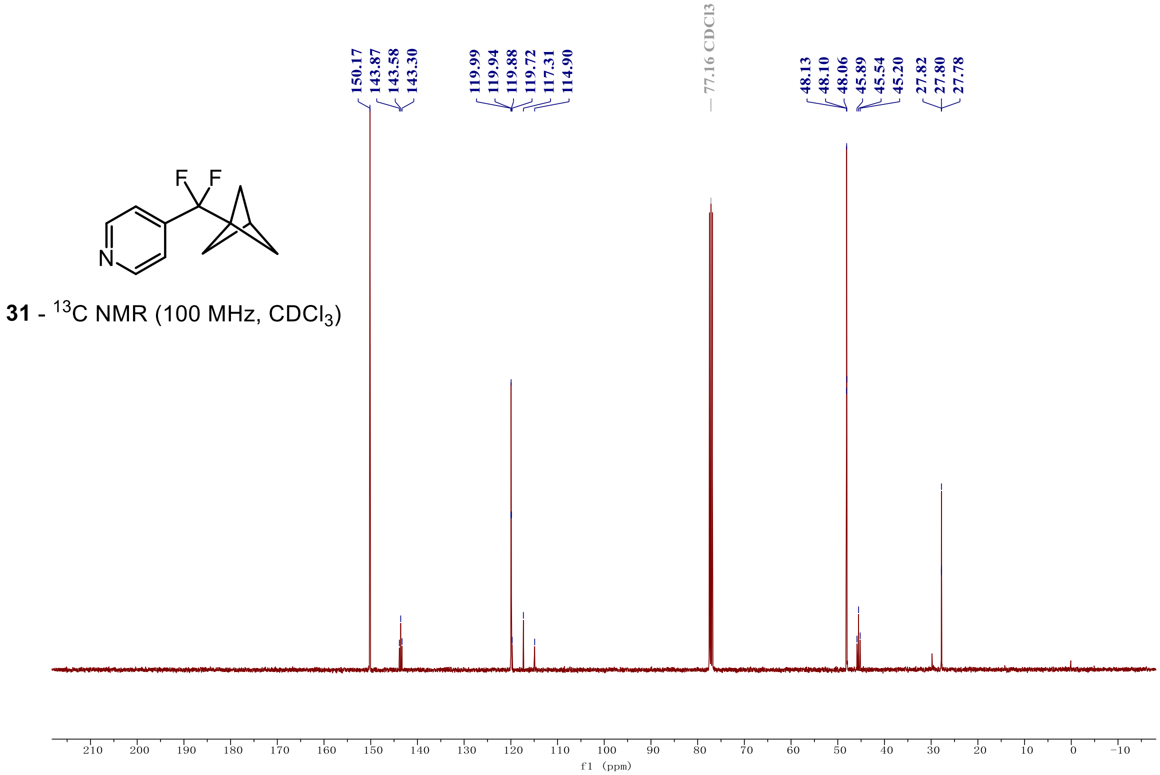

Supplementary Figure 112 ^{13}C NMR spectra of compound **31**

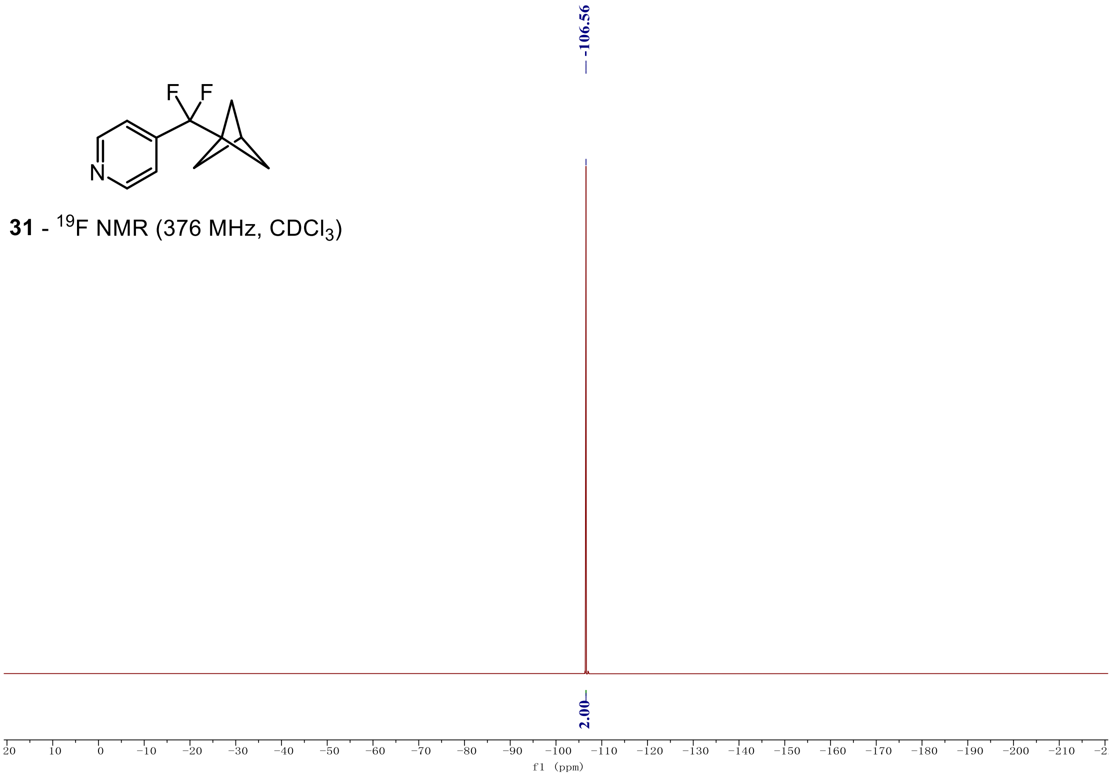

Supplementary Figure 113 ^{19}F NMR spectra of compound **31**

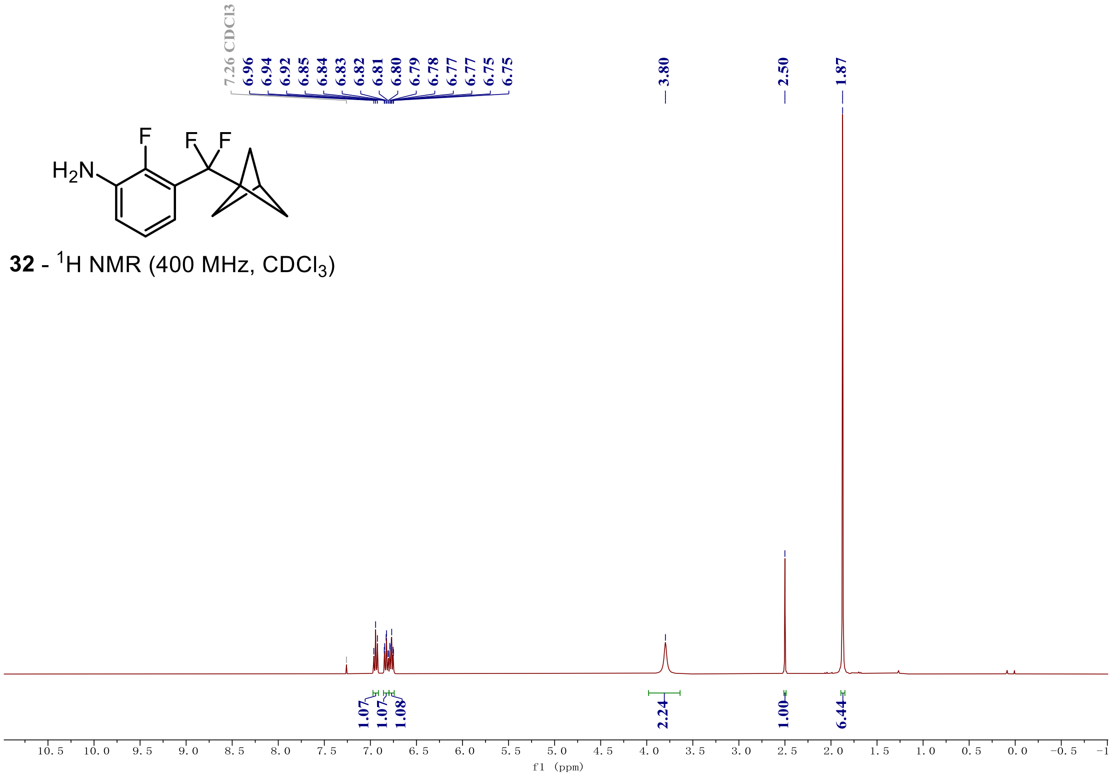

Supplementary Figure 114 ^1H NMR spectra of compound **32**

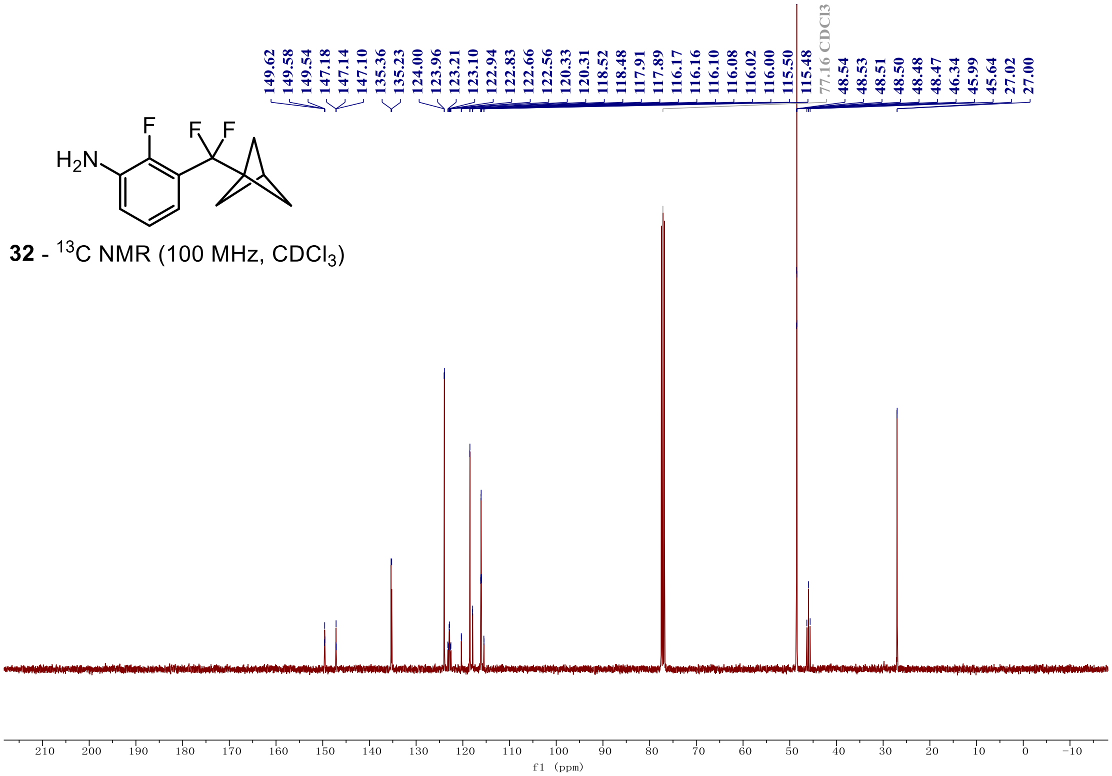

Supplementary Figure 115 ^{13}C NMR spectra of compound **32**

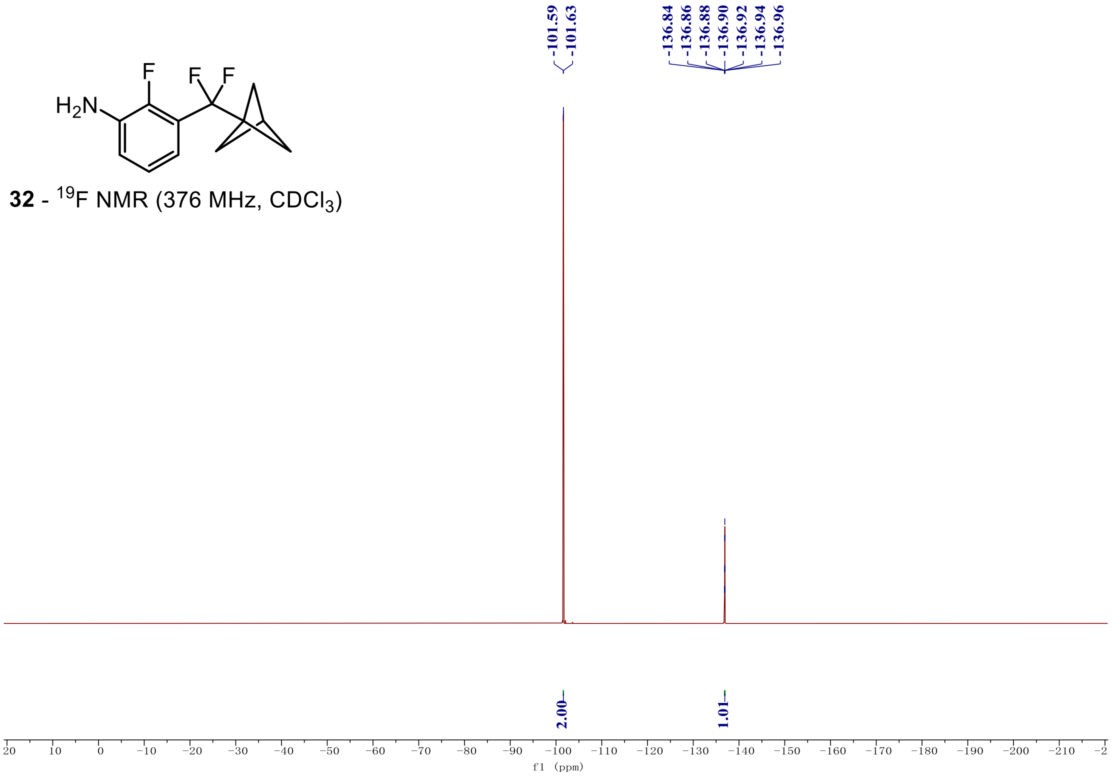

Supplementary Figure 116 ^{19}F NMR spectra of compound **32**

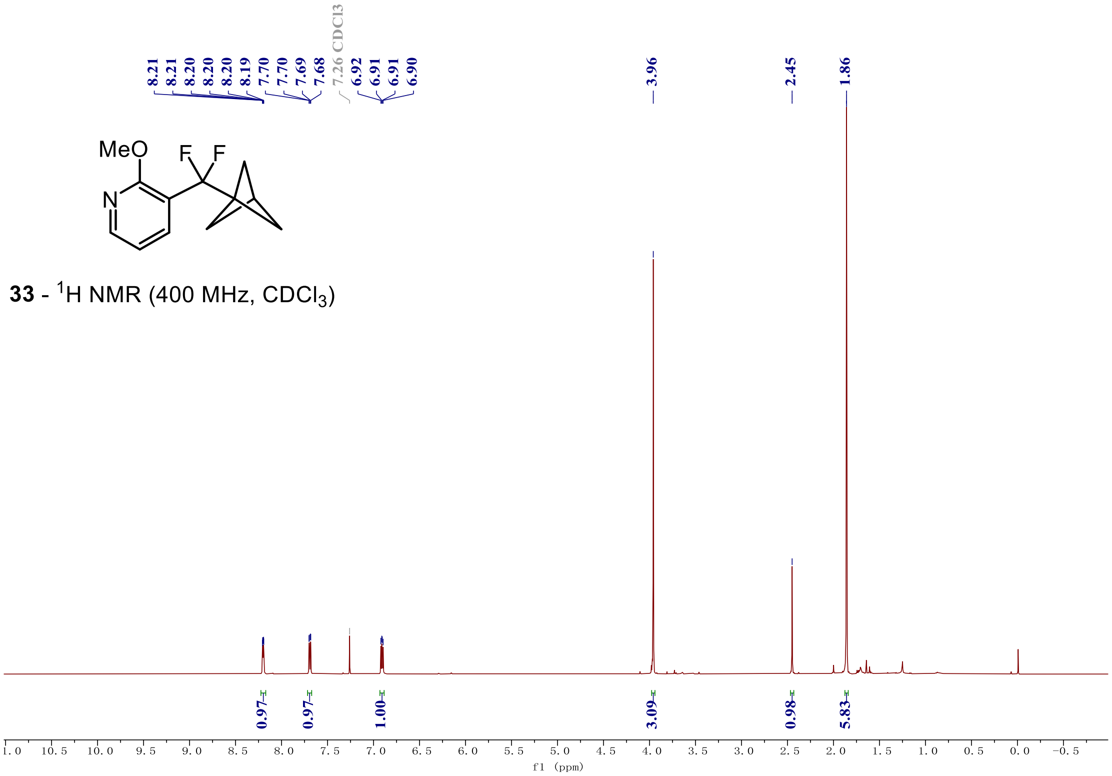

Supplementary Figure 117 ^{1}H NMR spectra of compound **33**

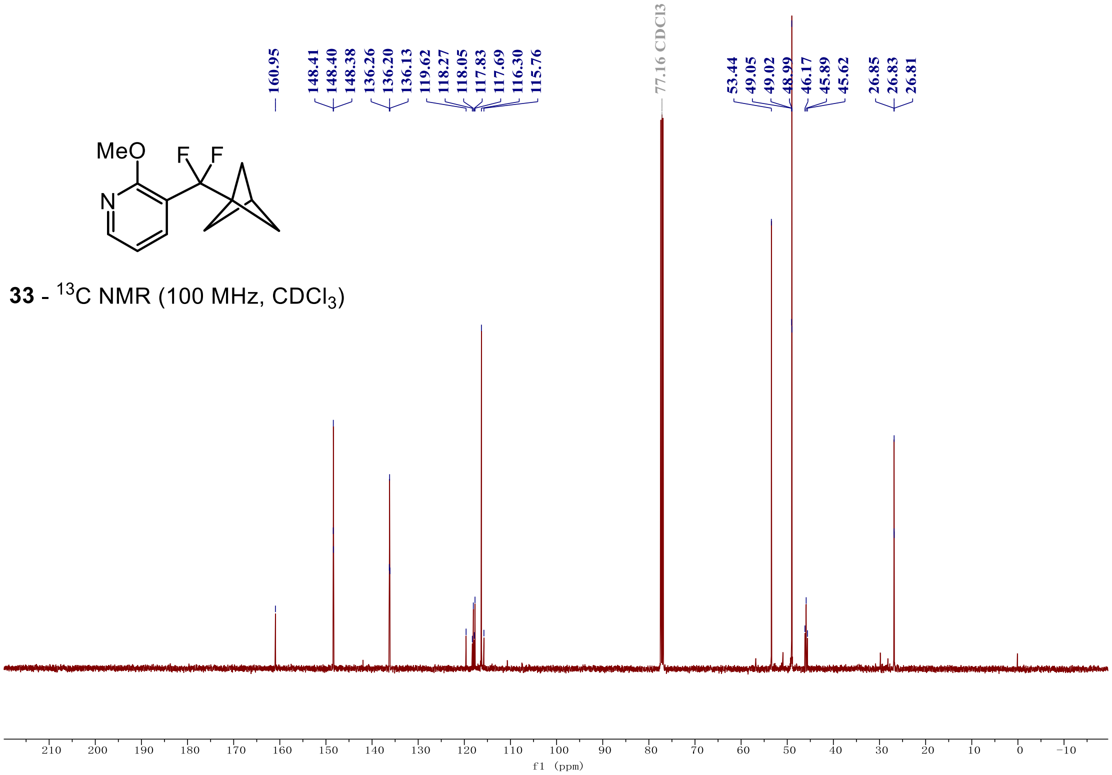

Supplementary Figure 118 ^{13}C NMR spectra of compound **33**

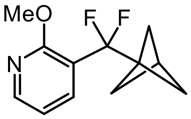

33 - ^{19}F NMR (376 MHz, $CDCl_3$)

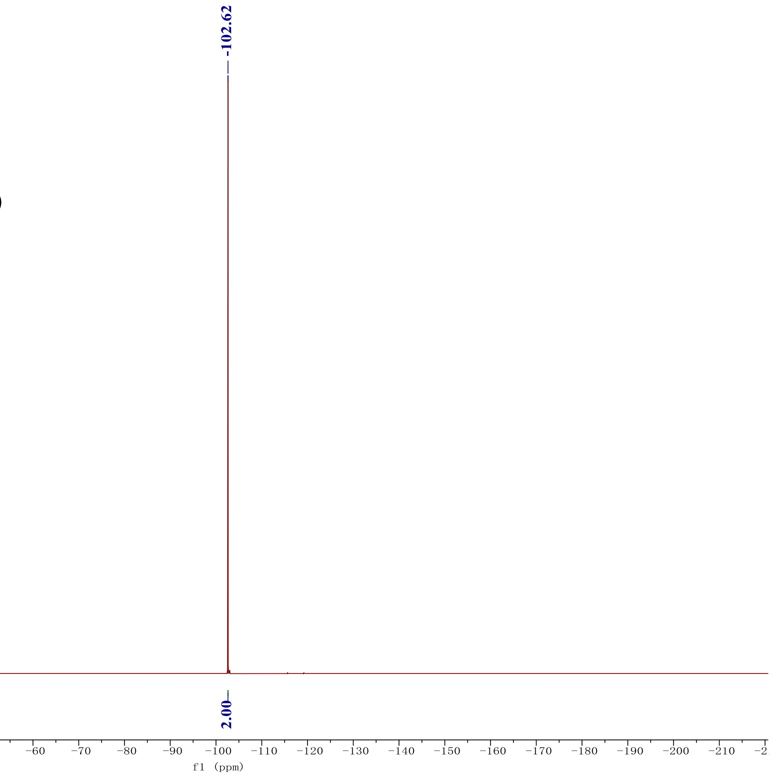

Supplementary Figure 119 ^{19}F NMR spectra of compound **33**

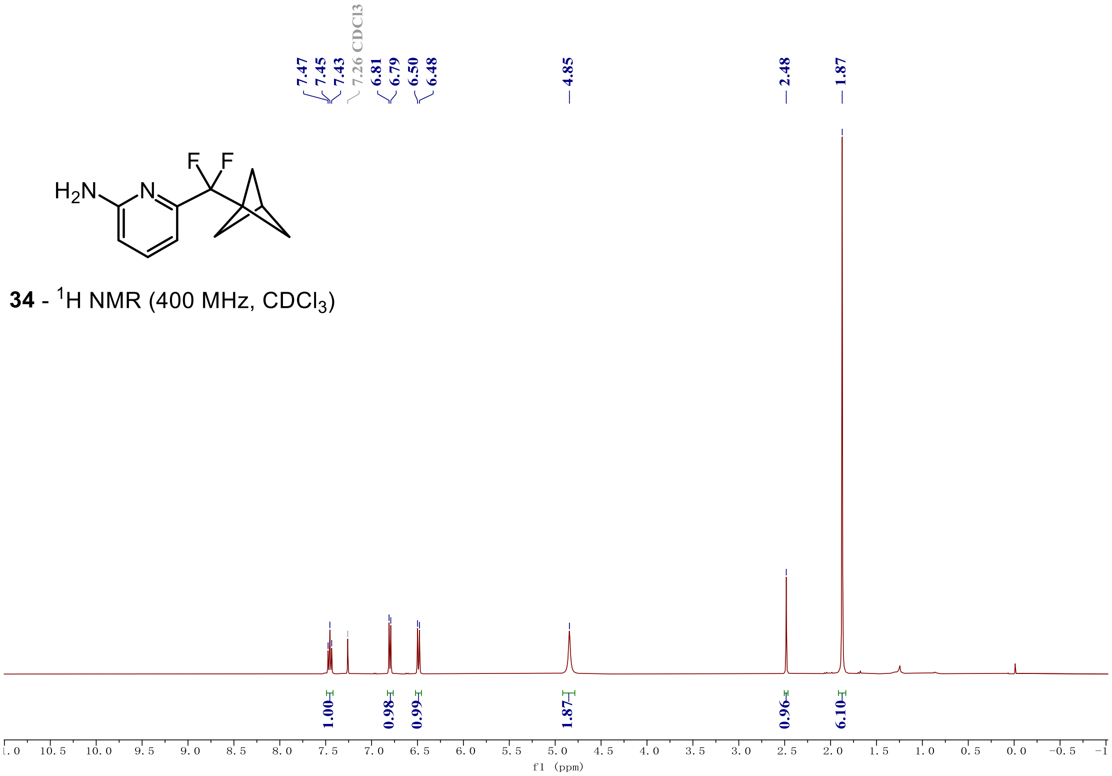

Supplementary Figure 120 ^{1}H NMR spectra of compound **34**

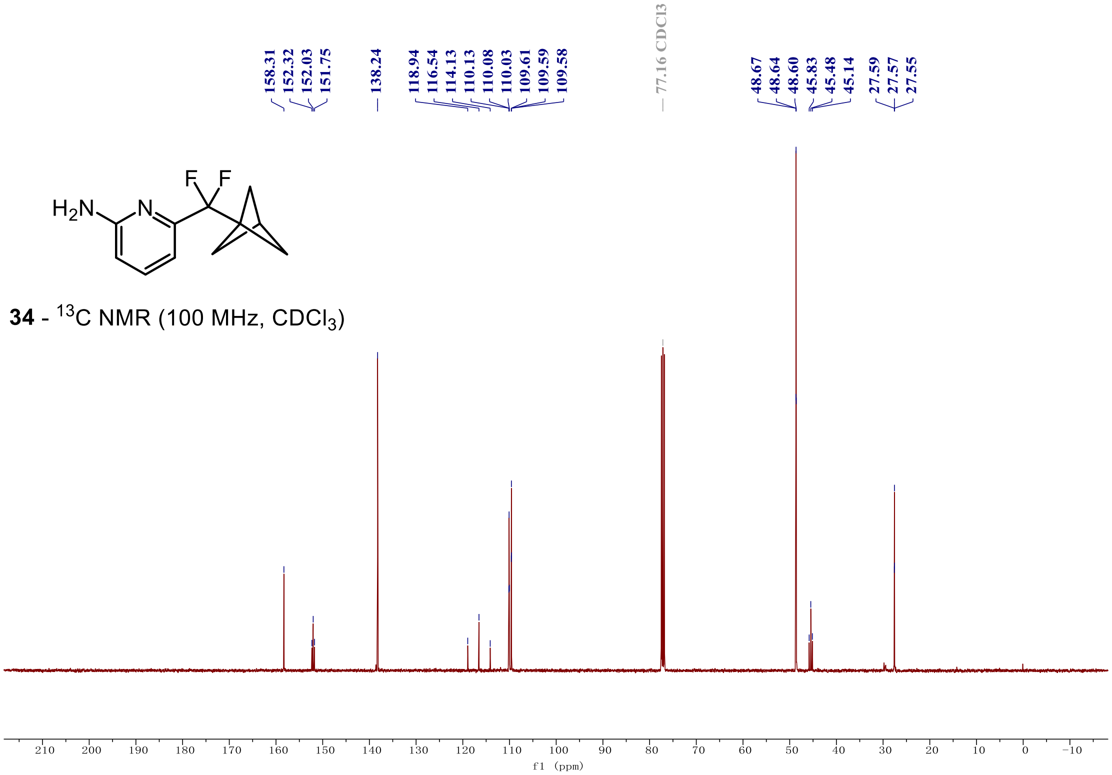

Supplementary Figure 121 ^{13}C NMR spectra of compound **34**

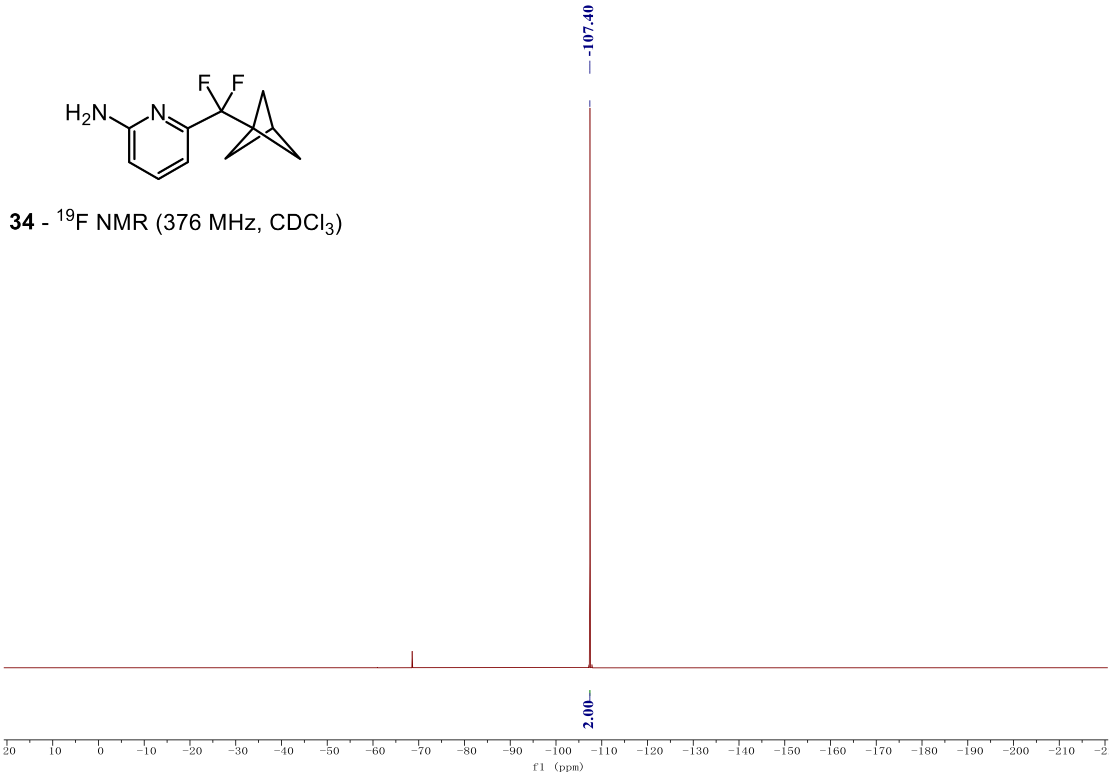

Supplementary Figure 122 ^{19}F NMR spectra of compound **34**

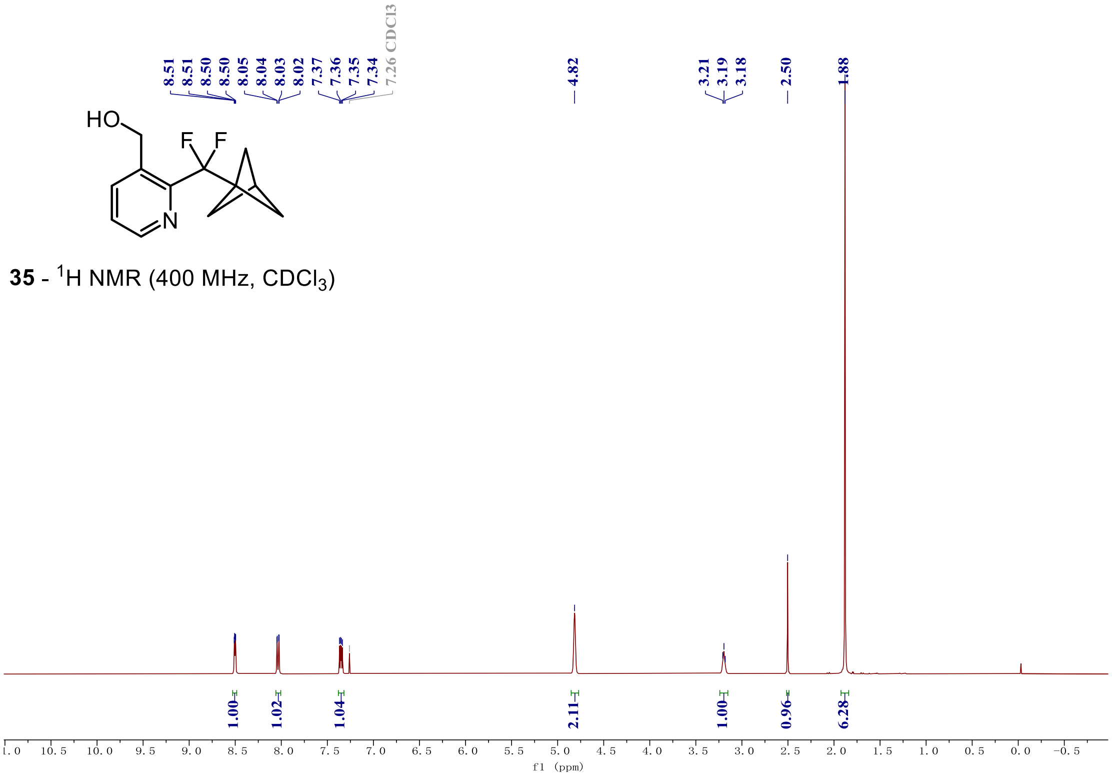

Supplementary Figure 123 ^{1}H NMR spectra of compound **35**

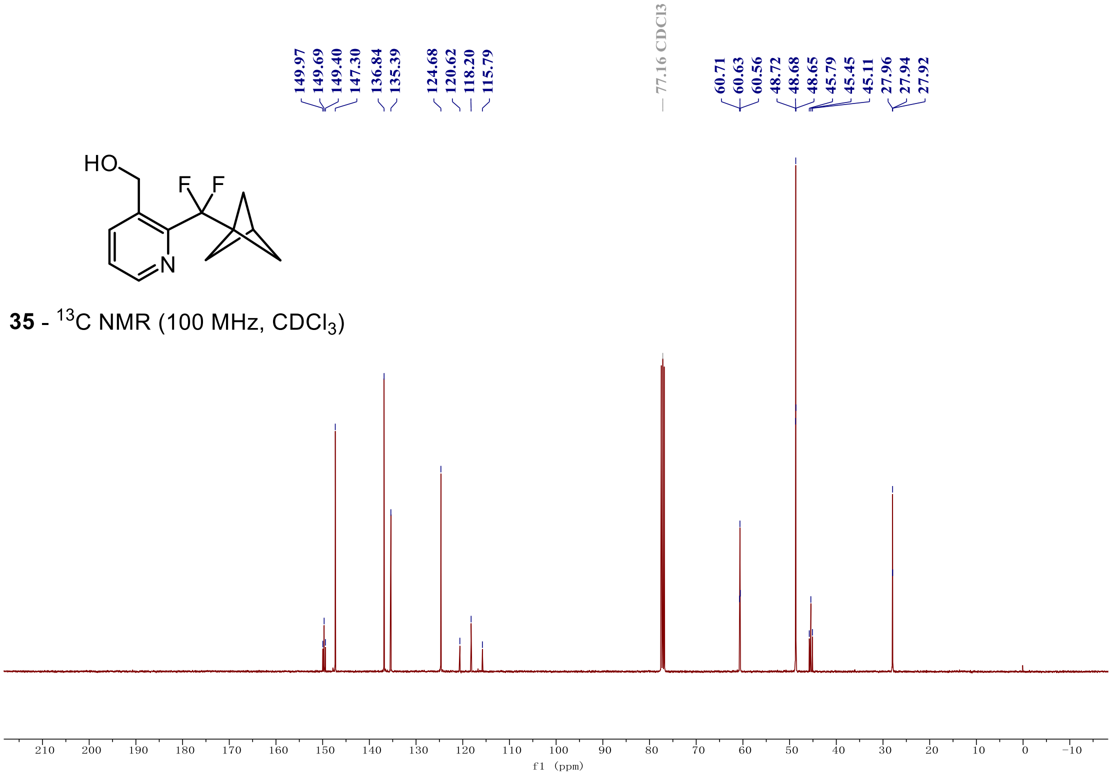

Supplementary Figure 124 ^{13}C NMR spectra of compound **35**

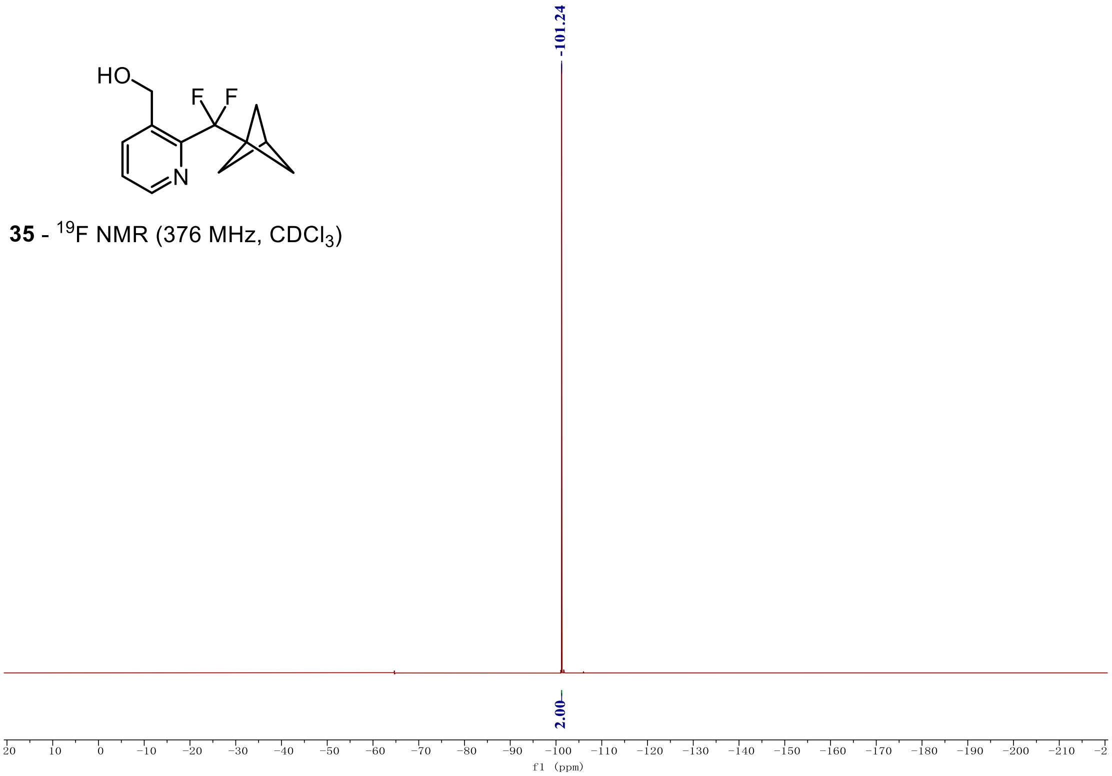

Supplementary Figure 125 ^{19}F NMR spectra of compound **35**

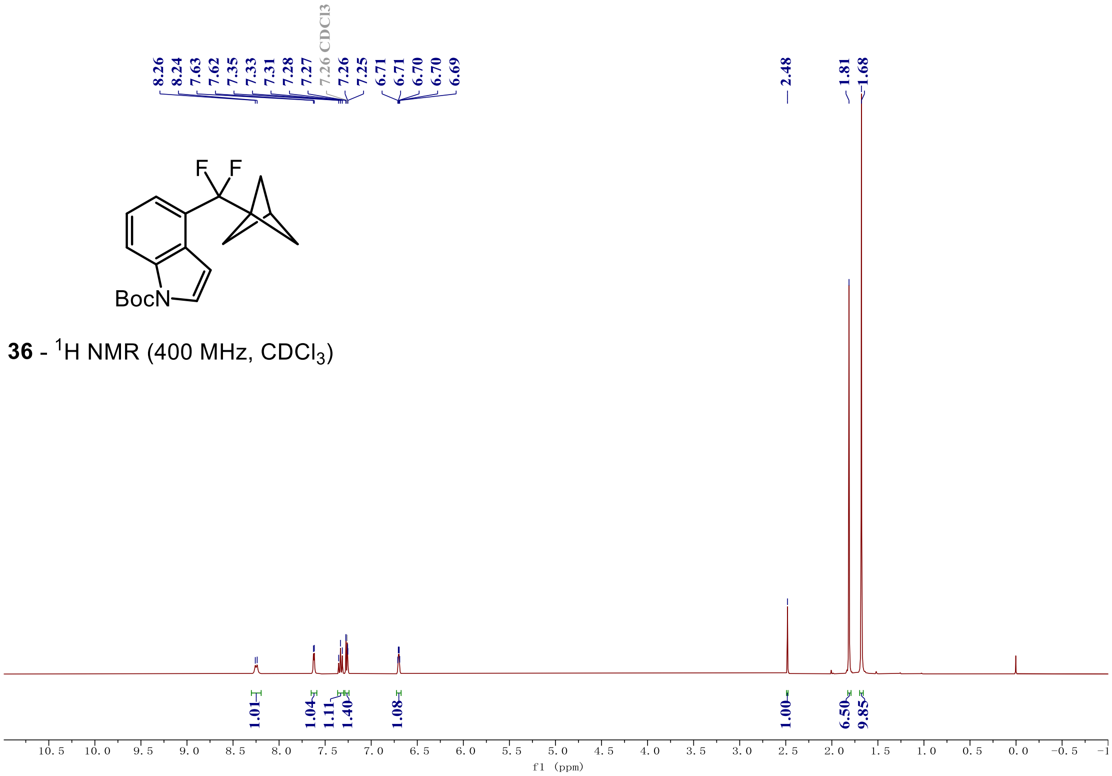

Supplementary Figure 126 ^{1}H NMR spectra of compound **36**

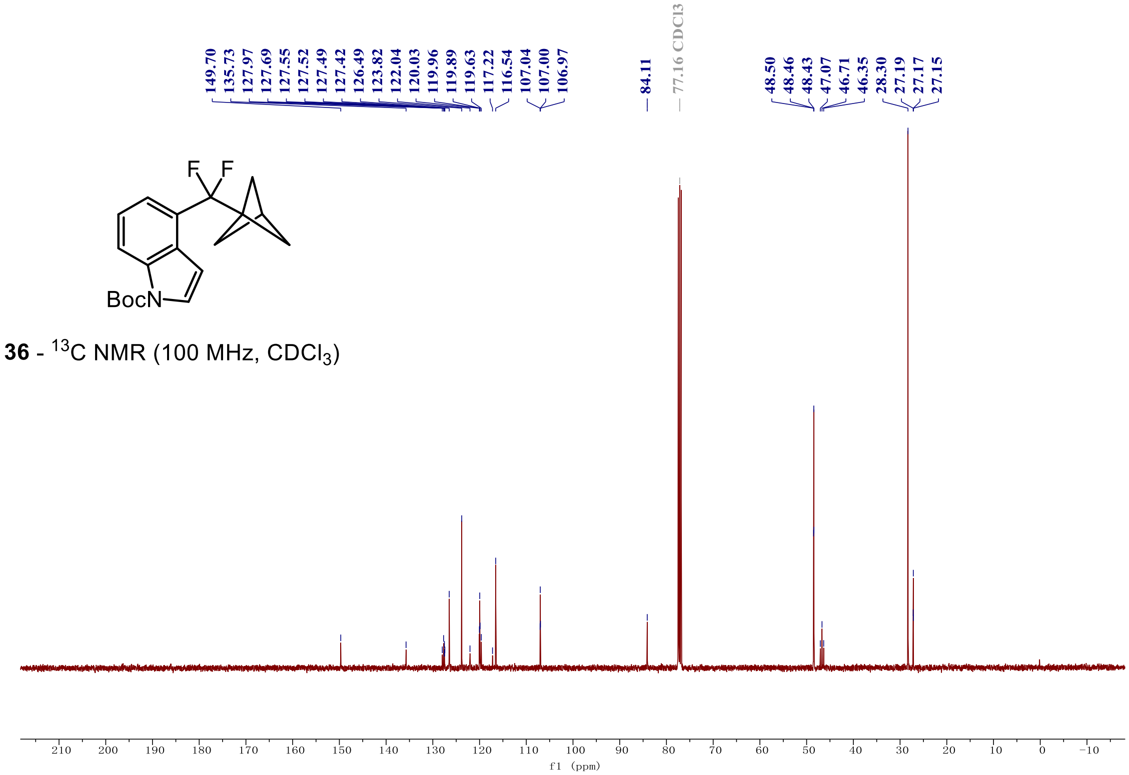

Supplementary Figure 127 ^{13}C NMR spectra of compound **36**

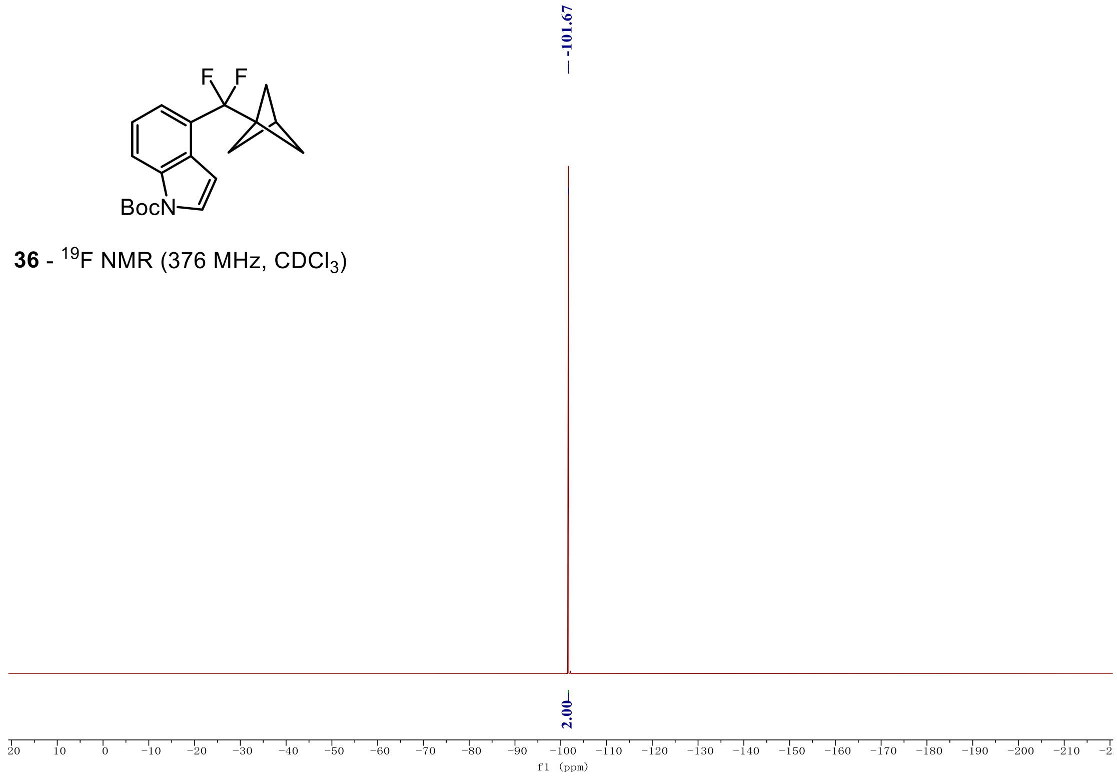

Supplementary Figure 128 ^{19}F NMR spectra of compound **36**

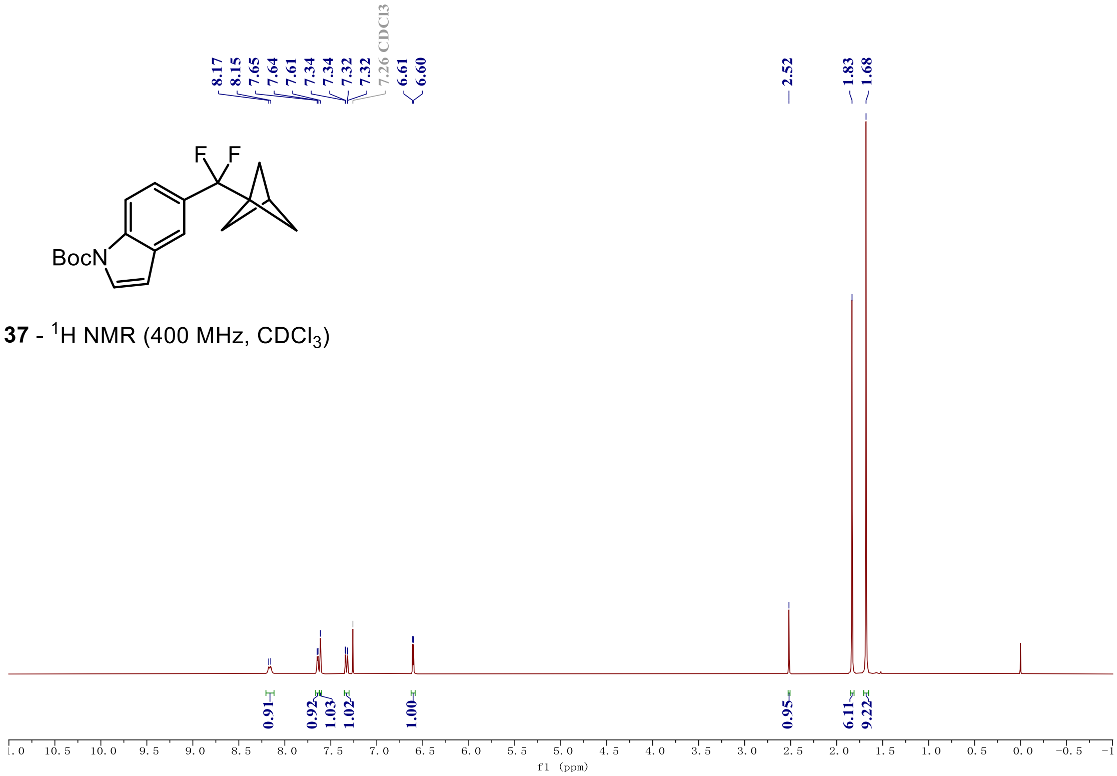

Supplementary Figure 129 ^{1}H NMR spectra of compound **37**

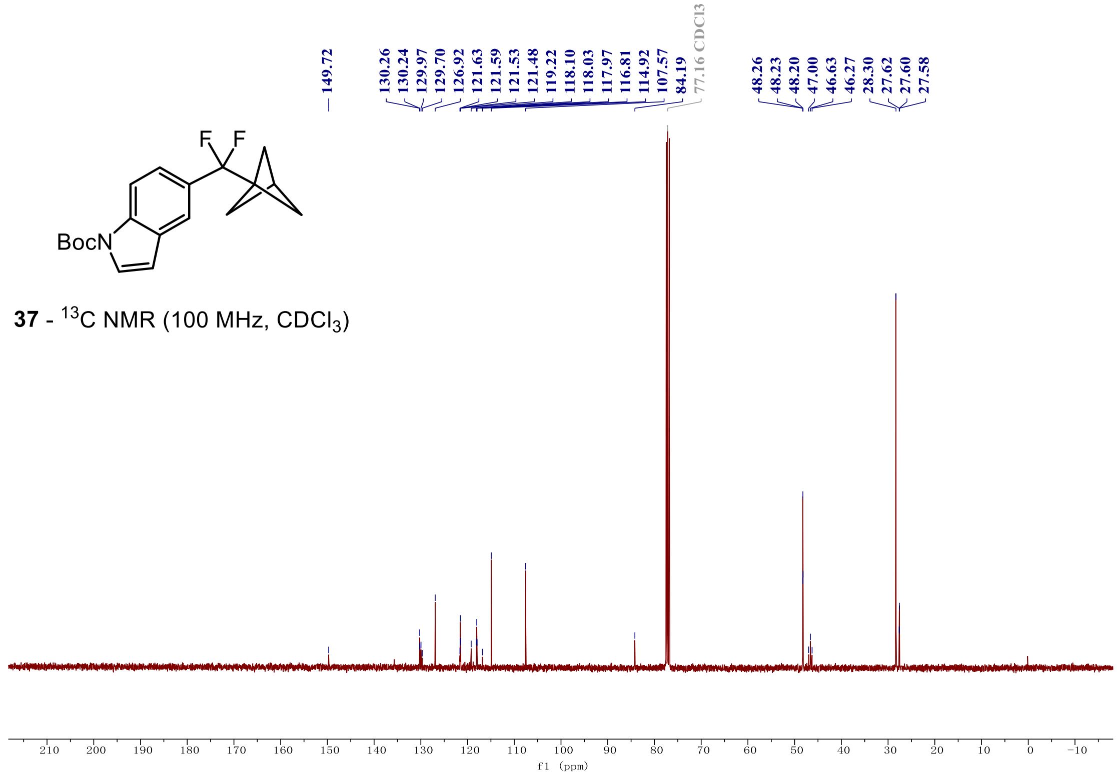

Supplementary Figure 130 ^{13}C NMR spectra of compound **37**

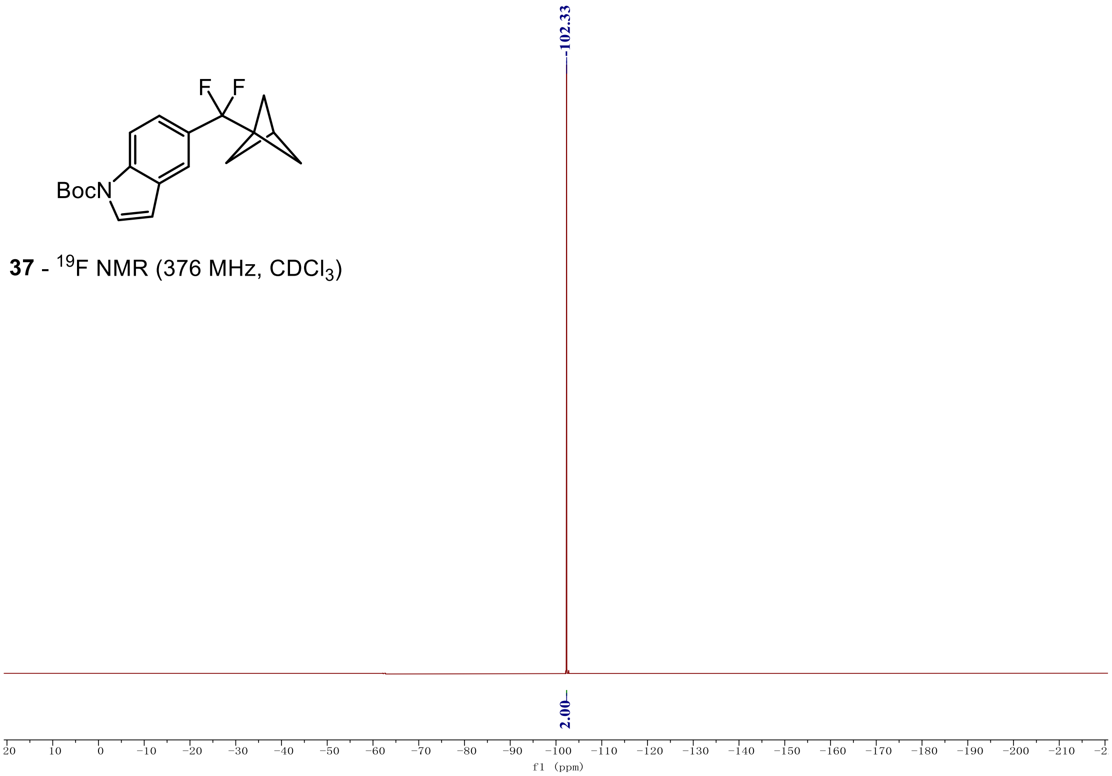

Supplementary Figure 131 ^{19}F NMR spectra of compound **37**

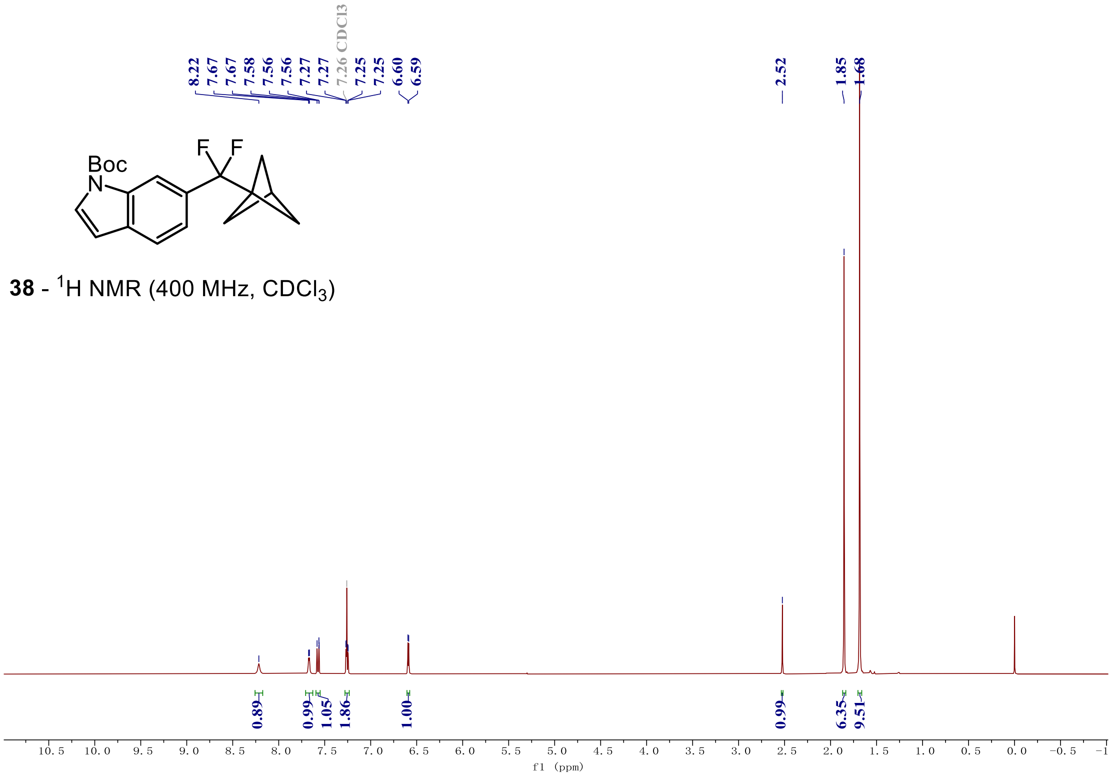

Supplementary Figure 132 ^{1}H NMR spectra of compound **38**

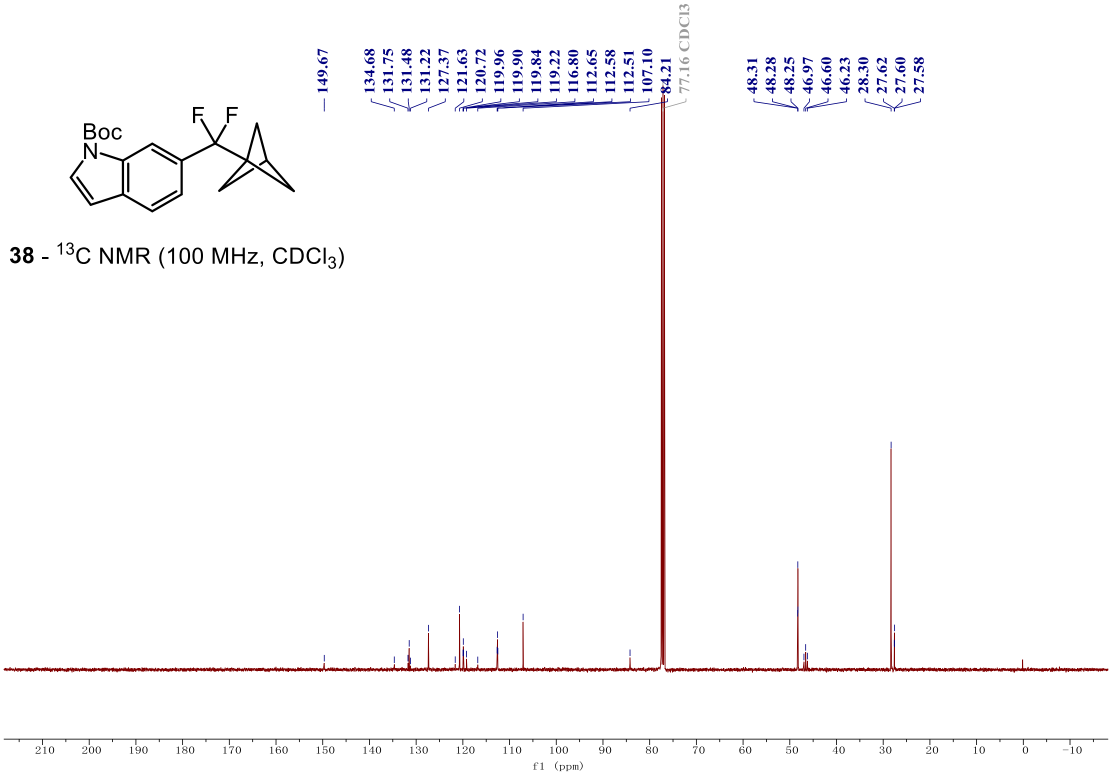

Supplementary Figure 133 ^{13}C NMR spectra of compound **38**

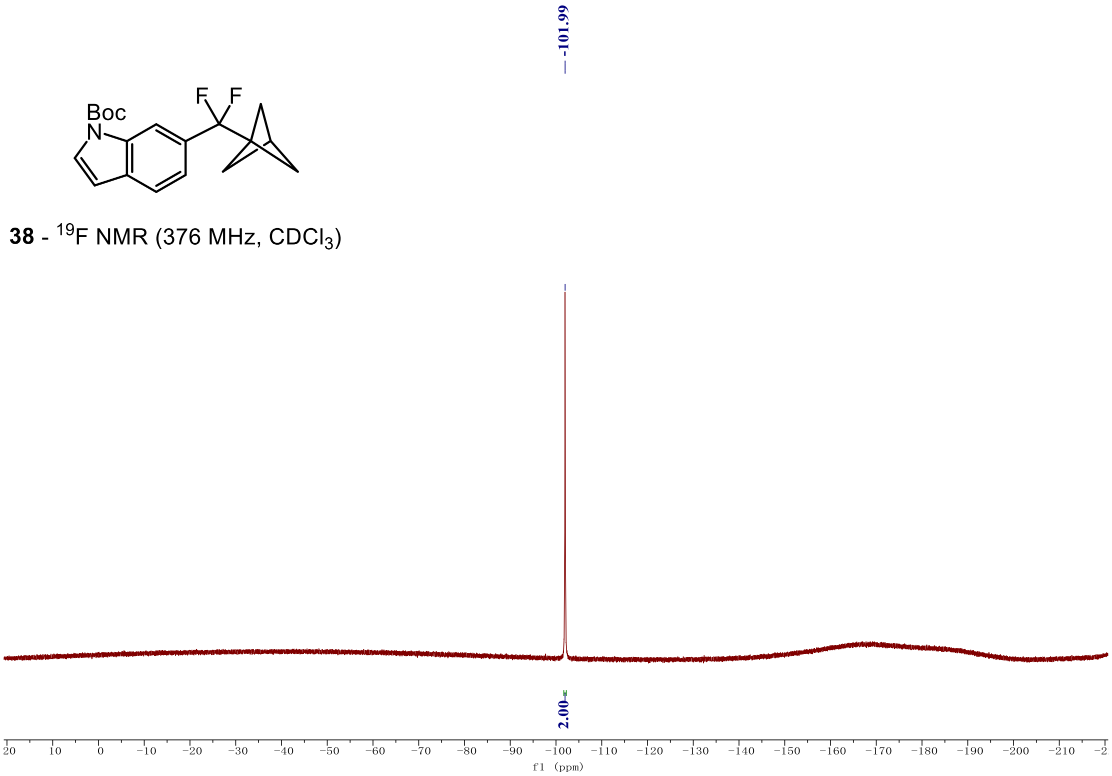

Supplementary Figure 134 ^{19}F NMR spectra of compound **38**

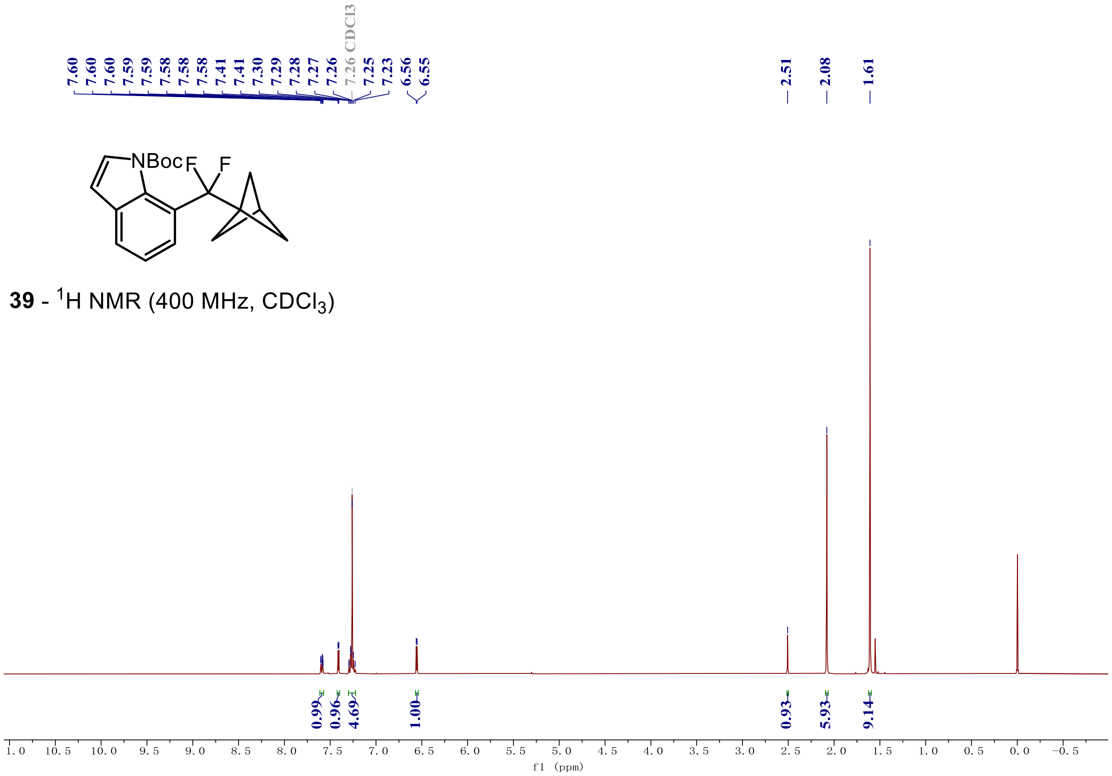

Supplementary Figure 135 ^{1}H NMR spectra of compound **39**

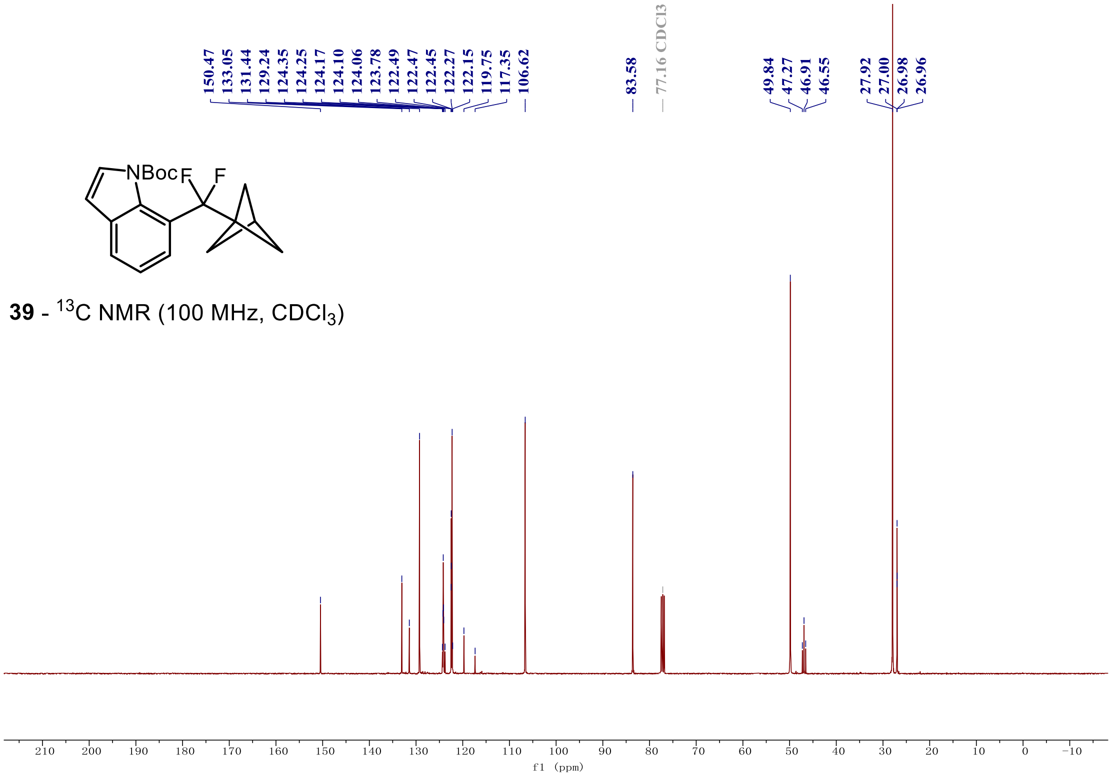

Supplementary Figure 136 ^{13}C NMR spectra of compound **39**

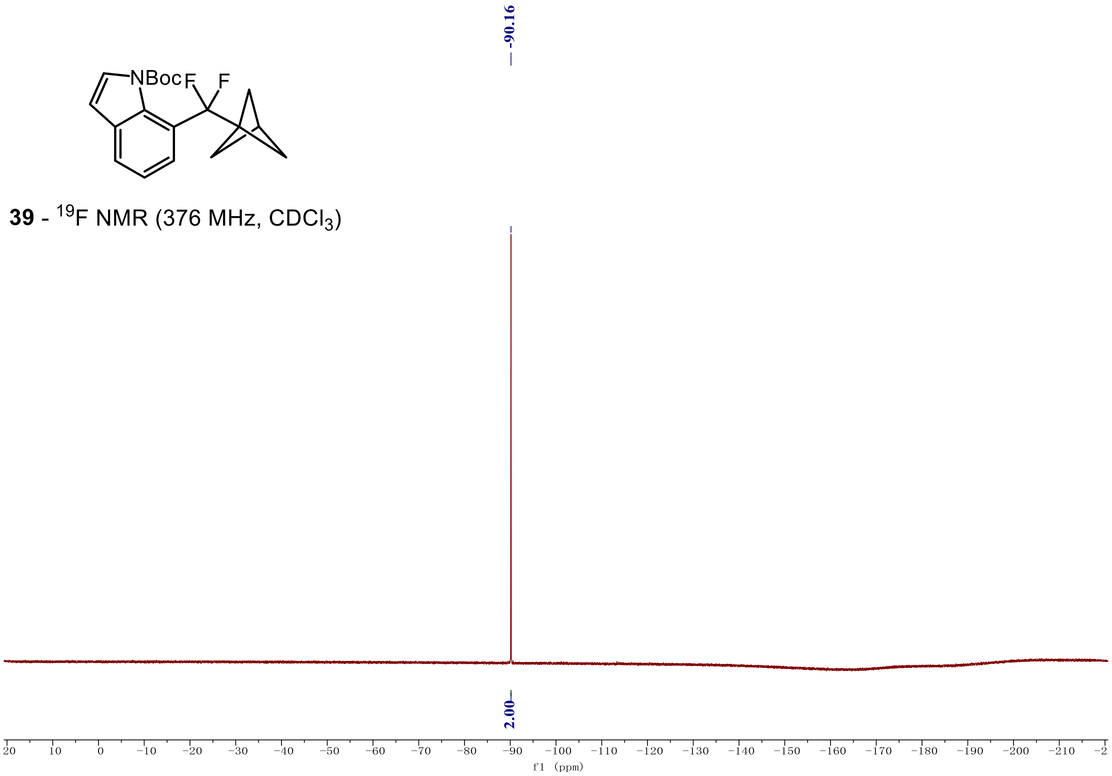

Supplementary Figure 137 ^{19}F NMR spectra of compound **39**

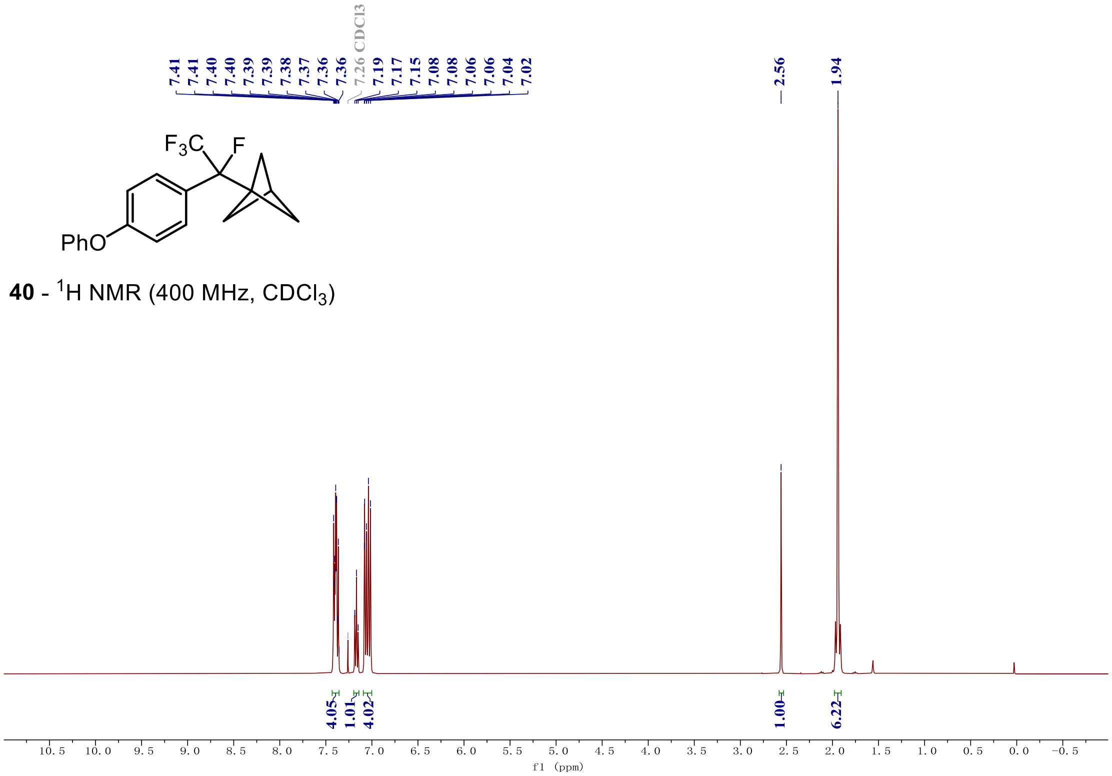

Supplementary Figure 138 ^{1}H NMR spectra of compound **40**

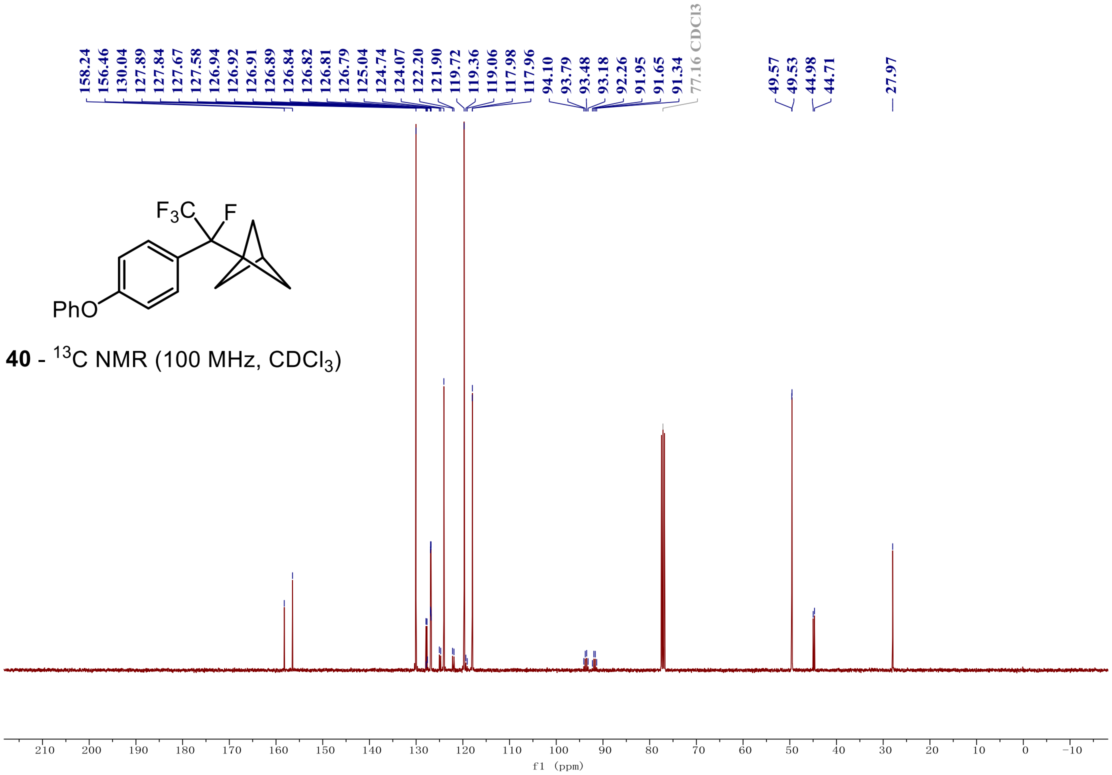

Supplementary Figure 139 ^{13}C NMR spectra of compound **40**

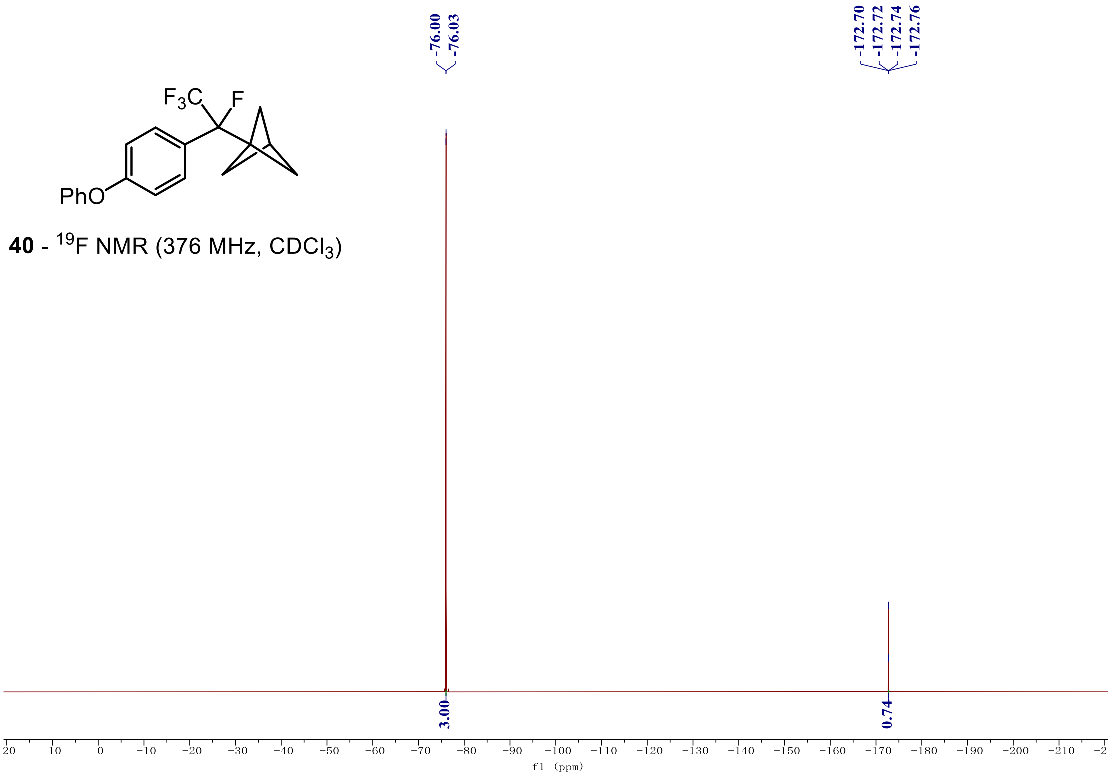

Supplementary Figure 140 ^{19}F NMR spectra of compound **40**

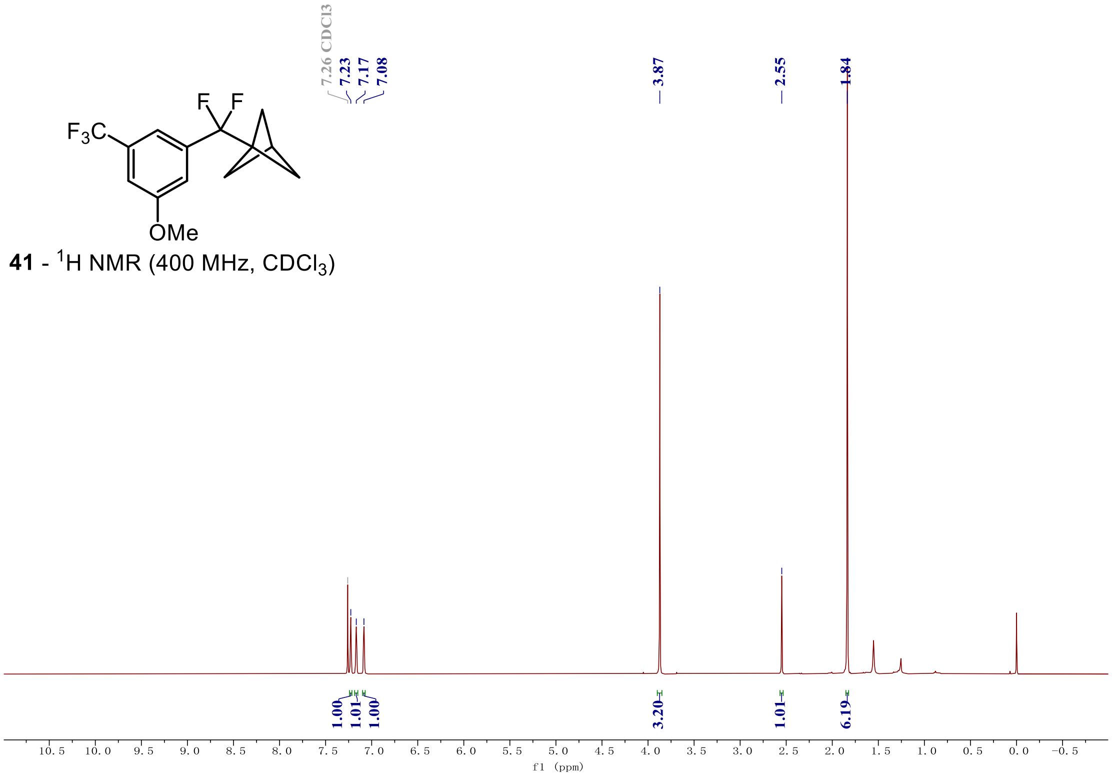

Supplementary Figure 141 ^{1}H NMR spectra of compound **41**

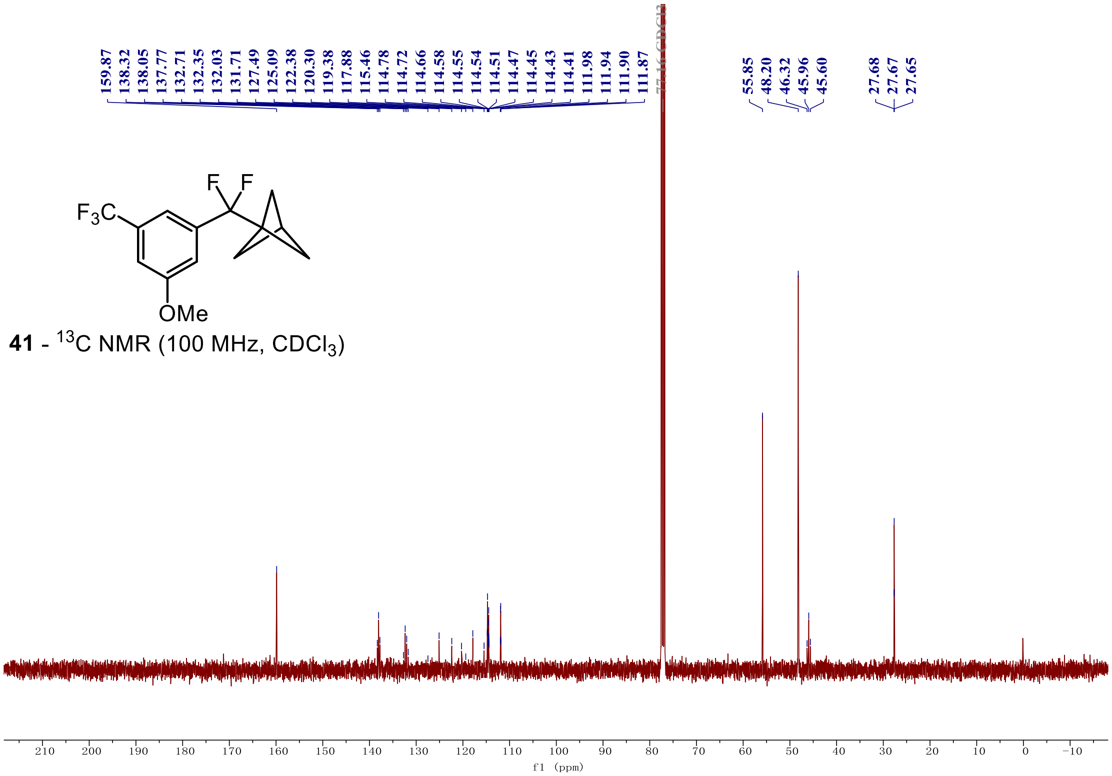

Supplementary Figure 142 ^{13}C NMR spectra of compound **41**

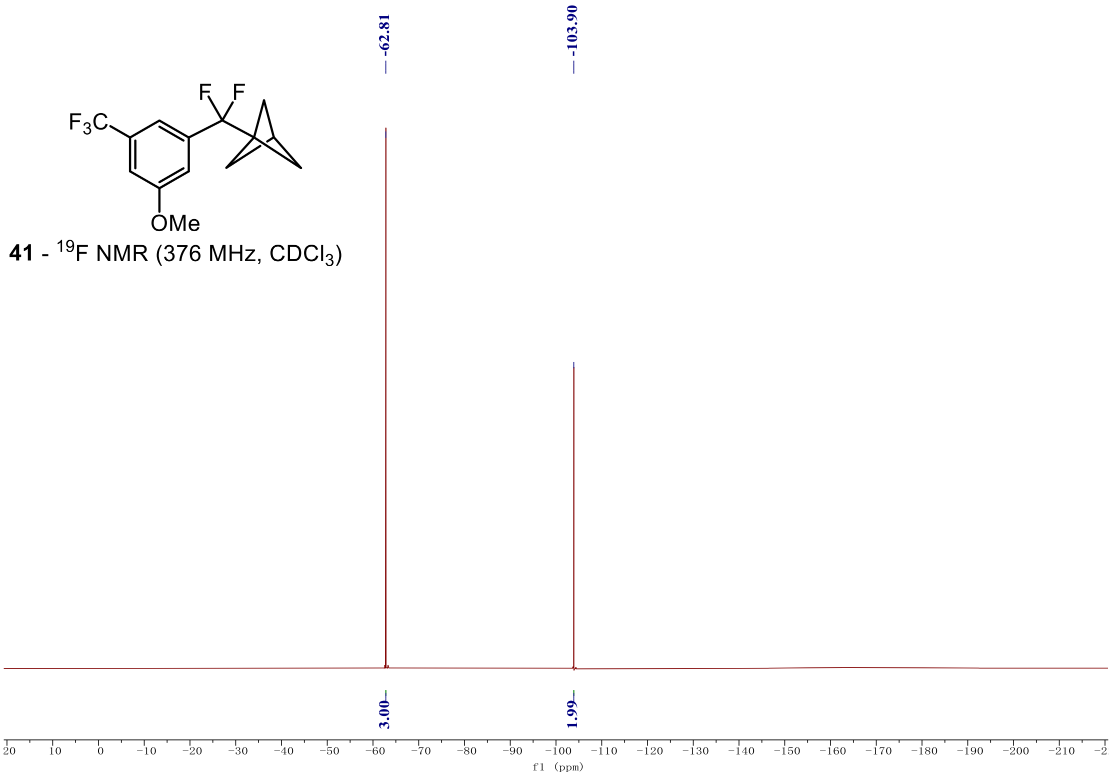

Supplementary Figure 143 ^{19}F NMR spectra of compound **41**

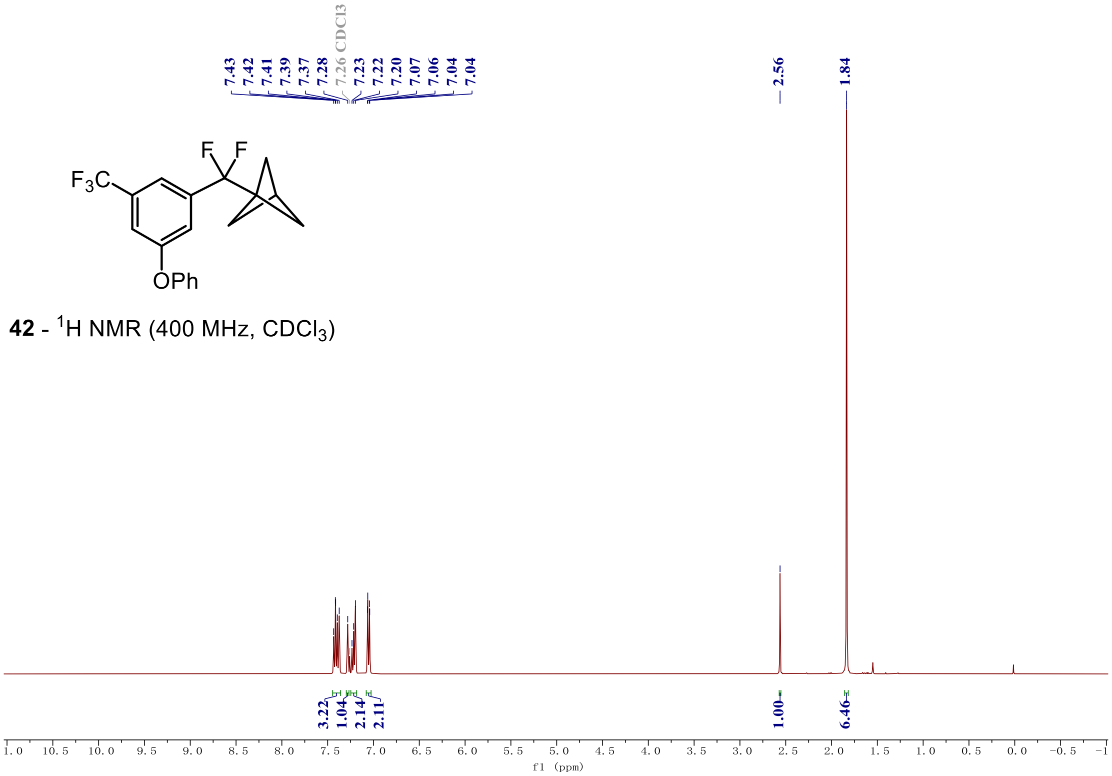

Supplementary Figure 144 ^{1}H NMR spectra of compound **42**

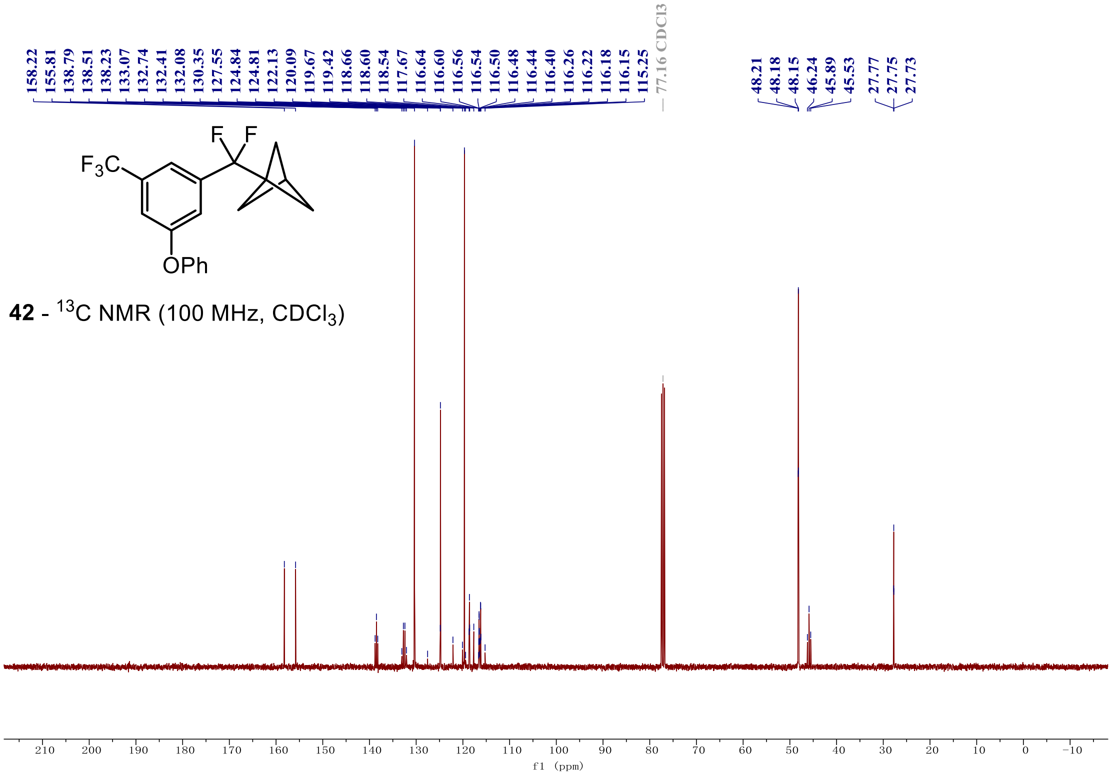

Supplementary Figure 145 ^{13}C NMR spectra of compound **42**

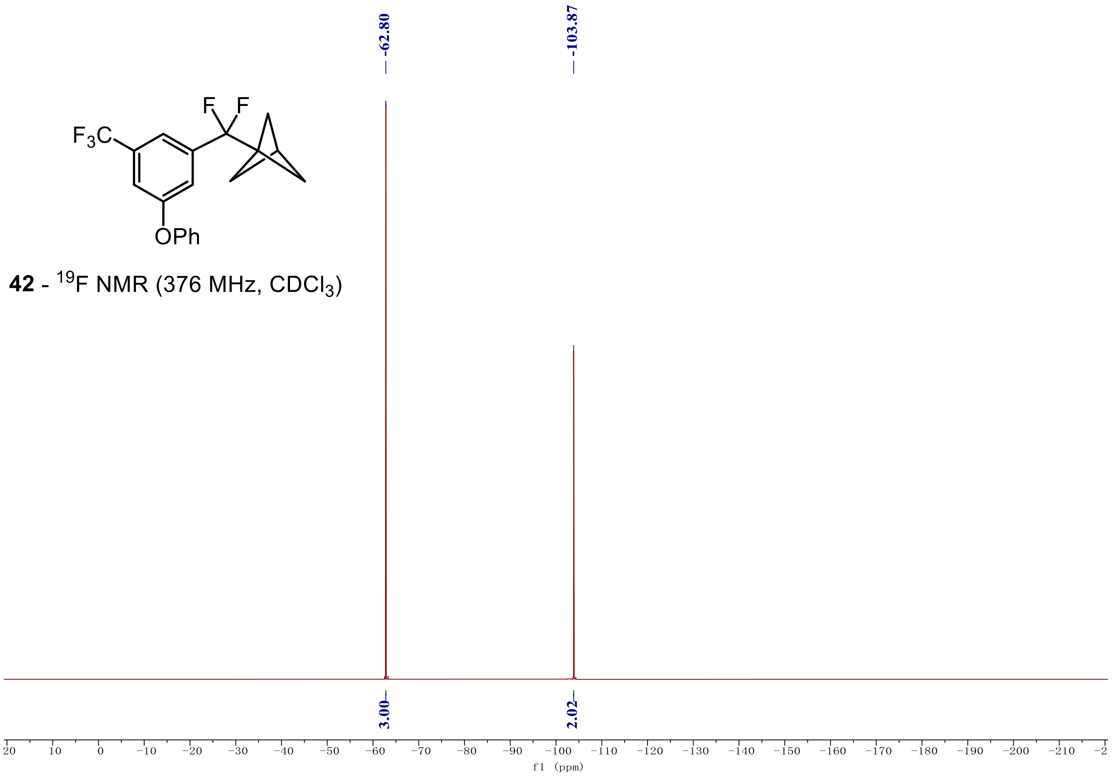

Supplementary Figure 146 ^{19}F NMR spectra of compound **42**

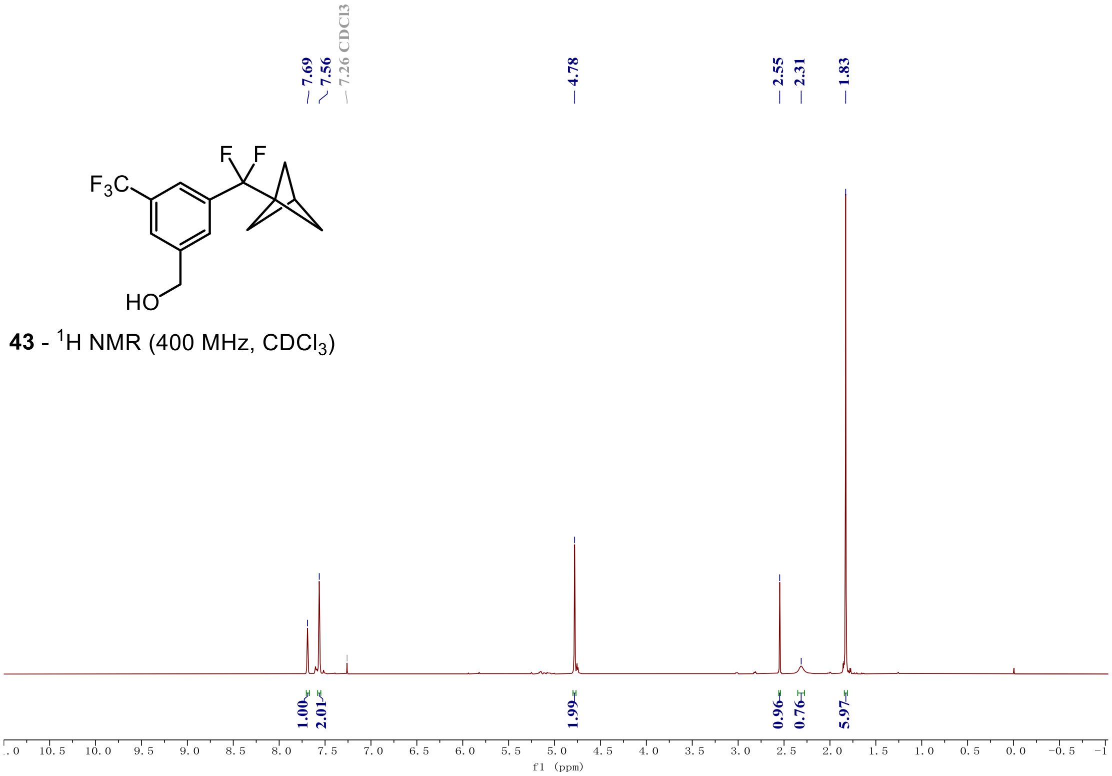

Supplementary Figure 147 ^{1}H NMR spectra of compound **43**

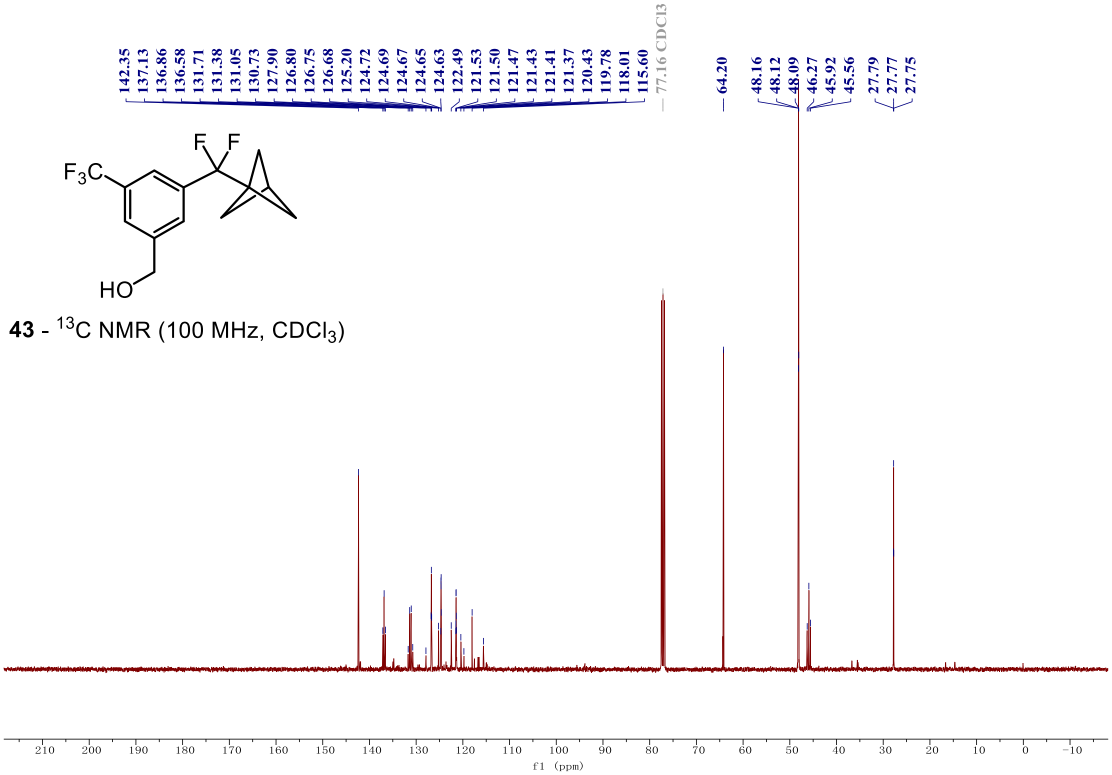

Supplementary Figure 148 ^{13}C NMR spectra of compound **43**

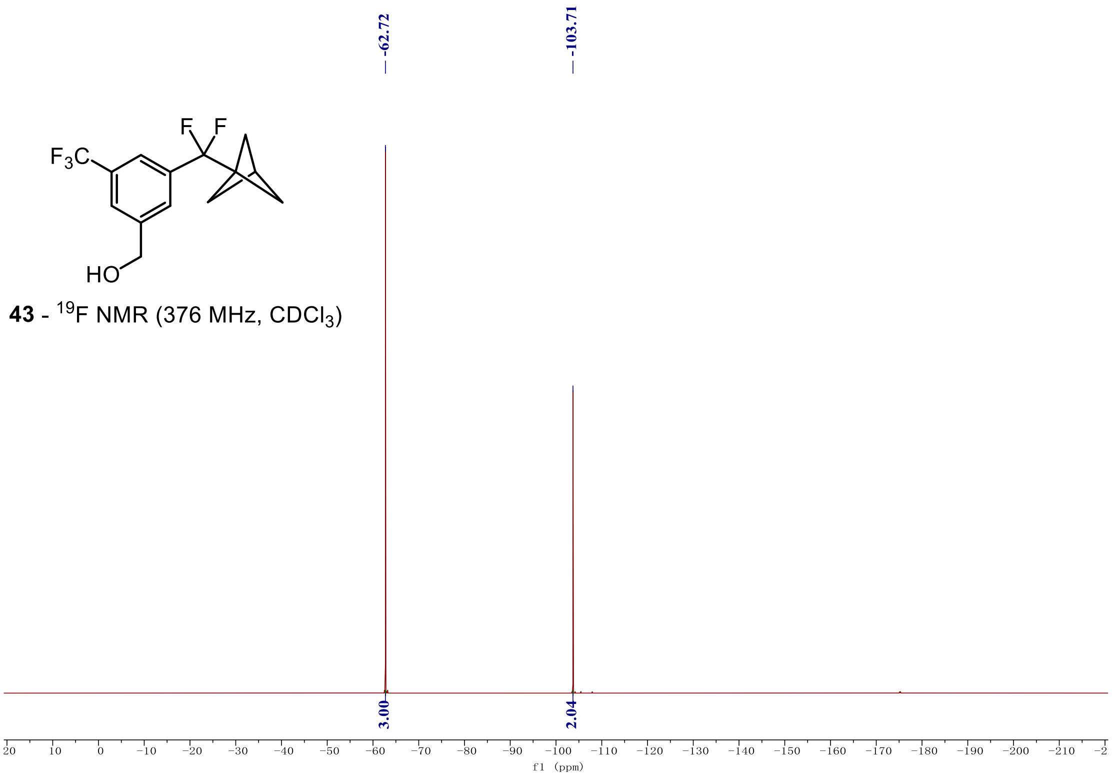

Supplementary Figure 149 ^{19}F NMR spectra of compound **43**

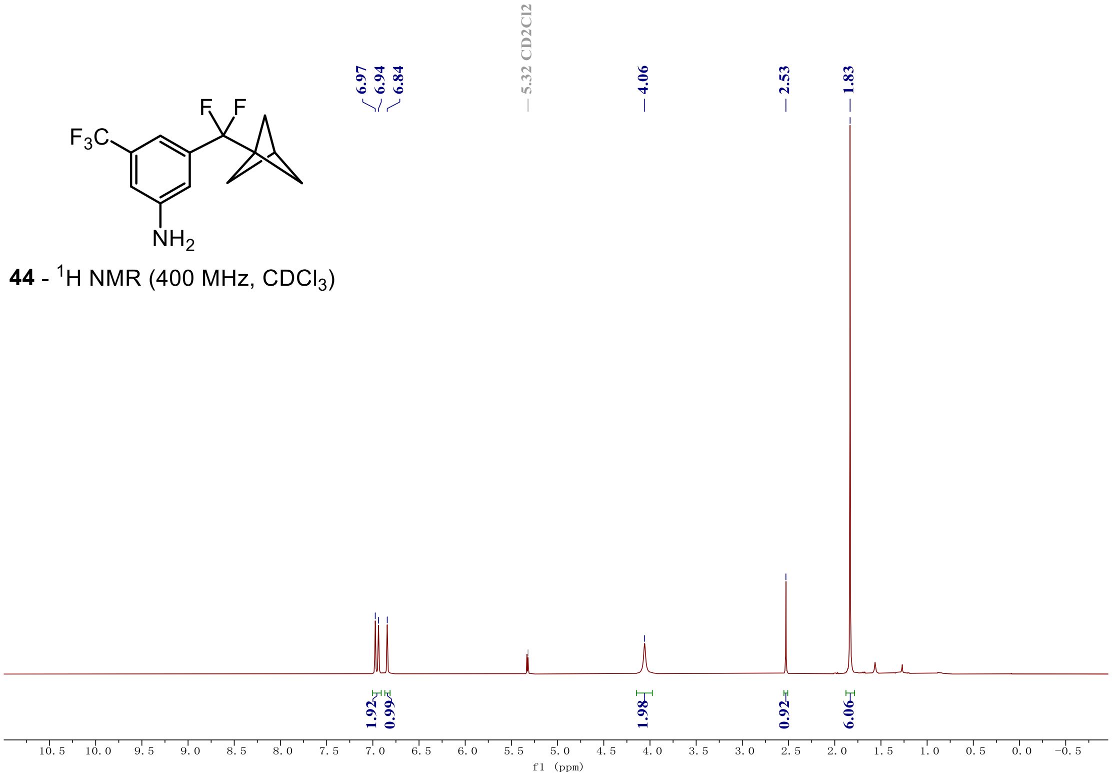

Supplementary Figure 150 ^{1}H NMR spectra of compound **44**

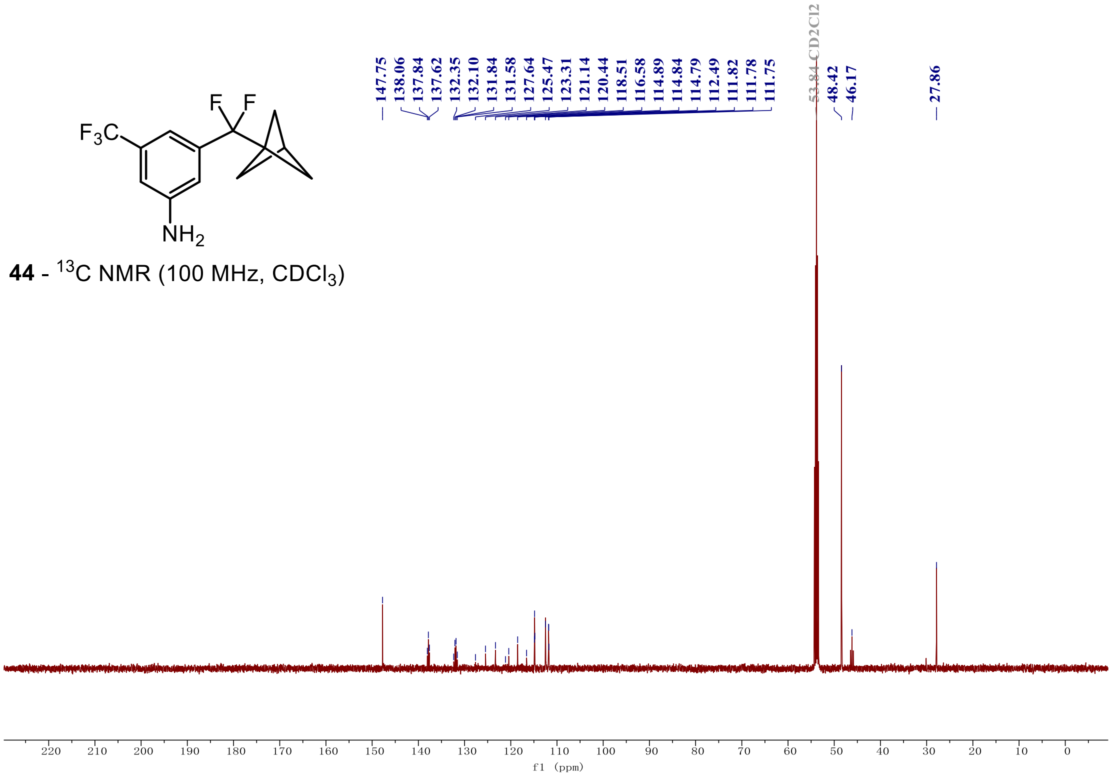

Supplementary Figure 151 ^{13}C NMR spectra of compound **44**

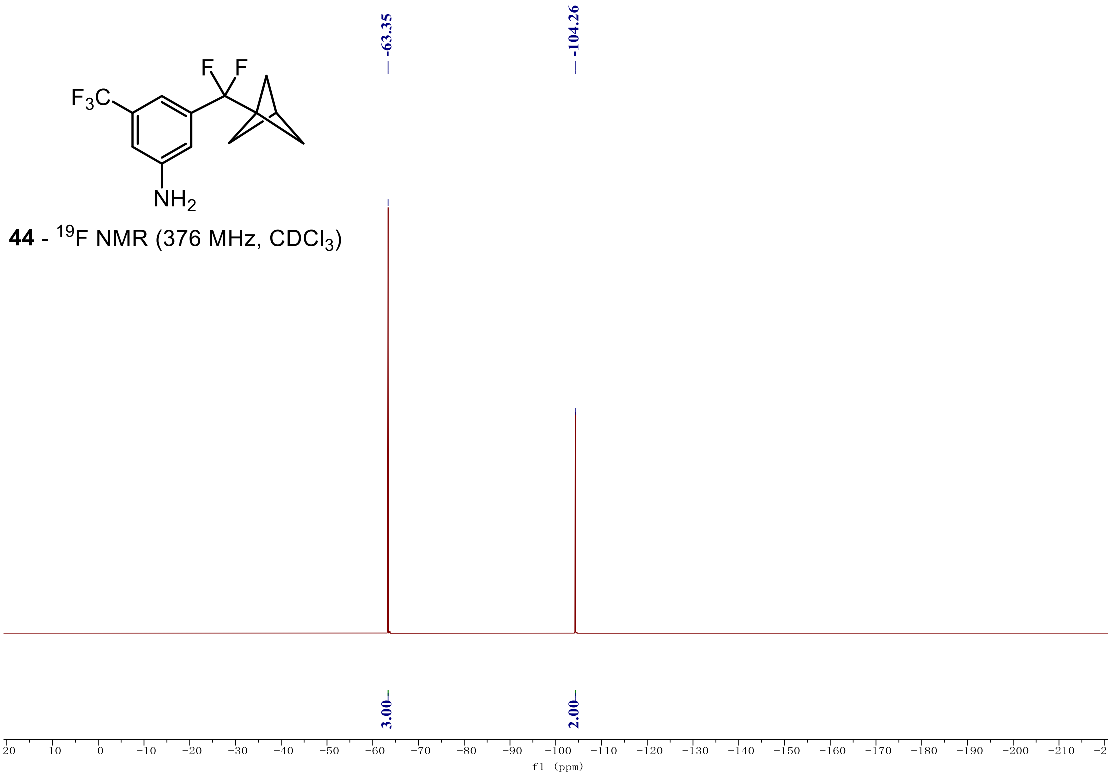

Supplementary Figure 152 ^{19}F NMR spectra of compound **44**

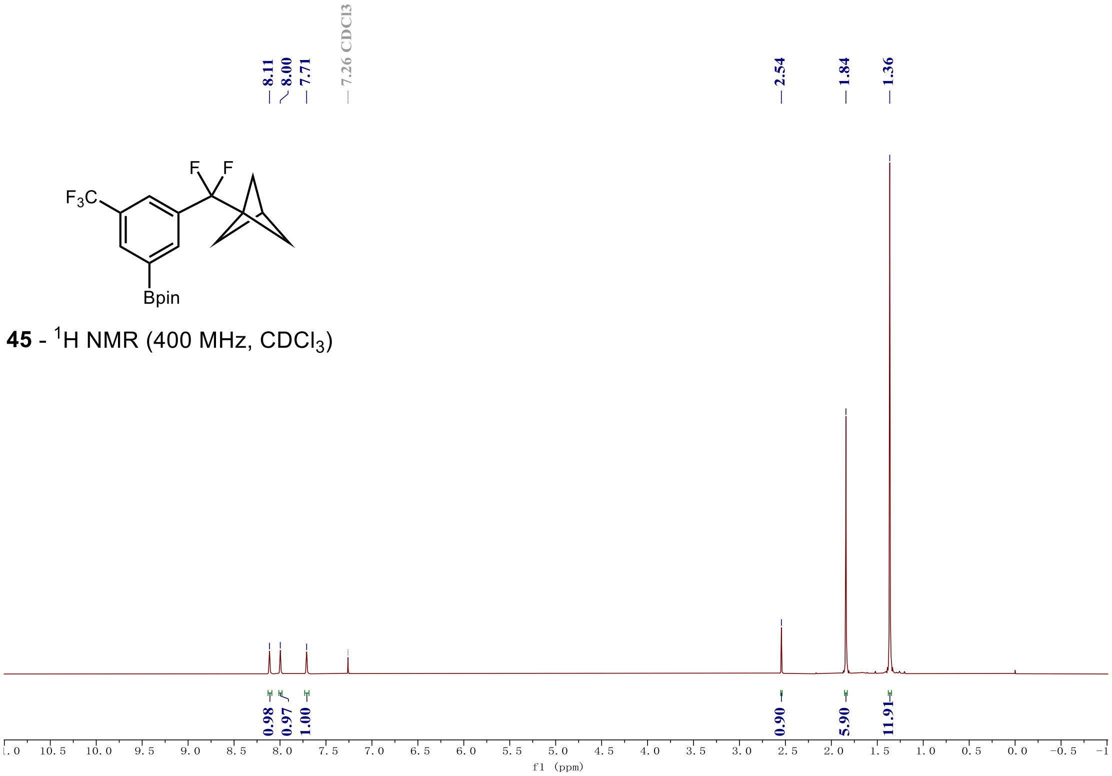

Supplementary Figure 153 ^{1}H NMR spectra of compound **45**

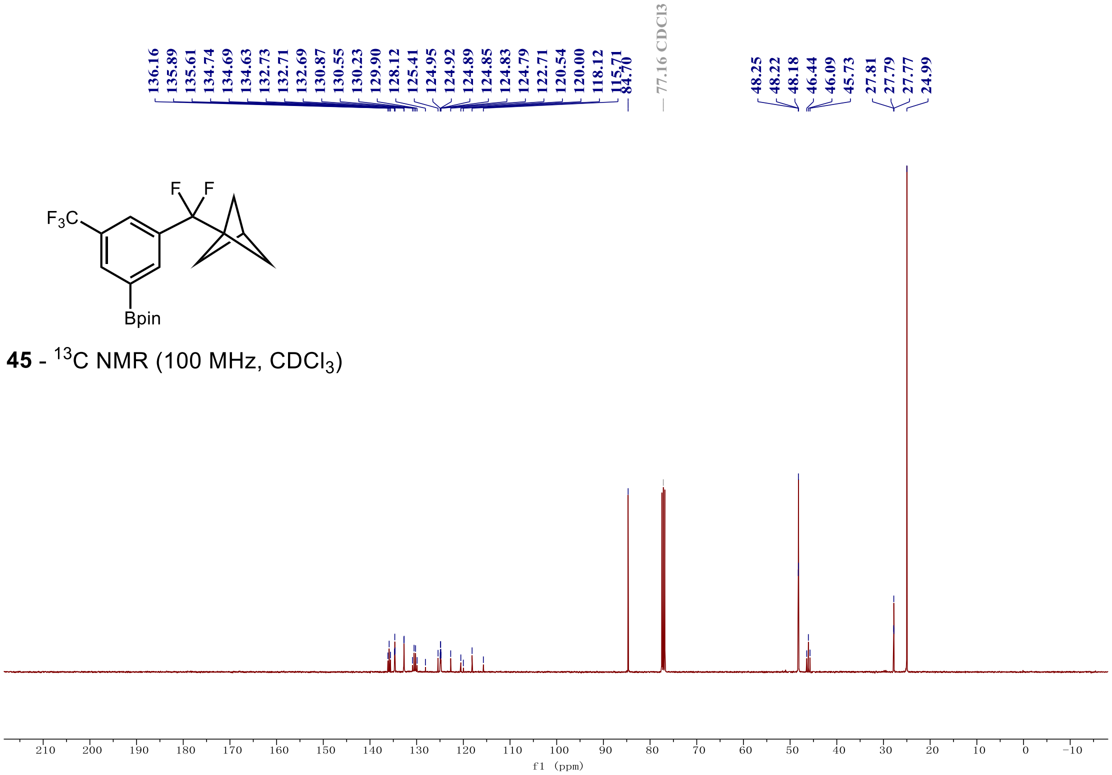

Supplementary Figure 154 ^{13}C NMR spectra of compound **45**

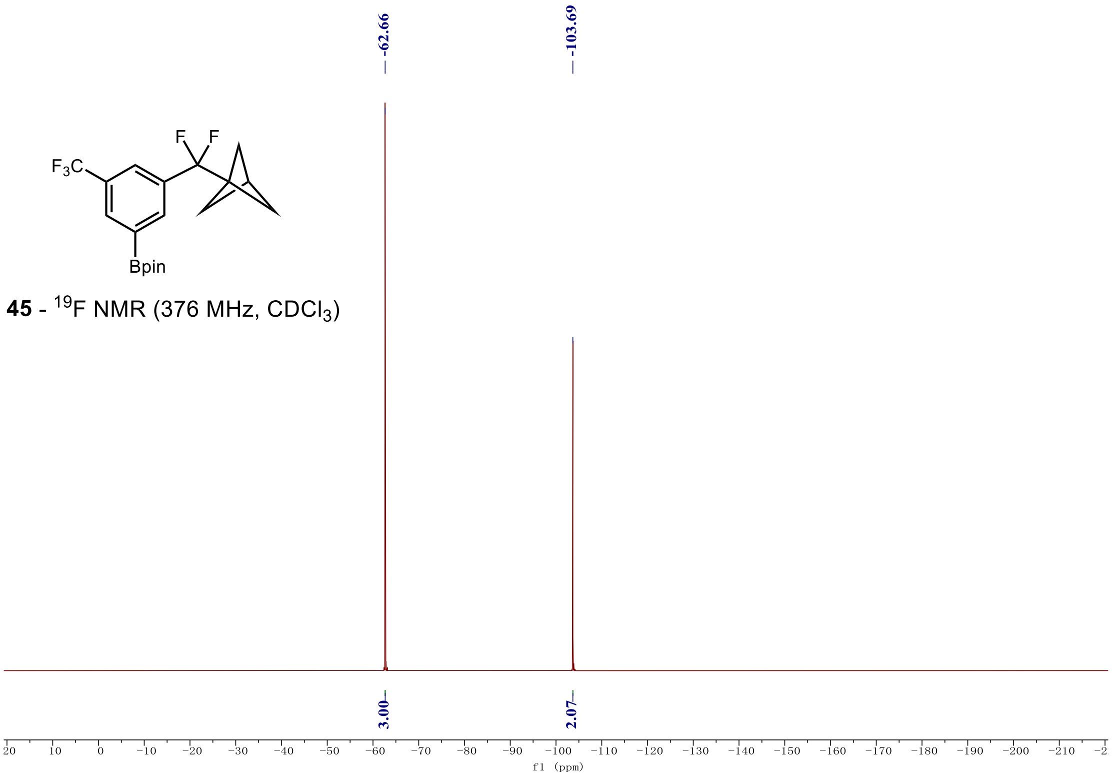

Supplementary Figure 155 ^{19}F NMR spectra of compound **45**

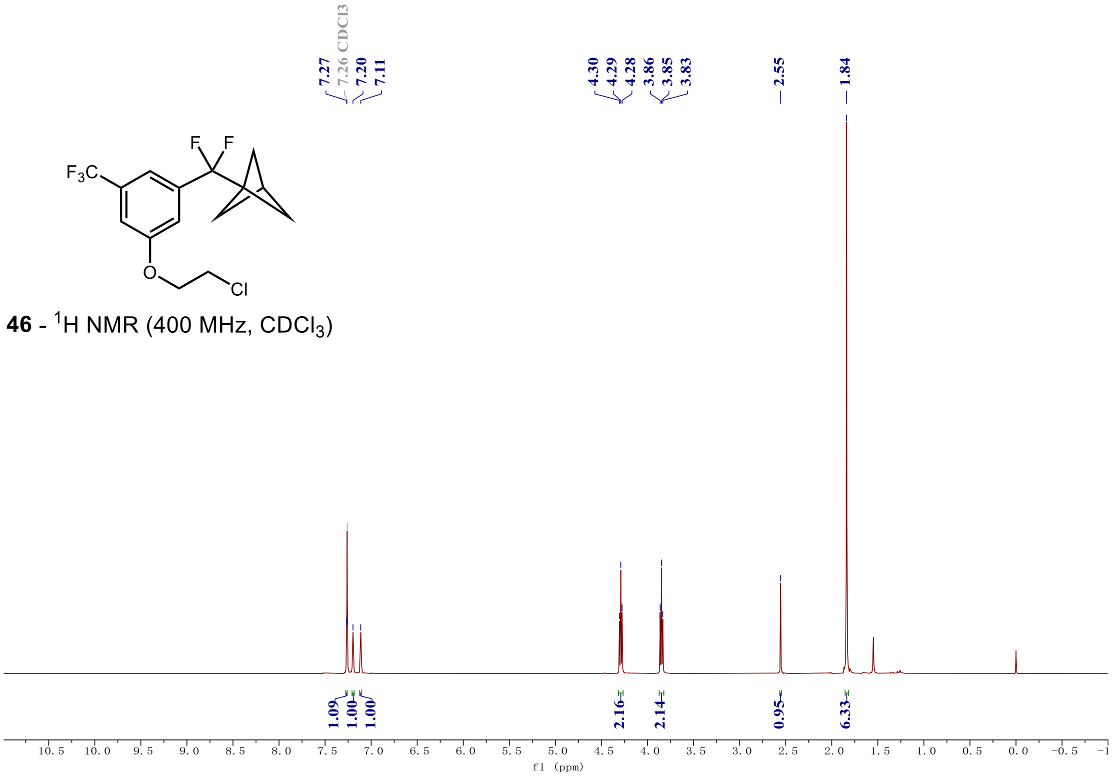

Supplementary Figure 156 ^{1}H NMR spectra of compound **46**

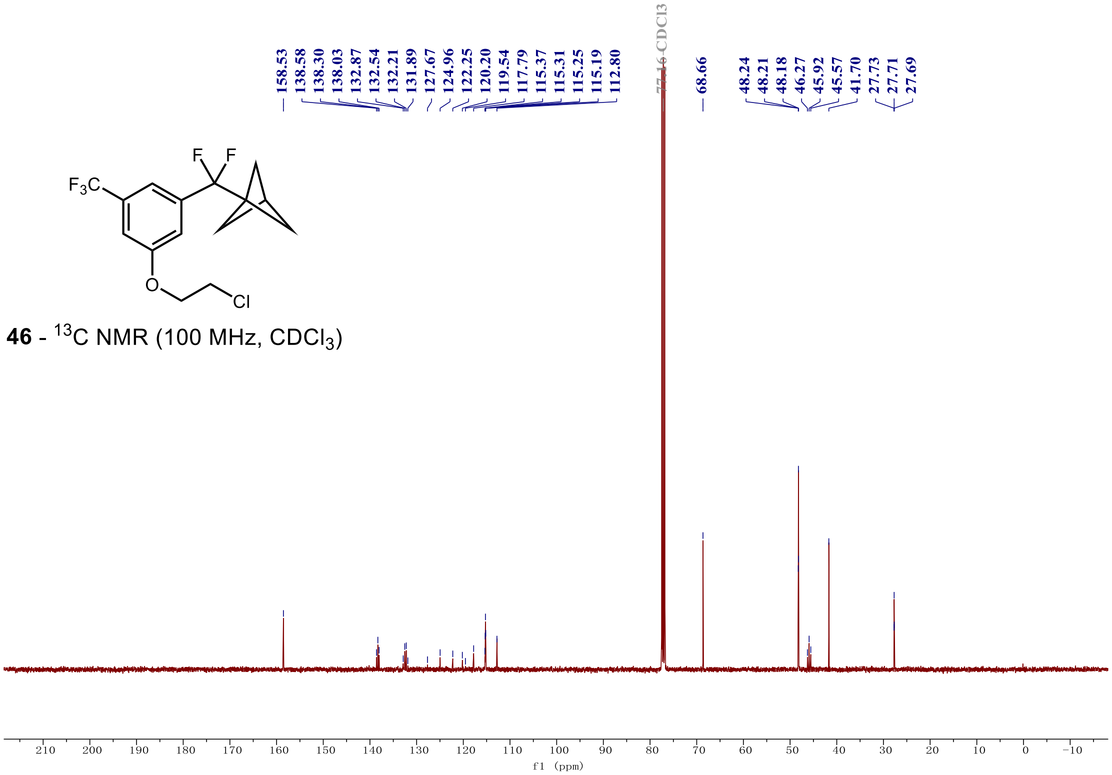

Supplementary Figure 157 ^{13}C NMR spectra of compound **46**

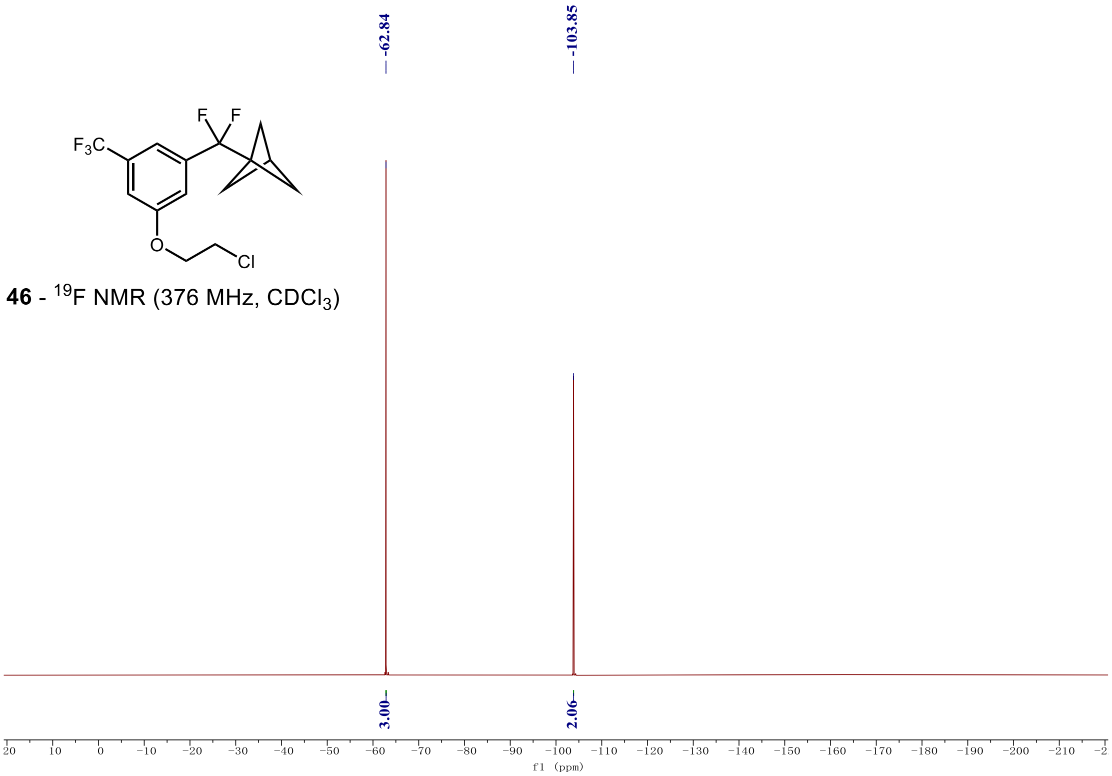

Supplementary Figure 158 ^{19}F NMR spectra of compound **46**

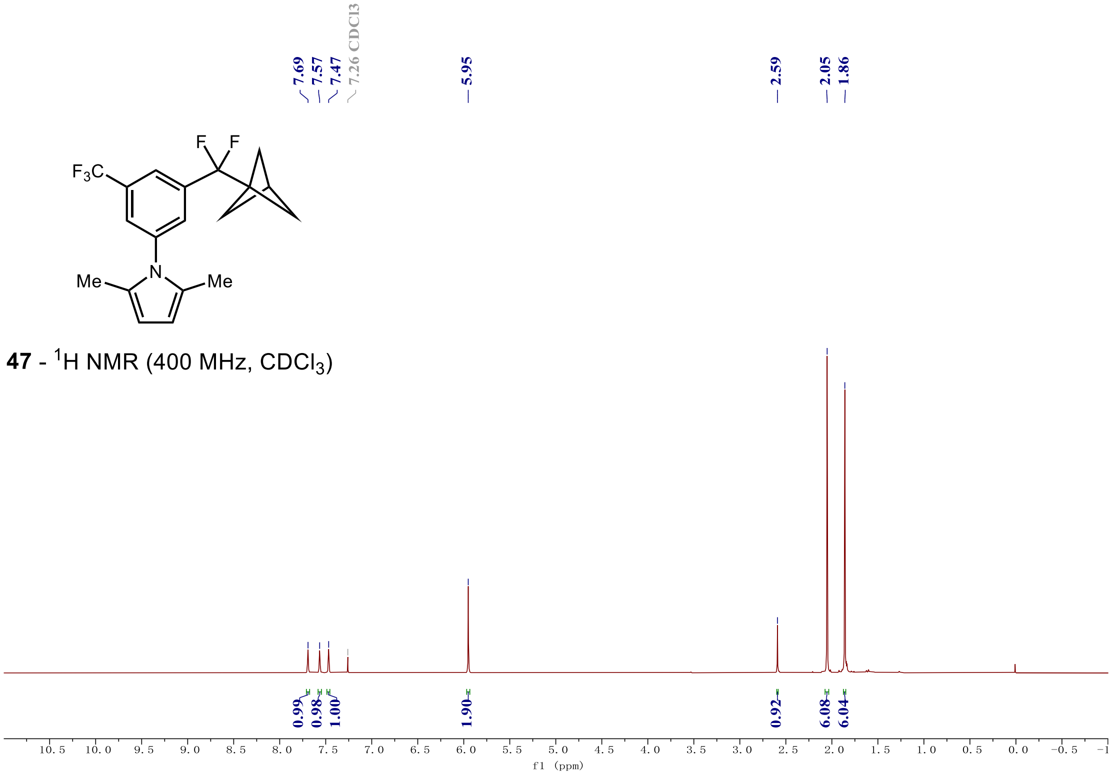

Supplementary Figure 159 ^{1}H NMR spectra of compound **47**

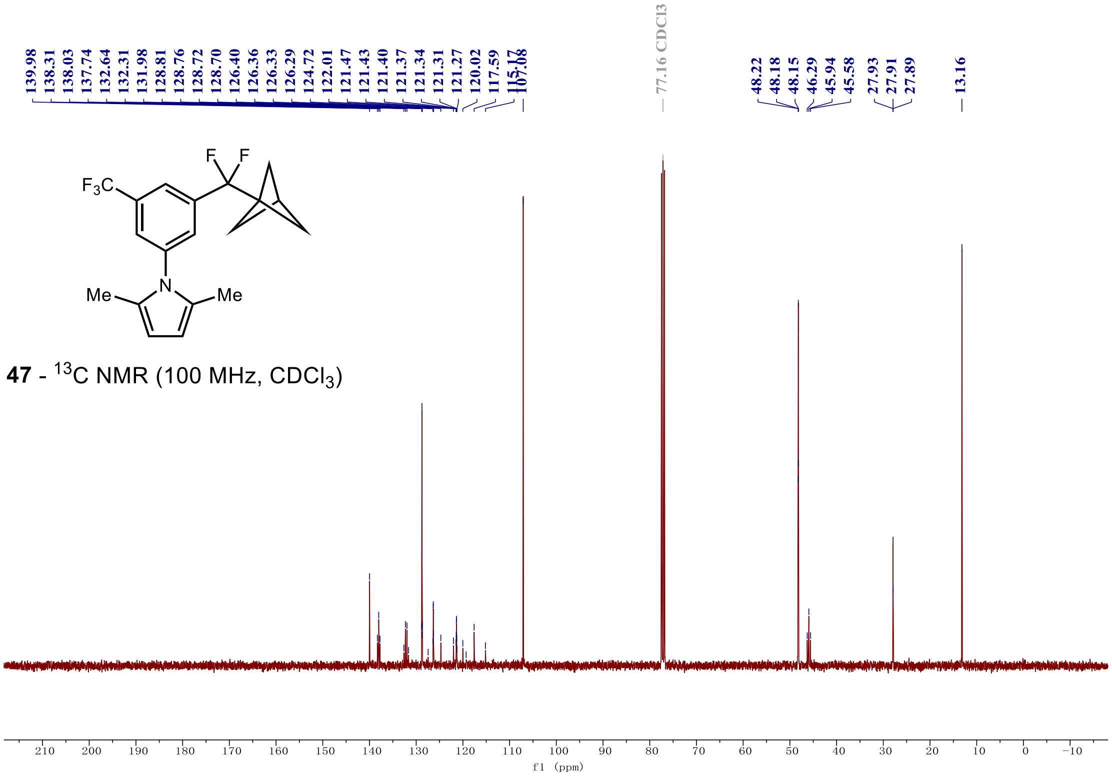

Supplementary Figure 160 ^{13}C NMR spectra of compound **47**

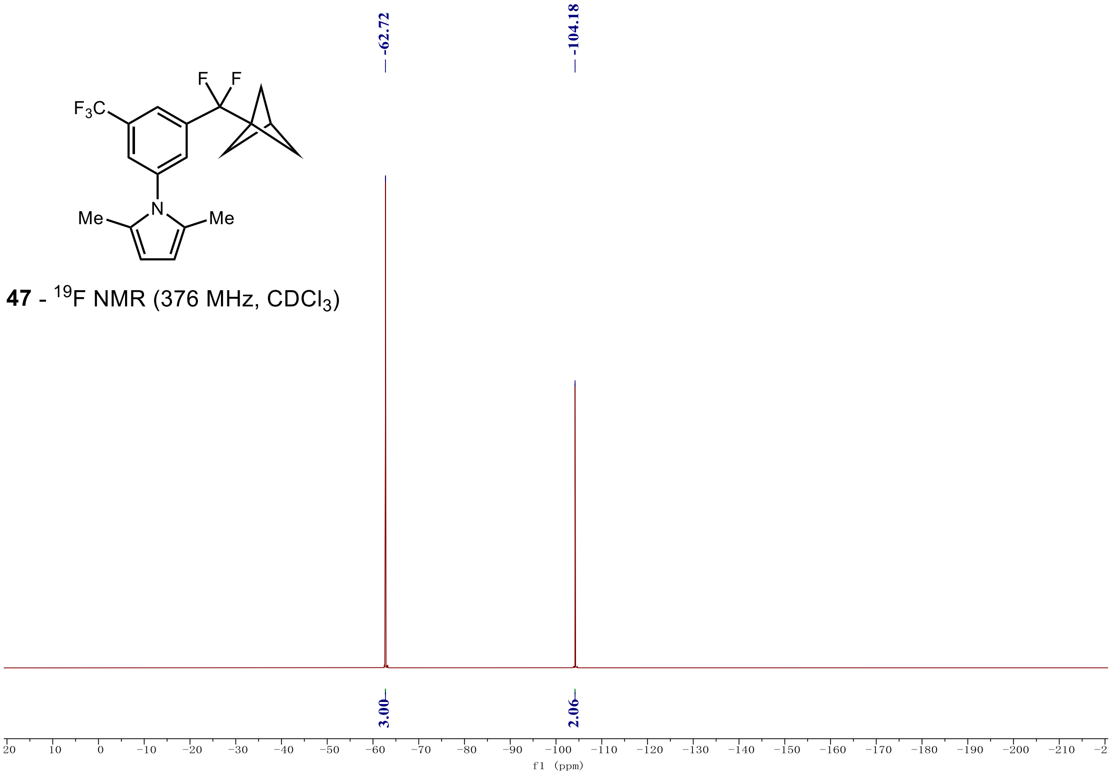

Supplementary Figure 161 ^{19}F NMR spectra of compound **47**

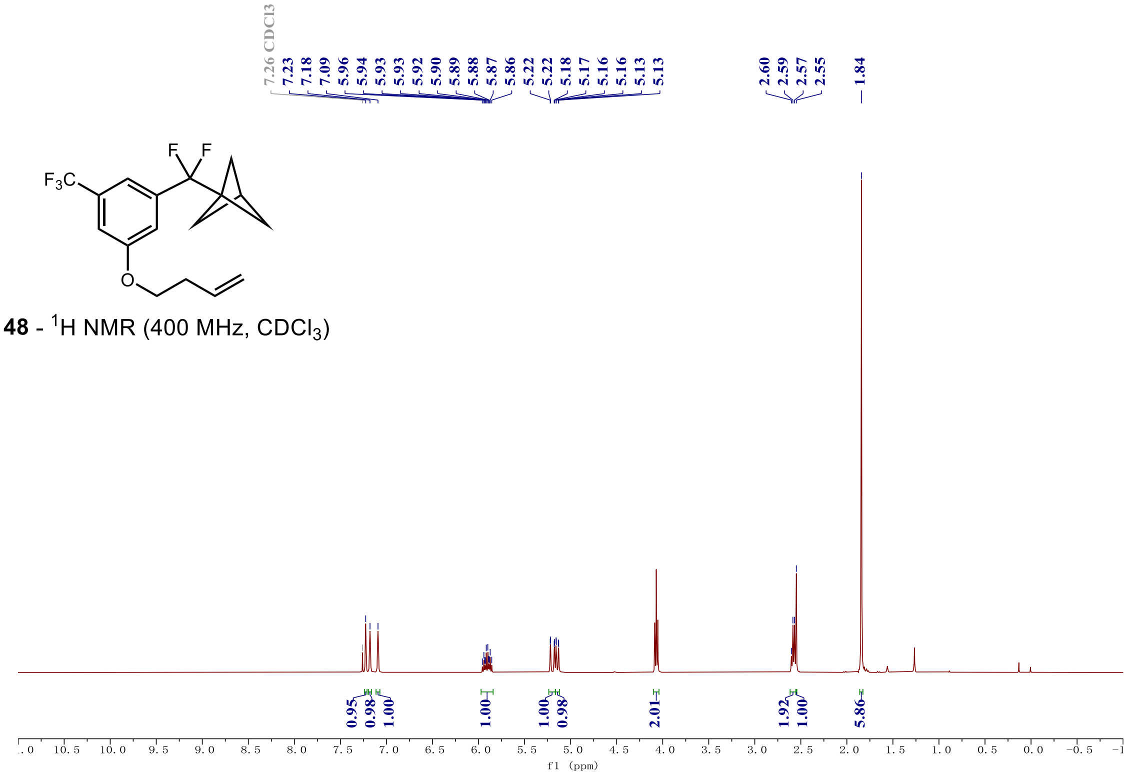

Supplementary Figure 162 ^{1}H NMR spectra of compound **48**

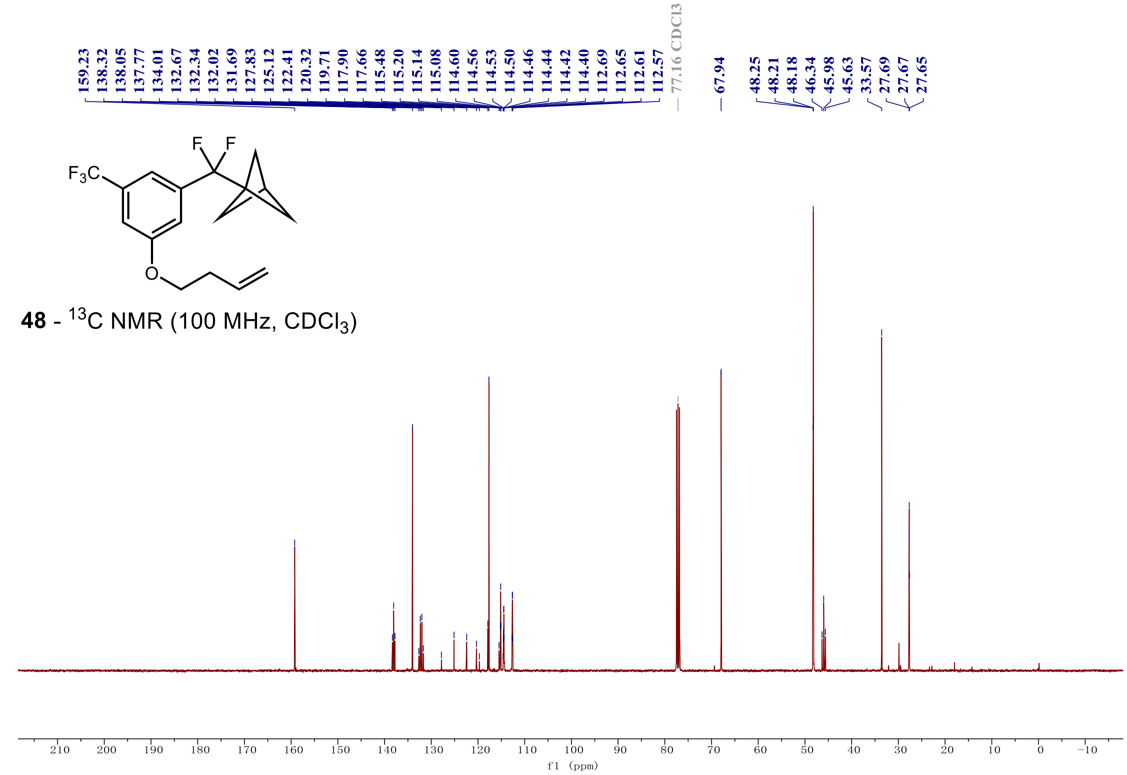

Supplementary Figure 163 ^{13}C NMR spectra of compound **48**

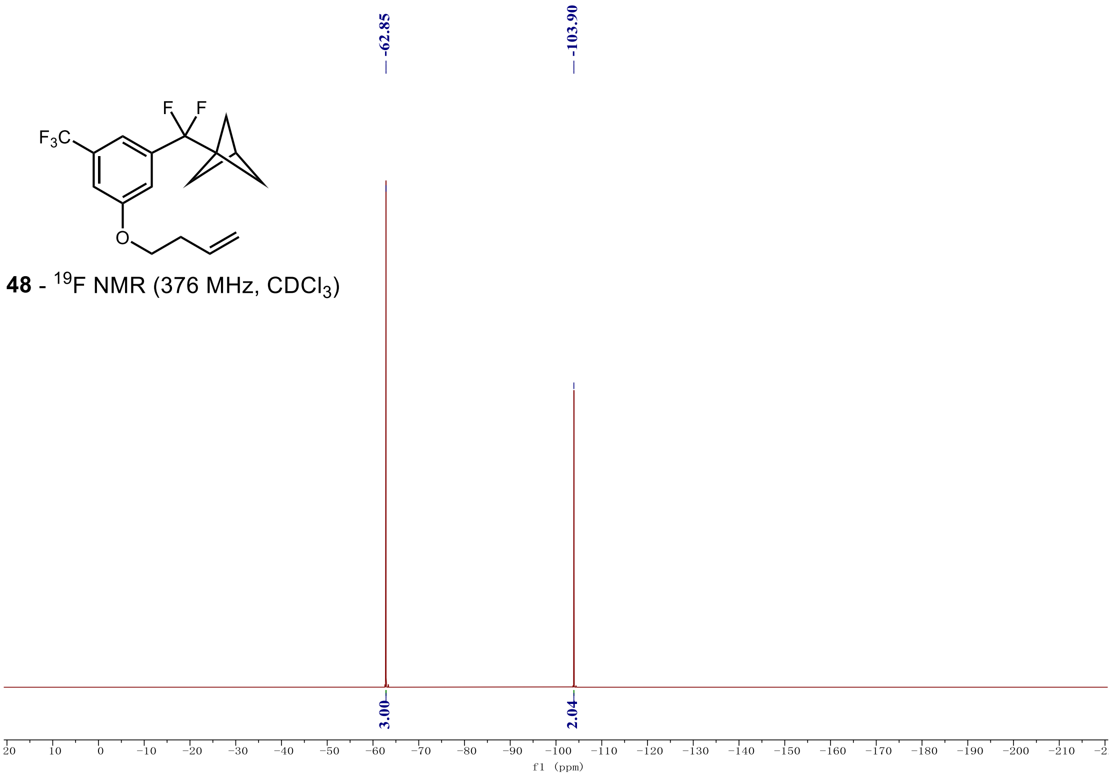

Supplementary Figure 164 ^{19}F NMR spectra of compound **48**

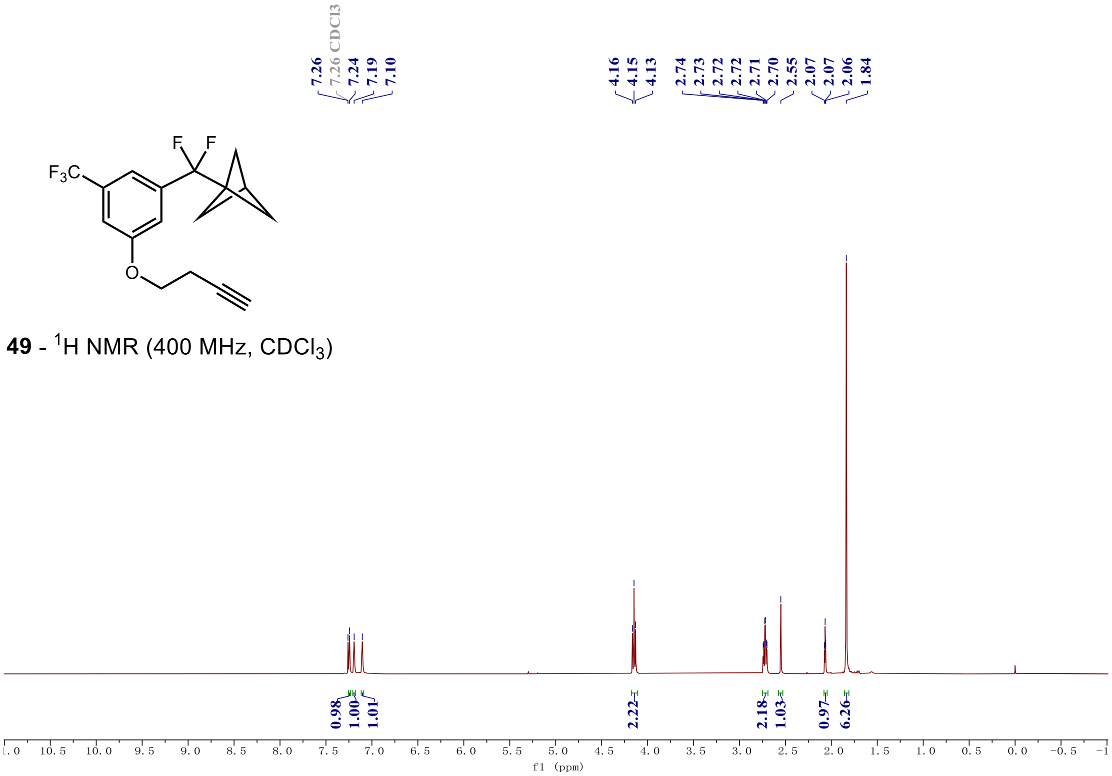

Supplementary Figure 165 ^{1}H NMR spectra of compound **49**

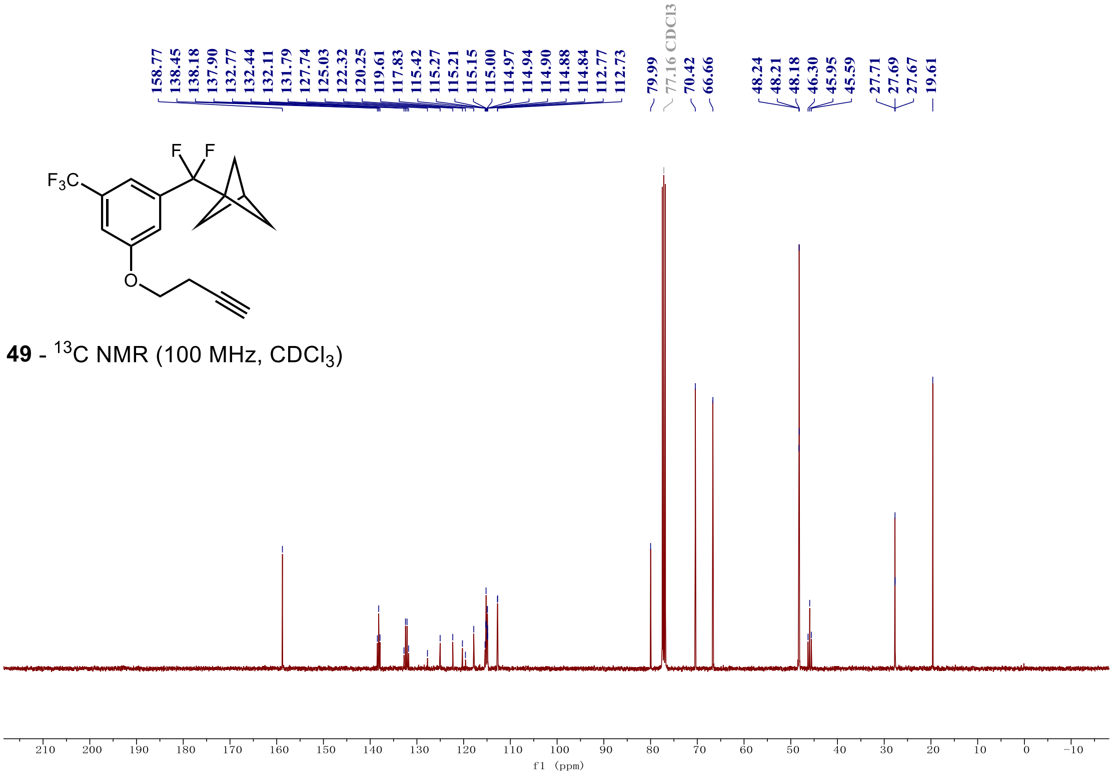

Supplementary Figure 166 ^{13}C NMR spectra of compound **49**

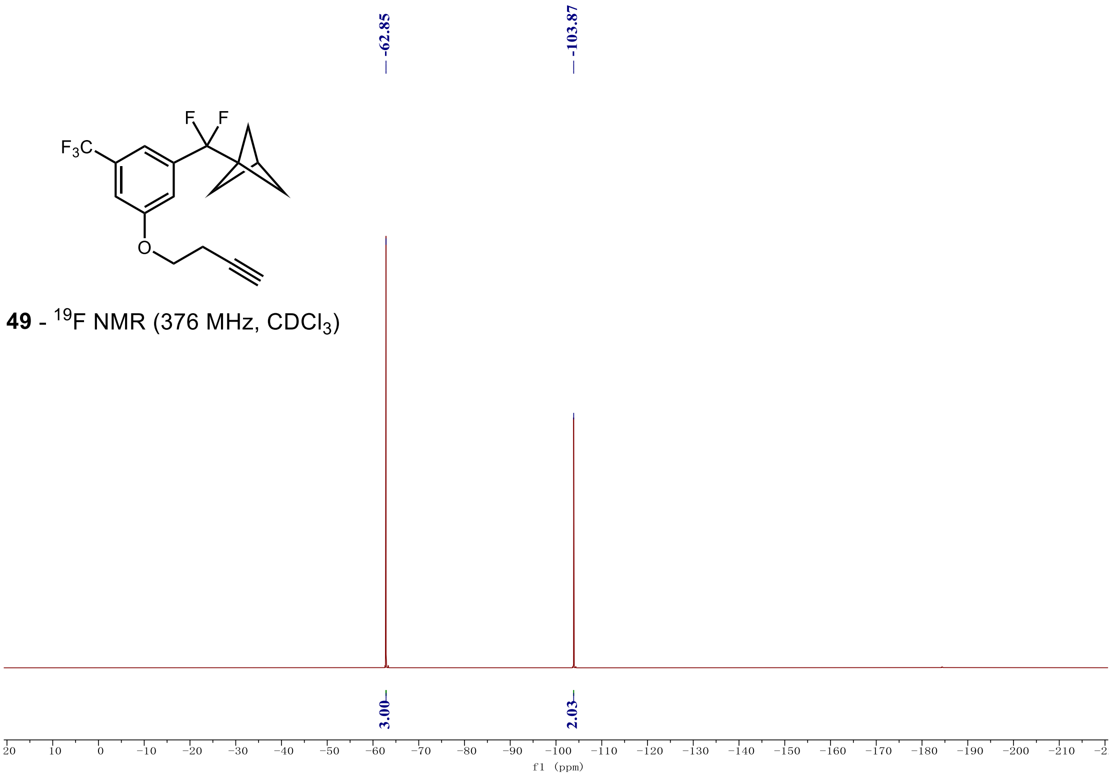

Supplementary Figure 167 ^{19}F NMR spectra of compound **49**

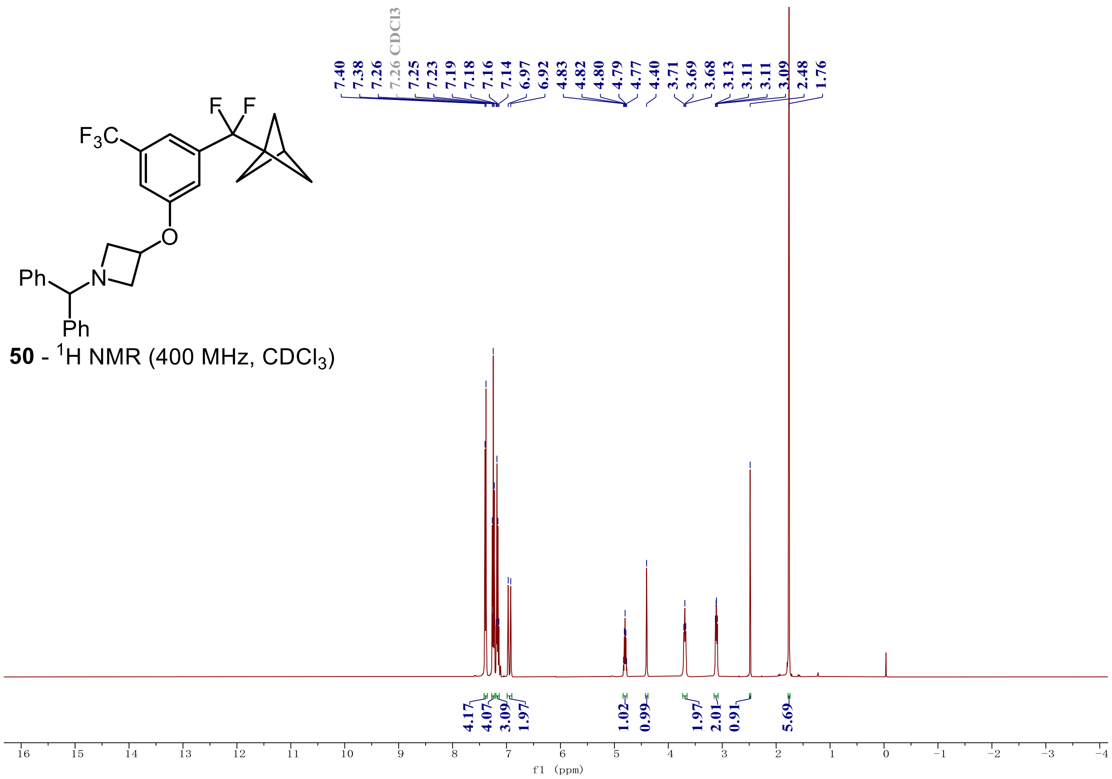

Supplementary Figure 168 ^{1}H NMR spectra of compound **50**

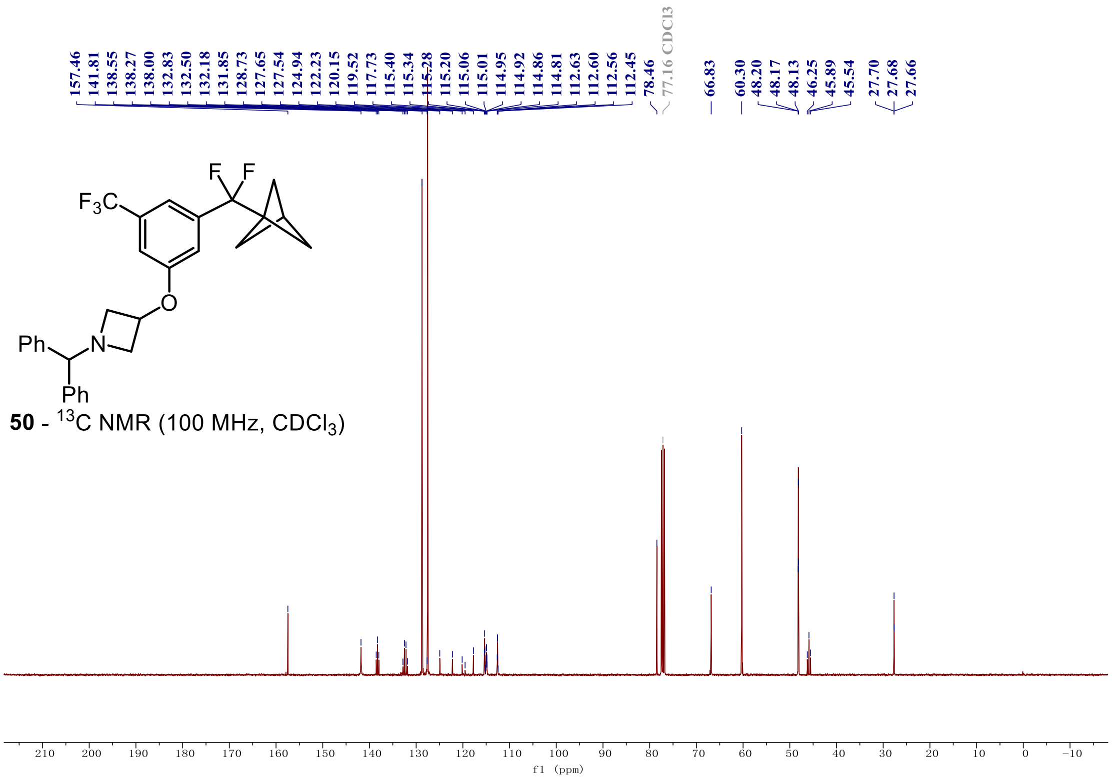

Supplementary Figure 169 ^{13}C NMR spectra of compound **50**

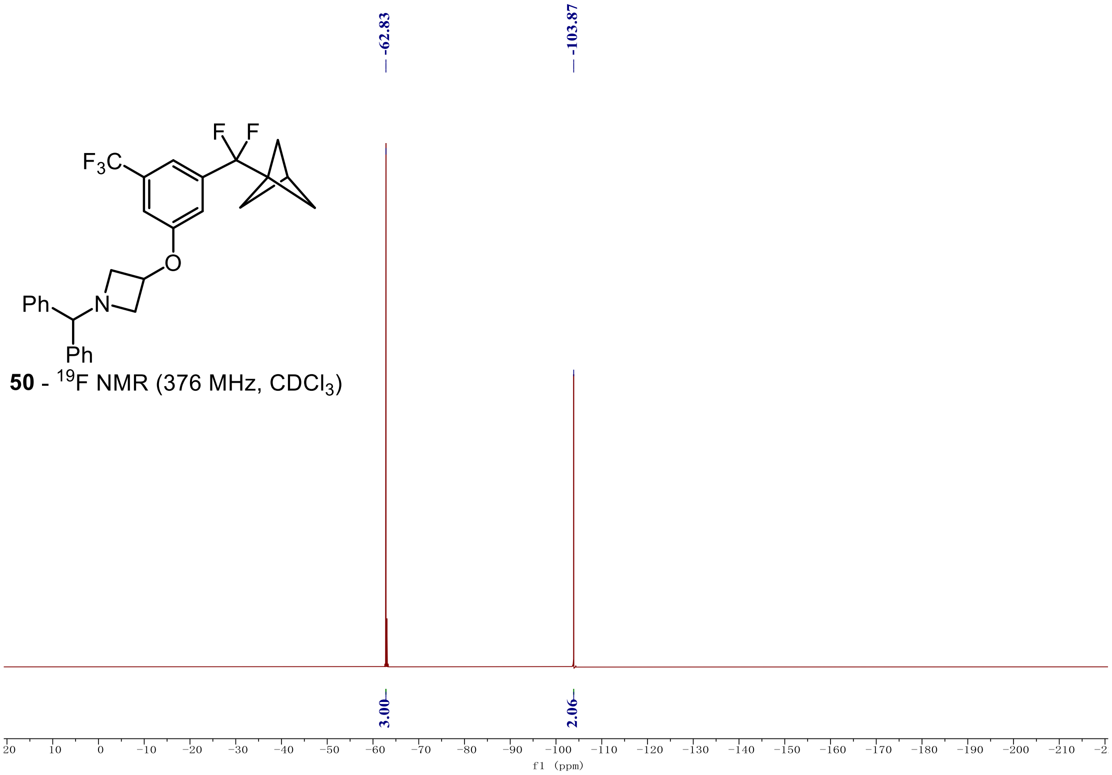

Supplementary Figure 170 ^{19}F NMR spectra of compound **50**

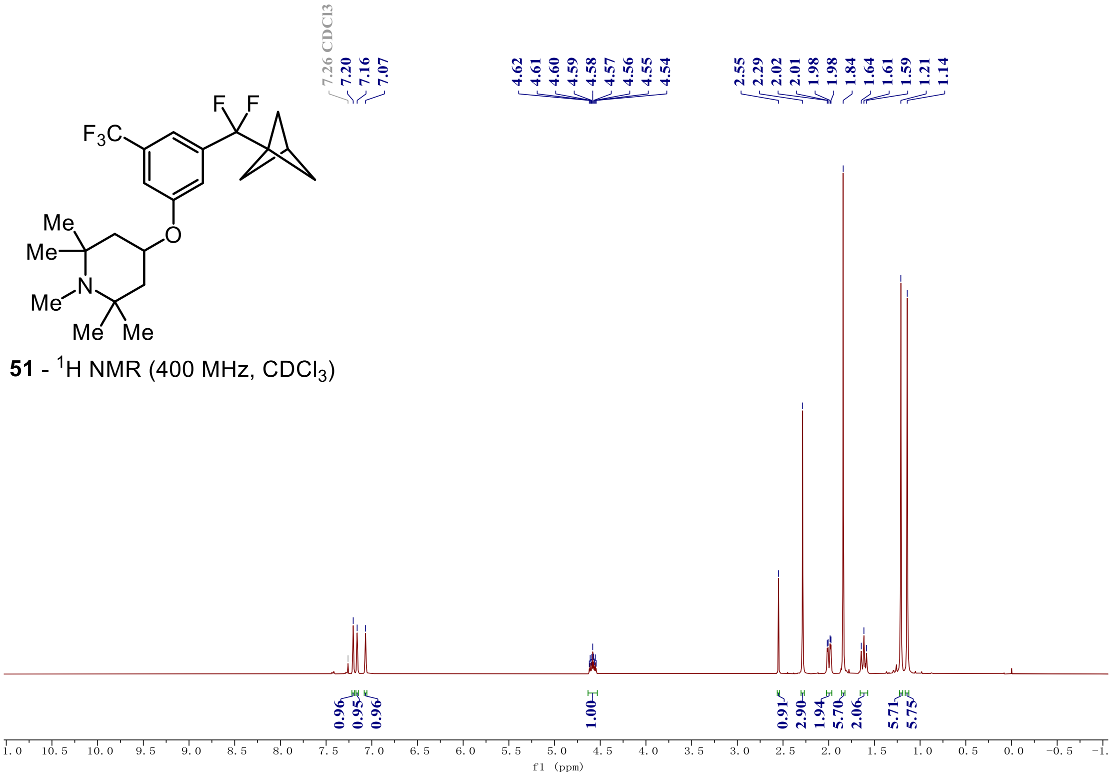

Supplementary Figure 171 ^{1}H NMR spectra of compound **51**

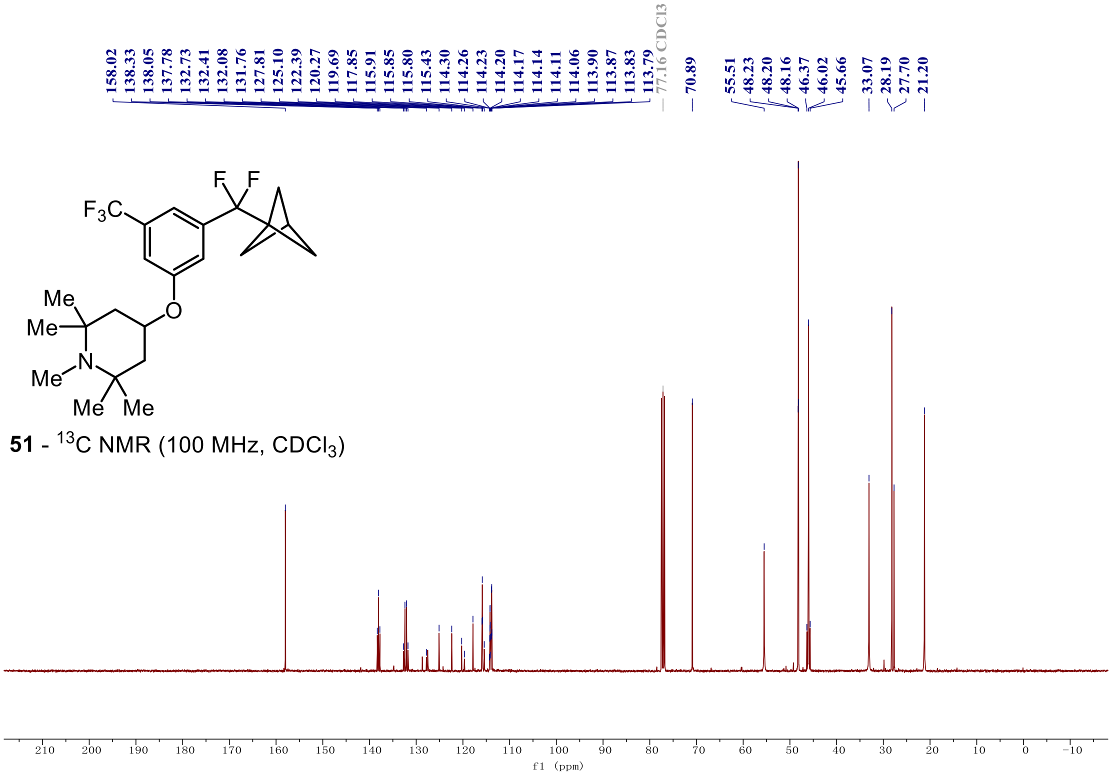

Supplementary Figure 172 ^{13}C NMR spectra of compound **51**

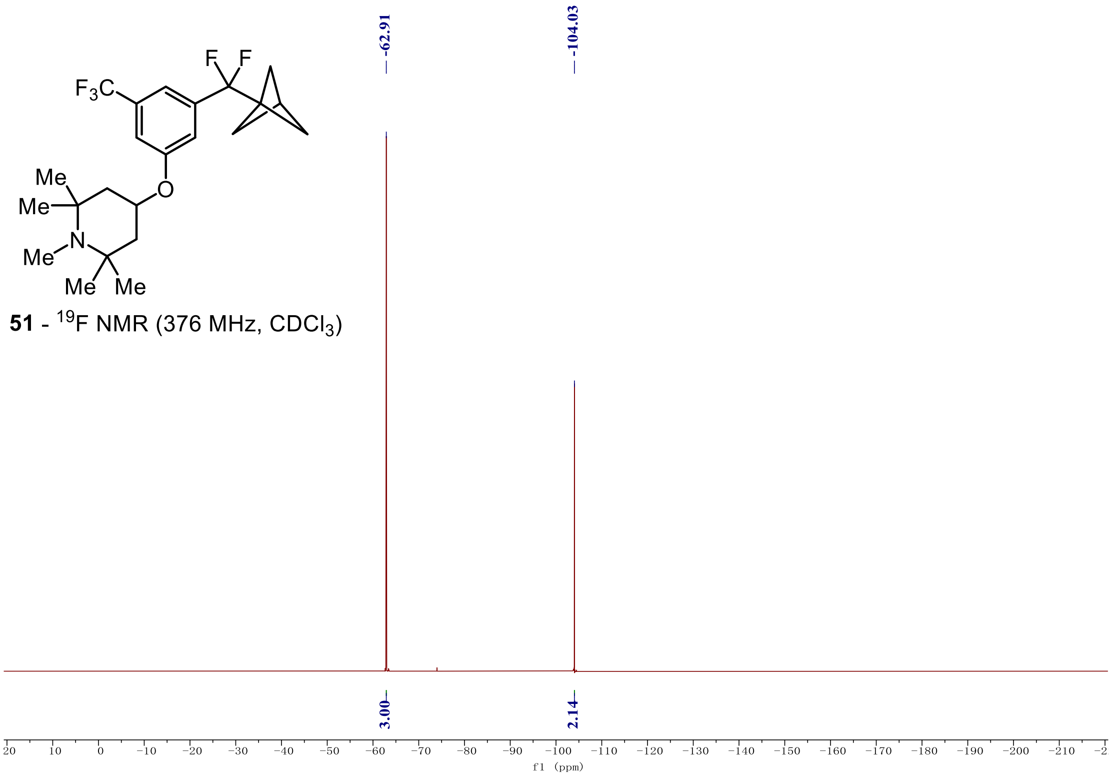

Supplementary Figure 173 ^{19}F NMR spectra of compound **51**

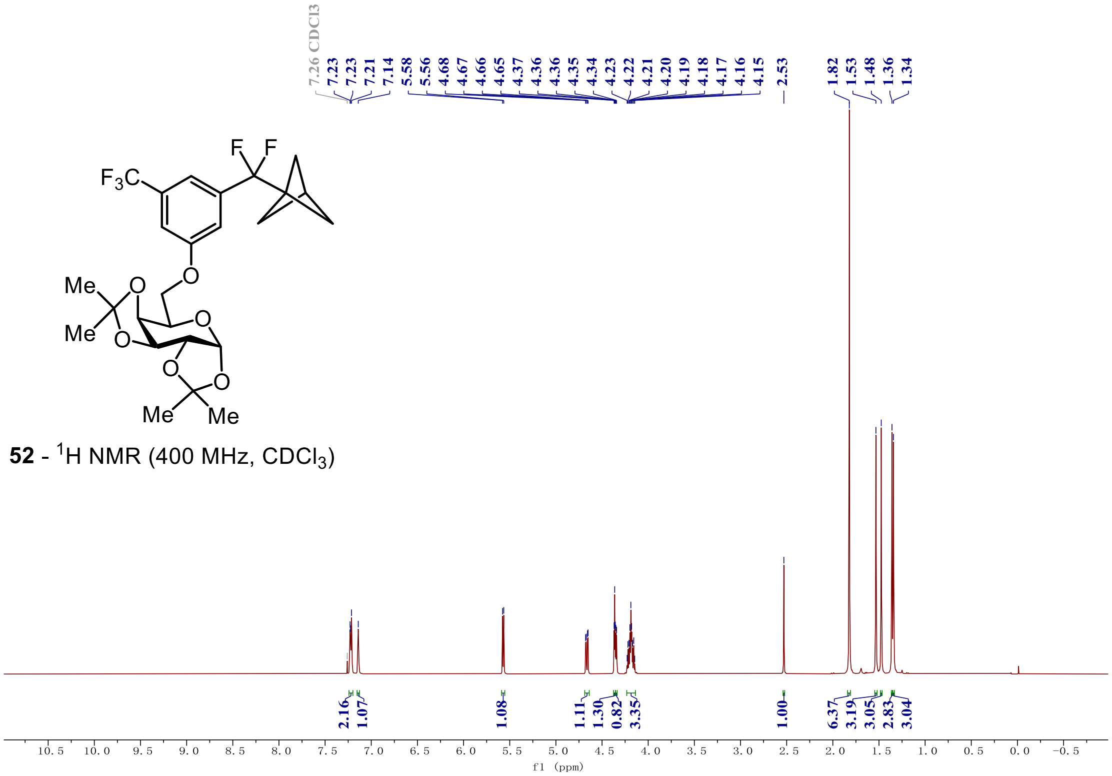

Supplementary Figure 174 ^{1}H NMR spectra of compound **52**

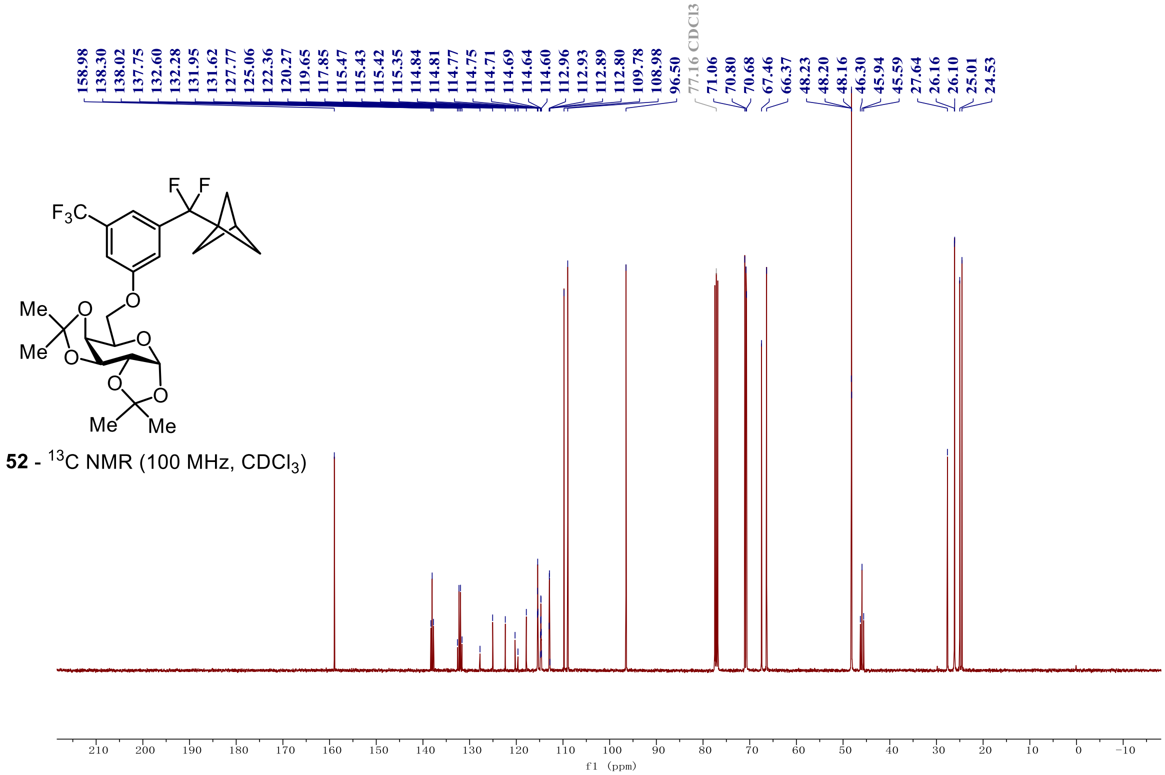

Supplementary Figure 175 ^{13}C NMR spectra of compound **52**

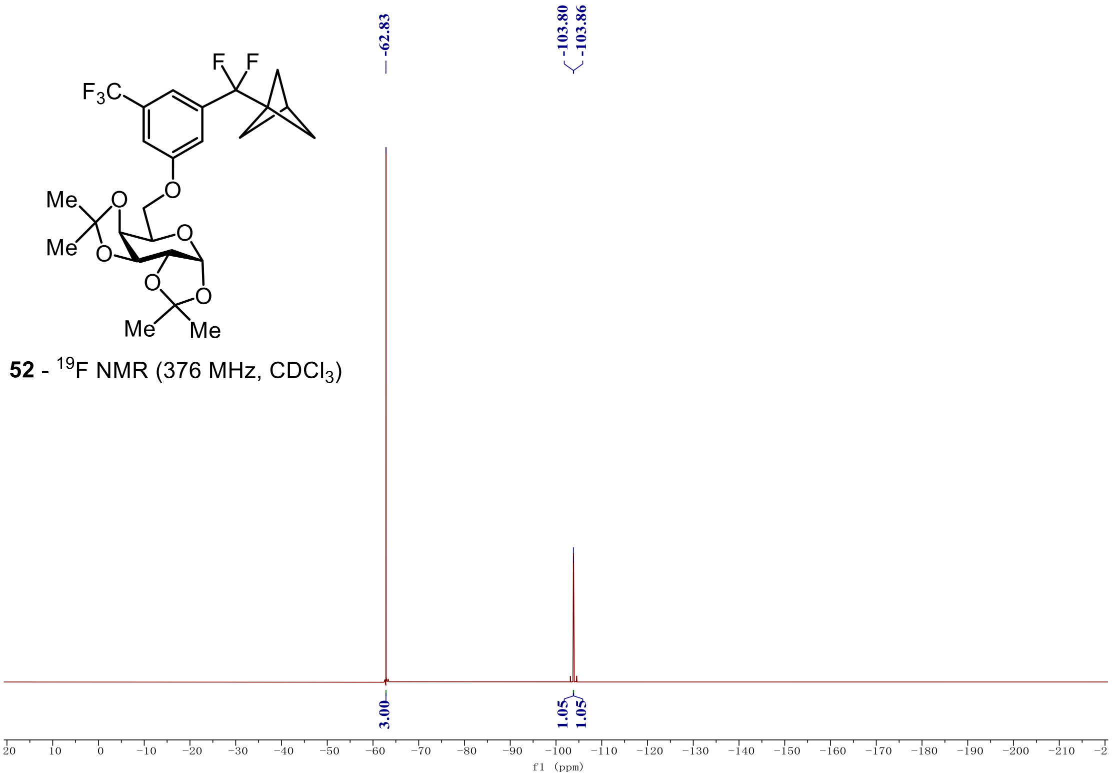

52 - ^{19}F NMR (376 MHz, $CDCl_3$)

Supplementary Figure 176 ^{19}F NMR spectra of compound **52**

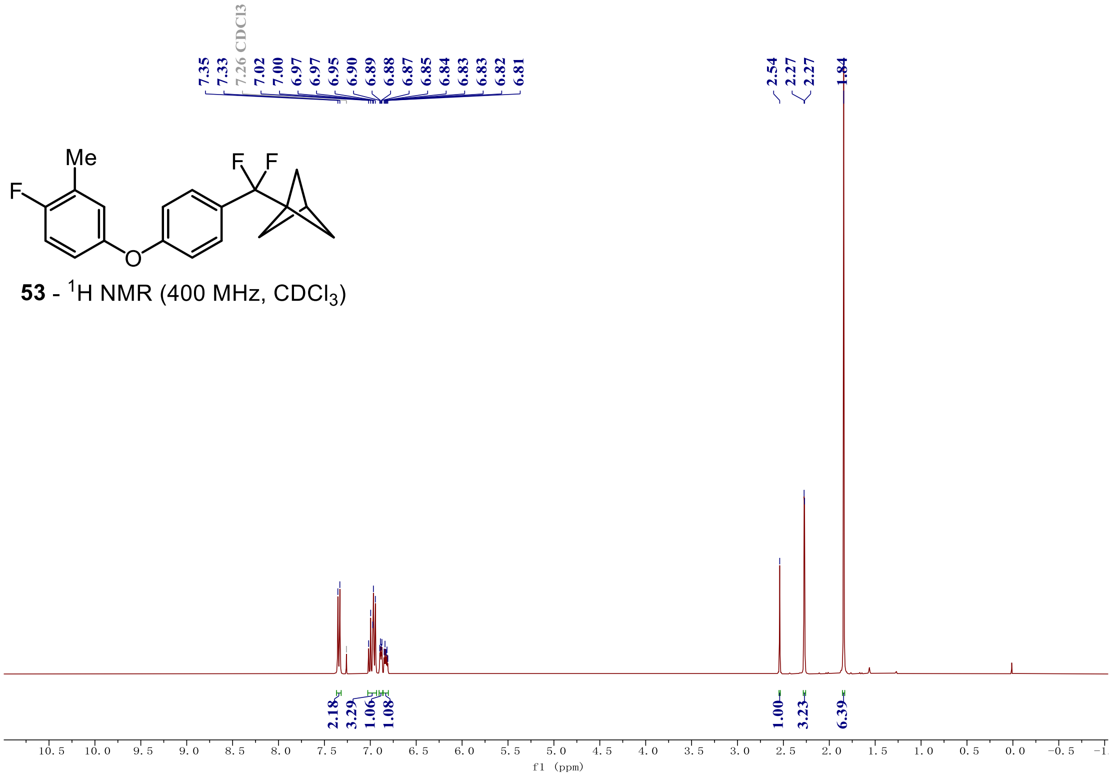

Supplementary Figure 177 ^{1}H NMR spectra of compound **53**

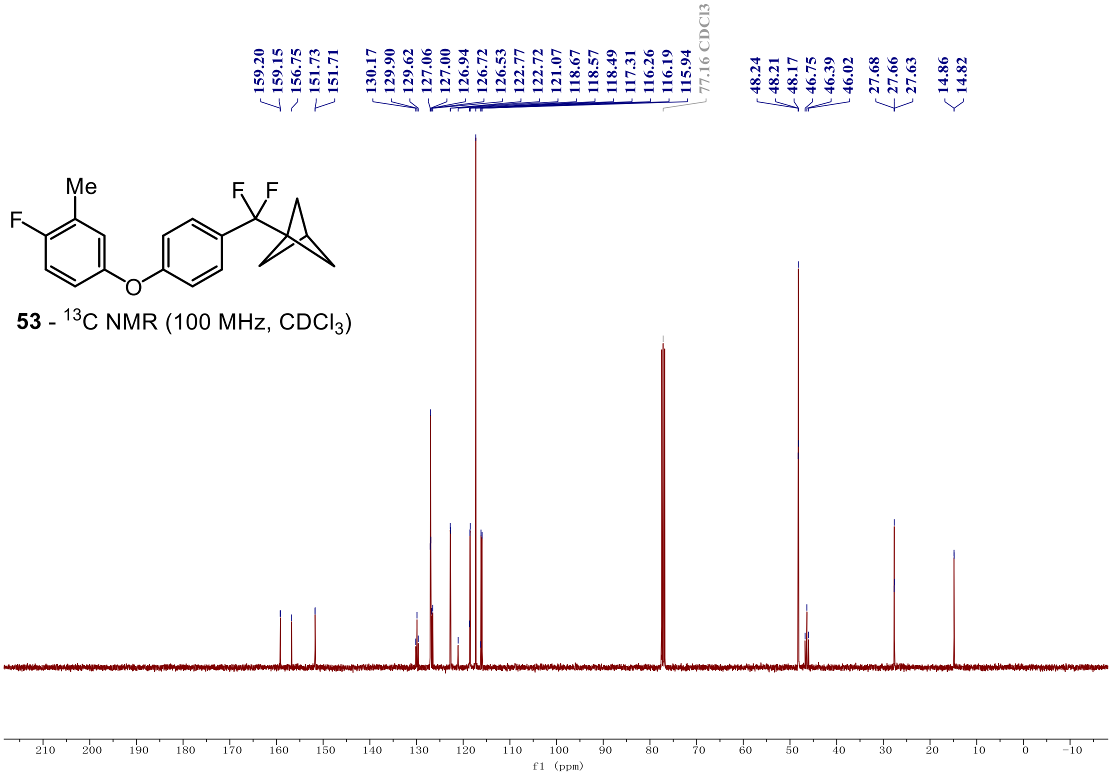

Supplementary Figure 178 ^{13}C NMR spectra of compound **53**

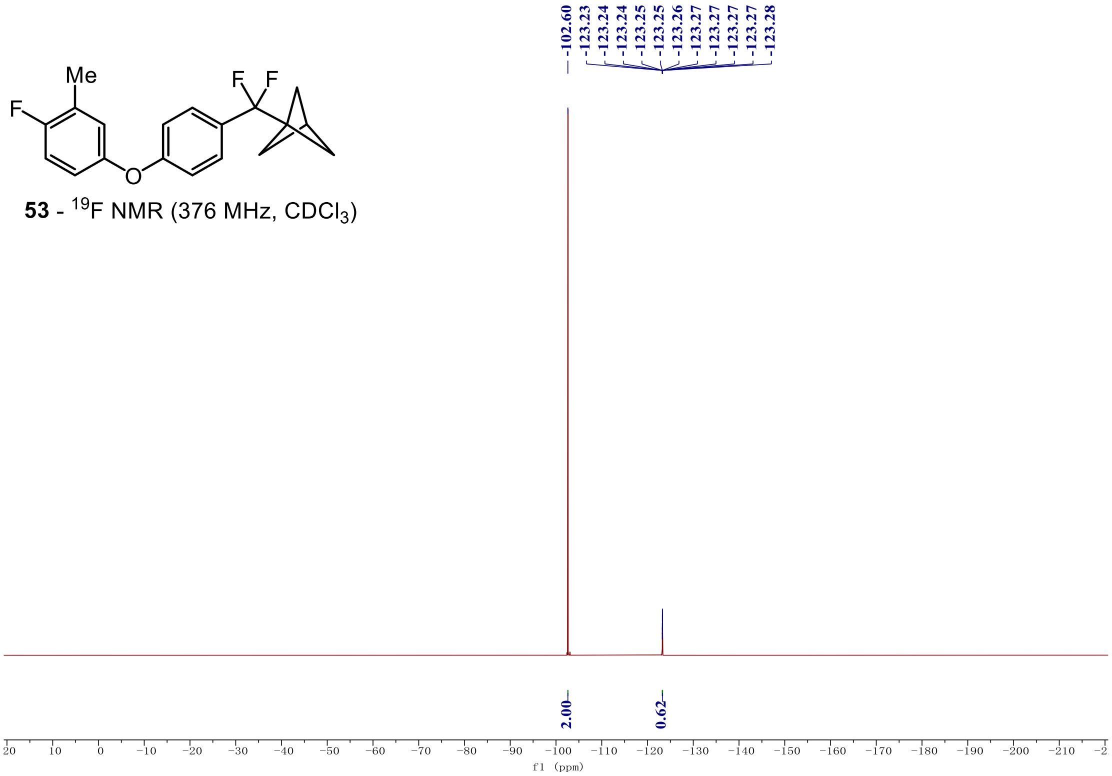

Supplementary Figure 179 ^{19}F NMR spectra of compound **53**

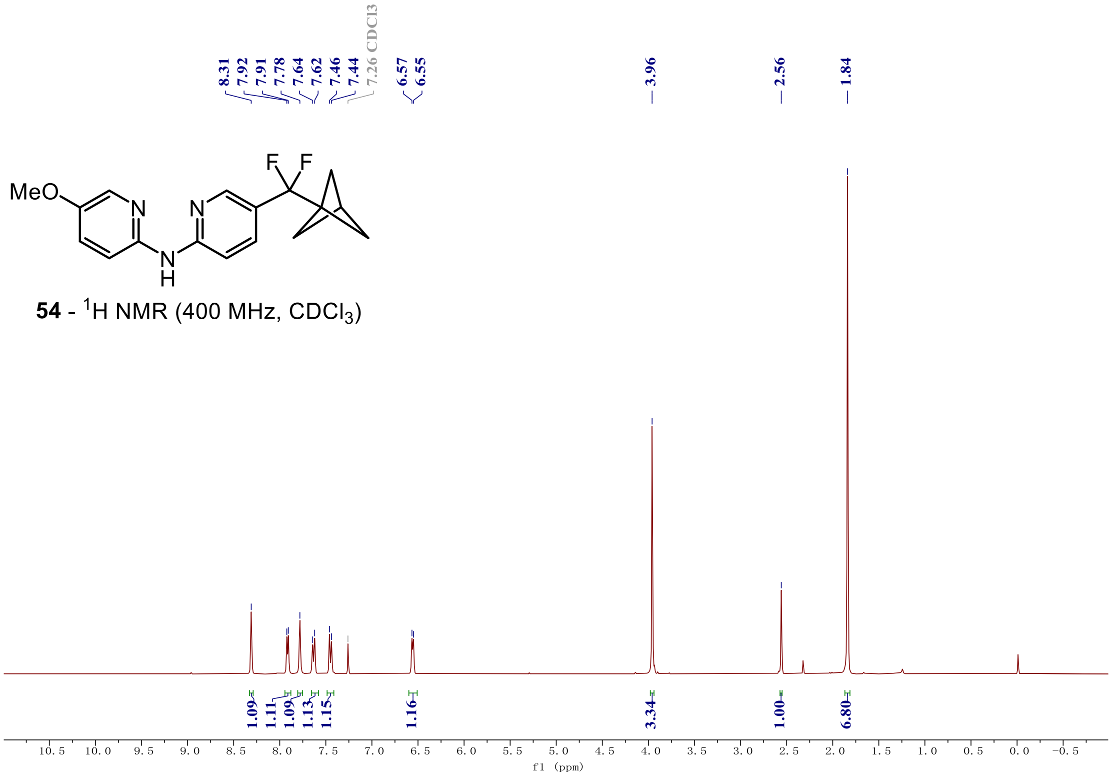

Supplementary Figure 180 ^{1}H NMR spectra of compound **54**

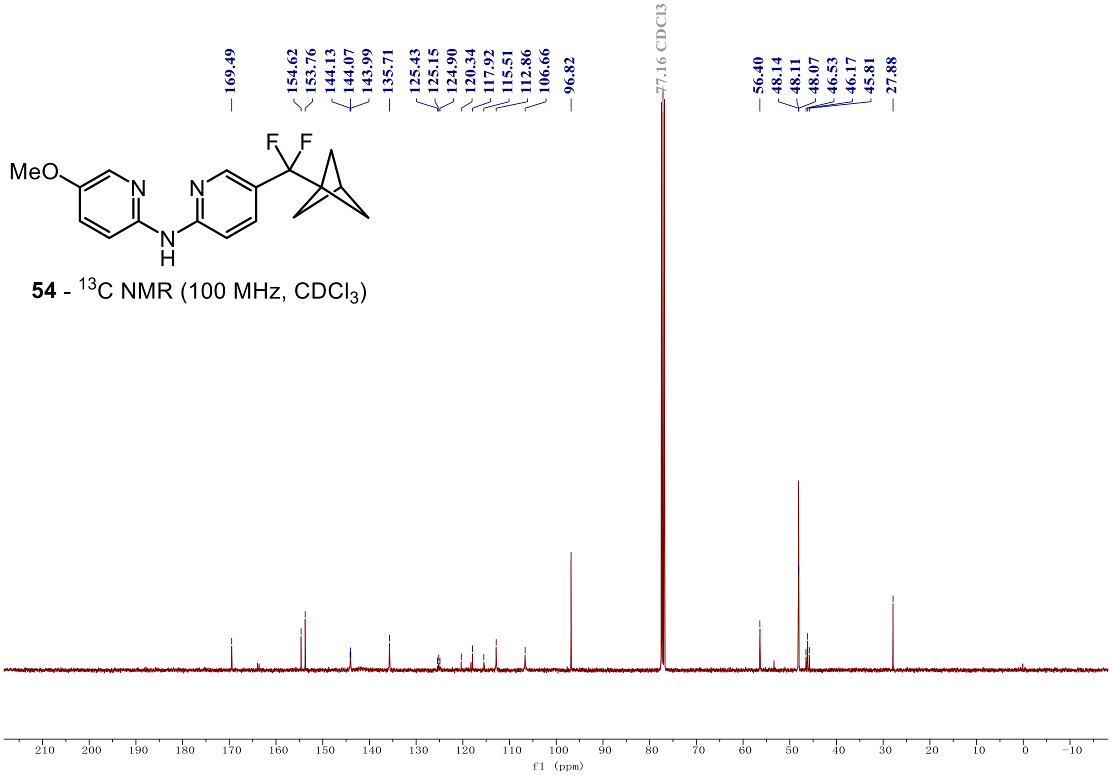

Supplementary Figure 181 ^{13}C NMR spectra of compound **54**

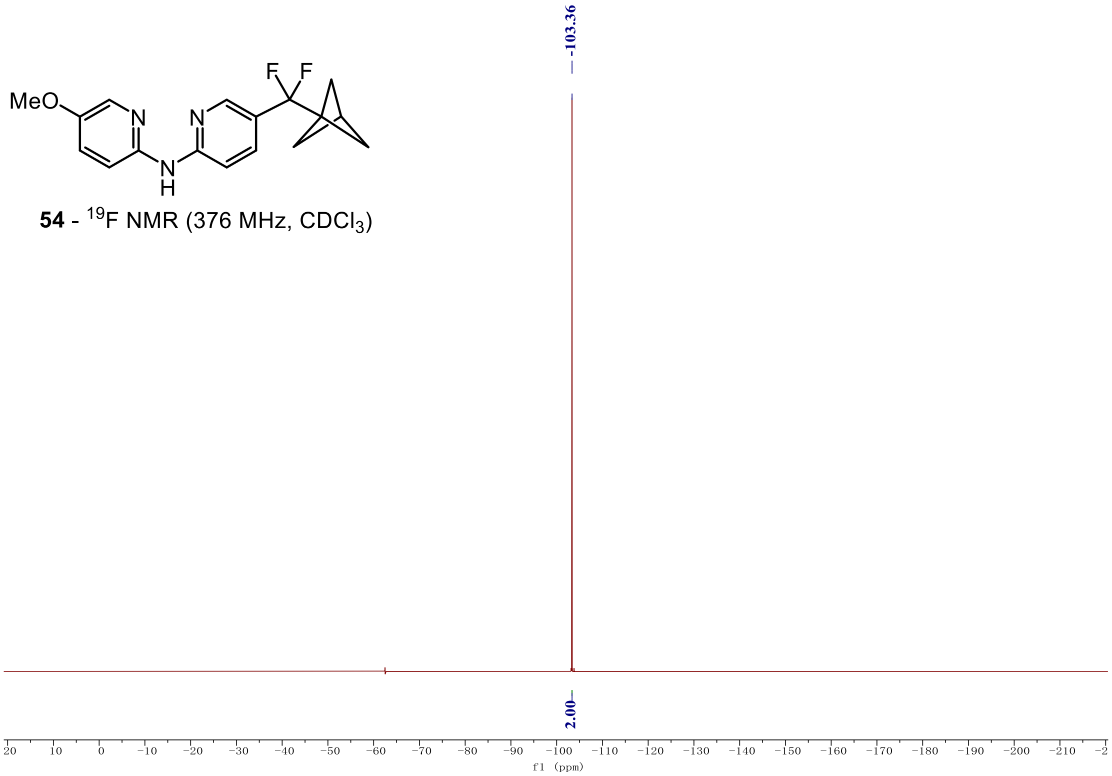

Supplementary Figure 182 ^{19}F NMR spectra of compound **54**

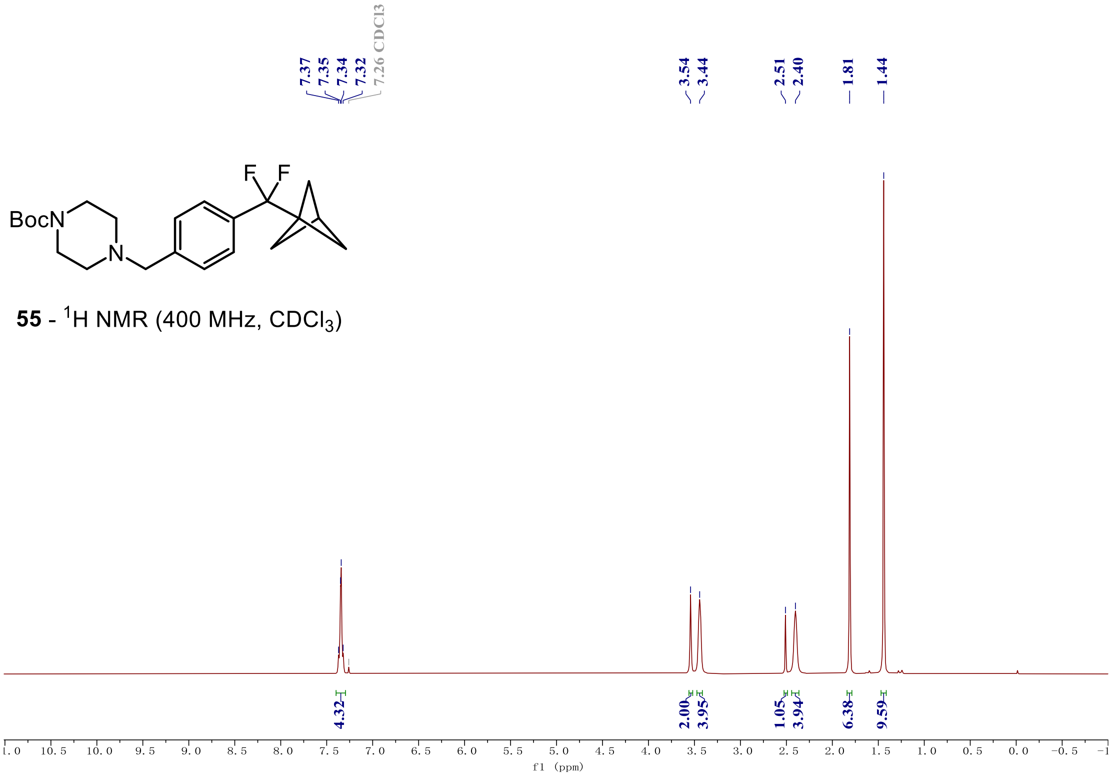

Supplementary Figure 183 ^{1}H NMR spectra of compound **55**

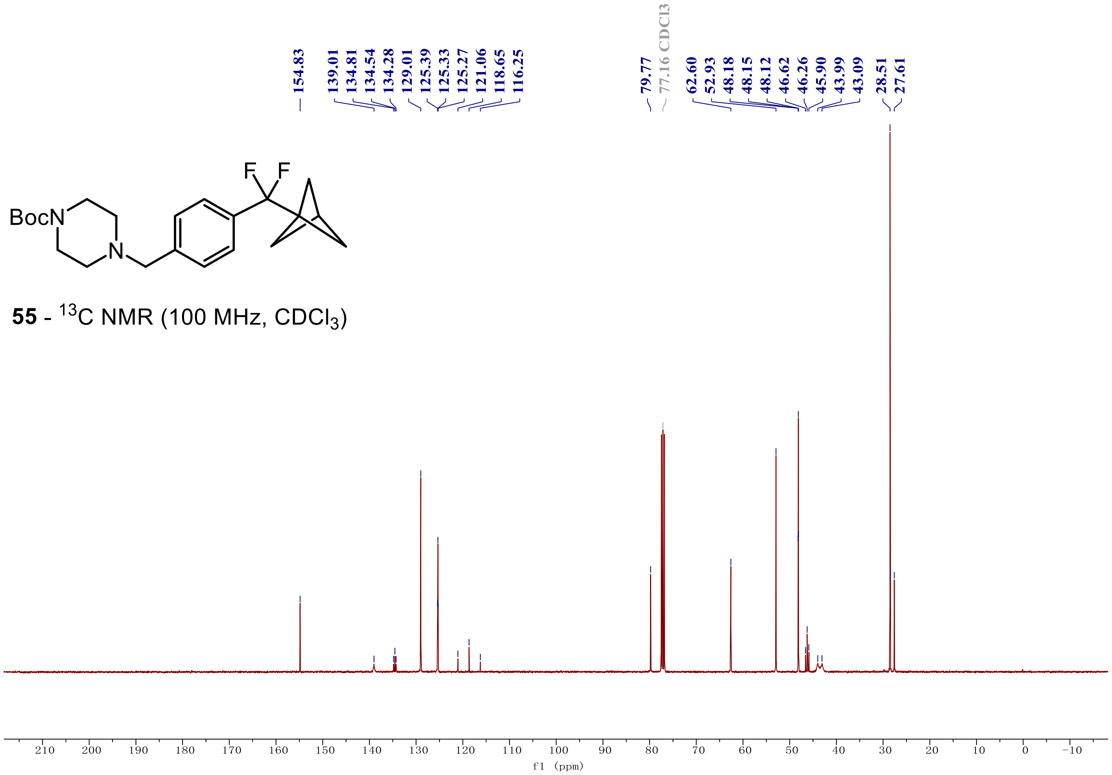

Supplementary Figure 184 ^{13}C NMR spectra of compound **55**

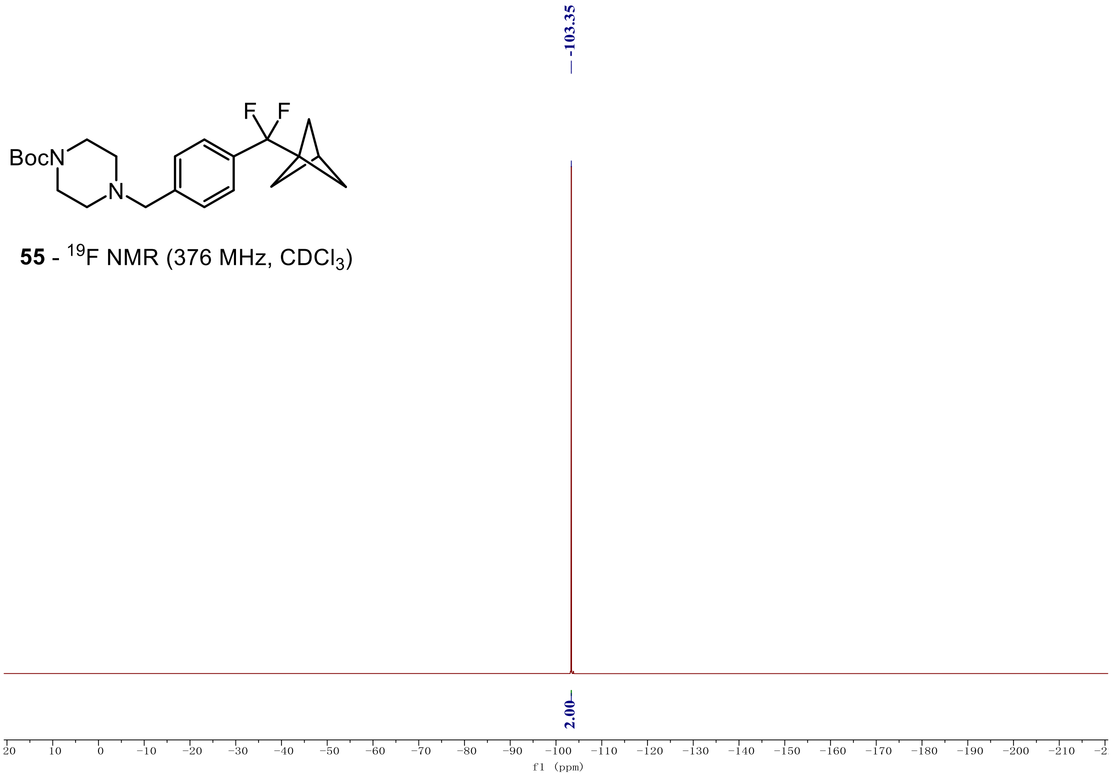

Supplementary Figure 185 ^{19}F NMR spectra of compound **55**

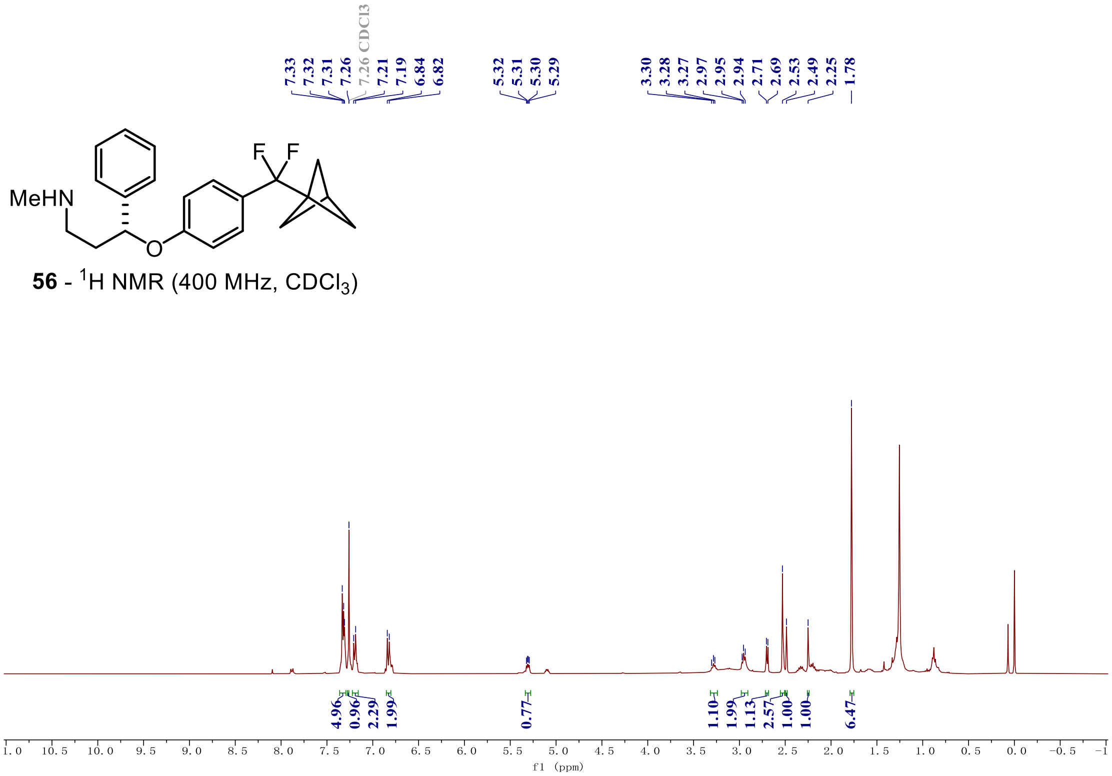

Supplementary Figure 186 ^{1}H NMR spectra of compound **56**

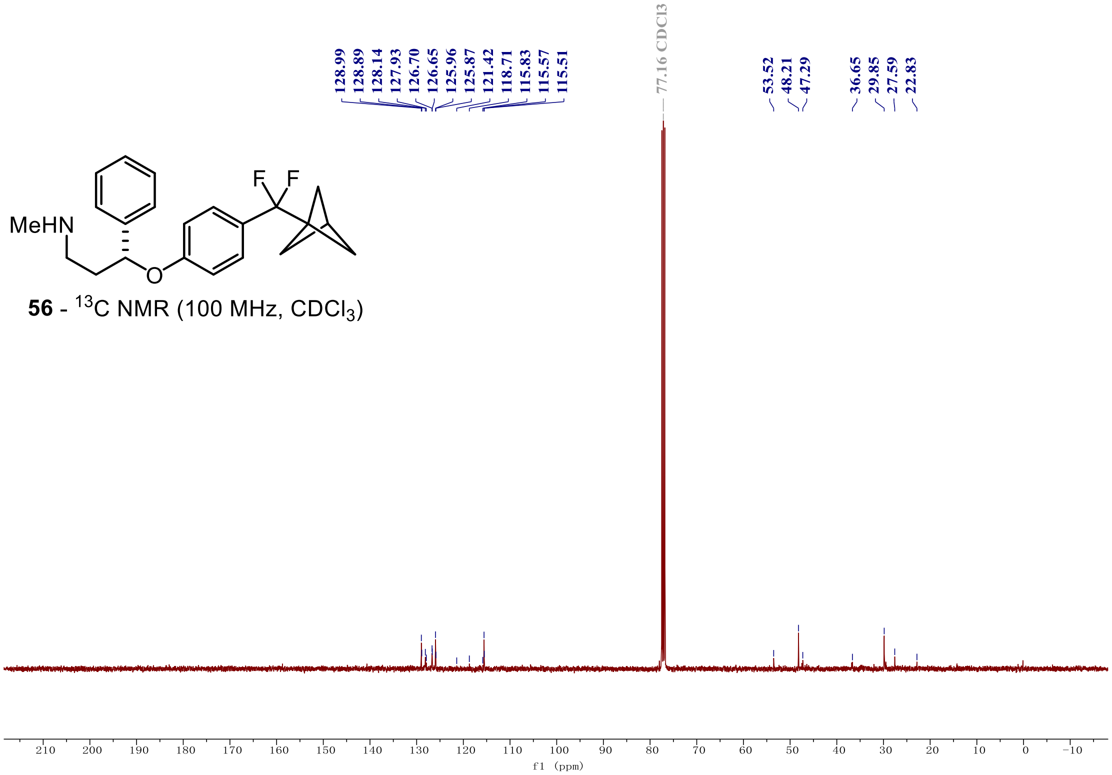

Supplementary Figure 187 ^{13}C NMR spectra of compound **56**

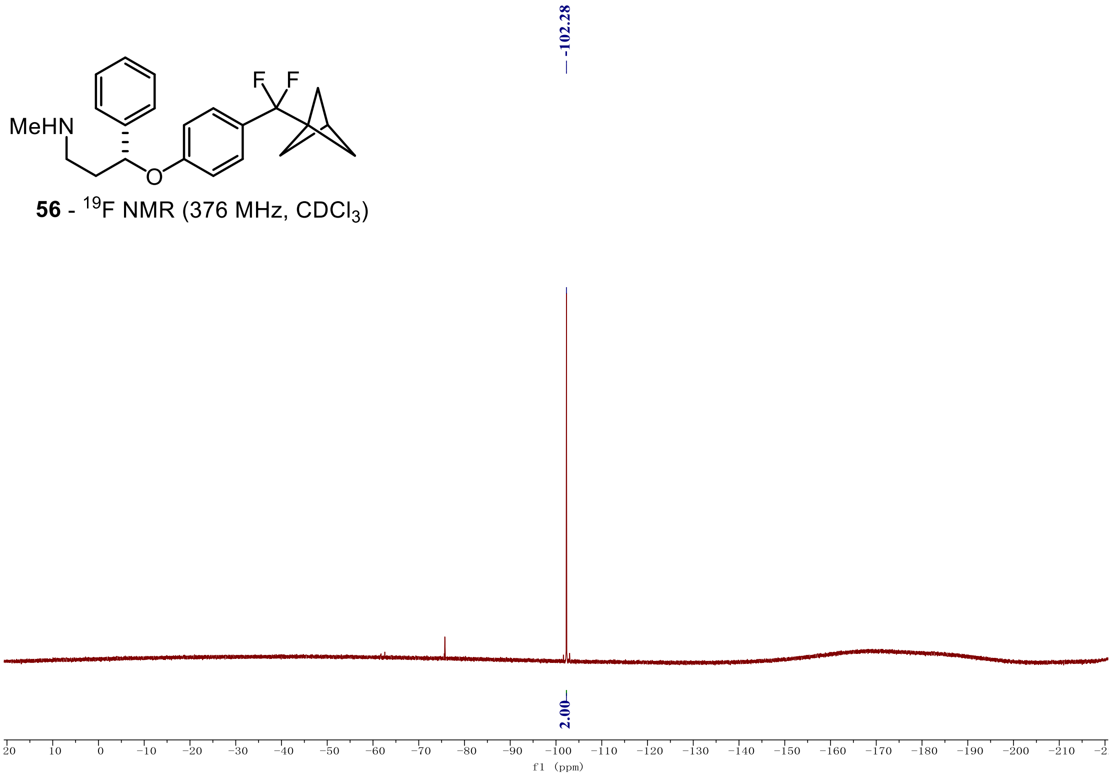

Supplementary Figure 188 ^{19}F NMR spectra of compound **56**

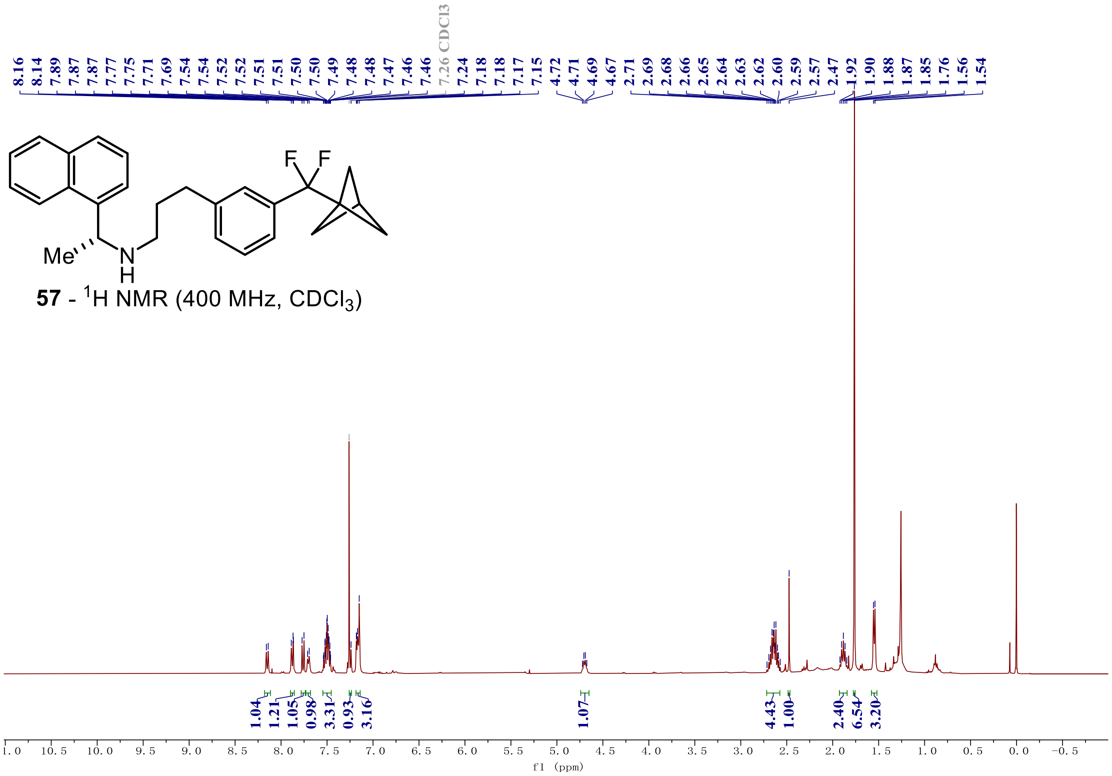

Supplementary Figure 189 ^{1}H NMR spectra of compound **57**

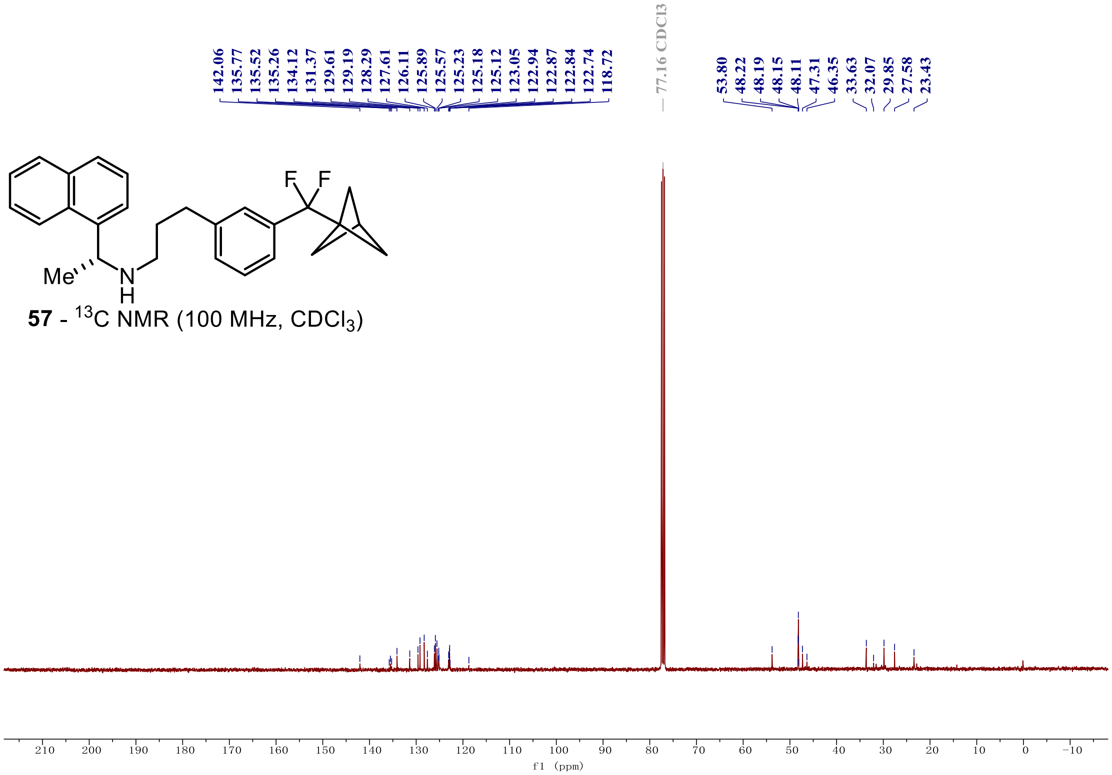

Supplementary Figure 190 ^{13}C NMR spectra of compound **57**

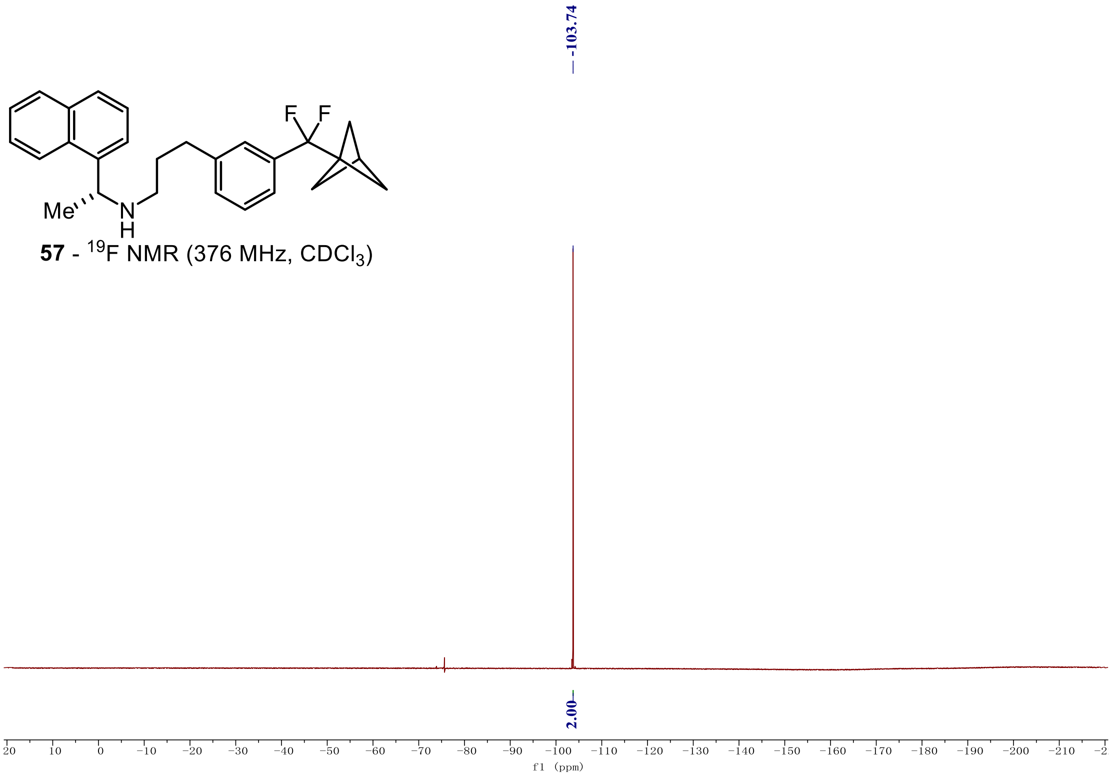

Supplementary Figure 191 ^{19}F NMR spectra of compound **57**

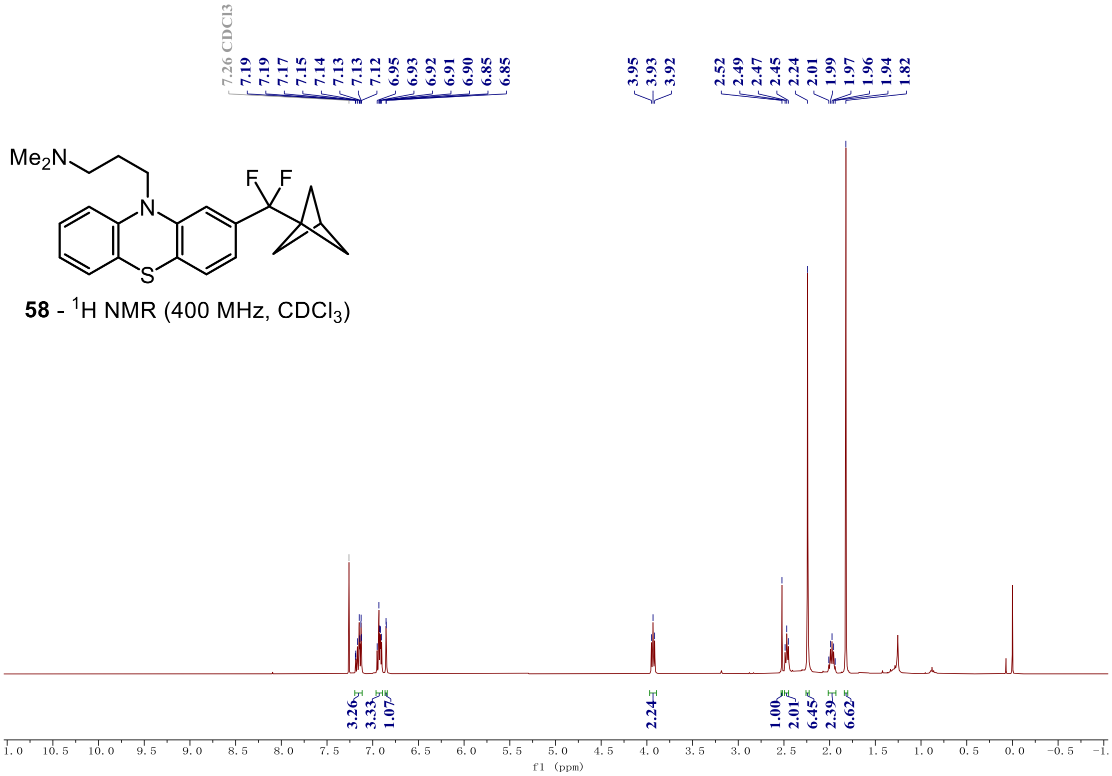

Supplementary Figure 192 ^{1}H NMR spectra of compound **58**

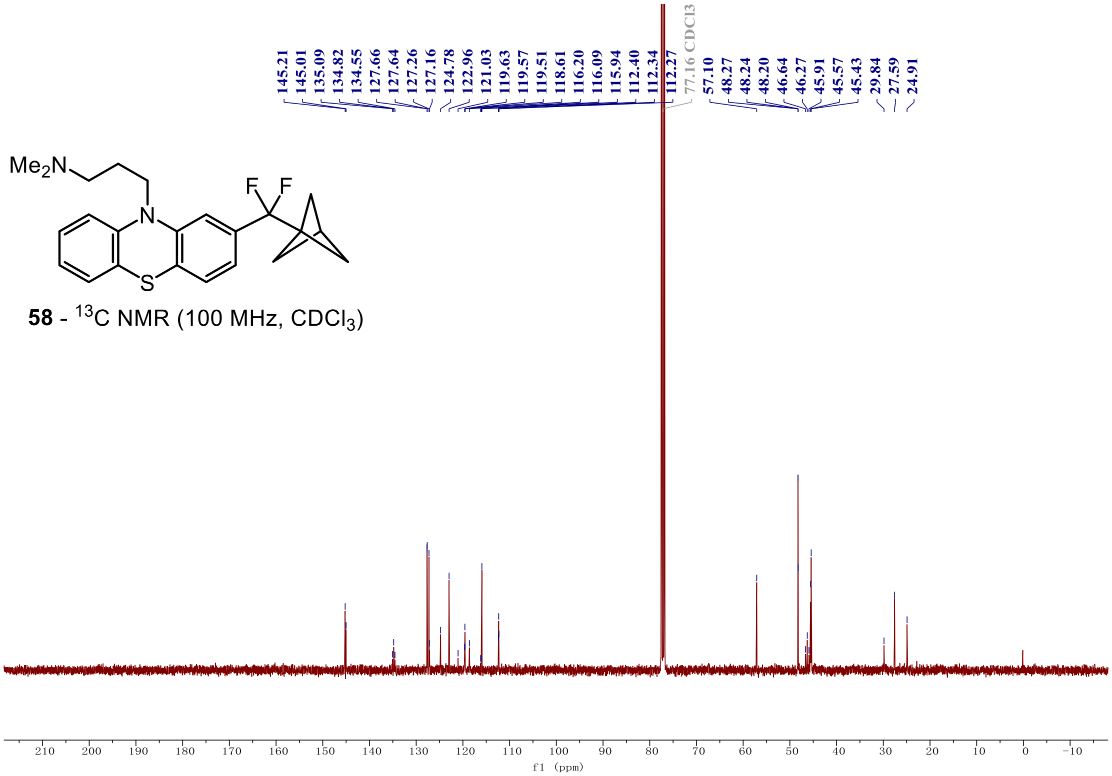

Supplementary Figure 193 ^{13}C NMR spectra of compound **58**

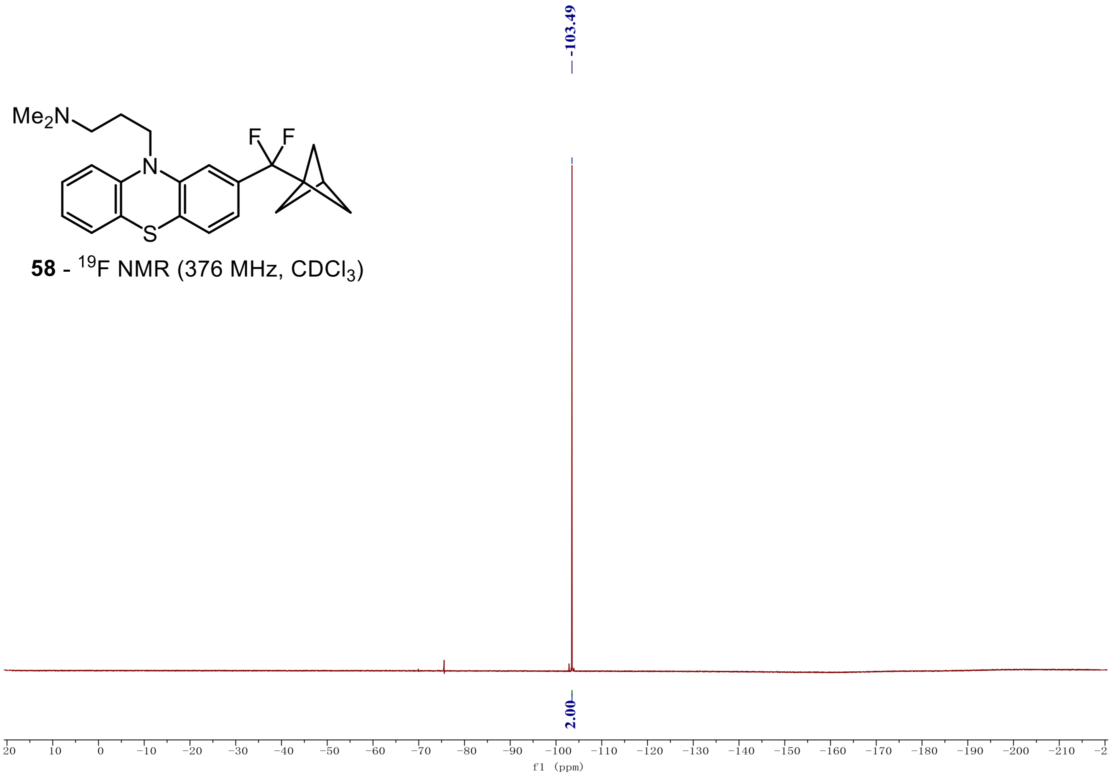

Supplementary Figure 194 ^{19}F NMR spectra of compound **58**

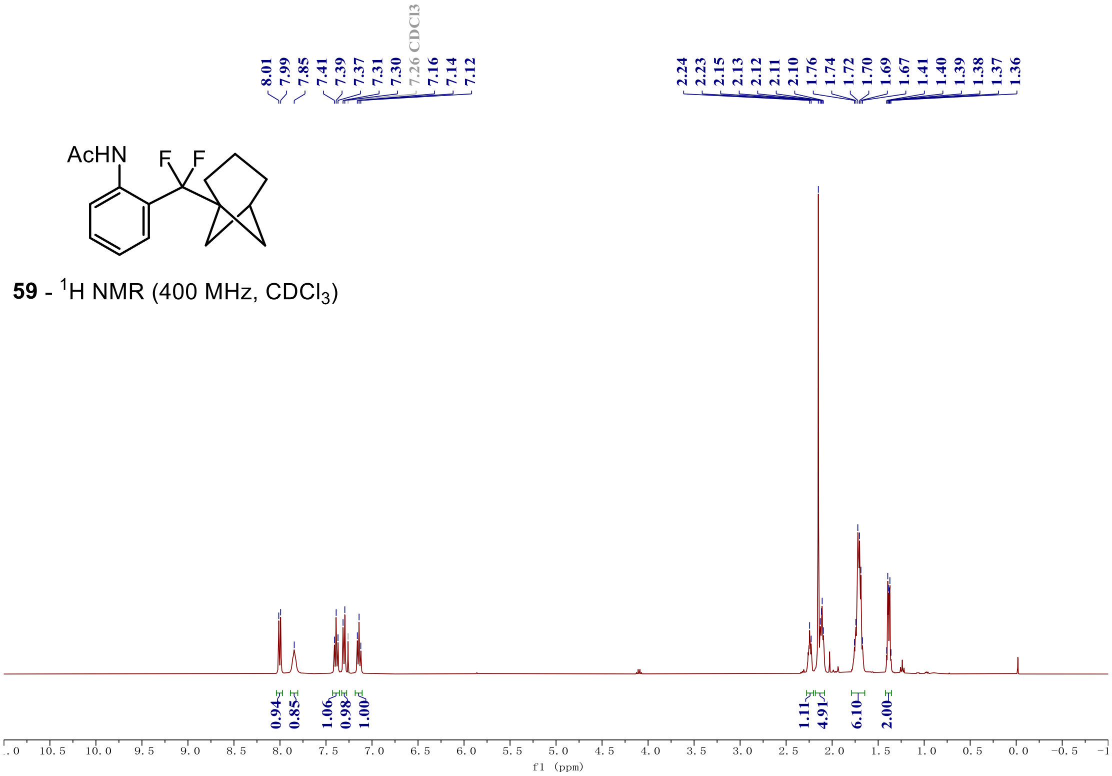

Supplementary Figure 195 ^{1}H NMR spectra of compound **59**

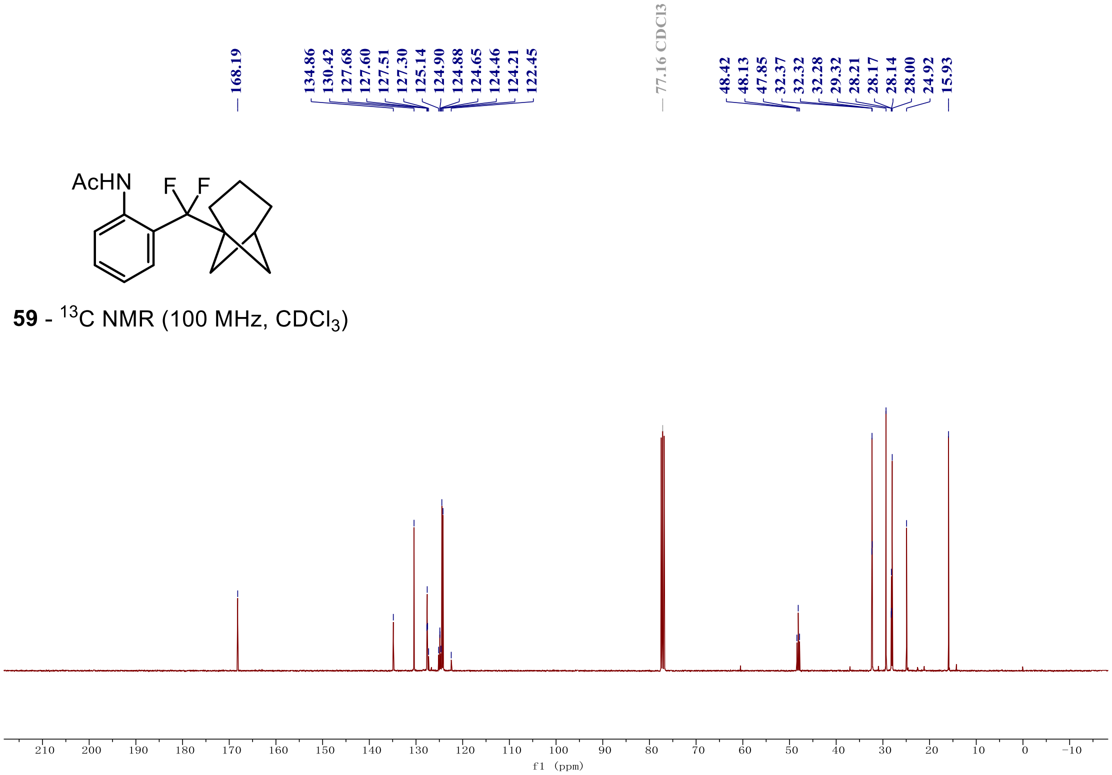

Supplementary Figure 196 ^{13}C NMR spectra of compound **59**

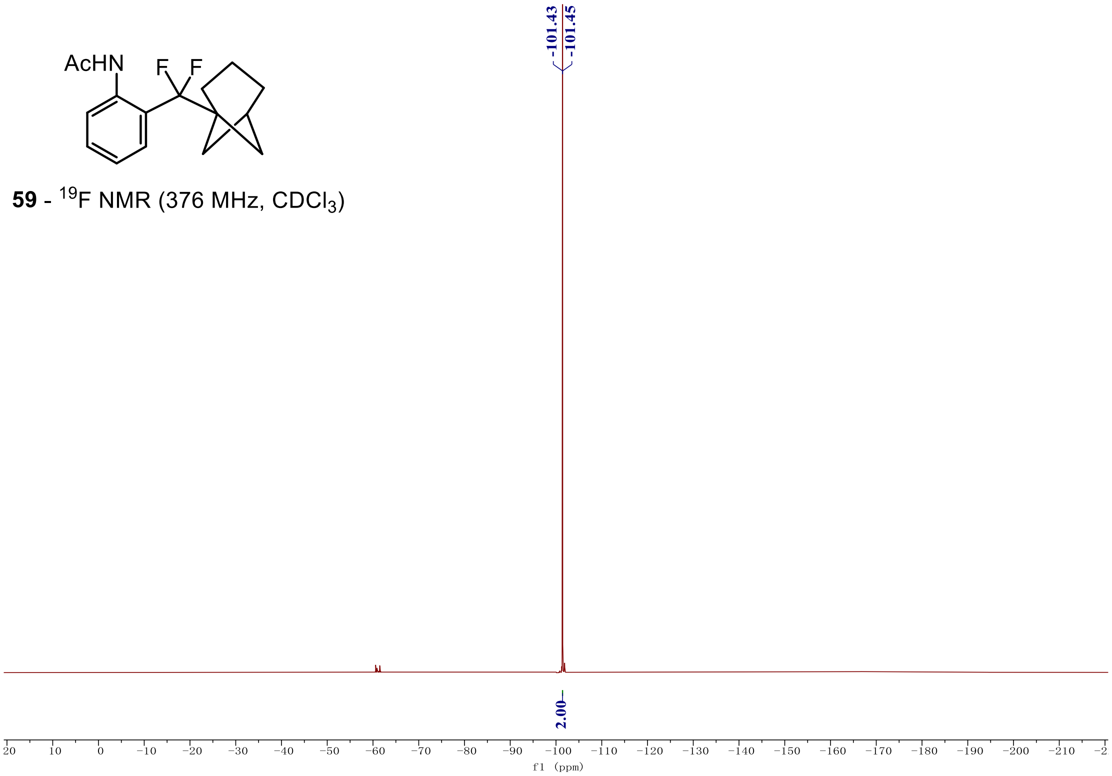

Supplementary Figure 197 ^{19}F NMR spectra of compound **59**

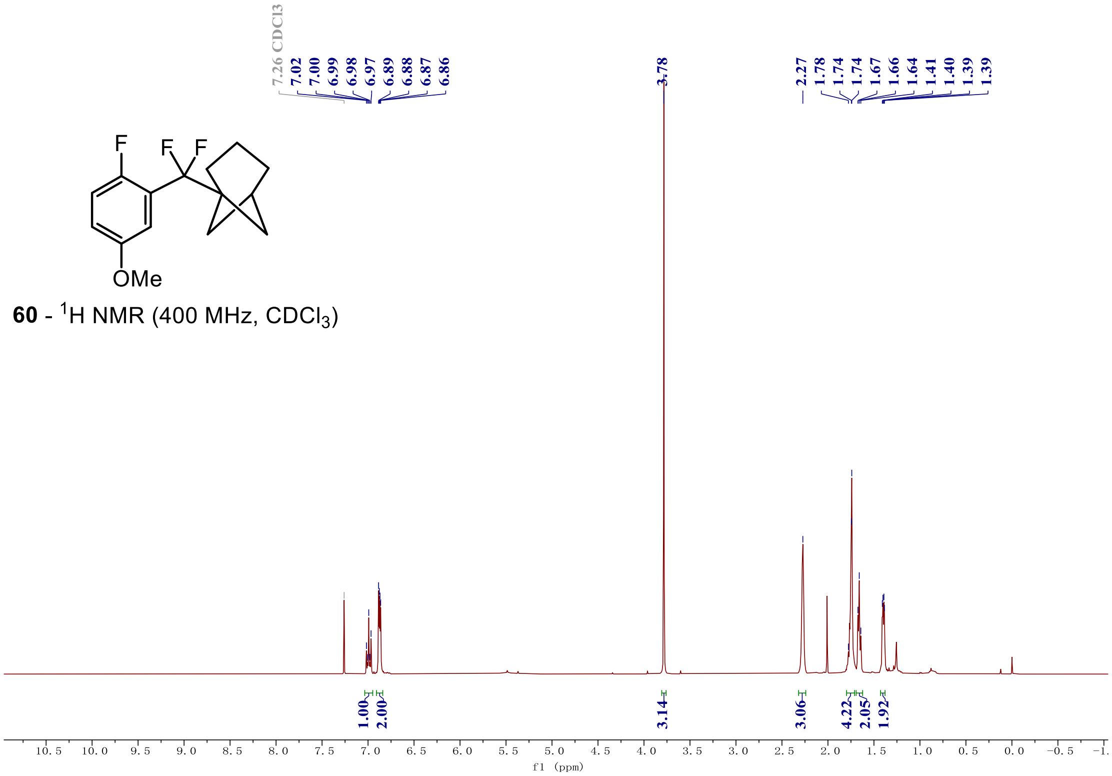

Supplementary Figure 198 ^{1}H NMR spectra of compound **60**

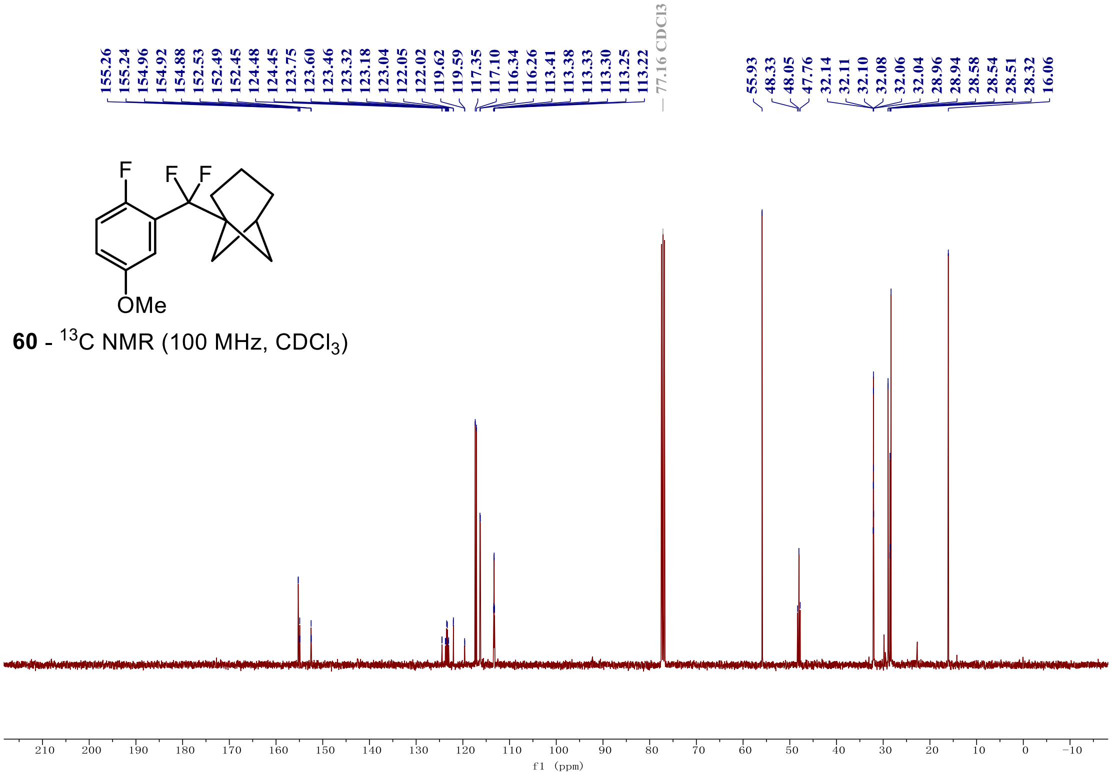

Supplementary Figure 199 ^{13}C NMR spectra of compound **60**

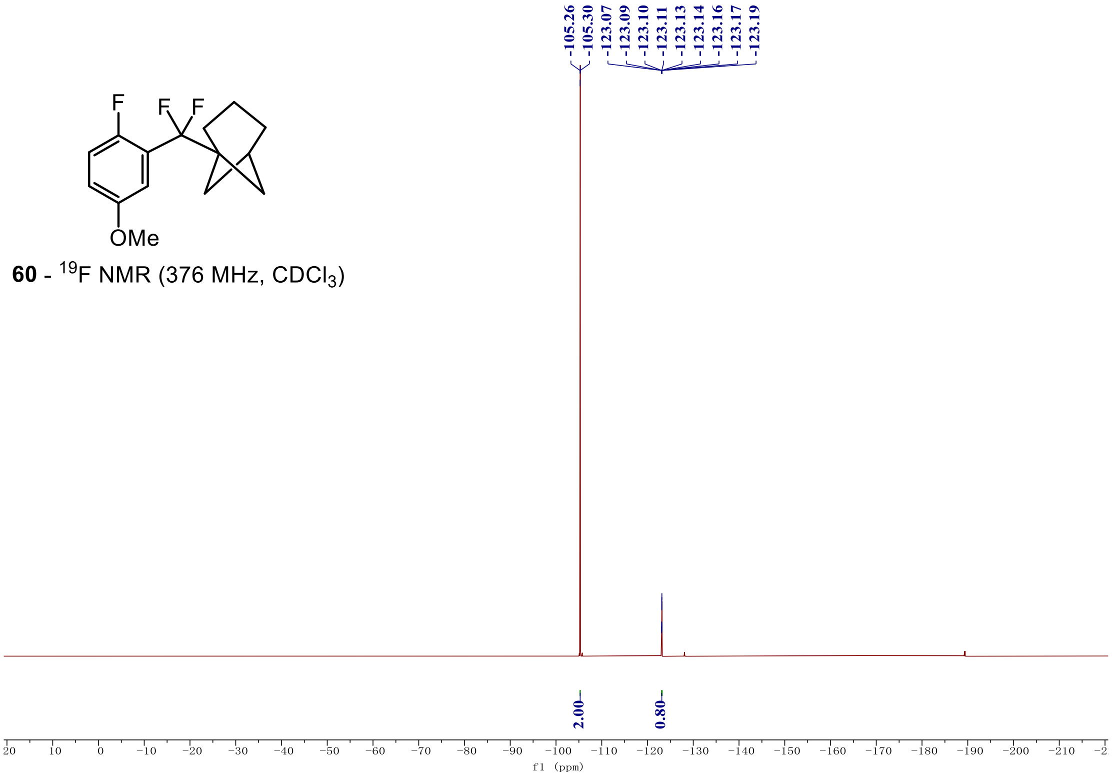

60 - ^{19}F NMR (376 MHz, $CDCl_3$)

Supplementary Figure 200 ^{19}F NMR spectra of compound **60**

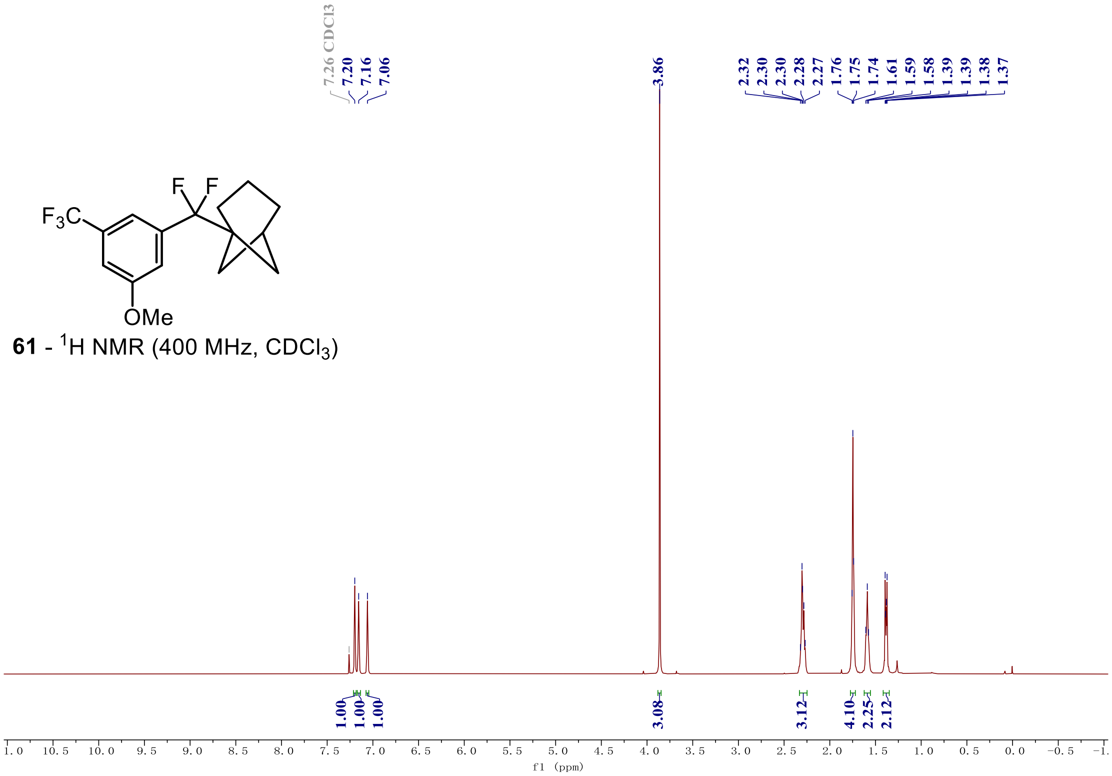

Supplementary Figure 201 ^{1}H NMR spectra of compound **61**

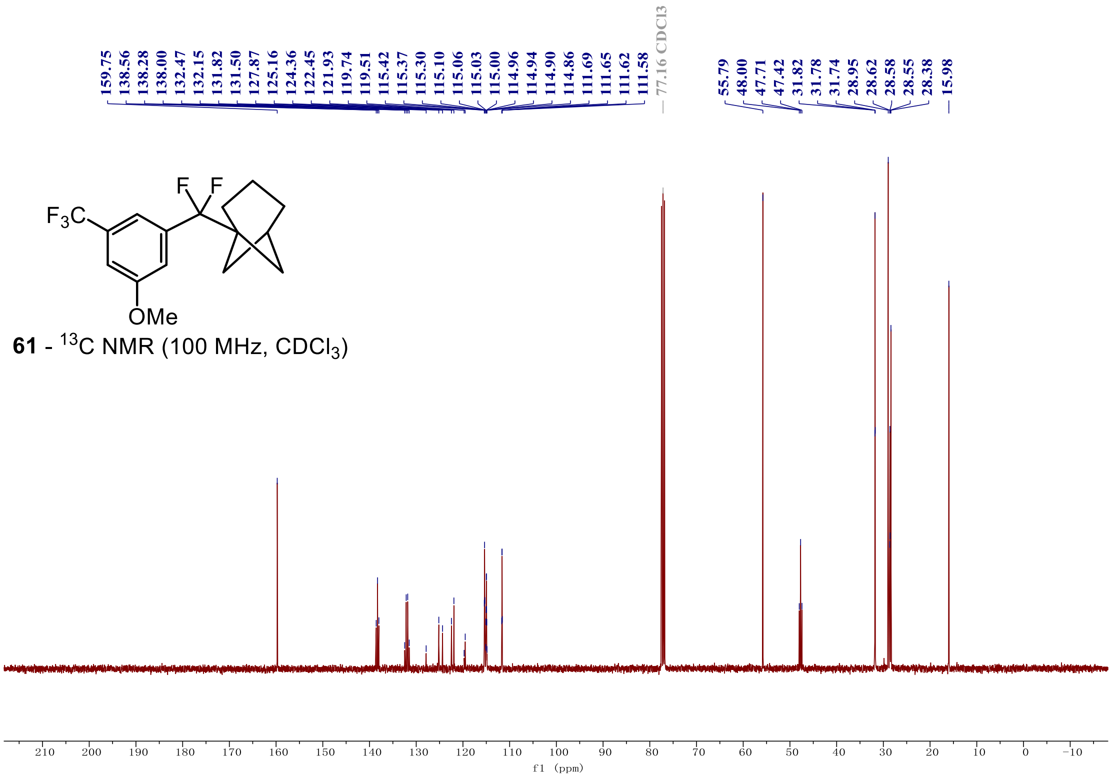

Supplementary Figure 202 ^{13}C NMR spectra of compound **61**

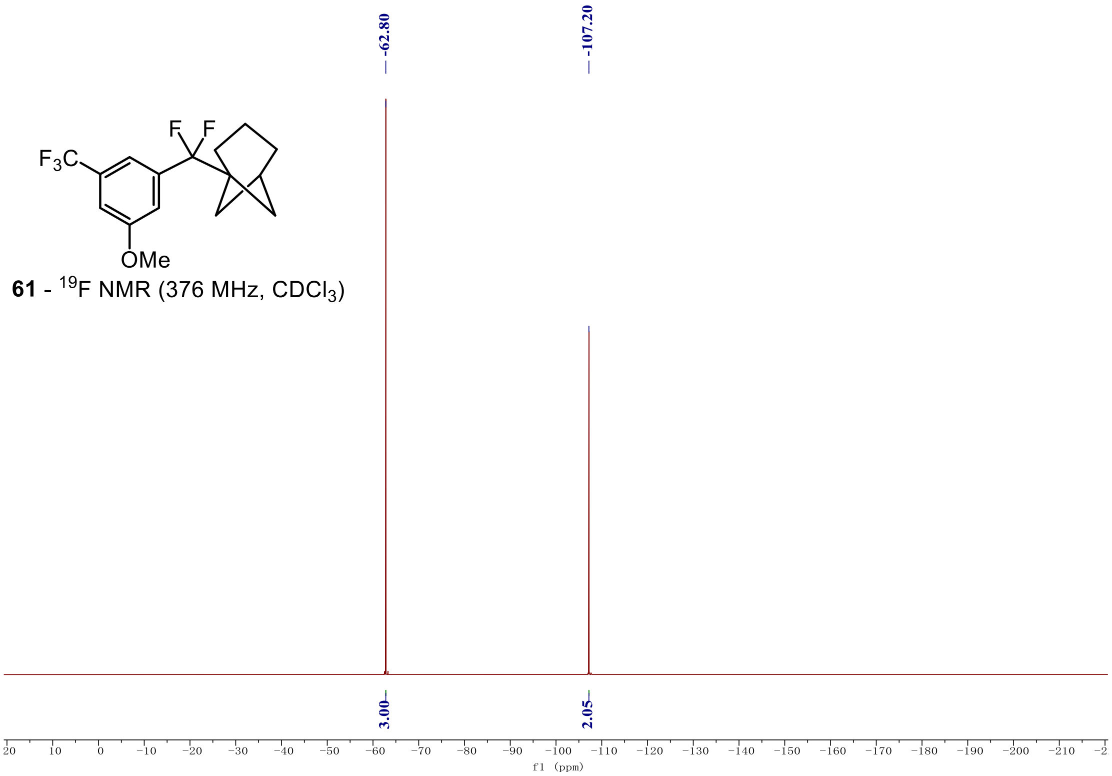

Supplementary Figure 203 ^{19}F NMR spectra of compound **61**

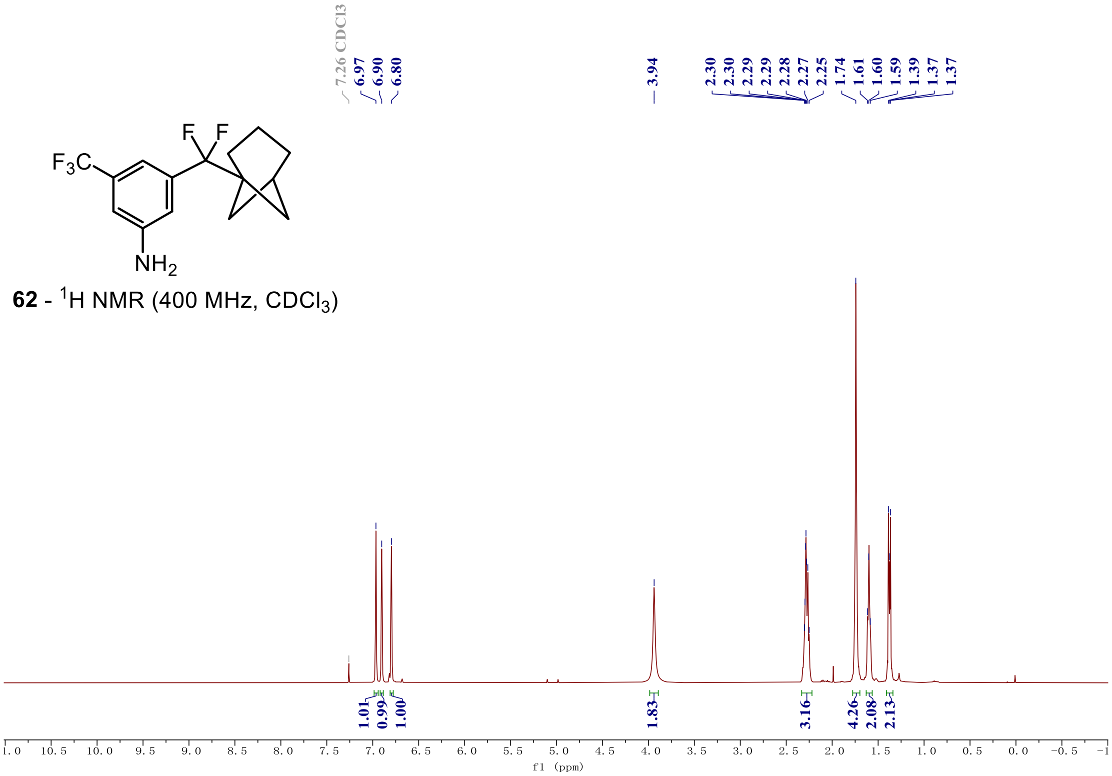

Supplementary Figure 204 ^{1}H NMR spectra of compound **62**

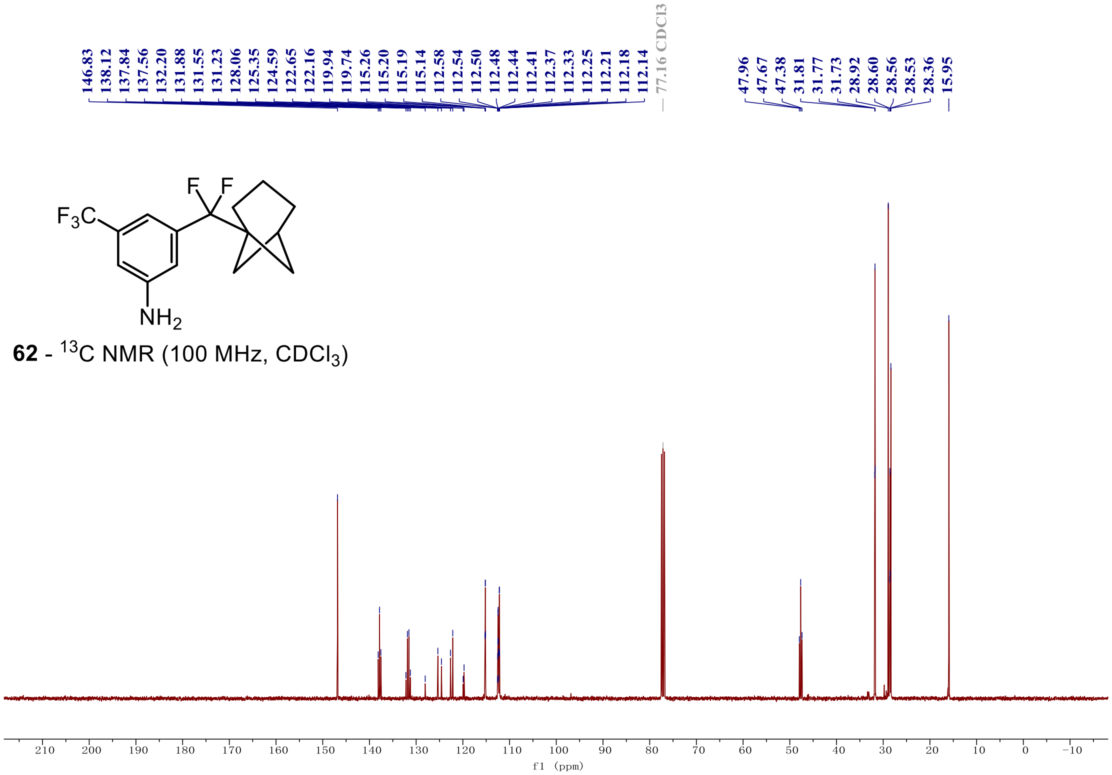

Supplementary Figure 205 ^{13}C NMR spectra of compound **62**

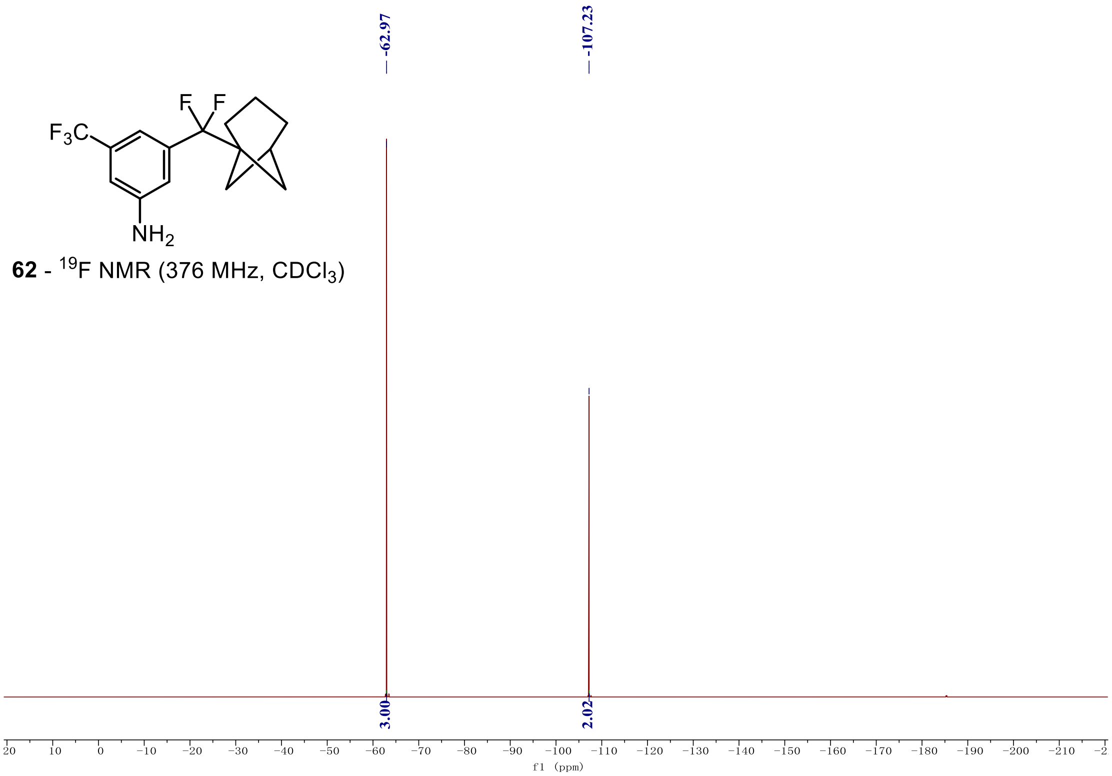

Supplementary Figure 206 ^{19}F NMR spectra of compound **62**

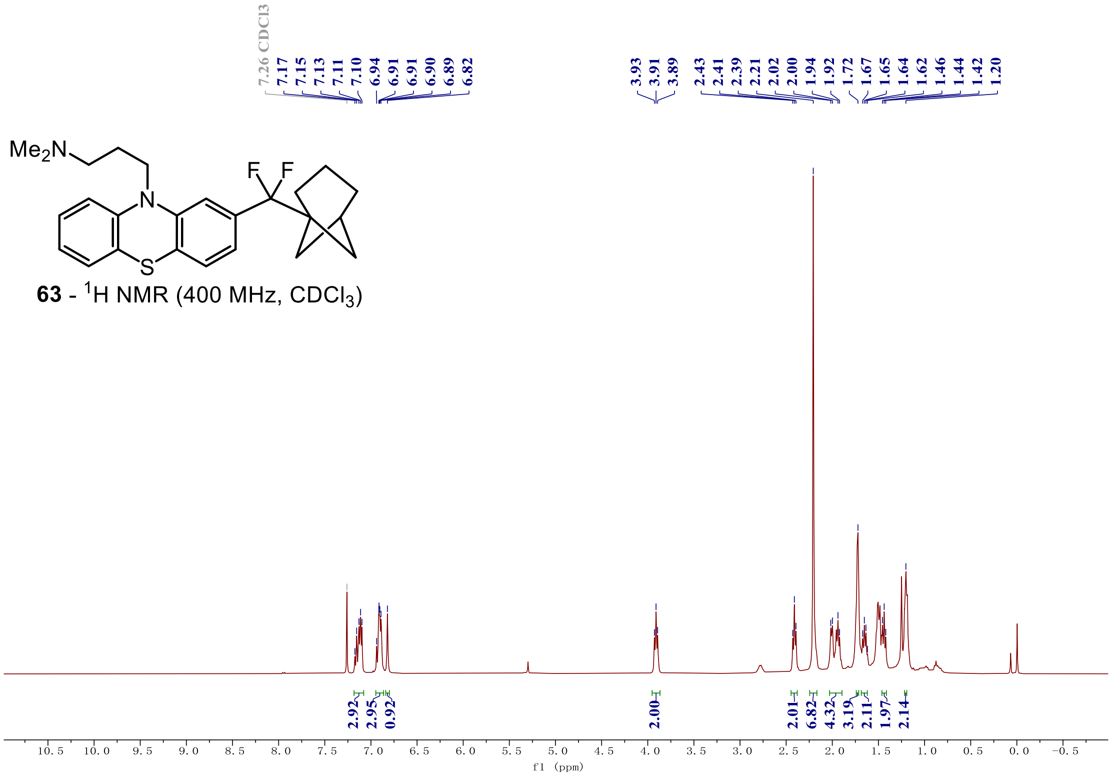

Supplementary Figure 207 ^{1}H NMR spectra of compound **63**

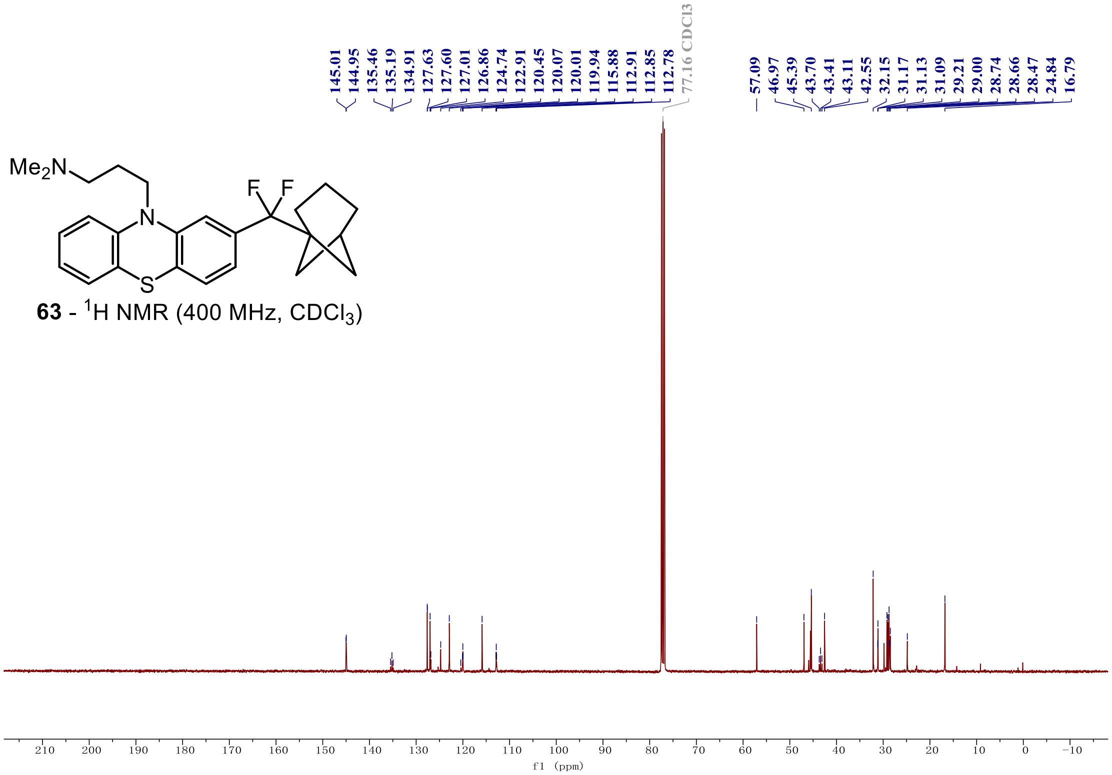

Supplementary Figure 208 ^{13}C NMR spectra of compound **63**

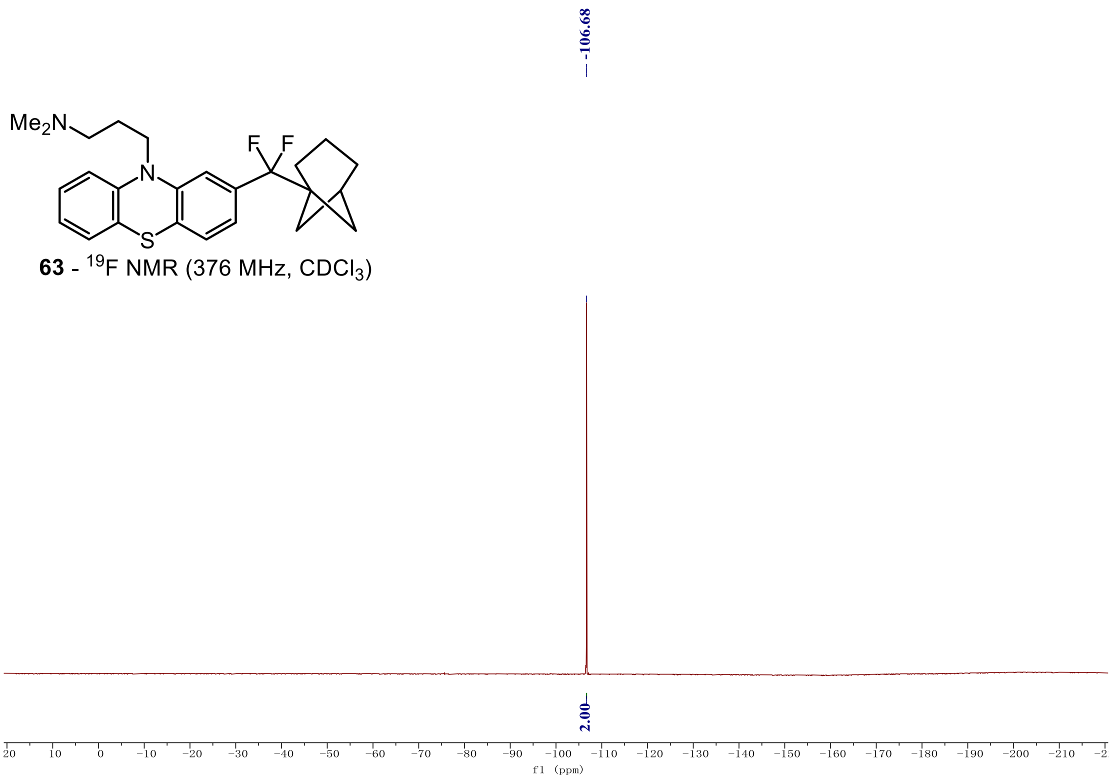

Supplementary Figure 209 ^{19}F NMR spectra of compound **63**

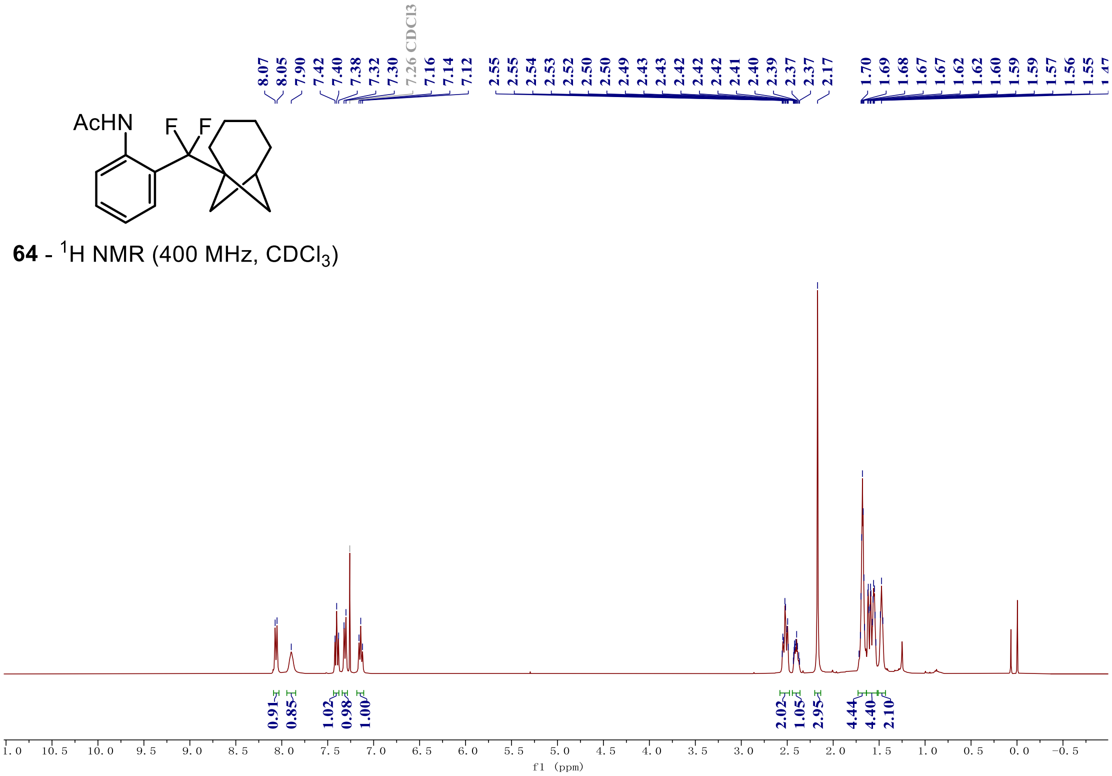

64 - ^{1}H NMR (400 MHz, $CDCl_3$)

Supplementary Figure 210 ^{1}H NMR spectra of compound **64**

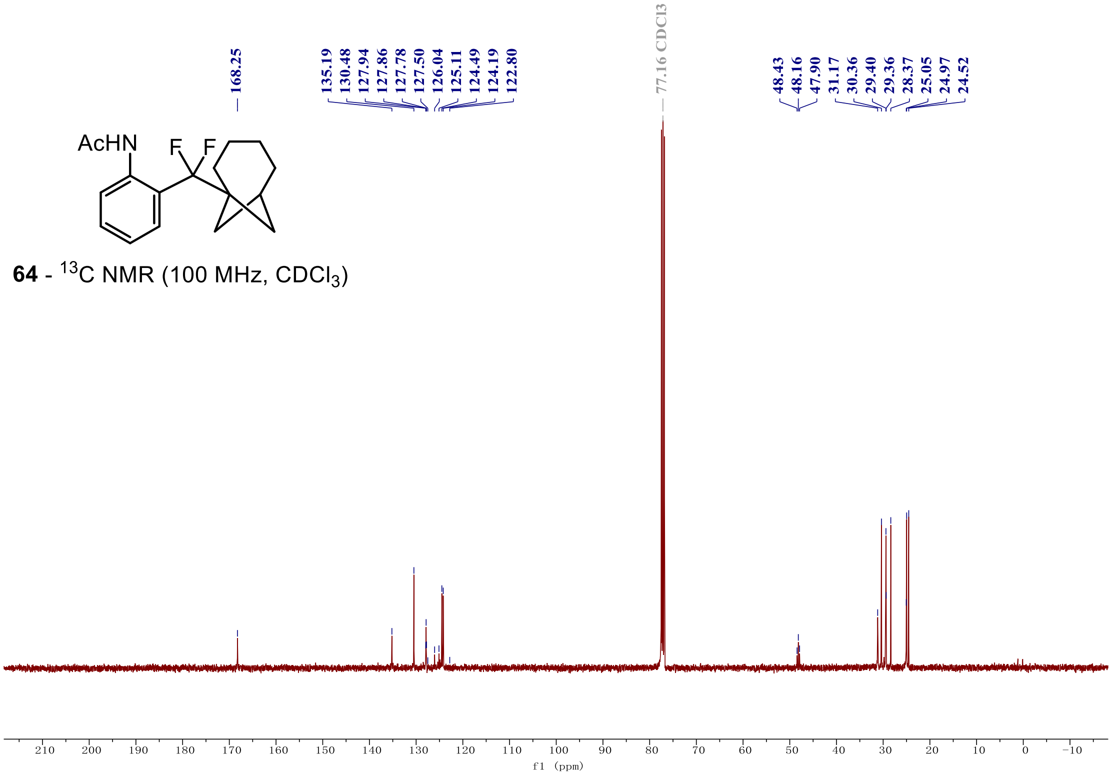

64 - ^{13}C NMR (100 MHz, $CDCl_3$)

Supplementary Figure 211 ^{13}C NMR spectra of compound **64**

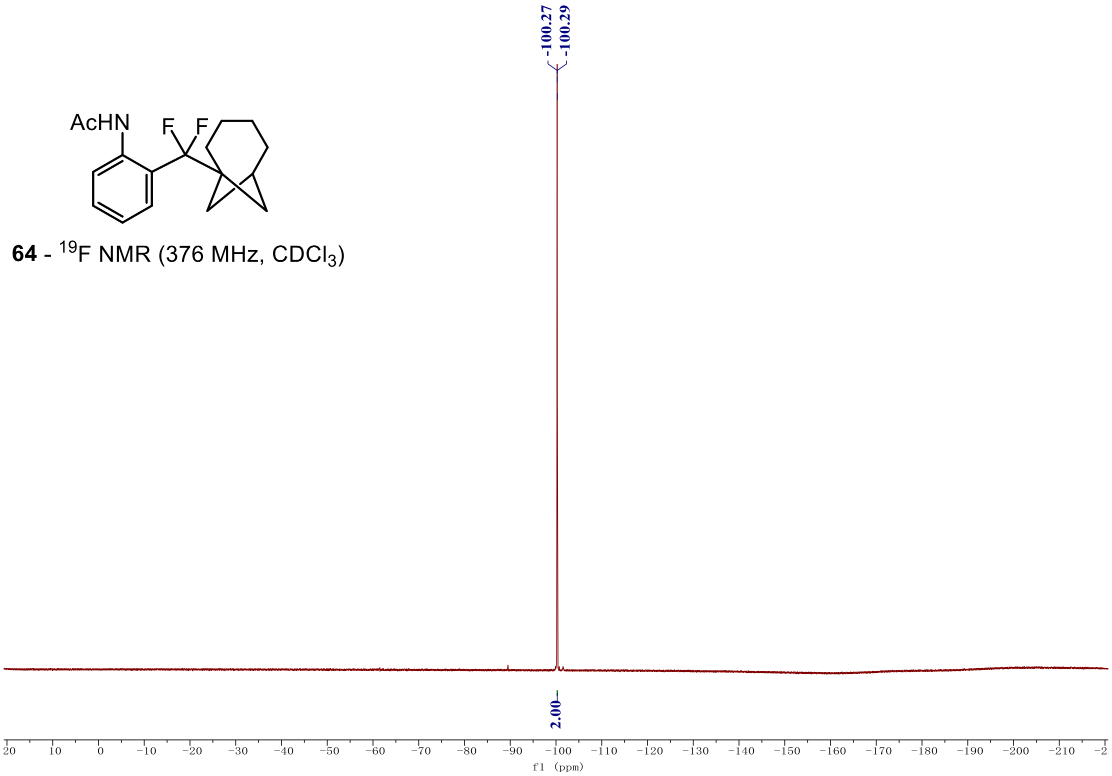

Supplementary Figure 212 ^{19}F NMR spectra of compound **64**

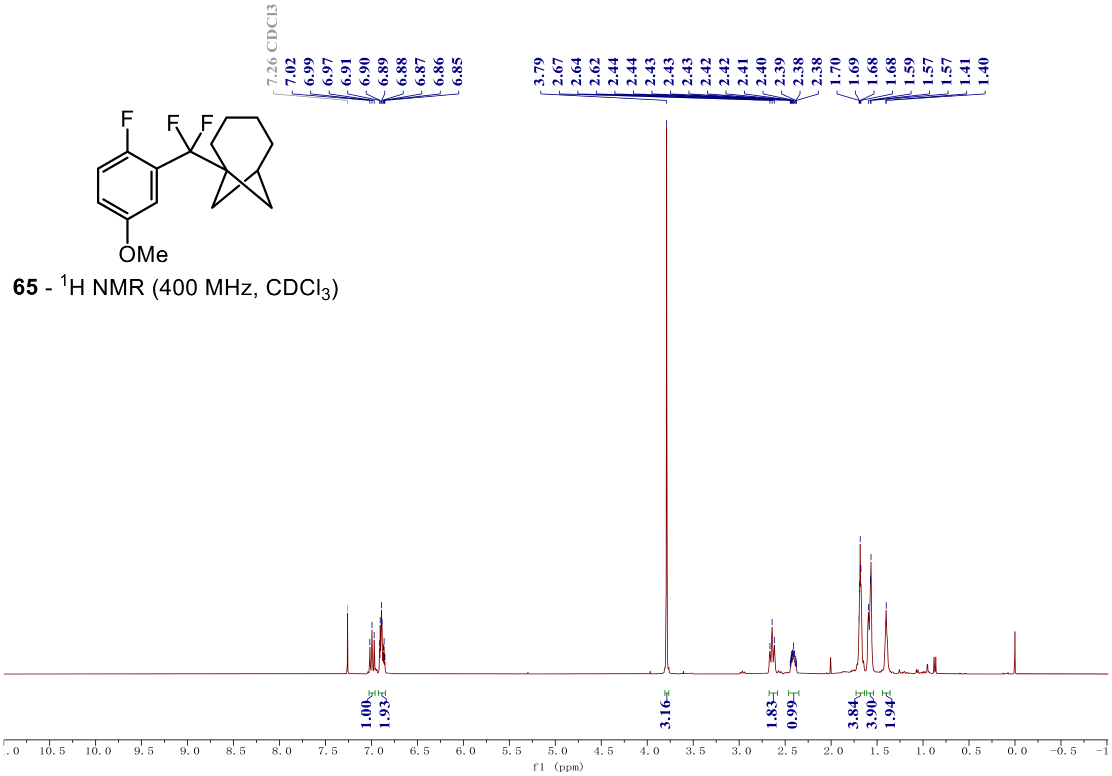

Supplementary Figure 213 ^{1}H NMR spectra of compound **65**

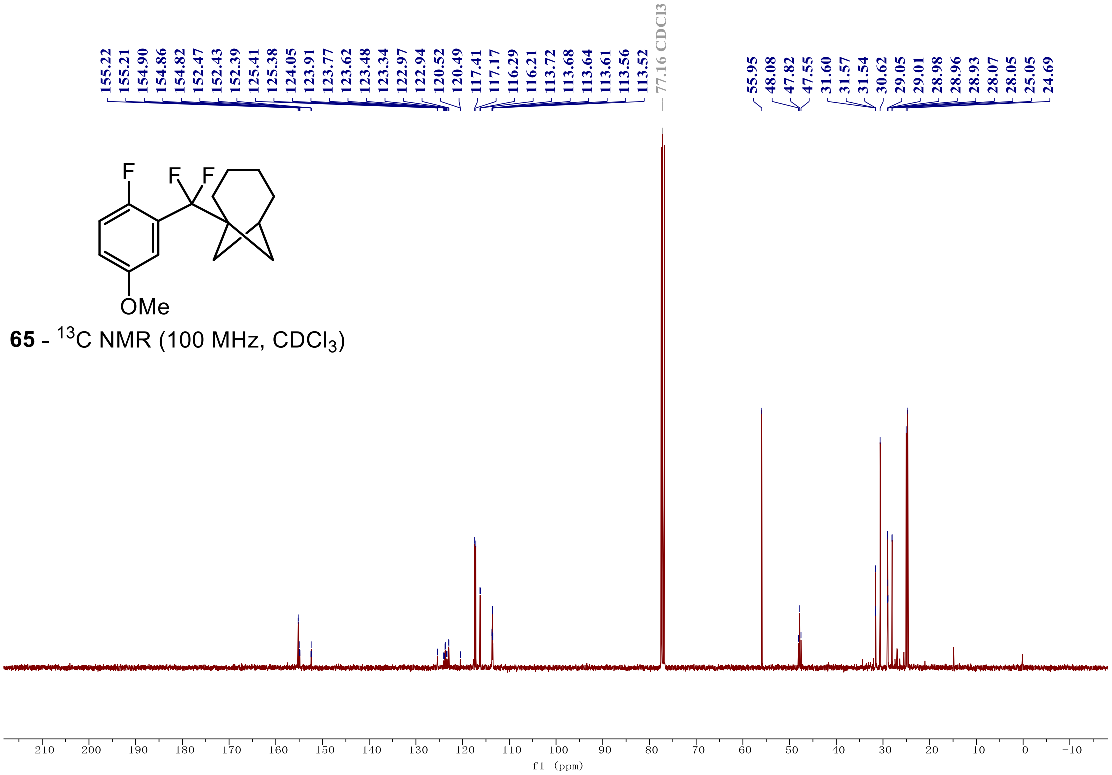

Supplementary Figure 214 ^{13}C NMR spectra of compound **65**

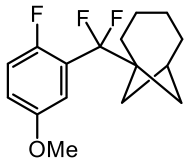

65 - ^{19}F NMR (376 MHz, $CDCl_3$)

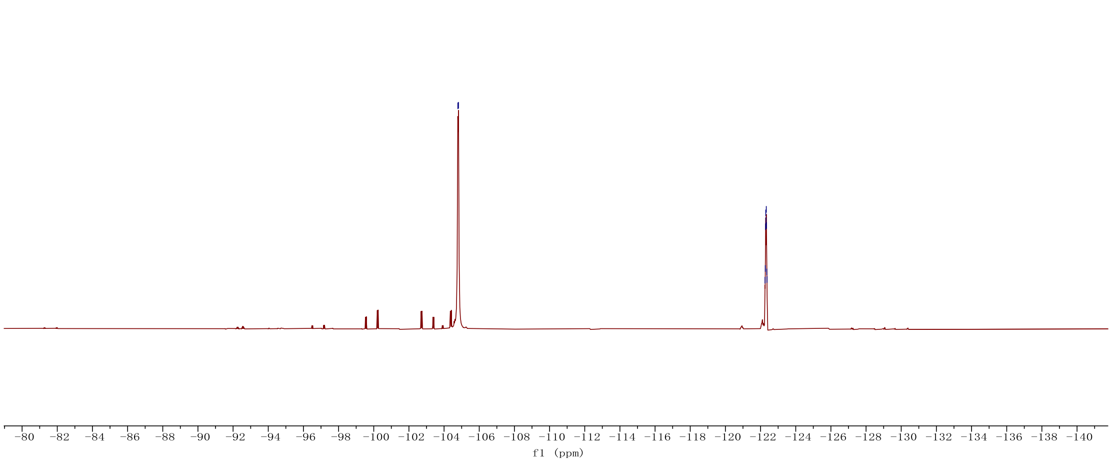

Supplementary Figure 215 ^{19}F NMR spectra of compound **65**

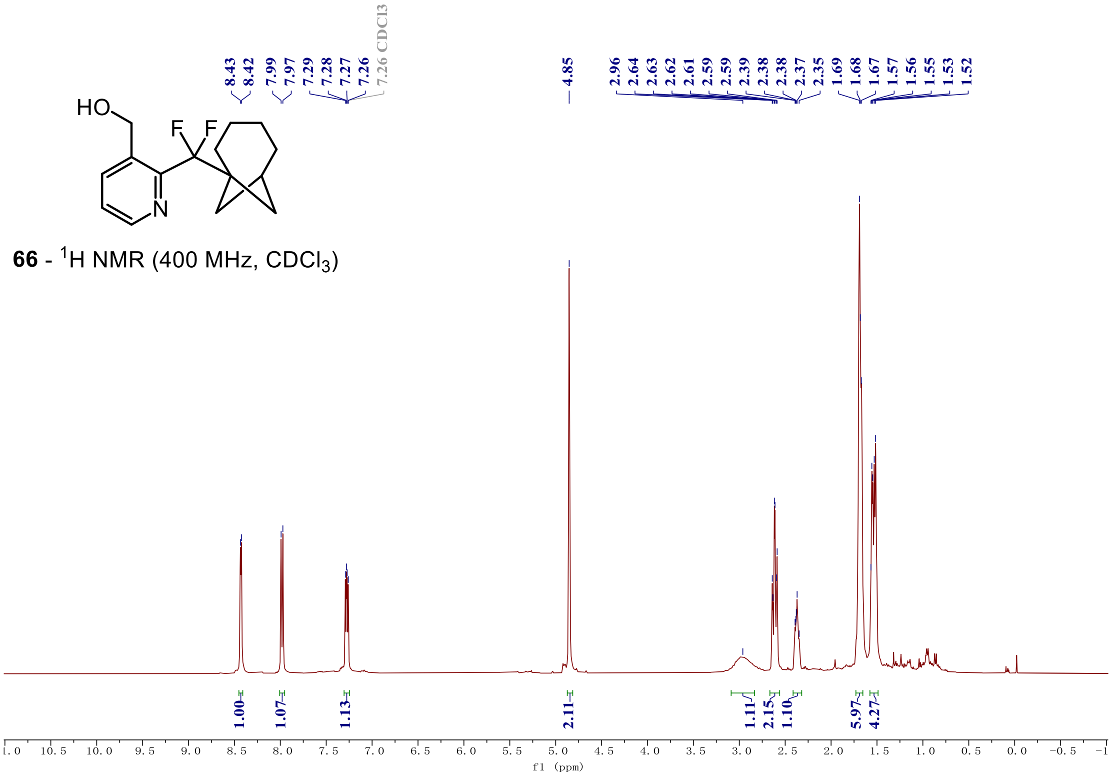

Supplementary Figure 216 ^{1}H NMR spectra of compound **66**

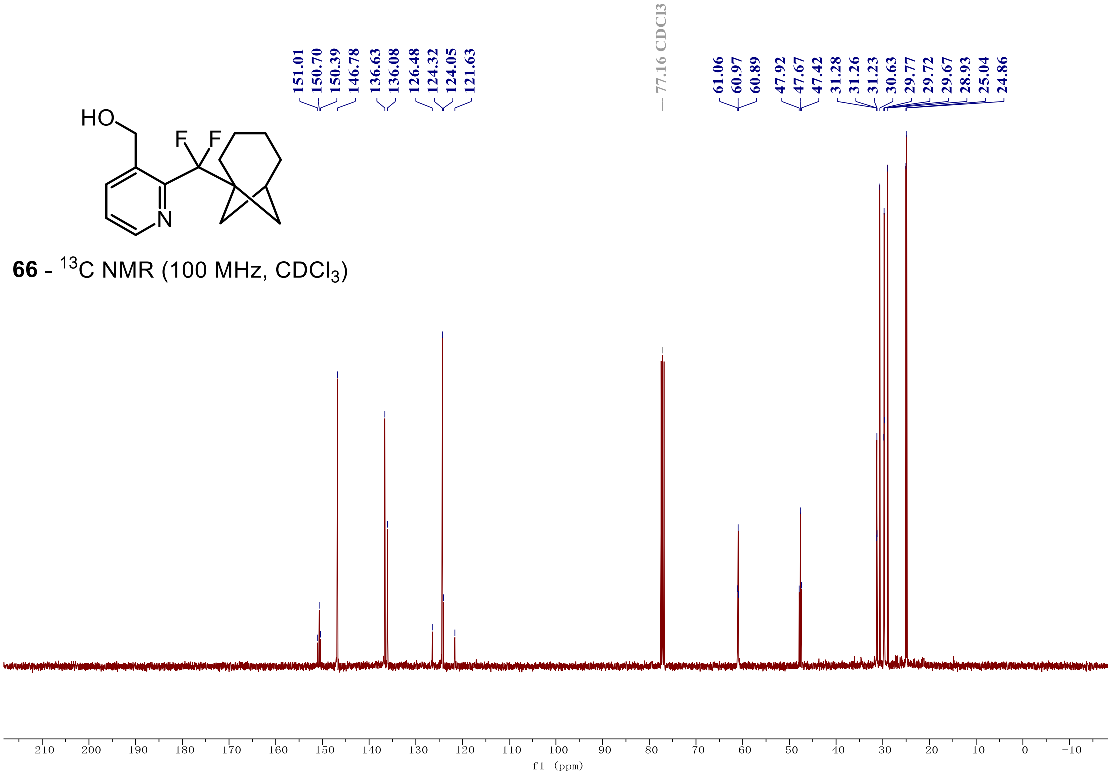

Supplementary Figure 217 ^{13}C NMR spectra of compound **66**

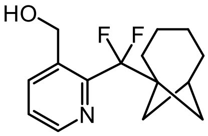

66 - ^{19}F NMR (376 MHz, $CDCl_3$)

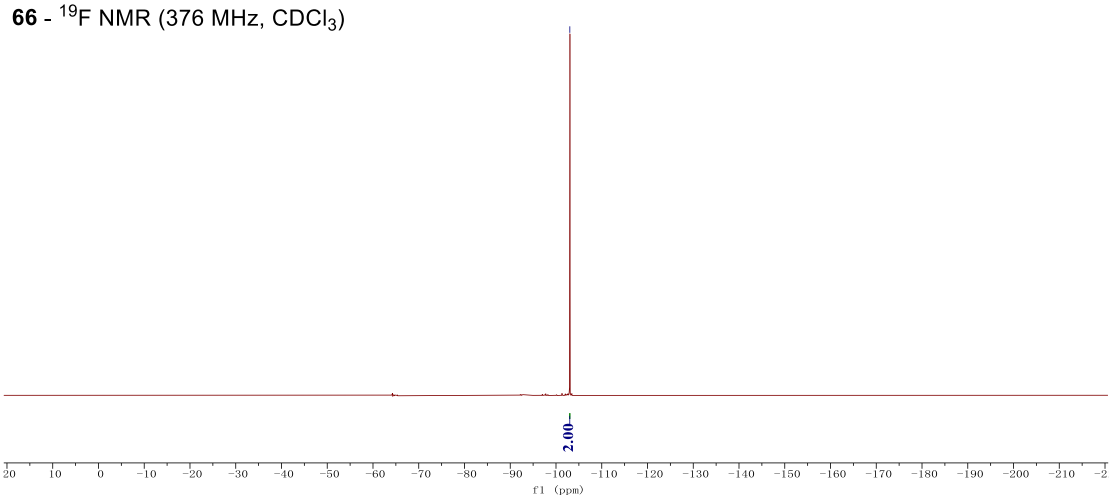

Supplementary Figure 218 ^{19}F NMR spectra of compound **66**

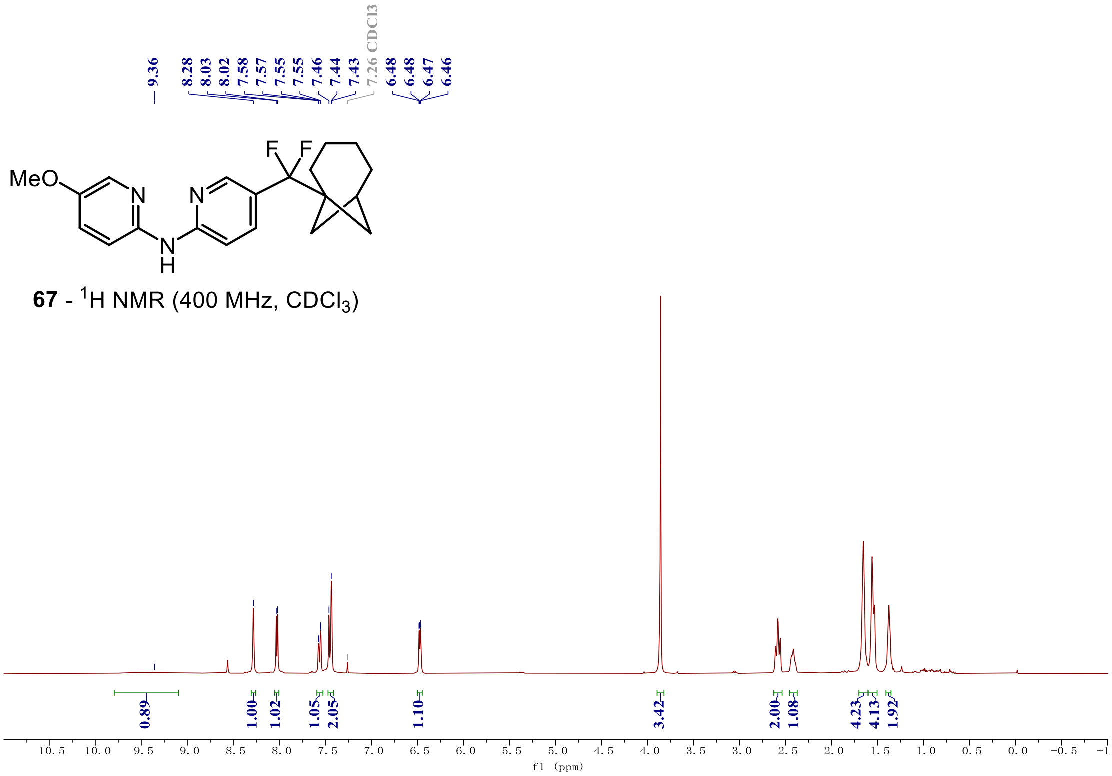

Supplementary Figure 219 ^{1}H NMR spectra of compound **67**

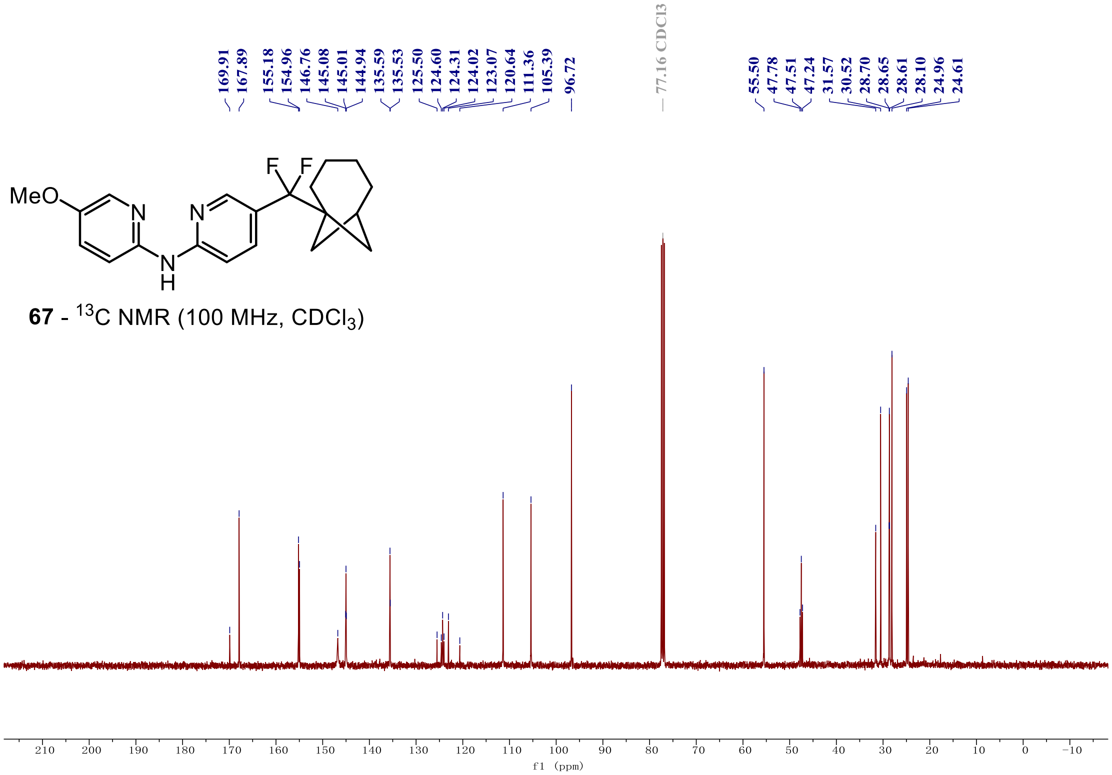

Supplementary Figure 220 ^{13}C NMR spectra of compound **67**

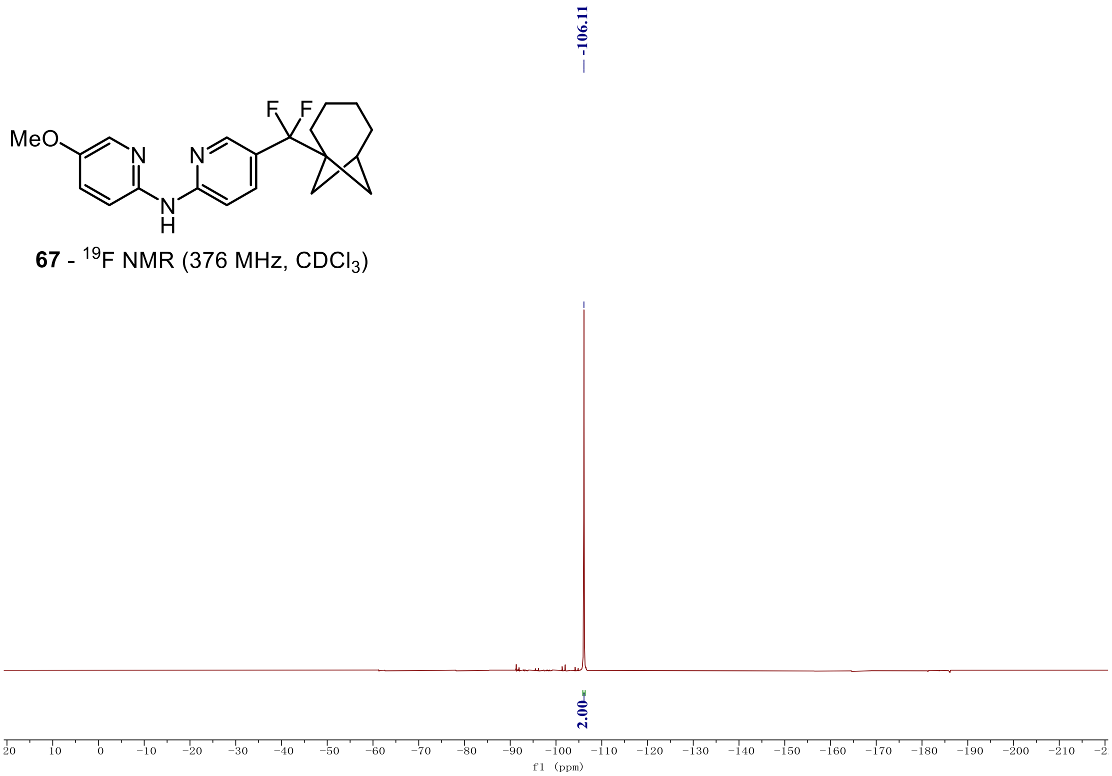

Supplementary Figure 221 ^{19}F NMR spectra of compound **67**

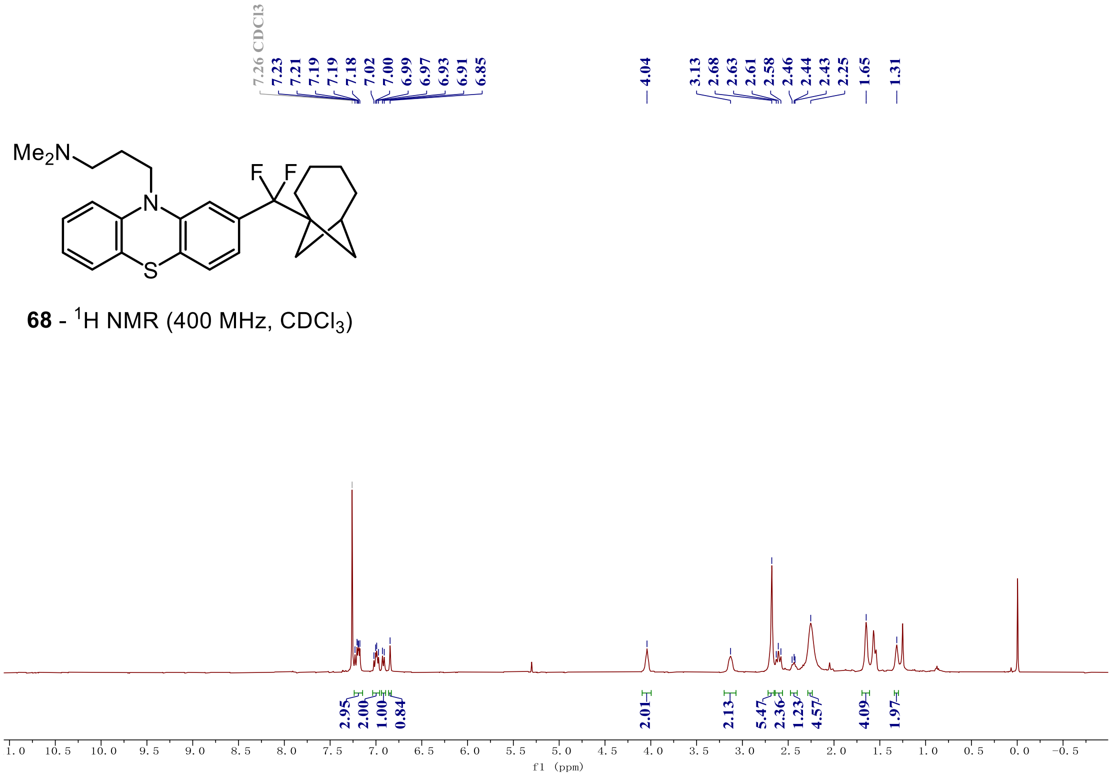

Supplementary Figure 222 ^{1}H NMR spectra of compound **68**

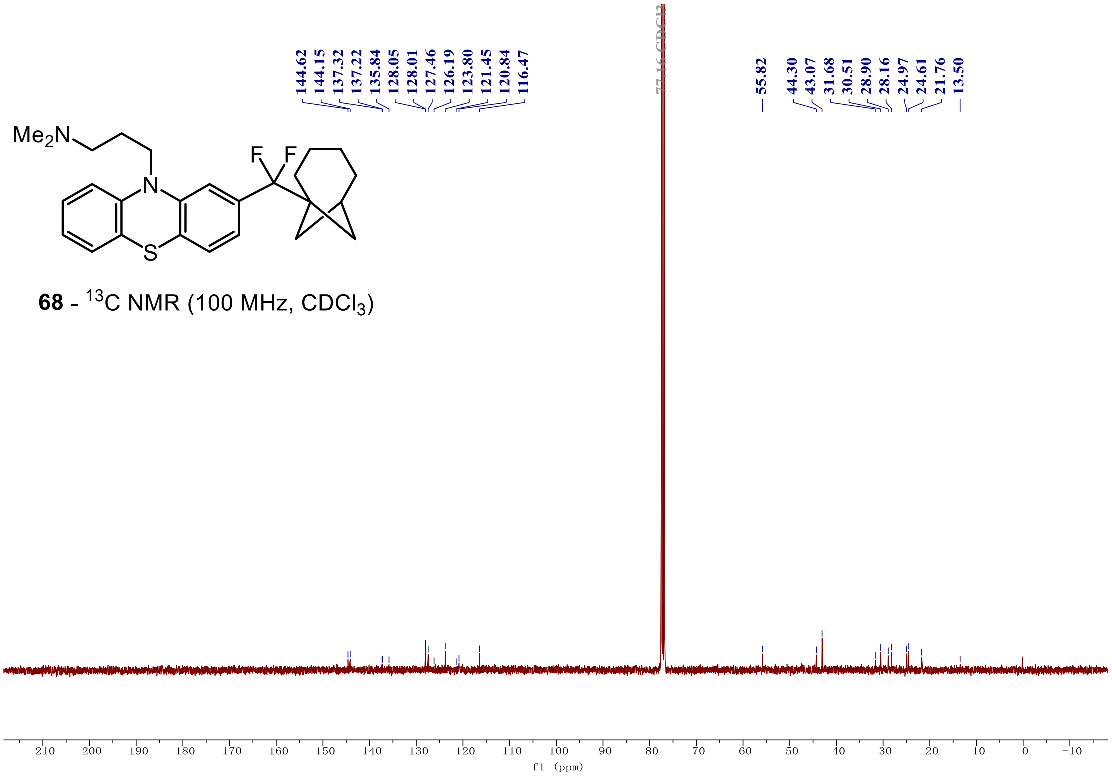

Supplementary Figure 223 ^{13}C NMR spectra of compound **68**

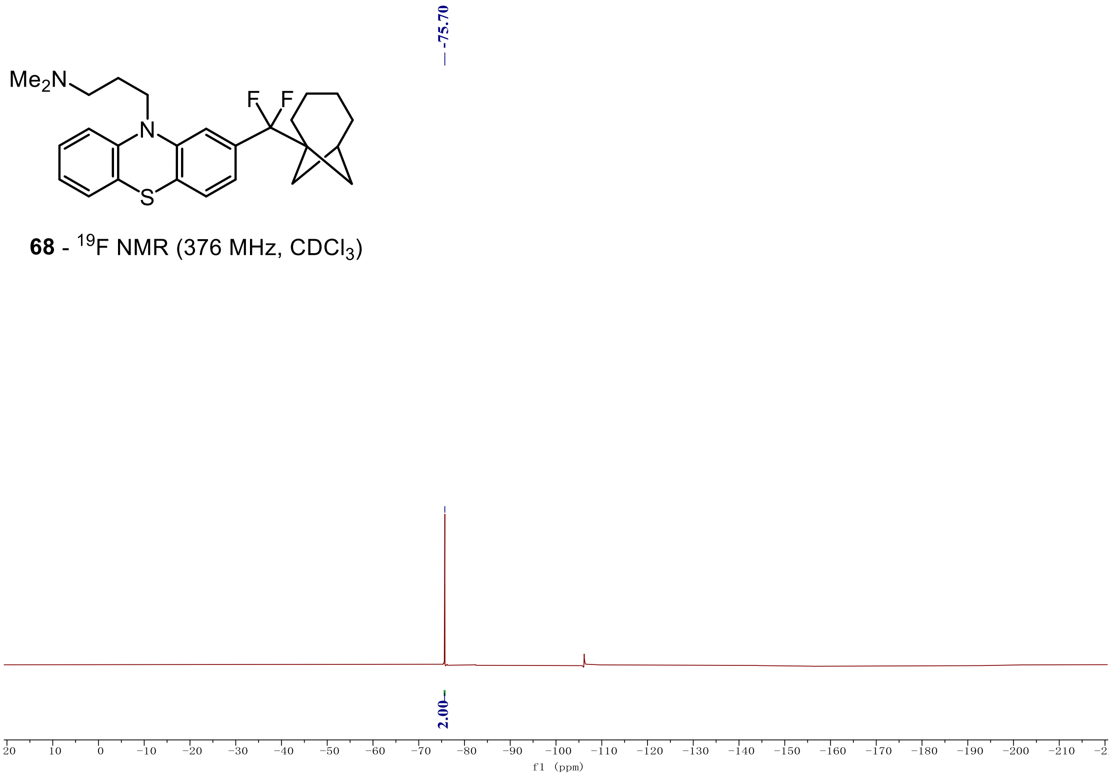

Supplementary Figure 224 ^{19}F NMR spectra of compound **68**

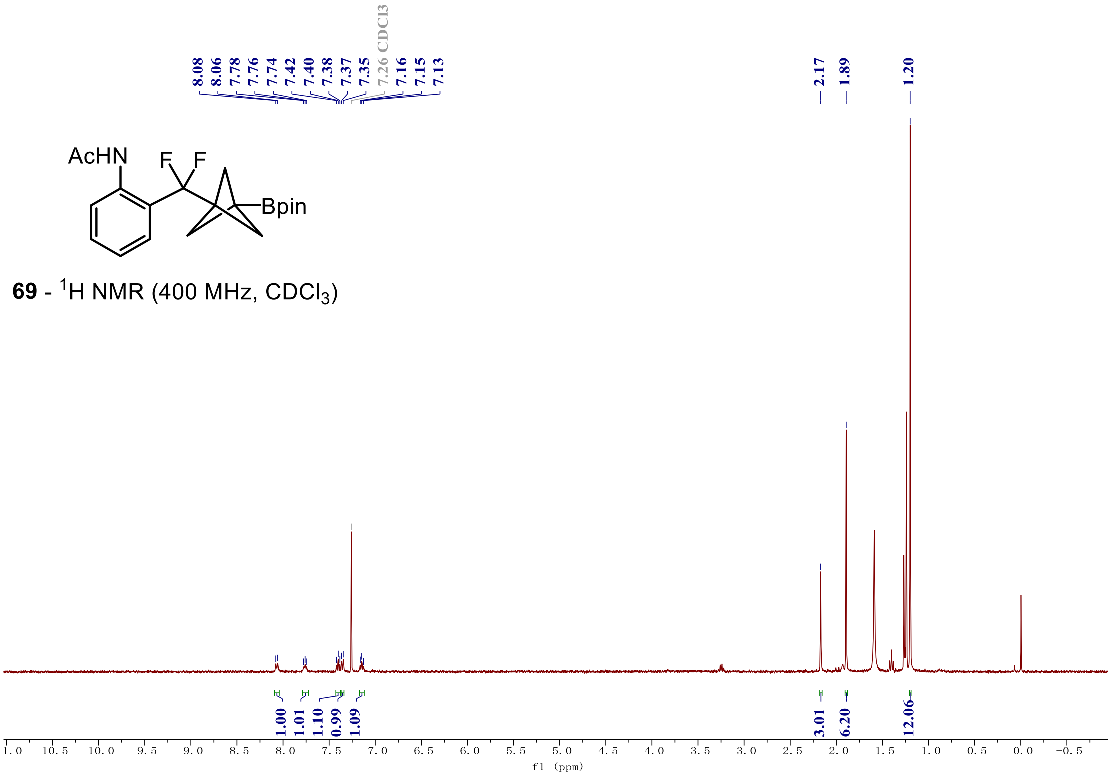

Supplementary Figure 225 ^{1}H NMR spectra of compound **69**

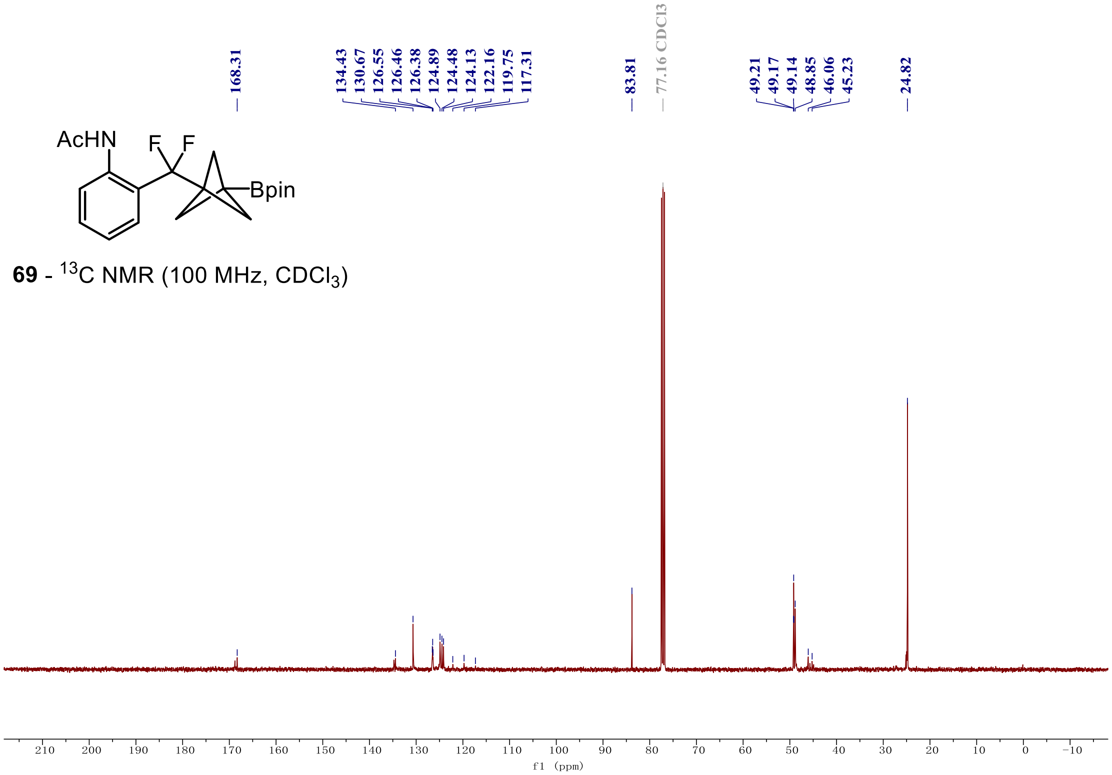

Supplementary Figure 226 ^{13}C NMR spectra of compound **69**

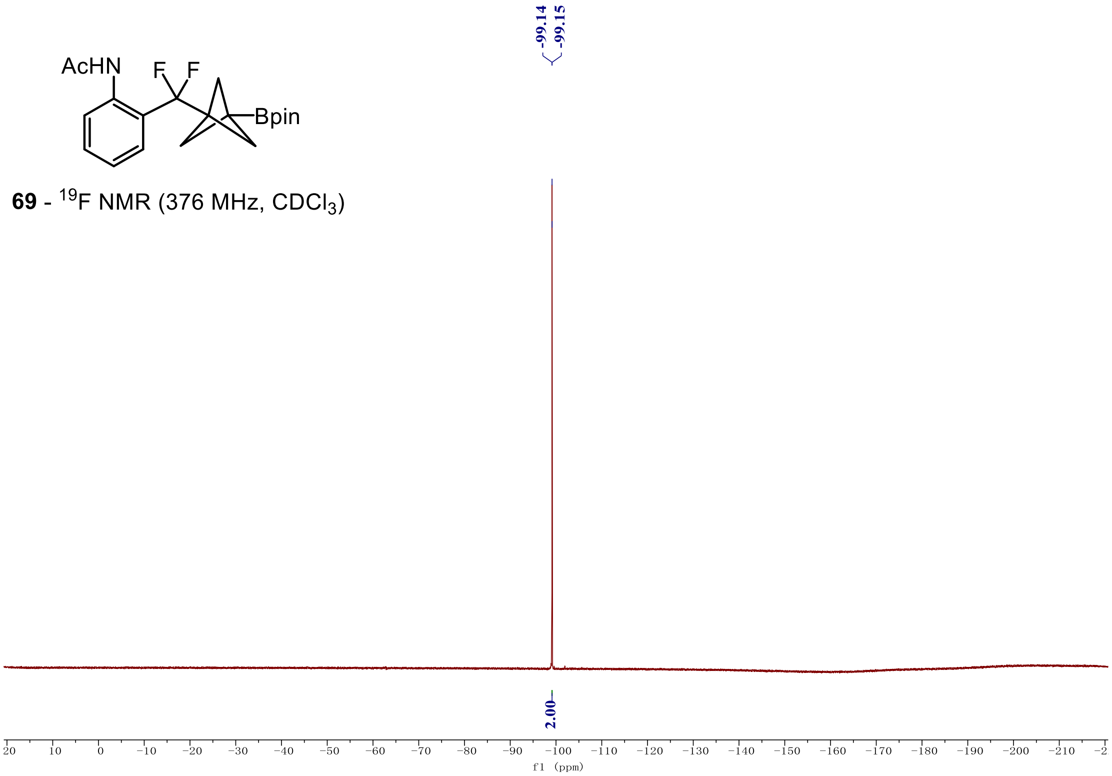

Supplementary Figure 227 ^{19}F NMR spectra of compound **69**

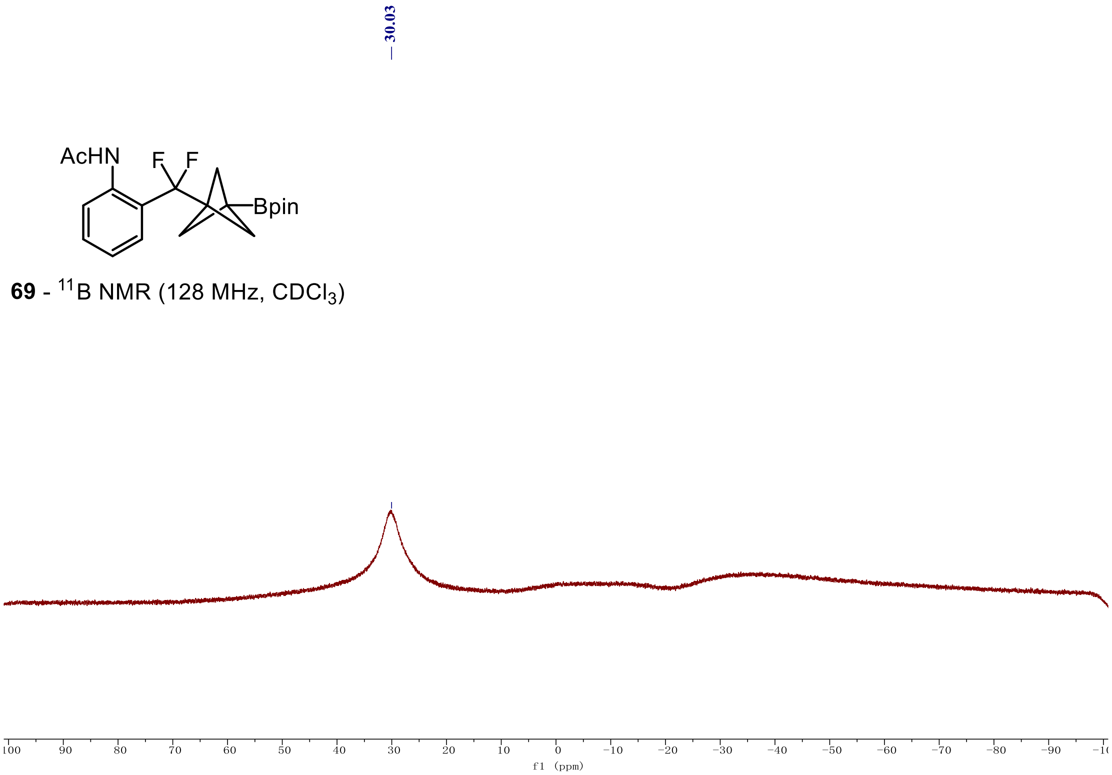

Supplementary Figure 228 ^{11}B NMR spectra of compound **69**

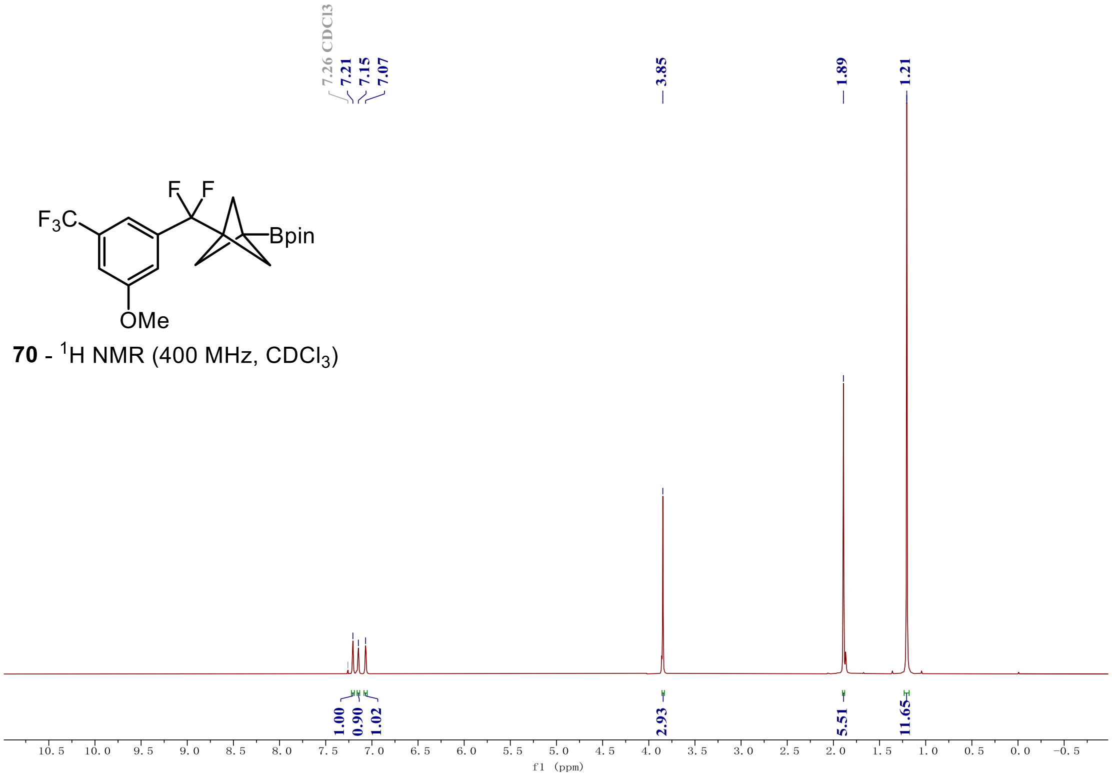

Supplementary Figure 229 ^{1}H NMR spectra of compound **70**

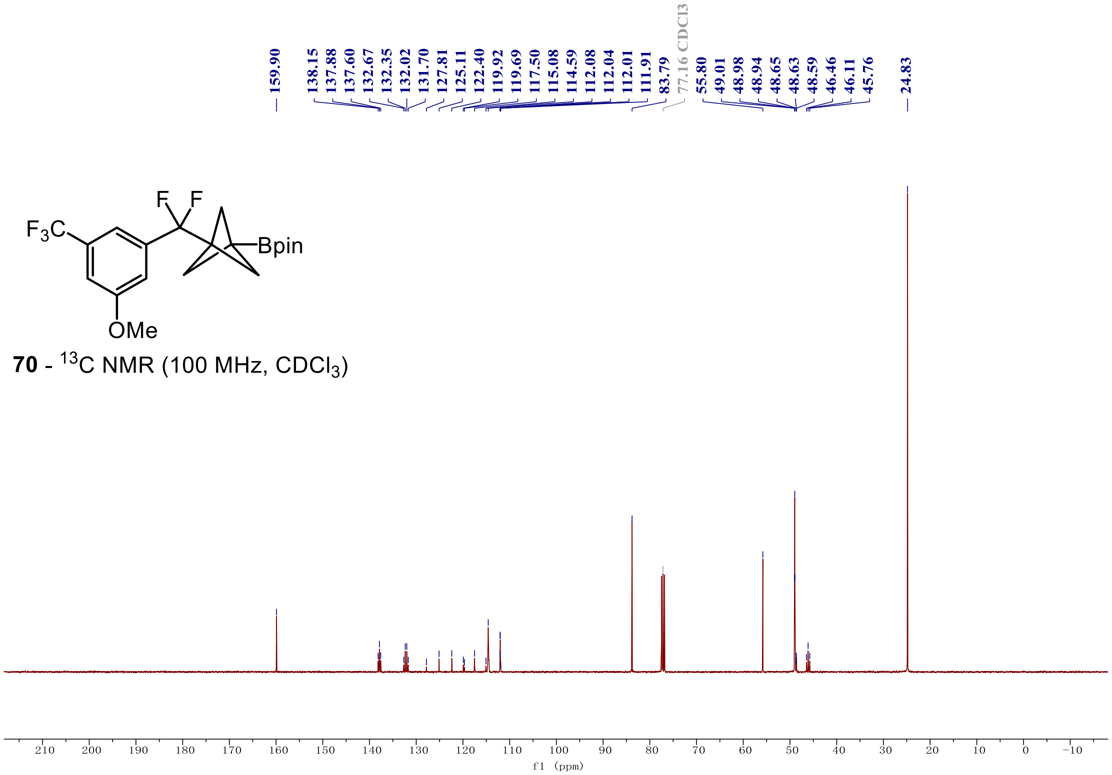

Supplementary Figure 230 ^{13}C NMR spectra of compound **70**

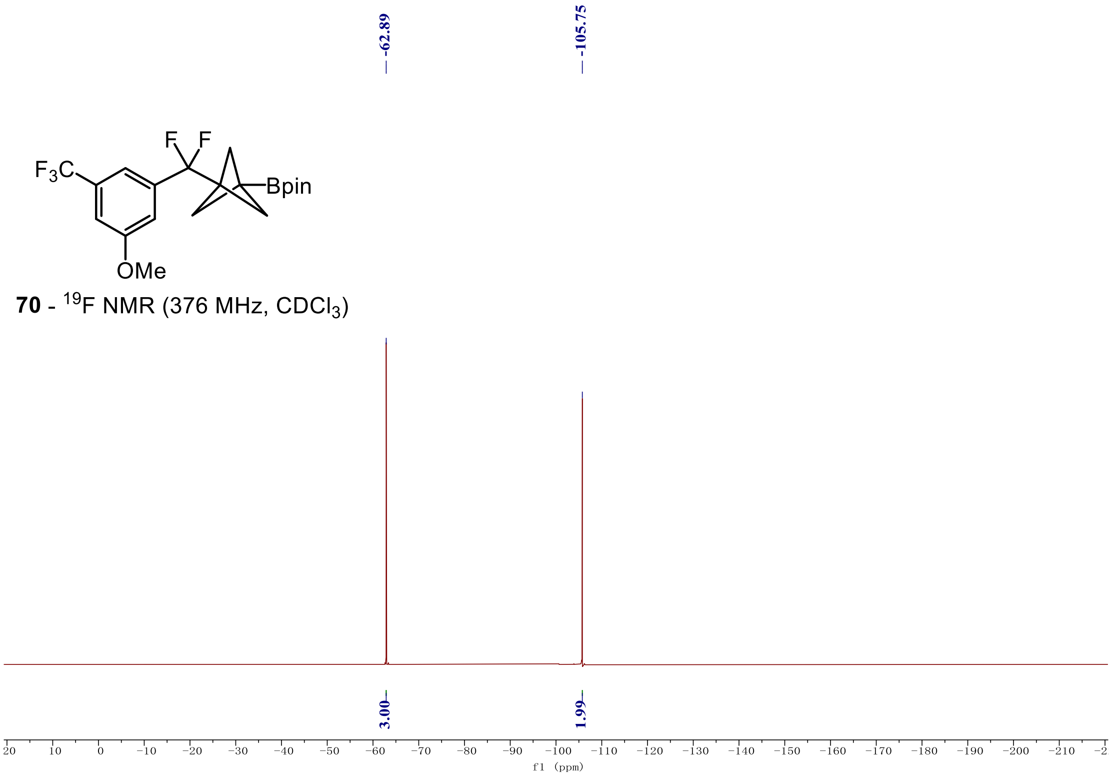

Supplementary Figure 231 ^{19}F NMR spectra of compound **70**

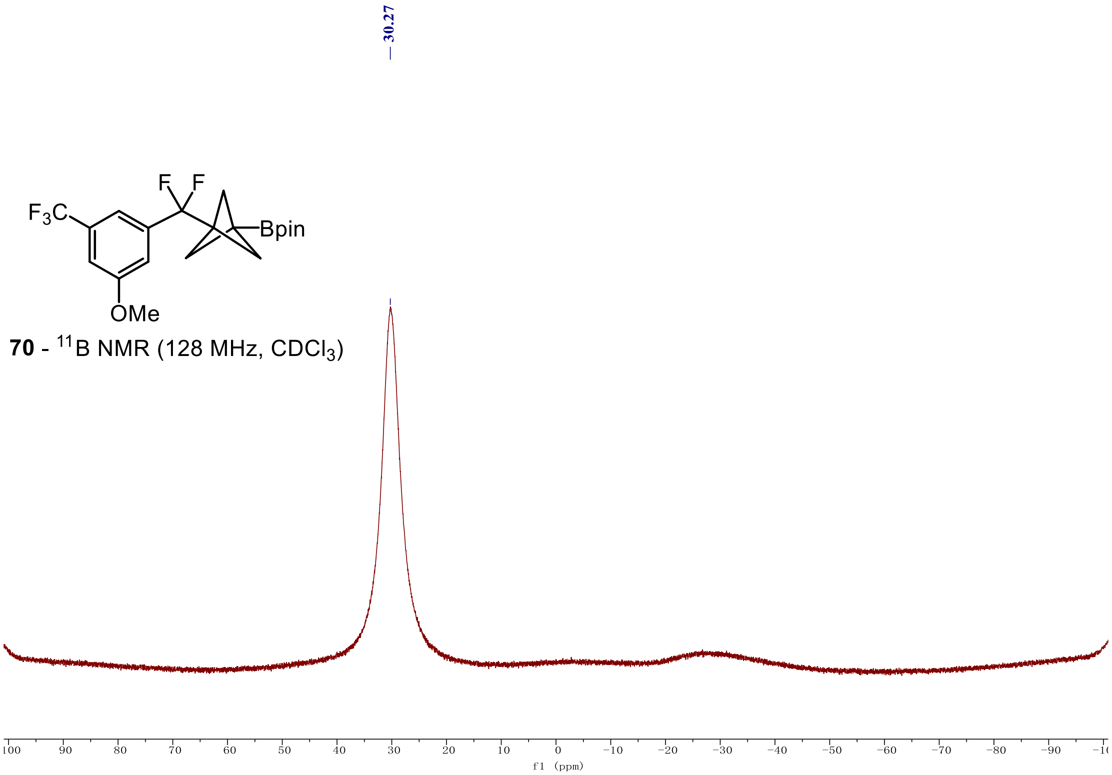

Supplementary Figure 232 ^{11}B NMR spectra of compound **70**

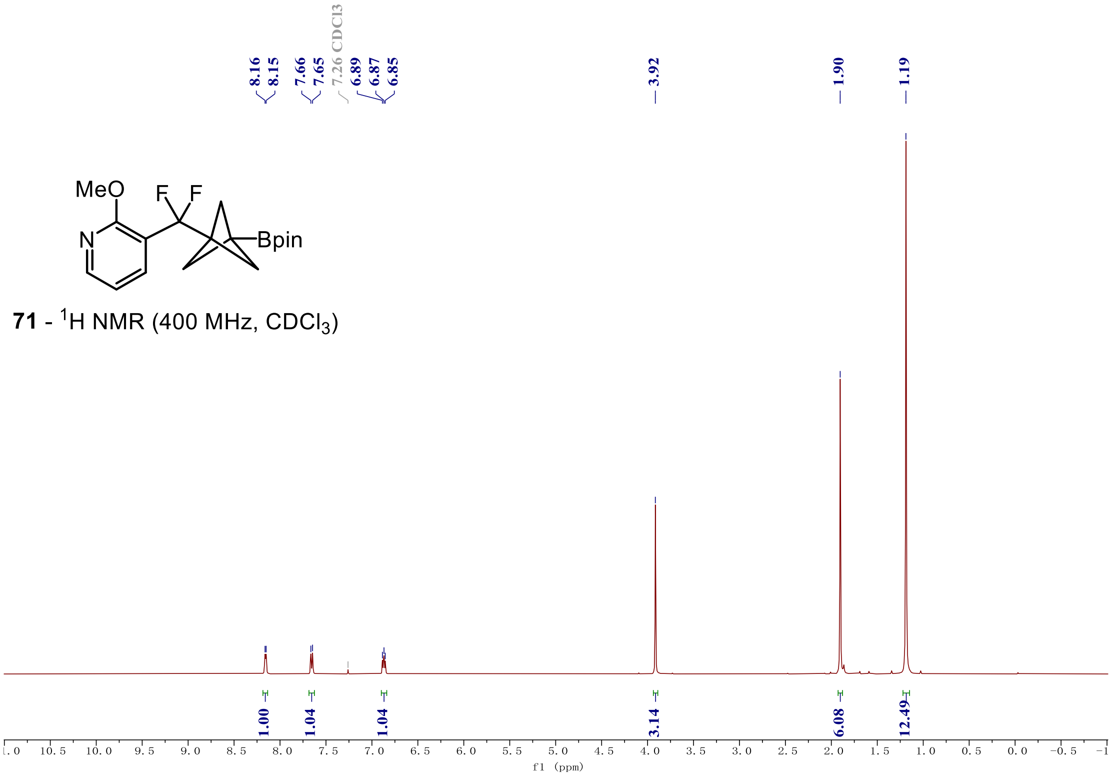

Supplementary Figure 233 ^{1}H NMR spectra of compound **71**

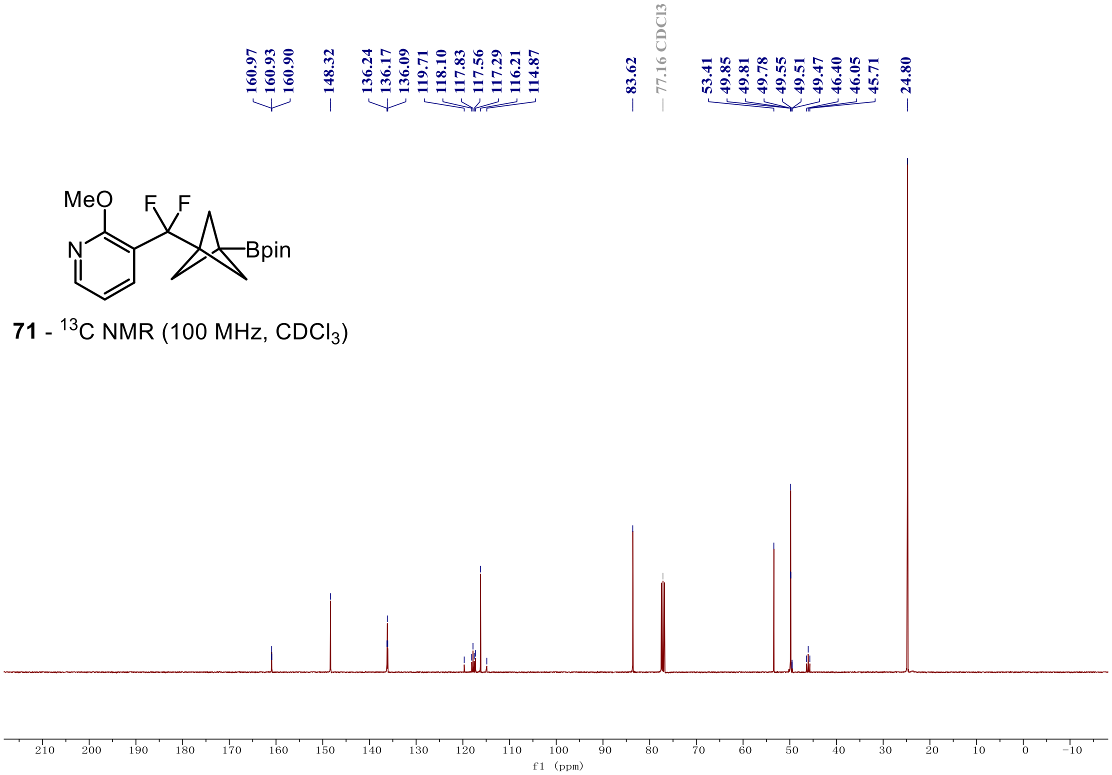

Supplementary Figure 234 ^{13}C NMR spectra of compound **71**

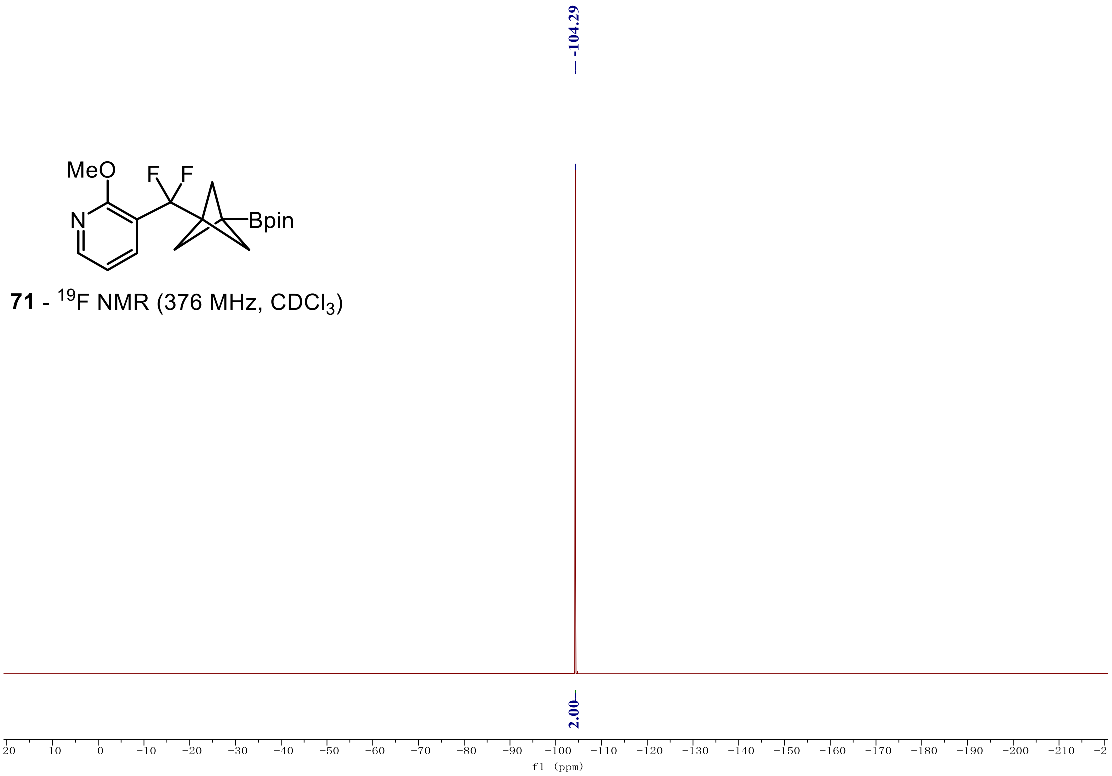

Supplementary Figure 235 ^{19}F NMR spectra of compound **71**

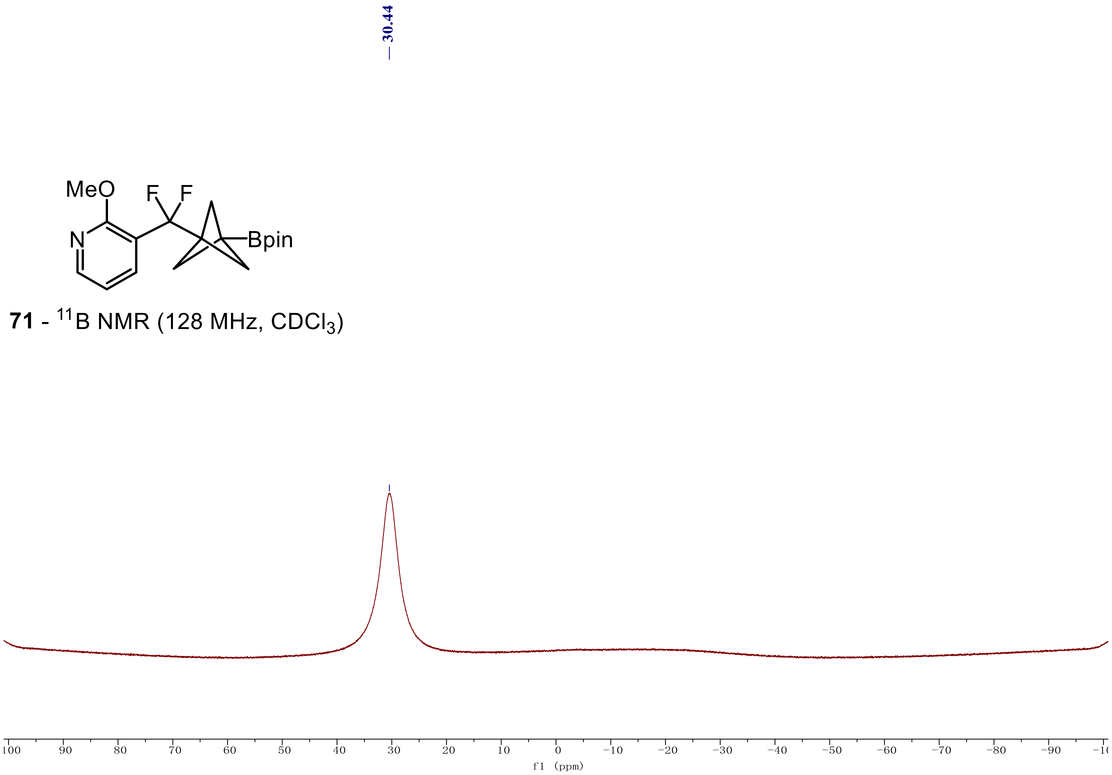

Supplementary Figure 236 ^{11}B NMR spectra of compound **71**

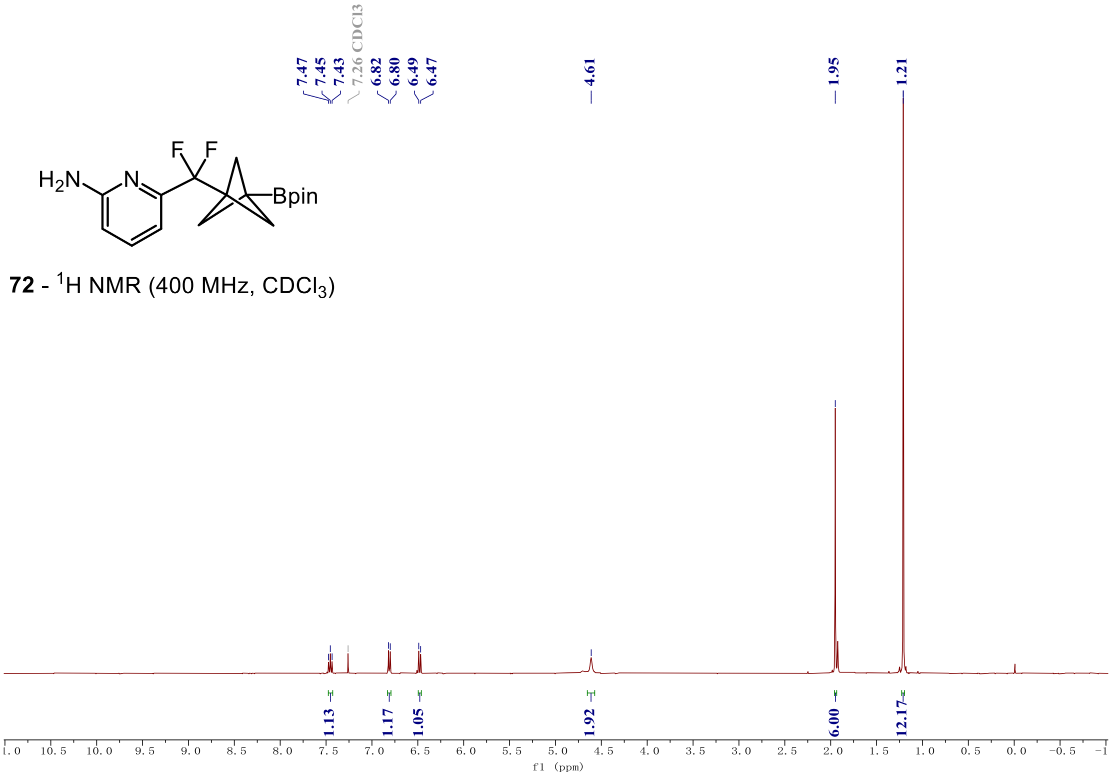

Supplementary Figure 237 ^{1}H NMR spectra of compound **72**

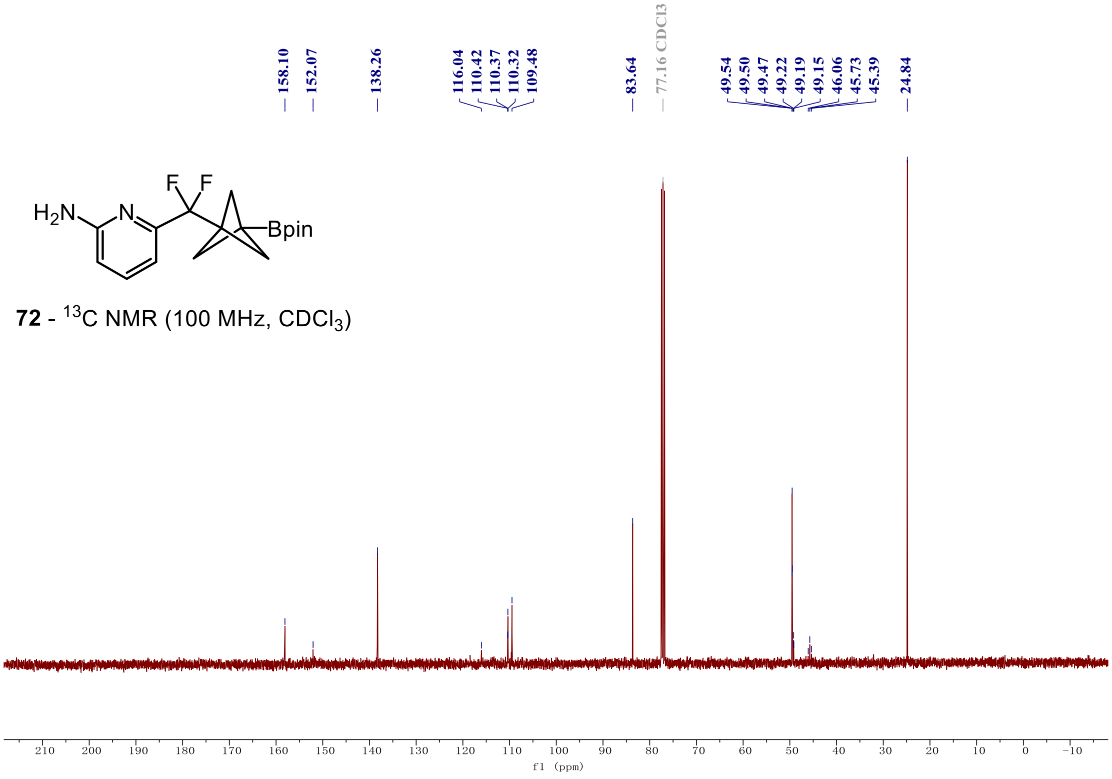

Supplementary Figure 238 ^{13}C NMR spectra of compound **72**

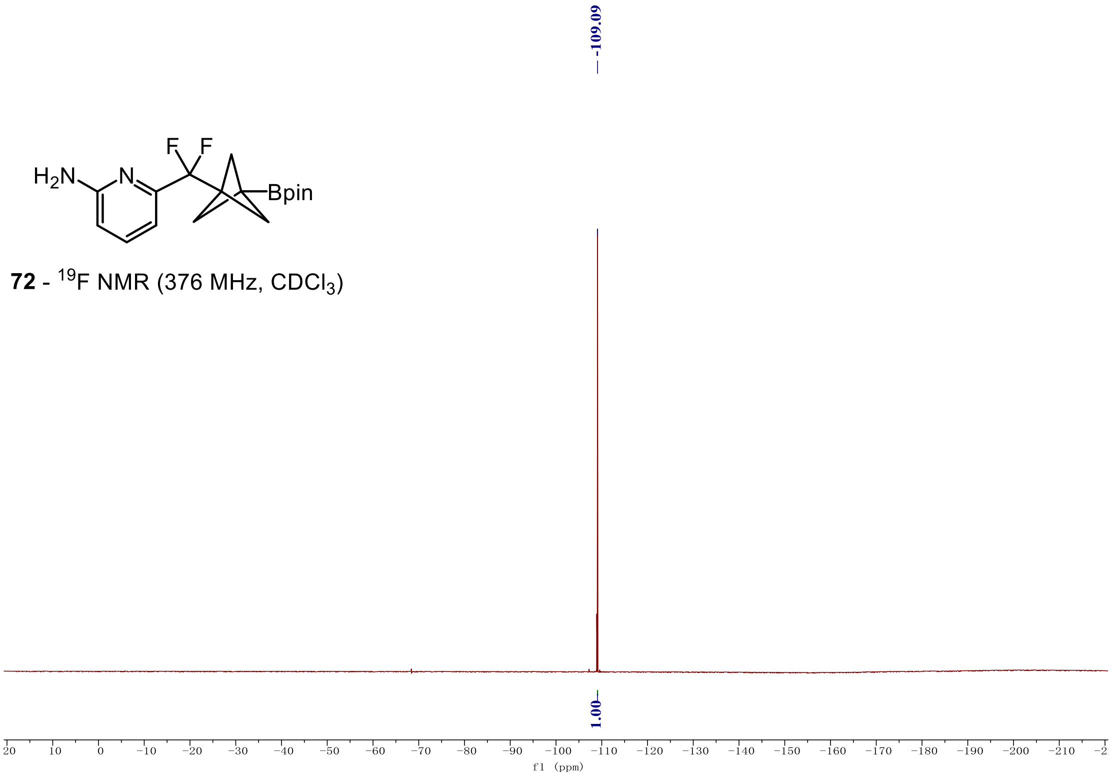

Supplementary Figure 239 ^{19}F NMR spectra of compound **72**

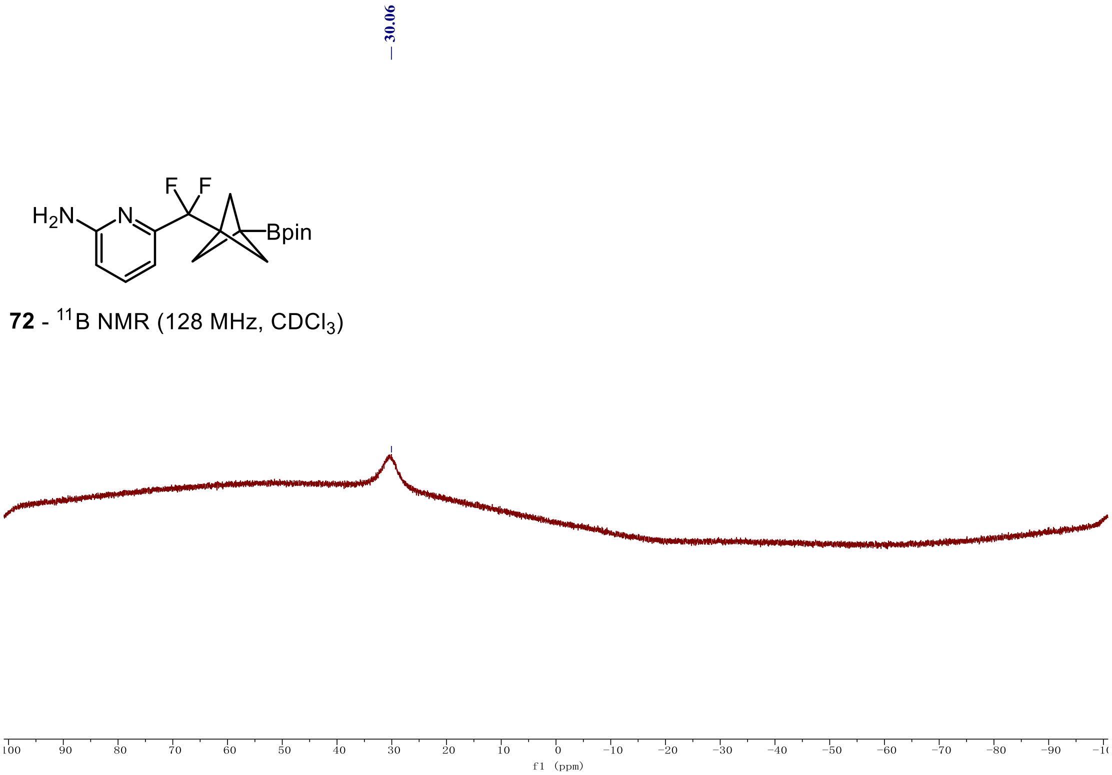

Supplementary Figure 240 ^{11}B NMR spectra of compound **72**

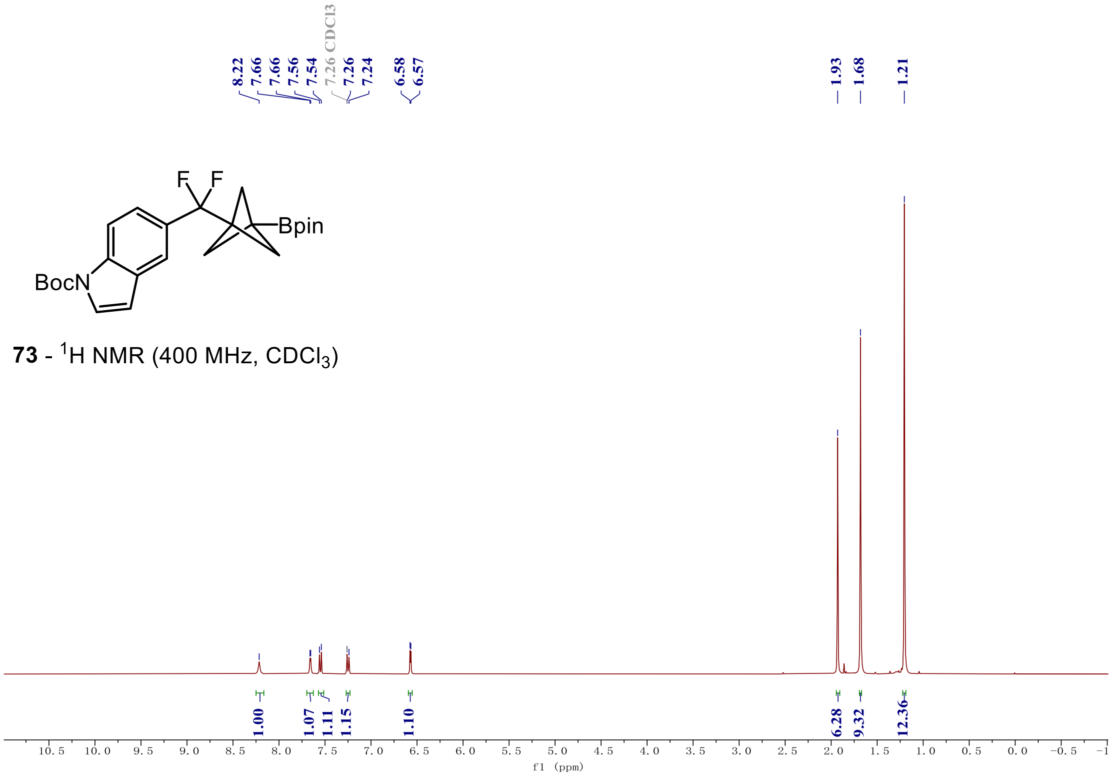

Supplementary Figure 241 ^{1}H NMR spectra of compound **73**

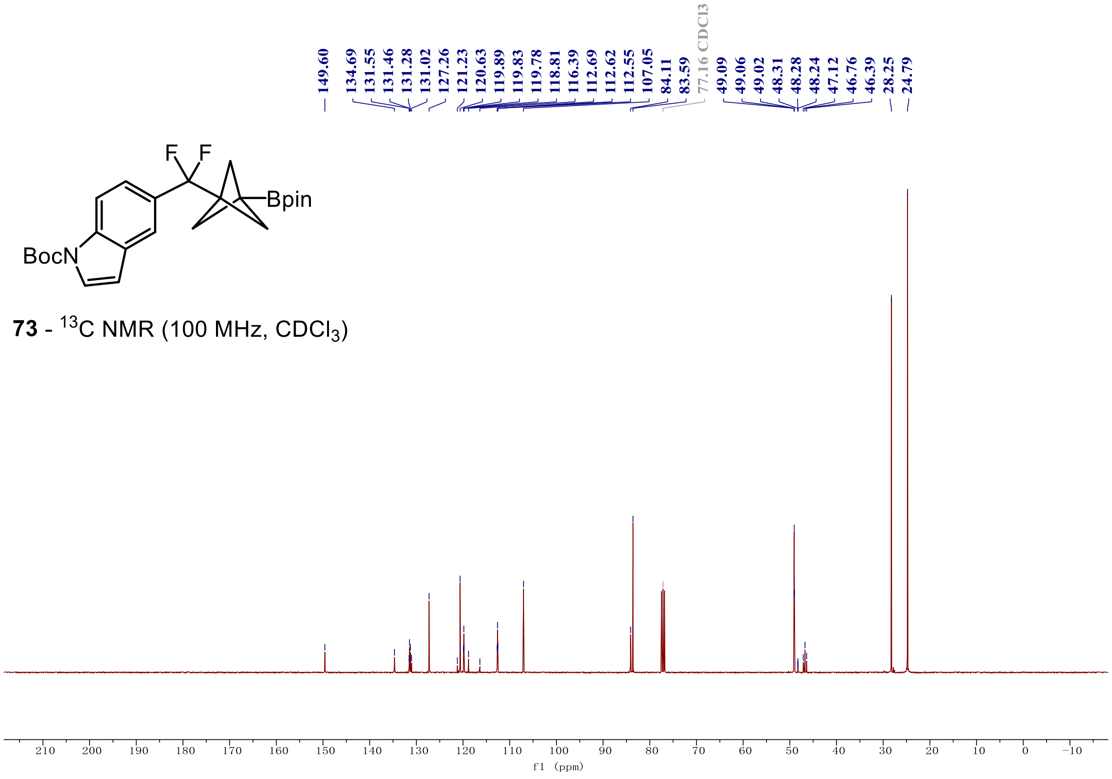

Supplementary Figure 242 ^{13}C NMR spectra of compound **73**

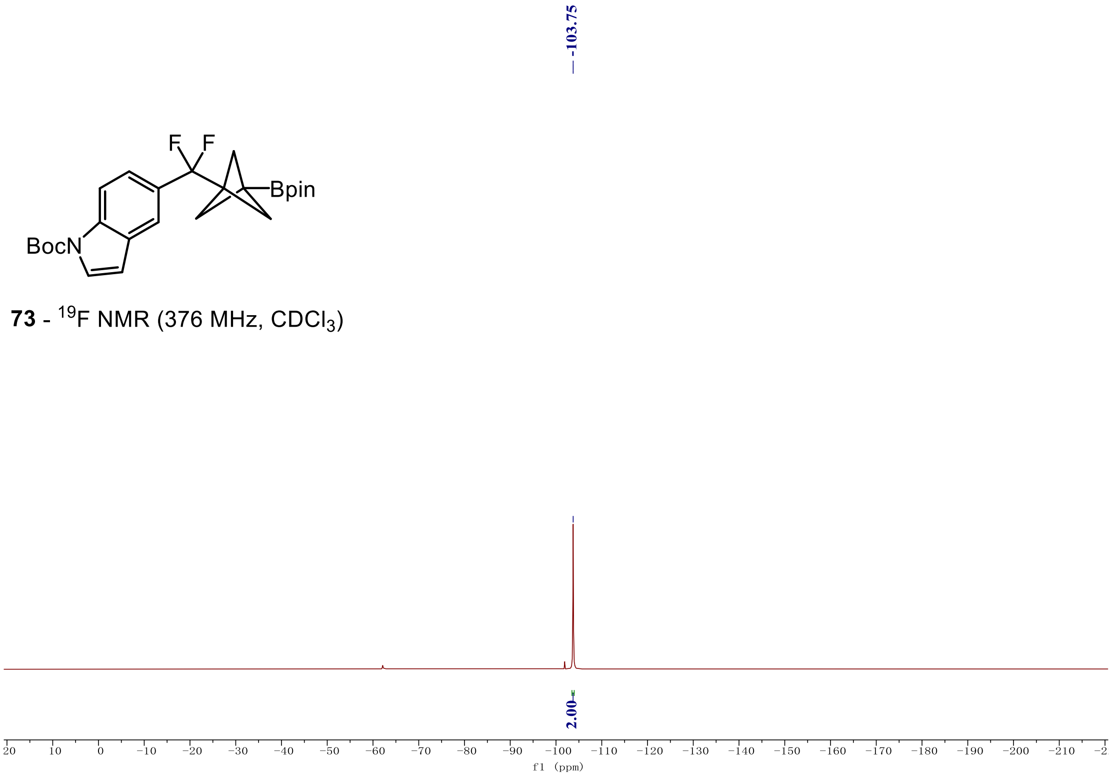

Supplementary Figure 243 ^{19}F NMR spectra of compound **73**

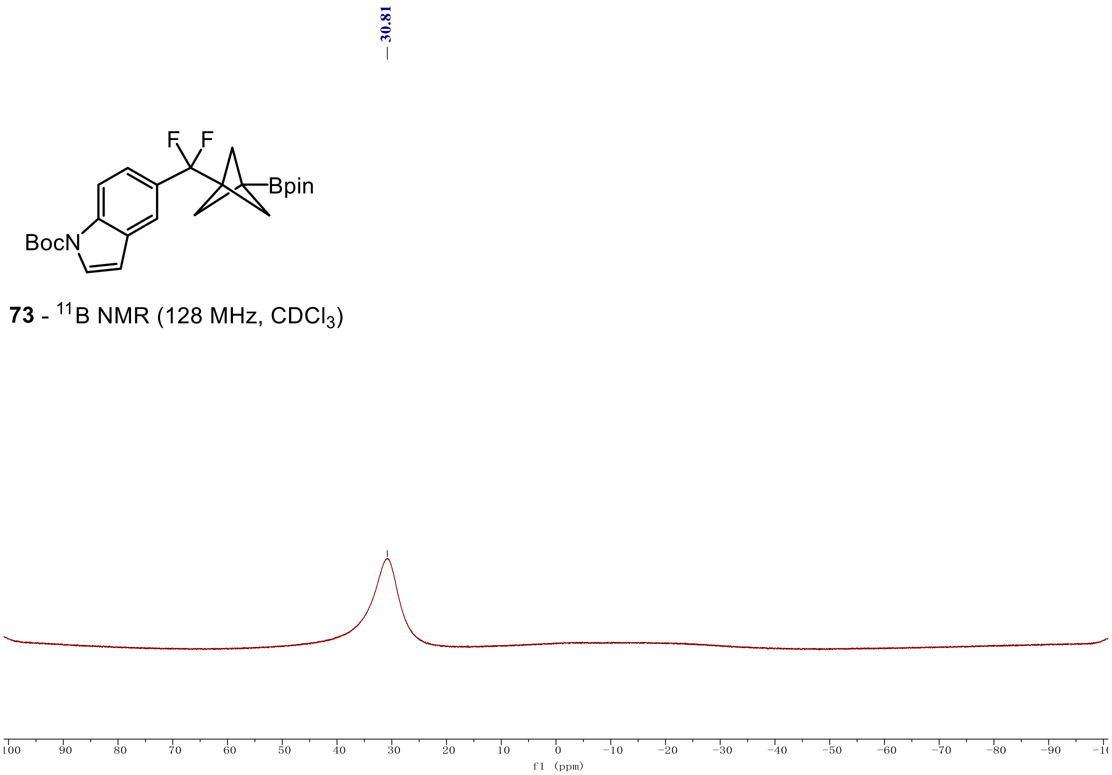

Supplementary Figure 244 ^{11}B NMR spectra of compound **73**

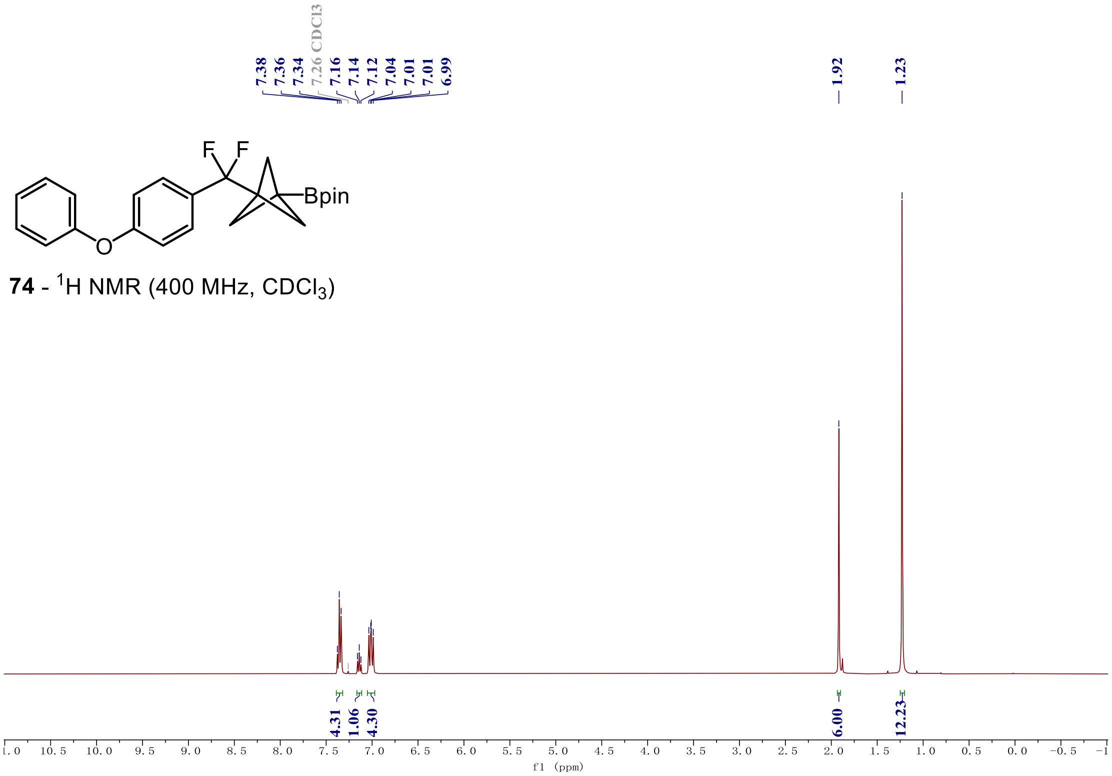

Supplementary Figure 245 ^{1}H NMR spectra of compound **74**

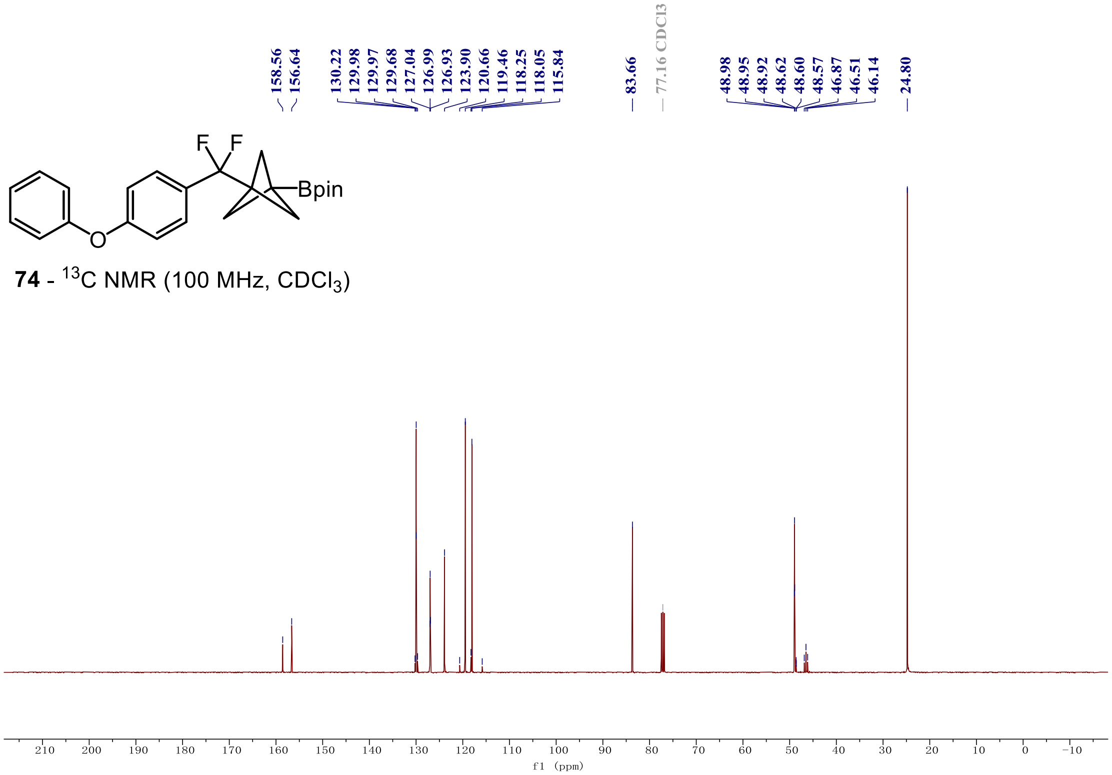

Supplementary Figure 246 ^{13}C NMR spectra of compound **74**

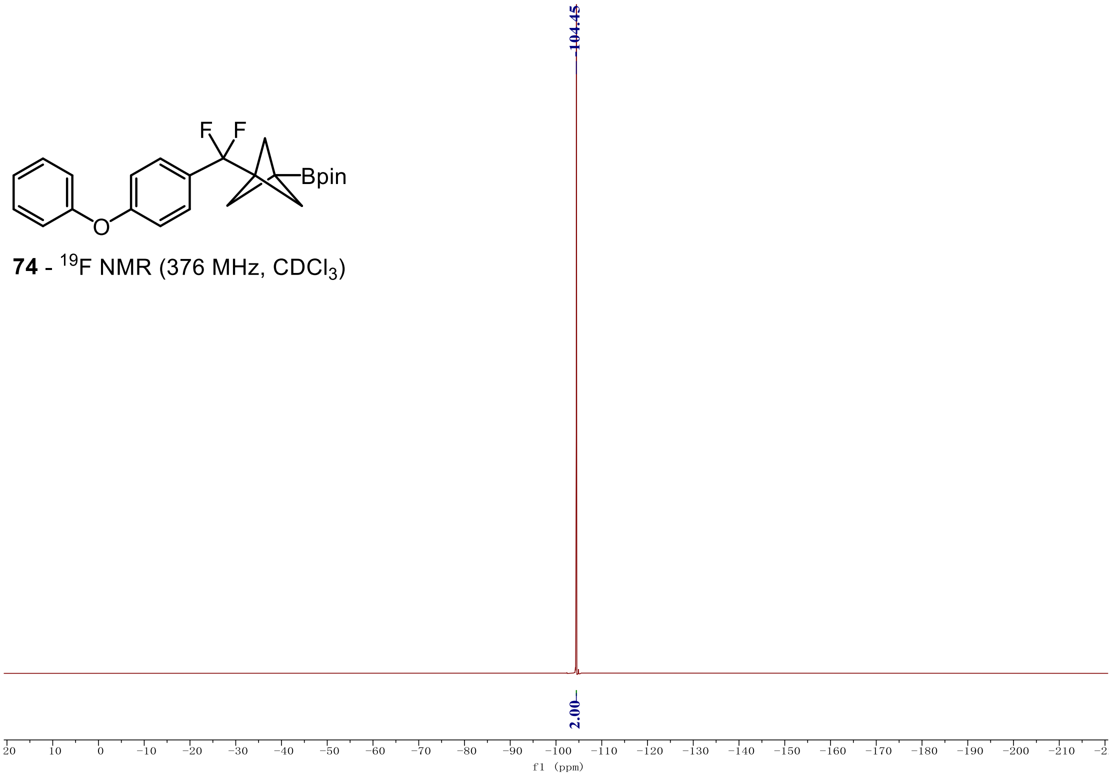

Supplementary Figure 247 ^{19}F NMR spectra of compound **74**

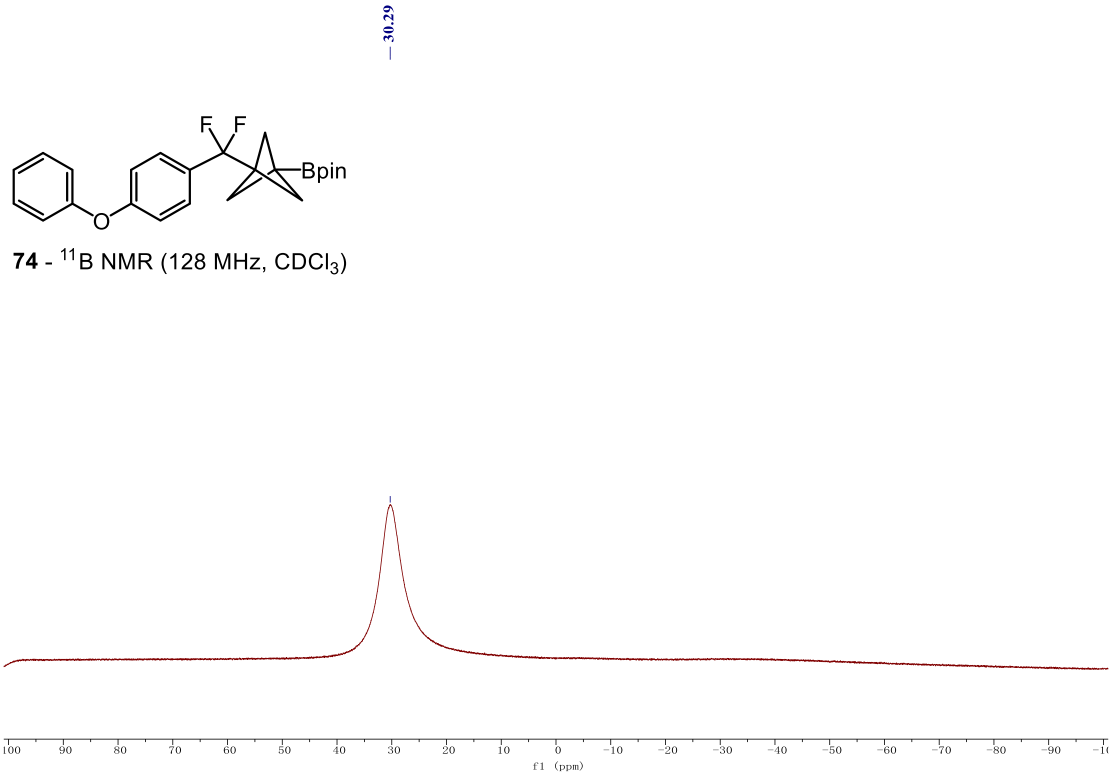

Supplementary Figure 248 ^{11}B NMR spectra of compound **74**

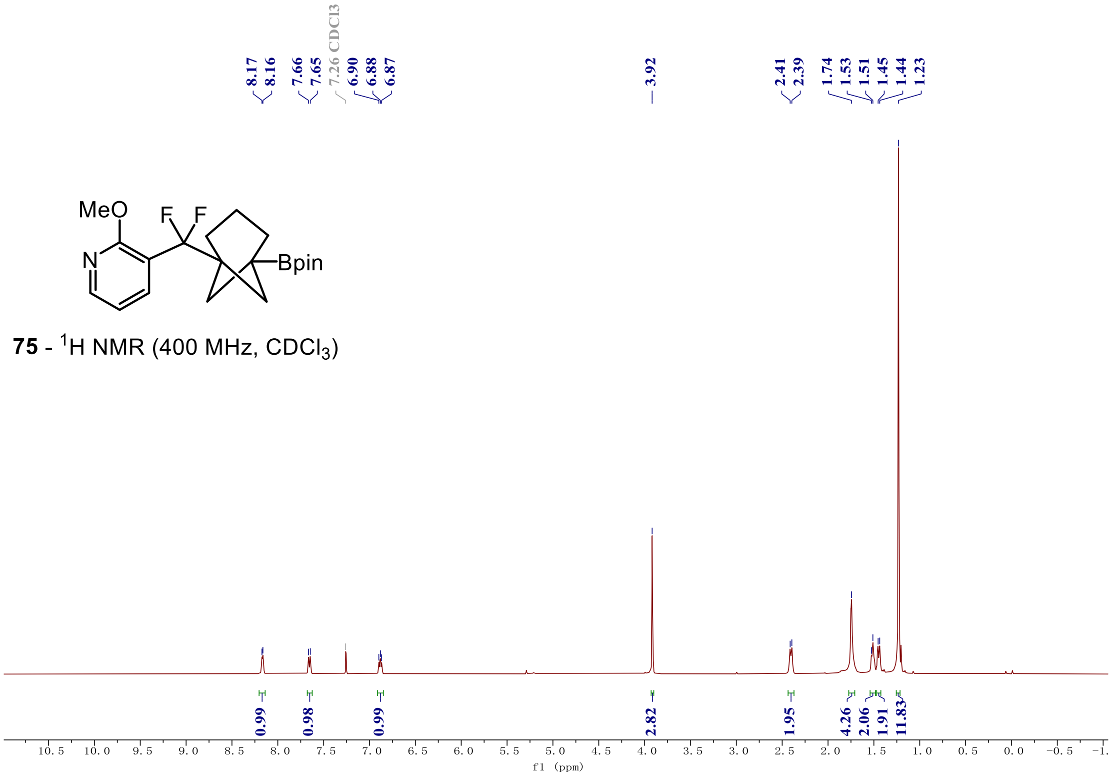

Supplementary Figure 249 ^{1}H NMR spectra of compound **75**

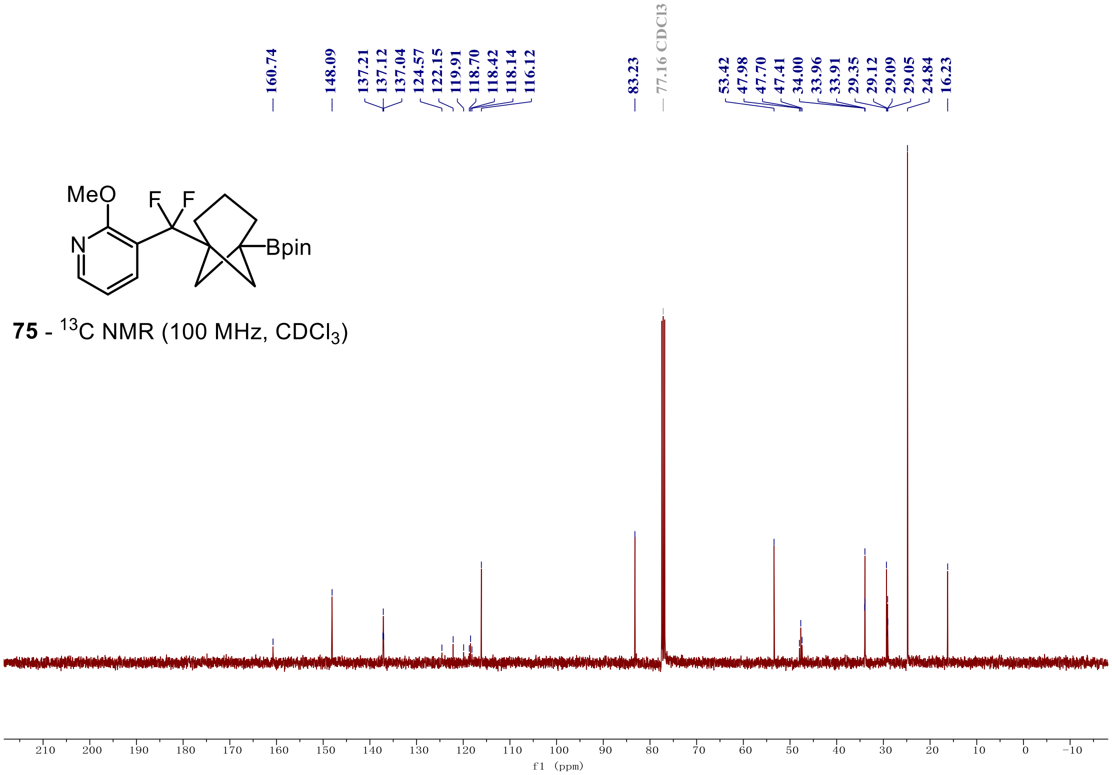

Supplementary Figure 250 ^{13}C NMR spectra of compound **75**

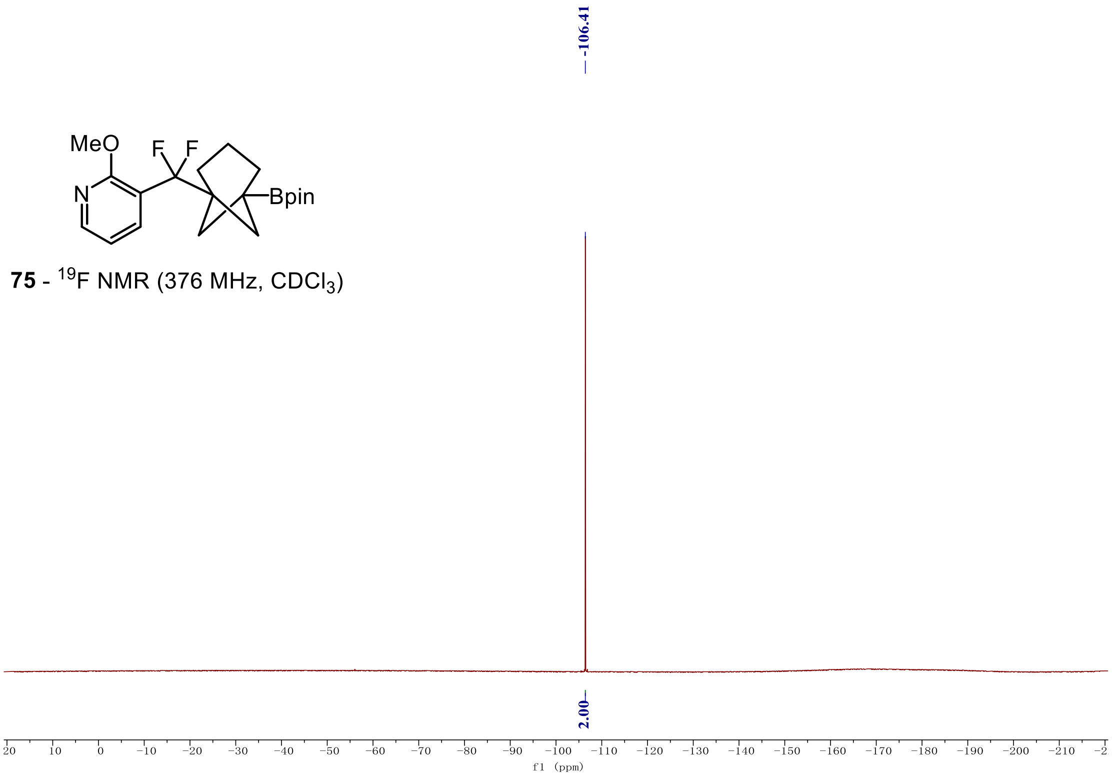

Supplementary Figure 251 ^{19}F NMR spectra of compound **75**

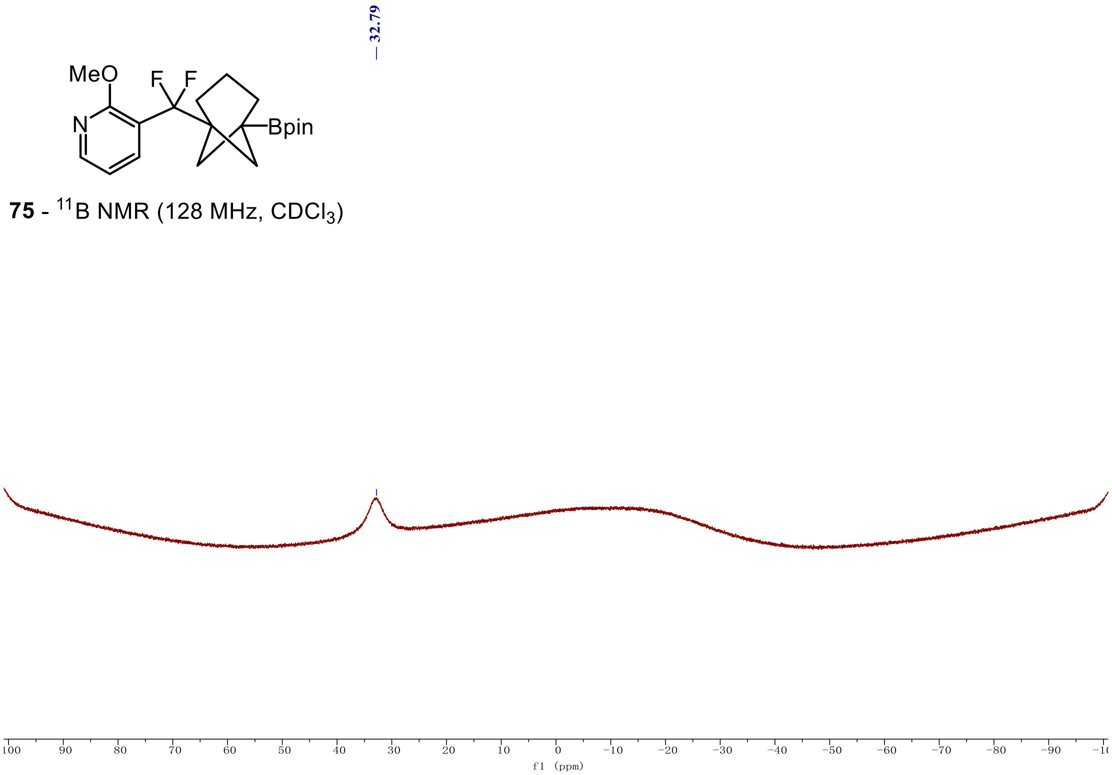

Supplementary Figure 252 ^{11}B NMR spectra of compound **75**

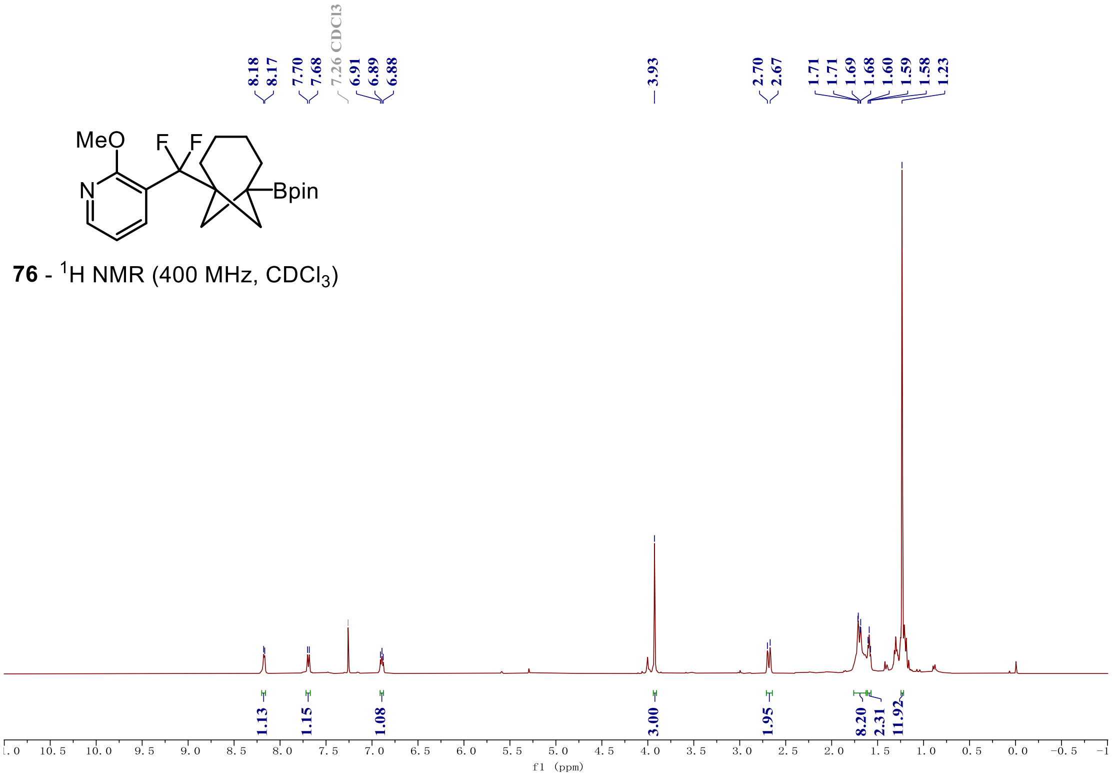

Supplementary Figure 253 ^{1}H NMR spectra of compound **76**

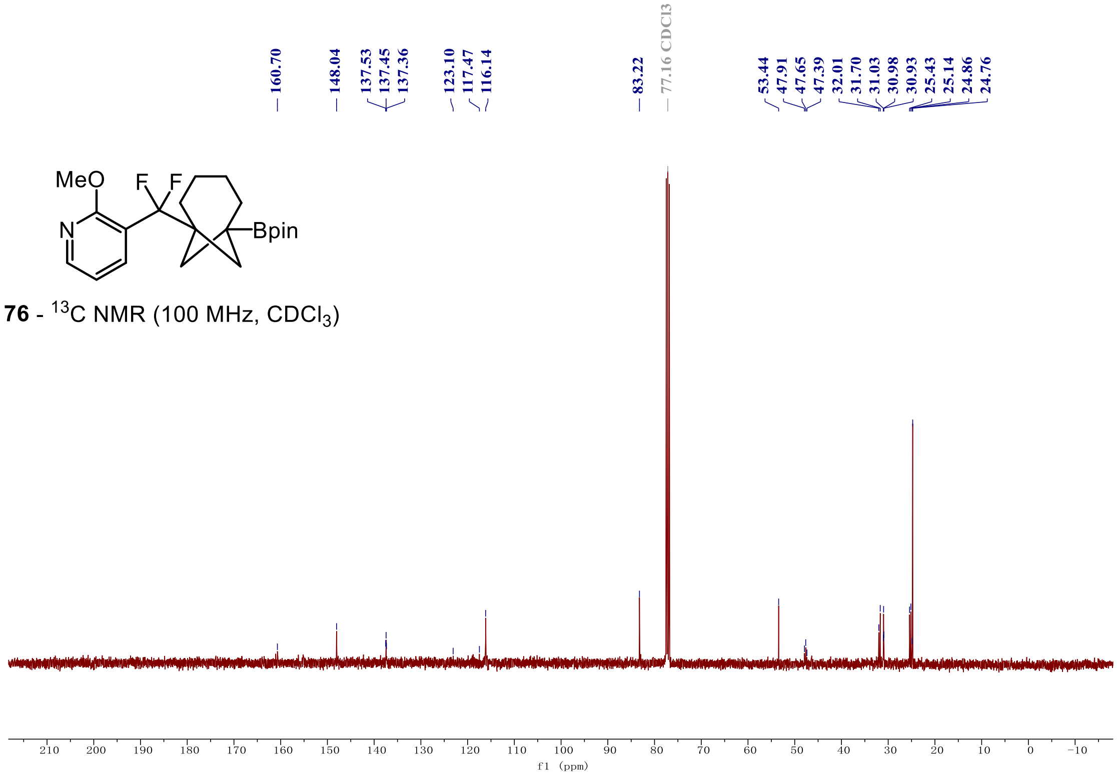

Supplementary Figure 254 ^{13}C NMR spectra of compound **76**

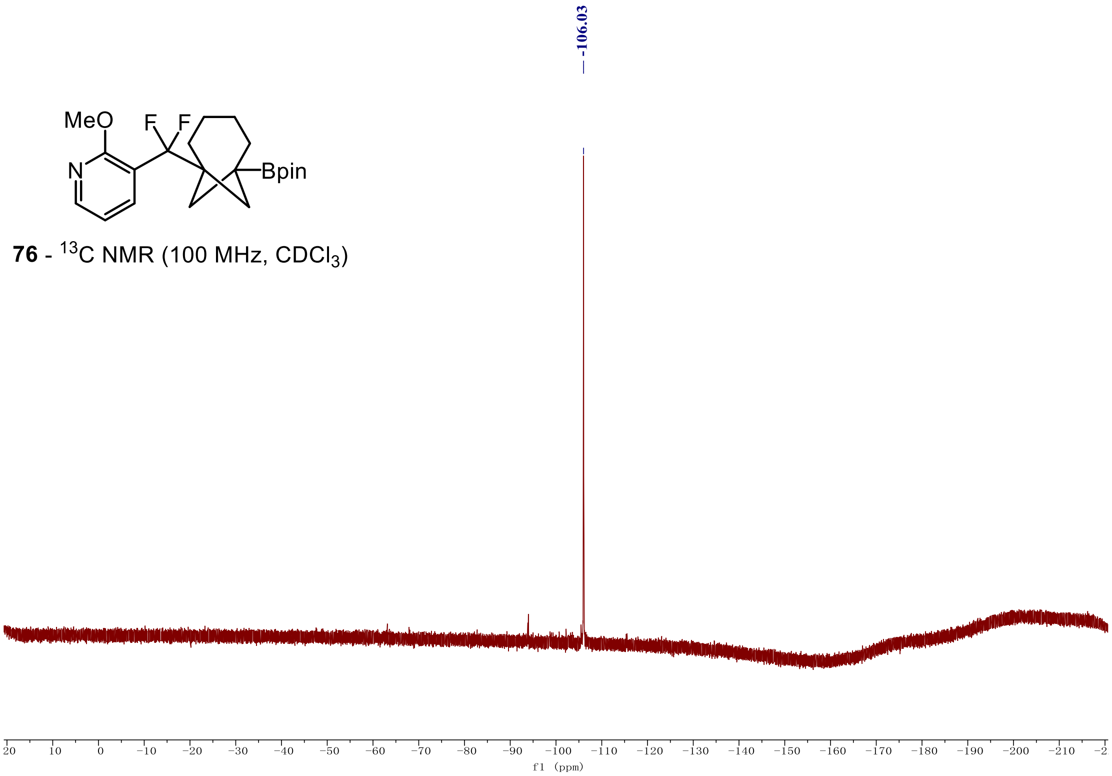

Supplementary Figure 255 ^{19}F NMR spectra of compound **76**

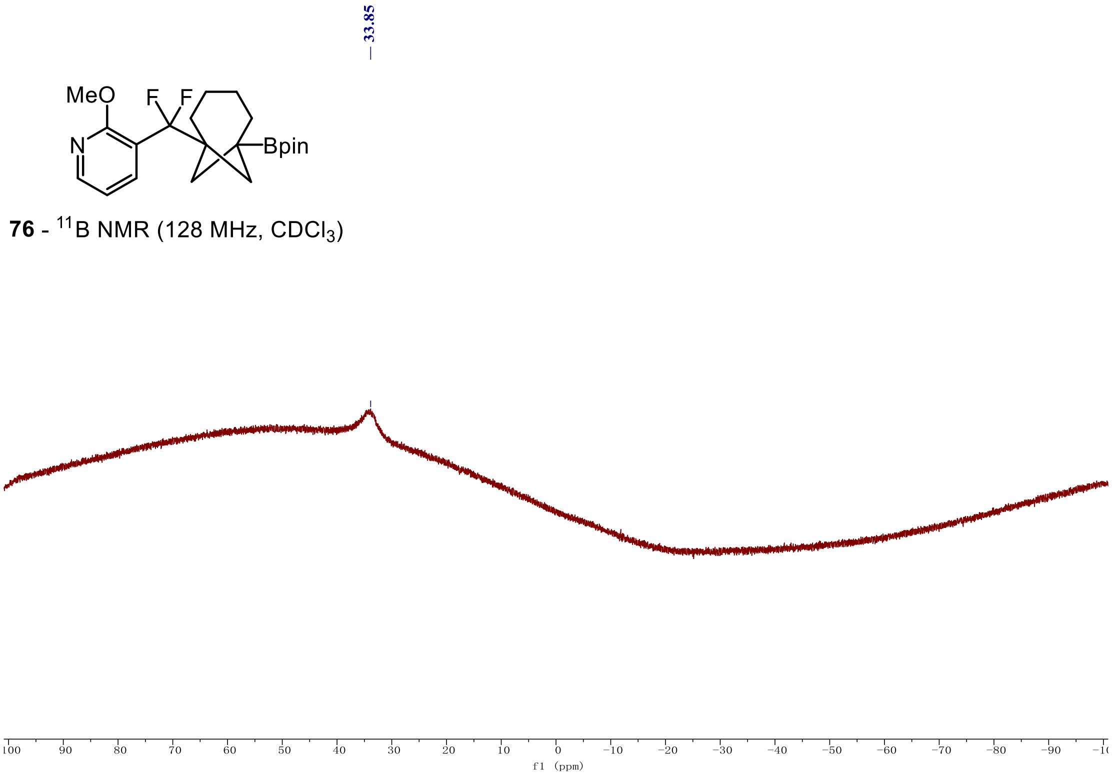

Supplementary Figure 256 ^{11}B NMR spectra of compound **76**

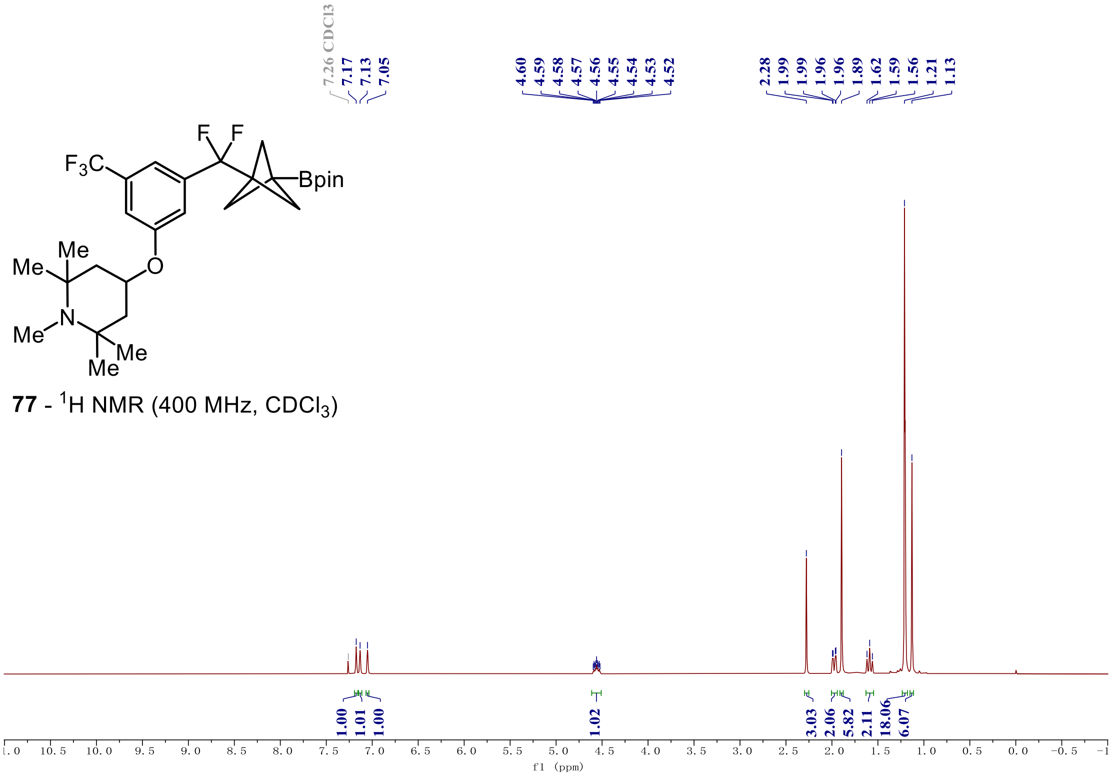

Supplementary Figure 257 ^{1}H NMR spectra of compound **77**

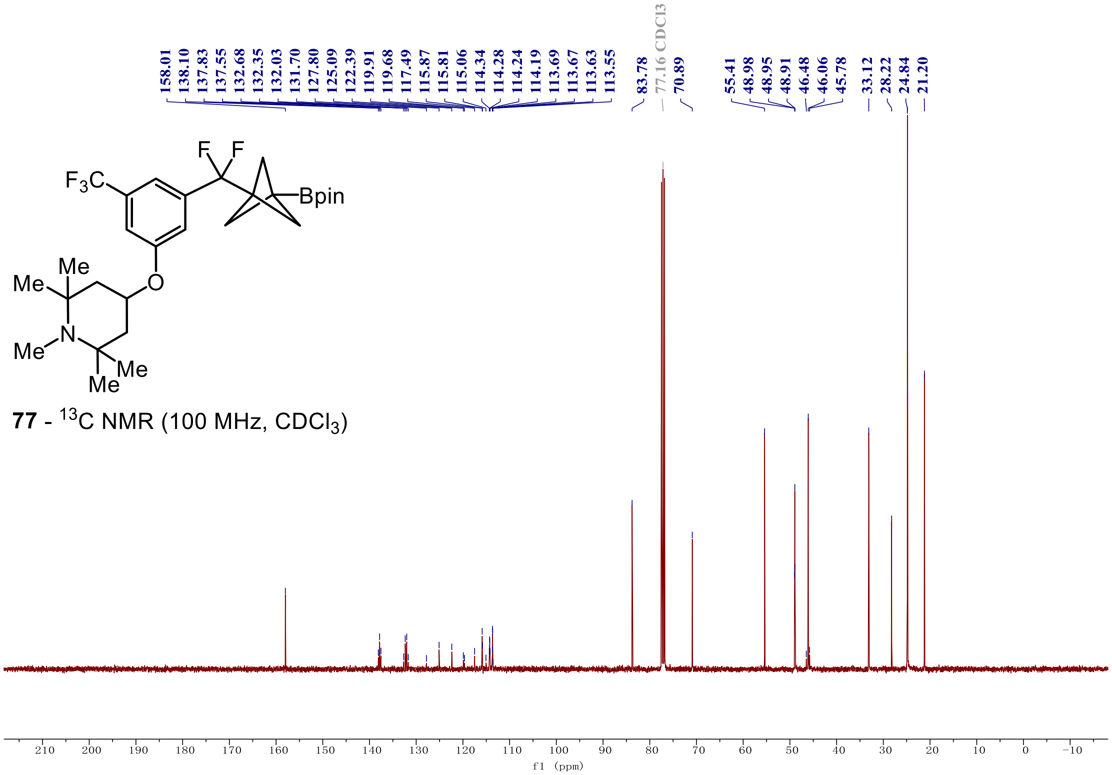

Supplementary Figure 258 ^{13}C NMR spectra of compound **77**

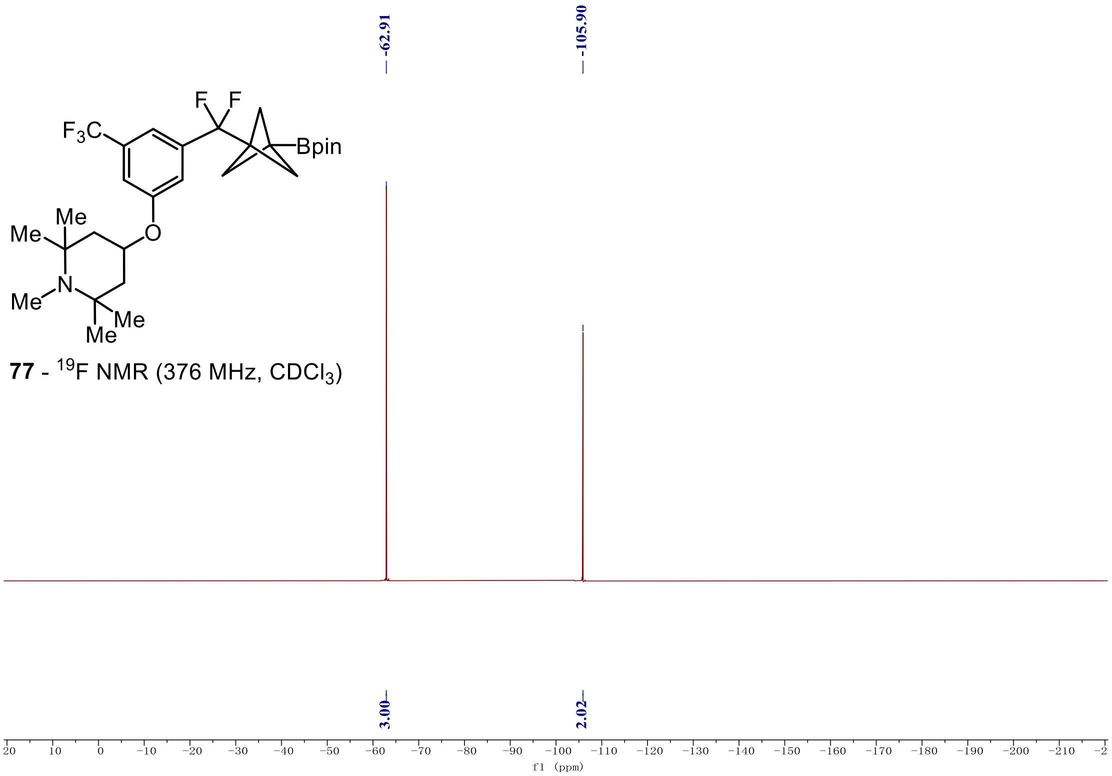

Supplementary Figure 259 ^{19}F NMR spectra of compound **77**

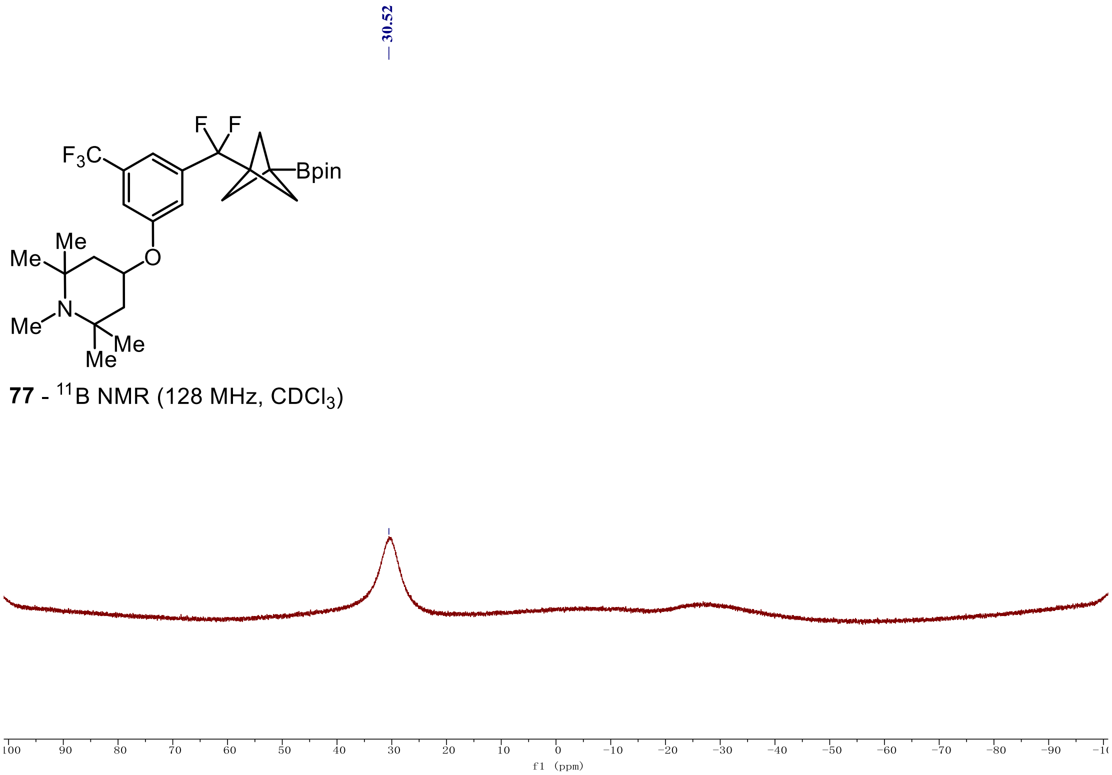

Supplementary Figure 260 ^{11}B NMR spectra of compound **77**

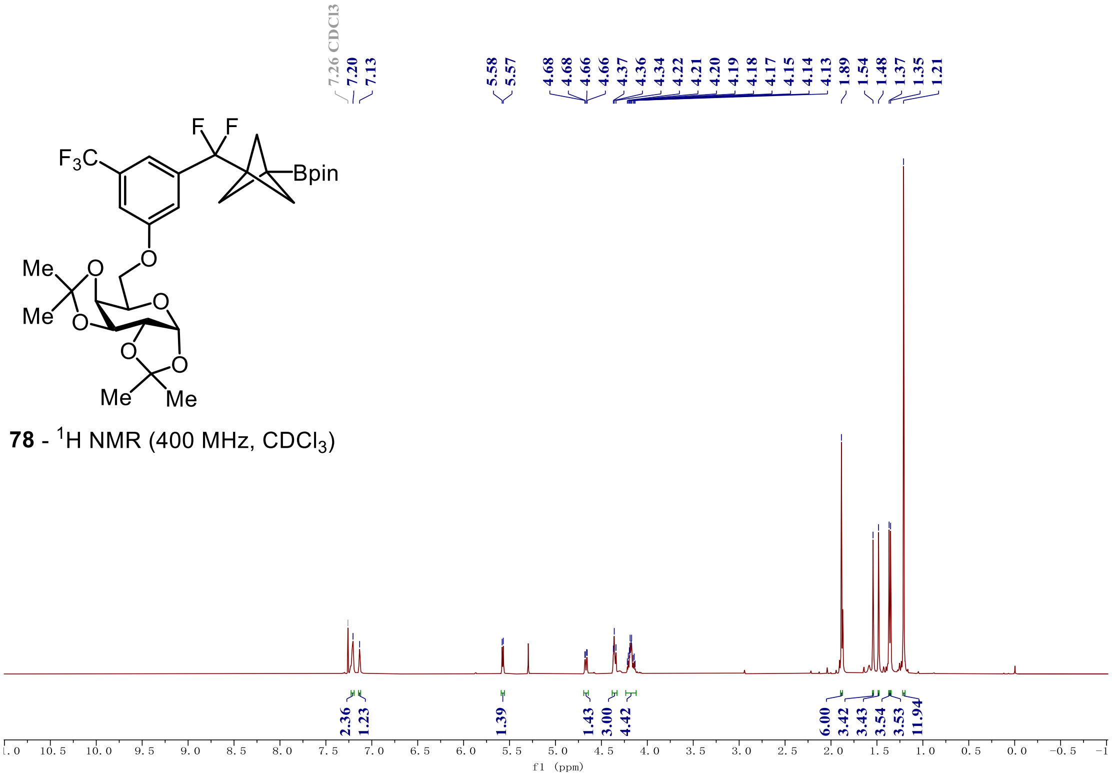

Supplementary Figure 261 ^{1}H NMR spectra of compound **78**

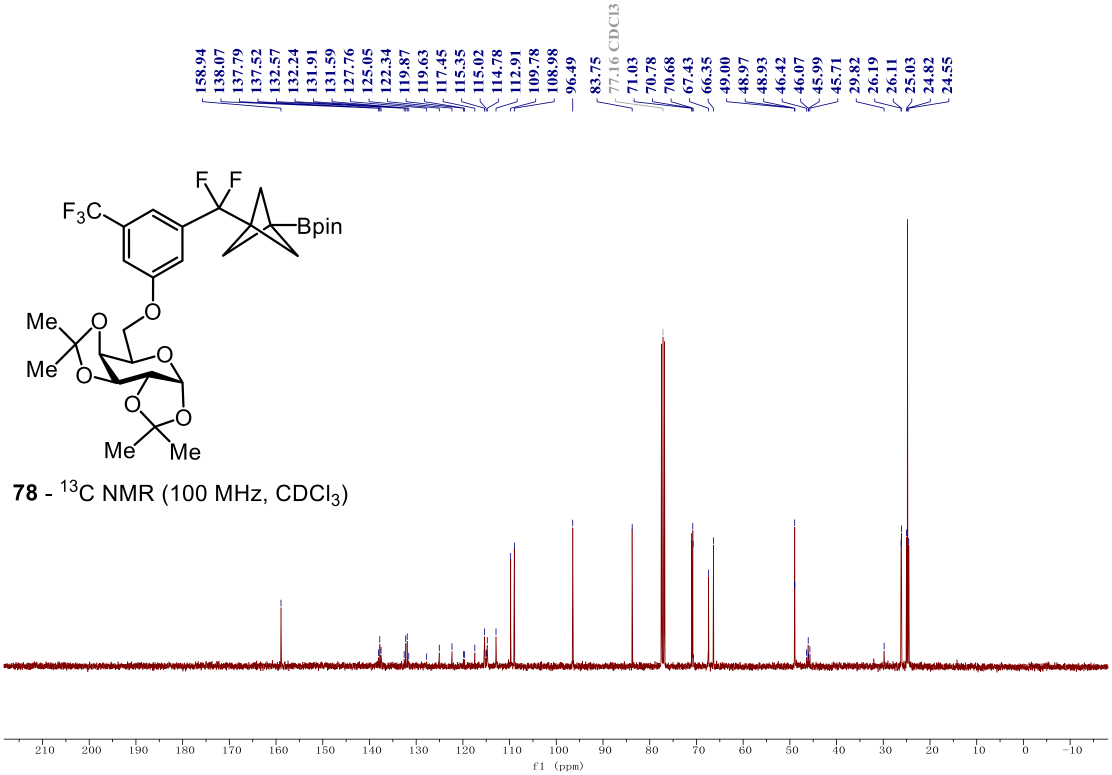

Supplementary Figure 262 ^{13}C NMR spectra of compound **78**

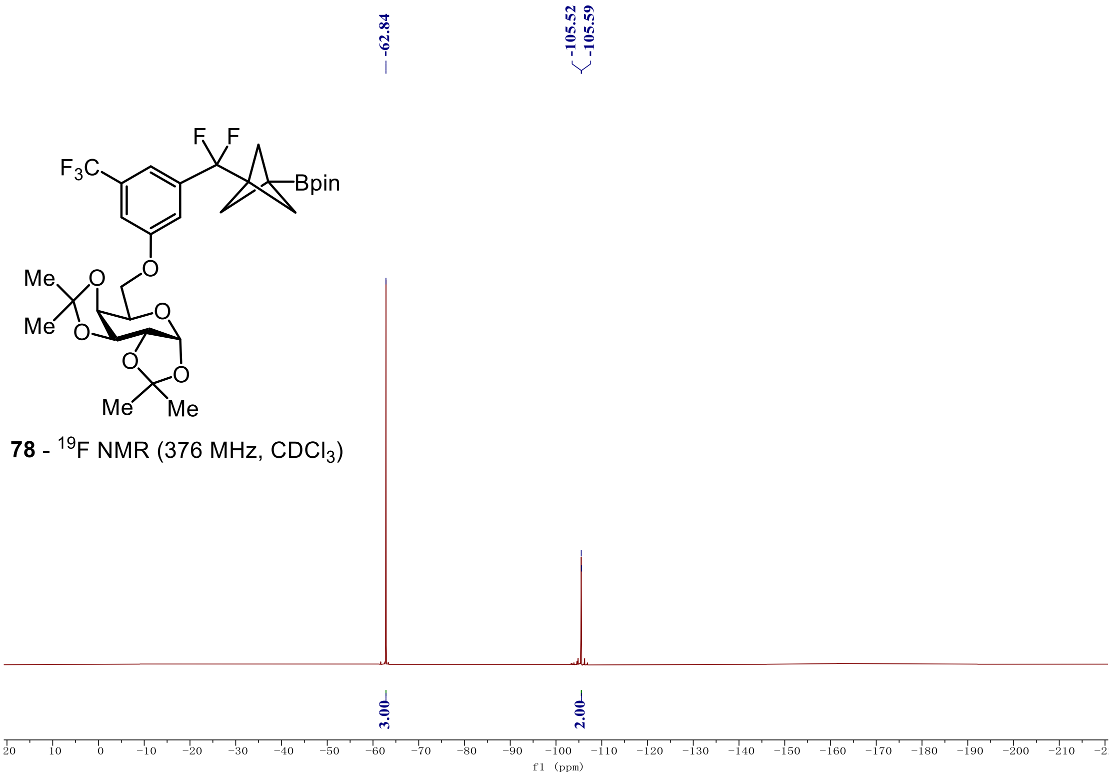

Supplementary Figure 263 ^{19}F NMR spectra of compound **78**

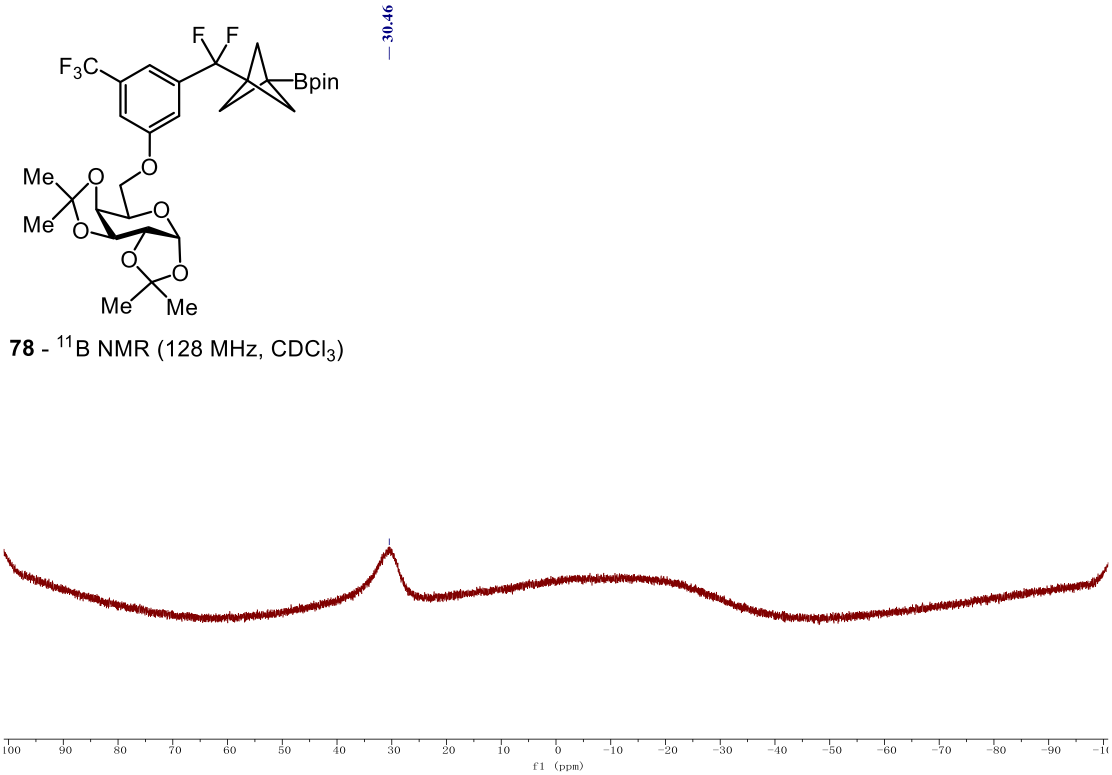

Supplementary Figure 264 ^{11}B NMR spectra of compound **78**

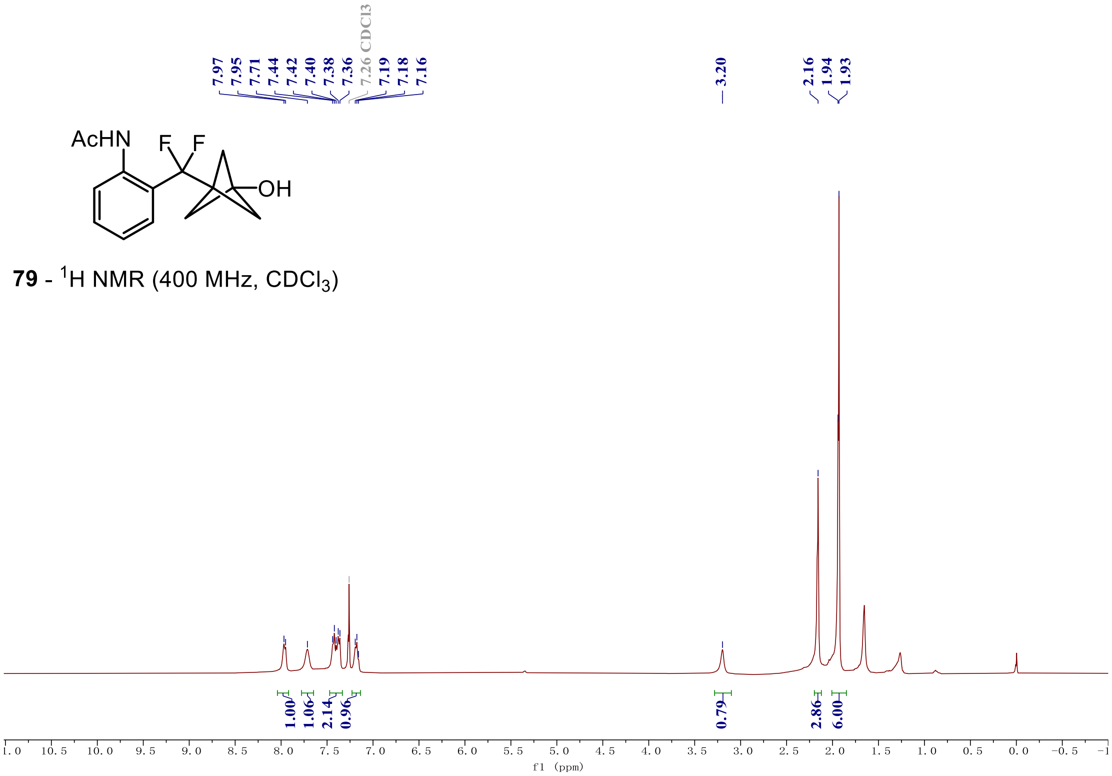

Supplementary Figure 265 ^{1}H NMR spectra of compound **79**

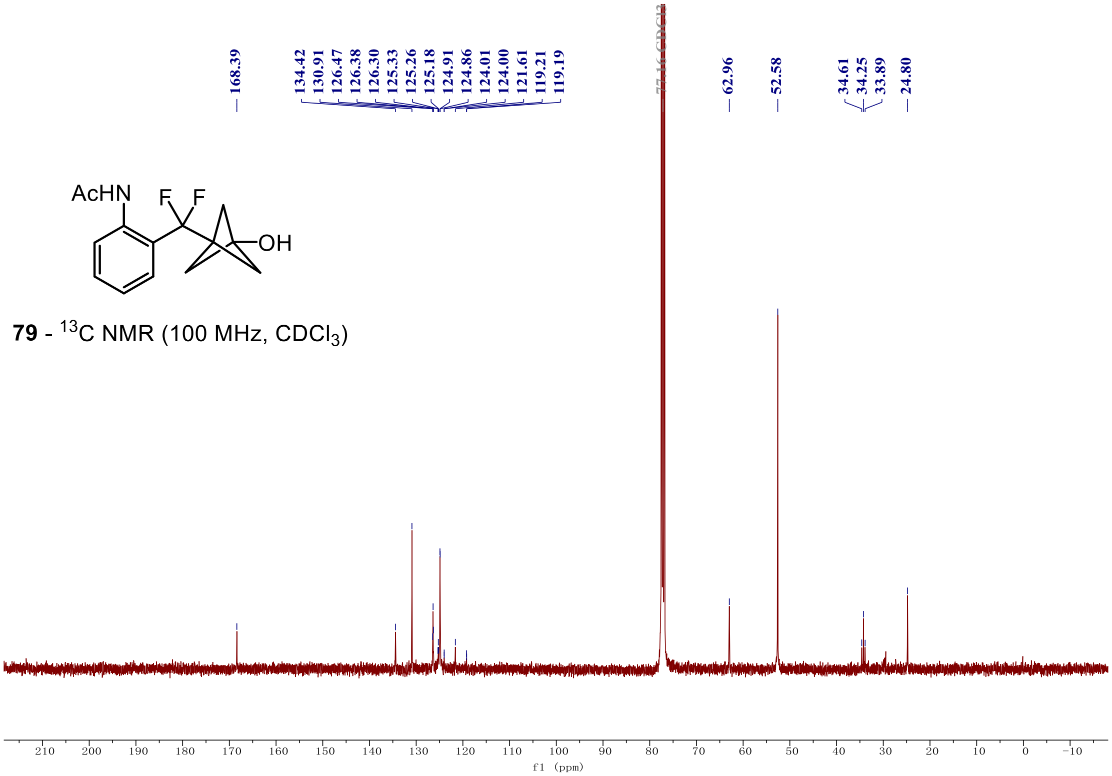

Supplementary Figure 266 ^{13}C NMR spectra of compound **79**

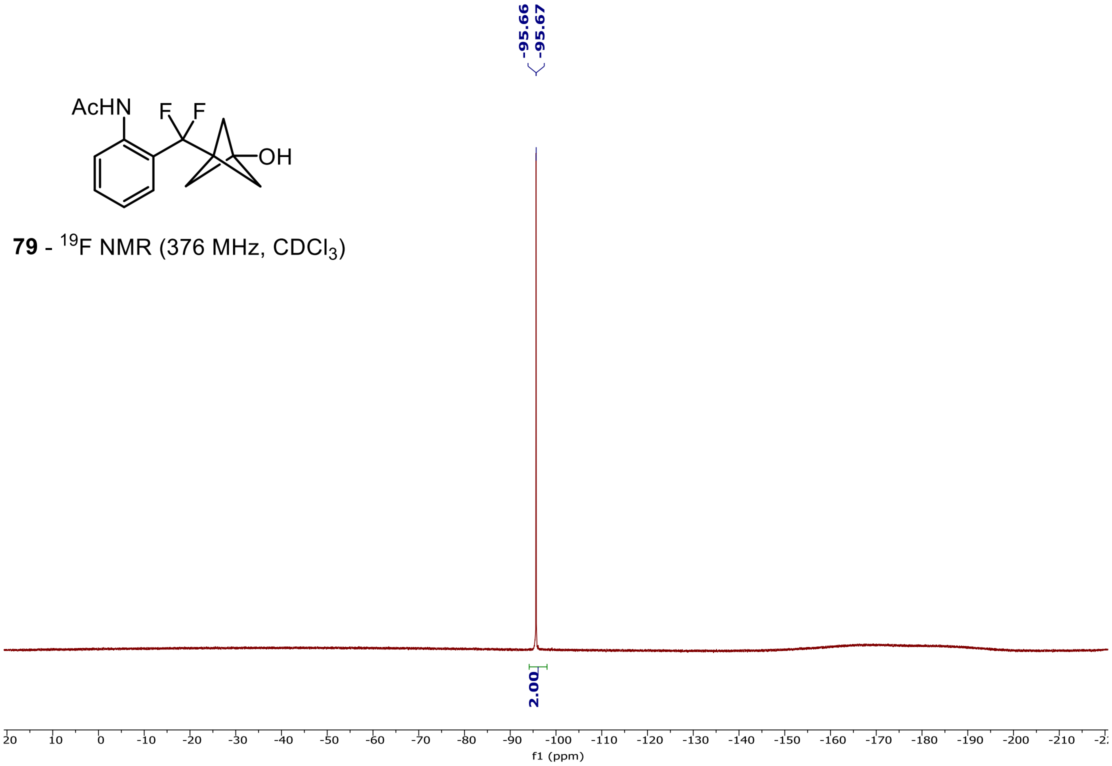

Supplementary Figure 267 ^{19}F NMR spectra of compound **79**

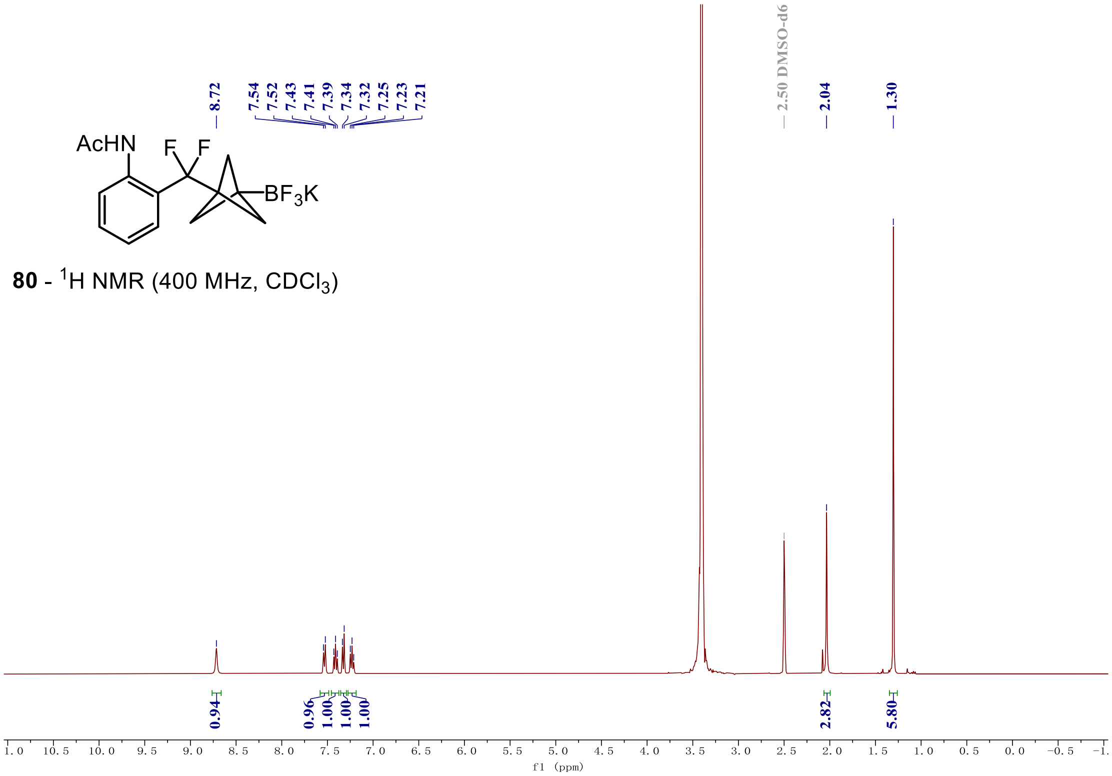

Supplementary Figure 268 ^{1}H NMR spectra of compound **80**

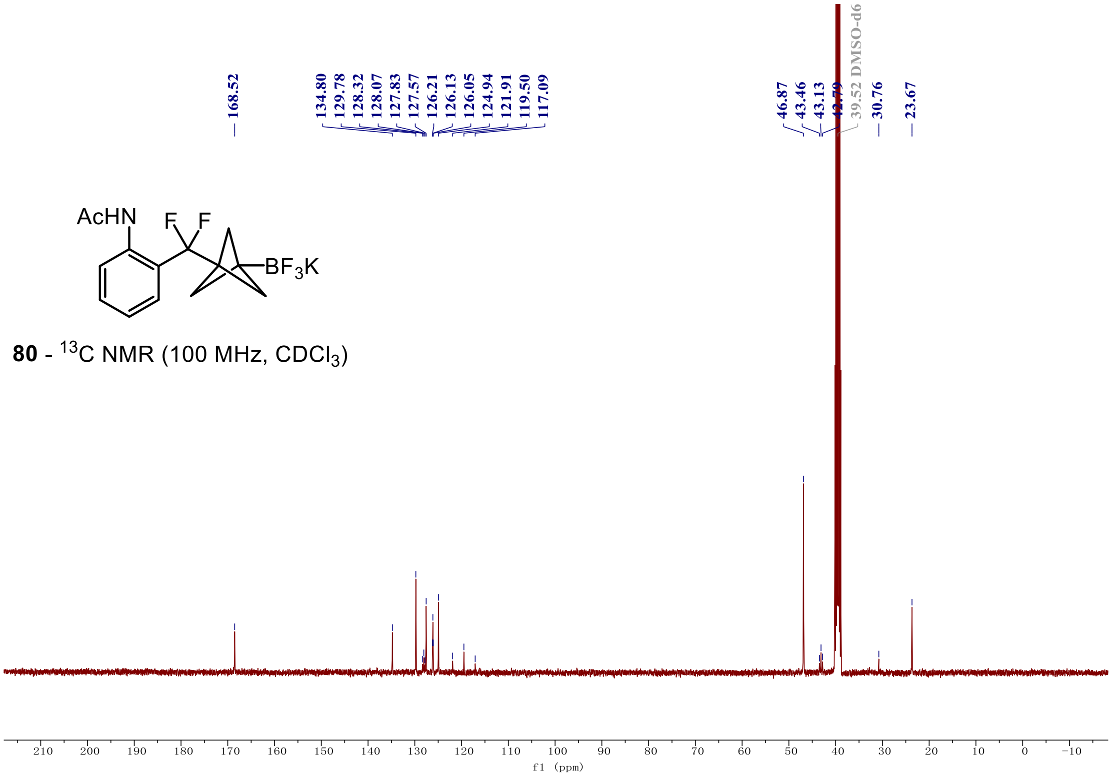

Supplementary Figure 269 ^{13}C NMR spectra of compound **80**

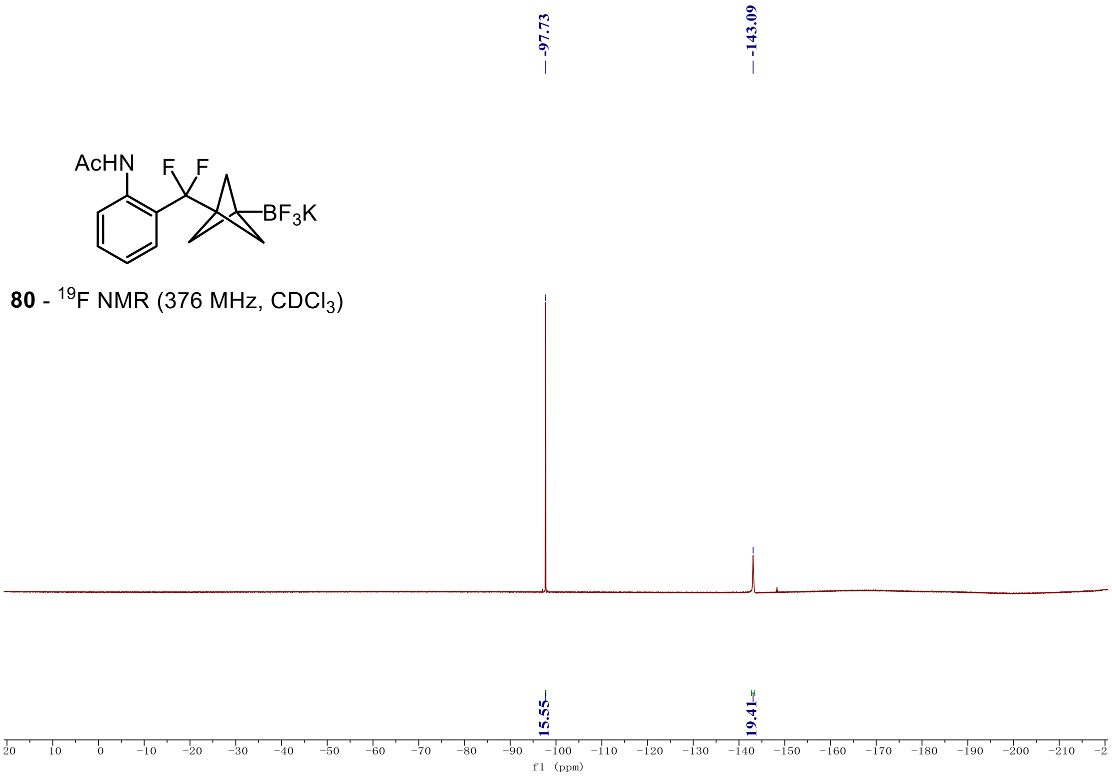

Supplementary Figure 270 ^{19}F NMR spectra of compound **80**

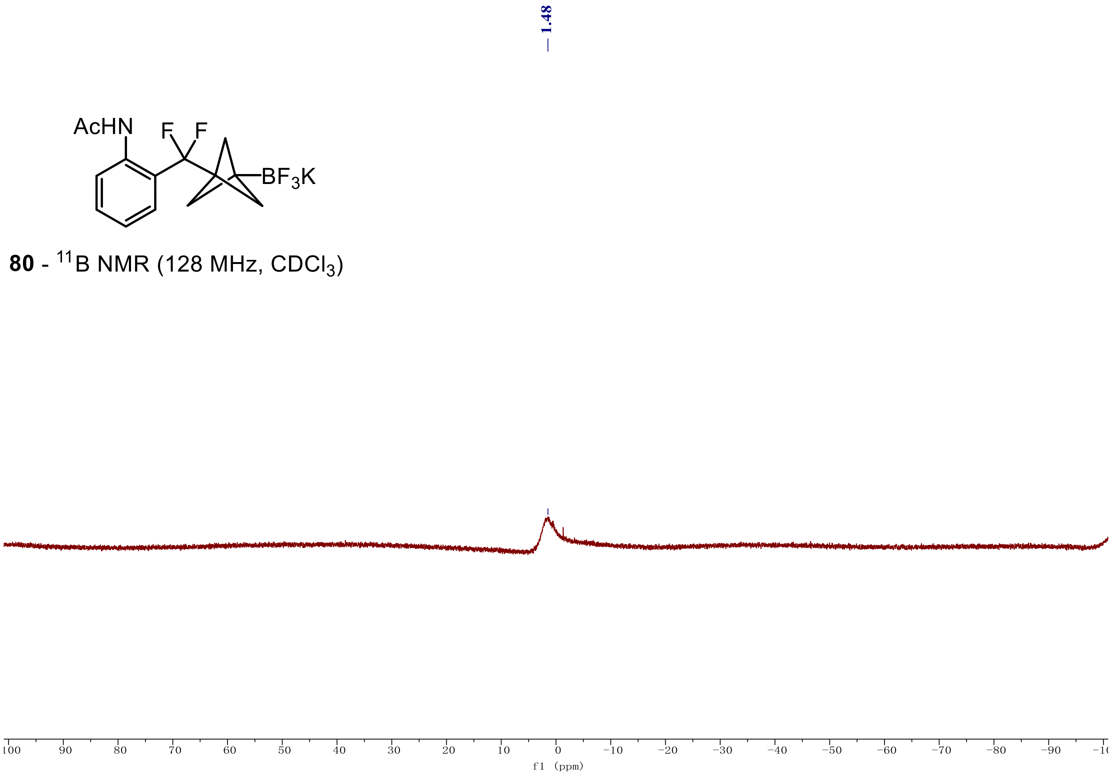

Supplementary Figure 271 ^{11}B NMR spectra of compound **80**

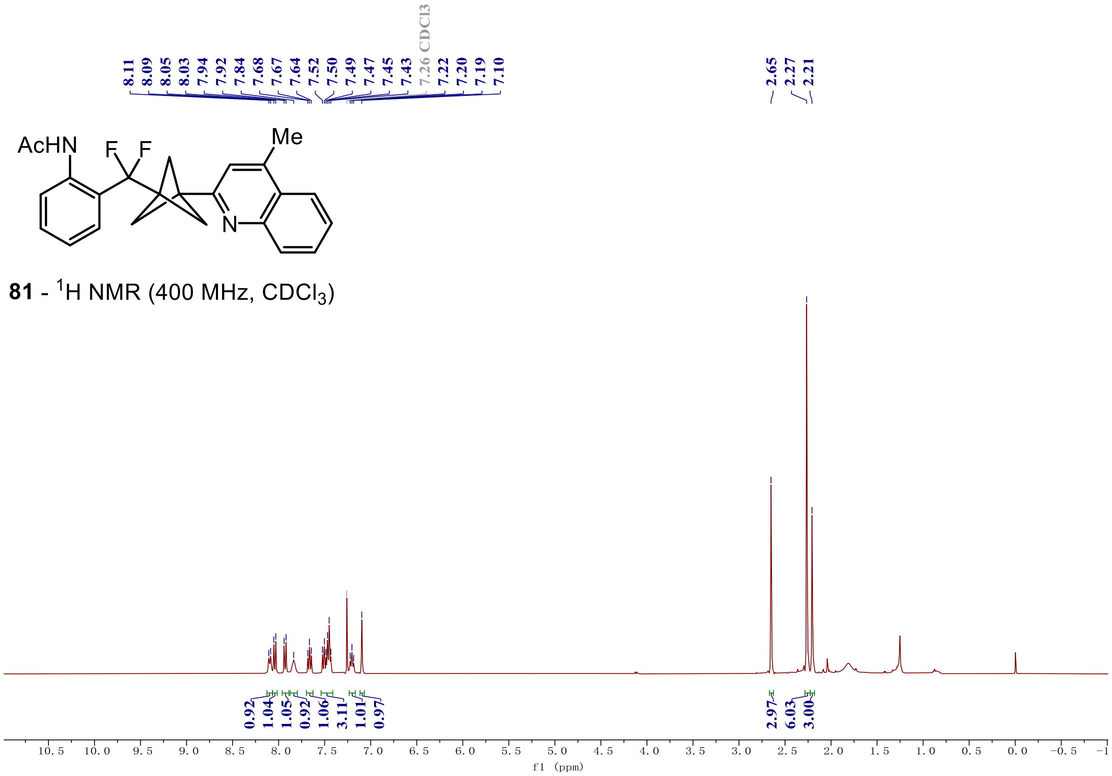

Supplementary Figure 272 ^{1}H NMR spectra of compound **81**

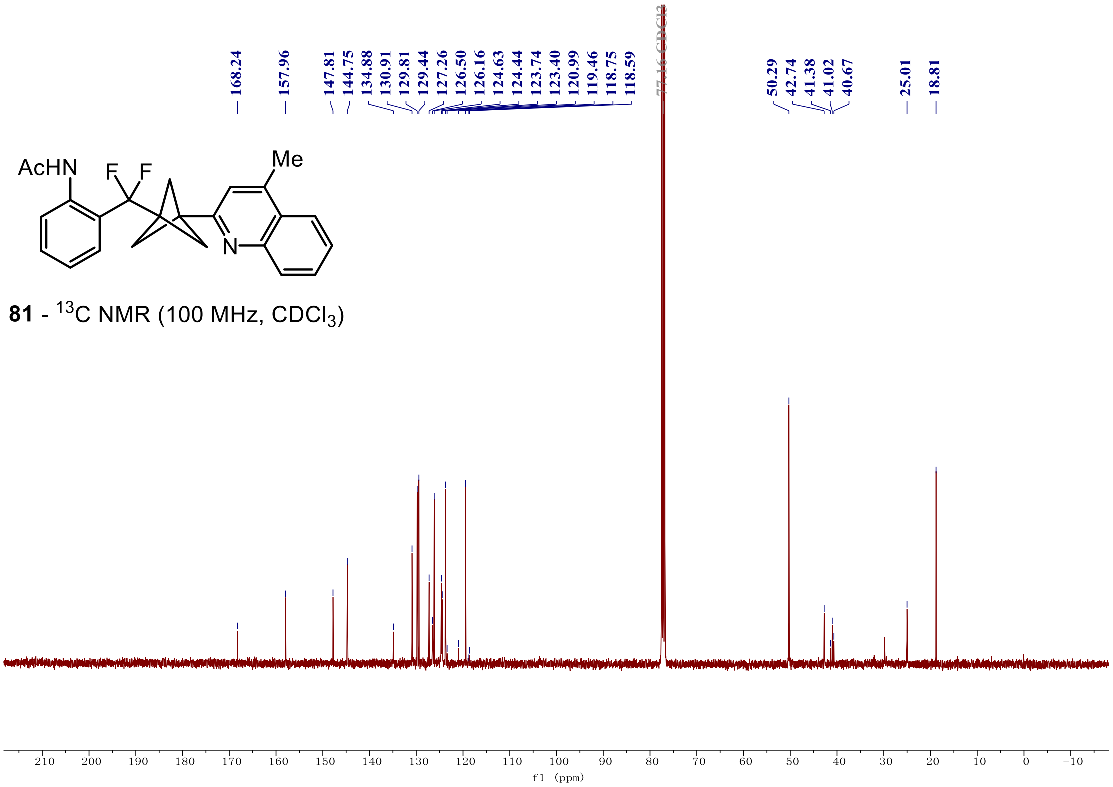

Supplementary Figure 273 ^{13}C NMR spectra of compound **81**

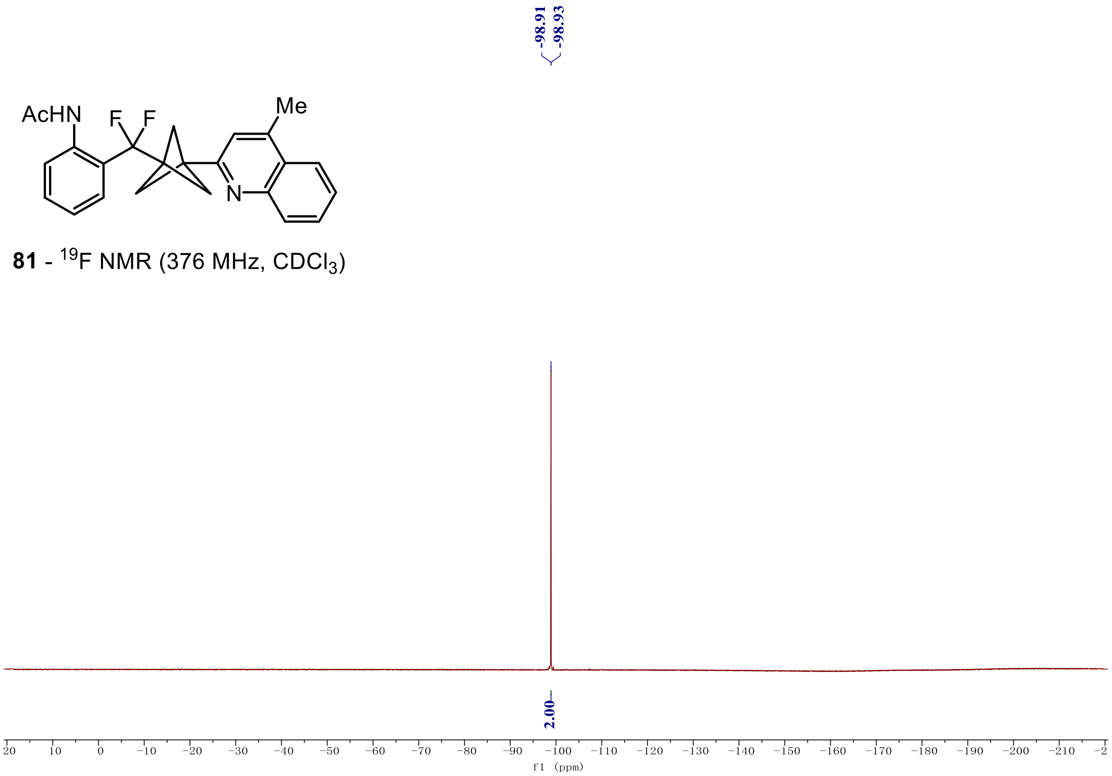

Supplementary Figure 274 ^{19}F NMR spectra of compound **81**

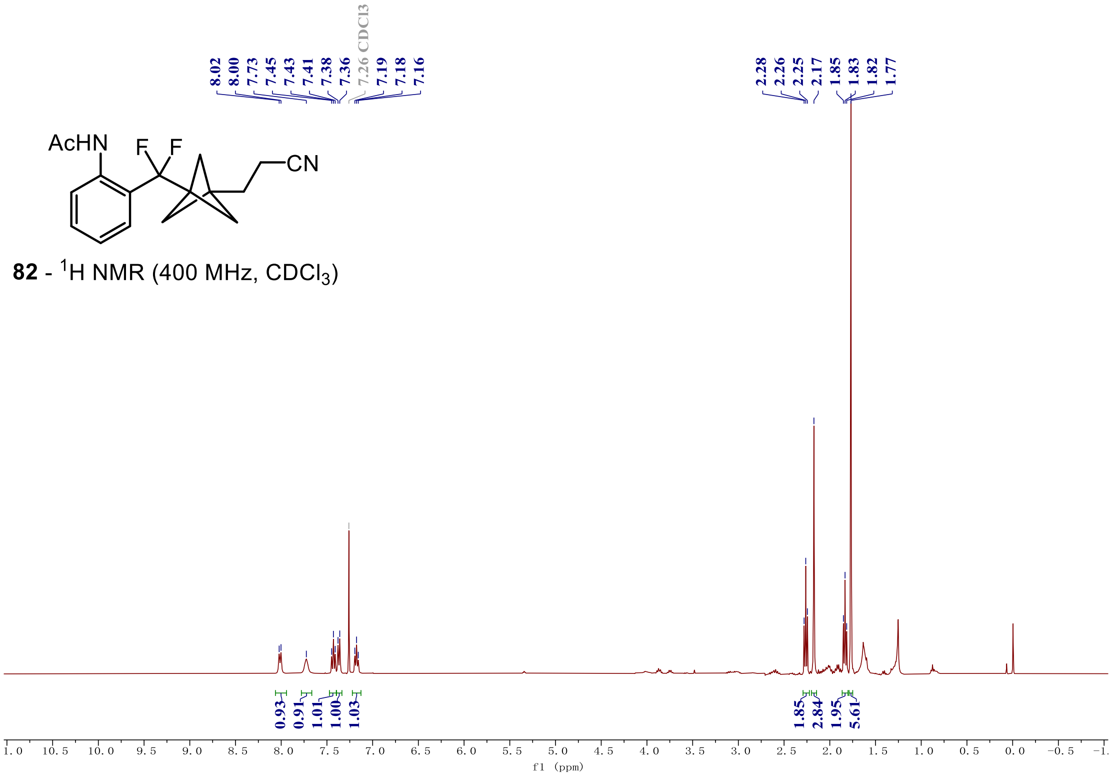

Supplementary Figure 275 ^{1}H NMR spectra of compound **82**

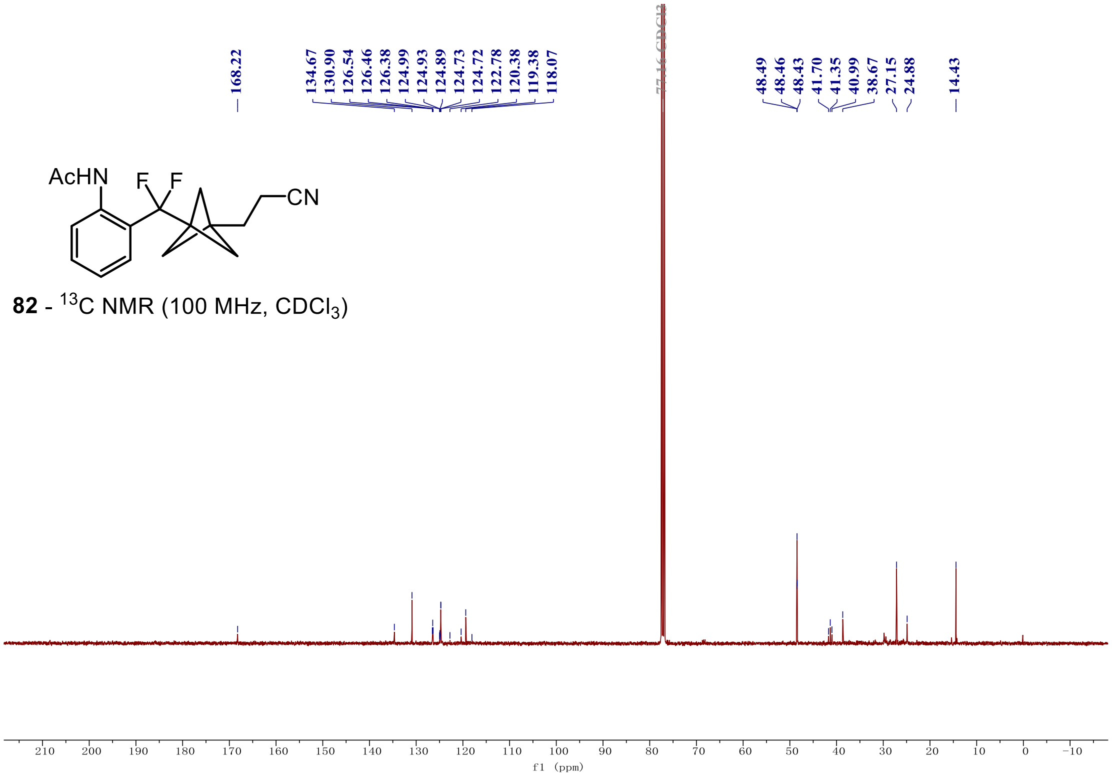

Supplementary Figure 276 ^{13}C NMR spectra of compound **82**

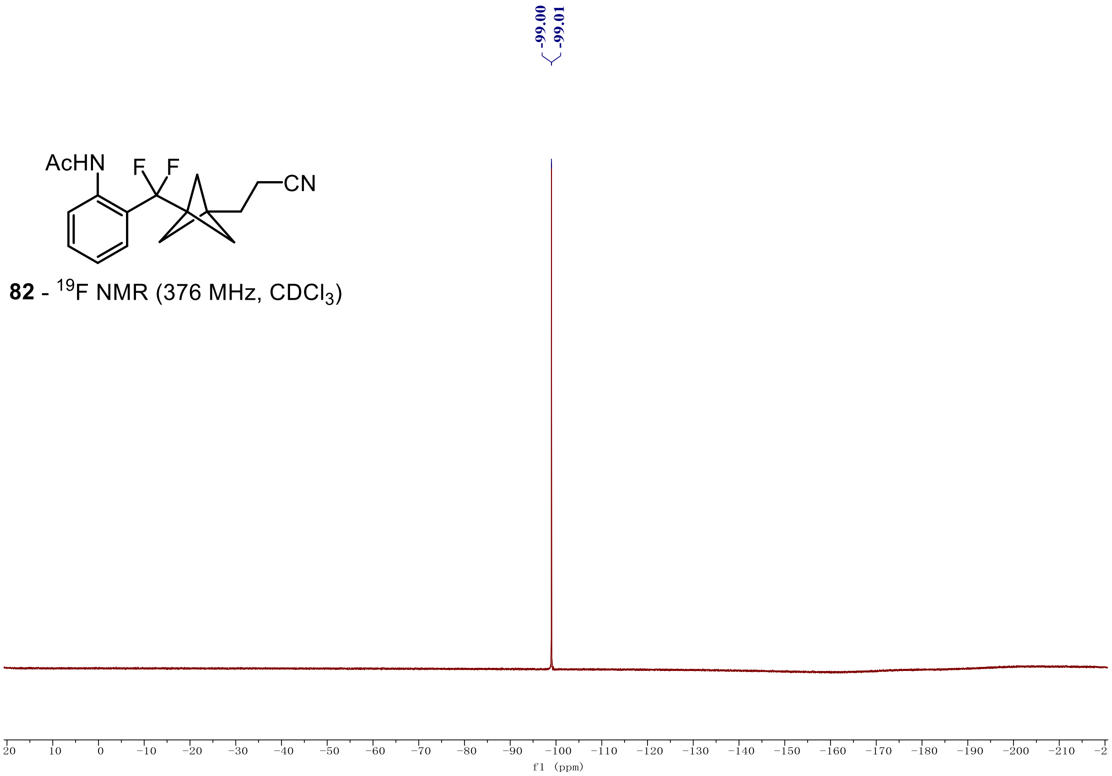

Supplementary Figure 277 ^{19}F NMR spectra of compound **82**

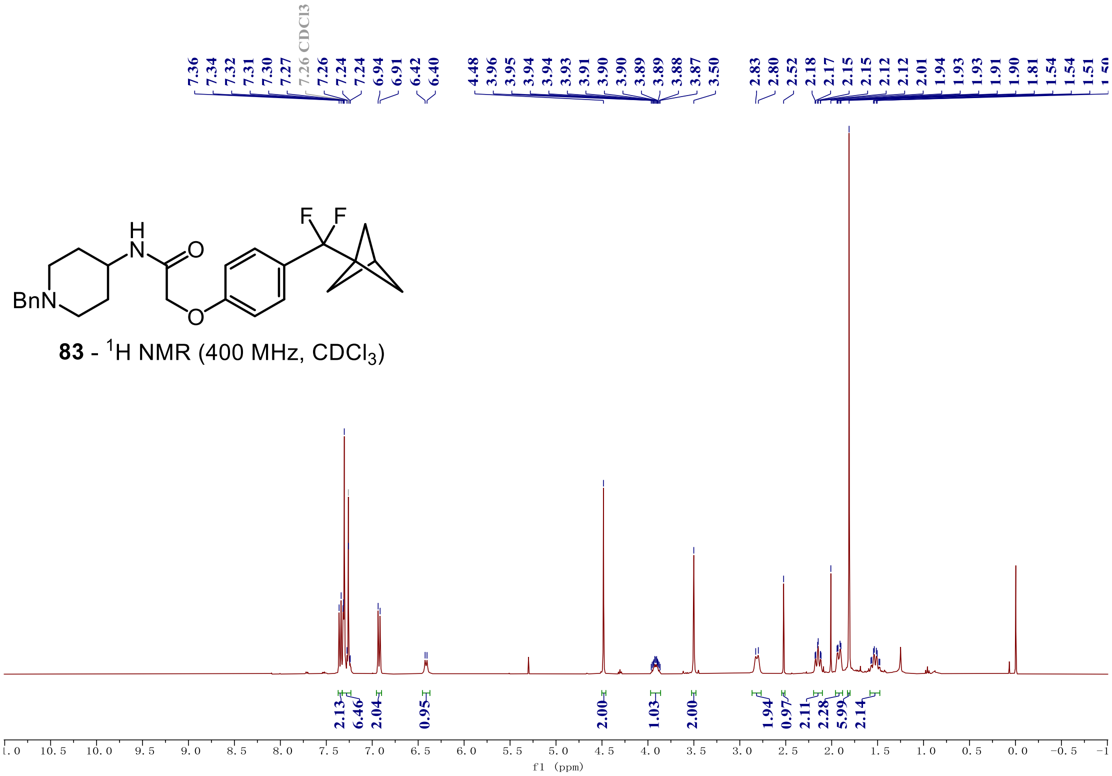

Supplementary Figure 278 ^{1}H NMR spectra of compound **83**

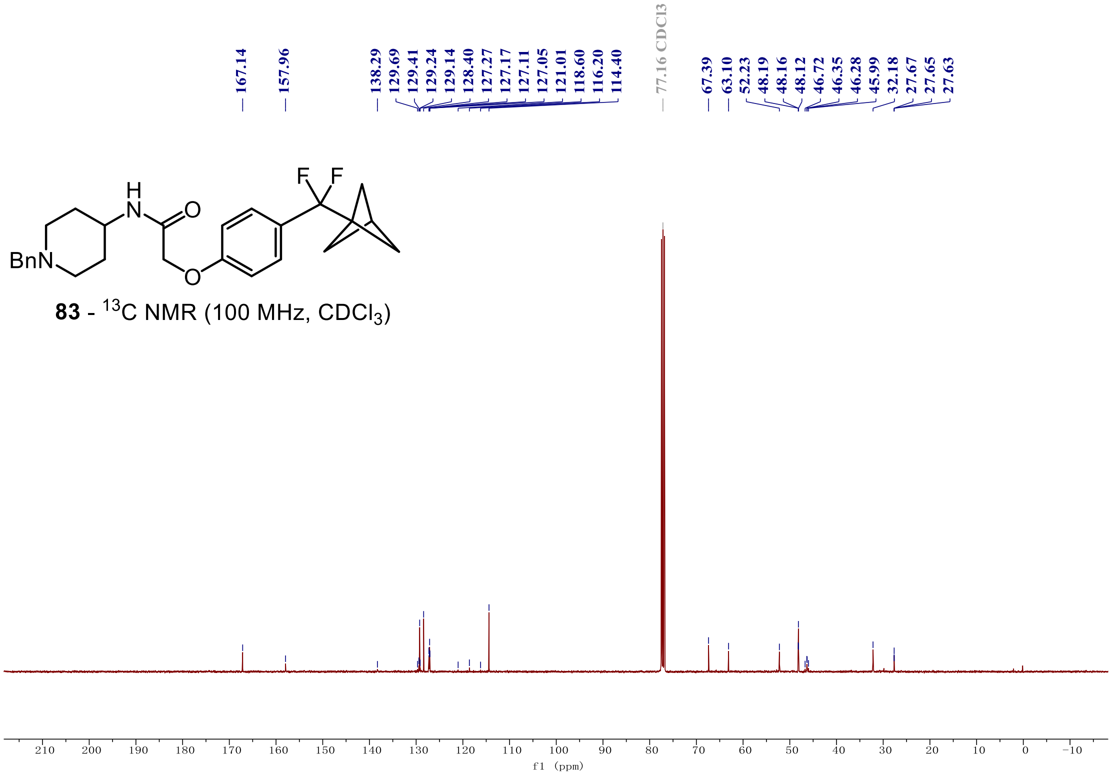

Supplementary Figure 279 ^{13}C NMR spectra of compound **83**

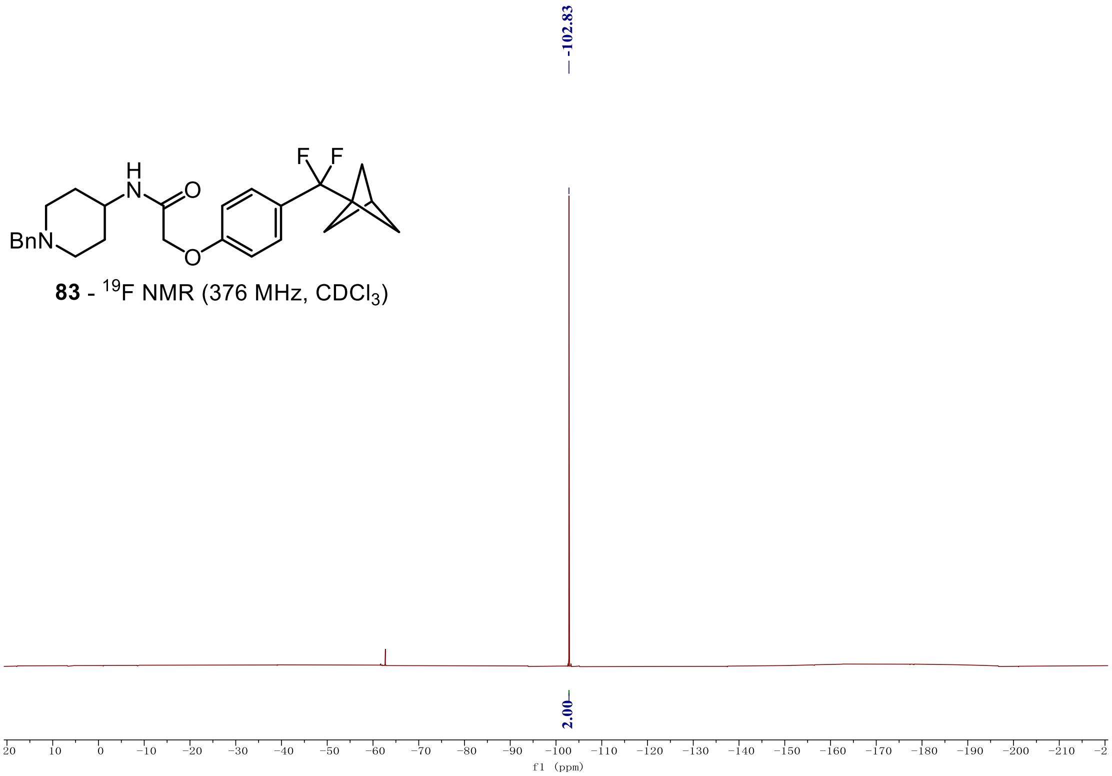

Supplementary Figure 280 ^{19}F NMR spectra of compound **83**

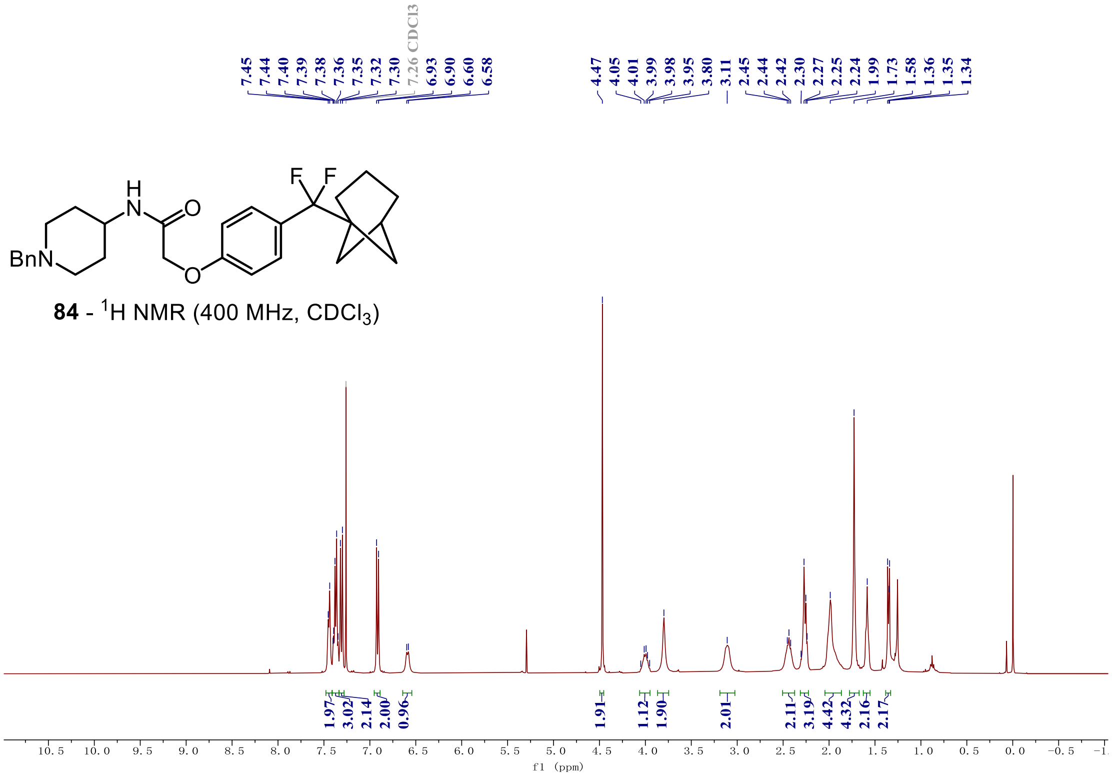

Supplementary Figure 281 ^{1}H NMR spectra of compound **84**

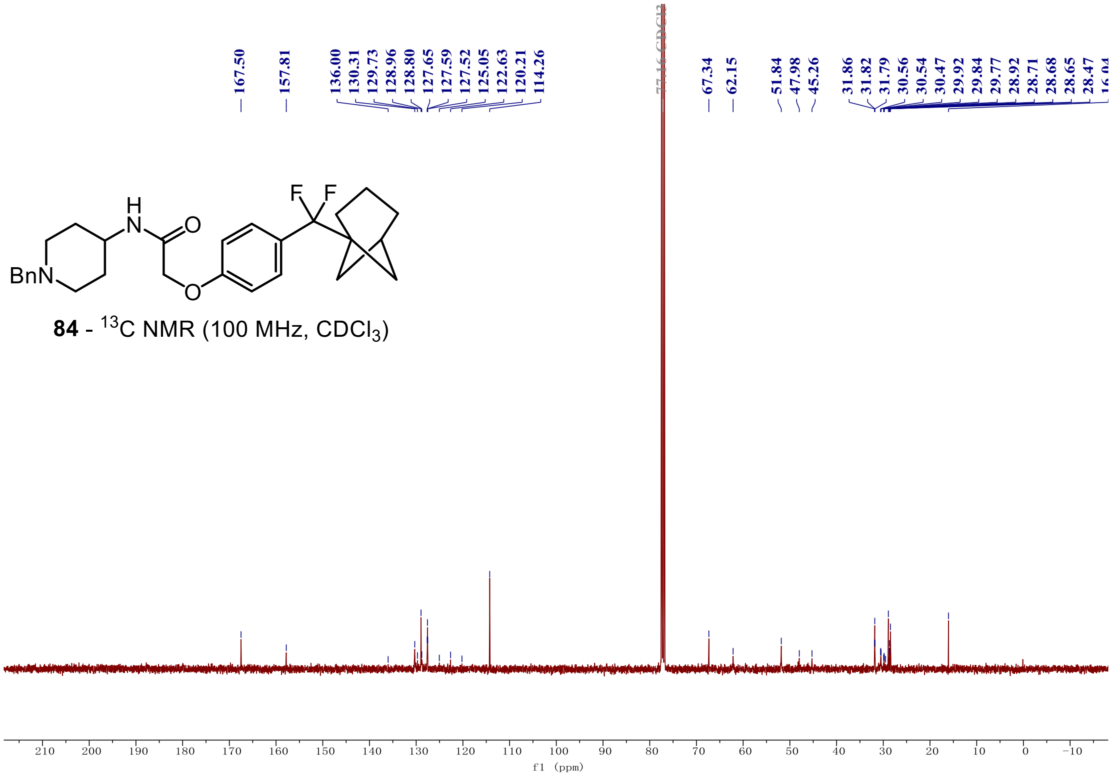

Supplementary Figure 282 ^{13}C NMR spectra of compound **84**

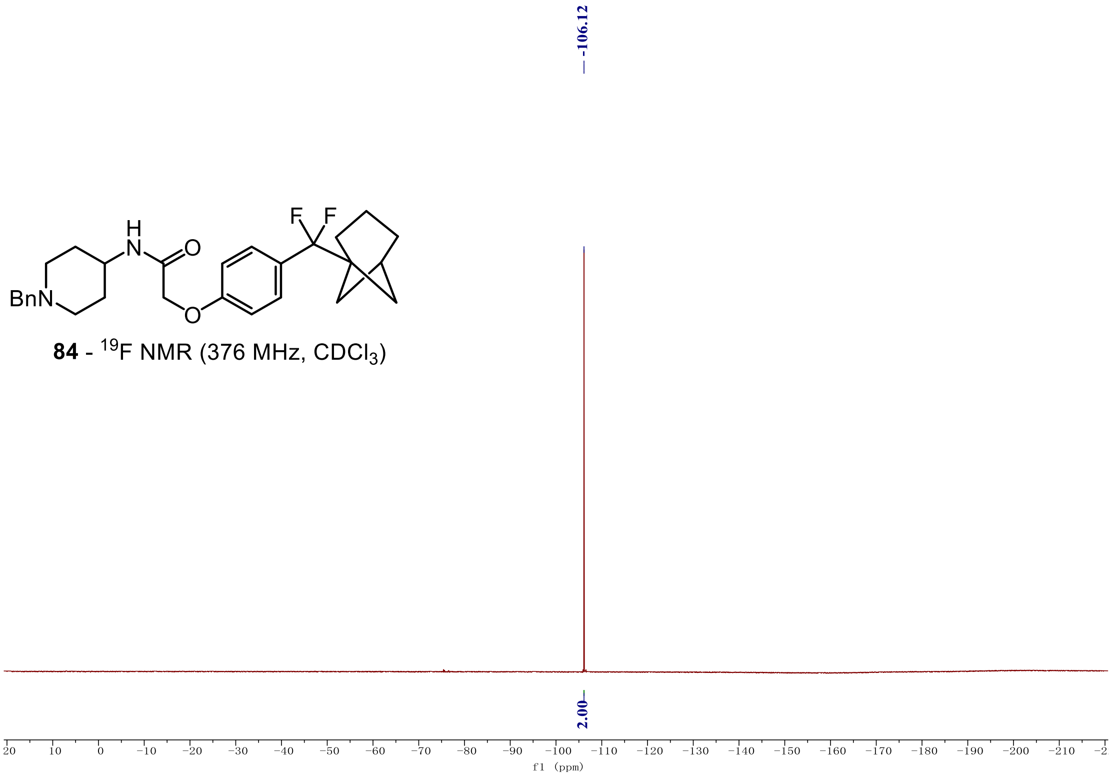

Supplementary Figure 283 ^{19}F NMR spectra of compound **84**

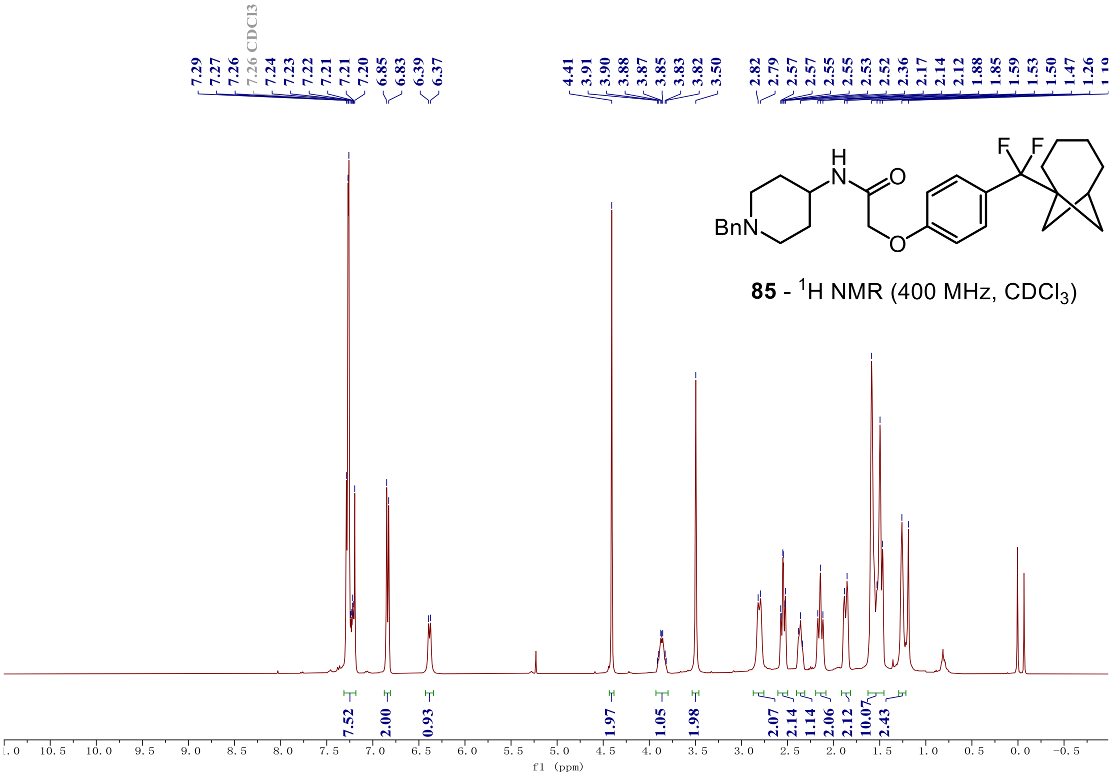

Supplementary Figure 284 ^{1}H NMR spectra of compound **85**

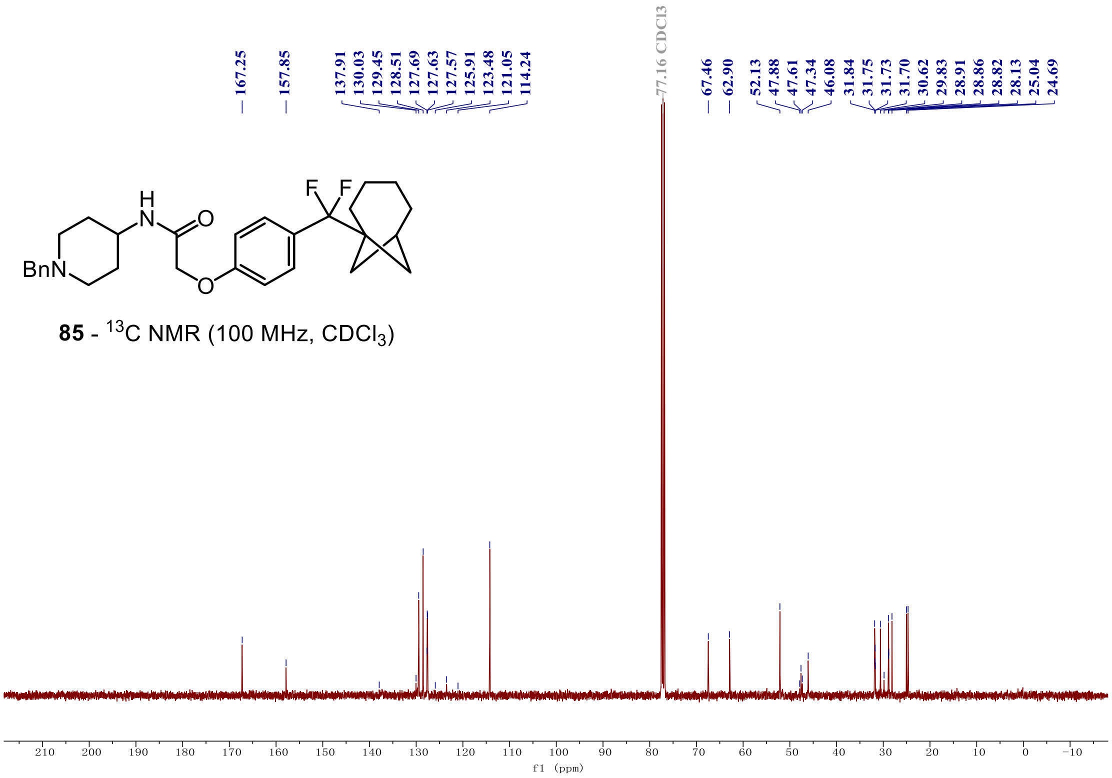

Supplementary Figure 285 ^{13}C NMR spectra of compound **85**

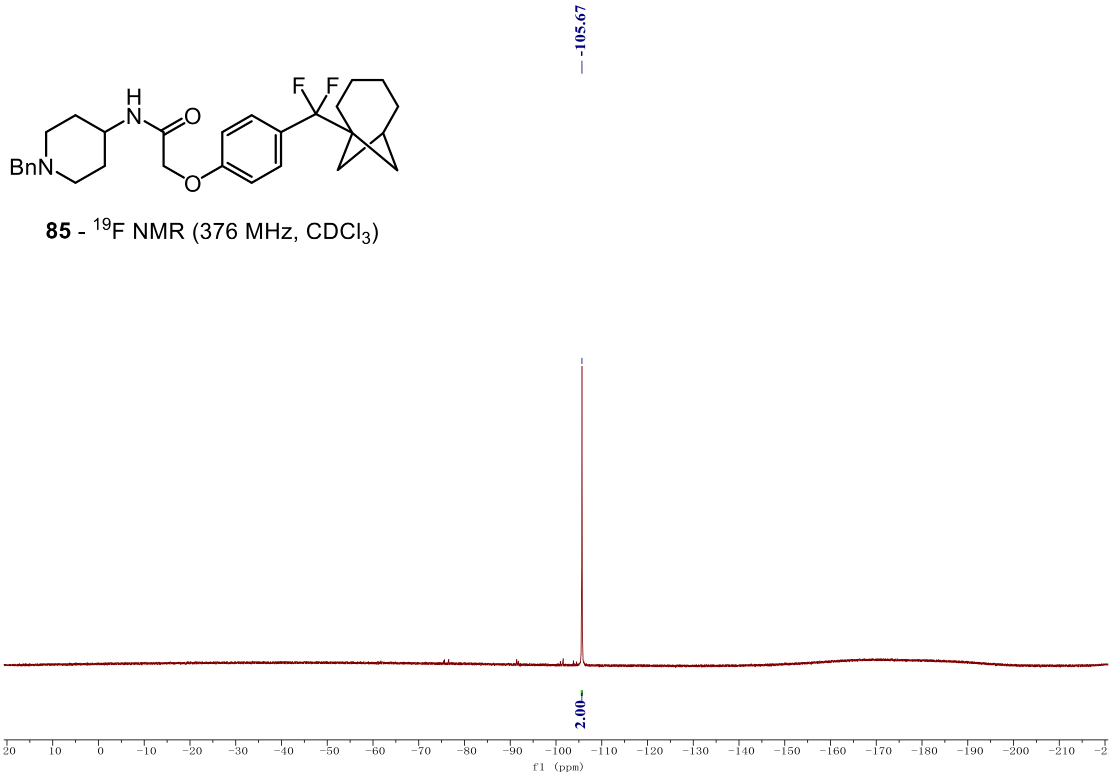

Supplementary Figure 286 ^{19}F NMR spectra of compound **85**
